# Comprehensive Comparative Analysis of Cholesterol Catabolic Genes/Proteins in Mycobacterial Species

**DOI:** 10.3390/ijms20051032

**Published:** 2019-02-27

**Authors:** Rochelle van Wyk, Mari van Wyk, Samson Sitheni Mashele, David R. Nelson, Khajamohiddin Syed

**Affiliations:** 1 Unit for Drug Discovery Research, Department of Health Sciences, Faculty of Health and Environmental Sciences, Central University of Technology, Bloemfontein 9300, Free State, South Africa; rochellevanwyk0@gmail.com (R.v.W.); marivanwyk764@gmail.com (M.v.W.); smashele@cut.ac.za (S.S.M.); 2 Department of Microbiology, Immunology and Biochemistry, University of Tennessee Health Science Center, Memphis, TN 38163, USA; drnelson1@gmail.com; 3 Department of Biochemistry and Microbiology, Faculty of Science and Agriculture, University of Zululand, KwaDlangezwa 3886, South Africa

**Keywords:** Cholesterol catabolism, Cholesterol catabolic genes/proteins, Comparative analysis, in silico analysis, *Mycobacterium tuberculosis*, *Mycobacterium tuberculosis* complex, Tuberculosis, *Mycobacterium chelonae-abscessus* complex, *Mycobacterium avium* complex, Mycobacteria causing leprosy, Non-tuberculous mycobacteria, Saprophytes, Software tool

## Abstract

In dealing with *Mycobacterium tuberculosis*, the causative agent of the deadliest human disease—tuberculosis (TB)—utilization of cholesterol as a carbon source indicates the possibility of using cholesterol catabolic genes/proteins as novel drug targets. However, studies on cholesterol catabolism in mycobacterial species are scarce, and the number of mycobacterial species utilizing cholesterol as a carbon source is unknown. The availability of a large number of mycobacterial species’ genomic data affords an opportunity to explore and predict mycobacterial species’ ability to utilize cholesterol employing *in silico* methods. In this study, comprehensive comparative analysis of cholesterol catabolic genes/proteins in 93 mycobacterial species was achieved by deducing a comprehensive cholesterol catabolic pathway, developing a software tool for extracting homologous protein data and using protein structure and functional data. Based on the presence of cholesterol catabolic homologous proteins proven or predicted to be either essential or specifically required for the growth of *M. tuberculosis* H37Rv on cholesterol, we predict that among 93 mycobacterial species, 51 species will be able to utilize cholesterol as a carbon source. This study’s predictions need further experimental validation and the results should be taken as a source of information on cholesterol catabolism and genes/proteins involved in this process among mycobacterial species.

## 1. Introduction

Tuberculosis (TB), is a chronic infectious disease caused by *Mycobacterium tuberculosis*, and is one of the leading causes of death worldwide, killing an estimated two million people annually [1,2]. It is estimated that one third of the world’s population (approximately two billion people) is infected with this highly pathogenic organism [3]. Once it has entered the human body, and after ingestion by macrophages, this intracellular pathogen can survive in a modified phagosome and cause latent infection for years and sometimes decades without any symptoms [4]. Tubercle bacilli can persist in this dormant state, from which they may be reactivated and cause TB [4]. The reactivation of latent phase *M. tuberculosis* into the active phase is observed among people whose immune systems are weakened by HIV infection, by immunosuppressive drugs or by malnutrition and/or aging [5]. Over the past decades, the threat of TB has become greater with the development of single-drug resistance to multiple-drug-resistant strains and, recently, the surfacing of extensive drug resistance that threatens to compromise the available drugs severely [6]. With the documentation of total drug-resistant strains [6], along with the insufficiency of new drug targets, we clearly need more research to discover novel drug targets.

*M. tuberculosis* can infect, grow and survive in the harsh environment of the macrophage and other host cells using mechanisms that are not yet well understood [7,8]. Host cholesterol levels are thought to play a role in the development of *M. tuberculosis* infection [9], with high levels of cholesterol in the diet significantly enhancing the bacterial burden in the lung [10] and impairing immunity to *M. tuberculosis* [11]. Specifically, cholesterol is required for the phagocytosis of mycobacteria into macrophages [12,13], where they bind and enter phagocytes through cholesterol-enriched membrane microdomains (lipid rafts) [14]. In addition, cholesterol plays a crucial role in the mediation of the infected phagosomal association of tryptophan–aspartate-containing coat protein [15], leading to the inhibition of phagosome–lysosome fusion [16]. This experimental evidence suggests an important role for cholesterol during *M. tuberculosis* infection and persistence.

Research studies have demonstrated that *M. tuberculosis* can grow using cholesterol as the sole carbon and energy source [17]. Therefore, cholesterol has recently been identified as an important lipid for mycobacterial infection [18,19]. The relatively abundant cholesterol distributed in host cells is an important growth substrate for these bacteria in different infection stages (e.g., intracellular growth or intracellular persistence) [20]. *M. tuberculosis* growing in human cells appears to obtain energy from host lipids rather than other nutrients such as carbohydrates [21].

Considering the above facts and recent momentum on cholesterol catabolism as a therapeutic target in *M. tuberculosis*, Ouellet and co-workers [19] suggest that more research needs to be done to understand cholesterol catabolism in mycobacterial species. Furthermore, performing laboratory experiments is laborious and time- and money-consuming, since each mycobacterial species has a different lifestyle and different culture conditions. Taking advantage of the genome sequencing of many mycobacterial species, this study is aimed at performing comprehensive comparative analysis of the genes/proteins involved in cholesterol catabolism and predicting mycobacterial species’ ability to utilize cholesterol as a carbon source.

## 2. Results and Discussion

### 2.1. Deducing Cholesterol Catabolic Pathway in M. Tuberculosis H37Rv

**Figure 1:**
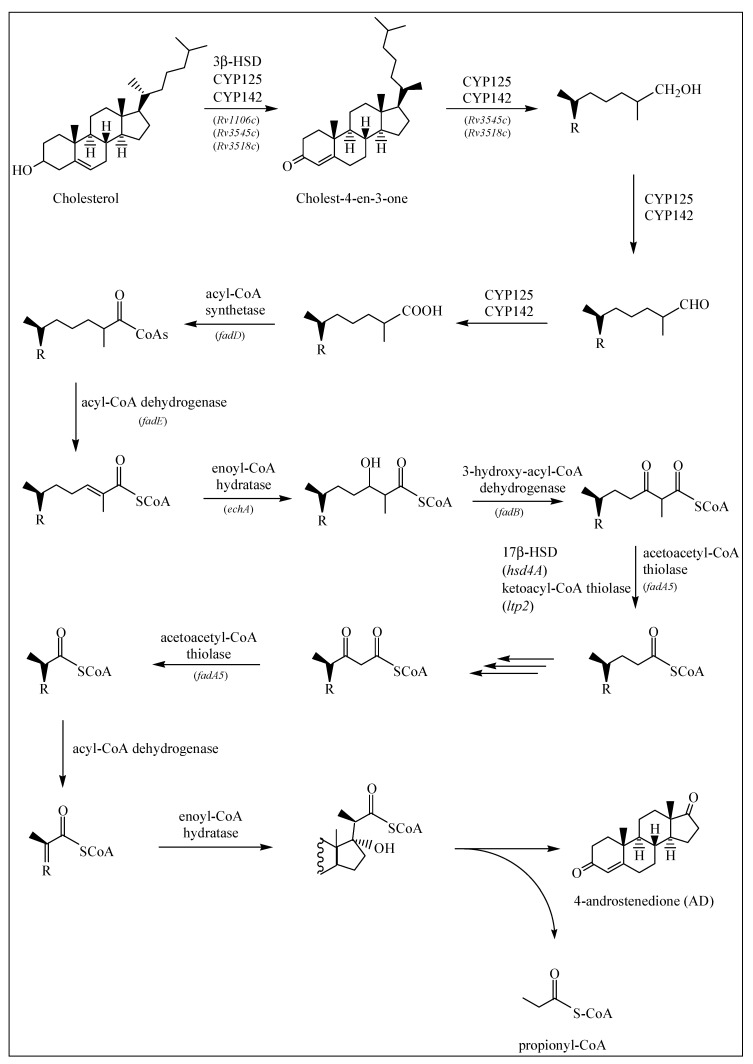
Cholesterol side chain degradation as described in Section 2.1.1. If known, the enzymes involved in each reaction are depicted by arrows, along with the gene coding for the specific enzyme.

**Figure 2:**
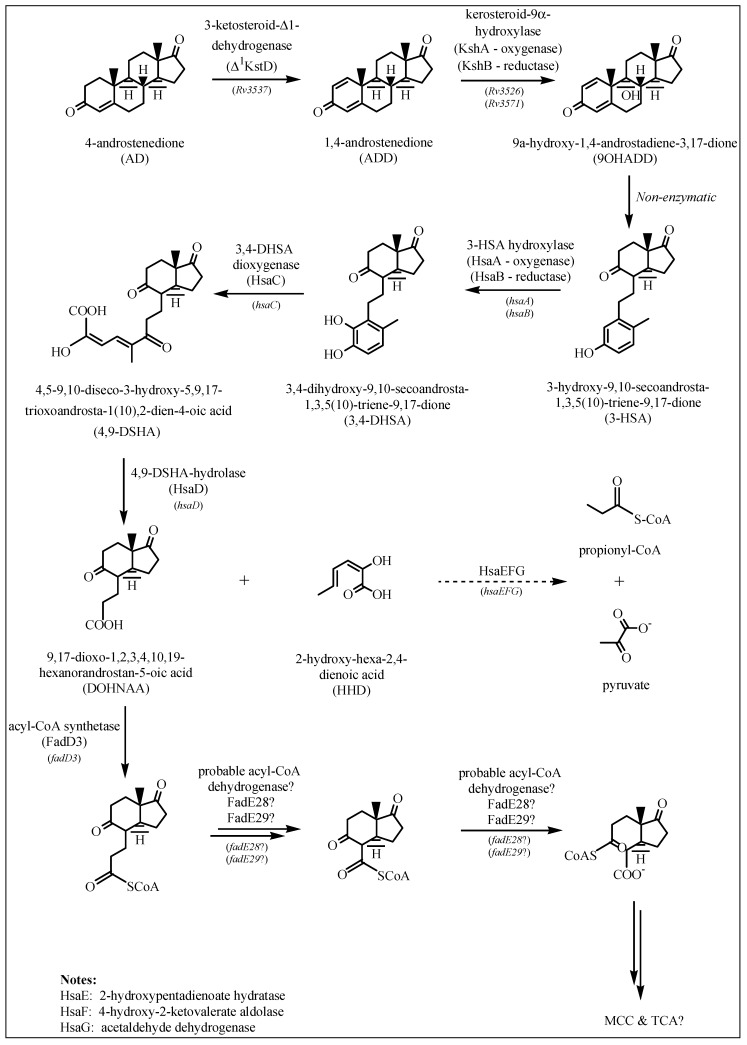
Cholesterol ring degradation as described in Section 2.1.2. If known, the enzymes involved in each reaction are depicted by arrows, along with the gene coding for the specific enzyme.

**Figure 3:**
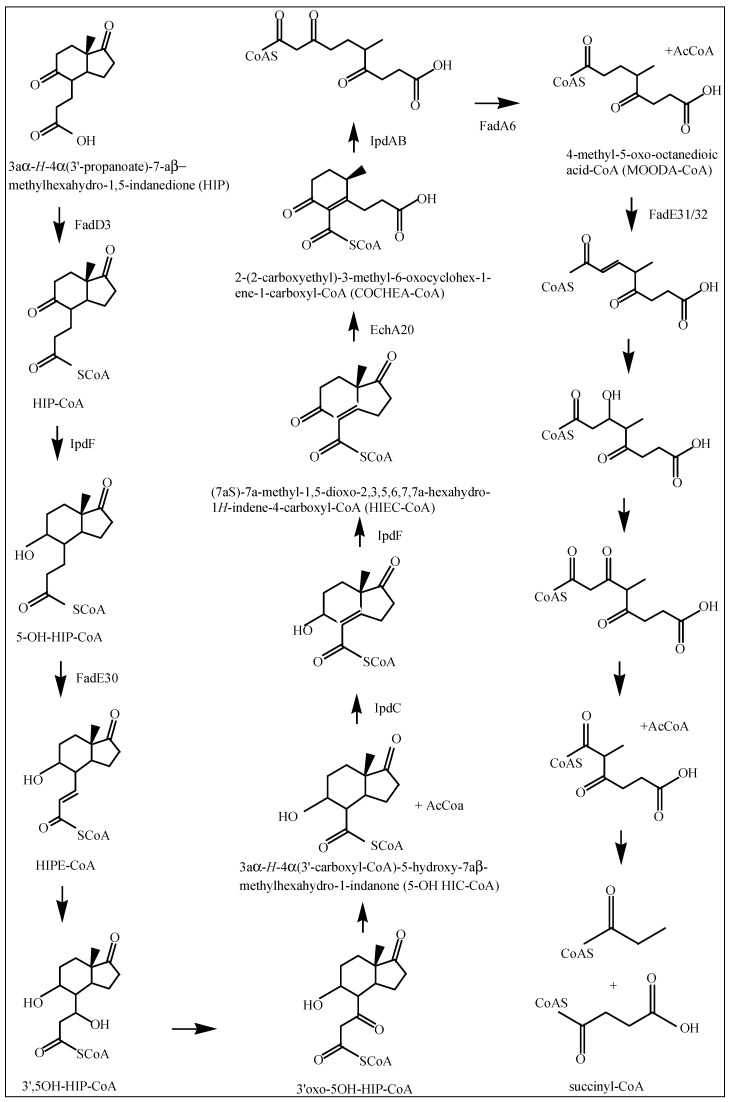
Proposed catabolic pathway of HIP [27]. If known, the enzymes involved in each reaction are depicted by arrows.

Based on the available literature [19,22,23,24,25,26,27], the cholesterol catabolic pathway in *M. tuberculosis* can be divided into two major phases—the initial degradation of the aliphatic side chain (Figure 1) and the subsequent degradation of the four alicyclic A–D rings (Figure 2 and Figure 3). It has not been confirmed whether there is a specific order to the degradation reactions regarding the side chain and rings, but for *M. tuberculosis* it has been suggested that the ring-degrading enzymes KsaAB and HsaA-C act optimally after the side chain has been removed, since blockage of the side chain degradation resulted in accumulation of cholest-4-en-3-one as a major metabolite [19].

#### 2.1.1. Degradation of Cholesterol: Side Chain Degradation

It is generally accepted that the cholesterol side chain is shortened by β-oxidation reactions [19]. Before the saturated side chain of cholesterol can enter into the *M. tuberculosis* β-oxidation pathway, it must first be chemically functionalized at the ω-position [19] (Figure 1). Of the four chemical steps necessary to prepare the side chain for β-oxidation, the first three are oxidation reactions catalyzed by cytochrome P450 enzymes CYP125 (*Rv3545c*), CYP142 (*Rv3518c*) and CYP124 (*Rv2266*) [19,28]. These are capable of oxidizing the side chains of cholesterol and cholest-4-en-3-one to the terminal alcohol (by introducing a hydroxyl group onto the side chain), aldehyde and carboxylic acid metabolites. A sterol-CoA ligase catalyzes the final ATP-dependent step [19] (Figure 1).

Research has demonstrated that CYP125 does not play a key role in cholesterol catabolism in the *M. tuberculosis* H37Rv strain and suggests that this strain carries out compensatory activities [29]. However, investigation of the *in vitro* enzyme specificities found that CYP125 and CYP142 are the dominant P450 enzymes responsible for initiating sterol side chain degradation in *M. tuberculosis* [29], although in the CDC1551 strain, CYP142 is present as a pseudogene [30]. *In vitro* analysis has also demonstrated that CYP142 can support the growth of the H37Rv strain on cholesterol in the absence of *cyp125A1* [29]. Using western blot analysis, researchers found that CYP124A1 was not detectably expressed in the H37Rv or CDC1551 strains, but CYP142 was found in H37Rv and not in CDC1551 [29]. In the absence of CYP125 or CYP142, cholest-4-en-3-one accumulates and inhibits bacterial growth on cholesterol [19].

β-oxidation is the pathway of the breakdown of fatty acids in the form of acyl-CoA molecules, [24]. Before the oxidative reactions of the β-oxidation cycle, the fatty acid is activated in a reaction catalyzed by an ATP-dependent ligase, to its thioester with coenzyme A (CoA). The thioester then undergoes dehydrogenation catalyzed by acyl-CoA dehydrogenase to form the enoyl-CoA, which is then hydrated to the hydroxyacyl-CoA by enoyl-CoA hydratase. Next, 3-hydroxyacyl-CoA dehydrogenase catalyzes the oxidation of the hydroxyl group. The thiolase in the next step, carryout the thiolytic cleavage of β-ketoacyl-CoA into two molecules of acyl-CoA as products, seems to correspond to the FadA5. A single round of the β-oxidation cycle of unbranched chain fatty acids produces acetyl-CoA and a CoA thioester of an acid that is shorter by two carbon atoms. The shortened fatty acyl-CoA then undergoes a further round of the β-oxidation cycle [24].

Genes believed to be encoding β-oxidation enzymes have been identified in the cholesterol regulons of *M. tuberculosis* [19]. One of these enzymes, a thiolase encoded by *fadA5*, catalyzes the thiolysis of acetoacetyl-CoA *in vitro*, which is consistent with removal of the side chain by β-oxidation, producing androstene metabolites, 4-androstenedione (AD) and 1,4-androstenedione (ADD). This activity is required for growth on cholesterol and virulence, especially during the late (chronic) stage of mouse infection, prior to the onset of the immune response [22,30]. Another set of enzymes, acyl-CoA dehydrogenases, is required to catalyze unsaturation reactions in β-oxidation of steroid-CoA substrates, and the *M. tuberculosis* genome contains six sets of these enzyme genes (*fadE*’s). Regulated by cholesterol, each set of these genes is found adjacent to another within the same operon [31].

The research of Schappinger et al. [32] indicates the induction of 18 genes predicted to encode all the enzymes necessary for the biochemical activation and β-oxidation of fatty acids, including fatty acid-CoA synthase (*fadD3*, *fadD9*, *fadD10*, *fadD19*), acyl-CoA dehydrogenase (*fadE5*, *fadE14*, *fadE22-24*, *fadE27-29*, *fadE31*), enoyl-CoA hydratase (*echA19*), hydroxybutyryl-CoA dehydrogenase (*fadB2*, *fadB3*) and acetyl-CoA transferase (*fadA5*, *fadA6*).

Griffin et al. [26] also found that *hsd4A*, another predicted β-oxidation gene, was required for growth on cholesterol, along with *ltp2*, *fadE29*, *fadE28*, *fadA5*, *fadE30*, *fadE32*, *fadE33*, *fadE34*, *hsd4B* and also *fadE5*, *echA9*, *fadD36* and *fadE25*.

#### 2.1.2. Degradation of Cholesterol: Sterol Ring Degradation

The first step in the breakdown of the sterol ring is the conversion of cholesterol to cholest-4-en-3-one (Figure 1). This reaction is catalyzed by either a 3β-HSD or a cholesterol oxidase (ChoD). As mentioned earlier, *Rv1106c* encodes a 3β-HSD. This enzyme uses NAD+ as a cofactor and oxidizes cholesterol (among others) to its 3-keto-4-ene product, cholest-4-en-3-one [19]. *Rv3409c* encodes ChoD and is required for *M. tuberculosis* virulence [33]. However, in a study by Yang et al. [34] it was found that *Rv3409c* was not required for growth on cholesterol as a sole carbon source, and they concluded that 3β-HSD is required for the initial conversion of cholesterol and that a second ChoD activity is not present in *M. tuberculosis*. In addition to this, mice infection experiments confirmed the significance of ChoD in the pathogenesis of *M. tuberculosis*, where it drives the oxidation of 3β-hydroxy-5-ene to 3-keto-4-ene [33].

It is assumed that 3-ketosteroid-Δ^1^-dehydrogenase (Δ^1^KstD) is coded by the *Rv3537* gene that is part of the cholesterol regulon [19,25]. This enzyme catalyzes the trans-axial elimination of the C1(α) and C2(β) hydrogen atoms (C1-C2 dehydrogenation) of the 3-ketosteroid A ring of 4-androstenedione (AD) to yield 1,4-androstenedione (ADD) (Figure 2) [19], and targeted disruption of this gene inhibited growth on cholesterol [35]. In research done by Brzostek et al. [35], direct evidence was found that *M. tuberculosis* degrades cholesterol exclusively *via* the AD/ADD intermediates, and that KstD plays an essential role in this process.

In the next step, 9-hydroxylation of the 3-ketosteroid is catalyzed by KshAB (3-ketosteroid 9α-hydroxylase), a two-component Rieske oxygenase, where KshA (*Rv3526*) is the oxygenase component and KshB (*Rv3571*) is the reductase component [36] (Figure 2). Research has shown that Δ*kshA* and Δ*kshB* deletion mutants are unable to utilize cholesterol and are essential in *M. tuberculosis* pathogenicity [37].

These two steps—the 9-hydroxylation of the 3-ketosteroid together with the C1-C2 dehydrogenation—are key to opening of the B ring and aromatization of the A ring *via* 9-hydroxy-1,4-androstene-3,17-dione (9OHADD) [19]. This intermediate is unstable and spontaneously hydrolyses to 3-hydroxy-9,10-secoandrosta-1,3,5(10)-triene-9,17-dione (3-HSA) [36].

The *hsaACDB* genes in *M. tuberculosis* are part of a single operon and transposon mutagenesis studies have indicated that their activity is critical for the survival of *M. tuberculosis* in macrophages [38,39]. The *hsaA* and *hsaB* genes encode for the putative oxygenase and reductase, respectively, of a flavin-dependent mono-oxygenase that hydroxylates (C4-hydroxylation) 3-HAS, a phenol, to a catechol, 3,4-dihydroxy-9,10-secoandrosta-1,3,5(10)-triene-9,17-dione (3,4-DHSA) [39]. Next, 3,4-DHSA is oxygenated and cleaved by HsaC, an iron-dependent extradiol dioxygenase, to produce 4,5-9,10-diseco-3-hydroxy-5,9,17-trioxoandrosta-1(10),2-dien-4-oic acid (4,9-DSHA) [19]. The inactivation of HsaC results in the death of *M. tuberculosis* due to the accumulation of catechol metabolites [19]. HsaD, a member of the *α/β* hydrolase family, is involved in the aerobic degradation of aromatic compounds in microbes and is coded by *hsaD*, one of the genes identified as required for survival in macrophages [19]. HsaD is hypothesized to catalyze the hydrolysis of a carbon-carbon bond in 4,9-DSHA to yield 9,17-dioxo-1,2,3,4,10,19-hexanorandrostan-5-oic acid (DOHNAA) and 2-hydroxy-hexa-2,4-dienoic acid (HHD). HHD is then metabolized to tricarboxylic acid cycle intermediates [40] and propionyl-CoA [19], probably by HsaEFG (*hsaEFG*) [26]. The metabolic fate of DOHNAA (corresponding to the C and D ring fragments), meanwhile, has recently been elucidated by Crowe et al. [27], who proposed a pathway for the metabolic fate of the C and D rings of steroids (Figure 3). The proposal was that the last two steroid rings of DOHNAA (referred as HIP) are hydrolytically opened by enzymes encoded by the KstR2 regulon, where cleavage of ring D precedes that of ring C (Figure 3). The process is initiated by the degradation of the propionyl side chain by β-oxidation to yield 5-OH HIP-CoA, which is then converted to HIEC-CoA ((7aS)-7a-methyl-1,5-dioxo-2,3,5,6,7,7a-hexahydro-1*H*-indene-4-carboxyl-CoA) by IpdF and IpdC. The two consecutive ring cleavage reactions occur, where EchA20 catalyzes the hydrolysis of ring D, followed by the hydrolysis of ring C catalyzed by IpdAB. The metabolite resulting from the opened ring C is then potentially thiolyzed by FadA6, or another thiolase, to produce MOODA-CoA. An acyl-CoA dehydrogenase, consisting wholly or partly of FadE32, then oxidizes this product to ^2^Δ-MOODA-CoA (4-methyl-5-oxo-octanedioicacid). It is proposed that a final round of β-oxidation yields 2-methyl-β-ketoadipyl-CoA (MβKA-CoA), which can then be cleaved to produce propionyl-CoA and succinyl-CoA (Figure 3). Griffin et al. [26] identified genes *fadE28*, *fadE29* and *fadD3* to be probably involved in the degradation of DOHNAA.

### 2.2. Genes/Proteins Involved in Cholesterol Catabolism in M. Tuberculosis H37Rv

Based on literature, 152 genes/proteins were found to be involved in cholesterol breakdown in *M. tuberculosis* H37Rv (Table 1). These genes/proteins can be classified into four different categories.

#### 2.2.1. Genes Predicted to be Specifically Required for Growth on Cholesterol

Griffin et al. [26] identified 96 genes that are important for the growth of *M. tuberculosis* on cholesterol through a deep sequencing-based mapping approach (Table 1). Independent studies confirm the genes identified to be important for *M. tuberculosis* growth on cholesterol [19,22,25,29,30,41]. A standalone set of genes/proteins predicted to be specifically required for growth on cholesterol is presented in Table S1.

#### 2.2.2. Cholesterol Catabolic Genes Proven to be or Predicted to be Essential for Survival of M. Tuberculosis in Macrophage Cells and in Murine Infection

In the article by Ouellet et al. [19], some of the cholesterol catabolic genes of *M. tuberculosis* were specified as genes proven to be essential for survival in macrophage cells and in murine infection (Table 1), or genes predicted to be essential for survival in macrophage cells and in murine infection (Table 1). Of the 24 genes listed in Table 1 that are proven to be essential for survival in macrophage cells and in murine infection, 17 genes were predicted to be specifically required for growth on cholesterol by Griffin et al. [26] and other studies [22,25,26,29,30,42]. A standalone set of genes/proteins proven to be essential for survival of *M. tuberculosis* in macrophage cells and in murine infection are presented in Table S2. Genes predicted to be essential for survival of *M. tuberculosis* in macrophage cells and in murine infection are presented in Table S3.

#### 2.2.3. Genes/Proteins that are Up-Regulated during Growth on Cholesterol

Van Der Geize et al. [25] predicted a total of 28 genes to be involved in cholesterol catabolism in *M. tuberculosis* H37Rv. Fifty-one genes specifically expressed during growth on cholesterol in *Rhodococcus jostii* are also found in an 82-gene cluster in the *M. tuberculosis* and *M. bovis* bacillus Calmette–Guérin (BCG) genomes. To annotate the cholesterol catabolic genes, the researchers compared the sequence similarity of the gene products of *R. jostii* RHA1 and *M. tuberculosis* H37Rv strains and compiled a table with 28 genes annotated for *M. tuberculosis* H37Rv (Table 1). Independent studies confirmed the importance of these genes in cholesterol catabolism by *M. tuberculosis* [19,22,26,30]. Out of the 28 genes, 18 were predicted to be specifically required for growth on cholesterol; 10 of these genes were proven to be essential for survival of *M. tuberculosis* in macrophage cells and in murine infection and 3 were predicted to be essential for survival of *M. tuberculosis* in macrophage cells and in murine infection (Table 1). A standalone set of genes/proteins predicted to be involved in cholesterol catabolism is presented in Table S4.

#### 2.2.4. Genes Involved in Cholesterol Catabolism by M. Tuberculosis H37Rv, but Not Confirmed or Predicted to Be Essential

Based on literature, 40 genes/proteins were found to be involved in cholesterol catabolism by *M. tuberculosis* H37Rv, but were not confirmed or predicted to be essential according to the published data [19,22,25,30,34,41,43] (Table 1). A standalone set of genes/proteins involved in cholesterol catabolism in *M. tuberculosis* H37Rv is presented in Table S5.

### 2.3. Key Cholesterol Catabolic Genes/Proteins are Not Found in a Large Number of Mycobacterial Species

Because of the omission of 1 gene (Rv3512, as mentioned in Section 3.3.4), 151 genes/proteins were selected to assess the different mycobacterial species’ ability for cholesterol catabolism instead of the initial 152 (Table 1). Mycobacterial species’ ability to catabolize cholesterol was predicted based on the presence of two categories of genes/proteins (i.e., cholesterol catabolic genes/proteins proven or predicted to be essential or specifically required for growth of *M. tuberculosis* H37Rv on cholesterol). Comprehensive comparative analysis of different categories of genes/proteins in mycobacterial species is presented in Table 2.

#### 2.3.1. Most of the M. Tuberculosis Complex Species Have the Ability to Catabolize Cholesterol

**Figure 4:**
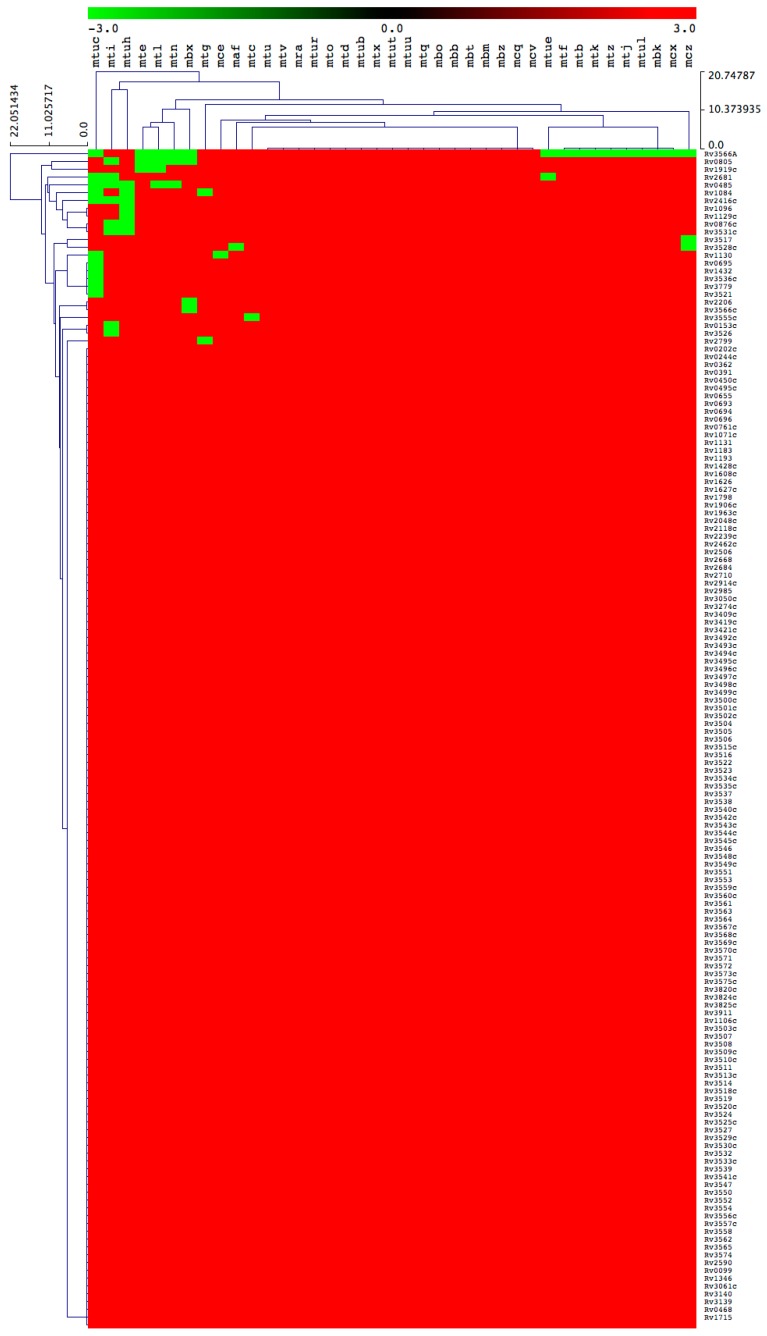
Heatmap of presence or absence of 151 cholesterol catabolic genes/proteins in 39 *M. tuberculosis* complex species. The data have been represented as –3 for gene absence (green) and 3 for gene presence (red). There are 39 mycobacterial species represented on the horizontal axis (see Table 3 for species codes) and 151 genes/proteins on the vertical axis.

Among 39 MTBC species, 29 species were predicted to be positively able to catabolize cholesterol as a carbon source (Figure 4 and Table 2). There were 10 mycobacterial species, namely *M. tuberculosis* RGTB327, *M. tuberculosis* RGTB423, *M. tuberculosis* CCDC5079 (2012), *M. tuberculosis* CCDC5180, *M. tuberculosis* Erdman = ATCC 35801, *M. tuberculosis* CAS/NITR204, *M. tuberculosis* EAI5/NITR206, *M. tuberculosis* Haarlem/NITR202, *M. bovis* BCG ATCC 35743 and *M. canettii* CIPT 140010059, that lacked some of the cholesterol catabolic genes/proteins (Table 2), thus we did not predict their ability to catabolize cholesterol, considering that the complete cholesterol catabolic pathway had not been elucidated.

Analysis of homologous genes/proteins among MTBC species followed the same criteria as described in Section 3.3, with some exceptions for certain homologs mentioned here. For Rv0495c, homolog proteins were identified based on percentage identity, as the NCBI CDD database did not assign proteins to a particular superfamily. The percentage identity was sourced from KEGG and ranged from 99 to 100%. For Rv0805, homolog proteins in *M. tuberculosis* RGTB423 and *M. bovis* BCG ATCC 35743 were not identified, as NCBI CDD did not yield any results. Furthermore, the KEGG database showed only 49% identity compared to other species’ homolog proteins that showed 100% identity. Based on this, we concluded that mti and mbx did not have Rv0805 homolog(s). For Rv1432, there were no hit data for *M. tuberculosis* CAS/NITR204, and KEGG data revealed a different dehydrogenase hit. Thus, it was concluded that the homolog was not present. Upon review of Rv2416c, we found that the homolog protein sequence for *M. tuberculosis* Haarlem/NITR202 was truncated and presented as 28 amino acids compared to the other species’ homologs with more than 360 amino acids. Therefore, it was decided that the homolog of Rv2416c had not been found in *M. tuberculosis* Haarlem/NITR202.

#### 2.3.2. M. Chelonae-Abscessus Complex Species Lack Key Cholesterol Catabolic Genes/Proteins

**Figure 5:**
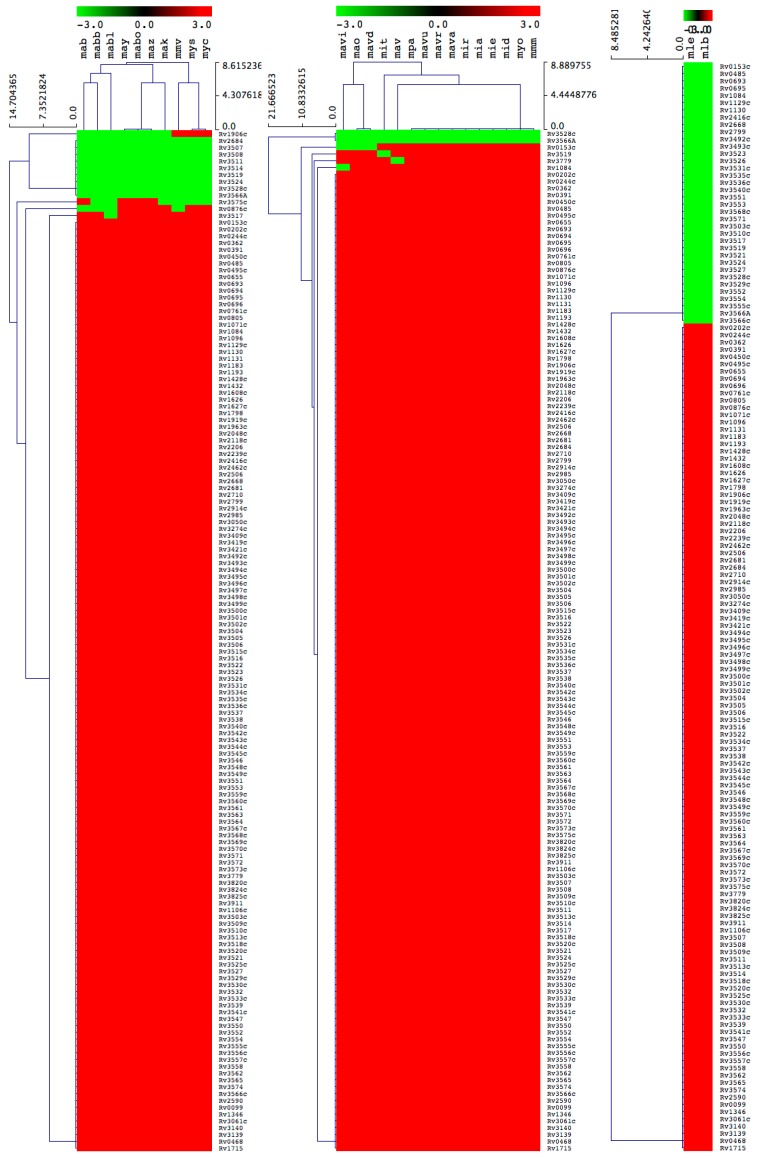
Heatmap of presence or absence of 151 cholesterol catabolic genes/proteins in 10 *M. chelonae-abscessus* complex species (left panel), 15 MAC species (center panel) and 2 *Mycobacterium* species causing leprosy (right panel). The data have been represented as –3 for gene absence (green) and 3 for gene presence (red). The 10, 15 and 2 mycobacterial species are represented on the horizontal axes (see Table 3 for species codes) with the 151 genes/proteins on the vertical axes.

All 10 MCAC species lack the homolog gene of Rv3519 from *M. tuberculosis H37Rv* that has been proven to be essential for survival of *M. tuberculosis* H37Rv in macrophage cells and in murine infection (Figure 5 and Table 2). The function of Rv3519 is not elucidated. In addition to this, all species lack a few genes that are predicted to be essential or specifically required for growth of *M. tuberculosis* H37Rv on cholesterol (Figure 5 and Table 2). Due to the absence of key cholesterol catabolic genes/proteins in MCAC species, and considering the limited information available on cholesterol catabolism in mycobacterial species, at present we do not predict MCAC species’ ability to catabolize cholesterol. Analysis of homologous genes/proteins among MCAC species followed the same criteria as described in Section 3.3, with the exception of Rv1906, as reported earlier in Section 2.3.1, where more than 40% identity to *M. tuberculosis* H37Rv was taken as positive across all the categories, as the proteins were hypothetical.

#### 2.3.3. Most of the M. Avium Complex Species Have the Ability to Catabolize Cholesterol

Among 15 MAC species, 10 were predicted to be positive for their ability to catabolize cholesterol as a carbon source (Figure 5 and Table 2). The remaining five MAC species, *M. avium* subsp. *paratuberculosis* MAP4; *M. avium* subsp. *paratuberculosis* E1; *M. avium* 104; *M. avium* subsp. *avium* DJO-44271 and *M. intracellulare* MOTT-02, did not have the either one or two homologous genes/proteins required for growth on cholesterol (Table 2). Among 151 genes, only 6 *M. tuberculosis* H37Rv homologs, Rv0153c, Rv1084, Rv3779, Rv3519, Rv3528c and Rv3566A, were not found in different MAC species (Figure 5 and Table 2). Four homologs were not found in *M. avium* subsp. *paratuberculosis* E1, and two of these are predicted to be specifically required for growth on cholesterol. Since only a few genes/proteins were missing in the five species, it is difficult to predict their capability to utilize cholesterol as carbon source.

#### 2.3.4. Mycobacterium Causing Leprosy Species Does Not Have the Ability to Catabolize Cholesterol

Two MCL species were predicted to be negative for their ability to catabolize cholesterol as a carbon source (Figure 5 and Table 2). Quite a large number of cholesterol catabolic genes/proteins were not found in both MCL species. Furthermore, experimental evidence proved that MCL species did not have the ability to utilize cholesterol as carbon source [44].

#### 2.3.5. Uncertainty about Non-Tuberculosis Mycobacterium and Saprophyte Species’ Ability to Utilize Cholesterol

**Figure 6:**
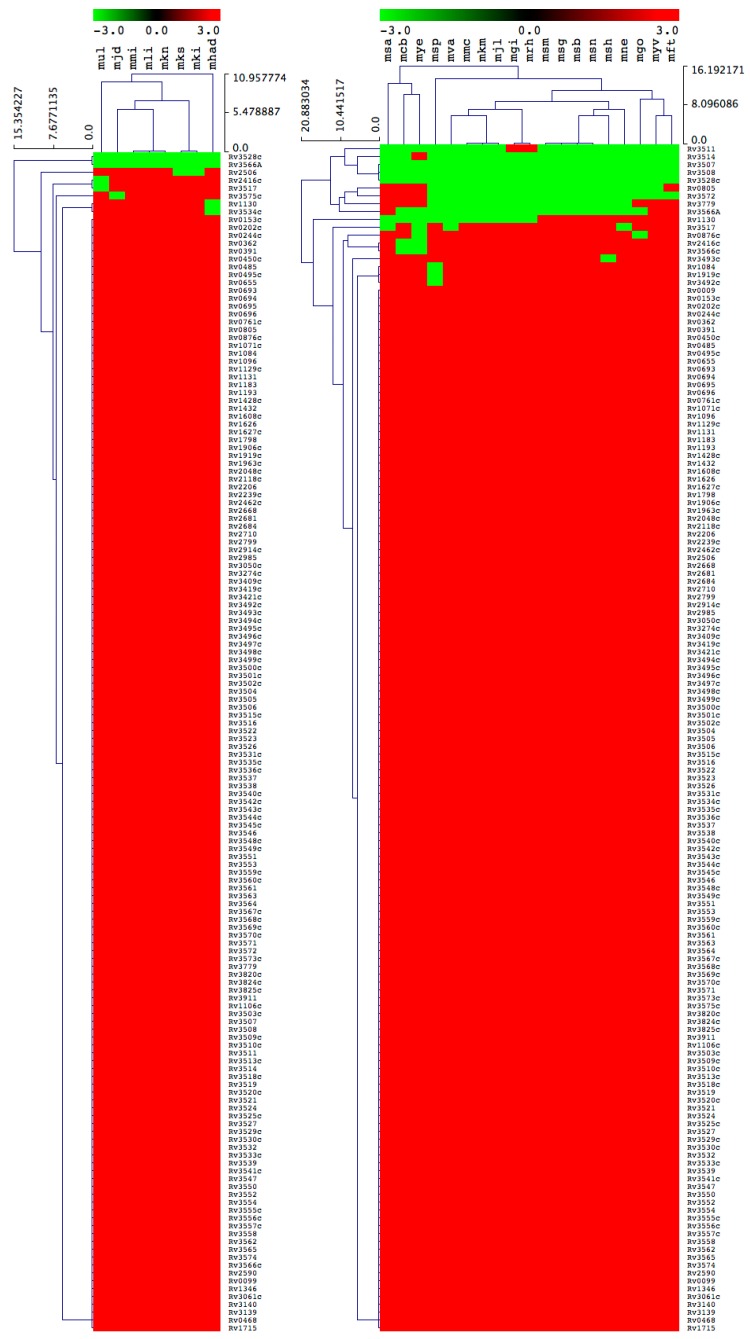
Heatmap of presence or absence of 151 cholesterol catabolic genes/proteins in 8 non-tuberculosis *Mycobacterium* species (left panel) and 19 SAP (right panel). The data have been represented as –3 for gene absence (green) and 3 for gene presence (red). The 8 and 19 mycobacterial species are represented on the horizontal axes (see Table 3 for species codes) with the 151 genes/proteins on the vertical axes.

Among eight NTM species, three species were predicted to be positive for cholesterol utilization as a carbon source (Figure 6 and Table 2). Of the remaining five species, *M. ulcerans*, *M. sinense*, *M. kansasii* 662 and *M. kansasii* 824 had only one missing cholesterol catabolic homolog gene/protein predicted to be essential or specifically required for *M. tuberculosis* H37Rv growth on cholesterol, whereas *M. haemophilum* had three missing cholesterol catabolic homologous genes/proteins proven to be essential (Rv3534c) and predicted to be essential or specifically required for *M. tuberculosis* H37Rv growth (Rv1130 and Rv3534c) on cholesterol (Figure 6 and Table 2). Because of the absence of only a few genes/proteins, it is difficult to predict the five NTM species’ cholesterol utilization ability as a carbon source.

In the SAP species, *Mycobacterium* sp. JS623 (msa) and *M. fortuitum* (mft) lacked a single homologous gene/protein, and the other SAP species had more than one missing cholesterol catabolic homologous gene/protein predicted to be essential or specifically required for *M. tuberculosis* H37Rv growth on cholesterol (Figure 6 and Table 2). However, considering the contrasting lifestyle and habitat of SAP species compared to *M. tuberculosis* H37Rv, the role of cholesterol catabolic genes/proteins proven to be or predicted to be essential for survival of *M. tuberculosis* in macrophage cells and in murine infection [19] that were not found in SAP species may indicate that these genes/proteins do not play any role in cholesterol utilization by SAP species, and possibly all SAPs can utilize cholesterol as a carbon source. The latest study by Guo et al. [45] strongly supports this argument where quite a number of saprophytes, including *M. vanbaalenii*, have been shown to degrade cholesterol. However, experimental evidence will shed more light on SAP species’ ability to metabolize cholesterol. For this reason, we did not predict SAP species’ ability to utilize cholesterol as carbon source.

## 3. Materials and Methods

### 3.1. Species and Database

In total 93 mycobacterial species belonging to 6 different categories were used in this study (Table 3). The 6 categories included *M. tuberculosis* complex (MTBC) (39 species), *M. chelonae-abscessus* complex (MCAC) (10 species), *M. avium* complex (MAC) (15 species), mycobacteria causing leprosy (MCL) (2 species), non-tuberculous mycobacteria (NTM) (8 species) and saprophytes (SAP) (19 species). The criteria for separation of the mycobacterial species into six different groups were based on their characteristic features, including ecological niches, as well as the nature and site of infection as described elsewhere [46,47]. Taxonomical grouping of mycobacterial species was also taken into consideration, as described elsewhere [48]. Detailed information on species, their categories and genome database links are listed in Table 3.

### 3.2. Cholesterol Catabolism

Published research and review articles [19,22,23,24,25,26,27] were consulted to create a schematic diagram of the cholesterol catabolic pathway of *M. tuberculosis* H37Rv, showing the intermediate metabolites and the enzymes involved in different reactions. According to Ouellet et al. [19], the cholesterol catabolic pathway of *M. tuberculosis* can be divided into two major phases—firstly, the initial degradation of the aliphatic side chain, and then the subsequent degradation of the A-D rings. In this study, the two phases were drawn up separately using ChemDraw software [49].

### 3.3. Cholesterol Catabolic Genes/Proteins Analysis in Mycobacterial Species

In total, 152 genes/proteins identified in the study as part of the cholesterol catabolic pathway in *M. tuberculosis* H37Rv. These were selected for comparative analysis from 92 mycobacterial species. The selected 152 protein sequences were retrieved from the Kyoto Encyclopedia of Genes and Genomes (KEGG) database, using their respective gene codes.

#### 3.3.1. BLAST Analysis

The protein sequences of 152 *M. tuberculosis* H37Rv proteins were copied and pasted into the Basic Local Alignment Search Tool (BLAST) in the KEGG database (http://www.genome.jp/tools/blast/). The amino acid sequence was entered in the “sequence data” field, then “favorite organism code or category” was selected under the “KEGG GENES” button, “Mycobacterium” was entered in the free text field provided and the “compute” link was selected at the top. Once the BLAST was complete, the “show all results” link was selected. The resulting output was copied and pasted into an Excel program to extract the required data (organism code, enzyme code, enzyme name, identity and homology (positives)) from all of the BLAST output data, which were then tabulated under each organism name and code (Supplementary Dataset 1).

#### 3.3.2. Excel Program for Extracting KEGG BLAST Data

To extract the required data from the BLAST output data obtained from the KEGG database, an Excel program written in an Excel worksheet was used. The generated program is presented in the Supplementary Materials.

#### 3.3.3. Data Collection and Protein Domain/Function Analysis

All the top hit protein sequences in 92 mycobacterial species were collected (Supplementary Dataset 2) and input into the National Center for Biotechnology Information Batch Web CD-search Tool (NCBI CDD) [50]. Based on the NCBI CDD results, proteins belonging to the same family/superfamily were identified (Supplementary Dataset 3). For some proteins, no results were obtained with the NCBI CDD. Thus, the KEGG database was searched for possible functions or domains to determine whether they belonged to the same group (Supplementary Dataset 1).

#### 3.3.4. Assessing the Presence or Absence of Cholesterol Catabolic Gene/Protein Homologs in Mycobacterial Species

The superfamilies, as per the NCBI CDD output, were considered to determine whether the genes/proteins from the 92 mycobacterial species matched those from *M. tuberculosis* H37Rv. If no data on superfamilies were available in the NCBI database, a secondary review was performed of the KEGG BLAST output data by looking at the percentage identity, percentage homology and name (and thus also the function) of each of the genes/proteins. However, the presence or absence of some proteins in different mycobacterial species was determined based on the information below.

The Rv3512 gene/protein homolog was not identified in many species in the KEGG BLAST output. This may have been due to annotation errors, as *M. tuberculosis* H37Rv (1998) (mtu) and *M. tuberculosis* H37Rv (2012) (mtv) showed different results. Furthermore, this gene is not shown to be essential for cholesterol catabolism. Thus, this gene was omitted from the analysis.

For Rv1906, more than 40% identity to *M. tuberculosis* H37Rv was taken as positive across all categories, as the proteins are hypothetical. According to this, the negative species were *M. abscessus* ATCC 19977, *M. abscessus* subsp. *bolletii* 50594, *M. abscessus* subsp. *bolletii* GO 06, *M. abscessus* subsp. *bolletii* MA 1948, *M. abscessus* subsp. *bolletii* MC1518, *M. abscessus* subsp. *bolletii* CCUG 48898 = JCM 15300, *M. abscessus* subsp. *bolletii* 103, *M. abscessus* subsp. *abscessus* MM1513, *M. abscessus* DJO-44274 and *M. abscessus* 4529.

For Rv3566A, Rv3527 and Rv3572, more than 40% identity to *M. tuberculosis* H37Rv was taken as positive across all categories, as the proteins are hypothetical.

The results were tabulated per complex by colour-coding the cells according to the following criteria: red = gene homolog present; green = gene homolog not found.

### 3.4. Generation of Gene/Protein Heatmaps

The presence or absence of genes/proteins in mycobacterial species was shown with heatmaps following the method described elsewhere [51]. Briefly, the data were represented as −3 for gene absence (green) and 3 for gene presence (red). A tab-delimited file was imported into a Multi-Experiment Viewer (Mev) [52]. A Euclidean distance metric was used to perform hierarchical clustering. Mycobacterial species are presented on the horizontal axis (see Supplementary Dataset 4 for codes) and the 151 genes on the vertical axis.

## 4. Conclusions

The study results were intended to predict mycobacterial species’ ability to utilize cholesterol as a carbon source. To achieve this task, a comprehensive cholesterol catabolic pathway was deduced from the available literature. Genes/proteins involved in the cholesterol catabolism were identified, and comprehensive comparative analysis of *M. tuberculosis* H37Rv homologous genes/proteins in different mycobacterial species was performed, using a newly developed software tool to extract homologous protein data. Gene/protein sequences were collected and subjected to protein family assignment and functional analysis. Finally, based on the presence of genes/proteins critical for cholesterol catabolism, mycobacterial species’ ability to catabolize cholesterol was determined. There are certain points to be taken from the study on predicting the cholesterol utilization capability of mycobacterial species belonging to categories such as MAC, SAP and NTM—i.e., that most of the homolog cholesterol catabolic genes/proteins missing from these species have in fact been proven to be essential for survival of *M. tuberculosis* H37Rv in macrophage cells and in murine infection, but the number of these missing genes/proteins is limited to a single gene in most cases. Thus, it is difficult to predict the cholesterol utilization ability for MAC and NTM species. It is not clear whether these genes/proteins play any role in cholesterol assimilation in SAP species, since these species have different lifestyle and habitat properties compared to *M. tuberculosis* H37Rv. Overall, this study opened new vistas on comparative analysis of cholesterol catabolic genes/proteins in mycobacterial species, and study results should be taken as a source of information on cholesterol catabolic genes/proteins in mycobacterial species.

## Figures and Tables

**Table 1:** List of genes/proteins selected for determining mycobacterial species’ ability to utilize cholesterol. A standalone set of genes representing different categories is presented in Tables S1–S5.

Gene Name	Gene Number	Protein Name
*mce4E/lprN*	*Rv3495c ^a,c,d^*	Mce4 transport system
*mce4C*	*Rv3497c ^a,c,d^*	Mce4 transport system
*mce4A*	*Rv3499c ^a,c,d^*	Mce4 transport system
*yrb4A/YrbE4A/supA*	*Rv3501c ^a,c,d^*	possible ABC transporter (Sterol uptake permease subunit)
*hsd4A*	*Rv3502c ^a,c,d^*	17β-hydroxysteroid dehydrogenase (17β-HSD)
*kshA*	*Rv3526 ^a,c,d^*	kerosteroid-9α-hydroxylase, oxygenase
*hsaF*	*Rv3534c ^a,c,d^*	probable 4-hydroxy-2-oxovalerate aldolase / 4-hydroxy-2-ketovalerate aldolase
*kstD*	*Rv3537 ^b,c,d^*	3-ketosteroid-Δ1-dehydrogenase (Δ1-KSTD)
*fadE28*	*Rv3544c ^a,b,c^*	probable acyl-CoA dehydrogenase
*ipdA*	*Rv3551 ^a,b,c^*	ATP-dependent CoA transferase α subunit
*fadE30*	*Rv3560c ^a,b,c^*	probable acyl-CoA dehydrogenase
*fadE32*	*Rv3563 ^a,b,c^*	probable acyl-CoA dehydrogenase
*hsaC*	*Rv3568c ^a,c,d^*	3,4-DHSA dioxygenase
*hsaD*	*Rv3569c ^b,c,d^*	4,9-DHSA hydrolase
*hsaA*	*Rv3570c ^b,c,d^*	3-hydroxy-9,10-seconandrost-1,3,5(10)-triene-9,17-dione hydroxylase (3-HSA hydroxylase, reductase)
*kshB*	*Rv3571 ^a,c,d^*	ketosteroid-9α-hydroxylase, reductase
*mce4F*	*Rv3494c ^c,d^*	Mce4 transport system
*mce4D*	*Rv3496c ^c,d^*	Mce4 transport system
*mce4B*	*Rv3498c ^c,d^*	Mce4 transport system
*yrb4B/YrbE4B/supB*	*Rv3500c ^c,d^*	possible ABC transporter (Sterol uptake permease subunit)
*fadD19*	*Rv3515c ^c,d^*	probable fatty-acid-CoA ligase
*ltp3*	*Rv3523 ^a,d^*	probable ketoacyl-CoA thiolase
*hsaE*	*Rv3536c ^c,d^*	probable hydratase / 2-hydroxypentadienoate hydratase
*ltp2*	*Rv3540c ^a,c^*	probable ketoacyl-CoA thiolase
	*Rv3542c ^a,c^*	CHP / putative enoyl-CoA hydratase
*cyp125*	*Rv3545c ^a,c^*	cytochrome P450
*fadA5*	*Rv3546 ^a,c^*	acetoacetyl-CoA thiolase
*fadA6*	*Rv3556c ^a,b^*	acetoacetyl-CoA thiolase
*ppiA*	*Rv0009 ^c^*	iron-regulated peptidyl-prolyl cis-trans isomerase A
*fadD10*	*Rv0099 ^e^*	fatty acid-CoA synthase
*ptbB*	*Rv0153c ^c^*	phosphotyrosine protein phosphatase PTPB (protein-tyrosine-phosphatase) (PTPase)
*mmpL11*	*Rv0202c ^c^*	transmembrane transport protein MmpL11
*fadE5*	*Rv0244c ^c^*	acyl-CoA dehydrogenase
*mgtE*	*Rv0362 ^c^*	Mg2+ transport transmembrane protein MgtE
*metZ*	*Rv0391 ^c^*	O-succinylhomoserine sulfhydrylase
*mmpL4*	*Rv0450c ^c^*	transmembrane transport protein MmpL4
*fadB2*	*Rv0468 ^e^*	hydroxybutyryl-CoA dehydrogenase
	*Rv0485 ^c^*	transcriptional regulatory protein
	*Rv0495c ^c^*	HP
*mkl*	*Rv0655 ^c^*	ribonucleotide ABC transporter ATP-binding protein
*pqqE*	*Rv0693 ^c^*	coenzyme PQQ synthesis protein E
*lldD1*	*Rv0694 ^c^*	L-lactate dehydrogenase (cytochrome) LldD1
	*Rv0695 ^c^*	HP
	*Rv0696 ^c^*	membrane sugar transferase
*adhB*	*Rv0761c ^c^*	zinc-containing alcohol dehydrogenase NAD dependent ADHB
	*Rv0805 ^c^*	HP
	*Rv0876c ^c^*	transmembrane protein
*echA9*	*Rv1071c ^c^*	3-hydroxyisobutyryl-CoA hydrolase
	*Rv1084 ^c^*	HP
	*Rv1096 ^c^*	glycosyl hydrolase
	*Rv1106c ^e^*	3β-HSD
	*Rv1129c ^c^*	transcriptional regulator protein
	*Rv1130 ^c^*	HP
*gltA1*	*Rv1131 ^c^*	citrate synthase
*mmpL10*	*Rv1183 ^c^*	transmembrane transport protein MmpL10
*fadD36*	*Rv1193 ^c^*	acyl-CoA synthetase
*mbtN (fadE14)*	*Rv1346 ^e^*	acyl-CoA dehydrogenase
	*Rv1428c ^c^*	HP
	*Rv1432 ^c^*	dehydrogenase
*bcpB*	*Rv1608c ^c^*	peroxidoxin BcpB
	*Rv1626 ^c^*	two-component system transcriptional regulator
	*Rv1627c ^c^*	lipid-transfer protein
*fadB3*	*Rv1715 ^e^*	hydroxybutyryl-CoA dehydrogenase
	*Rv1798 ^c^*	HP
	*Rv1906c ^c^*	HP
	*Rv1919c ^c^*	HP
*mce3R*	*Rv1963c ^c^*	transcriptional repressor (probably TETR-family) MCE3R
*pks12*	*Rv2048c ^c^*	polyketide synthase pks12
	*Rv2118c ^c^*	RNA methyltransferase
	*Rv2206 ^c^*	transmembrane protein
	*Rv2239c ^c^*	HP
*eis*	*Rv2416c ^c^*	HP
*tig*	*Rv2462c ^c^*	trigger factor
	*Rv2506 ^c^*	TetR family transcriptional regulator
*fadD9*	*Rv2590 ^e^*	fatty acid-CoA synthase
	*Rv2668 ^c^*	HP
	*Rv2681 ^c^*	HP
*arsA*	*Rv2684 ^c^*	arsenic-transport integral membrane protein ArsA
*sigB*	*Rv2710 ^c^*	RNA polymerase sigma factor SigB
	*Rv2799 ^c^*	HP
*pknI*	*Rv2914c ^c^*	transmembrane serine/threonine-protein kinase I
*mutT1*	*Rv2985 ^c^*	hydrolase MutT1
	*Rv3050c ^c^*	AsnC family transcriptional regulator
*fadE22*	*Rv3061c ^e^*	acyl-CoA dehydrogenase
*fadE24*	*Rv3139 ^e^*	acyl-CoA dehydrogenase
*fadE23*	*Rv3140 ^e^*	acyl-CoA dehydrogenase
*fadE25*	*Rv3274c ^c^*	acyl-CoA dehydrogenase FADE25
*choD*	*Rv3409c ^d^*	cholesterol oxidase
*gcp*	*Rv3419c ^c^*	putative DNA-binding/iron metalloprotein/AP endonuclease
	*Rv3421c ^c^*	HP
	*Rv3492c ^c^*	CHP MCE associated protein
	*Rv3493c ^c^*	CHP MCE associated protein
*fdxD*	*Rv3503c ^e^*	probable ferredoxin
*fadE26*	*Rv3504 ^d^*	probable acyl-CoA dehydrogenase
*fadE27*	*Rv3505 ^d^*	probable acyl-CoA dehydrogenase
*fadD17*	*Rv3506 ^d^*	possible fatty-acid-CoA ligase
*PE PGRS53*	*Rv3507 ^e^*	PE PGRS family
*PE PGRS54*	*Rv3508 ^e^*	PE PGRS family
*ilvX*	*Rv3509c ^e^*	probable acetohydroxy-acid synthase
	*Rv3510c ^e^*	CHP
*PE PGRS55*	*Rv3511 ^e^*	PE PGRS family
*PE PGRS56*	*Rv3512 ^e^*	PE PGRS family
*fadD18*	*Rv3513c ^e^*	possible fatty-acid-CoA ligase
*PE PGRS57*	*Rv3514 ^e^*	PE PGRS family
*echA19*	*Rv3516 ^d^*	possible enoyl-CoA hydratase
*whiB3*	*Rv3517 ^e^*	conserved hypothetical protein (CHP) / transcription factor
*cyp142*	*Rv3518c ^e^*	cytochrome P450
	*Rv3519 ^a^*	CHP
	*Rv3520c ^e^*	coenzyme F420-dependent oxidoreductase
	*Rv3521 ^e^*	CHP
*ltp4*	*Rv3522 ^d^*	probable ketoacyl-CoA thiolase
	*Rv3524 ^e^*	probable conserved membrane protein
	*Rv3525c ^e^*	possible siderophore binding protein
	*Rv3527 ^a^*	hypothetical protein (HP)
	*Rv3528c ^e^*	HP
	*Rv3529c ^e^*	CHP
	*Rv3530c ^e^*	possible oxidoreductase
	*Rv3531c ^c^*	hypothetical protein
*PPE61*	*Rv3532 ^e^*	PPE family
*PPE62*	*Rv3533c ^e^*	PPE family
*hsaG*	*Rv3535c ^d^*	probable aldehyde dehydrogenase
*hsd4B*	*Rv3538 ^d^*	probable enoyl-CoA hydratase
*PPE63*	*Rv3539 ^e^*	PE
	*Rv3541c ^a^*	CHP / putative enoyl-CoA hydratase
*fadE29*	*Rv3543c ^c^*	probable acyl-CoA dehydrogenase
	*Rv3547 ^e^*	CHP
	*Rv3548c ^c^*	probable short chain dehydrogenase/reductase
	*Rv3549c ^c^*	probable short chain dehydrogenase/reductase
*echA20*	*Rv3550 ^e^*	possible enoyl-CoA hydratase
*ipdB*	*Rv3552 ^a^*	ATP-dependent CoA transferase β subunit
	*Rv3553 ^c^*	possible oxidoreductase / 2-nitropropane dioxygenase
*fdxB*	*Rv3554 ^e^*	possible electron transfer protein / ferredoxin
	*Rv3555c ^e^*	CHP
*kstR2*	*Rv3557c ^e^*	Tet-R transcriptional regulator (repressor)
*PPE64*	*Rv3558 ^e^*	PPE
	*Rv3559c ^c^*	probable oxidoreductase
*fadD3*	*Rv3561 ^c^*	acyl-CoA synthetase (AMP forming)
*fadE31*	*Rv3562 ^e^*	probable acyl-CoA dehydrogenase
*fadE33*	*Rv3564 ^c^*	probable acyl-CoA dehydrogenase
*aspB*	*Rv3565 ^e^*	possible aspartate aminotransferase
	*Rv3566A ^e^*	CHP
*nhoA/nat*	*Rv3566c ^e^*	arylamine N-acetyltransferase
*hsaB*	*Rv3567c ^d^*	3-hydroxy-9,10-seconandrost-1,3,5(10)-triene-9,17-dione hydroxylase (3-HSA hydroxylase, reductase)
	*Rv3572 ^c^*	HP
*fadE34*	*Rv3573c ^c^*	probable acyl-CoA dehydrogenase
*kstR*	*Rv3574 ^a^*	Tet-R transcriptional regulator (repressor)
	*Rv3575c ^c^*	transcriptional regulatory protein LacI-family
	*Rv3779 ^c^*	transmembrane protein alanine and leucine rich
*papA2*	*Rv3820c ^c^*	polyketide synthase associated protein PapA2
*papA1*	*Rv3824c ^c^*	polyketide synthase associated protein
*pks2*	*Rv3825c ^c^*	polyketide synthase PKS2
*sigM*	*Rv3911 ^c^*	RNA polymerase sigma factor SigM

Notes: *^a^* Genes proven to be essential for survival in macrophage cells and in murine infection. *^b^* Genes predicted to be essential for survival in macrophage cells and in murine infection. *^c^* Genes predicted to be specifically required for growth on cholesterol. *^d^* Genes predicted to be involved in cholesterol catabolism compiled from annotation of RHA1, H37Rv and BCG (bacillus Calmette–Guérin) genes assigned to cholesterol pathway. *^e^* Genes involved in cholesterol catabolism by *M. tuberculosis* H37Rv but not confirmed or predicted as essential, according to the published data. Abbreviations: 3-HSA = 3-hydroxy-9,10-secoandrosta-1,3,5(10)-triene-9,17-dione; 3,4-DHSA = 3,4-dihydroxy-9,10-secoandrosta-1,3,5(10)-triene-9,17-dione; 3β-HSD = 3β-hydroxysteroid dehydrogenase; 4,9-DHSA hydrolase = 4,5-9,10-diseco-3-hydroxy-5,9,17-trioxoandrosta-1(10),2-dien-4-oic acid; 17β-HSD = 17β-hydroxysteroid dehydrogenase; Δ1-KSTD = 3-ketosteroid-Δ1-dehydrogenase; ABC = ATP-binding cassette; ADH = alcohol dehydrogenase; AMP = adenosine monophosphate; AP = apurinic/apyrimidinic; ATP = adenosine triphosphate; Bcp = bacterioferritin comigratory protein; CHP = conserved hypothetical protein; CoA = coenzyme A; DNA = deoxyribonucleic acid; HP = hypothetical protein; LldD = L-lactate dehydrogenase; MCE = mammalian cell entry; MgtE = Mg2+ transport transmembrane protein; MmpL = *Mycobacterium* membrane protein laboratory; NAD = nicotinamide adenine dinucleotide; PE = protein family with highly conserved Proline-Glutamate residues near the start of their encoded proteins; PGRS = polymorphic GC-rich-repetitive sequence; pks = polyketide synthase; PPE = protein family with highly conserved proline-proline-glutamate; PQQ = pyrrolo-quinoline quinone; PTP/PTPase = phosphotyrosine protein phosphatase /protein-tyrosine-phosphatase; RNA = ribonucleic acid; TetR/TETR = tetracycline repressor.

**Table 2:** Comparative analysis of cholesterol degrading genes/proteins in mycobacterial species. *M. tuberculosis* H37Rv homologs belonging to different categories not found in mycobacterial species were listed under different categories. The relevant data on BLAST analysis, homolog proteins and protein family analysis are presented in Supplementary Datasets 1–3, respectively. The cholesterol catabolic ability of mycobacterial species was predicted following the presence of genes/proteins that are proven to be essential, and predicted to be essential or specifically required for *M. tuberculosis* H37Rv growth on cholesterol.

Organism Code	H37Rv Homolog(s) Not Found Relating to Cholesterol Catabolism				Ability to Degrade Cholesterol
	Proven to Be Essential	Predicted to Be Essential or Specifically Required	Predicted to Be Involved	Involved but Not Proven or Predicted to Be Essential	
***Mycobacterium tuberculosis* complex (MTBC)**					
mtu	None	None	None	None	Positive
mtv	None	None	None	None	Positive
mtc	None	None	None	Rv3555c	Positive
mra	None	None	None	None	Positive
mtf	None	None	None	Rv3566A	Positive
mtb	None	None	None	Rv3566A	Positive
mtk	None	None	None	Rv3566A	Positive
mtz	None	None	None	Rv3566A	Positive
mtg	None	Rv1084 Rv2799	None	None	No prediction
mti	Rv3526	Rv0153c Rv0485 Rv0805 Rv0876c Rv2416c Rv2681 Rv3526 Rv3531c	Rv3526	None	No prediction
mte	None	Rv0805 Rv1919c	None	Rv3566A	No prediction
mtur	None	None	None	None	Positive
mtl	None	Rv0805 Rv1919c	None	Rv3566A	No prediction
mto	None	None	None	None	Positive
mtd	None	None	None	None	Positive
mtn	None	Rv0805	None	Rv3566A	No prediction
mtj	None	None	None	Rv3566A	Positive
mtub	None	None	None	None	Positive
mtuc	None	Rv0485 Rv0695 Rv1084 Rv1130 Rv1432 Rv2416c Rv2681 Rv3536c Rv3779	Rv3536c	Rv3521 Rv3566A	No prediction
mtue	None	Rv2681	None	Rv3566A	No prediction
mtx	None	None	None	None	Positive
mtuh	None	Rv0485 Rv0876c Rv1084 Rv1096 Rv1129c Rv2416c Rv3531c	None	None	No prediction
mtul	None	None	None	Rv3566A	Positive
mtut	None	None	None	None	Positive
mtuu	None	None	None	None	Positive
mtq	None	None	None	None	Positive
mbo	None	None	None	None	Positive
mbb	None	None	None	None	Positive
mbt	None	None	None	None	Positive
mbm	None	None	None	None	Positive
mbk	None	None	None	Rv3566A	Positive
mbx	None	Rv0805 Rv2206	None	Rv3566A Rv3566c	No prediction
mbz	None	None	None	None	Positive
maf	None	None	None	Rv3528c	Positive
mce	None	Rv1130	None	None	No prediction
mcq	None	None	None	None	Positive
mcv	None	None	None	None	Positive
mcx	None	None		Rv3566A	Positive
mcz	None	None	None	Rv3517 Rv3528c Rv3566A	Positive
***Mycobacterium chelonae-abscessus* complex (MCAC)**					
mab	Rv3519	Rv0876c Rv1906c Rv2684	None	Rv3507 Rv3508 Rv3511 Rv3514 Rv3524 Rv3528c Rv3566A	No prediction
mabb	Rv3519	Rv0876c Rv1906c Rv2684 Rv3575c	None	Rv3507 Rv3508 Rv3511 Rv3514 Rv3524 Rv3528c Rv3566A	No prediction
mmv	Rv3519	Rv0876c Rv2684 Rv3575c	None	Rv3507 Rv3508 Rv3511 Rv3514 Rv3524 Rv3528c Rv3566A	No prediction
may	Rv3519	Rv1906c Rv2684	None	Rv3507 Rv3508 Rv3511 Rv3514 Rv3524 Rv3528c Rv3566A	No prediction
mabo	Rv3519	Rv1906c Rv2684	None	Rv3507 Rv3508 Rv3511 Rv3514 Rv3524 Rv3528c Rv3566A	No prediction
mabl	Rv3519	Rv0876c Rv1906c Rv2684 Rv3575c	None	Rv3507 Rv3508 Rv3511 Rv3514 Rv3517 Rv3524 Rv3528c Rv3566A	No prediction
maz	Rv3519	Rv1906c Rv2684	None	Rv3507 Rv3508 Rv3511 Rv3514 Rv3524 Rv3528c Rv3566A	No prediction
mak	Rv3519	Rv1906c Rv2684 Rv3575c	None	Rv3507 Rv3508 Rv3511 Rv3514 Rv3524 Rv3528c Rv3566A	No prediction
mys	Rv3519	Rv2684 Rv3575c	None	Rv3507 Rv3508 Rv3511 Rv3514 Rv3524 Rv3528c Rv3566A	No prediction
myc	Rv3519	Rv2684 Rv3575c	None	Rv3507 Rv3508 Rv3511 Rv3514 Rv3524 Rv3528c Rv3566A	No prediction
***Mycobacterium avium* complex (MAC)**					
mpa	None	None	None	Rv3528c Rv3566A	Positive
mao	None	Rv0153c	None	Rv3528c Rv3566A	No prediction
mavi	None	Rv0153c Rv1084	None	Rv3528c Rv3566A	No prediction
mavu	None	None	None	Rv3528c Rv3566A	Positive
mav	None	Rv3779	None	Rv3528c Rv3566A	No prediction
mavd	None	Rv0153c	None	Rv3528c Rv3566A	No prediction
mavr	None	None	None	Rv3528c Rv3566A	Positive
mava	None	None	None	Rv3528c Rv3566A	Positive
mit	Rv3519	None	None	Rv3528c Rv3566A	No prediction
mir	None	None	None	Rv3528c Rv3566A	Positive
mia	None	None	None	Rv3528c Rv3566A	Positive
mie	None	None	None	Rv3528c Rv3566A	Positive
mid	None	None	None	Rv3528c Rv3566A	Positive
myo	None	None	None	Rv3528c Rv3566A	Positive
mmm	None	None	None	Rv3528c Rv3566A	Positive
**Mycobacteria causing leprosy (MCL)**					
mle	Rv3523 Rv3526 Rv3540c Rv3551 Rv3568c Rv3571 Rv3519 Rv3527 Rv3552	Rv0153c Rv0485 Rv0693 Rv0695 Rv1084 Rv1129c Rv1130 Rv2416c Rv2668 Rv2799 Rv3492c Rv3493c Rv3526 Rv3531c Rv3536c Rv3540c Rv3551 Rv3553 Rv3568c Rv3571	Rv3523 Rv3526 Rv3535c Rv3536c Rv3568c Rv3571	Rv3503c Rv3510c Rv3517 Rv3521 Rv3524 Rv3528c Rv3529c Rv3554 Rv3555c Rv3566A Rv3566c	Negative
mlb	Rv3523 Rv3526 Rv3540c Rv3551 Rv3568c Rv3571 Rv3519 Rv3527 Rv3552	Rv0153c Rv0485 Rv0693 Rv0695 Rv1084 Rv1129c Rv1130 Rv2416c Rv2668 Rv2799 Rv3492c Rv3493c Rv3526 Rv3531c Rv3536c Rv3540c Rv3551 Rv3553 Rv3568c Rv3571	Rv3523 Rv3526 Rv3535c Rv3536c Rv3568c Rv3571	Rv3503c Rv3510c Rv3517 Rv3521 Rv3524 Rv3528c Rv3529c Rv3554 Rv3555c Rv3566A Rv3566c	Negative
**Non-tuberculosis *Mycobacterium* (NTM)**					
mul	None	Rv2416c	None	Rv3517 Rv3528c Rv3566A	No prediction
mjd	None	Rv3575c	None	Rv3528c Rv3566A	No prediction
mmi	None	None	None	Rv3528c Rv3566A	Positive
mli	None	None	None	Rv3528c Rv3566A	Positive
mkn	None	None	None	Rv3528c Rv3566A	Positive
mks	None	Rv2462c	None	Rv3528c Rv3566A	No prediction
mki	None	Rv2462c	None	Rv3528c Rv3566A	No prediction
mhad	Rv3534c	Rv1130 Rv3534c	Rv3534c	Rv3528c Rv3566A	No prediction
**Saprophytes (SAP)**					
msm	None	Rv0805 Rv3572 Rv3779	None	Rv3507 Rv3508 Rv3511 Rv3514 Rv3528c Rv3566A	No prediction
msg	None	Rv0805 Rv3572 Rv3779	None	Rv3507 Rv3508 Rv3511 Rv3514 Rv3528c Rv3566A	No prediction
msb	None	Rv0805 Rv3572 Rv3779	None	Rv3507 Rv3508 Rv3511 Rv3514 Rv3528c Rv3566A	No prediction
msn	None	Rv0805 Rv3493c Rv3572 Rv3779	None	Rv3507 Rv3508 Rv3511 Rv3514 Rv3528c Rv3566A	No prediction
msh	None	Rv0805 Rv3572 Rv3779	None	Rv3507 Rv3508 Rv3511 Rv3514 Rv3528c Rv3566A	No prediction
msa	None	Rv1130	None	Rv3507 Rv3508 Rv3511 Rv3514 Rv3517 Rv3528c	No prediction
mva	None	Rv0805 Rv1130 Rv3572 Rv3779	None	Rv3507 Rv3508 Rv3511 Rv3514 Rv3517 Rv3528c Rv3566A	No prediction
mgi	None	Rv0805 Rv1130 Rv3572 Rv3779	None	Rv3507 Rv3508 Rv3514 Rv3528c Rv3566A	No prediction
msp	None	Rv0805 Rv1084 Rv1130 Rv1919c Rv3492c Rv3572 Rv3779	None	Rv3507 Rv3508 Rv3511 Rv3514 Rv3528c Rv3566A	No prediction
mmc	None	Rv0805 Rv1130 Rv3572 Rv3779	None	Rv3507 Rv3508 Rv3511 Rv3514 Rv3528c Rv3566A	No prediction
mkm	None	Rv0805 Rv1130 Rv3572 Rv3779	None	Rv3507 Rv3508 Rv3511 Rv3514 Rv3528c Rv3566A	No prediction
mjl	None	Rv0805 Rv1130 Rv3572 Rv3779	None	Rv3507 Rv3508 Rv3511 Rv3514 Rv3528c Rv3566A	No prediction
mrh	None	Rv0805 Rv1130 Rv3572 Rv3779	None	Rv3507 Rv3508 Rv3514 Rv3528c Rv3566A	No prediction
mcb	None	Rv1130 Rv2416c	None	Rv3507 Rv3508 Rv3511 Rv3514 Rv3528c Rv3566A Rv3566c	No prediction
mne	None	Rv0805 Rv3572 Rv3779	None	Rv3507 Rv3508 Rv3511 Rv3514 Rv3517 Rv3528c Rv3566A	No prediction
myv	None	Rv0805 Rv3572	None	Rv3507 Rv3508 Rv3511 Rv3514 Rv3528c	No prediction
mye	None	Rv0876c Rv1130 Rv2416c	None	Rv3507 Rv3508 Rv3511 Rv3517 Rv3528c Rv3566A Rv3566c	No prediction
mgo	None	Rv0805 Rv0876c Rv3572	None	Rv3507 Rv3508 Rv3511 Rv3514 Rv3528c Rv3566A	No prediction
mft	None	Rv3572	None	Rv3507 Rv3508 Rv3511 Rv3514 Rv3528c	No prediction

**Table 3:** List of mycobacterial species and their database links used in the study. For some species, references were not available despite the genome database being available for public use at the Kyoto Encyclopedia of Genes and Genomes (KEGG) database [53] and thus references were not cited for these.

Species Name	Organism Code	Database Link	Reference
***Mycobacterium tuberculosis* complex (MTBC)**			
*Mycobacterium tuberculosis* H37Rv	mtu	http://www.genome.jp/kegg-bin/show_organism?org=mtu	[54]
*Mycobacterium tuberculosis* H37Rv	mtv	http://www.genome.jp/kegg-bin/show_organism?org=mtv	
*Mycobacterium tuberculosis* CDC1551	mtc	http://www.genome.jp/kegg-bin/show_organism?org=mtc	[55]
*Mycobacterium tuberculosis* H37Ra	mra	http://www.genome.jp/kegg-bin/show_organism?org=mra	[56]
*Mycobacterium tuberculosis* F11	mtf	http://www.genome.jp/kegg-bin/show_organism?org=mtf	
*Mycobacterium tuberculosis* KZN 1435	mtb	http://www.genome.jp/kegg-bin/show_organism?org=mtb	
*Mycobacterium tuberculosis* KZN 4207	mtk	http://www.genome.jp/kegg-bin/show_organism?org=mtk	
*Mycobacterium tuberculosis* KZN 605	mtz	http://www.genome.jp/kegg-bin/show_organism?org=mtz	
*Mycobacterium tuberculosis* RGTB327	mtg	http://www.genome.jp/kegg-bin/show_organism?org=mtg	[57]
*Mycobacterium tuberculosis* RGTB423	mti	http://www.genome.jp/kegg-bin/show_organism?org=mti	[57]
*Mycobacterium tuberculosis* CCDC5079	mte	http://www.genome.jp/kegg-bin/show_organism?org=mte	[58]
*Mycobacterium tuberculosis* CCDC5079	mtur	http://www.genome.jp/kegg-bin/show_organism?org=mtur	[59]
*Mycobacterium tuberculosis* CCDC5180	mtl	http://www.genome.jp/kegg-bin/show_organism?org=mtl	[58]
*Mycobacterium tuberculosis* CTRI-2	mto	http://www.genome.jp/kegg-bin/show_organism?org=mto	[60]
*Mycobacterium tuberculosis* UT205	mtd	http://www.genome.jp/kegg-bin/show_organism?org=mtd	[61]
*Mycobacterium tuberculosis* Erdman = ATCC 35801	mtn	http://www.genome.jp/kegg-bin/show_organism?org=mtn	[62]
*Mycobacterium tuberculosis* Beijing/NITR203	mtj	http://www.genome.jp/kegg-bin/show_organism?org=mtj	[63]
*Mycobacterium tuberculosis* 7199-99	mtub	http://www.genome.jp/kegg-bin/show_organism?org=mtub	[64]
*Mycobacterium tuberculosis* CAS/NITR204	mtuc	http://www.genome.jp/kegg-bin/show_organism?org=mtuc	[63]
*Mycobacterium tuberculosis* EAI5/NITR206	mtue	http://www.genome.jp/kegg-bin/show_organism?org=mtue	[63]
*Mycobacterium tuberculosis* EAI5	mtx	http://www.genome.jp/kegg-bin/show_organism?org=mtx	[65]
*Mycobacterium tuberculosis* Haarlem/NITR202	mtuh	http://www.genome.jp/kegg-bin/show_organism?org=mtuh	[63]
*Mycobacterium tuberculosis* Haarlem	mtul	http://www.genome.jp/kegg-bin/show_organism?org=mtul	
*Mycobacterium tuberculosis* BT1	mtut	http://www.genome.jp/kegg-bin/show_organism?org=mtut	
*Mycobacterium tuberculosis* BT2	mtuu	http://www.genome.jp/kegg-bin/show_organism?org=mtuu	
*Mycobacterium tuberculosis* HKBS1	mtq	http://www.genome.jp/kegg-bin/show_organism?org=mtq	
*Mycobacterium bovis* AF2122/97	mbo	http://www.genome.jp/kegg-bin/show_organism?org=mbo	[66]
*Mycobacterium bovis* BCG Pasteur 1173P2	mbb	http://www.genome.jp/kegg-bin/show_organism?org=mbb	[67]
*Mycobacterium bovis* BCG Tokyo 172	mbt	http://www.genome.jp/kegg-bin/show_organism?org=mbt	[68]
*Mycobacterium bovis* BCG Mexico	mbm	http://www.genome.jp/kegg-bin/show_organism?org=mbm	[69]
*Mycobacterium bovis* BCG Korea 1168P	mbk	http://www.genome.jp/kegg-bin/show_organism?org=mbk	[70]
*Mycobacterium bovis* BCG ATCC 35743	mbx	http://www.genome.jp/kegg-bin/show_organism?org=mbx	[71]
*Mycobacterium bovis* ATCC BAA-935	mbz	http://www.genome.jp/kegg-bin/show_organism?org=mbz	
*Mycobacterium africanum*	maf	http://www.genome.jp/kegg-bin/show_organism?org=maf	[72]
*Mycobacterium canettii* CIPT 140010059	mce	http://www.genome.jp/kegg-bin/show_organism?org=mce	[72]
*Mycobacterium canettii* CIPT 140060008	mcq	http://www.genome.jp/kegg-bin/show_organism?org=mcq	[73]
*Mycobacterium canettii* CIPT 140070008	mcv	http://www.genome.jp/kegg-bin/show_organism?org=mcv	[73]
*Mycobacterium canettii* CIPT 140070010	mcx	http://www.genome.jp/kegg-bin/show_organism?org=mcx	[73]
*Mycobacterium canettii* CIPT 140070017	mcz	http://www.genome.jp/kegg-bin/show_organism?org=mcz	[73]
**Mycobacteria causing leprosy (MCL)**			
*Mycobacterium leprae* TN	mle	http://www.genome.jp/kegg-bin/show_organism?org=mle	[74]
*Mycobacterium leprae* Br4923	mlb	http://www.genome.jp/kegg-bin/show_organism?org=mlb	[75]
***Mycobacterium avium* complex (MAC)**			
*Mycobacterium avium* subsp. *paratuberculosis* K-10	mpa	http://www.genome.jp/kegg-bin/show_organism?org=mpa	[76]
*Mycobacterium avium* subsp. *paratuberculosis* MAP4	mao	http://www.genome.jp/kegg-bin/show_organism?org=mao	[77]
*Mycobacterium avium* subsp. *paratuberculosis* E1	mavi	http://www.genome.jp/kegg-bin/show_organism?org=mavi	[78]
*Mycobacterium avium* subsp. *paratuberculosis* E93	mavu	http://www.genome.jp/kegg-bin/show_organism?org=mavu	[78]
*Mycobacterium avium* 104	mav	http://www.genome.jp/kegg-bin/show_organism?org=mav	
*Mycobacterium avium* subsp. *avium* DJO-44271	mavd	http://www.genome.jp/kegg-bin/show_organism?org=mavd	
*Mycobacterium avium* subsp. *avium* 2285 (R)	mavr	http://www.genome.jp/kegg-bin/show_organism?org=mavr	
*Mycobacterium avium* subsp. *avium* 2285 (S)	mava	http://www.genome.jp/kegg-bin/show_organism?org=mava	
*Mycobacterium intracellulare* MOTT-02	mit	http://www.genome.jp/kegg-bin/show_organism?org=mit	[79]
*Mycobacterium intracellulare* MOTT-64	mir	http://www.genome.jp/kegg-bin/show_organism?org=mir	[80]
*Mycobacterium intracellulare* ATCC 13950	mia	http://www.genome.jp/kegg-bin/show_organism?org=mia	[81]
*Mycobacterium intracellulare* 1956	mie	http://www.genome.jp/kegg-bin/show_organism?org=mie	
*Mycobacterium indicus pranii*	mid	http://www.genome.jp/kegg-bin/show_organism?org=mid	[82]
*Mycobacterium yongonense*	myo	http://www.genome.jp/kegg-bin/show_organism?org=myo	[83]
*Mycobacterium* sp. MOTT36Y	mmm	http://www.genome.jp/kegg-bin/show_organism?org=mmm	[84]
**Saprophytes (SAP)**			
*Mycobacterium smegmatis* MC2 155	msm	http://www.genome.jp/kegg-bin/show_organism?org=msm	
*Mycobacterium smegmatis* MC2 155	msg	http://www.genome.jp/kegg-bin/show_organism?org=msg	[85]
*Mycobacterium smegmatis* MC2 155	msb	http://www.genome.jp/kegg-bin/show_organism?org=msb	[86]
*Mycobacterium smegmatis* INHR1	msn	http://www.genome.jp/kegg-bin/show_organism?org=msn	[87]
*Mycobacterium smegmatis* INHR2	msh	http://www.genome.jp/kegg-bin/show_organism?org=msh	[86]
*Mycobacterium* sp. JS623	msa	http://www.genome.jp/kegg-bin/show_organism?org=msa	
*Mycobacterium vanbaalenii*	mva	http://www.genome.jp/kegg-bin/show_organism?org=mva	
*Mycobacterium gilvum* PYR-GCK	mgi	http://www.genome.jp/kegg-bin/show_organism?org=mgi	
*Mycobacterium gilvum* Spyr1	msp	http://www.genome.jp/kegg-bin/show_organism?org=msp	[87]
*Mycobacterium* sp. MCS	mmc	http://www.genome.jp/kegg-bin/show_organism?org=mmc	
*Mycobacterium* sp. KMS	mkm	http://www.genome.jp/kegg-bin/show_organism?org=mkm	
*Mycobacterium* sp. JLS	mjl	http://www.genome.jp/kegg-bin/show_organism?org=mjl	
*Mycobacterium rhodesiae*	mrh	http://www.genome.jp/kegg-bin/show_organism?org=mrh	
*Mycobacterium chubuense*	mcb	http://www.genome.jp/kegg-bin/show_organism?org=mcb	
*Mycobacterium neoaurum*	mne	http://www.genome.jp/kegg-bin/show_organism?org=mne	[88]
*Mycobacterium* sp. VKM Ac-1817D	myv	http://www.genome.jp/kegg-bin/show_organism?org=myv	[88]
*Mycobacterium* sp. EPa45	mye	http://www.genome.jp/kegg-bin/show_organism?org=mye	[89]
*Mycobacterium goodii*	mgo	http://www.genome.jp/kegg-bin/show_organism?org=mgo	[90]
*Mycobacterium fortuitum*	mft	http://www.genome.jp/kegg-bin/show_organism?org=mft	[91]
**Non-tuberculosis mycobacteria (NTM)**			
*Mycobacterium ulcerans*	mul	http://www.genome.jp/kegg-bin/show_organism?org=mul	[92]
*Mycobacterium sinense*	mjd	http://www.genome.jp/kegg-bin/show_organism?org=mjd	[93]
*Mycobacterium marinum*	mmi	http://www.genome.jp/kegg-bin/show_organism?org=mmi	[94]
*Mycobacterium liflandii*	mli	http://www.genome.jp/kegg-bin/show_organism?org=mli	[95]
*Mycobacterium kansasii* ATCC 12478	mkn	http://www.genome.jp/kegg-bin/show_organism?org=mkn	
*Mycobacterium kansasii* 662	mks	http://www.genome.jp/kegg-bin/show_organism?org=mks	
*Mycobacterium kansasii* 824	mki	http://www.genome.jp/kegg-bin/show_organism?org=mki	
*Mycobacterium haemophilum*	mhad	http://www.genome.jp/kegg-bin/show_organism?org=mhad	[96]
***Mycobacterium chelonae-abscessus* complex (MCAC)**			
*Mycobacterium abscessus* ATCC 19977	mab	http://www.genome.jp/kegg-bin/show_organism?org=mab	[97]
*Mycobacterium abscessus* subsp. *bolletii* 50594	mabb	http://www.genome.jp/kegg-bin/show_organism?org=mabb	[98]
*Mycobacterium abscessus* subsp. *bolletii* GO 06	mmv	http://www.genome.jp/kegg-bin/show_organism?org=mmv	[99]
*Mycobacterium abscessus* subsp. *bolletii* MA 1948	may	http://www.genome.jp/kegg-bin/show_organism?org=may	
*Mycobacterium abscessus* subsp. *bolletii* MC1518	mabo	http://www.genome.jp/kegg-bin/show_organism?org=mabo	
*Mycobacterium abscessus* subsp. *bolletii* CCUG 48898 = JCM 15300	mabl	http://www.genome.jp/kegg-bin/show_organism?org=mabl	[100]
*Mycobacterium abscessus* subsp. *bolletii* 103	maz	http://www.genome.jp/kegg-bin/show_organism?org=maz	
*Mycobacterium abscessus* subsp. *abscessus*	mak	http://www.genome.jp/kegg-bin/show_organism?org=mak	
*Mycobacterium abscessus* DJO-44274	mys	http://www.genome.jp/kegg-bin/show_organism?org=mys	
*Mycobacterium abscessus* 4529	myc	http://www.genome.jp/kegg-bin/show_organism?org=myc	

## Supplementary Materials

Supplementary materials can be found at https://www.mdpi.com/1422-0067/20/5/1032/s1.
