# Supplementary material for: Comprehensive Comparative Analysis of Cholesterol Catabolic Genes/Proteins in Mycobacterial Species
Source: Int J Mol Sci. 2019 Feb 27;20(5):1032. doi: 10.3390/ijms20051032 (PMC6429209; doi:10.3390/ijms20051032)
Supplement: Supplementary file 1 [file ijms-20-01032-s001.zip › Supplementary information/Supplementary Dataset 2.docx]

Rv0009 Homologs

>mtu:Rv0009 ppiA; iron-regulated peptidyl-prolyl cis-trans isomerase PpiA; K03767 peptidyl-prolyl cis-trans isomerase A (cyclophilin A) [EC:5.2.1.8] (A)

MADCDSVTNSPLATATATLHTNRGDIKIALFGNHAPKTVANFVGLAQGTKDYSTQNASGG

PSGPFYDGAVFHRVIQGFMIQGGDPTGTGRGGPGYKFADEFHPELQFDKPYLLAMANAGP

GTNGSQFFITVGKTPHLNRRHTIFGEVIDAESQRVVEAISKTATDGNDRPTDPVVIESIT

IS

>mtv:RVBD_0009 iron-regulated peptidyl-prolyl cis-trans isomerase A PpiA; K03767 peptidyl-prolyl cis-trans isomerase A (cyclophilin A) [EC:5.2.1.8] (A)

MADCDSVTNSPLATATATLHTNRGDIKIALFGNHAPKTVANFVGLAQGTKDYSTQNASGG

PSGPFYDGAVFHRVIQGFMIQGGDPTGTGRGGPGYKFADEFHPELQFDKPYLLAMANAGP

GTNGSQFFITVGKTPHLNRRHTIFGEVIDAESQRVVEAISKTATDGNDRPTDPVVIESIT

IS

>mtc:MT0011 ppi-1; peptidyl-prolyl cis-trans isomerase (EC:5.2.1.8); K03767 peptidyl-prolyl cis-trans isomerase A (cyclophilin A) [EC:5.2.1.8] (A)

MADCDSVTNSPLATATATLHTNRGDIKIALFGNHAPKTVANFVGLAQGTKDYSTQNASGG

PSGPFYDGAVFHRVIQGFMIQGGDPTGTGRGGPGYKFADEFHPELQFDKPYLLAMANAGP

GTNGSQFFITVGKTPHLNRRHTIFGEVIDAESQRVVEAISKTATDGNDRPTDPVVIESIT

IS

>mra:MRA_0009 ppiA; iron-regulated peptidyl-prolyl cis-trans isomerase A; K03767 peptidyl-prolyl cis-trans isomerase A (cyclophilin A) [EC:5.2.1.8] (A)

MADCDSVTNSPLATATATLHTNRGDIKIALFGNHAPKTVANFVGLAQGTKDYSTQNASGG

PSGPFYDGAVFHRVIQGFMIQGGDPTGTGRGGPGYKFADEFHPELQFDKPYLLAMANAGP

GTNGSQFFITVGKTPHLNRRHTIFGEVIDAESQRVVEAISKTATDGNDRPTDPVVIESIT

IS

>mtf:TBFG_10009 iron-regulated peptidyl-prolyl-cis-trans-isomerase A ppiA; K03767 peptidyl-prolyl cis-trans isomerase A (cyclophilin A) [EC:5.2.1.8] (A)

MADCDSVTNSPLATATATLHTNRGDIKIALFGNHAPKTVANFVGLAQGTKDYSTQNASGG

PSGPFYDGAVFHRVIQGFMIQGGDPTGTGRGGPGYKFADEFHPELQFDKPYLLAMANAGP

GTNGSQFFITVGKTPHLNRRHTIFGEVIDAESQRVVEAISKTATDGNDRPTDPVVIESIT

IS

>mtb:TBMG_00009 iron-regulated peptidyl-prolyl-cis-trans-isomerase A ppiA; K03767 peptidyl-prolyl cis-trans isomerase A (cyclophilin A) [EC:5.2.1.8] (A)

MADCDSVTNSPLATATATLHTNRGDIKIALFGNHAPKTVANFVGLAQGTKDYSTQNASGG

PSGPFYDGAVFHRVIQGFMIQGGDPTGTGRGGPGYKFADEFHPELQFDKPYLLAMANAGP

GTNGSQFFITVGKTPHLNRRHTIFGEVIDAESQRVVEAISKTATDGNDRPTDPVVIESIT

IS

>mtk:TBSG_00009 iron-regulated peptidyl-prolyl-cis-trans-isomerase A ppiA; K03767 peptidyl-prolyl cis-trans isomerase A (cyclophilin A) [EC:5.2.1.8] (A)

MADCDSVTNSPLATATATLHTNRGDIKIALFGNHAPKTVANFVGLAQGTKDYSTQNASGG

PSGPFYDGAVFHRVIQGFMIQGGDPTGTGRGGPGYKFADEFHPELQFDKPYLLAMANAGP

GTNGSQFFITVGKTPHLNRRHTIFGEVIDAESQRVVEAISKTATDGNDRPTDPVVIESIT

IS

>mtz:TBXG_000009 iron-regulated peptidyl-prolyl-cis-trans-isomerase A ppiA; K03767 peptidyl-prolyl cis-trans isomerase A (cyclophilin A) [EC:5.2.1.8] (A)

MADCDSVTNSPLATATATLHTNRGDIKIALFGNHAPKTVANFVGLAQGTKDYSTQNASGG

PSGPFYDGAVFHRVIQGFMIQGGDPTGTGRGGPGYKFADEFHPELQFDKPYLLAMANAGP

GTNGSQFFITVGKTPHLNRRHTIFGEVIDAESQRVVEAISKTATDGNDRPTDPVVIESIT

IS

>mtg:MRGA327_00065 iron-regulated peptidyl-prolyl cis-trans isomerase A; K03767 peptidyl-prolyl cis-trans isomerase A (cyclophilin A) [EC:5.2.1.8] (A)

MADCDSVTNSPLATATATLHTNRGDIKIALFGNHAPKTVANFVGLAQGTKDYSTQNASGG

PSGPFYDGAVFHRVIQGFMIQGGDPTGTGRGGPGYKFADEFHPELQFDKPYLLAMANAGP

GTNGSQFFITVGKTPHLNRRHTIFGEVIDAESQRVVEAISKTATDGNDRPTDPVVIESIT

IS

>mti:MRGA423_00070 iron-regulated peptidyl-prolyl cis-trans isomerase A; K03767 peptidyl-prolyl cis-trans isomerase A (cyclophilin A) [EC:5.2.1.8] (A)

MADCDSVTNSPLATATATLHTNRGDIKIALFGNHAPKTVANFVGLAQGTKDYSTQNASGG

PSGPFYDGAVFHRVIQGFMIQGGDPTGTGRGGPGYKFADEFHPELQFDKPYLLAMANAGP

GTNGSQFFITVGKTPHLNRRHTIFGEVIDAESQRVVEAISKTATDGNDRPTDPVVIESIT

IS

>mte:CCDC5079_0010 iron-regulated peptidyl-prolyl-cis-trans-isomerase A ppiA; K03767 peptidyl-prolyl cis-trans isomerase A (cyclophilin A) [EC:5.2.1.8] (A)

MTNSPLATATATLHTNRGDIKIALFGNHAPKTVANFVGLAQGTKDYSTQNASGGPSGPFY

DGAVFHRVIQGFMIQGGDPTGTGRGGPGYKFADEFHPELQFDKPYLLAMANAGPGTNGSQ

FFITVGKTPHLNRRHTIFGEVIDAESQRVVEAISKTATDGNDRPTDPVVIESITIS

>mtur:CFBS_0013 ppiA; iron-regulated peptidyl-prolyl-cis-trans-isomerase A; K03767 peptidyl-prolyl cis-trans isomerase A (cyclophilin A) [EC:5.2.1.8] (A)

MADCDSVTNSPLATATATLHTNRGDIKIALFGNHAPKTVANFVGLAQGTKDYSTQNASGG

PSGPFYDGAVFHRVIQGFMIQGGDPTGTGRGGPGYKFADEFHPELQFDKPYLLAMANAGP

GTNGSQFFITVGKTPHLNRRHTIFGEVIDAESQRVVEAISKTATDGNDRPTDPVVIESIT

IS

>mtl:CCDC5180_0011 iron-regulated peptidyl-prolyl-cis-trans-isomerase A ppiA; K03767 peptidyl-prolyl cis-trans isomerase A (cyclophilin A) [EC:5.2.1.8] (A)

MTNSPLATATATLHTNRGDIKIALFGNHAPKTVANFVGLAQGTKDYSTQNASGGPSGPFY

DGAVFHRVIQGFMIQGGDPTGTGRGGPGYKFADEFHPELQFDKPYLLAMANAGPGTNGSQ

FFITVGKTPHLNRRHTIFGEVIDAESQRVVEAISKTATDGNDRPTDPVVIESITIS

>mto:MTCTRI2_0011 ppiA; iron-regulated peptidyl-prolyl cis-trans isomerase A; K03767 peptidyl-prolyl cis-trans isomerase A (cyclophilin A) [EC:5.2.1.8] (A)

MADCDSVTNSPLATATATLHTNRGDIKIALFGNHAPKTVANFVGLAQGTKDYSTQNASGG

PSGPFYDGAVFHRVIQGFMIQGGDPTGTGRGGPGYKFADEFHPELQFDKPYLLAMANAGP

GTNGSQFFITVGKTPHLNRRHTIFGEVIDAESQRVVEAISKTATDGNDRPTDPVVIESIT

IS

>mtd:UDA_0009 ppiA; ppiA; K03767 peptidyl-prolyl cis-trans isomerase A (cyclophilin A) [EC:5.2.1.8] (A)

MADCDSVTNSPLATATATLHTNRGDIKIALFGNHAPKTVANFVGLAQGTKDYSTQNASGG

PSGPFYDGAVFHRVIQGFMIQGGDPTGTGRGGPGYKFADEFHPELQFDKPYLLAMANAGP

GTNGSQFFITVGKTPHLNRRHTIFGEVIDAESQRVVEAISKTATDGNDRPTDPVVIESIT

IS

>mtn:ERDMAN_0013 ppiA; iron-regulated peptidyl-prolyl cis-transisomerase A (EC:5.2.1.8); K03767 peptidyl-prolyl cis-trans isomerase A (cyclophilin A) [EC:5.2.1.8] (A)

MTNSPLATATATLHTNRGDIKIALFGNHAPKTVANFVGLAQGTKDYSTQNASGGPSGPFY

DGAVFHRVIQGFMIQGGDPTGTGRGGPGYKFADEFHPELQFDKPYLLAMANAGPGTNGSQ

FFITVGKTPHLNRRHTIFGEVIDAESQRVVEAISKTATDGNDRPTDPVVIESITIS

>mtj:J112_00055 peptidyl-prolyl cis-trans isomerase; K03767 peptidyl-prolyl cis-trans isomerase A (cyclophilin A) [EC:5.2.1.8] (A)

MADCDSVTNSPLATATATLHTNRGDIKIALFGNHAPKTVANFVGLAQGTKDYSTQNASGG

PSGPFYDGAVFHRVIQGFMIQGGDPTGTGRGGPGYKFADEFHPELQFDKPYLLAMANAGP

GTNGSQFFITVGKTPHLNRRHTIFGEVIDAESQRVVEAISKTATDGNDRPTDPVVIESIT

IS

>mtub:MT7199_0009 putative IRON-REGULATED PEPTIDYL-PROLYL CIS-TRANS ISOMERASE A PPIA (PPIase A) (ROTAMASE A) (EC:5.2.1.8); K03767 peptidyl-prolyl cis-trans isomerase A (cyclophilin A) [EC:5.2.1.8] (A)

MADCDSVTNSPLATATATLHTNRGDIKIALFGNHAPKTVANFVGLAQGTKDYSTQNASGG

PSGPFYDGAVFHRVIQGFMIQGGDPTGTGRGGPGYKFADEFHPELQFDKPYLLAMANAGP

GTNGSQFFITVGKTPHLNRRHTIFGEVIDAESQRVVEAISKTATDGNDRPTDPVVIESIT

IS

>mtuc:J113_00070 peptidyl-prolyl cis-trans isomerase; K03767 peptidyl-prolyl cis-trans isomerase A (cyclophilin A) [EC:5.2.1.8] (A)

MADCDSVTNSPLATATATLHTNRGDIKIALFGNHAPKTVANFVGLAQGTKDYSTQNASGG

PSGPFYDGAVFHRVIQGFMIQGGDPTGTGRGGPGYKFADEFHPELQFDKPYLLAMANAGP

GTNGSQFFITVGKTPHLNRRHTIFGEVIDAESQRVVEAISKTATDGNDRPTDPVVIESIT

IS

>mtue:J114_00060 peptidyl-prolyl cis-trans isomerase; K03767 peptidyl-prolyl cis-trans isomerase A (cyclophilin A) [EC:5.2.1.8] (A)

MADCDSVTNSPLATATATLHTNRGDIKIALFGNHAPKTVANFVGLAQGTKDYSTQNASGG

PSGPFYDGAVFHRVIQGFMIQGGDPTGTGRGGPGYKFADEFHPELQFDKPYLLAMANAGP

GTNGSQFFITVGKTPHLNRRHTIFGEVIDAESQRVVEAISKTATDGNDRPTDPVVIESIT

IS

>mtx:M943_00075 peptidyl-prolyl cis-trans isomerase; K03767 peptidyl-prolyl cis-trans isomerase A (cyclophilin A) [EC:5.2.1.8] (A)

MADCDSVTNSPLATATATLHTNRGDIKIALFGNHAPKTVANFVGLAQGTKDYSTQNASGG

PSGPFYDGAVFHRVIQGFMIQGGDPTGTGRGGPGYKFADEFHPELQFDKPYLLAMANAGP

GTNGSQFFITVGKTPHLNRRHTIFGEVIDAESQRVVEAISKTATDGNDRPTDPVVIESIT

IS

>mtuh:I917_00060 peptidyl-prolyl cis-trans isomerase; K03767 peptidyl-prolyl cis-trans isomerase A (cyclophilin A) [EC:5.2.1.8] (A)

MADCDSVTNSPLATATATLHTNRGDIKIALFGNHAPKTVANFVGLAQGTKDYSTQNASGG

PSGPFYDGAVFHRVIQGFMIQGGDPTGTGRGGPGYKFADEFHPELQFDKPYLLAMANAGP

GTNGSQFFITVGKTPHLNRRHTIFGEVIDAESQRVVEAISKTATDGNDRPTDPVVIESIT

IS

>mtul:TBHG_00009 iron-regulated peptidyl-prolyl cis-trans isomerase A PpiA; K03767 peptidyl-prolyl cis-trans isomerase A (cyclophilin A) [EC:5.2.1.8] (A)

MADCDSVTNSPLATATATLHTNRGDIKIALFGNHAPKTVANFVGLAQGTKDYSTQNASGG

PSGPFYDGAVFHRVIQGFMIQGGDPTGTGRGGPGYKFADEFHPELQFDKPYLLAMANAGP

GTNGSQFFITVGKTPHLNRRHTIFGEVIDAESQRVVEAISKTATDGNDRPTDPVVIESIT

IS

>mtut:HKBT1_0013 ppiA; iron-regulated peptidyl-prolyl-cis-trans-isomerase A; K03767 peptidyl-prolyl cis-trans isomerase A (cyclophilin A) [EC:5.2.1.8] (A)

MADCDSVTNSPLATATATLHTNRGDIKIALFGNHAPKTVANFVGLAQGTKDYSTQNASGG

PSGPFYDGAVFHRVIQGFMIQGGDPTGTGRGGPGYKFADEFHPELQFDKPYLLAMANAGP

GTNGSQFFITVGKTPHLNRRHTIFGEVIDAESQRVVEAISKTATDGNDRPTDPVVIESIT

IS

>mtuu:HKBT2_0013 ppiA; iron-regulated peptidyl-prolyl-cis-trans-isomerase A; K03767 peptidyl-prolyl cis-trans isomerase A (cyclophilin A) [EC:5.2.1.8] (A)

MADCDSVTNSPLATATATLHTNRGDIKIALFGNHAPKTVANFVGLAQGTKDYSTQNASGG

PSGPFYDGAVFHRVIQGFMIQGGDPTGTGRGGPGYKFADEFHPELQFDKPYLLAMANAGP

GTNGSQFFITVGKTPHLNRRHTIFGEVIDAESQRVVEAISKTATDGNDRPTDPVVIESIT

IS

>mtq:HKBS1_0013 ppiA; iron-regulated peptidyl-prolyl-cis-trans-isomerase A; K03767 peptidyl-prolyl cis-trans isomerase A (cyclophilin A) [EC:5.2.1.8] (A)

MADCDSVTNSPLATATATLHTNRGDIKIALFGNHAPKTVANFVGLAQGTKDYSTQNASGG

PSGPFYDGAVFHRVIQGFMIQGGDPTGTGRGGPGYKFADEFHPELQFDKPYLLAMANAGP

GTNGSQFFITVGKTPHLNRRHTIFGEVIDAESQRVVEAISKTATDGNDRPTDPVVIESIT

IS

>mbo:Mb0009 ppiA; iron-regulated peptidyl-prolyl cis-trans isomerase A (EC:5.2.1.8); K03767 peptidyl-prolyl cis-trans isomerase A (cyclophilin A) [EC:5.2.1.8] (A)

MADCDSVTNSPLATATATLHTNRGDIKIALFGNHAPKTVANFVGLAQGTKDYSTQNASGG

PSGPFYDGAVFHRVIQGFMIQGGDPTGTGRGGPGYKFADEFHPELQFDKPYLLAMANAGP

GTNGSQFFITVGKTPHLNRRHTIFGEVIDAESQRVVEAISKTATDGNDRPTDPVVIESIT

IS

>mbb:BCG_0039 ppiA_2; Probable iron-regulated peptidyl-prolyL cis-trans isomerase a ppiA (EC:5.2.1.8); K03767 peptidyl-prolyl cis-trans isomerase A (cyclophilin A) [EC:5.2.1.8] (A)

MADCDSVTNSPLATATATLHTNRGDIKIALFGNHAPKTVANFVGLAQGTKDYSTQNASGG

PSGPFYDGAVFHRVIQGFMIQGGDPTGTGRGGPGYKFADEFHPELQFDKPYLLAMANAGP

GTNGSQFFITVGKTPHLNRRHTIFGEVIDAESQRVVEAISKTATDGNDRPTDPVVIESIT

IS

>mbt:JTY_0009 ppiA; putative iron-regulated peptidyl-prolyl cis-trans isomerase A (EC:5.2.1.8); K03767 peptidyl-prolyl cis-trans isomerase A (cyclophilin A) [EC:5.2.1.8] (A)

MADCDSVTNSPLATATATLHTNRGDIKIALFGNHAPKTVANFVGLAQGTKDYSTQNASGG

PSGPFYDGAVFHRVIQGFMIQGGDPTGTGRGGPGYKFADEFHPELQFDKPYLLAMANAGP

GTNGSQFFITVGKTPHLNRRHTIFGEVIDAESQRVVEAISKTATDGNDRPTDPVVIESIT

IS

>mbm:BCGMEX_0009 ppiA; Peptidyl-prolyl cis-trans isomerase (EC:5.2.1.8); K03767 peptidyl-prolyl cis-trans isomerase A (cyclophilin A) [EC:5.2.1.8] (A)

MADCDSVTNSPLATATATLHTNRGDIKIALFGNHAPKTVANFVGLAQGTKDYSTQNASGG

PSGPFYDGAVFHRVIQGFMIQGGDPTGTGRGGPGYKFADEFHPELQFDKPYLLAMANAGP

GTNGSQFFITVGKTPHLNRRHTIFGEVIDAESQRVVEAISKTATDGNDRPTDPVVIESIT

IS

>mbk:K60_000110 iron-regulated peptidyl-prolyl-cis-trans-isomerase A ppiA; K03767 peptidyl-prolyl cis-trans isomerase A (cyclophilin A) [EC:5.2.1.8] (A)

MTNSPLATATATLHTNRGDIKIALFGNHAPKTVANFVGLAQGTKDYSTQNASGGPSGPFY

DGAVFHRVIQGFMIQGGDPTGTGRGGPGYKFADEFHPELQFDKPYLLAMANAGPGTNGSQ

FFITVGKTPHLNRRHTIFGEVIDAESQRVVEAISKTATDGNDRPTDPVVIESITIS

>mbx:BCGT_3799 Peptidyl-prolyl cis-trans isomerase (EC:5.2.1.8); K03767 peptidyl-prolyl cis-trans isomerase A (cyclophilin A) [EC:5.2.1.8] (A)

MTNSPLATATATLHTNRGDIKIALFGNHAPKTVANFVGLAQGTKDYSTQNASGGPSGPFY

DGAVFHRVIQGFMIQGGDPTGTGRGGPGYKFADEFHPELQFDKPYLLAMANAGPGTNGSQ

FFITVGKTPHLNRRHTIFGEVIDAESQRVVEAISKTATDGNDRPTDPVVIESITIS

>mbz:LH58_00070 peptidylprolyl isomerase; K03767 peptidyl-prolyl cis-trans isomerase A (cyclophilin A) [EC:5.2.1.8] (A)

MADCDSVTNSPLATATATLHTNRGDIKIALFGNHAPKTVANFVGLAQGTKDYSTQNASGG

PSGPFYDGAVFHRVIQGFMIQGGDPTGTGRGGPGYKFADEFHPELQFDKPYLLAMANAGP

GTNGSQFFITVGKTPHLNRRHTIFGEVIDAESQRVVEAISKTATDGNDRPTDPVVIESIT

IS

>maf:MAF_00090 ppiA; putative iron-regulated peptidyl-prolyl cis-trans isomerase A PpiA (EC:5.2.1.8); K03767 peptidyl-prolyl cis-trans isomerase A (cyclophilin A) [EC:5.2.1.8] (A)

MADCDSVTNSPLATATATLHTNRGDIKIALFGNHAPKTVANFVGLAQGTKDYSTQNASGG

PSGPFYDGAVFHRVIQGFMIQGGDPTGTGRGGPGYKFADEFHPELQFDKPYLLAMANAGP

GTNGSQFFITVGKTPHLNRRHTIFGEVIDAESQRVVEAISKTATDGNDRPTDPVVIESIT

IS

>mce:MCAN_00081 ppiA; putative iron-regulated peptidyl-prolyl cis-trans isomerase A PPIA (PPIase A) (rotamase A); K03767 peptidyl-prolyl cis-trans isomerase A (cyclophilin A) [EC:5.2.1.8] (A)

MADCDSVTNSPLATATATLHTNRGDIKIALFGNHAPKTVANFVGLAQGTKDYSTQNASGG

PSGPFYDGAVFHRVIQGFMIQGGDPTGTGRGGPGYKFADEFHPELQFDKPYLLAMANAGP

GTNGSQFFITVGKTPHLNRRHTIFGEVIDAESQRVVEAISKTATDGNDRPTDPVVIESIT

IS

>mcq:BN44_10015 ppiA; Putative peptidyl-prolyl cis-trans isomerase A (EC:5.2.1.8); K03767 peptidyl-prolyl cis-trans isomerase A (cyclophilin A) [EC:5.2.1.8] (A)

MADCDSVTNSPLATATATLHTNRGDIKIALFGNHAPKTVANFVGLAQGTKDYSTQNASGG

PSGPFYDGAVFHRVIQGFMIQGGDPTGTGRGGPGYKFADEFHPELQFDKPYLLAMANAGP

GTNGSQFFITVGKTPHLNRRHTIFGEVIDAESQRVVEAISKTATDGNDRPTDPVVIESIT

IS

>mcv:BN43_10013 ppiA; Putative peptidyl-prolyl cis-trans isomerase A (EC:5.2.1.8); K03767 peptidyl-prolyl cis-trans isomerase A (cyclophilin A) [EC:5.2.1.8] (A)

MADCDSVTNSPLATATATLHTNRGDIKIALFGNHAPKTVANFVGLAQGTKDYSTQNASGG

PSGPFYDGAVFHRVIQGFMIQGGDPTGTGRGGPGYKFADEFHPELQFDKPYLLAMANAGP

GTNGSQFFITVGKTPHLNRRHTIFGEVIDAESQRVVEAISKTATDGNDRPTDPVVIESIT

IS

>mcx:BN42_10030 ppiA; Putative peptidyl-prolyl cis-trans isomerase A (EC:5.2.1.8); K03767 peptidyl-prolyl cis-trans isomerase A (cyclophilin A) [EC:5.2.1.8] (A)

MADCDSVTNSPLATATATLHTNRGDIKIALFGNHAPKTVANFVGLAQGTKDYSTQNASGG

PSGPFYDGAVFHRVIQGFMIQGGDPTGTGRGGPGYKFADEFHPELQFDKPYLLAMANAGP

GTNGSQFFITVGKTPHLNRRHTIFGEVIEPESQRVVEAISKTATDGNDRPTDPVVIESIT

IS

>mcz:BN45_10012 ppiA; Putative peptidyl-prolyl cis-trans isomerase A (EC:5.2.1.8); K03767 peptidyl-prolyl cis-trans isomerase A (cyclophilin A) [EC:5.2.1.8] (A)

MADCDSVTNSPLATATATLHTNRGDIKIALFGNHAPKTVANFVGLAQGTKDYSTQNASGG

PSGPFYDGAVFHRVIQGFMIQGGDPTGTGRGGPGYKFADEFHPELQFDKPYLLAMANAGP

GTNGSQFFITVGKTPHLNRRHTIFGEVIDAESQRVVEAISKTATDGNDRPTDPVVIESIT

IS

>mle:ML0011 ppiA; peptidyl-prolyl cis-trans isomerase (EC:5.2.1.8); K03767 peptidyl-prolyl cis-trans isomerase A (cyclophilin A) [EC:5.2.1.8] (A)

MAHCDFVTNSLIQNATATLHTNRGDIKVALFGNHVPKTVANFVGLAQGTKEYSTQNASGG

PSGPFYDGAVFHRVIQGFMIQGGDPTGTGRGGPGYKFADEFHPELQFDKPYLLAMANAGP

GTNGSQFFITVGETPHLNRRHTIFGEVTDPDSQKVVDAISTTATDGNDRPTEPVVIDSIT

IS

>mlb:MLBr00011 ppiA; putative peptidyl-prolyl cis-trans isomerase (EC:5.2.1.8); K03767 peptidyl-prolyl cis-trans isomerase A (cyclophilin A) [EC:5.2.1.8] (A)

MAHCDFVTNSLIQNATATLHTNRGDIKVALFGNHVPKTVANFVGLAQGTKEYSTQNASGG

PSGPFYDGAVFHRVIQGFMIQGGDPTGTGRGGPGYKFADEFHPELQFDKPYLLAMANAGP

GTNGSQFFITVGETPHLNRRHTIFGEVTDPDSQKVVDAISTTATDGNDRPTEPVVIDSIT

IS

>mpa:MAP_0011 ppiA; PpiA; K03767 peptidyl-prolyl cis-trans isomerase A (cyclophilin A) [EC:5.2.1.8] (A)

MADSDAVTNSPFQTATATLHTNRGDIKVALFGNHAPKTVANFVGLAQGTKEYSTQNASGG

SSGPFYDGAVFHRVIRGFMIQGGDPTGTGRGGPGYKFADEFHPELQFDRPYLLAMANAGP

GTNGSQFLITVDKTPHLNRRHTIFGEVVDPESQKVVDAISTTSTDGNDRPSEPVVIESIT

IS

>mao:MAP4_3864 iron-regulated peptidyl-prolyl cis-transisomerase PpiA; K03767 peptidyl-prolyl cis-trans isomerase A (cyclophilin A) [EC:5.2.1.8] (A)

MADSDAVTNSPFQTATATLHTNRGDIKVALFGNHAPKTVANFVGLAQGTKEYSTQNASGG

SSGPFYDGAVFHRVIRGFMIQGGDPTGTGRGGPGYKFADEFHPELQFDRPYLLAMANAGP

GTNGSQFLITVDKTPHLNRRHTIFGEVVDPESQKVVDAISTTSTDGNDRPSEPVVIESIT

IS

>mavi:RC58_19180 peptidylprolyl isomerase; K03767 peptidyl-prolyl cis-trans isomerase A (cyclophilin A) [EC:5.2.1.8] (A)

MADSDAVTNSPFQTATATLHTNRGDIKVALFGNHAPKTVANFVGLAQGTKEYSTQNASGG

SSGPFYDGAVFHRVIRGFMIQGGDPTGTGRGGPGYKFADEFHPELQFDRPYLLAMANAGP

GTNGSQFLITVDKTPHLNRRHTIFGEVVDPESQKVVDAISTTSTDGNDRPSEPVVIESIT

IS

>mavu:RE97_19230 peptidylprolyl isomerase; K03767 peptidyl-prolyl cis-trans isomerase A (cyclophilin A) [EC:5.2.1.8] (A)

MADSDAVTNSPFQTATATLHTNRGDIKVALFGNHAPKTVANFVGLAQGTKEYSTQNASGG

SSGPFYDGAVFHRVIRGFMIQGGDPTGTGRGGPGYKFADEFHPELQFDRPYLLAMANAGP

GTNGSQFLITVDKTPHLNRRHTIFGEVVDPESQKVVDAISTTSTDGNDRPSEPVVIESIT

IS

>mav:MAV_0013 peptidyl-prolyl cis-trans isomerase B (EC:5.2.1.8); K03767 peptidyl-prolyl cis-trans isomerase A (cyclophilin A) [EC:5.2.1.8] (A)

MTNSPFQTATATLHTNRGDIKVALFGNHAPKTVANFVGLAQGTKEYSTQNASGGSSGPFY

DGAVFHRVIRGFMIQGGDPTGTGRGGPGYKFADEFHPELQFDRPYLLAMANAGPGTNGSQ

FFITVDKTPHLNRRHTIFGEVVDPESQKVVDAISTTSTDGNDRPSEPVVIESITIS

>mavr:LA63_00065 peptidylprolyl isomerase; K03767 peptidyl-prolyl cis-trans isomerase A (cyclophilin A) [EC:5.2.1.8] (A)

MADSDAVTNSPFQTATATLHTNRGDIKVALFGNHAPKTVANFVGLAQGTKEYSTQNASGG

SSGPFYDGAVFHRVIRGFMIQGGDPTGTGRGGPGYKFADEFHPELQFDRPYLLAMANAGP

GTNGSQFFITVDKTPHLNRRHTIFGEVVDPESQKVVDAISTTSTDGNDRPSEPVVIESIT

IS

>mavd:NF84_00065 peptidylprolyl isomerase; K03767 peptidyl-prolyl cis-trans isomerase A (cyclophilin A) [EC:5.2.1.8] (A)

MADSDAVTNSPFQTATATLHTNRGDIKVALFGNHAPKTVANFVGLAQGTKEYSTQNASGG

SSGPFYDGAVFHRVIRGFMIQGGDPTGTGRGGPGYKFADEFHPELQFDRPYLLAMANAGP

GTNGSQFFITVDKTPHLNRRHTIFGEVVDPESQKVVDAISTTSTDGNDRPSEPVVIESIT

IS

>mava:LA64_00065 peptidylprolyl isomerase; K03767 peptidyl-prolyl cis-trans isomerase A (cyclophilin A) [EC:5.2.1.8] (A)

MADSDAVTNSPFQTATATLHTNRGDIKVALFGNHAPKTVANFVGLAQGTKEYSTQNASGG

SSGPFYDGAVFHRVIRGFMIQGGDPTGTGRGGPGYKFADEFHPELQFDRPYLLAMANAGP

GTNGSQFFITVDKTPHLNRRHTIFGEVVDPESQKVVDAISTTSTDGNDRPSEPVVIESIT

IS

>mit:OCO_00110 ppiA (EC:5.2.1.8); K03767 peptidyl-prolyl cis-trans isomerase A (cyclophilin A) [EC:5.2.1.8] (A)

MTNSPFQTATATLHTNRGDIKVALFGNHAPKTVANFVGLAQGTKEYSTQNASGGSSGPFY

DGAVFHRVIRGFMIQGGDPTGTGRGGPGYKFADEFHPELQFDKPYLLAMANAGPGTNGSQ

FFITVDKTPHLNRRHTIFGEVTDPDSQQVVDAIATTSTDGNDRPSEPVVIESITIS

>mir:OCQ_00110 ppiA (EC:5.2.1.8); K03767 peptidyl-prolyl cis-trans isomerase A (cyclophilin A) [EC:5.2.1.8] (A)

MTNSPFQTATATLHTNRGDIKVALFGNHAPKTVANFVGLAQGTKEYSTQNASGGSSGPFY

DGAVFHRVIRGFMIQGGDPTGTGRGGPGYKFADEFHPELQFDKPYLLAMANAGPGTNGSQ

FFITVDKTPHLNRRHTIFGEVTDPDSQQVVDAIATTSTDGNDRPSEPVVIESITIS

>mia:OCU_00110 ppiA (EC:5.2.1.8); K03767 peptidyl-prolyl cis-trans isomerase A (cyclophilin A) [EC:5.2.1.8] (A)

MTNSPFQTATATLHTNRGDIKVALFGNHAPKTVANFVGLAQGTKEYSTQNASGGSSGPFY

DGAVFHRVIRGFMIQGGDPTGTGRGGPGYKFADEFHPELQFDKPYLLAMANAGPGTNGSQ

FFITVDKTPHLNRRHTIFGEVTDPDSQQVVDAIATTSTDGNDRPSEPVVIESITIS

>mie:LG41_00065 peptidylprolyl isomerase; K03767 peptidyl-prolyl cis-trans isomerase A (cyclophilin A) [EC:5.2.1.8] (A)

MADSHAVTNSPFQTATATLHTNRGDIKVALFGNHAPKTVANFVGLAQGTKEYSTQNASGG

SSGPFYDGAVFHRVIRGFMIQGGDPTGTGRGGPGYKFADEFHPELQFDKPYLLAMANAGP

GTNGSQFFITVDKTPHLNRRHTIFGEVTDTDSQQVVDAIATTSTDGNDRPSEPVVIESIT

IS

>mid:MIP_00014 putative peptidyl-prolyl cis-trans isomerase A; K03767 peptidyl-prolyl cis-trans isomerase A (cyclophilin A) [EC:5.2.1.8] (A)

MTNSPFQTATATLHTNRGDIKVALFGNHAPKTVANFVGLAQGTKEYSTQNASGGSSGPFY

DGAVFHRVIRGFMIQGGDPTGTGRGGPGYKFADEFHPELQFDKPYLLAMANAGPGTNGSQ

FFITVDKTPHLNRRHTIFGEVTDPDSQQVVDAIATTSTDGNDRPSEPVVIESITIS

>myo:OEM_00110 ppiA (EC:5.2.1.8); K03767 peptidyl-prolyl cis-trans isomerase A (cyclophilin A) [EC:5.2.1.8] (A)

MTNSPFQTATATLHTNRGDIKVALFGNHAPKTVANFVGLAQGTKEYSTQNASGGSSGPFY

DGAVFHRVIRGFMIQGGDPTGTGRGGPGYKFADEFHPELQFDKPYLLAMANAGPGTNGSQ

FFITVDKTPHLNRRHTIFGEVTDPDSQQVVDAIATTSTDGNDRPSEPVVIESITIS

>msm:MSMEG_0024 peptidyl-prolyl cis-trans isomerase B (EC:5.2.1.8); K03767 peptidyl-prolyl cis-trans isomerase A (cyclophilin A) [EC:5.2.1.8] (A)

MTSPIQTATATLHTNRGDIKIALFGNHAPKTVNNFVGLAQGTKDYSTENASGGTSGPFYD

GAVFHRVIDGFMIQGGDPTGTGRGGPGYKFEDEFHPELQFDKPYLLAMANAGPGTNGSQF

FITVGKTPHLNRRHTIFGEVVDEESQKVVDAIASTPTDRSDRPTEPVVIESITIA

>msg:MSMEI_0026 ppiA; putative peptidyl-prolyl cis-trans isomerase A (EC:5.2.1.8); K03767 peptidyl-prolyl cis-trans isomerase A (cyclophilin A) [EC:5.2.1.8] (A)

MTSPIQTATATLHTNRGDIKIALFGNHAPKTVNNFVGLAQGTKDYSTENASGGTSGPFYD

GAVFHRVIDGFMIQGGDPTGTGRGGPGYKFEDEFHPELQFDKPYLLAMANAGPGTNGSQF

FITVGKTPHLNRRHTIFGEVVDEESQKVVDAIASTPTDRSDRPTEPVVIESITIA

>msb:LJ00_00120 peptidylprolyl isomerase; K03767 peptidyl-prolyl cis-trans isomerase A (cyclophilin A) [EC:5.2.1.8] (A)

MTSPIQTATATLHTNRGDIKIALFGNHAPKTVNNFVGLAQGTKDYSTENASGGTSGPFYD

GAVFHRVIDGFMIQGGDPTGTGRGGPGYKFEDEFHPELQFDKPYLLAMANAGPGTNGSQF

FITVGKTPHLNRRHTIFGEVVDEESQKVVDAIASTPTDRSDRPTEPVVIESITIA

>msn:LI99_00120 peptidylprolyl isomerase; K03767 peptidyl-prolyl cis-trans isomerase A (cyclophilin A) [EC:5.2.1.8] (A)

MTSPIQTATATLHTNRGDIKIALFGNHAPKTVNNFVGLAQGTKDYSTENASGGTSGPFYD

GAVFHRVIDGFMIQGGDPTGTGRGGPGYKFEDEFHPELQFDKPYLLAMANAGPGTNGSQF

FITVGKTPHLNRRHTIFGEVVDEESQKVVDAIASTPTDRSDRPTEPVVIESITIA

>msh:LI98_00120 peptidylprolyl isomerase; K03767 peptidyl-prolyl cis-trans isomerase A (cyclophilin A) [EC:5.2.1.8] (A)

MTSPIQTATATLHTNRGDIKIALFGNHAPKTVNNFVGLAQGTKDYSTENASGGTSGPFYD

GAVFHRVIDGFMIQGGDPTGTGRGGPGYKFEDEFHPELQFDKPYLLAMANAGPGTNGSQF

FITVGKTPHLNRRHTIFGEVVDEESQKVVDAIASTPTDRSDRPTEPVVIESITIA

>msa:Mycsm_00015 peptidyl-prolyl cis-trans isomerase (rotamase) - cyclophilin family; K03767 peptidyl-prolyl cis-trans isomerase A (cyclophilin A) [EC:5.2.1.8] (A)

MAHWHPVTSPIQTATATLHTNRGDIKIALFGNHAPKTVANFVGLAQGTKEYSTENASGSS

SGPFYDGAVFHRVISGFMIQGGDPTGTGRGGPGYQFADEFHPELVFDKPYLLAMANAGPG

TNGSQFFITVGQTPHLNRKHTIFGEVVDPESQKVVDAIATTATDRSDRPTDPVVIESITV

S

>mul:MUL_0013 ppiA; peptidyl-prolyl cis-trans isomerase A, PpiA; K03767 peptidyl-prolyl cis-trans isomerase A (cyclophilin A) [EC:5.2.1.8] (A)

MADCDSVTNSPIQTATATLHTNRGDIKVALFGNHAPKTVANFVGLAQGTKEYSTQNASGS

TSGPFYDGAVFHRVIRGFMIQGGDPTGTGRGGPGYKFADEFHPELQFDKPYLLAMANAGP

GTNGSQFFITVGPTPHLNRRHTIFGEVSDPDSQGVVEAIATTSTDGSDRPTEPVVIESIT

IS

>mva:Mvan_0017 Peptidylprolyl isomerase (EC:5.2.1.8); K03767 peptidyl-prolyl cis-trans isomerase A (cyclophilin A) [EC:5.2.1.8] (A)

MALRPGVAHWIPVTSPIQTATATLHTNRGDIKIALFGNHAPKTVSNFVGLAQGTKEYSTE

NASGGSSGPFYDGVIFHRVIEGFMIQGGDPTGTGRGDAGYKFADEFHPELQFDKPYLLAM

ANAGPGTNGSQFFITVTETPHLNRRHTIFGEVVDPESRKVVDAIATTATDRSDRPTDPVV

IESITIS

>mgi:Mflv_0817 Peptidylprolyl isomerase (EC:5.2.1.8); K03767 peptidyl-prolyl cis-trans isomerase A (cyclophilin A) [EC:5.2.1.8] (A)

MADLIPVTSPIQTATATLHTNRGDIKIALFGNHAPKTVSNFVGLAQGTKEYSTENASGGS

SGPFYDGVIFHRVIEGFMIQGGDPTGTGRGDAGYKFADEFHPELQFDKPYLLAMANAGPG

TNGSQFFITVTETPHLNRRHTIFGEVVDPESRKVVDAIATTATDRSDRPVEPVVIESITI

S

>msp:Mspyr1_00150 peptidyl-prolyl cis-trans isomerase (rotamase) - cyclophilin family (EC:5.2.1.8); K03767 peptidyl-prolyl cis-trans isomerase A (cyclophilin A) [EC:5.2.1.8] (A)

MADLIPVTSPIQTATATLHTNRGDIKIALFGNHAPKTVSNFVGLAQGTKEYSTENASGGS

SGPFYDGVIFHRVIEGFMIQGGDPTGTGRGDAGYKFADEFHPELQFDKPYLLAMANAGPG

TNGSQFFITVTETPHLNRRHTIFGEVVDPESRKVVDAIATTATDRSDRPVEPVVIESITI

S

>mab:MAB_0025 Peptidyl-prolyl cis-trans isomerase; K03767 peptidyl-prolyl cis-trans isomerase A (cyclophilin A) [EC:5.2.1.8] (A)

MTSPTSPIQTATATLHTNRGDVVIALFGNHAPKTVANFVGLADGSKEYSTKNAKGEAAGP

FYDGAIFHRVIDGFMIQGGDPTGTGRGGPGYQFADEFHPELQFDKPYLLAMANAGPGTNG

SQFFITVGKTPHLNRRHTIFGEVVDPASQQVVDSIASTAVDRGDRPTEDVVINSITIS

>mabb:MASS_0025 peptidyl-prolyl cis-trans isomerase; K03767 peptidyl-prolyl cis-trans isomerase A (cyclophilin A) [EC:5.2.1.8] (A)

MAVAKHPRECLFRADKVVVVTSPTSPIQTQTATLHTNRGDVVIALFGNHAPKTVANFVGL

ADGSKDYSTKNAKGEASGPFYDGAIFHRVIDGFMIQGGDPTGTGRGGPGYQFADEFHPEL

QFDKPYLLAMANAGPGTNGSQFFITVGKTPHLNRRHTIFGEVVDPASQQVVDAIASTAVD

RGDRPTEDVVINSITIS

>mmv:MYCMA_0015 peptidyl-prolyl cis-trans isomerase; K03767 peptidyl-prolyl cis-trans isomerase A (cyclophilin A) [EC:5.2.1.8] (A)

MAVAKHPRECLFRADKVVVVTSPTSPIQTQTATLHTNRGDVVIALFGNHAPKTVANFVGL

ADGSKDYSTKNAKGEASGPFYDGAIFHRVIDGFMIQGGDPTGTGRGGPGYQFADEFHPEL

QFDKPYLLAMANAGPGTNGSQFFITVGKTPHLNRRHTIFGEVVDPASQQVVDAIASTAVD

RGDRPTEDVVINSITIS

>may:LA62_00130 peptidylprolyl isomerase; K03767 peptidyl-prolyl cis-trans isomerase A (cyclophilin A) [EC:5.2.1.8] (A)

MTSPTSPIQTATATLHTNRGDVVIALFGNHAPKTVANFVGLADGSKEYSTKNAKGEAAGP

FYDGAIFHRVIDGFMIQGGDPTGTGRGGPGYQFADEFHPELQFDKPYLLAMANAGPGTNG

SQFFITVGKTPHLNRRHTIFGEVVDPASQQVVDSIASTAVDRGDRPTEDVVINSITIS

>mabo:NF82_00125 peptidylprolyl isomerase; K03767 peptidyl-prolyl cis-trans isomerase A (cyclophilin A) [EC:5.2.1.8] (A)

MTSPTSPIQTATATLHTNRGDVVIALFGNHAPKTVANFVGLADGSKEYSTKNAKGEAAGP

FYDGAIFHRVIDGFMIQGGDPTGTGRGGPGYQFADEFHPELQFDKPYLLAMANAGPGTNG

SQFFITVGKTPHLNRRHTIFGEVVDPASQQVVDSIASTAVDRGDRPTEDVVINSITIS

>mabl:MMASJCM_0026 peptidyl-prolyl cis-trans isomerase; K03767 peptidyl-prolyl cis-trans isomerase A (cyclophilin A) [EC:5.2.1.8] (A)

MAVAKHPRECLFRADKVVVVTSPTSPIQTQTATLHTNRGDVVIALFGNHAPKTVANFVGL

ADGSKDYSTKNAKGEASGPFYDGAIFHRVIDGFMIQGGDPTGTGRGGPGYQFADEFHPEL

QFDKPYLLAMANAGPGTNGSQFFITVGKTPHLNRRHTIFGEVVDPASQQVVDAIASTAVD

RGDRPTEDVVINSITIS

>maz:LA61_00130 peptidylprolyl isomerase; K03767 peptidyl-prolyl cis-trans isomerase A (cyclophilin A) [EC:5.2.1.8] (A)

MTSPTSPIQTATATLHTNRGDVVIALFGNHAPKTVANFVGLADGSKEYSTKNAKGEAAGP

FYDGAIFHRVIDGFMIQGGDPTGTGRGGPGYQFADEFHPELQFDKPYLLAMANAGPGTNG

SQFFITVGKTPHLNRRHTIFGEVVDPASQQVVDSIASTAVDRGDRPTEDVVINSITIS

>mak:LH56_22225 peptidylprolyl isomerase; K03767 peptidyl-prolyl cis-trans isomerase A (cyclophilin A) [EC:5.2.1.8] (A)

MTSPTSPIQTQTATLHTNRGDVVIALFGNHAPKTVANFVGLADGSKDYSTKNAKGEASGP

FYDGAIFHRVIDGFMIQGGDPTGTGRGGPGYQFADEFHPELQFDKPYLLAMANAGPGTNG

SQFFITVGKTPHLNRRHTIFGEVVDPASQQVVDAIASTAVDRGDRPTEDVVINSITIS

>mys:NF92_22910 peptidylprolyl isomerase; K03767 peptidyl-prolyl cis-trans isomerase A (cyclophilin A) [EC:5.2.1.8] (A)

MTSPTSPIQTQTATLHTNRGDVVIALFGNHAPKTVANFVGLADGSKDYSTKNAKGEASGP

FYDGAIFHRVIDGFMIQGGDPTGTGRGGPGYQFADEFHPELQFDKPYLLAMANAGPGTNG

SQFFITVGKTPHLNRRHTIFGEVVDPASQQVVDAIASTAVDRGDRPTEDVVINSITIS

>myc:NF90_22930 peptidylprolyl isomerase; K03767 peptidyl-prolyl cis-trans isomerase A (cyclophilin A) [EC:5.2.1.8] (A)

MTSPTSPIQTQTATLHTNRGDVVIALFGNHAPKTVANFVGLADGSKDYSTKNAKGEASGP

FYDGAIFHRVIDGFMIQGGDPTGTGRGGPGYQFADEFHPELQFDKPYLLAMANAGPGTNG

SQFFITVGKTPHLNRRHTIFGEVVDPASQQVVDAIASTAVDRGDRPTEDVVINSITIS

>mmc:Mmcs_0010 Peptidylprolyl isomerase (EC:5.2.1.8); K03767 peptidyl-prolyl cis-trans isomerase A (cyclophilin A) [EC:5.2.1.8] (A)

MTSPIQTATATLHTNRGDIKIALFGNHAPKTVSNFVGLAQGTKDYKGENASGSTSGPFYD

GAVFHRVIDGFMIQGGDPTGTGRGGPGYQFADEFHPELQFDKPYLLAMANAGPGTNGSQF

FITVGKTPHLNRRHTIFGEVVDPESQKVVDAIATTATDRNDRPTDPVVIESVTIS

>mkm:Mkms_0018 Peptidylprolyl isomerase (EC:5.2.1.8); K03767 peptidyl-prolyl cis-trans isomerase A (cyclophilin A) [EC:5.2.1.8] (A)

MTSPIQTATATLHTNRGDIKIALFGNHAPKTVSNFVGLAQGTKDYKGENASGSTSGPFYD

GAVFHRVIDGFMIQGGDPTGTGRGGPGYQFADEFHPELQFDKPYLLAMANAGPGTNGSQF

FITVGKTPHLNRRHTIFGEVVDPESQKVVDAIATTATDRNDRPTDPVVIESVTIS

>mjl:Mjls_0010 Peptidylprolyl isomerase (EC:5.2.1.8); K03767 peptidyl-prolyl cis-trans isomerase A (cyclophilin A) [EC:5.2.1.8] (A)

MTSPIQTATATLHTNRGDIKIALFGNHAPKTVSNFVGLAQGTKDYKGENASGSTSGPFYD

GAVFHRVIDGFMIQGGDPTGTGRGGPGYQFADEFHPELQFDKPYLLAMANAGPGTNGSQF

FITVGKTPHLNRRHTIFGEVVDPESQKVVDAIATTATDRNDRPTDPVVIESVTIS

>mjd:JDM601_0010 ppiA; iron-regulated peptidyl-prolyl cis-trans isomerase A PpiA; K03767 peptidyl-prolyl cis-trans isomerase A (cyclophilin A) [EC:5.2.1.8] (A)

MTTSPIATATATMHTNRGDIKIALFGNHAPKTVANFVGLAQGTKEYSTTNASGGSSGPFY

DGAVFHRVIGGFMIQGGDPTGTGRGGPGYKFEDEFHPELVFDKPYLLAMANAGPGTNGSQ

FFITVGQTPHLNRKHTIFGEVVDPDSRGVVDAIANTATDRGDRPTEPVVIESVTVS

>mmi:MMAR_0011 ppiA; peptidyl-prolyl cis-trans isomerase A, PpiA; K03767 peptidyl-prolyl cis-trans isomerase A (cyclophilin A) [EC:5.2.1.8] (A)

MADCDSVTNSPIQTATATLHTNRGDIKVALFGNHAPKTVANFVGLAQGTKEYSTQNASGS

TSGPFYDGAVFHRVIRGFMIQGGDPTGTGRGGPGYKFADEFHPELQFDKPYLLAMANAGP

GTNGSQFFITVGPTPHLNRRHTIFGEVSDPDSQRVVETIATTSTDGSDRPTEPVVIESIT

IS

>mrh:MycrhN_1832 peptidyl-prolyl cis-trans isomerase (rotamase) - cyclophilin family; K03767 peptidyl-prolyl cis-trans isomerase A (cyclophilin A) [EC:5.2.1.8] (A)

MTSPIQTATATLHTNRGDIKIALFGNHAPKTVANFVGLSQGTKDYTTTNASGGSSGPFYD

GVIFHRVIDGFMIQGGDPTGTGRGDAGYKFADEFHPELQFDKPYLLAMANAGPGTNGSQF

FITVGKTPHLNRKHTIFGEVVDPESQKVVDAIASTPVDRSDRPTDPVVVESITIA

>mmm:W7S_00055 ppiA; K03767 peptidyl-prolyl cis-trans isomerase A (cyclophilin A) [EC:5.2.1.8] (A)

MADSHAVTNSPFQTATATLHTNRGDIKVALFGNHAPKTVANFVGLAQGTKEYSTQNASGG

SSGPFYDGAVFHRVIRGFMIQGGDPTGTGRGGPGYKFADEFHPELQFDKPYLLAMANAGP

GTNGSQFFITVDKTPHLNRRHTIFGEVTDPDSQQVVDAIATTSTDGNDRPSEPVVIESIT

IS

>mcb:Mycch_0014 peptidyl-prolyl cis-trans isomerase (rotamase) - cyclophilin family; K03767 peptidyl-prolyl cis-trans isomerase A (cyclophilin A) [EC:5.2.1.8] (A)

MTSPLQTATATLHTNRGDIKIALFGNHAPKTVANFVGLAQGTKDYSTENSSGGSSGPFYD

GAVFHRVIDGFMIQGGDPTGTGRGGPGYQFADEFHPELQFDKPYLLAMANAGPGTNGSQF

FITVTKTPHLNRRHTIFGEVVDPESQKVVDAIATTSTDRSDRPTEPVVIESITIS

>mli:MULP_00011 ppiA; peptidyl-prolyl isomerase, PpiA (EC:5.2.1.8); K03767 peptidyl-prolyl cis-trans isomerase A (cyclophilin A) [EC:5.2.1.8] (A)

MADCDSVTNSPIQTATATLRTNRGDIKVALFGNHAPKTVANFVGLAQGTKEYSTQNASGS

TSGPFYDGAVFHRVIRGFMIQGGDPTGTGRGGPGYKFADEFHPELQFDKPYLLAMANAGP

GTNGSQFFITVGPTPHLNRRHTIFGEVSDPDSQGVVEAIATTSTDGSDRPTEPVVIESIT

IS

>mkn:MKAN_14925 peptidyl-prolyl cis-trans isomerase; K03767 peptidyl-prolyl cis-trans isomerase A (cyclophilin A) [EC:5.2.1.8] (A)

MAHCDPVTNSPFQTATATLHTNRGDIKVALFGNHAPKTVANFVGLAQGTKDYSTQNASGG

TSGPFYDGAVFHRVINGFMIQGGDPTGTGRGGPGYKFADEFHPELQFDKPYLLAMANAGP

GTNGSQFFITVGKTPHLNRRHTIFGEVTDPESQRVVDAIATTPTDGNDRPTDPVVIESIT

IS

>mks:LG40_14740 peptidylprolyl isomerase; K03767 peptidyl-prolyl cis-trans isomerase A (cyclophilin A) [EC:5.2.1.8] (A)

MAHCDPVTNSPFQTATATLHTNRGDIKVALFGNHAPKTVANFVGLAQGTKDYSTQNASGG

TSGPFYDGAVFHRVINGFMIQGGDPTGTGRGGPGYKFADEFHPELQFDKPYLLAMANAGP

GTNGSQFFITVGKTPHLNRRHTIFGEVTDPESQRVVDAIATTPTDGNDRPTDPVVIESIT

IS

>mki:LH54_14865 peptidylprolyl isomerase; K03767 peptidyl-prolyl cis-trans isomerase A (cyclophilin A) [EC:5.2.1.8] (A)

MAHCDPVTNSPFQTATATLHTNRGDIKVALFGNHAPKTVANFVGLAQGTKDYSTQNASGG

TSGPFYDGAVFHRVINGFMIQGGDPTGTGRGGPGYKFADEFHPELQFDKPYLLAMANAGP

GTNGSQFFITVGKTPHLNRRHTIFGEVTDPESQRVVDAIATTPTDGNDRPTDPVVIESIT

IS

>mne:D174_02110 peptidylprolyl isomerase; K03767 peptidyl-prolyl cis-trans isomerase A (cyclophilin A) [EC:5.2.1.8] (A)

MTSPIQTATATLHTNRGDIKIALFGNHAPKTVANFTGLAQGTKDYSTENATGGSSGPFYD

GAVFHRVIDGFMIQGGDPTGTGRGGPGYQFADEFHPELQFDKPYLLAMANAGPGTNGSQF

FITVGKTPHLNRRHTIFGEVVDPESQKVVDAIATTATDRSDRPTEPVVIESITIS

>myv:G155_00070 peptidylprolyl isomerase; K03767 peptidyl-prolyl cis-trans isomerase A (cyclophilin A) [EC:5.2.1.8] (A)

MTSPIQTATATLHTNRGDIKIALFGNHAPKTVANFVGLAQGTKDYTTQNASGGTSGPFYD

GVIFHRVIDGFMIQGGDPTGTGRGDAGYKFADEFHPELQFDKPYLLAMANAGPGTNGSQF

FITVGPTPHLNRRHTIFGEVVDPESQKVVDAIASTPTDRSDRPTDPVVIESITVS

>mye:AB431_00100 peptidylprolyl isomerase; K03767 peptidyl-prolyl cis-trans isomerase A (cyclophilin A) [EC:5.2.1.8] (A)

MTSDIATATATLHTNRGDIKIALFGNHAPKTVANFVGLAQGTKEYSTQNASGSTSGPFYD

GAVFHRVIDGFMIQGGDPTGTGRGGPGYKFADEFHGELQFDKPYLLAMANAGPGTNGSQF

FITVGKTPHLNRRHTIFGEVVDPESQKVVDAIATTATDRNDRPTDDVVIESITIS

>mgo:AFA91_07005 peptidylprolyl isomerase; K03767 peptidyl-prolyl cis-trans isomerase A (cyclophilin A) [EC:5.2.1.8] (A)

MTSPIQTATATLHTNRGDIKIALFGNHAPKTVNNFVGLAQGTKDYSTENASGGTSGPFYD

GAIFHRVIDGFMIQGGDPTGTGRGGPGYKFDDEFHPELQFDKPYLLAMANAGPGTNGSQF

FITVGKTPHLNRRHTIFGEVVDPESQKVVDAIASTPTDRGDRPTDPVVIESITIA

>mft:XA26_00160 Peptidyl-prolyl cis-trans isomerase (EC:5.2.1.8); K03767 peptidyl-prolyl cis-trans isomerase A (cyclophilin A) [EC:5.2.1.8] (A)

MTSPIQTATATLHTNRGDIKIALFGNHAPKTVANFVGLAQGTKDYTTQNASGGTSGPFYD

GVIFHRVIDGFMIQGGDPTGTGRGDAGYKFADEFHPELQFDKPYLLAMANAGPGTNGSQF

FITVGPTPHLNRRHTIFGEVVDPESQKVVDAIASTPTDRSDRPTDPVVIESITVS

>mhad:B586_02460 peptidylprolyl isomerase; K03767 peptidyl-prolyl cis-trans isomerase A (cyclophilin A) [EC:5.2.1.8] (A)

MAHCDLVTNSPIQTATATLHTNRGDIKVALFGNHAPKTVANFVGLAQGTKEYSTQNASGG

PSGPFYDGAVFHRVIQGFMIQGGDPTGTGRGGPGYKFADEFHPELSFDKPYLLAMANAGP

GTNGSQFFITVGETPHLNRRHTIFGEVTDSDSQRVVDAISTTATDGNDRPTDPVVIESIT

IS

Rv0099 Homologs

>mtu:Rv0099 K12422 long chain fatty acid CoA ligase FadD10 [EC:6.2.1.-] | (RefSeq) fadD10; fatty-acid--CoA ligase FadD10 (A)

MGGKKFQAMPQLPSTVLDRVFEQARQQPEAIALRRCDGTSALRYRELVAEVGGLAADLRA

QSVSRGSRVLVISDNGPETYLSVLACAKLGAIAVMADGNLPIAAIERFCQITDPAAALVA

PGSKMASSAVPEALHSIPVIAVDIAAVTRESEHSLDAASLAGNADQGSEDPLAMIFTSGT

TGEPKAVLLANRTFFAVPDILQKEGLNWVTWVVGETTYSPLPATHIGGLWWILTCLMHGG

LCVTGGENTTSLLEILTTNAVATTCLVPTLLSKLVSELKSANATVPSLRLVGYGGSRAIA

ADVRFIEATGVRTAQVYGLSETGCTALCLPTDDGSIVKIEAGAVGRPYPGVDVYLAATDG

IGPTAPGAGPSASFGTLWIKSPANMLGYWNNPERTAEVLIDGWVNTGDLLERREDGFFYI

KGRSSEMIICGGVNIAPDEVDRIAEGVSGVREAACYEIPDEEFGALVGLAVVASAELDES

AARALKHTIAARFRRESEPMARPSTIVIVTDIPRTQSGKVMRASLAAAATADKARVVVRG

>mtv:RVBD_0099 K12422 long chain fatty acid CoA ligase FadD10 [EC:6.2.1.-] | (GenBank) fatty-acid-CoA ligase FadD10 (A)

MGGKKFQAMPQLPSTVLDRVFEQARQQPEAIALRRCDGTSALRYRELVAEVGGLAADLRA

QSVSRGSRVLVISDNGPETYLSVLACAKLGAIAVMADGNLPIAAIERFCQITDPAAALVA

PGSKMASSAVPEALHSIPVIAVDIAAVTRESEHSLDAASLAGNADQGSEDPLAMIFTSGT

TGEPKAVLLANRTFFAVPDILQKEGLNWVTWVVGETTYSPLPATHIGGLWWILTCLMHGG

LCVTGGENTTSLLEILTTNAVATTCLVPTLLSKLVSELKSANATVPSLRLVGYGGSRAIA

ADVRFIEATGVRTAQVYGLSETGCTALCLPTDDGSIVKIEAGAVGRPYPGVDVYLAATDG

IGPTAPGAGPSASFGTLWIKSPANMLGYWNNPERTAEVLIDGWVNTGDLLERREDGFFYI

KGRSSEMIICGGVNIAPDEVDRIAEGVSGVREAACYEIPDEEFGALVGLAVVASAELDES

AARALKHTIAARFRRESEPMARPSTIVIVTDIPRTQSGKVMRASLAAAATADKARVVVRG

>mtc:MT0108 K12422 long chain fatty acid CoA ligase FadD10 [EC:6.2.1.-] | (GenBank) substrate--CoA ligase, putative (A)

MGGKKFQAMPQLPSTVLDRVFEQARQQPEAIALRRCDGTSALRYRELVAEVGGLAADLRA

QSVSRGSRVLVISDNGPETYLSVLACAKLGAIAVMADGNLPIAAIERFCQITDPAAALVA

PGSKMASSAVPEALHSIPVIAVDIAAVTRESEHSLDAASLAGNADQGSEDPLAMIFTSGT

TGEPKAVLLANRTFFAVPDILQKEGLNWVTWVVGETTYSPLPATHIGGLWWILTCLMHGG

LCVTGGENTTSLLEILTTNAVATTCLVPTLLSKLVSELKSANATVPSLRLVGYGGSRAIA

ADVRFIEATGVRTAQVYGLSETGCTALCLPTDDGSIVKIEAGAVGRPYPGVDVYLAATDG

IGPTAPGAGPSASFGTLWIKSPANMLGYWNNPERTAEVLIDGWVNTGDLLERREDGFFYI

KGRSSEMIICGGVNIAPDEVDRIAEGVSGVREAACYEIPDEEFGALVGLAVVASAELDES

AARALKHTIAARFRRESEPMARPSTIVIVTDIPRTQSGKVMRASLAAAATADKARVVVRG

>mra:MRA_0103 K12422 long chain fatty acid CoA ligase FadD10 [EC:6.2.1.-] | (GenBank) fadD10; fatty-acid-CoA ligase FadD10 (A)

MGGKKFQAMPQLPSTVLDRVFEQARQQPEAIALRRCDGTSALRYRELVAEVGGLAADLRA

QSVSRGSRVLVISDNGPETYLSVLACAKLGAIAVMADGNLPIAAIERFCQITDPAAALVA

PGSKMASSAVPEALHSIPVIAVDIAAVTRESEHSLDAASLAGNADQGSEDPLAMIFTSGT

TGEPKAVLLANRTFFAVPDILQKEGLNWVTWVVGETTYSPLPATHIGGLWWILTCLMHGG

LCVTGGENTTSLLEILTTNAVATTCLVPTLLSKLVSELKSANATVPSLRLVGYGGSRAIA

ADVRFIEATGVRTAQVYGLSETGCTALCLPTDDGSIVKIEAGAVGRPYPGVDVYLAATDG

IGPTAPGAGPSASFGTLWIKSPANMLGYWNNPERTAEVLIDGWVNTGDLLERREDGFFYI

KGRSSEMIICGGVNIAPDEVDRIAEGVSGVREAACYEIPDEEFGALVGLAVVASAELDES

AARALKHTIAARFRRESEPMARPSTIVIVTDIPRTQSGKVMRASLAAAATADKARVVVRG

>mtf:TBFG_10100 K12422 long chain fatty acid CoA ligase FadD10 [EC:6.2.1.-] | (GenBank) fatty-acid-CoA ligase fadD10 (A)

MGGKKFQAMPQLPSTVLDRVFEQARQQPEAIALRRCDGTSALRYRELVAEVGGLAADLRA

QSVSRGSRVLVISDNGPETYLSVLACAKLGAIAVMADGNLPIAAIERFCQITDPAAALVA

PGSKMASSAVPEALHSIPVIAVDIAAVTRESEHSLDAASLAGNADQGSEDPLAMIFTSGT

TGEPKAVLLANRTFFAVPDILQKEGLNWVTWVVGETTYSPLPATHIGGLWWILTCLMHGG

LCVTGGENTTSLLEILTTNAVATTCLVPTLLSKLVSELKSANATVPSLRLVGYGGSRAIA

ADVRFIEATGVRTAQVYGLSETGCTALCLPTDDGSIVKIEAGAVGRPYPGVDVYLAATDG

IGPTAPGAGPSASFGTLWIKSPANMLGYWNNPERTAEVLIDGWVNTGDLLERREDGFFYI

KGRSSEMIICGGVNIAPDEVDRIAEGVSGVREAACYEIPDEEFGALVGLAVVASAELDES

AARALKHTIAARFRRESEPMARPSTIVIVTDIPRTQSGKVMRASLAAAATADKARVVVRG

>mtb:TBMG_00100 K12422 long chain fatty acid CoA ligase FadD10 [EC:6.2.1.-] | (GenBank) fatty-acid-CoA ligase fadD10 (A)

MGGKKFQAMPQLPSTVLDRVFEQARQQPEAIALRRCDGTSALRYRELVAEVGGLAADLRA

QSVSRGSRVLVISDNGPETYLSVLACAKLGAIAVMADGNLPIAAIERFCQITDPAAALVA

PGSKMASSAVPEALHSIPVIAVDIAAVTRESEHSLDAASLAGNADQGSEDPLAMIFTSGT

TGEPKAVLLANRTFFAVPDILQKEGLNWVTWVVGETTYSPLPATHIGGLWWILTCLMHGG

LCVTGGENTTSLLEILTTNAVATTCLVPTLLSKLVSELKSANATVPSLRLVGYGGSRAIA

ADVRFIEATGVRTAQVYGLSETGCTALCLPTDDGSIVKIEAGAVGRPYPGVDVYLAATDG

IGPTAPGAGPSASFGTLWIKSPANMLGYWNNPERTAEVLIDGWVNTGDLLERREDGFFYI

KGRSSEMIICGGVNIAPDEVDRIAEGVSGVREAACYEIPDEEFGALVGLAVVASAELDES

AARALKHTIAARFRRESEPMARPSTIVIVTDIPRTQSGKVMRASLAAAATADKARVVVRG

>mtk:TBSG_00100 K12422 long chain fatty acid CoA ligase FadD10 [EC:6.2.1.-] | (GenBank) fatty-acid-CoA ligase fadD10 (A)

MGGKKFQAMPQLPSTVLDRVFEQARQQPEAIALRRCDGTSALRYRELVAEVGGLAADLRA

QSVSRGSRVLVISDNGPETYLSVLACAKLGAIAVMADGNLPIAAIERFCQITDPAAALVA

PGSKMASSAVPEALHSIPVIAVDIAAVTRESEHSLDAASLAGNADQGSEDPLAMIFTSGT

TGEPKAVLLANRTFFAVPDILQKEGLNWVTWVVGETTYSPLPATHIGGLWWILTCLMHGG

LCVTGGENTTSLLEILTTNAVATTCLVPTLLSKLVSELKSANATVPSLRLVGYGGSRAIA

ADVRFIEATGVRTAQVYGLSETGCTALCLPTDDGSIVKIEAGAVGRPYPGVDVYLAATDG

IGPTAPGAGPSASFGTLWIKSPANMLGYWNNPERTAEVLIDGWVNTGDLLERREDGFFYI

KGRSSEMIICGGVNIAPDEVDRIAEGVSGVREAACYEIPDEEFGALVGLAVVASAELDES

AARALKHTIAARFRRESEPMARPSTIVIVTDIPRTQSGKVMRASLAAAATADKARVVVRG

>mtz:TBXG_000100 K12422 long chain fatty acid CoA ligase FadD10 [EC:6.2.1.-] | (GenBank) fatty-acid-CoA ligase fadD10 (A)

MGGKKFQAMPQLPSTVLDRVFEQARQQPEAIALRRCDGTSALRYRELVAEVGGLAADLRA

QSVSRGSRVLVISDNGPETYLSVLACAKLGAIAVMADGNLPIAAIERFCQITDPAAALVA

PGSKMASSAVPEALHSIPVIAVDIAAVTRESEHSLDAASLAGNADQGSEDPLAMIFTSGT

TGEPKAVLLANRTFFAVPDILQKEGLNWVTWVVGETTYSPLPATHIGGLWWILTCLMHGG

LCVTGGENTTSLLEILTTNAVATTCLVPTLLSKLVSELKSANATVPSLRLVGYGGSRAIA

ADVRFIEATGVRTAQVYGLSETGCTALCLPTDDGSIVKIEAGAVGRPYPGVDVYLAATDG

IGPTAPGAGPSASFGTLWIKSPANMLGYWNNPERTAEVLIDGWVNTGDLLERREDGFFYI

KGRSSEMIICGGVNIAPDEVDRIAEGVSGVREAACYEIPDEEFGALVGLAVVASAELDES

AARALKHTIAARFRRESEPMARPSTIVIVTDIPRTQSGKVMRASLAAAATADKARVVVRG

>mtg:MRGA327_01065 K00666 fatty-acyl-CoA synthase [EC:6.2.1.-] | (GenBank) long-chain-fatty-acid--CoA ligase (A)

MSGRGVGFGDRVMILMLNRTEFVESVLAANMIGAIAVPLNFRLTPTEIAVLVEDCVAHVM

LTEAALAPVAIGVRNIQPLLSVIVVAGGSSQDSVFGYEDLLNEAGDVHEPVDIPNDSPAL

IMYTSGTTGRPKGAVLTHANLTGQAMTALYTSGANINSDVGFVGVPLFHIAGIGNMLTGL

LLGLPTVIYPLGAFDPGQLLDVLEAEKVTGIFLVPAQWQAVCTEQQARPRDLRLRVLSWG

AAPAPDALLRQMSATFPETQILAAFGQTEMSPVTCMLLGEDAIAKRGSVGRVIPTVAARV

VDQNMNDVPVGEVGEIVYRAPTLMSCYWNNPEATAEAFAGGWFHSGDLVRMDSDGYVWVV

DRKKDMIISGGENIYCAELENVLASHPDIAEVAVIGRADEKWGEVPIAVAAVTNDDLRIE

DLGEFLTDRLARYKHPKALEIVDALPRNPAGKVLKTELRLRYGACVNVERRSASAGFTER

RENRQKL

>mti:MRGA423_00635 K12422 long chain fatty acid CoA ligase FadD10 [EC:6.2.1.-] | (GenBank) acyl-CoA synthetase (A)

MGGKKFQAMPQLPSTVLDRVFEQARQQPEAIALRRCDGTSALRYRELVAEVGGLAADLRA

QSVSRGSRVLVISDNGPETYLSVLACAKLGAIAVMADGNLPIAAIERFCQITDPAAALVA

PGSKMASSAVPEALHSIPVIAVDIAAVTRESEHSLDAASLAGNADQGSEDPLAMIFTSGT

TGEPKAVLLANRTFFAVPDILQKEGLNWVTWVVGETTYSPLPATHIGGLWWILTCLMHGG

LCVTGGENTTSLLEILTTNAVATTCLVPTLLSKLVSELKSANATVPSLRLVGYGGSRAIA

ADVRFIEATGVRTAQVYGLSETGCTALCLPTDDGSIVKIEAGAVGRPYPGVDVYLAATDG

IGPTAPGAGPSASFGTLWIKSPANMLGYWNNPERTAEVLIDGWVNTGDLLERREDGFFYI

KGRSSEMIICGGVNIAPDEVDRIAEGVSGVREAACYEIPDEEFGALVGLAVVASAELDES

AARALKHTIAARFRRESEPMARPSTIVIVTDIPRTQSGKVMRASLAAAATADKARVVVRG

>mte:CCDC5079_0087 K12422 long chain fatty acid CoA ligase FadD10 [EC:6.2.1.-] | (GenBank) acyl-CoA synthetase (A)

MLDRVFEQARQQPEAIALRRCDGTSALRYRELVAEVGGLAADLRAQSVSRGSRVLVISDN

GPETYLSVLACAKLGAIAVMADGNLPIAAIERFCQITDPAAALVAPGSKMASSAVPEALH

SIPVIAVDIAAVTRESEHSLDAASLAGNADQGSEDPLAMIFTSGTTGEPKAVLLANRTFF

AVPDILQKEGLNWVTWVVGETTYSPLPATHIGGLWWILTCLMHGGLCVTGGENTTSLLEI

LTTNAVATTCLVPTLLSKLVSELKSANATVPSLRLVGYGGSRAIAADVRFIEATGVRTAQ

VYGLSETGCTALCLPTDDGSIVKIEAGAVGRPYPGVDVYLAATDGIGPTAPGAGPSASFG

TLWIKSPANMLGYWNNPERTAEVLIDGWVNTGDLLERREDGFFYIKGRSSEMIICGGVNI

APDEVDRIAEGVSGVREAACYEIPDEEFGALVGLAVVASAELDESAARALKHTIAARFRR

ESEPMARPSTIVIVTDIPRTQSGKVMRASLAAAATADKARVVVRG

>mtur:CFBS_0105 K12422 long chain fatty acid CoA ligase FadD10 [EC:6.2.1.-] | (GenBank) fadD10; fatty-acid-CoA ligase (A)

MGGKKFQAMPQLPSTVLDRVFEQARQQPEAIALRRCDGTSALRYRELVAEVGGLAADLRA

QSVSRGSRVLVISDNGPETYLSVLACAKLGAIAVMADGNLPIAAIERFCQITDPAAALVA

PGSKMASSAVPEALHSIPVIAVDIAAVTRESEHSLDAASLAGNADQGSEDPLAMIFTSGT

TGEPKAVLLANRTFFAVPDILQKEGLNWVTWVVGETTYSPLPATHIGGLWWILTCLMHGG

LCVTGGENTTSLLEILTTNAVATTCLVPTLLSKLVSELKSANATVPSLRLVGYGGSRAIA

ADVRFIEATGVRTAQVYGLSETGCTALCLPTDDGSIVKIEAGAVGRPYPGVDVYLAATDG

IGPTAPGAGPSASFGTLWIKSPANMLGYWNNPERTAEVLIDGWVNTGDLLERREDGFFYI

KGRSSEMIICGGVNIAPDEVDRIAEGVSGVREAACYEIPDEEFGALVGLAVVASAELDES

AARALKHTIAARFRRESEPMARPSTIVIVTDIPRTQSGKVMRASLAAAATADKARVVVRG

>mtl:CCDC5180_0088 K12422 long chain fatty acid CoA ligase FadD10 [EC:6.2.1.-] | (GenBank) acyl-CoA synthetase (A)

MPQLPSTVLDRVFEQARQQPEAIALRRCDGTSALRYRELVAEVGGLAADLRAQSVSRGSR

VLVISDNGPETYLSVLACAKLGAIAVMADGNLPIAAIERFCQITDPAAALVAPGSKMASS

AVPEALHSIPVIAVDIAAVTRESEHSLDAASLAGNADQGSEDPLAMIFTSGTTGEPKAVL

LANRTFFAVPDILQKEGLNWVTWVVGETTYSPLPATHIGGLWWILTCLMHGGLCVTGGEN

TTSLLEILTTNAVATTCLVPTLLSKLVSELKSANATVPSLRLVGYGGSRAIAADVRFIEA

TGVRTAQVYGLSETGCTALCLPTDDGSIVKIEAGAVGRPYPGVDVYLAATDGIGPTAPGA

GPSASFGTLWIKSPANMLGYWNNPERTAEVLIDGWVNTGDLLERREDGFFYIKGRSSEMI

ICGGVNIAPDEVDRIAEGVSGVREAACYEIPDEEFGALVGLAVVASAELDESAARALKHT

IAARFRRESEPMARPSTIVIVTDIPRTQSGKVMRASLAAAATADKARVVVRG

>mto:MTCTRI2_0102 K12422 long chain fatty acid CoA ligase FadD10 [EC:6.2.1.-] | (GenBank) fadD10; acyl-CoA synthetase (A)

MGGKKFQAMPQLPSTVLDRVFEQARQQPEAIALRRCDGTSALRYRELVAEVGGLAADLRA

QSVSRGSRVLVISDNGPETYLSVLACAKLGAIAVMADGNLPIAAIERFCQITDPAAALVA

PGSKMASSAVPEALHSIPVIAVDIAAVTRESEHSLDAASLAGNADQGSEDPLAMIFTSGT

TGEPKAVLLANRTFFAVPDILQKEGLNWVTWVVGETTYSPLPATHIGGLWWILTCLMHGG

LCVTGGENTTSLLEILTTNAVATTCLVPTLLSKLVSELKSANATVPSLRLVGYGGSRAIA

ADVRFIEATGVRTAQVYGLSETGCTALCLPTDDGSIVKIEAGAVGRPYPGVDVYLAATDG

IGPTAPGAGPSASFGTLWIKSPANMLGYWNNPERTAEVLIDGWVNTGDLLERREDGFFYI

KGRSSEMIICGGVNIAPDEVDRIAEGVSGVREAACYEIPDEEFGALVGLAVVASAELDES

AARALKHTIAARFRRESEPMARPSTIVIVTDIPRTQSGKVMRASLAAAATADKARVVVRG

>mtd:UDA_0099 K12422 long chain fatty acid CoA ligase FadD10 [EC:6.2.1.-] | (GenBank) fadD10; fadD10 (A)

MGGKKFQAMPQLPSTVLDRVFEQARQQPEAIALRRCDGTSALRYRELVAEVGGLAADLRA

QSVSRGSRVLVISDNGPETYLSVLACAKLGAIAVMADGNLPIAAIERFCQITDPAAALVA

PGSKMASSAVPEALHSIPVIAVDIAAVTRESEHSLDAASLAGNADQGSEDPLAMIFTSGT

TGEPKAVLLANRTFFAVPDILQKEGLNWVTWVVGETTYSPLPATHIGGLWWILTCLMHGG

LCVTGGENTTSLLEILTTNAVATTCLVPTLLSKLVSELKSANATVPSLRLVGYGGSRAIA

ADVRFIEATGVRTAQVYGLSETGCTALCLPTDDGSIVKIEAGAVGRPYPGVDVYLAATDG

IGPTAPGAGPSASFGTLWIKSPANMLGYWNNPERTAEVLIDGWVNTGDLLERREDGFFYI

KGRSSEMIICGGVNIAPDEVDRIAEGVSGVREAACYEIPDEEFGALVGLAVVASAELDES

AARALKHTIAARFRRESEPMARPSTIVIVTDIPRTQSGKVMRASLAAAATADKARVVVRG

>mtn:ERDMAN_0116 K12422 long chain fatty acid CoA ligase FadD10 [EC:6.2.1.-] | (GenBank) fadD10; acyl-CoA synthetase (A)

MLDRVFEQARQQPEAIALRRCDGTSALRYRELVAEVGGLAADLRAQSVSRGSRVLVISDN

GPETYLSVLACAKLGAIAVMADGNLPIAAIERFCQITDPAAALVAPGSKMASSAVPEALH

SIPVIAVDIAAVTRESEHSLDAASLAGNADQGSEDPLAMIFTSGTTGEPKAVLLANRTFF

AVPDILQKEGLNWVTWVVGETTYSPLPATHIGGLWWILTCLMHGGLCVTGGENTTSLLEI

LTTNAVATTCLVPTLLSKLVSELKSANATVPSLRLVGYGGSRAIAADVRFIEATGVRTAQ

VYGLSETGCTALCLPTDDGSIVKIEAGAVGRPYPGVDVYLAATDGIGPTAPGAGPSASFG

TLWIKSPANMLGYWNNPERTAEVLIDGWVNTGDLLERREDGFFYIKGRSSEMIICGGVNI

APDEVDRIAEGVSGVREAACYEIPDEEFGALVGLAVVASAELDESAARALKHTIAARFRR

ESEPMARPSTIVIVTDIPRTQSGKVMRASLAAAATADKARVVVRG

>mtj:J112_00550 K12422 long chain fatty acid CoA ligase FadD10 [EC:6.2.1.-] | (GenBank) acyl-CoA synthetase (A)

MGGKKFQAMPQLPSTVLDRVFEQARQQPEAIALRRCDGTSALRYRELVAEVGGLAADLRA

QSVSRGSRVLVISDNGPETYLSVLACAKLGAIAVMADGNLPIAAIERFCQITDPAAALVA

PGSKMASSAVPEALHSIPVIAVDIAAVTRESEHSLDAASLAGNADQGSEDPLAMIFTSGT

TGEPKAVLLANRTFFAVPDILQKEGLNWVTWVVGETTYSPLPATHIGGLWWILTCLMHGG

LCVTGGENTTSLLEILTTNAVATTCLVPTLLSKLVSELKSANATVPSLRLVGYGGSRAIA

ADVRFIEATGVRTAQVYGLSETGCTALCLPTDDGSIVKIEAGAVGRPYPGVDVYLAATDG

IGPTAPGAGPSASFGTLWIKSPANMLGYWNNPERTAEVLIDGWVNTGDLLERREDGFFYI

KGRSSEMIICGGVNIAPDEVDRIAEGVSGVREAACYEIPDEEFGALVGLAVVASAELDES

AARALKHTIAAPFRRESEPMARPSTIVIVTDIPRTQSGKVMRASLAAAATADKARVVVRG

>mtub:MT7199_0101 K12422 long chain fatty acid CoA ligase FadD10 [EC:6.2.1.-] | (GenBank) putative FATTY-ACID-CoA LIGASE FADD10 (FATTY-ACID-CoA SYNTHETASE) (FATTY-ACID-CoA SYNTHASE) (A)

MGGKKFQAMPQLPSTVLDRVFEQARQQPEAIALRRCDGTSALRYRELVAEVGGLAADLRA

QSVSRGSRVLVISDNGPETYLSVLACAKLGAIAVMADGNLPIAAIERFCQITDPAAALVA

PGSKMASSAVPEALHSIPVIAVDIAAVTRESEHSLDAASLAGNADQGSEDPLAMIFTSGT

TGEPKAVLLANRTFFAVPDILQKEGLNWVTWVVGETTYSPLPATHIGGLWWILTCLMHGG

LCVTGGENTTSLLEILTTNAVATTCLVPTLLSKLVSELKSANATVPSLRLVGYGGSRAIA

ADVRFIEATGVRTAQVYGLSETGCTALCLPTDDGSIVKIEAGAVGRPYPGVDVYLAATDG

IGPTAPGAGPSASFGTLWIKSPANMLGYWNNPERTAEVLIDGWVNTGDLLERREDGFFYI

KGRSSEMIICGGVNIAPDEVDRIAEGVSGVREAACYEIPDEEFGALVGLAVVASAELDES

AARALKHTIAARFRRESEPMARPSTIVIVTDIPRTQSGKVMRASLAAAATADKARVVVRG

>mtuc:J113_00670 K12422 long chain fatty acid CoA ligase FadD10 [EC:6.2.1.-] | (GenBank) acyl-CoA synthetase (A)

MGGKKFQAMPQLPSTVLDRVFEQARQQPEAIALRRCDGTSALRYRELVAEVGGLAADLRA

QSVSRGSRVLVISDNGPETYLSVLACAKLGAIAVMADGNLPIAAIERFCQITDPAAALVA

PGSKMASSAVPEALHSIPVIAVDIAAVTRESEHSLDAASLAGNADQGSEDPVAMIFTSGT

TGEPKAVLLANRTFFAVPDILQKEGLNWVTWVVGETTYSPLPATHIGGLWWILTCLMHGG

LCVTGGENTTSLLEILTTNAVATTCLVPTLLSKLVSELKSANATVPSLRLVGYGGSRAIA

ADVRFIEATGVRTAQVYGLSETGCTALCLPTDDGSIVKIEAGAVGRPYPGVDVYLAATDG

IGPTAPGAGPSASFGTLWIKSPANMLGYWNNPERTAEVLIDGWVNTGDLLERREDGFFYI

KGRSSEMIICGGVNIAPDEVDRIAEGVSGVREAACYEIPDEEFGALVGLAVVASAELDES

AARALKHTMVVGSSEPMARPSTIVIVTDIPRTQSGKVMRASLAAAATADKARVVVRG

>mtue:J114_00555 K12422 long chain fatty acid CoA ligase FadD10 [EC:6.2.1.-] | (GenBank) acyl-CoA synthetase (A)

MLQLPSTVLDRVFEQARQQPEAIALRRCDGTSALRYRELVAEVGGLAADLRAQSVSRGSR

VLVISDNGPETYLSVLACAKLGAIAVMADGNLPIAAIERFCQITDPAAALVAPGSKMASS

AVPEALHSIPVIAVDIAAVTRESEHSLDAASLAGNADQGSEDPLAMIFTSGTTGEPKAVL

LANRTFFAVPDILQKEGLNWVTWVVGETTYSPLPATHIGGLWWILTCLMHGGLCVTGGEN

TTSLLEILTTNAVATTCLVPTLLSKLVSELKSANATVPSLRLVGYGGSRAIAADVRFIEA

TGVRTAQVYGLSETGCTALCLPTDDGSIVKIEAGAVGRPYPGVDVYLAATDGIGPTAPGA

GPSASFGTLWIKSPANMLGYWNNPERTAEVLIDGWVNTGDLLERREDGFFYIKGRSSEMI

ICGGVNIAPDEVDRIAEGVSGVREAACYEIPDEEFGALVGLAVVASAELDESAARALKHT

IAAPFSTGVRADGAAVDNCDRHRHSTNAVRQGHAGLACSGGNSRQGQSGRSWLSRCGTES

SPPSATCCISTRRISLMATKRISATSGWTLFGLFC

>mtx:M943_00555 K12422 long chain fatty acid CoA ligase FadD10 [EC:6.2.1.-] | (GenBank) acyl-CoA synthetase (A)

MGGKKFQAMLQLPSTVLDRVFEQARQQPEAIALRRCDGTSALRYRELVAEVGGLAADLRA

QSVSRGSRVLVISDNGPETYLSVLACAKLGAIAVMADGNLPIAAIERFCQITDPAAALVA

PGSKMASSAVPEALHSIPVIAVDIAAVTRESEHSLDAASLAGNADQGSEDPLAMIFTSGT

TGEPKAVLLANRTFFAVPDILQKEGLNWVTWVVGETTYSPLPATHIGGLWWILTCLMHGG

LCVTGGENTTSLLEILTTNAVATTCLVPTLLSKLVSELKSANATVPSLRLVGYGGSRAIA

ADVRFIEATGVRTAQVYGLSETGCTALCLPTDDGSIVKIEAGAVGRPYPGVDVYLAATDG

IGPTAPGAGPSASFGTLWIKSPANMLGYWNNPERTAEVLIDGWVNTGDLLERREDGFFYI

KGRSSEMIICGGVNIAPDEVDRIAEGVSGVREAACYEIPDEEFGALVGLAVVASAELDES

AARALKHTIAARFRRESEPMARPSTIVIVTDIPRTQSGKVMRASLAAAATADKARVVVRG

>mtuh:I917_00710 K12422 long chain fatty acid CoA ligase FadD10 [EC:6.2.1.-] | (GenBank) acyl-CoA synthetase (A)

MGGKKFQAMPQLPSTVLDRVFEQARQQPEAIALRRCDGTSALRYRELVAEVGGLAADLRA

QSVSRGSRVLVISDNGPETYLSVLACAKLGAIAVMADGNLPIAAIERFCQITDPAAALVA

PGSKMASSAVPEALHSIPVIAVDIAAVTRESEHSLDAASLAGNADQGSEDPLAMIFTSGT

TGEPKAVLLANRTFFAVPDILQKEGLNWVTWVVGETTYSPLPATHIGGLWWILTCLMHGG

LCVTGGENTTSLLEILTTNAVATTCLVPTLLSKLVSELKSANATVPSLRLVGYGGSRAIA

ADVRFIEATGVRTAQVYGLSETGCTALCLPTDDGSIVKIEAGAVGRPYPGVDVYLAATDG

IGPTAPGAGPSASFGTLWIKSPANMLGYWNNPERTAEVLIDGWVNTGDLLERREDGFFYI

KGRSSEMIICGGVNIAPDEVDRIAEGVSGVREAACYEIPDEEFGALVGLAVVASAELDES

AARALKHTIAARFRRESEPMARPXXXVIVTDIPRTXSGKVMXASLAAAATADKARVVVRG

>mtul:TBHG_00100 K12422 long chain fatty acid CoA ligase FadD10 [EC:6.2.1.-] | (GenBank) fatty-acid-CoA ligase FadD10 (A)

MGGKKFQAMPQLPSTVLDRVFEQARQQPEAIALRRCDGTSALRYRELVAEVGGLAADLRA

QSVSRGSRVLVISDNGPETYLSVLACAKLGAIAVMADGNLPIAAIERFCQITDPAAALVA

PGSKMASSAVPEALHSIPVIAVDIAAVTRESEHSLDAASLAGNADQGSEDPLAMIFTSGT

TGEPKAVLLANRTFFAVPDILQKEGLNWVTWVVGETTYSPLPATHIGGLWWILTCLMHGG

LCVTGGENTTSLLEILTTNAVATTCLVPTLLSKLVSELKSANATVPSLRLVGYGGSRAIA

ADVRFIEATGVRTAQVYGLSETGCTALCLPTDDGSIVKIEAGAVGRPYPGVDVYLAATDG

IGPTAPGAGPSASFGTLWIKSPANMLGYWNNPERTAEVLIDGWVNTGDLLERREDGFFYI

KGRSSEMIICGGVNIAPDEVDRIAEGVSGVREAACYEIPDEEFGALVGLAVVASAELDES

AARALKHTIAARFRRESEPMARPSTIVIVTDIPRTQSGKVMRASLAAAATADKARVVVRG

>mtut:HKBT1_0105 K12422 long chain fatty acid CoA ligase FadD10 [EC:6.2.1.-] | (GenBank) fadD10; fatty-acid-CoA ligase (A)

MGGKKFQAMPQLPSTVLDRVFEQARQQPEAIALRRCDGTSALRYRELVAEVGGLAADLRA

QSVSRGSRVLVISDNGPETYLSVLACAKLGAIAVMADGNLPIAAIERFCQITDPAAALVA

PGSKMASSAVPEALHSIPVIAVDIAAVTRESEHSLDAASLAGNADQGSEDPLAMIFTSGT

TGEPKAVLLANRTFFAVPDILQKEGLNWVTWVVGETTYSPLPATHIGGLWWILTCLMHGG

LCVTGGENTTSLLEILTTNAVATTCLVPTLLSKLVSELKSANATVPSLRLVGYGGSRAIA

ADVRFIEATGVRTAQVYGLSETGCTALCLPTDDGSIVKIEAGAVGRPYPGVDVYLAATDG

IGPTAPGAGPSASFGTLWIKSPANMLGYWNNPERTAEVLIDGWVNTGDLLERREDGFFYI

KGRSSEMIICGGVNIAPDEVDRIAEGVSGVREAACYEIPDEEFGALVGLAVVASAELDES

AARALKHTIAARFRRESEPMARPSTIVIVTDIPRTQSGKVMRASLAAAATADKARVVVRG

>mtuu:HKBT2_0105 K12422 long chain fatty acid CoA ligase FadD10 [EC:6.2.1.-] | (GenBank) fadD10; fatty-acid-CoA ligase (A)

MGGKKFQAMPQLPSTVLDRVFEQARQQPEAIALRRCDGTSALRYRELVAEVGGLAADLRA

QSVSRGSRVLVISDNGPETYLSVLACAKLGAIAVMADGNLPIAAIERFCQITDPAAALVA

PGSKMASSAVPEALHSIPVIAVDIAAVTRESEHSLDAASLAGNADQGSEDPLAMIFTSGT

TGEPKAVLLANRTFFAVPDILQKEGLNWVTWVVGETTYSPLPATHIGGLWWILTCLMHGG

LCVTGGENTTSLLEILTTNAVATTCLVPTLLSKLVSELKSANATVPSLRLVGYGGSRAIA

ADVRFIEATGVRTAQVYGLSETGCTALCLPTDDGSIVKIEAGAVGRPYPGVDVYLAATDG

IGPTAPGAGPSASFGTLWIKSPANMLGYWNNPERTAEVLIDGWVNTGDLLERREDGFFYI

KGRSSEMIICGGVNIAPDEVDRIAEGVSGVREAACYEIPDEEFGALVGLAVVASAELDES

AARALKHTIAARFRRESEPMARPSTIVIVTDIPRTQSGKVMRASLAAAATADKARVVVRG

>mtq:HKBS1_0105 K12422 long chain fatty acid CoA ligase FadD10 [EC:6.2.1.-] | (GenBank) fadD10; fatty-acid-CoA ligase (A)

MGGKKFQAMPQLPSTVLDRVFEQARQQPEAIALRRCDGTSALRYRELVAEVGGLAADLRA

QSVSRGSRVLVISDNGPETYLSVLACAKLGAIAVMADGNLPIAAIERFCQITDPAAALVA

PGSKMASSAVPEALHSIPVIAVDIAAVTRESEHSLDAASLAGNADQGSEDPLAMIFTSGT

TGEPKAVLLANRTFFAVPDILQKEGLNWVTWVVGETTYSPLPATHIGGLWWILTCLMHGG

LCVTGGENTTSLLEILTTNAVATTCLVPTLLSKLVSELKSANATVPSLRLVGYGGSRAIA

ADVRFIEATGVRTAQVYGLSETGCTALCLPTDDGSIVKIEAGAVGRPYPGVDVYLAATDG

IGPTAPGAGPSASFGTLWIKSPANMLGYWNNPERTAEVLIDGWVNTGDLLERREDGFFYI

KGRSSEMIICGGVNIAPDEVDRIAEGVSGVREAACYEIPDEEFGALVGLAVVASAELDES

AARALKHTIAARFRRESEPMARPSTIVIVTDIPRTQSGKVMRASLAAAATADKARVVVRG

>mbo:Mb0102 K12422 long chain fatty acid CoA ligase FadD10 [EC:6.2.1.-] | (RefSeq) fadD10; acyl-CoA synthetase (A)

MGGKKFQAMPQLPSTVLDRVFEQARQQPEAIALRRCDGTSALRYRELVAEVGGLAADLRA

QSVSRGSRVLVISDNGPETYLSVLACAKLGAIAVMADGNLPIAAIERFCQITDPAAALVA

PGSKMASSAVPEALHSIPVIAVDIAAVTRESEHSLDAASLAGNADQGSEDPLAMIFTSGT

TGEPKAVLLANRTFFAVPDILQKEGLNWVTWVVGETTYSPLPATHIGGLWWILTCLMHGG

LCVTGGENTTSLLEILTTNAVATTCLVPTLLSKLVSELKSANATVPSLRLVGYGGSRAIA

ADVRFIEATGVRTAQVYGLSETGCTALCLPTDDGSIVKIEAGAVGRPYPGVDVYLAATDG

IGPTAPGAGPSASFGTLWIKSPANMLGYWNNPERTAEVLIDGWVNTGDLLERREDGFFYI

KGRSSEMIICGGVNIAPDEVDRIAEGVSGVREAACYEIPDEEFGALVGLAVVASAELDES

AARALKHTIAARFRRESEPMARPSTIVIVTDIPRTQSGKVMRASLAAAATADKARVVVRG

>mbb:BCG_0132 K12422 long chain fatty acid CoA ligase FadD10 [EC:6.2.1.-] | (GenBank) fadD10; Possible fatty-acid-CoA ligase fadD10 (A)

MGGKKFQAMPQLPSTVLDRVFEQARQQPEAIALRRCDGTSALRYRELVAEVGGLAADLRA

QSVSRGSRVLVISDNGPETYLSVLACAKLGAIAVMADGNLPIAAIERFCQITDPAAALVA

PGSKMASSAVPEALHSIPVIAVDIAAVTRESEHSLDAASLAGNADQGSEDPLAMIFTSGT

TGEPKAVLLANRTFFAVPDILQKEGLNWVTWVVGETTYSPLPATHIGGLWWILTCLMHGG

LCVTGGENTTSLLEILTTNAVATTCLVPTLLSKLVSELKSANATVPSLRLVGYGGSRAIA

ADVRFIEATGVRTAQVYGLSETGCTALCLPTDDGSIVKIEAGAVGRPYPGVDVYLAATDG

IGPTAPGAGPSASFGTLWIKSPANMLGYWNNPERTAEVLIDGWVNTGDLLERREDGFFYI

KGRSSEMIICGGVNIAPDEVDRIAEGVSGVREAACYEIPDEEFGALVGLAVVASAELDES

AARALKHTIAARFRRESEPMARPSTIVIVTDIPRTQSGKVMRASLAAAATADKARVVVRG

>mbt:JTY_0103 K12422 long chain fatty acid CoA ligase FadD10 [EC:6.2.1.-] | (GenBank) fadD10; acyl-CoA synthetase (A)

MGGKKFQAMPQLPSTVLDRVFEQARQQPEAIALRRCDGTSALRYRELVAEVGGLAADLRA

QSVSRGSRVLVISDNGPETYLSVLACAKLGAIAVMADGNLPIAAIERFCQITDPAAALVA

PGSKMASSAVPEALHSIPVIAVDIAAVTRESEHSLDAASLAGNADQGSEDPLAMIFTSGT

TGEPKAVLLANRTFFAVPDILQKEGLNWVTWVVGETTYSPLPATHIGGLWWILTCLMHGG

LCVTGGENTTSLLEILTTNAVATTCLVPTLLSKLVSELKSANATVPSLRLVGYGGSRAIA

ADVRFIEATGVRTAQVYGLSETGCTALCLPTDDGSIVKIEAGAVGRPYPGVDVYLAATDG

IGPTAPGAGPSASFGTLWIKSPANMLGYWNNPERTAEVLIDGWVNTGDLLERREDGFFYI

KGRSSEMIICGGVNIAPDEVDRIAEGVSGVREAACYEIPDEEFGALVGLAVVASAELDES

AARALKHTIAARFRRESEPMARPSTIVIVTDIPRTQSGKVMRASLAAAATADKARVVVRG

>mbm:BCGMEX_0103 K12422 long chain fatty acid CoA ligase FadD10 [EC:6.2.1.-] | (GenBank) fadD10; putative fatty-acid-CoA ligase (A)

MGGKKFQAMPQLPSTVLDRVFEQARQQPEAIALRRCDGTSALRYRELVAEVGGLAADLRA

QSVSRGSRVLVISDNGPETYLSVLACAKLGAIAVMADGNLPIAAIERFCQITDPAAALVA

PGSKMASSAVPEALHSIPVIAVDIAAVTRESEHSLDAASLAGNADQGSEDPLAMIFTSGT

TGEPKAVLLANRTFFAVPDILQKEGLNWVTWVVGETTYSPLPATHIGGLWWILTCLMHGG

LCVTGGENTTSLLEILTTNAVATTCLVPTLLSKLVSELKSANATVPSLRLVGYGGSRAIA

ADVRFIEATGVRTAQVYGLSETGCTALCLPTDDGSIVKIEAGAVGRPYPGVDVYLAATDG

IGPTAPGAGPSASFGTLWIKSPANMLGYWNNPERTAEVLIDGWVNTGDLLERREDGFFYI

KGRSSEMIICGGVNIAPDEVDRIAEGVSGVREAACYEIPDEEFGALVGLAVVASAELDES

AARALKHTIAARFRRESEPMARPSTIVIVTDIPRTQSGKVMRASLAAAATADKARVVVRG

>mbk:K60_001110 K12422 long chain fatty acid CoA ligase FadD10 [EC:6.2.1.-] | (GenBank) acyl-CoA synthetase (A)

MGGKKFQAMPQLPSTVLDRVFEQARQQPEAIALRRCDGTSALRYRELVAEVGGLAADLRA

QSVSRGSRVLVISDNGPETYLSVLACAKLGAIAVMADGNLPIAAIERFCQITDPAAALVA

PGSKMASSAVPEALHSIPVIAVDIAAVTRESEHSLDAASLAGNADQGSEDPLAMIFTSGT

TGEPKAVLLANRTFFAVPDILQKEGLNWVTWVVGETTYSPLPATHIGGLWWILTCLMHGG

LCVTGGENTTSLLEILTTNAVATTCLVPTLLSKLVSELKSANATVPSLRLVGYGGSRAIA

ADVRFIEATGVRTAQVYGLSETGCTALCLPTDDGSIVKIEAGAVGRPYPGVDVYLAATDG

IGPTAPGAGPSASFGTLWIKSPANMLGYWNNPERTAEVLIDGWVNTGDLLERREDGFFYI

KGRSSEMIICGGVNIAPDEVDRIAEGVSGVREAACYEIPDEEFGALVGLAVVASAELDES

AARALKHTIAARFRRESEPMARPSTIVIVTDIPRTQSGKVMRASLAAAATADKARVVVRG

>mbx:BCGT_3894 K12422 long chain fatty acid CoA ligase FadD10 [EC:6.2.1.-] | (GenBank) Long-chain-fatty-acid--CoA ligase (A)

MLDRVFEQARQQPEAIALRRCDGTSALRYRELVAEVGGLAADLRAQSVSRGSRVLVISDN

GPETYLSVLACAKLGAIAVMADGNLPIAAIERFCQITDPAAALVAPGSKMASSAVPEALH

SIPVIAVDIAAVTRESEHSLDAASLAGNADQGSEDPLAMIFTSGTTGEPKAVLLANRTFF

AVPDILQKEGLNWVTWVVGETTYSPLPATHIGGLWWILTCLMHGGLCVTGGENTTSLLEI

LTTNAVATTCLVPTLLSKLVSELKSANATVPSLRLVGYGGSRAIAADVRFIEATGVRTAQ

VYGLSETGCTALCLPTDDGSIVKIEAGAVGRPYPGVDVYLAATDGIGPTAPGAGPSASFG

TLWIKSPANMLGYWNNPERTAEVLIDGWVNTGDLLERREDGFFYIKGRSSEMIICGGVNI

APDEVDRIAEGVSGVREAACYEIPDEEFGALVGLAVVASAELDESAARALKHTIAARFRR

ESEPMARPSTIVIVTDIPRTQSGKVMRASLAAAATADKARVVVRG

>mbz:LH58_00565 K12422 long chain fatty acid CoA ligase FadD10 [EC:6.2.1.-] | (GenBank) acyl-CoA synthetase (A)

MPQLPSTVLDRVFEQARQQPEAIALRRCDGTSALRYRELVAEVGGLAADLRAQSVSRGSR

VLVISDNGPETYLSVLACAKLGAIAVMADGNLPIAAIERFCQITDPAAALVAPGSKMASS

AVPEALHSIPVIAVDIAAVTRESEHSLDAASLAGNADQGSEDPLAMIFTSGTTGEPKAVL

LANRTFFAVPDILQKEGLNWVTWVVGETTYSPLPATHIGGLWWILTCLMHGGLCVTGGEN

TTSLLEILTTNAVATTCLVPTLLSKLVSELKSANATVPSLRLVGYGGSRAIAADVRFIEA

TGVRTAQVYGLSETGCTALCLPTDDGSIVKIEAGAVGRPYPGVDVYLAATDGIGPTAPGA

GPSASFGTLWIKSPANMLGYWNNPERTAEVLIDGWVNTGDLLERREDGFFYIKGRSSEMI

ICGGVNIAPDEVDRIAEGVSGVREAACYEIPDEEFGALVGLAVVASAELDESAARALKHT

IAARFRRESEPMARPSTIVIVTDIPRTQSGKVMRASLAAAATADKARVVVRG

>maf:MAF_01000 K12422 long chain fatty acid CoA ligase FadD10 [EC:6.2.1.-] | (GenBank) fadD10; putative fatty-acid-CoA ligase FadD10 (A)

MGGKKFQAMPQLPSTVLDRVFEQARQQPEAIALRRCDGTSALRYRELVAEVGGLAADLRA

QSVSRGSRVLVISDNGPETYLSVLACAKLGAIAVMADGNLPIAAIERFCQITDPAAALVA

PGSKMASSAVPEALHSIPVIAVDIAAVTRESEHSLDAASLAGNADQGSEDPLAMIFTSGT

TGEPKAVLLANRTFFAVPDILQKEGLNWVTWVVGETTYSPLPATHIGGLWWILTCLMHGG

LCVTGGENTTSLLEILTTNAVATTCLVPTLLSKLVSELKSANATVPSLRLVGYGGSRAIA

ADVRFIEATGVRTAQVYGLSETGCTALCLPTDDGSIVKIEAGAVGRPYPGVDVYLAATDG

IGPTAPGAGPSASFGTLWIKSPANMLGYWNNPERTAEVLIDGWVNTGDLLERREDGFFYI

KGRSSEMIICGGVNIAPDEVDRIAEGVSGVREAACYEIPDEEFGALVGLAVVASAELDES

AARALKHTIAARFRRESEPMARPSTIVIVTDIPRTQSGKVMRASLAAAATADKARVVVRG

>mce:MCAN_01021 K12422 long chain fatty acid CoA ligase FadD10 [EC:6.2.1.-] | (GenBank) fadD10; putative fatty-acid-CoA ligase FADD10 (fatty-acid-CoA synthetase) (fatty-acid-CoA synthase) (A)

MGGKKFQAMPQLPSTVLDRVFEQARQRPEAIALRRCDGTSALRYRELVAEVGGLAAALRA

QSVTRGSRVLVISDNGPETYLSVLACAKLGAIAVMADGNLPIAAIERFCQITDPAAALVA

PGSKMASSAVPEALHSIPVIAVDIAAVTRESEHSLDAASLAGNADQGSEDPLAMIFTSGT

TGEPKAVLLANRTFFAVPDILQKEGLNWITWVVGETTYSPLPATHIGGLWWILTCLMHGG

LCVTGGENTTSLLEILTTNAVATTCLVPTLLSKLVSELKSANATVPSLRLVGYGGSRAIA

ADVRFIEATGVRTAQVYGLSETGCTALCLPTDDGSIAKIEAGAVGRPYPGVDVYLAATNG

IGPTAPGAGPSASFGTLWIKSPANMLGYWNNPERTAEVLIDGWVNTGDLLERREDGYFYI

KGRSSEMIICGGVNIAPDEVDRIAEGVPGVREAACYEIPDEEFGALVGLAVVASAELDES

AARALKHTISARFRRESEPMARPSIIEIVTDIPRTQSGKVMRASLAAATTADKARVVVRG

>mcq:BN44_10122 K12422 long chain fatty acid CoA ligase FadD10 [EC:6.2.1.-] | (GenBank) fadD; Putative fatty-acid-CoA ligase FadD10 (fatty-acid-CoA synthetase) (fatty-acid-CoA synthase) (A)

MGGKKFQAMPQLPSTVLDRVFEQARQQPEAIALRRCDGTSALRYRELVAEVGGLAADLRA

QSVSRGSRVLVISDNGPETYLSVLACAKLGAIAVMADGNLPIAAIERFCQITDPAAALVA

PGSKMASSAVPEALHSIPVIAVDIAAVTRESEHSLDAASLAGNADQGSEDPLAMIFTSGT

TGEPKAVLLANRTFFAVPDILQKEGLNWVTWVVGETTYSPLPATHIGGLWWILTCLMHGG

LCVTGGENTTSLLEILTTNAVATTCLVPTLLSKLVSELKSANATVPSLRLVGYGGSRAIA

ADVRFIEATGVRTAQVYGLSETGCTALCLPTDDGSIVKIEAGAVGRPYPGVDVYLAATDG

IGPTAPGAGPSASFGTLWIKSPANMLGYWNNPERTAEVLIDGWVNTGDLLERREDGFFYI

KGRSSEMIICGGVNIAPDEVDRIAEGVSGVREAACYEIPDEEFGALVGLAVVASAELDES

AARALKHTIAARFRRESEPMARPSIIEIVTDIPRTQSGKVMRASLAAATTADKARVVVRG

>mcv:BN43_10117 K12422 long chain fatty acid CoA ligase FadD10 [EC:6.2.1.-] | (GenBank) fadD; Putative fatty-acid-CoA ligase FadD10 (fatty-acid-CoA synthetase) (fatty-acid-CoA synthase) (A)

MGGKKFQAMPQLPSTVLDRVFEQARQQPEAIALRRCDGTSALRYRELVAEVGGLAAALRA

QSVTRGSRVLVISDNGPETYLSVLACAKLGAIAVMADGNLPIAAIERFCQITNPAAALVA

PGSKMASSAVPEALHSIPVIAVDIAAAARESEHSLDAASLAGNADQASEDPLAMIFTSGT

TGEPKAVLLANRTFFAVPDILQKEGLNWVTWVVGETTYSPLPAAHIGGLWWILTCLMHGG

LCVTGGENTTSLLETLTTNAVATTCLVPTLLSKLVSELKSANATVPSLRLVGYGGSRAIA

ADVRFIEATGVRTAQVYGLSETGCTALCLPTDDGSIAKIEAGAVGRPYPGVDVYLAATDG

IGPTAPGAGPSASFGTLWIKSPANMLGYWNNPERTAEVLIDGWVNTGDLLERREDGFFYI

KGRSSEMIICGGVNIAPDEVDRIAEGVPGVREAACYEIPDEEFGALVGLAVVASAELDES

AARALKHTIAARFRRESEPMARPSTIVIVTDIPRTQSGKVMRASLAAATTADKARVVVRG

>mcx:BN42_10137 K12422 long chain fatty acid CoA ligase FadD10 [EC:6.2.1.-] | (GenBank) fadD; Putative fatty-acid-CoA ligase FadD10 (fatty-acid-CoA synthetase) (fatty-acid-CoA synthase) (A)

MGGKKFQAMPQLPSTVLDRVFEQARQRPEAIALRRCDGTSALRYRELVAEVGGLAAALRA

QSVARGSRVLVISDNGPETYLSVLACAKLGAIAVMADGNLPIAAIERFCQITYPAAALVA

PGSRMASSAVPEALHSIPVIAVDIAAAARESEHSLDAASLAGNADQGSEDPLAMIFTSGT

TGEPKAVLLANRTFFAVPDILQKEGLNWITWVVGETTYSPLPATHIGGLWWILTCLMHGG

LCVTGGENTTSLLETLTTNAAATTCLVPTLLSKLVSELKSANATVPSLRLVGYGGSRAIA

ADVRFIEATGVRTAQVYGLSETGCTALCLPTDDGSIAKIEAGAVGRPYPGVDVYLAATDG

IGPTAPGAGPSASFGTLWIKSPANMLGYWNNPERTAEVLIDGWVNTGDLLERREDGFFYI

KGRSSEMIICGGVNIAPDEVDRIAEGVPGVREAACYEIPDEEFGALVGLAVVASAELDES

AARALKHTIAARFRRESEPMARPSIIEIVTDIPRTQSGKVMRASLAAATTADKARVVVRG

>mcz:BN45_10115 K12422 long chain fatty acid CoA ligase FadD10 [EC:6.2.1.-] | (GenBank) fadD; Putative fatty-acid-CoA ligase FadD10 (fatty-acid-CoA synthetase) (fatty-acid-CoA synthase) (A)

MGGAKFQAMPQLPSTVLDRVFEQARQRPEAIALRRCDGTSALRYRELVAEVGGLAAALRA

QSVTRGSRVLVISDNGPETYLSVLACAKLGAIAVMADGNLPIAAIERFCQITDPAAALVA

PGSKMASSAVPEALHSIPVIAVDIAAVARESEHSLDAASLAGNADQGSEDPLAMIFTSGT

TGEPKAVLLANRTFFAVPDILQKEGLNWVTWVVGETTYSPLPATHIGGLWWILTCLMHGG

LCVTGGENTTSLLEILTTNAVATTCLVPTLLSKLVSELTSANATVPSLRLVGYGGSRAIA

ADVRFIEATGVRTAQVYGLSETGCTALCLPTDDGSIAKIEAGAVGRPYPGVDVYLAATDG

IGPTAPGAGPSASFGTLWIKSPANMLGYWNNPERTAEVLIDGWVNTGDLLERREDGFFYI

KGRSSEMIICGGVNIAPDEVDRIAEGVPGVREAACYEIPDEEFGALVGLAVVASAELDES

AARALKHTIAARFRRESEPMARPSTIVIVTDIPRTQSGKVMRASLAAATTADKARVVVRG

>mle:ML1994 K12422 long chain fatty acid CoA ligase FadD10 [EC:6.2.1.-] | (RefSeq) fadD10; acyl-CoA synthetase (A)

MSHLPPTVLERILKQAHERPEAIALRRSDGTSELRYGQLVAEVNGLAAHLSAQSVSQRSR

VLVISDNGPETYLSVLACAKLGAIAVMVDGNLPPATISRFSEICDPSAVLVARECRIDSS

SLPEILHSIPAITVNTTADATYSACSLDIDYLAGNLNYGTDDPLAMTFTSGTTGEPKAVL

LPNRTFFAIPDILREKGLAWIDWVVNETTYSPLSATHIGGLWWILNCLMHGGLCITGGEH

TSSLTEVLNANKVATTCLVPTLLSKLVYELKFGDVVTPPLRLIVYGGSRVIAADVRFIEA

AGVRTAQFYGLSETGCTALCLPTDNGSISKIEAGAVGRPYPGVEVYLADPNGGGPTVADT

ASSASFGTLWIKSPANMLGYWNNPERTREILVDGWVNTGDLVERREDGFFYIKGRSSEMI

VSGGVNIVPDEVDRIAESVSGVGEAACYEIPDAHFGALVGLAVIPSAELNESTAIELKRR

IAARYRRESESMARPSKIVIVADIPRTQSGKVIRATLAAALNGEQLGMVVRD

>mlb:MLBr01994 K12422 long chain fatty acid CoA ligase FadD10 [EC:6.2.1.-] | (GenBank) fadD10; acyl-CoA synthase (A)

MSHLPPTVLERILKQAHERPEAIALRRSDGTSELRYGQLVAEVNGLAAHLSAQSVSQRSR

VLVISDNGPETYLSVLACAKLGAIAVMVDGNLPPATISRFSEICDPSAVLVARECRIDSS

SLPEILHSIPAITVNTTADATYSACSLDIDYLAGNLNYGTDDPLAMTFTSGTTGEPKAVL

LPNRTFFAIPDILREKGLAWIDWVVNETTYSPLSATHIGGLWWILNCLMHGGLCITGGEH

TSSLTEVLNANKVATTCLVPTLLSKLVYELKFGDVVTPPLRLIVYGGSRVIAADVRFIEA

AGVRTAQFYGLSETGCTALCLPTDNGSISKIEAGAVGRPYPGVEVYLADPNGGGPTVADT

ASSASFGTLWIKSPANMLGYWNNPERTREILVDGWVNTGDLVERREDGFFYIKGRSSEMI

VSGGVNIVPDEVDRIAESVSGVGEAACYEIPDAHFGALVGLAVIPSAELNESTAIELKRR

IAARYRRESESMARPSKIVIVADIPRTQSGKVIRATLAAALNGEQLGMVVRD

>mpa:MAP_2747 no KO assigned | (GenBank) hypothetical protein (A)

MPAAETRETLAGIVERHAQRRPDAIAIRYGERQWSWAEWSSRIRRAAGALRGAGIQRGQC

VAFLDKNHPACLEVLIGGASVGAVTTVVNWRVIGDELVHVLADSGARVLVVGAELRPAAE

AAARRVPSLERIIEVGDEYESLLAAAEPAPSDAGVDTDETALVIYSSGTTGRPKGVLLSQ

RALVNHAANLAPAFPFGDGDANLVAMPLFHVGGIGYALFGIRAGAPTIMTREPDAAALIG

AVRAGATHAFFVPPVIARFLDAGEAARASIAGLRYIVYGAAPMPLPLLHRALSTWPGTKF

VQVYGQTELCGAVTALSDDDHRDAARPQLQLSAGKAVQGCEIRIVDPNSCAELPAGRSGE

VWVRSNQNMSGYLNRAEATAETITADGWVRTGDVGRLDADGYVYIEDRLKDMIITGGENV

YGPEVESVLIEHPAVVDAAVIGVPDDFWGESVKAIVVADGDVDAADVIEFCRRHLAGFKC

PRTVDFVAELPRNASGKILKTQLREPFWRDRDRRV

>mao:MAP4_1067 no KO assigned | (GenBank) fatty-acid--CoA ligase (A)

MPAAETRETLAGIVERHAQRRPDAIAIRYGERQWSWAEWSSRIRRAAGALRGAGIQRGQC

VAFLDKNHPACLEVLIGGASVGAVTTVVNWRVIGDELVHVLADSGARVLVVGAELRPAAE

AAARRVPSLERIIEVGDEYESLLAAAEPAPSDAGVDTDETALVIYSSGTTGRPKGVLLSQ

RALVNHAANLAPAFPFGDGDANLVAMPLFHVGGIGYALFGIRAGAPTIMTREPDAAALIG

AVRAGATHAFFVPPVIARFLDAGEAARASIAGLRYIVYGAAPMPLPLLHRALSTWPGTKF

VQVYGQTELCGAVTALSDDDHRDAARPQLQLSAGKAVQGCEIRIVDPNSCAELPAGRSGE

VWVRSNQNMSGYLNRAEATAETITADGWVRTGDVGRLDADGYVYIEDRLKDMIITGGENV

YGPEVESVLIEHPAVVDAAVIGVPDDFWGESVKAIVVADGDVDAADVIEFCRRHLAGFKC

PRTVDFVAELPRNASGKILKTQLREPFWRDRDRRV

>mavi:RC58_00810 K00666 fatty-acyl-CoA synthase [EC:6.2.1.-] | (GenBank) long-chain fatty acid--CoA ligase (A)

MTAQLAHHPTQANEQPYLSRRQNWVNQLERHALMQPNATALRFLGKGLTWGELHGRVRAL

ADALSRRGVGFGDRVMVLMLNRPEFMESVLAINMLGAIAVPLNFRLTAAEIAFLVQDCQA

RVVITEAVLAPVATGVRDIESLLDTVVVAGGSSDDTVLGYEDLIDETGAAHQPVDIPNDA

AALIMYTSGTTGRPKGAVLTHTNLTGQTMTGLYTNGADINNDVGFIGVPFFHIAGIGNML

TGLLLGIPTVIYPLGAFEPGQLLDVLAAEKVTGIFLVPAQWQAVCAEQRARPRDLKLRVI

SWGAAPAPDALLREMSAMFPGTQILAAFGQTEMSPVTCMLLGEDAIRKRGSVGKVIPTVA

ARVVDENMNDVPVGEVGEIVYRAPTLMSGYWNNPEATAEAFAGGWFHSGDLVRMDEDGYV

WVVDRKKDMIISGGENIYCAEVENVLASHPDIVEVAVIGRAHEKWGEVPIAVAAVANDNL

ALEDLDEFLTERLARYKHPKALEIVDALPRNPAGKVLKTELRIRYGGG

>mavu:RE97_05295 no KO assigned | (GenBank) long-chain fatty acid--CoA ligase (A)

MPAAETRETLAGIVERHAQRRPDAIAIRYGERQWSWAEWSSRIRRAAGALRGAGIQRGQC

VAFLDKNHPACLEVLIGGASVGAVTTVVNWRVIGDELVHVLADSGARVLVVGAELRPAAE

AAARRVPSLERIIEVGDEYESLLAAAEPAPSDAGVDTDETALVIYSSGTTGRPKGVLLSQ

RALVNHAANLAPAFPFGDGDANLVAMPLFHVGGIGYALFGIRAGAPTIMTREPDAAALIG

AVRAGATHAFFVPPVIARFLDAGEAARASIAGLRYIVYGAAPMPLPLLHRALSTWPGTKF

VQVYGQTELCGAVTALSDDDHRDAARPQLQLSAGKAVQGCEIRIVDPNSCAELPAGRSGE

VWVRSNQNMSGYLNRAEATAETITADGWVRTGDVGRLDADGYVYIEDRLKDMIITGGENV

YGPEVESVLIEHPAVVDAAVIGVPDDFWGESVKAIVVADGDVDAADVIEFCRRHLAGFKC

PRTVDFVAELPRNASGKILKTQLREPFWRDRDRRV

>mav:MAV_3523 no KO assigned | (GenBank) acyl-CoA synthase (A)

MPAAETRETLAGIVERHAQRRPDAIAIRYGERQWSWAEWSSRIRRAAGALRGDGIERGQC

VAFLDKNHPACLEVLIGGASVGAVTTVVNWRVIGDELVHVLADSGARVLVVGAELRPAAE

AAARRVPSLERIIEVGDEYESLLAAAEPAPSDAGVDTDETALVIYSSGTTGRPKGVLLSQ

RALVNHAANLAPAFPFGDGDANLVAMPLFHVGGIGYALFGIRAGAPTIMTREPDAAALIG

AVRAGATHAFFVPPVIARFLDAGEAARASIAGLRYIVYGAAPMPLPLLHRALSTWPGTKF

VQVYGQTELCGAVTALSDDDHRDAARPQLQLSAGKAVQGCEIRIVDPNNCAELPAGRSGE

VWVRSNQNMSGYLNRAEATAETITADGWVRTGDVGRLDADGYVYIEDRLKDMIITGGENV

YGPEVESVLIEHPAVVDAAVIGVPDDFWGESVKAIVVADGDVDAADVIEFCRRHLAGFKC

PRTVDFVAELPRNASGKILKTQLREPFWRDRDRRV

>mavr:LA63_16135 no KO assigned | (GenBank) long-chain fatty acid--CoA ligase (A)

MPAAETRETLAGIVERHAQRRPDAIAIRYGERQWSWAEWSSRIRRAAGALRGDGIERGQC

VAFLDKNHPACLEVLIGGASVGAVTTVVNWRVIGDELVHVLADSGARVLVVGAELRPAAE

AAARRVPSLERIIEVGDEYESLLAAAEPAPSDAGVDTDETALVIYSSGTTGRPKGVLLSQ

RALVNHAANLAPAFPFGDGDANLVAMPLFHVGGIGYALFGIRAGAPTIMTREPDAAALIG

AVRAGATHAFFVPPVIARFLDAGEAARASIAGLRYIVYGAAPMPLPLLHRALSTWPGTKF

VQVYGQTELCGAVTALSDDDHRDAARPQLQLSAGKAVQGCEIRIVDPNNCAELPAGRSGE

VWVRSNQNMSGYLNRAEATAETITADGWVRTGDVGRLDADGYVYIEDRLKDMIITGGENV

YGPEVESVLIEHPAVVDAAVIGVPDDFWGESVKAIVVADGDVDAADVIEFCRRHLAGFKC

PRTVDFVAELPRNASGKILKTQLREPFWRDRDRRV

>mavd:NF84_15990 no KO assigned | (GenBank) long-chain fatty acid--CoA ligase (A)

MPAAETRETLAGIVERHAQRRPDAIAIRYGERQWSWAEWSSRIRRAAGALRGDGIERGQC

VAFLDKNHPACLEVLIGGASVGAVTTVVNWRVIGDELVHVLADSGARVLVVGAELRPAAE

AAARRVPSLERIIEVGDEYESLLAAAEPAPSDAGVDTDETALVIYSSGTTGRPKGVLLSQ

RALVNHAANLAPAFPFGDGDANLVAMPLFHVGGIGYALFGIRAGAPTIMTREPDAAALIG

AVRAGATHAFFVPPVIARFLDAGEAARASIAGLRYIVYGAAPMPLPLLHRALSTWPGTKF

VQVYGQTELCGAVTALSDDDHRDAARPQLQLSAGKAVQGCEIRIVDPNNCAELPAGRSGE

VWVRSNQNMSGYLNRAEATAETITADGWVRTGDVGRLDADGYVYIEDRLKDMIITGGENV

YGPEVESVLIEHPAVVDAAVIGVPDDFWGESVKAIVVADGDVDAADVIEFCRRHLAGFKC

PRTVDFVAELPRNASGKILKTQLREPFWRDRDRRV

>mava:LA64_16140 no KO assigned | (GenBank) long-chain fatty acid--CoA ligase (A)

MPAAETRETLAGIVERHAQRRPDAIAIRYGERQWSWAEWSSRIRRAAGALRGDGIERGQC

VAFLDKNHPACLEVLIGGASVGAVTTVVNWRVIGDELVHVLADSGARVLVVGAELRPAAE

AAARRVPSLERIIEVGDEYESLLAAAEPAPSDAGVDTDETALVIYSSGTTGRPKGVLLSQ

RALVNHAANLAPAFPFGDGDANLVAMPLFHVGGIGYALFGIRAGAPTIMTREPDAAALIG

AVRAGATHAFFVPPVIARFLDAGEAARASIAGLRYIVYGAAPMPLPLLHRALSTWPGTKF

VQVYGQTELCGAVTALSDDDHRDAARPQLQLSAGKAVQGCEIRIVDPNNCAELPAGRSGE

VWVRSNQNMSGYLNRAEATAETITADGWVRTGDVGRLDADGYVYIEDRLKDMIITGGENV

YGPEVESVLIEHPAVVDAAVIGVPDDFWGESVKAIVVADGDVDAADVIEFCRRHLAGFKC

PRTVDFVAELPRNASGKILKTQLREPFWRDRDRRV

>mit:OCO_13920 no KO assigned | (GenBank) acyl-CoA synthase (A)

MLIGDIATNNSRRYPDKRALVDADRALTWSQVDDRARRLAAFLIGRGLVPGDRVMVIARN

CIEWPEISFGLAKAGLITVPVNIRLAPDEVAHVRDDSGARAVIIHADHLDKFLGELTELA

LILGVGAHSMLGTSELVTDYETALAQAQPGAERRDITPDDVAFILYTSGTTGRAKGVMHT

HRALLYQAADTNLVTEANRSDVMLATTPFFTAGGMVRTVSWLYLGQTMVIHQRFDPQAVI

DEIERSAITFTTFIPTMLHRTLAILEDGPPRDMSSLRRISYGSAPVPPGLARKAMDLLGC

ELQQRYGLTECGGQATILTPQDHRDIVAGKTSIATSCGQETPMCVIRVIDVDGNDAPPGD

VGEIVITSPANAIGYWNRPEQTAETFRSDGLRSGDLGYLDEEGYLHITGRKTDLIISGGF

NVYPAEIERVIAQHSDVDMVAVVGVPDPEWGETPVAAVIAKSHVGDQDALTAELAALCRA

ELAGYKQPRRFVFWREFPLGPAGKILKREIANQVNHVSQSGGKMPVSTGSTEERR

>mir:OCQ_50360 K00666 fatty-acyl-CoA synthase [EC:6.2.1.-] | (GenBank) acyl-CoA synthetase (A)

MSSLTAQLADHLTEQPYLARRQNWVNQLERHALMQPNATALRFLGKTLTWGELRGRVAAL

ADALSRRGVGFGDRVMILMLNRTEFVESVLAINMLGAIAVPLNFRLTAAEIAFLVQDCEA

RVIITESVLAPVATGVRDIESMLGTIVVAGGSTDDTVLGYEDLVNETGESHQPVDIPNDS

PALIMYTSGTTGRPKGAVLTHTNLTGQTMTGLYTNGADINSDVGFIGVPFFHIAGIGNLL

TGVLLGIPTVIYPLGAFEPGQLLDVLAAEKVTGIFLVPAQWQAVCAEQQARPRDLKLRVI

SWGAAPAPDALLREMSARFPGTQILAAFGQTEMSPVTCMLLGEDAIRKRGSVGKVIPTVA

ARVVDENMNDVPIGEVGEIVYRAPTLMSGYWNNPEATAEAFAGGWFHSGDLVRMDSDGYV

WVVDRKKDMIISGGENIYCAEVENVLASHPSIVEVAVIGRAHDKWGEVPIAVAAVAGEGL

ALDELDEFLTERLARYKHPKALEIVDALPRNPAGKVLKTELRIRYGAAKID

>mia:OCU_14390 no KO assigned | (GenBank) acyl-CoA synthase (A)

MLIGDIATNNSRRYPDKRALVDADRALTWSQVDDRARRLAAFLIGRGLVPGDRVMVIARN

CIEWPEISFGLAKAGLITVPVNIRLAPDEVAHVRDDSGARAVIIHADHLDKFLGELTELA

LILGVGAHSMLGTSELVTDYETALAQAQPGAERRDITPDDVAFILYTSGTTGRAKGVMHT

HRALLYQAADTNLVTEANRSDVMLATTPFFTAGGMVRTVSWLYLGQTMVIHQRFDPQAVI

DEIERSAITFTTFIPTMLHRTLAILEDGPPRDMSSLRRISYGSAPVPPGLARKAMDLLGC

ELQQRYGLTECGGQATILTPQDHRDIVAGKTSIATSCGQETPMCVIRVIDVDGNDAPPGD

VGEIVITSPANAIGYWNRPEQTAETFRSDGLRSGDLGYLDEEGYLHITGRKTDLIISGGF

NVYPAEIERVIAQHSDVDMVAVVGVPDPEWGETPVAAVIAKSHVGDQDALTAELAALCRA

ELAGYKQPRRFVFWREFPLGPAGKILKREIANQVNHVSQSGGKMPVSTGSTEERR

>mie:LG41_06935 no KO assigned | (GenBank) acyl-CoA synthetase (A)

MLIGDIATNNSRRYPDKRALVDADRALTWSQVDDRARRLAAFLIGRGLVPGDRVMVIARN

CIEWPEISFGLAKAGLITVPVNIRLAPDEVAHVRDDSGARAVIIHADHLDKFLGELTELA

LILGVGAHSMLGTSELVTDYETALAQAQPGAERRDITPDDVAFILYTSGTTGRAKGVMHT

HRALLYQAADTNLVTEANRSDVMLATTPFFTAGGMVRTVSWLYLGQTMVIHQRFDPQAVI

DEIERSAITFTTFIPTMLHRTLAILEDGPPRDMSSLRRISYGSAPVPPGLARKAMDLLGC

ELQQRYGLTECGGQATILTPQDHRDIVAGKTSIATSCGQETPMCVIRVIDVDGNDAPPGD

VGEIVITSPANAIGYWNRPEQTAETFRSDGLRSGDLGYLDEEGYLHITGRKTDLIISGGF

NVYPAEIERVIAQHSDVDMVAVVGVPDPEWGETPVAAVIAKSHVGDQDALTAELAALCRA

ELAGYKQPRRFVFWREFPLGPAGKILKREIANQVNHVSQSGGKMPVSTGSTEERR

>mid:MIP_00267 K00666 fatty-acyl-CoA synthase [EC:6.2.1.-] | (GenBank) Long-chain-fatty-acid--CoA ligase (A)

MMTMPSRTFRFPVSDWVAHHASVRPHAIALASADTGERVSWAQLEQQVGLAAAALLAHGL

RPGDRIAVVADNAPRAFVLQFAAMRAGVVMVPLNWRLVTAEMRHQCIDAAVSALTHDATW

AEPARDVADAAGVQTILELETLTSAEPGNGLVPLPPQPHDPDDITHILYTSGTTGTPKGA

LVSHASMMWNAFNILTAAQVAAPDVNMLNPMPLFHAGGLNVLANPILMHGGQVTTMARWN

PAAILAYIGDQTNGVTHLTTAPSLLQTLVEDPTFGTTDFGTMRKIVLGGGTTTPHLLRAF

AAKGVALHPQYGGTETGPAALVLEEGLERALSGTCGKPVLHTAVRLVDPDTLTDVADDVV

GEVWLKGPAVTPGYWNLPNEQYFVDGWFRTGDAARRDADGYFYIAGRYKDMYKSGGENVY

AAEVENVLIDLPEVAEVAIIGVPDPKWGEVGLAVVVASPGADVTLDALHSACAGRIARYK

YPKHLDVVAALPRNATGKVAKMALREHYHSAVPLRTDHESLVAHQKESHDVSRR

>myo:OEM_33770 no KO assigned | (GenBank) hypothetical protein (A)

MPQTDTAETLAGIVERHALQRPEAIAIRYGERQWSWAEWSSRIRRAAGALRDAGIERGQC

VAFLDKNHPACLEVLIAGASVGVVTTVVNWRVIGDELVHVLADSGARVLLVGAELRAAAE

AAAERVPGLERIIEVGEEYESLLAAASPAEADAGLDPNETALVIYSSGTTGRPKGVLLSQ

RALVNHAANLAPAFPFADGDANLVAMPLFHVGGIGYALFGIRSGAPTFLTREPDAATLIG

AVRAGATHAFFVPPVIARFLQAGEAATAAIAGLRYIVYGAAPMPLPLLHRALSTWPDTKF

VQVYGQTELCGAVTALSDADHRDSARPELQLSAGKAVLGCEIRIVDPETGASLPPGESGE

VWVRSNQNMTGYLNRPEATAETITADDWVRTGDVGRLDADDYVYIEDRLKDMIITGGENV

YGPEVESVLIEHPAVADAAVIGVPDDFWGESVKAIVVADGEADPAGIIEFCRQHLAGFKC

PRTVDFVSELPRNASGKILKTQLREPFWRDRSRGI

>msm:MSMEG_3694 no KO assigned | (RefSeq) feruloyl-CoA synthetase (A)

MSLLHQLISSVAAAAPHRPAIIADDGSTIGYAEFDHQIRAVARWIASRTEPGDRVAVIAD

NSPAYAVLYYAVPLGGRVLTLINQRLSPAEQAAQLETTRPALVLGDTVYLDALPQIDSVA

FGSPAWRDAVSTTAAAPEVDHAEAQPGDPAWLLFTSGSTGVPKGVVHSHRSILAAVHGTV

DGRSVTPAGVYLLPFPMCHIAGYNMLVQHAVGATVVLMSRFRPESFAAQVRAHGVRSCSL

APTMLHALLAHVESTGTTLPTLEAVAYGSAAMPLDLLRRAIEALGVEFHQGYGMTETGGN

VTFLGPDDHRAGAAGHPEVLVSAGHPHSGVEIGIVDADGAPLPPGGIGEIVVRGAQVARG

YWPDRPSTVDGWLHTGDIGRLDHTGRLFVVDRLKDIIVTGGENVSSREVEDVLSGHPDVD

MVAVVGVPDGYWGEAVCAVVVPVPGRNPRADDLIDHVRSAIAAFKRPREVLFVDELPLTG

NGKIAKDRVRDIARTALTA

>msg:MSMEI_3608 no KO assigned | (GenBank) AMP-dependent synthetase and ligase (A)

MSLLHQLISSVAAAAPHRPAIIADDGSTIGYAEFDHQIRAVARWIASRTEPGDRVAVIAD

NSPAYAVLYYAVPLGGRVLTLINQRLSPAEQAAQLETTRPALVLGDTVYLDALPQIDSVA

FGSPAWRDAVSTTAAAPEVDHAEAQPGDPAWLLFTSGSTGVPKGVVHSHRSILAAVHGTV

DGRSVTPAGVYLLPFPMCHIAGYNMLVQHAVGATVVLMSRFRPESFAAQVRAHGVRSCSL

APTMLHALLAHVESTGTTLPTLEAVAYGSAAMPLDLLRRAIEALGVEFHQGYGMTETGGN

VTFLGPDDHRAGAAGHPEVLVSAGHPHSGVEIGIVDADGAPLPPGGIGEIVVRGAQVARG

YWPDRPSTVDGWLHTGDIGRLDHTGRLFVVDRLKDIIVTGGENVSSREVEDVLSGHPDVD

MVAVVGVPDGYWGEAVCAVVVPVPGRNPRADDLIDHVRSAIAAFKRPREVLFVDELPLTG

NGKIAKDRVRDIARTALTA

>msb:LJ00_18365 no KO assigned | (GenBank) AMP-dependent synthetase (A)

MSLLHQLISSVAAAAPHRPAIIADDGSTIGYAEFDHQIRAVARWIASRTEPGDRVAVIAD

NSPAYAVLYYAVPLGGRVLTLINQRLSPAEQAAQLETTRPALVLGDTVYLDALPQIDSVA

FGSPAWRDAVSTTAAAPEVDHAEAQPGDPAWLLFTSGSTGVPKGVVHSHRSILAAVHGTV

DGRSVTPAGVYLLPFPMCHIAGYNMLVQHAVGATVVLMSRFRPESFAAQVRAHGVRSCSL

APTMLHALLAHVESTGTTLPTLEAVAYGSAAMPLDLLRRAIEALGVEFHQGYGMTETGGN

VTFLGPDDHRAGAAGHPEVLVSAGHPHSGVEIGIVDADGAPLPPGGIGEIVVRGAQVARG

YWPDRPSTVDGWLHTGDIGRLDHTGRLFVVDRLKDIIVTGGENVSSREVEDVLSGHPDVD

MVAVVGVPDGYWGEAVCAVVVPVPGRNPRADDLIDHVRSAIAAFKRPREVLFVDELPLTG

NGKIAKDRVRDIARTALTA

>msn:LI99_18370 no KO assigned | (GenBank) AMP-dependent synthetase (A)

MSLLHQLISSVAAAAPHRPAIIADDGSTIGYAEFDHQIRAVARWIASRTEPGDRVAVIAD

NSPAYAVLYYAVPLGGRVLTLINQRLSPAEQAAQLETTRPALVLGDTVYLDALPQIDSVA

FGSPAWRDAVSTTAAAPEVDHAEAQPGDPAWLLFTSGSTGVPKGVVHSHRSILAAVHGTV

DGRSVTPAGVYLLPFPMCHIAGYNMLVQHAVGATVVLMSRFRPESFAAQVRAHGVRSCSL

APTMLHALLAHVESTGTTLPTLEAVAYGSAAMPLDLLRRAIEALGVEFHQGYGMTETGGN

VTFLGPDDHRAGAAGHPEVLVSAGHPHSGVEIGIVDADGAPLPPGGIGEIVVRGAQVARG

YWPDRPSTVDGWLHTGDIGRLDHTGRLFVVDRLKDIIVTGGENVSSREVEDVLSGHPDVD

MVAVVGVPDGYWGEAVCAVVVPVPGRNPRADDLIDHVRSAIAAFKRPREVLFVDELPLTG

NGKIAKDRVRDIARTALTA

>msh:LI98_18375 no KO assigned | (GenBank) AMP-dependent synthetase (A)

MSLLHQLISSVAAAAPHRPAIIADDGSTIGYAEFDHQIRAVARWIASRTEPGDRVAVIAD

NSPAYAVLYYAVPLGGRVLTLINQRLSPAEQAAQLETTRPALVLGDTVYLDALPQIDSVA

FGSPAWRDAVSTTAAAPEVDHAEAQPGDPAWLLFTSGSTGVPKGVVHSHRSILAAVHGTV

DGRSVTPAGVYLLPFPMCHIAGYNMLVQHAVGATVVLMSRFRPESFAAQVRAHGVRSCSL

APTMLHALLAHVESTGTTLPTLEAVAYGSAAMPLDLLRRAIEALGVEFHQGYGMTETGGN

VTFLGPDDHRAGAAGHPEVLVSAGHPHSGVEIGIVDADGAPLPPGGIGEIVVRGAQVARG

YWPDRPSTVDGWLHTGDIGRLDHTGRLFVVDRLKDIIVTGGENVSSREVEDVLSGHPDVD

MVAVVGVPDGYWGEAVCAVVVPVPGRNPRADDLIDHVRSAIAAFKRPREVLFVDELPLTG

NGKIAKDRVRDIARTALTA

>msa:Mycsm_00659 no KO assigned | (GenBank) acyl-CoA synthetase (AMP-forming)/AMP-acid ligase II (A)

MPHPDIATIADIVRVHAAERPDAVALVVGDSTITFAELDARSSQAAQAFSAAGVGYGDRV

AFIEKNGAEFFEVVCGLAKLGAVAVPVNWRLAAPEMLHIVEDAQAKVVVAGSQFFDHVEA

IEDRLTASIVAVGDHARWPDFDTWVSSQPAEDPGVTTESDDVAFLMYTSGTTGPPKGVML

SNNNYFSKATGIADKWRFDSESVSLAVMPMFHMAGSGWALVGLAEGCTTVVLRDVDPPVI

LDAIARHRITNMLLVPAVIQMLLATPSDTDFSSVRAIVYGASPITDDVLVKGLKRFGCEF

LQVYGLTETTGSITQLDDHDPLLRPGLLRSCGKPYPWVEVRIVDKTGVGVPEGTVGELWT

RSSQNMLGYWNNPDATAATVTPDGWLKTGDAGYRDAEGYIYLHDRVKDMIVSGGENISPA

EVENVLMAHPAVGDVAVIGVPDEKWGEAVKAIVVPAADTKPSEAELIAYARHRLAGFKLP

KSVGFASALPRTPSGKLLKRTLREPYWEGVGRRIG

>mul:MUL_4856 K12422 long chain fatty acid CoA ligase FadD10 [EC:6.2.1.-] | (GenBank) fadD10; fatty-acid-CoA ligase FadD10 (A)

MSDLPATVLERIIEQAQRRPEAIALRRCDGTSALPYGELVAEVDRYAGALRAQSASRGSR

VLVISDNGPETYLAVLACAKLGAIAVMADGNLPPATIDRFCQITDPVAVLIAPGSKVGSS

SLPEGLAAIPAIRVDIGSGTGEFAHSPDTDRPATEPGLGADDPLAMIFTSGTTGEPKAVL

LANRTFFAVPDILRNEGLNWVTWVDGETTYSPLPATHIGGLWWILTCLMRGGLCITGGEN

TLSLMQILNSNAVNTTCLVPTLLSKLVSELKSAATTVPSLRLLGYGGSRAIAADVRFIEA

TGVRTAQVYGLSETGCTALCLPTDDGSIAKIEAGAVGRPYPGVEVYLAADDEADGAGPNA

PGAGPSASFGTLWIKSPANMLGYWSNPQRTQEVLIDGWVNTGDLLERHEDGFFYIKGRSS

EMIISGGVNIAPDEVDRIAEGVPGVREAACFEIPDPEFGALVGLAVVAATDVDASAARKL

KHTIAAHYRRESESVARPSTIVIVSEIPRTQSGKVMRTSLATAANQVQTGG

>mva:Mvan_0943 K01897 long-chain acyl-CoA synthetase [EC:6.2.1.3] | (GenBank) AMP-dependent synthetase and ligase (A)

MIHNLADIIRVHGRERPGAPALVVGDRTVTYGELDDRSSRAAQAFAQAGVGVGDRVAFVD

KNGAEFFEVTFGLAKVGAVGVPVNWRLAAPEMRHIIADSGAKIVVVGQDFAGHLEAIEDG

LDADIVVIGDHPRWPAFDDWVASHPPVDPGVVTGPDDVVLLMYTSGTTGAPKGVMLSNTN

YVYKTGGVAGPWQFDADAVSLAVMPLFHMAGSGWALAGLWQGATTVVLRDVEPAAILDAI

ARHRITNMLLVPAVIQFLLDTDGVAEVDLSTLRVIVYGASPISDDVLVRGIERFGPIFAQ

VYGMTETTGSITQLDGPDHVPALLRSCGRPYPWVQIRIVDETGADAVAGTVGEVWTRSEQ

NMLGYWNNPDATASTLTADGWLKTGDAGYVDDDGYLFLHDRIKDMIVSGGENVYPVEVEN

VLMTHPAVADAAVIGVPDRRWGEAVKAVVVAARGAQLTEAELIAFARDRIGGFKLPKSVD

FVDVLPRNPSGKLLKRALREPYWDGADRHIG

>mgi:Mflv_0027 K01897 long-chain acyl-CoA synthetase [EC:6.2.1.3] | (GenBank) AMP-dependent synthetase and ligase (A)

MFHNLADIVRSHGRNRPDAPALIVGDRTVSYRELDERSNRTAQAFSRTGVGPGDRVAFVE

RNGIEFFDVAFGSAKLGAVAVPVNWRLTAPEMRHVIADCGASVVVVGQEFTGRIEAIADD

LDVEVVVIGKHRHWPTFDDLLAAAPAVDPGVVTGPDDLVFLMYTSGTTGAPKGVMLSNAN

VVCKTAGVGGPWKFDADSVSLAVMPLFHMAGFGWALAGLWEGAVTVVLRDVDPGAILDAV

ARHRVTNMLLVPAVIQFLLDTTGLDDTDLSRLRIVVYGASPITDDVLMRGIDRFGGIFAQ

VYGMTESTGSVTQLDGDEHLPQLLRSCGRPYPWVQIRVVDPTGRDVAPGTVGEVWTRSDQ

NMLGYWNNPEATAATLTPDGWLRTGDAGYLDRDGYLYLHDRIKDMIVSGAENVYPAEVEN

VLMTHPAVTDVAVIGIPDTRWGEAVKAVVVAGAPVTEAELIAFAREQLAGFKLPKSVDFV

DALPRNPSGKLLKRQLREPYWTDEDRHIG

>msp:Mspyr1_07630 no KO assigned | (GenBank) acyl-CoA synthetase (AMP-forming)/AMP-acid ligase II (A)

MFHNLADIVRSHGRNRPDAPALIVGDRTVSYRELDERSNRTAQAFSRTGVGPGDRVAFVE

RNGIEFFDVAFGSAKLGAVAVPVNWRLTAPEMRHVIADCGASVVVVGQEFTDHLDVIADE

LDVEVVVIGNHSRWPDFEGWLAAQPSVDPGVVTGPDDLVFLMYTSGTTGAPKGVMLSNAN

VVCKTAGVGGPWKFDADSVSLAVMPLFHMAGFGWALAGLWEGAVTVVLRDVDPGAILDAV

ARHRVTNMLLVPAVIQFLLDTPGLDDTDLSRLRIVVYGASPITDDVLMRGIDRFGGIFAQ

VYGMTESTGSVTQLDGDEHLPQLLRSCGRPYPWVQIRVVDPTGRDVAPGTVGEVWTRSDQ

NMLGYWNNPEATAATLTPDGWLRTGDAGYLDRDGYLYLHDRIKDMIVSGAENVYPAEVEN

VLMTHPAVTDVAVIGIPDTRWGEAVKAVVVAGAPVTEAELIAFAREQLAGFKLPKSVDFV

DALPRNPSGKLLKRQLREPYWTDEDRHIG

>mab:MAB_0661 K12422 long chain fatty acid CoA ligase FadD10 [EC:6.2.1.-] | (RefSeq) Putative long chain fatty acid-coA ligase (A)

MSYGELIGAVEDLAATLEAGSVTHGSRVVVLSDNGPQTYLSVLACARLGAIAVMVDDSLP

AATVARFSEITRPAAIIPNEGGGVGAENGERAYPKAQPDSGADEALAMIFTSGTTGEPKA

VLLPNRTFFAIPDILRAEGLRWIDWVAGETTYSPLPATHIGGLWWILNGLMHGASCITGG

EQGASLRELLVDNEVATACLVPTLLTRLVSELKLTGTDVPALRFIAYGGSRAIAADVRFI

EAAGVRTAQVYGLSETGCTALCLPTDSESISRIEQGAVGRPYPGVQTYLSPEGGGDPTTG

AESASYGTLWIKSPANMLGYWGSPERTGEVLAEGWVNTGDLVERRADGFFYIRGRSSEMI

ISGGVNVVPDEVDRIAEDIAGVREAACYEIPDEEFGALVGLAVVATTELDGAGTAALKQR

IAARYRRESESMARPSTIVLVEDIPRTKSGKVMRSALSASLNGARARV

>mabb:MASS_0632 K12422 long chain fatty acid CoA ligase FadD10 [EC:6.2.1.-] | (GenBank) acyl-CoA synthetase (A)

MSHSPPPTVLERIVAQARTRPDSVALRRCDGRDAMSYGELIGAVEDLAAALEADSVTHGS

RVVVLSDNGPQTYLSVLACARLGAIAVMVDDSLPAATISRFCEIARPAAIIPNKGGGVGA

ENGEIAYPKAQPDSSANEALAMIFTSGTTGEPKAVLLPNRTFFAIPDILRTEGLRWIDWV

AGETTYSPLPATHIGGLWWILNGLMHGASCITGGEQGASLRELLVDNEVATACLVPTLLT

RLVSELKLTGTDVPALRFIAYGGSRAIAADVRFIEAAGVRTAQVYGLSETGCTALCLPTD

SESISRIEQGAVGRPYPGVQTYLSPEGGGGPTTGAESASYGTLWIKSPANMLEYWGSPER

TGEVLAEGWVNTGDLVERRADGFFYIRGRSSEMIISGGVNVVPDEVDRIAEDIAGVREAA

CYEIPDEEFGALVGLAVVPATELDGPGTAALKQRIAATYRRESESMARPSTIVLVEDIPR

TTSGKVMRSALSASLNGARARV

>mmv:MYCMA_0348 K12422 long chain fatty acid CoA ligase FadD10 [EC:6.2.1.-] | (GenBank) acyl-CoA synthetase (A)

MSHSPPPTVLERIVAQARTRPDSIALRRCDGRDAMSYGELIGAVEDLAAALEADSVTHGS

RVVVLSDNGPQTYLSVLACARLGAIAVMVDDSLPAATISRFCEIARPAAIIPNKGGGVGA

ENGEIAYPKAQPDSGANEALVMIFTSGTTGEPKAVLLPNRTFFAIPDILRTEGLRWIDWV

AGETTYSPLPATHIGGLWWILNGLMHGASCITGGEQGASLRELLVDNEVATACLVPTLLT

RLVSELKLTGTDVPALRFIAYGGSRAIAADVRFIEAAGVRTAQVYGLSETGCTALCLPTD

SESISRIEQGAVGRPYPGVQTYLSPEGGGGPTTGAESASYGTLWIKSPANMLEYWGSPER

TGEVLAEGWVNTGDLVERRADGFFYIRGRSSEMIISGGVNVVPDEVDRIAEDIAGVREAA

CYEIPDEEFGALVGLAVVPATELDGPGTAALKQRIAATYRRESESMARPSTIVLVEDIPR

TTSGKVMRSALSASLNGARARV

>may:LA62_03335 K12422 long chain fatty acid CoA ligase FadD10 [EC:6.2.1.-] | (GenBank) acyl-CoA synthetase (A)

MSHSPPPTVLERIVAQARTRPDSIALRRCDGRDAMSYGELIGAVEDLAATLEAGSVTHGS

RVVVLSDNGPQTYLSVLACARLGAIAVMVDDSLPAATVARFSEITRPAAIIPNEGGGVGA

ENGERAYPKAQPDSGADEALAMIFTSGTTGEPKAVLLPNRTFFAIPDILRAEGLRWIDWV

AGETTYSPLPATHIGGLWWILNGLMHGASCITGGEQGASLRELLVDNEVATACLVPTLLT

RLVSELKLTGTDVPALRFIAYGGSRAIAADVRFIEAAGVRTAQVYGLSETGCTALCLPTD

SESISRIEQGAVGRPYPGVQTYLSPEGGGDPTTGAESASYGTLWIKSPANMLGYWGSPER

TGEVLAEGWVNTGDLVERRADGFFYIRGRSSEMIISGGVNVVPDEVDRIAEDIAGVREAA

CYEIPDEEFGALVGLAVVATTELDGAGTAALKQRIAARYRRESESMARPSTIVLVEDIPR

TKSGKVMRSALSASLNGARARV

>mabo:NF82_03330 K12422 long chain fatty acid CoA ligase FadD10 [EC:6.2.1.-] | (GenBank) acyl-CoA synthetase (A)

MSHSPPPTVLERIVAQARTRPDSIALRRCDGRDAMSYGELIGAVEDLAATLEAGSVTHGS

RVVVLSDNGPQTYLSVLACARLGAIAVMVDDSLPAATVARFSEITRPAAIIPNEGGGVGA

ENGERAYPKAQPDSGADEALAMIFTSGTTGEPKAVLLPNRTFFAIPDILRAEGLRWIDWV

AGETTYSPLPATHIGGLWWILNGLMHGASCITGGEQGASLRELLVDNEVATACLVPTLLT

RLVSELKLTGTDVPALRFIAYGGSRAIAADVRFIEAAGVRTAQVYGLSETGCTALCLPTD

SESISRIEQGAVGRPYPGVQTYLSPEGGGDPTTGAESASYGTLWIKSPANMLGYWGSPER

TGEVLAEGWVNTGDLVERRADGFFYIRGRSSEMIISGGVNVVPDEVDRIAEDIAGVREAA

CYEIPDEEFGALVGLAVVATTELDGAGTAALKQRIAARYRRESESMARPSTIVLVEDIPR

TKSGKVMRSALSASLNGARARV

>mabl:MMASJCM_0651 K12422 long chain fatty acid CoA ligase FadD10 [EC:6.2.1.-] | (GenBank) long-chain-fatty-acid--CoA ligase (A)

MSHSPPPTVLERIVAQARTRPDSIALRRCDGRDAMSYGELIGAVEDLAAALEADSVTHGS

RVVVLSDNGPQTYLSVLACARLGAIAVMVDDSLPAATISRFCEIARPAAIIPNKGGGVGA

ENGEIAYPKAQPDSSANEALAMIFTSGTTGEPKAVLLPNRTFFAIPDILRTEGLRWIDWV

AGETTYSPLPATHIGGLWWILNGLMHGASCITGGEQGASLRELLVDNEVATACLVPTLLT

RLVSELKLTGTDVPALRFIAYGGSRAIAADVRFIEAAGVRTAQVYGLSETGCTALCLPTD

SESISRIEQGAVGRPYPGVQTYLSPEGGGGPTTGAESASYGTLWIKSPANMLEYWGSPER

TGEVLAEGWVNTGDLVERRADGFFYIRGRSSEMIISGGVNVVPDEVDRIAEDIAGVREAA

CYEIPDEEFGALVGLAVVPATELDGPGTAALKQRIAATYRRESESMARPSTIVLVEDIPR

TTSGKVMRSALSASLNGARARV

>maz:LA61_03250 K12422 long chain fatty acid CoA ligase FadD10 [EC:6.2.1.-] | (GenBank) acyl-CoA synthetase (A)

MSHSPPPTVLERIVAQARTRPDSIALRRCDGRDAMSYGELIGAVEDLAATLEAGSVTHGS

RVVVLSDNGPQTYLSVLACARLGAIAVMVDDSLPAATVARFSEITRPAAIIPNEGGGVGA

ENGERAYPKAQPDSGADEALAMIFTSGTTGEPKAVLLPNRTFFAIPDILRAEGLRWIDWV

AGETTYSPLPATHIGGLWWILNGLMHGASCITGGEQGASLRELLVDNEVATACLVPTLLT

RLVSELKLTGTDVPALRFIAYGGSRAIAADVRFIEAAGVRTAQVYGLSETGCTALCLPTD

SESISRIEQGAVGRPYPGVQTYLSPEGGGDPTTGAESASYGTLWIKSPANMLGYWGSPER

TGEVLAEGWVNTGDLVERRADGFFYIRGRSSEMIISGGVNVVPDEVDRIAEDIAGVREAA

CYEIPDEEFGALVGLAVVATTELDGAGTAALKQRIAARYRRESESMARPSTIVLVEDIPR

TKSGKVMRSALSASLNGARARV

>mak:LH56_19305 K12422 long chain fatty acid CoA ligase FadD10 [EC:6.2.1.-] | (GenBank) acyl-CoA synthetase (A)

MSHSPPPTVLERIVAQARTRPDSIALRRCDGRDAMSYGELIGAVEDLAAALEADSVTHGS

RVVVLSDNGPQTYLSVLACARLGAIAVMVDDSLPAATISRFCEIARPAAIIPNKGGGVGA

ENGEIAYPKAQPDSSANEALAMIFTSGTTGEPKAVLLPNRTFFAIPDILRTEGLRWIDWV

AGETTYSPLPATHIGGLWWILNGLMHGASCITGGEQGASLRELLVDNEVATACLVPTLLT

RLVSELKLTGTDVPALRFIAYGGSRAIAADVRFIEAAGVRTAQVYGLSETGCTALCLPTD

SESISRIKQGAVGRPYPGVQTYLSPEGGGGPTTGAESASYGTLWIKSPANMLEYWGSPER

TGEVLAEGWVNTGDLVERRADGFFYIRGRSSEMIISGGVNVVPDEVDRIAEDIAGVREAA

CYEIPDEEFGALVGLAVVPATELDGPGTAALKQRIAATYRRESESMARPSTIVLVEDIPR

TTSGKVMRSALSASLNGARARV

>mys:NF92_19815 K12422 long chain fatty acid CoA ligase FadD10 [EC:6.2.1.-] | (GenBank) acyl-CoA synthetase (A)

MSHSPPPTVLERIVAQARTRPDSIALRRCDGRDAMSYGELIGAVEDLAAALEADSVTHGS

RVVVLSDNGPQTYLSVLACARLGAIAVMVDDSLPAATISRFCEIARPAAIIPNKGGGVGA

ENGEIAYPKAQPDSGANEALVMIFTSGTTGEPKAVLLPNRTFFAIPDILRTEGLRWIDWV

AGETTYSPLPATHIGGLWWILNGLMHGASCITGGEQGASLRELLVDNEVATACLVPTLLT

RLVSELKLTGTDVPALRFIAYGGSRAIAADVRFIEAAGVRTAQVYGLSETGCTALCLPTD

SESISRIEQGAVGRPYPGVQTYLSPEGGGGPTTGAESASYGTLWIKSPANMLEYWGSPER

TGEVLAEGWVNTGDLVERRADGFFYIRGRSSEMIISGGVNVVPDEVDRIAEDIAGVREAA

CYEIPDEEFGALVGLAVVPATELDGPGTAALKQRIAATYRRESESMARPSTIVLVEDIPR

TTSGKVMRSALSASLNGARARV

>myc:NF90_19820 K12422 long chain fatty acid CoA ligase FadD10 [EC:6.2.1.-] | (GenBank) acyl-CoA synthetase (A)

MSHSPPPTVLERIVAQARTRPDSIALRRCDGRDAMSYGELIGAVEDLAAALEADSVTHGS

RVVVLSDNGPQTYLSVLACARLGAIAVMVDDSLPAATISRFCEIARPAAIIPNKGGGVGA

ENGEIAYPKAQPDSGANEALVMIFTSGTTGEPKAVLLPNRTFFAIPDILRTEGLRWIDWV

AGETTYSPLPATHIGGLWWILNGLMHGASCITGGEQGASLRELLVDNEVATACLVPTLLT

RLVSELKLTGTDVPALRFIAYGGSRAIAADVRFIEAAGVRTAQVYGLSETGCTALCLPTD

SESISRIEQGAVGRPYPGVQTYLSPEGGGGPTTGAESASYGTLWIKSPANMLEYWGSPER

TGEVLAEGWVNTGDLVERRADGFFYIRGRSSEMIISGGVNVVPDEVDRIAEDIAGVREAA

CYEIPDEEFGALVGLAVVPATELDGPGTAALKQRIAATYRRESESMARPSTIVLVEDIPR

TTSGKVMRSALSASLNGARARV

>mmc:Mmcs_3828 no KO assigned | (GenBank) AMP-dependent synthetase and ligase (A)

MSISLLLEMSVSADPERTAVVSDGLRLSVEELSALADGGAGVIAKSGAAHVAYVGTGGVL

LPLLLFASARAAIPFTPLNYRLSREGLHELIARLPDALVIADPDYREVVAGAGKQLMDSE

EFLAAARTAEPTAEFADPDSVAVVLFTSGTTSRPKAVELTHNNLTSYVTGTVEFASADPE

DAALICVPPYHIAGVGAAMSNLYAGRKMVYLRHFDAREWIRLVSTEGVTTATVVPTMLDR

IVSALSEEPVALPTLRNLAYGGSKVALPLVRRALELLPGVGFVNAYGLTETSSTIAVLGP

DDHRAALASDDAAVARRLGSVGQIVPGIEVQIRADDGTVLGPGETGELFVRGDQVSGRYT

DIGSVLDADGWFPTKDVASLDEDGYLFIGGRSDDTIIRGGENIAPAEIEDVLVEHPDVRD

VAVVGPEDPQWGQIIVAVVVPAPGADPDADDLRAHVRKHLRGSRTPDRVVFRAELPTNAT

GKVLRRELIEEYAAAARDPEKEPA

>mkm:Mkms_3902 no KO assigned | (GenBank) AMP-dependent synthetase and ligase (A)

MSISLLLEMSVSADPERTAVVSDGLRLSVEELSALADGGAGVIAKSGAAHVAYVGTGGVL

LPLLLFASARAAIPFTPLNYRLSREGLHELIARLPDALVIADPDYREVVAGAGKQLMDSE

EFLAAARTAEPTAEFADPDSVAVVLFTSGTTSRPKAVELTHNNLTSYVTGTVEFASADPE

DAALICVPPYHIAGVGAAMSNLYAGRKMVYLRHFDAREWIRLVSTEGVTTATVVPTMLDR

IVSALSEEPVALPTLRNLAYGGSKVALPLVRRALELLPGVGFVNAYGLTETSSTIAVLGP

DDHRAALASDDAAVARRLGSVGQIVPGIEVQIRADDGTVLGPGETGELFVRGDQVSGRYT

DIGSVLDADGWFPTKDVASLDEDGYLFIGGRSDDTIIRGGENIAPAEIEDVLVEHPDVRD

VAVVGPEDPQWGQIIVAVVVPAPGADPDADDLRAHVRKHLRGSRTPDRVVFRAELPTNAT

GKVLRRELIEEYAAAARDPEKEPA

>mjl:Mjls_4073 no KO assigned | (GenBank) AMP-dependent synthetase and ligase (A)

MLISDIATNNARRYPNKRALVEADRVHTWAEVDARARRLAGFLTGRGLMPGDRVMVIARN

CIEWPEISFGLAKAGLIAVPVNIRLAPDEVAHVRDDCGARAVLIHADHLERFLGELTDLP

LVVGIGARSSTGADEVVTDYETALAQAQPAAPRGDVSPDDVAVILYTSGTTGRAKGVMHT

HRGLLYQAADTNLVTEANRSDVMLATTPFFTAGGMVRTVSWLYLGQTMVIHQRFDPQAVI

DEIERNAITFTTFIPTMLHRTLAILEDGPPRDMSSLRRISYGSAPVPPGLARKAMDLLGC

DLQQRYGLTECGGQATILTPQDHREILAGRTSIATSCGQETPMCAIRVVDVDGNDAQAGE

VGEIVIVSPANAVGYWNRPEQTAETFRPDGLYSGDLGYLDEDGYLHITGRKTDLIISGGF

NVYPAEIERVIAQHPGVDMVAVVGVPDPEWGETPVAAVIPKTHVEDRDALTAELVSLCRA

ELAGYKQPRRFVFREEFPLGPAGKILKREIANQVTEVGPAGAKMPVSTGSTEERP

>mjd:JDM601_2520 no KO assigned | (GenBank) fadD13_1; fatty acid-CoA ligase FadD13_1 (A)

MTGIVEISRRRNPFPATGVIRDGDGVAHYQQLPATLLDVLAEQAQRRPDTEAVVELGAGA

LTYRQLWQRAARVAGGLRADGLGRGDRVGLRYPAGLNWVLAFWGTLLAGGIPVAVNTRSA

APEVDFVLTDAGVRVDLAPGTALPDGQPYVVDGVEAGDIAALFYTSGTTGRPKGVPTTHE

AFLTNAENMVRSMGQSRQLGEELRTLISVPLFHVTGCNSQLLVAAYAGGAAVIMPALSLP

ELIAALPAQRISSMVTVPAVYSLLLRHPDFAGVDVSGVRWVGYGGAPTAPALVRSLRDAF

GAATVFNGYGMTETASLMTVLPDSDAVAHADSVGYAVPSGDLGVAPLDSNPGDPTTGELV

VRGANVMRGYWNRPDATAATIVDGWLHTGDVVRVDDAGRVHIIDRLKDIIIRGGENVSSV

EVEAALLSAPGVADACVLGVPDDVMGEKVGAVLLGAGQQDLDVAAVLDHCRGQLADYKVP

QYITIVADELPRTASGKLLKGRLRERVQWGDPLR

>mmi:MMAR_0258 K12422 long chain fatty acid CoA ligase FadD10 [EC:6.2.1.-] | (GenBank) fadD10; fatty-acid-CoA ligase FadD10 (A)

MSDLPATVLERIIEQAQRRPEAIALRRCDGTSALPYSELAAEVDRYAGALRAQSASRGSR

VLVISDNGPETYLAVLACAKLGAIAVMADGNLPPATIDRFCQITDPVAVLIAPGSKVGSS

SLPEGLAAIPAIRVDIGSGTGEFAHSPDTDRPATEPGLGADDPLAMIFTSGTTGEPKAVL

LANRTFFAVPDILRNEGLNWVTWVDGETTYSPLPATHIGGLWWILTCLMRGGLCITGGEN

TPSLMQILNSNAVNTTCLVPTLLSKLVSELKSAATTVPSLRLLGYGGSRAIAADVRFIEA

TGVRTAQVYGLSETGCTALCLPTDDDSIAKIEAGAVGRPYPGVEVYLAADDEADGAGPNA

PGAGPSASFGTLWIKSPANMLGYWSNPQRTQEVLIDGWVNTGDLLERHEDGFFYIKGRSS

EMIISGGVNIAPDEVDRIAEGVPGVREAACFEIPDPEFGALVGLAVVAATDMDASAARKL

KHTIAAHYRRESESVARPSTIVIVSEIPRTQSGKVMRTSLAAAANQVQTGG

>mrh:MycrhN_1047 no KO assigned | (GenBank) acyl-CoA synthetase (AMP-forming)/AMP-acid ligase II (A)

MPDIANIADIVRVHARLRPDTTALIVGDRTITFAELDARSSQAAQAFRAAGVGFGDRVAF

IEKNSAEFFEVVFGLAKLGAVGVPVNWRLAPPEMAHIIDDAQARIVVVGSEFFGHVEAIE

DGLTTVTGIVAIGTHDRWQSFEDWIDGHVGEDPGVATGSDDVALLMYTSGTTGLPKGVML

SNGNYLCKSTGISQQWRFTADSVSLAVMPMFHMAGSGWVFVGLYEGATTVVLRDVDPIAI

LDSIVQHRITNLLLVPAVIQMMLNSPAVDDADFSSVRAIVYGASPITDDVLIRGLERFGC

EFLQVYGLTETTGSVTQLDEHDPAGRPELLRSCGKPYPWVELRIVDNGGRTVPVGTVGEV

WTRSAQNMRGYWNNPDATAATVTDDGWLKTGDAGYVDRDGYVYLHDRKKDMIVSGGENVY

PIEVENVLMTHAGVDDVAIIGVPDEKWGEAVKAIVVPTAGTAPTEAELIAYARERLAGFK

LPKSVDFTDVLPRNPSGKLLKRVLREPHWQGVDRRIG

>mmm:W7S_08480 K00666 fatty-acyl-CoA synthase [EC:6.2.1.-] | (GenBank) AMP-dependent synthetase and ligase (A)

MPSRTFQFPVSDWVAHHASVRPHAIALASADTGEQVSWAQLEQRVGLAAAALLAHGLRPG

DRIAVVADNAPRAFVLQFAAMRAGVVMVPLNWRLVTAEMRHQCIDAAVSALTHDATWVEP

ARDVADGAGVQTILELETLTSAEPGNGLVPLPPQPHDPDAITHILYTSGTTGTPKGALVS

HASMMWNAFNILTAAQVAAPAVNMLNPMPLFHAGGLNVLANPILMHGGQVTTMARWNPAA

ILAYIGEQTNGVTHLTTAPSLLQTLIDDPTFGTTDFGTMRKIVLGGGTTTPQLLRAFPAK

GVALHPQYGGTETGPAALVLEEGLERALSGTCGKPVLHTAVRLVDPDTLTDVADDVVGEV

WLKGPAVTPGYWNLPNEQYFVDGWFRTGDAARRDADGYFYFAGRYKDMYKSGGENVYAAE

VENVLIDLPEVAEVAIIGVPDAKWGEVGLAVVVASPGADVTLDALQSACAGRIARYKYPK

HLDVVAALPRNATGKVAKAALREHYHSATATPAPTDHESLVTHQKESHDVSRR

>mcb:Mycch_0673 no KO assigned | (GenBank) acyl-CoA synthetase (AMP-forming)/AMP-acid ligase II (A)

MTSPGIGAIADIIRTHGREHPDVPALITGERVITFGDLDARSSQVAQAFAHAGVGAGDRV

AFIERNGVEFFEVVFGLAKLGAVGVPVNWRLAAPEIRHVLQDSGASAVVVGEDFFDRIEA

IEHELSASVIAIGSHARWPEFAAWVAAHPAVDPGVVTGPDDLVFLMYTSGTTGAPKGVML

SNANYAAKTGGVAAGPWRITADAVCLAVMPLFHMAGSGWAFAALWQGATTVVLRDVDPAA

ILDAVATHRITNMLVVPAVIQFLLDTPGIDTADFSHLRVIVYGASPISDDVLVRGIERFG

GVFAQVYGMTETTGSITQLDGEEHLPQLLRSCGRPYPWVRIRVVAPDGRDVAPGTVGEVW

TKSAQNMLGYWNNPEATAATLTADGWLRTGDAGYVDADGYLYLHDRLKDMIVSGGENVYP

IEVENVLMTHPAVSDAAVIGVPDDRWGEAVKAVVVRTRRSSIGEAELIDFARQRLAGFKL

PKSVDFVDALPRTPSGKILKRALREPHWAGADRRIG

>mli:MULP_00230 K12422 long chain fatty acid CoA ligase FadD10 [EC:6.2.1.-] | (GenBank) fadD10; fatty-acid-CoA ligase FadD10 (A)

MSDLPATVLERIIEQAQRRPEAIALRRCDGTSALPYGELAAEVDRYAGALRAQSASRGSR

VLVILDNGPETYLAVLACAKLGAIAVMADGNLPPATIDRFCQITDPVAVLIAPGSKVGSS

SLPEGLAAIPAIRVDIGSGTGEFAHSPDTDRPATEPGLGADDPLAMIFTSGTTGEPKAVL

LANRTFFAVPDILRNEGLNWVTWVDGETTYSPLPATHIGGLWWILTCLMRGGLCITGGEN

TPSLMQILNSNAVNTTCLVPTLLSKLVSELKSAATTVPSLRLLGYGGSRAIAADVRFIEA

TGVRTAQVYGLSETGCTALCLPTDDGSIAKIEAGAVGRPYPGVEVYLAADDEADGAGPNA

PGAGPSASFGTLWIKSPANMLGYWSNPQRTQEVLIDGWVNTDDLLERHEDGFFYIKGRSS

EMIISSGVNIAPDEVDRIAEGVPGVREAACFEIPDPEFGALVGLAVVAATDVDASAARKL

KHTIAAHYRRESESVARPSTIVIVSEIPRTQSGKVMRTSLAAAANQVQTGG

>mkn:MKAN_16235 K00666 fatty-acyl-CoA synthase [EC:6.2.1.-] | (GenBank) long-chain fatty acid--CoA ligase (A)

MTAQLASHLSQATHGLEQPYLARRQNWVNQLQRHALMQPGATALRFTGHTLTWSDLSHRV

SALAGALSRRGVGFGDRVMILMLNRTEFIESVFAANMLGAIAVPLNFRLTPTEIAFLVED

CAPRVLVTEAVLAPVATGVRAIHPLVDTIVVAGGASDDTVLGYDNLLSEPGDPPAPVDIP

NDSPALIMYTSGTTGRPKGAVLTHTNLTGQAITALYTSGANVNSDVGFIGVPLFHIAGIG

NMLTGMMLGVPTVLYPLGAFDPGQLLDVLEAERVTGIFLVPAQWQAVCAEQQARPRNLKL

RVMSWGAAPAPDALLRQMSEVFPGTQILAAFGQTEMSPVTCMLLGEDAIRKRGSVGKVIP

TVAARVVDDQMNDVPVGEVGEIVYRAPTLMSGYWNNPEATAEAFAGGWFHSGDLVRMDED

GYVWVVDRKKDMIISGGENIYCAEVENVLAGHPGIVEVAVIGRPDEKWGEVPVAIAAVTD

APLGIEDLTEYLTERLARYKHPKALEIVEALPRNPAGKVLKTELRLRYGARKSFGNRSAP

TYSTTREDD

>mks:LG40_16065 K00666 fatty-acyl-CoA synthase [EC:6.2.1.-] | (GenBank) long-chain fatty acid--CoA ligase (A)

MTAQLASHLSQATHGLEQPYLARRQNWVNQLQRHALMQPGATALRFTGHTLTWSDLSHRV

SALAGALSRRGVGFGDRVMILMLNRTEFIESVFAANMLGAIAVPLNFRLTPTEIAFLVED

CAPRVLVTEAVLAPVATGVRAIHPLVDTIVVAGGASDDTVLGYDNLLSEPGDPPAPVDIP

NDSPALIMYTSGTTGRPKGAVLTHTNLTGQAITALYTSGANVNSDVGFIGVPLFHIAGIG

NMLTGMMLGVPTVLYPLGAFDPGQLLDVLEAERVTGIFLVPAQWQAVCAEQQARPRNLKL

RVMSWGAAPAPDALLRQMSEVFPGTQILAAFGQTEMSPVTCMLLGEDAIRKRGSVGKVIP

TVAARVVDDQMNDVPVGEVGEIVYRAPTLMSGYWNNPEATAEAFAGGWFHSGDLVRMDED

GYVWVVDRKKDMIISGGENIYCAEVENVLAGHPGIVEVAVIGRPDEKWGEVPVAIAAVTD

APLGIEDLTEYLTERLARYKHPKALEIVEALPRNPAGKVLKTELRLRYGARKSFGNRSAP

TYSTTREDD

>mki:LH54_16180 K00666 fatty-acyl-CoA synthase [EC:6.2.1.-] | (GenBank) long-chain fatty acid--CoA ligase (A)

MTAQLASHLSQATHGLEQPYLARRQNWVNQLQRHALMQPGATALRFTGHTLTWSDLSHRV

SALAGALSRRGVGFGDRVMILMLNRTEFIESVFAANMLGAIAVPLNFRLTPTEIAFLVED

CAPRVLVTEAVLAPVATGVRAIHPLVDTIVVAGGASDDTVLGYDNLLSEPGDPPAPVDIP

NDSPALIMYTSGTTGRPKGAVLTHTNLTGQAITALYTSGANVNSDVGFIGVPLFHIAGIG

NMLTGMMLGVPTVLYPLGAFDPGQLLDVLEAERVTGIFLVPAQWQAVCAEQQARPRNLKL

RVMSWGAAPAPDALLRQMSEVFPGTQILAAFGQTEMSPVTCMLLGEDAIRKRGSVGKVIP

TVAARVVDDQMNDVPVGEVGEIVYRAPTLMSGYWNNPEATAEAFAGGWFHSGDLVRMDED

GYVWVVDRKKDMIISGGENIYCAEVENVLAGHPGIVEVAVIGRPDEKWGEVPVAIAAVTD

APLGIEDLTEYLTERLARYKHPKALEIVEALPRNPAGKVLKTELRLRYGARKSFGNRSAP

TYSTTREDD

>mne:D174_25920 K00666 fatty-acyl-CoA synthase [EC:6.2.1.-] | (GenBank) long-chain fatty acid--CoA ligase (A)

MSSTEQPYLARQQNWSNQLSRHALMQPDKTALRFLGQTTTWADLDRRVDALAAALHERGI

TAGDRVLILMLNRPEFIESFLAANRLGAIAVPVNFRMTPPEIAFLVSDCAARVVVTESVL

APVATAVRDLDSTLSTVIVAGAGTEDGVLGYDDLLAEQNPPPEPVDVPNDSPALIMYTSG

TTGRPKGAVLTHTNISGQSLTHLFTNGADLNHDVGFIGVPLFHIAGIGNMIPGLLLGRPT

VVYPLGAFDPGALLDVLEAEEVTGIFLVPAQWQAVCAAQRAQPRSLKLRVLSWGAAPASD

TLLKDMAQTFPGANILAAFGQTEMSPVTCMLLGEDAIRKLGSVGKVIPTVSARIVDEDMN

DVPIGEVGEIVYRAPTLMAGYWNNPKATAEAFAGGWFHSGDLVRQDDEGYIWVVDRKKDM

IISGGENIYCAEVENVLAAHPAIAEVAVIGRPHEKWGEVPVAVIALAALSGEPTPSALTL

AELDDFLTERLARYKHPKALEIVEALPRNPSGKVLKTELRTMFGS

>myv:G155_20080 K12422 long chain fatty acid CoA ligase FadD10 [EC:6.2.1.-] | (GenBank) acyl-CoA synthetase (A)

MAHPPPTVLARIAERARQRPDAIALRRCDGGSAIRYRDLIAESDKLARQLVSHGIGTGSR

VFVMSDNGPETYLSVLACARVGAIAVMVDGGLPRAAIDRFSEITTPGAVLVTPGSRWQPA

DLGALPIPAVAVHVTADRRGDSVSVSETEPDPSEWGADHPLAMIFTSGTTGTPKAVLLPN

RTFYAIADTLQRERLTWIDWVVGETTYSPLPATHIGGLWWILNCLMQGGLCITGGENTPS

IAEILTANDVATTCLVPTLLARLVSELRAAGAPVPPALRMVGYGGSRAVASDVAFVEAAG

VHTAQVYGLSETGCTALCLPTDDGSIARIDGGAVGRPYPGVEVHLDGDGSGPPTRSGSAS

ASSGTLWIKSPANMLGYWDNTERTAEVLVDGWVNTGDLLDRHADGFFYIRGRSSEMIISG

GVNVAPDEVDRIAEGVAGVDEAACYEIPDAEFGALVGLAVVASANLDDTAAKKLKQAIAA

RFRQECEPMARPSTIRFVDQIPRTQSGKVIRTSLGTVADRTTTVAAGG

>mye:AB431_09575 no KO assigned | (GenBank) fatty-acid--CoA ligase (A)

MAATVASALRWWARTKGDQTALIIGDEQITYRQLHEWTGRLARWLADQGVKPSDRVGVLA

PNTPQWPVAALAVMKCGATLVPLNARLKPAEVRKIADDADISAVIAASTHVDVVDEARTA

GRDFTVWGLGVVDTHRTGDADDFVIEPGPEDPIAVIFTSGSTGLSKGVILTSNTLMGMVL

ENTLTEEGFRPGTVTLLVLPLAFTPGLVYGLLITSVLGGTLIVEPELNPSRAVTLIENHS

VRALFGVPLVFDALSRAEEFADADLSSLQTAIVGGAAVPVDLLRRWAEKGVLLRQIYGMT

ETGGVATATLKAEALEHPDSCGSGSIFTEVAVMLDDGSLAGPGELGELVVRGPGVTPGYW

NDPQTTAAAIRDGWLHSGDVGVRDEQGRITFRDRIKELIISGGINISPVELESAISALDG

VDEVAVIAAPDPRFGETPAAIITVAAGSNLDEAAVVQHCEHVLSDYKVPRYIVLRSEMLP

RLPSGKLDKRAIRAEYSDIPDRFQRAR

>mgo:AFA91_19515 no KO assigned | (GenBank) AMP-dependent synthetase (A)

MGALEVVWGSDIAVEEVRGIPFKTYSQRPRHIGDLLMFADRWGDRPHIVQGSRVVTFTDL

RRAAQVKARELADGGLESGERVLLLGWNSPEWIINFWACAILGAVPVLGNGWWGAGEVAD

AADTAKVTLALVDQRGAAKLPEGLATVPWECDIPGARHAVDFDAAERAAEDSPAVIVFTS

GTSGRPKAVVLAHRSLLAGLQMLLHITRRLPHQVDETTGDAGLHTGPMFHIGGVQTLLRA

IMVGDTLVMPEGSFKPDEALRLIEEWKIARWSAVPTMVSRVLEHPDAQTRDLTSLRSVTV

GGAPVHPEFVELLRQGLPGVQPRVATGYGLTENGGQGTAASGRDTLERPGSCGRPLPCVE

VKIAGATEYVDGEILLRSPTQMLYYYGEDSSPITDDGWLHTGDLGRIDDDGYLYITGRAK

DMIIRGGENIAPAAVEAALAKVPGVVESAVFGVPHADLGEEVMAAVVVSDATTAEQLTEH

LRSSIASFAVPTRWRIETEPLPTNHAGKIDKPAIAAAARAELAGAAQ

>mft:XA26_41000 K12422 long chain fatty acid CoA ligase FadD10 [EC:6.2.1.-] | (GenBank) Long-chain-fatty-acid--CoA ligase (A)

MAHPPPTVLARIAERARQRPDAIALRRCDGGSAIRYRDLIAESDKLARQLVSHGIGTGSR

VFVMSDNGPETYLSVLACARVGAIAVMVDGGLPRAAIDRFSEITTPGAVLVTPGSRWQPA

DLGALPIPAVAVHVTADPRGDSVSVSETEPDPSEWGADHPLAMIFTSGTTGTPKAVLLPN

RTFYAIADTLQRERLTWIDWVVGETTYSPLPATHIGGLWWILNCLMQGGLCITGGENTPS

IAEILTANDVATTCLVPTLLARLVSELRAAGAPVPPALRMVGYGGSRAVASDVAFVEAAG

VHTAQVYGLSETGCTALCLPTDDGSIARIDGGAVGRPYPGVEVHLDGDGSGPPTRSGSAS

ASSGTLWIKSPANMLGYWDNTERTAEVLVDGWVNTGDLLDRHADGFFYIRGRSSEMIISG

GVNVAPDEVDRIAEGVAGVDEAACYEIPDAEFGALVGLAVVASANLDDTAAKKLKQAIAA

RFRQECEPMARPSTIRFVEQIPRTQSGKVIRTSLGTVADRTTTVAAGG

>mhad:B586_11345 K12422 long chain fatty acid CoA ligase FadD10 [EC:6.2.1.-] | (GenBank) acyl-CoA synthetase (A)

MSHLSFTVLERIFEQARQRPKEIALRRCDGTSTLRYGQLVAEVNRLAANLRAQSISQRSR

VFVISDNGPETYLSVLACAKLGAIAVMVDGNLPPATIGRFYEISDPGAILVAPESKIGSS

PLPEALHSIPAITVNIDAGATYSACSVDADCLVGNLDQGADDPLAMIFTSGTTGEPKAVL

LPNRTFFAIPDILREEGMTWIDWVVGETTYSPLPATHIGGLWWILTCLMHGELCITGGEG

TSSLIELLNVNAVATTLLVPTLLSRLVSELKSTDTPVPSLRLVGYGGSRAIASDVRFIEA

AGVRTAQVYGLSETGCTALCLPTADGSISKIEAGAVGRPYPGVEVYLADADGGGPAVADA

ASSASFGTLWIKSPANMLGYWNNPKRTREVLVDGWVNTGDLVERREDGFFYIKGRSSEMI

ISGGVNIVPDEVDRIAESVSGVREAACYEIPDAHFGALVGLAVVPAAKLDESQALELKRR

IAACYRRESESMARPSKIVIVADIPRTQSGKVMRATLAAVLNGEQAGMVVRD

Rv0153c Homologs

>mtu:Rv0153c ptbB; phosphotyrosine protein phosphatase; K01104 protein-tyrosine phosphatase [EC:3.1.3.48] (A)

MAVRELPGAWNFRDVADTATALRPGRLFRSSELSRLDDAGRATLRRLGITDVADLRSSRE

VARRGPGRVPDGIDVHLLPFPDLADDDADDSAPHETAFKRLLTNDGSNGESGESSQSIND

AATRYMTDEYRQFPTRNGAQRALHRVVTLLAAGRPVLTHCFAGKDRTGFVVALVLEAVGL

DRDVIVADYLRSNDSVPQLRARISEMIQQRFDTELAPEVVTFTKARLSDGVLGVRAEYLA

AARQTIDETYGSLGGYLRDAGISQATVNRMRGVLLG

>mtv:RVBD_0153c phosphotyrosine protein phosphatase PtpB; K01104 protein-tyrosine phosphatase [EC:3.1.3.48] (A)

MAVRELPGAWNFRDVADTATALRPGRLFRSSELSRLDDAGRATLRRLGITDVADLRSSRE

VARRGPGRVPDGIDVHLLPFPDLADDDADDSAPHETAFKRLLTNDGSNGESGESSQSIND

AATRYMTDEYRQFPTRNGAQRALHRVVTLLAAGRPVLTHCFAGKDRTGFVVALVLEAVGL

DRDVIVADYLRSNDSVPQLRARISEMIQQRFDTELAPEVVTFTKARLSDGVLGVRAEYLA

AARQTIDETYGSLGGYLRDAGISQATVNRMRGVLLG

>mtc:MT0162 conserved hypothetical protein; K01104 protein-tyrosine phosphatase [EC:3.1.3.48] (A)

MAVRELPGAWNFRDVADTATALRPGRLFRSSELSRLDDAGRATLRRLGITDVADLRSSRE

VARRGPGRVPDGIDVHLLPFPDLADDDADDSAPHETAFKRLLTNDGSNGESGESSQSIND

AATRYMTDEYRQFPTRNGAQRALHRVVTLLAAGRPVLTHCFAGKDRTGFVVALVLEAVGL

DRDVIVADYLRSNDSVPQLRARISEMIQQRFDTELAPEVVTFTKARLSDGVLGVRAEYLA

AARQTIDETYGSLGGYLRDAGISQATVNRMRGVLLG

>mra:MRA_0161 ptbB; phosphotyrosine protein phosphatase PtpB; K01104 protein-tyrosine phosphatase [EC:3.1.3.48] (A)

MAVRELPGAWNFRDVADTATALRPGRLFRSSELSRLDDAGRATLRRLGITDVADLRSSRE

VARRGPGRVPDGIDVHLLPFPDLADDDADDSAPHETAFKRLLTNDGSNGESGESSQSIND

AATRYMTDEYRQFPTRNGAQRALHRVVTLLAAGRPVLTHCFAGKDRTGFVVALVLEAVGL

DRDVIVADYLRSNDSVPQLRARISEMIQQRFDTELAPEVVTFTKARLSDGVLGVRAEYLA

AARQTIDETYGSLGGYLRDAGISQATVNRMRGVLLG

>mtf:TBFG_10154 phosphotyrosine protein phosphatase ptpb; K01104 protein-tyrosine phosphatase [EC:3.1.3.48] (A)

MAVRELPGAWNFRDVADTATALRPGRLFRSSELSRLDDAGRATLRRLGITDVADLRSSRE

VARRGPGRVPDGIDVHLLPFPDLADDDADDSAPHETAFKRLLTNDGSNGESGESSQSIND

AATRYMTDEYRQFPTRNGAQRALHRVVTLLAAGRPVLTHCFAGKDRTGFVVALVLEAVGL

DRDVIVADYLRSNDSVPQLRARISEMIQQRFDTELAPEVVTFTKARLSDGVLGVRAEYLA

AARQTIDETYGSLGGYLRDAGISQATVNRMRGVLLG

>mtb:TBMG_00154 phosphotyrosine protein phosphatase ptpb; K01104 protein-tyrosine phosphatase [EC:3.1.3.48] (A)

MAVRELPGAWNFRDVADTATALRPGRLFRSSELSRLDDAGRATLRRLGITDVADLRSSRE

VARRGPGRVPDGIDVHLLPFPDLADDDADDSAPHETAFKRLLTNDGSNGESGESSQSIND

AATRYMTDEYRQFPTRNGAQRALHRVVTLLAAGRPVLTHCFAGKDRTGFVVALVLEAVGL

DRDVIVADYLRSNDSVPQLRARISEMIQQRFDTELAPEVVTFTKARLSDGVLGVRAEYLA

AARQTIDETYGSLGGYLRDAGISQATVNRMRGVLLG

>mtk:TBSG_00156 phosphotyrosine protein phosphatase ptpb; K01104 protein-tyrosine phosphatase [EC:3.1.3.48] (A)

MAVRELPGAWNFRDVADTATALRPGRLFRSSELSRLDDAGRATLRRLGITDVADLRSSRE

VARRGPGRVPDGIDVHLLPFPDLADDDADDSAPHETAFKRLLTNDGSNGESGESSQSIND

AATRYMTDEYRQFPTRNGAQRALHRVVTLLAAGRPVLTHCFAGKDRTGFVVALVLEAVGL

DRDVIVADYLRSNDSVPQLRARISEMIQQRFDTELAPEVVTFTKARLSDGVLGVRAEYLA

AARQTIDETYGSLGGYLRDAGISQATVNRMRGVLLG

>mtz:TBXG_000154 phosphotyrosine protein phosphatase ptpb; K01104 protein-tyrosine phosphatase [EC:3.1.3.48] (A)

MAVRELPGAWNFRDVADTATALRPGRLFRSSELSRLDDAGRATLRRLGITDVADLRSSRE

VARRGPGRVPDGIDVHLLPFPDLADDDADDSAPHETAFKRLLTNDGSNGESGESSQSIND

AATRYMTDEYRQFPTRNGAQRALHRVVTLLAAGRPVLTHCFAGKDRTGFVVALVLEAVGL

DRDVIVADYLRSNDSVPQLRARISEMIQQRFDTELAPEVVTFTKARLSDGVLGVRAEYLA

AARQTIDETYGSLGGYLRDAGISQATVNRMRGVLLG

>mtg:MRGA327_00980 phosphotyrosine protein phosphatase; K01104 protein-tyrosine phosphatase [EC:3.1.3.48] (A)

MAVRELPGAWNFRDVADTATALRPGRLFRSSELSRLDDAGRATLRRLGITDVADLRSSRE

VARRGPGRVPDGIDVHLLPFPDLADDDADDSAPHETAFKRLLTNDGSNGESGESSQSIND

AATRYMTDEYRQFPTRNGAQRALHRVVTLLAAGRPVLTHCFAGKDRTGFVVALVLEAVGL

DRDVIVADYLRSNDSVPQLRARISEMIQQRFDTELAPEVVTFTKARLSDGVLGVRAEYLA

AARQTIDETYGSLGGYLRDAGISQATVNRMRGVLLG

>mti:MRGA423_23360 hypothetical protein (A)

MRIAAAVVSIGLAVIAGFAVPVADAHPSEPGVVSYAVLGKGSVGNIVGAPMRWEAVFTRP

FQAFWVELPACNNWVDIGLPEVYDDPDLASFNGATTQTSATDQTHLVKQAVGVFASNDAA

DRAFHRVVDRTVGCSGQTTAIHLDDGTTQVWSFAGGPSTGTDEAWTKQEAGTDRRCFVQT

RLRENVLLQAKVCQSGNAGPAVNVLAGAMQNTLG

>mte:CCDC5079_0139 ptbB, phosphotyrosine protein phosphatase PtpB; K01104 protein-tyrosine phosphatase [EC:3.1.3.48] (A)

MRPGRLFRSSELSRLDDAGRATLRRLGITDVADLRSSREVARRGPGRVPDGIDVHLLPFP

DLADDDADDSAPHETAFKRLLTNDGSNGESGESSQSINDAATRYMTDEYRQFPTRNGAQR

ALHRVVTLLAAGRPVLTHCFAGKDRTGFVVALVLEAVGLDRDVIVADYLRSNDSVPQLRA

RISEMIQQRFDTELAPEVVTFTKARLSDGVLGVRAEYLAAARQTIDETYGSLGGYLRDAG

ISQATVNRMRGVLLG

>mtur:CFBS_0166 ptpb; phosphotyrosine protein phosphatase; K01104 protein-tyrosine phosphatase [EC:3.1.3.48] (A)

MAVRELPGAWNFRDVADTATALRPGRLFRSSELSRLDDAGRATLRRLGITDVADLRSSRE

VARRGPGRVPDGIDVHLLPFPDLADDDADDSAPHETAFKRLLTNDGSNGESGESSQSIND

AATRYMTDEYRQFPTRNGAQRALHRVVTLLAAGRPVLTHCFAGKDRTGFVVALVLEAVGL

DRDVIVADYLRSNDSVPQLRARISEMIQQRFDTELAPEVVTFTKARLSDGVLGVRAEYLA

AARQTIDETYGSLGGYLRDAGISQATVNRMRGVLLG

>mtl:CCDC5180_0137 ptbB, phosphotyrosine protein phosphatase PtpB; K01104 protein-tyrosine phosphatase [EC:3.1.3.48] (A)

MAVRELPGAWNFRDVADTATALRPGRLFRSSELSRLDDAGRATLRRLGITDVADLRSSRE

VARRGPGRVPDGIDVHLLPFPDLADDDADDSAPHETAFKRLLTNDGSNGESGESSQSIND

AATRYMTDEYRQFPTRNGAQRALHRVVTLLAAGRPVLTHCFAGKDRTGFVVALVLEAVGL

DRDVIVADYLRSNDSVPQLRARISEMIQQRFDTELAPEVVTFTKARLSDGVLGVRAEYLA

AARQTIDETYGSLGGYLRDAGISQATVNRMRGVLLG

>mto:MTCTRI2_0157 ptbB; phosphotyrosine protein phosphatase PTPB (protein-tyrosine-phosphatase) (PTPase); K01104 protein-tyrosine phosphatase [EC:3.1.3.48] (A)

MAVRELPGAWNFRDVADTATALRPGRLFRSSELSRLDDAGRATLRRLGITDVADLRSSRE

VARRGPGRVPDGIDVHLLPFPDLADDDADDSAPHETAFKRLLTNDGSNGESGESSQSIND

AATRYMTDEYRQFPTRNGAQRALHRVVTLLAAGRPVLTHCFAGKDRTGFVVALVLEAVGL

DRDVIVADYLRSNDSVPQLRARISEMIQQRFDTELAPEVVTFTKARLSDGVLGVRAEYLA

AARQTIDETYGSLGGYLRDAGISQATVNRMRGVLLG

>mtd:UDA_0153c ptbB; ptbB; K01104 protein-tyrosine phosphatase [EC:3.1.3.48] (A)

MAVRELPGAWNFRDVADTATALRPGRLFRSSELSRLDDAGRATLRRLGITDVADLRSSRE

VARRGPGRVPDGIDVHLLPFPDLADDDADDSAPHETAFKRLLTNDGSNGESGESSQSIND

AATRYMTDEYRQFPTRNGAQRALHRVVTLLAAGRPVLTHCFAGKDRTGFVVALVLEAVGL

DRDVIVADYLRSNDSVPQLRARISEMIQQRFDTELAPEVVTFTKARLSDGVLGVRAEYLA

AARQTIDETYGSLGGYLRDAGISQATVNRMRGVLLG

>mtn:ERDMAN_0177 ptbB; phosphotyrosine protein phosphatase; K01104 protein-tyrosine phosphatase [EC:3.1.3.48] (A)

MAVRELPGAWNFRDVADTATALRPGRLFRSSELSRLDDAGRATLRRLGITDVADLRSSRE

VARRGPGRVPDGIDVHLLPFPDLADDDADDSAPHETAFKRLLTNDGSNGESGESSQSIND

AATRYMTDEYRQFPTRNGAQRALHRVVTLLAAGRPVLTHCFAGKDRTGFVVALVLEAVGL

DRDVIVADYLRSNDSVPQLRARISEMIQQRFDTELAPEVVTFTKARLSDGVLGVRAEYLA

AARQTIDETYGSLGGYLRDAGISQATVNRMRGVLLG

>mtj:J112_00845 phosphotyrosine protein phosphatase; K01104 protein-tyrosine phosphatase [EC:3.1.3.48] (A)

MAVRELPGAWNFRDVADTATALRPGRLFRSSELSRLDDAGRATLRRLGITDVADLRSSRE

VARRGPGRVPDGIDVHLLPFPDLADDDADDSAPHETAFKRLLTNDGSNGESGESSQSIND

AATRYMTDEYRQFPTRNGAQRALHRVVTLLAAGRPVLTHCFAGKDRTGFVVALVLEAVGL

DRDVIVADYLRSNDSVPQLRARISEMIQQRFDTELAPEVVTFTKARLSDGVLGVRAEYLA

AARQTIDETYGSLGGYLRDAGISQATVNRMRGVLLG

>mtub:MT7199_0155 ptbB; PHOSPHOTYROSINE protein PHOSPHATASE PTPB (protein-TYROSINE-PHOSPHATASE) (PTPase); K01104 protein-tyrosine phosphatase [EC:3.1.3.48] (A)

MAVRELPGAWNFRDVADTATALRPGRLFRSSELSRLDDAGRATLRRLGITDVADLRSSRE

VARRGPGRVPDGIDVHLLPFPDLADDDADDSAPHETAFKRLLTNDGSNGESGESSQSIND

AATRYMTDEYRQFPTRNGAQRALHRVVTLLAAGRPVLTHCFAGKDRTGFVVALVLEAVGL

DRDVIVADYLRSNDSVPQLRARISEMIQQRFDTELAPEVVTFTKARLSDGVLGVRAEYLA

AARQTIDETYGSLGGYLRDAGISQATVNRMRGVLLG

>mtuc:J113_01090 phosphotyrosine protein phosphatase; K01104 protein-tyrosine phosphatase [EC:3.1.3.48] (A)

MNCRARGTFVTSPTPQAQYSAGRLFRSSELSRLDDAGRATLRRLGITDVADLRSSREVAR

RGPGRVPDGIDVHLLPFPDLADDDADDSAPHETAFKRLLTNDGSNGESGESSQSINDAAT

RYMTDEYRQFPTRNGAQRALHRVVTLLAAGRPVLTHCFAGKDRTGFVVALVLEAVGLDRD

VIVADYLRSNDSVPQLRARISEMIQQRFDTELAPEVVTFTKARLSDGVLGVRAEYLAAAR

QTIDETYGSLGGYLRDAGISQATVNRMRGVLLG

>mtue:J114_00850 phosphotyrosine protein phosphatase; K01104 protein-tyrosine phosphatase [EC:3.1.3.48] (A)

MAVRELPGAWNFRDVADTATALRPGRLFRSSELSRLDDAGRATLRRLGITDVADLRSSRE

VARRGPGRVPDGIDVHLLPFPDLADDDADDSAPHETAFKRLLTNDGSNGESGESSQSIND

AATRYMTDEYRQFPTRNGAQRALHRVVTLLAAGRPVLTHCFAGKDRTGFVVALVLEAVGL

DRDVIVADYLRSNDSVPQLRARISEMIQQRFDTELAPEVVTFTKARLSDGVLGVRAEYLA

AARQTIDETYGSLGGYLRDAGISQATVNRMRGVLLG

>mtx:M943_00840 phosphotyrosine protein phosphatase; K01104 protein-tyrosine phosphatase [EC:3.1.3.48] (A)

MAVRELPGAWNFRDVADTATALRPGRLFRSSELSRLDDAGRATLRRLGITDVADLRSSRE

VARRGPGRVPDGIDVHLLPFPDLADDDADDSAPHETAFKRLLTNDGSNGESGESSQSIND

AATRYMTDEYRQFPTRNGAQRALHRVVTLLAAGRPVLTHCFAGKDRTGFVVALVLEAVGL

DRDVIVADYLRSNDSVPQLRARISEMIQQRFDTELAPEVVTFTKARLSDGVLGVRAEYLA

AARQTIDETYGSLGGYLRDAGISQATVNRMRGVLLG

>mtuh:I917_01145 Phosphotyrosine protein phosphatase PtpB (protein-tyrosine-phosphatase)(PTPase); K01104 protein-tyrosine phosphatase [EC:3.1.3.48] (A)

MAVRELPGAWNFRDVADTATALRPGRLFRSSELSRLDDAGRATLRRLGITDVADLRSSRE

VARRGPGRVPDGIDVHLLPFPDLADDDADDSAPHETAFKRLLTNDGSNGESGESSQSIND

AATRYMTDEYRQFPTRNGAQRALHRVVTLLAAGRPVLTHCFAGKDRTGFVVALVLEAVGL

DRDVIVADYLSQQRLRATTAGPDLRDDPAAFRHRTGTRGGDVHQGPAVRRGPGCPRGVPG

RRTPDH

>mtul:TBHG_00153 phosphotyrosine protein phosphatase PtpB; K01104 protein-tyrosine phosphatase [EC:3.1.3.48] (A)

MAVRELPGAWNFRDVADTATALRPGRLFRSSELSRLDDAGRATLRRLGITDVADLRSSRE

VARRGPGRVPDGIDVHLLPFPDLADDDADDSAPHETAFKRLLTNDGSNGESGESSQSIND

AATRYMTDEYRQFPTRNGAQRALHRVVTLLAAGRPVLTHCFAGKDRTGFVVALVLEAVGL

DRDVIVADYLRSNDSVPQLRARISEMIQQRFDTELAPEVVTFTKARLSDGVLGVRAEYLA

AARQTIDETYGSLGGYLRDAGISQATVNRMRGVLLG

>mtut:HKBT1_0166 ptpb; phosphotyrosine protein phosphatase; K01104 protein-tyrosine phosphatase [EC:3.1.3.48] (A)

MAVRELPGAWNFRDVADTATALRPGRLFRSSELSRLDDAGRATLRRLGITDVADLRSSRE

VARRGPGRVPDGIDVHLLPFPDLADDDADDSAPHETAFKRLLTNDGSNGESGESSQSIND

AATRYMTDEYRQFPTRNGAQRALHRVVTLLAAGRPVLTHCFAGKDRTGFVVALVLEAVGL

DRDVIVADYLRSNDSVPQLRARISEMIQQRFDTELAPEVVTFTKARLSDGVLGVRAEYLA

AARQTIDETYGSLGGYLRDAGISQATVNRMRGVLLG

>mtuu:HKBT2_0166 ptpb; phosphotyrosine protein phosphatase; K01104 protein-tyrosine phosphatase [EC:3.1.3.48] (A)

MAVRELPGAWNFRDVADTATALRPGRLFRSSELSRLDDAGRATLRRLGITDVADLRSSRE

VARRGPGRVPDGIDVHLLPFPDLADDDADDSAPHETAFKRLLTNDGSNGESGESSQSIND

AATRYMTDEYRQFPTRNGAQRALHRVVTLLAAGRPVLTHCFAGKDRTGFVVALVLEAVGL

DRDVIVADYLRSNDSVPQLRARISEMIQQRFDTELAPEVVTFTKARLSDGVLGVRAEYLA

AARQTIDETYGSLGGYLRDAGISQATVNRMRGVLLG

>mtq:HKBS1_0166 ptpb; phosphotyrosine protein phosphatase; K01104 protein-tyrosine phosphatase [EC:3.1.3.48] (A)

MAVRELPGAWNFRDVADTATALRPGRLFRSSELSRLDDAGRATLRRLGITDVADLRSSRE

VARRGPGRVPDGIDVHLLPFPDLADDDADDSAPHETAFKRLLTNDGSNGESGESSQSIND

AATRYMTDEYRQFPTRNGAQRALHRVVTLLAAGRPVLTHCFAGKDRTGFVVALVLEAVGL

DRDVIVADYLRSNDSVPQLRARISEMIQQRFDTELAPEVVTFTKARLSDGVLGVRAEYLA

AARQTIDETYGSLGGYLRDAGISQATVNRMRGVLLG

>mbo:Mb0158c ptbB; phosphotyrosine protein phosphatase PtpB (EC:3.1.3.48); K01104 protein-tyrosine phosphatase [EC:3.1.3.48] (A)

MAVRELPGAWNFRDVADTATALRPGRLFRSSELSRLDDAGRATLRRLGITDVADLRSSRE

VARRGPGRVPDGIDVHLLPFPDLADDDADDSAPHETAFKRLLTNGGSNGESGESSQSIND

AATRYMTDEYRQFPTRNGAQRALHRVVTLLAAGRPVLTHCFAGKDRTGFVVALVLEAVGL

DRDVIVADYLRSNDSVPQLRARISEMIQQRFDTELAPEVVTFTKARLSDGVLGVRAEYLA

AARQTIDETYGSLGGYLRDAGISQATVNRMRGVLLG

>mbb:BCG_0189c ptbB; Phosphotyrosine protein phosphatase ptpB (EC:3.1.3.48); K01104 protein-tyrosine phosphatase [EC:3.1.3.48] (A)

MAVRELPGAWNFRDVADTATALRPGRLFRSSELSRLDDAGRATLRRLGITDVADLRSSRE

VARRGPGRVPDGIDVHLLPFPDLADDDADDSAPHETAFKRLLTNGGSNGESGESSQSIND

AATRYMTDEYRQFPTRNGAQRALHRVVTLLAAGRPVLTHCFAGKDRTGFVVALVLEAVGL

DRDVIVADYLRSNDSVPQLRARISEMIQQRFDTELAPEVVTFTKARLSDGVLGVRAEYLA

AARQTIDETYGSLGGYLRDAGISQATVNRMRGVLLG

>mbt:JTY_0159 ptbB; phosphotyrosine protein phosphatase (EC:3.1.3.48); K01104 protein-tyrosine phosphatase [EC:3.1.3.48] (A)

MAVRELPGAWNFRDVADTATALRPGRLFRSSELSRLDDAGRATLRRLGITDVADLRSSRE

VARRGPGRVPDGIDVHLLPFPDLADDDADDSAPHETAFKRLLTNGGSNGESGESSQSIND

AATRYMTDEYRQFPTRNGAQRALHRVVTLLAAGRPVLTHCFAGKDRTGFVVALVLEAVGL

DRDVIVADYLRSNDSVPQLRARISEMIQQRFDTELAPEVVTFTKARLSDGVLGVRAEYLA

AARQTIDETYGSLGGYLRDAGISQATVNRMRGVLLG

>mbm:BCGMEX_0159c ptbB; Phosphotyrosine protein phosphatase (EC:3.1.3.48); K01104 protein-tyrosine phosphatase [EC:3.1.3.48] (A)

MAVRELPGAWNFRDVADTATALRPGRLFRSSELSRLDDAGRATLRRLGITDVADLRSSRE

VARRGPGRVPDGIDVHLLPFPDLADDDADDSAPHETAFKRLLTNGGSNGESGESSQSIND

AATRYMTDEYRQFPTRNGAQRALHRVVTLLAAGRPVLTHCFAGKDRTGFVVALVLEAVGL

DRDVIVADYLRSNDSVPQLRARISEMIQQRFDTELAPEVVTFTKARLSDGVLGVRAEYLA

AARQTIDETYGSLGGYLRDAGISQATVNRMRGVLLG

>mbk:K60_001700 phosphotyrosine protein phosphatase PTPB (protein-tyrosine-phosphatase) (PTPase); K01104 protein-tyrosine phosphatase [EC:3.1.3.48] (A)

MAVRELPGAWNFRDVADTATALRPGRLFRSSELSRLDDAGRATLRRLGITDVADLRSSRE

VARRGPGRVPDGIDVHLLPFPDLADDDADDSAPHETAFKRLLTNGGSNGESGESSQSIND

AATRYMTDEYRQFPTRNGAQRALHRVVTLLAAGRPVLTHCFAGKDRTGFVVALVLEAVGL

DRDVIVADYLRSNDSVPQLRARISEMIQQRFDTELAPEVVTFTKARLSDGVLGVRAEYLA

AARQTIDETYGSLGGYLRDAGISQATVNRMRGVLLG

>mbx:BCGT_3954 Protein tyrosine phosphatase (EC:3.1.3.48); K01104 protein-tyrosine phosphatase [EC:3.1.3.48] (A)

MAVRELPGAWNFRDVADTATALRPGRLFRSSELSRLDDAGRATLRRLGITDVADLRSSRE

VARRGPGRVPDGIDVHLLPFPDLADDDADDSAPHETAFKRLLTNGGSNGESGESSQSIND

AATRYMTDEYRQFPTRNGAQRALHRVVTLLAAGRPVLTHCFAGKDRTGFVVALVLEAVGL

DRDVIVADYLRSNDSVPQLRARISEMIQQRFDTELAPEVVTFTKARLSDGVLGVRAEYLA

AARQTIDETYGSLGGYLRDAGISQATVNRMRGVLLG

>mbz:LH58_00860 phosphotyrosine protein phosphatase; K01104 protein-tyrosine phosphatase [EC:3.1.3.48] (A)

MAVRELPGAWNFRDVADTATALRPGRLFRSSELSRLDDAGRATLRRLGITDVADLRSSRE

VARRGPGRVPDGIDVHLLPFPDLADDDADDSAPHETAFKRLLTNGGSNGESGESSQSIND

AATRYMTDEYRQFPTRNGAQRALHRVVTLLAAGRPVLTHCFAGKDRTGFVVALVLEAVGL

DRDVIVADYLRSNDSVPQLRARISEMIQQRFDTELAPEVVTFTKARLSDGVLGVRAEYLA

AARQTIDETYGSLGGYLRDAGISQATVNRMRGVLLG

>maf:MAF_01530 ptbB; phosphotyrosine protein phosphatase PtpB (protein-tyrosine-phosphatase) (PTPase) (EC:3.1.3.48); K01104 protein-tyrosine phosphatase [EC:3.1.3.48] (A)

MAVRELPGAWNFRDVADTATALRPGRLFRSSELSRLDDAGRATLRRLGITDVADLRSSRE

VARRGPGRVPDGIDVHLLPFPDLADDDADDSAPHETAFKRLLTNDGSNGESGESSQSIND

AATRYMTDEYRQFPTRNGAQRALHRVVTLLAAGRPVLTHCFAGKDRTGFVVALVLEAVGL

DRDVIVADYLRSNDSVPQLRARISEMIQQRFDTELAPEVVTFTKARLSDGVLGVRAEYLA

AARQTIDETYGSLGGYLRDAGISQATVNRMRGVLLG

>mce:MCAN_01581 ptbB; phosphotyrosine protein phosphatase PTPB (protein-tyrosine-phosphatase) (PTPase); K01104 protein-tyrosine phosphatase [EC:3.1.3.48] (A)

MAVRELPGAWNFRDVADTATALQPGRLFRSSELSRLDDAGRATLRRLGITDVADLRSSRE

VARRGPGRVPDGIDVHLLPFPDLADDEADDSAPHETAFKRLLTNDGSNGEYGESSQSIND

AATRYMTDEYRQFPTRSGAQRALHRVVTLLAAGRPVLTHCFAGKDRTGFVVALVLEAVGL

DRDVIVADYLRSNDSVPQLRARISEMIQQRSDTELAPEVVTFTKARLSDGVLGVRAEYLA

AARQTIDETYGSLGGYLRDAGISQATVNRMRGVLLG

>mcq:BN44_10182 ptbB; Phosphotyrosine protein phosphatase PtpB (protein-tyrosine-phosphatase)(PTPase) (EC:3.1.3.48); K01104 protein-tyrosine phosphatase [EC:3.1.3.48] (A)

MAVRELPGAWNFRDVADTATALQPGRLFRSSELSRLDDAGRATLRRLGITDVADLRSSRE

VARRGPGRVPDGIDVHLLPFPDLADDEADDSAPHETAFKRLLTNDGSNGEYGESSQSIND

AATRYMTDEYRQFPTRSGAQRALHRVVTLLAAGRPVLTHCFAGKDRTGFVVALVLEAVGL

DRDVIVADYLRSNDSVPQLRARISEMIQQRSDTELAPEVVTFTKARLSDGVLGVRAEYLA

AARQTIDETYGSLGGYLRDAGISQATVNRMRGVLLG

>mcv:BN43_10175 ptbB; Phosphotyrosine protein phosphatase PtpB (protein-tyrosine-phosphatase)(PTPase) (EC:3.1.3.48); K01104 protein-tyrosine phosphatase [EC:3.1.3.48] (A)

MAVRELPGAWNFRDVADTATALQPGRLFRSSELSRLDDAGRATLRRLGITDVADLRSSRE

VARRGPGRVPDGIDVHLLPFPDLADDEADDSAPHETAFKRLLTNDGSNGEYGESSQSIND

AATRYMTDEYRQFPTRSGAQRALHRVVTLLAAGRPVLTHCFAGKDRTGFVVALVLEAVGL

DRDVIVADYLRSNDSVPQLRARISEMIQQRSDTELAPEVVTFTKARLSDGVLGVRAEYLA

AARQTIDETYGSLGGYLRDAGISQATVNRMRGVLLG

>mcx:BN42_10192 ptbB; Phosphotyrosine protein phosphatase PtpB (protein-tyrosine-phosphatase)(PTPase) (EC:3.1.3.48); K01104 protein-tyrosine phosphatase [EC:3.1.3.48] (A)

MAVRELPGAWNFRDVADTATALQPGRLFRSSELSRLDDAGRATLHRLGITDVADLRSSRE

VARRGPGRVPDGIDVHLLPFPDLADDEADDSAPHETAFKRLLTNDGSNGEYGESSQSIND

AATRYMTDEYRQFPTRSGAQRALHRVVTLLAAGRPVLTHCFAGKDRTGFVVALVLEAVGL

DRDVIVADYLRSNDSVPQLRARISEMIQQRSDTELAPEVVTFTKARLSDGVLGVRAEYLA

AARQTIDETYGSLGGYLRDAGISQATVNRMRGVLLG

>mcz:BN45_10171 ptbB; Phosphotyrosine protein phosphatase PtpB (protein-tyrosine-phosphatase)(PTPase) (EC:3.1.3.48); K01104 protein-tyrosine phosphatase [EC:3.1.3.48] (A)

MAVRELPGAWNFRDVADTATALRPGRLFRSSELSRLDDAGRATLRRLGITDVADLRSSRE

VARRGPGRVPDGIDVHLLPFPDLADDDADDSAPHETAFKRLLTNDGSNGESGESSQSIND

AATRYMTDEYRQFPTRSGAQRALHRVVTLLAAGRPVLTHCFAGKDRTGFVVALVLEAVGL

DRDVIVADYLRSNDSVPQLRARISEMIQQRFDTELAPEVVTFTKARLSDVVLGVRAEYLA

AARQTIDETYGSLGGYLRDAGISQATVNRMRGVLLG

>mle:ML1635 panB; 3-methyl-2-oxobutanoate hydroxymethyltransferase (EC:2.1.2.11); K00606 3-methyl-2-oxobutanoate hydroxymethyltransferase [EC:2.1.2.11] (A)

MSEQNVNVYGADPSTSSIQSIQRTKIRTKHLQKMKAEGHKWAMLTAYDYSTARVFDEAGI

PVLLVGDSAANVVYGYDTTVPVSADELLPLVRGVVRGAGHALVVADLPFGSYEPGPTAAL

AVATRFMKEGGAHAVKLEGGQRVAEQIACLTAAGIPVMAHIGFTPQSVNSLGGFRVQGRG

GDAEQTVADAVAVAEAGAFSVVMEMVPTELATQITGKLTIPTIGIGAGLNCDAQVLVWQD

MAGLSSGKVARFVKQYADIAGELRRAAMQYAEEVASAVFPAEEHCF

>mlb:MLBr01635 panB; 3-methyl-2-oxobutanoate hydroxymethyltransferase; K00606 3-methyl-2-oxobutanoate hydroxymethyltransferase [EC:2.1.2.11] (A)

MSEQNVNVYGADPSTSSIQSIQRTKIRTKHLQKMKAEGHKWAMLTAYDYSTARVFDEAGI

PVLLVGDSAANVVYGYDTTVPVSADELLPLVRGVVRGAGHALVVADLPFGSYEPGPTAAL

AVATRFMKEGGAHAVKLEGGQRVAEQIACLTAAGIPVMAHIGFTPQSVNSLGGFRVQGRG

GDAEQTVADAVAVAEAGAFSVVMEMVPTELATQITGKLTIPTIGIGAGLNCDAQVLVWQD

MAGLSSGKVARFVKQYADIAGELRRAAMQYAEEVASAVFPAEEHCF

>mpa:MAP_3568c hypothetical protein; K01104 protein-tyrosine phosphatase [EC:3.1.3.48] (A)

MIDEYRQFPTRNGAQRALHRVISLLGAGRAVLTHCFAGKDRTGFVVATVLEAIGVDRDVI

VADFLRSNDAAPALRAQISAMIAQRQDTELTPEVVTWTEARLSDGVLGVREEYLAAARQT

IDEKFGSLQAYLRDAGVGEADVQRLRAALLA

>mao:MAP4_2648 transcriptional regulatory protein moxR1; K03924 MoxR-like ATPase [EC:3.6.3.-] (A)

MTAAGGPPPGAGGYSGPGGQSGPGAHEAPTGGAGAGNGLAAEVQTLERAIFEVKRIIVGQ

DQLVERMLVGLLSKGHVLLEGVPGVAKTLAVETFAKVVGGTFARIQFTPDLVPTDIIGTR

IYRQGKEEFDTELGPVVVNFLLADEINRAPAKVQSALLEVMQERHVSIGGKTFPLPNPFL

VMATQNPIEHEGVYPLPEAQRDRFLFKINVGYPSPEEEREIIYRMGVRPPEPKQILNTGD

LVRLQDIAANVFVHHALVDYVVRVVTATRHPEQLGMNDVKTWISFGASPRASLGIIAGAR

SLALVRGRDYVIPQDVVDVIPDVLRHRLVLTYDALADEISPEIVINRVLQTVALPQVNAV

PQQGHSVPPVMQPAGAASGR

>mavi:RC58_13135 ATPase; K03924 MoxR-like ATPase [EC:3.6.3.-] (A)

MTAAGGPPPGAGGYSGPGGQSGPGAHEAPTGGAGAGNGLAAEVQTLERAIFEVKRIIVGQ

DQLVERMLVGLLSKGHVLLEGVPGVAKTLAVETFAKVVGGTFARIQFTPDLVPTDIIGTR

IYRQGKEEFDTELGPVVVNFLLADEINRAPAKVQSALLEVMQERHVSIGGKTFPLPNPFL

VMATQNPIEHEGVYPLPEAQRDRFLFKINVGYPSPEEEREIIYRMGVRPPEPKQILNTGD

LVRLQDIAANVFVHHALVDYVVRVVTATRHPEQLGMNDVKTWISFGASPRASLGIIAGAR

SLALVRGRDYVIPQDVVDVIPDVLRHRLVLTYDALADEISPEIVINRVLQTVALPQVNAV

PQQGHSVPPVMQPAGAASGR

>mavu:RE97_00975 hypothetical protein; K01104 protein-tyrosine phosphatase [EC:3.1.3.48] (A)

MIDEYRQFPTRNGAQRALHRVISLLGAGRAVLTHCFAGKDRTGFVVATVLEAIGVDRDVI

VADFLRSNDAAPALRAQISAMIAQRQDTELTPEVVTWTEARLSDGVLGVREEYLAAARQT

IDEKFGSLQAYLRDAGVGEADVQRLRAALLA

>mav:MAV_5145 phosphotyrosine protein phosphatase ptpb; K01104 protein-tyrosine phosphatase [EC:3.1.3.48] (A)

MPEALRELSGAWNFRDVADGAPMLRPGRLFRSGELSGLDDEGRATLRRLGITDVADLRAA

REVARRGPGRVPDGVEVHLLPFPDLGEHEAGTDDQAPHEHAFQRLLTGDGAEQSAESVDE

AATRYMIDEYRQFPTRNGAQRALHRVISLLAAGRAVLTHCFAGKDRTGFVVATVLEAVGV

DRDVIVADFLRSNDAAPALRAQISAMIAQRQDTELTPEVVTWTEARLSDGVLGVREEYLA

AARQTIDEKFGSLQAYLRDAGVGEADVQRLRAALLA

>mavr:LA63_23790 phosphotyrosine protein phosphatase; K01104 protein-tyrosine phosphatase [EC:3.1.3.48] (A)

MPEALRELSGAWNFRDVADGAPMLRPGRLFRSGELSGLDDEGRATLRRLGITDVADLRAA

REVARRGPGRVPDGVEVHLLPFPDLGEHEAGTDDQAPHEHAFQRLLTGDGAEQSAESVDE

AATRYMIDEYRQFPTRNGAQRALHRVISLLAAGRAVLTHCFAGKDRTGFVVATVLEAVGV

DRDVIVADFLRSNDAAPALRAQISAMIAQRQDTELTPEVVTWTEARLSDGVLGVREEYLA

AARQTIDEKFGSLQAYLRDAGVGEADVQRLRAALLA

>mavd:NF84_14890 ATPase; K03924 MoxR-like ATPase [EC:3.6.3.-] (A)

MTAAGGPPPGAGGYSGPGGQSGPGAHEAPTGGAGAGNGLAAEVQTLERAIFEVKRIIVGQ

DQLVERMLVGLLSKGHVLLEGVPGVAKTLAVETFAKVVGGTFARIQFTPDLVPTDIIGTR

IYRQGKEEFDTELGPVVVNFLLADEINRAPAKVQSALLEVMQERHVSIGGKTFPLPNPFL

VMATQNPIEHEGVYPLPEAQRDRFLFKINVGYPSPEEEREIIYRMGVRPPEPKQILNTGD

LVRLQDIAANVFVHHALVDYVVRVVTATRHPEQLGMNDVKTWISFGASPRASLGIIAGAR

SLALVRGRDYVIPQDVVDVIPDVLRHRLVLTYDALADEISPEIVINRVLQTVALPQVNAV

PQQGHSVPPVMQPAGAASGR

>mava:LA64_23755 phosphotyrosine protein phosphatase; K01104 protein-tyrosine phosphatase [EC:3.1.3.48] (A)

MPEALRELSGAWNFRDVADGAPMLRPGRLFRSGELSGLDDEGRATLRRLGITDVADLRAA

REVARRGPGRVPDGVEVHLLPFPDLGEHEAGTDDQAPHEHAFQRLLTGDGAEQSAESVDE

AATRYMIDEYRQFPTRNGAQRALHRVISLLAAGRAVLTHCFAGKDRTGFVVATVLEAVGV

DRDVIVADFLRSNDAAPALRAQISAMIAQRQDTELTPEVVTWTEARLSDGVLGVREEYLA

AARQTIDEKFGSLQAYLRDAGVGEADVQRLRAALLA

>mit:OCO_49720 phosphotyrosine protein phosphatase ptpb; K01104 protein-tyrosine phosphatase [EC:3.1.3.48] (A)

MTEALRELSGAWNFRDVSDGAPALKPGRLFRSGELSGLDDDGRATLSRLGITDVADLRAA

REVARRGPGLVPDGVEVHLLPFPDLGEQDAGTDDAAPHEHAFQRLLTGEGVEESEQSVNE

AATRYMIDEYRQFPTRNGAQRALHRVISLLADGHSVLTHCFAGKDRTGFVVATVLEAIGI

DRDTILADFLRSNDAAPALRAQISAMIAQRQDAELTPEVVTWTEARLSDGVLGVREVYLA

AARQTIDEEFGSLDAYLRAASVSESDVERLREALLA

>mir:OCQ_50710 phosphotyrosine protein phosphatase ptpb; K01104 protein-tyrosine phosphatase [EC:3.1.3.48] (A)

MTEALRELSGAWNFRDVSDGAPALKPGRLFRSGELSGLDDDGRATLSRLGITDVADLRAA

REVARRGPGLVPDGVEVHLLPFPDLGEQDAGTDDAAPHEHAFQRLLTGEGVEESEQSVNE

AATRYMIDEYRQFPTRNGAQRALHRVISLLADGHSVLTHCFAGKDRTGFVVATVLEAIGI

DRDTILADFLRSNDAAPALRAQISAMIAQRQDAELTPEVVTWTEARLSDGVLGVREVYLA

AARQTIDEEFGSLDAYLRAASVSESDVERLREALLA

>mia:OCU_49650 phosphotyrosine protein phosphatase ptpb; K01104 protein-tyrosine phosphatase [EC:3.1.3.48] (A)

MTEALRELSGAWNFRDVSDGAPALKPGRLFRSGELSGLDDDGRATLSRLGITDVADLRAA

REVARRGPGLVPDGVEVHLLPFPDLGEQDAGTDDAAPHEHAFQRLLTGEGVEESEQSVNE

AATRYMIDEYRQFPTRNGAQRALHRVISLLADGHSVLTHCFAGKDRTGFVVATVLEAIGI

DRDTILADFLRSNDAAPALRAQISAMIAQRQDAELTPEVVTWTEARLSDGVLGVREVYLA

AARQTIDEEFGSLDAYLRAASVSESDVERLREALLA

>mie:LG41_23565 phosphotyrosine protein phosphatase; K01104 protein-tyrosine phosphatase [EC:3.1.3.48] (A)

MTEALRELSGAWNFRDVSDGAPALKPGRLFRSGELSGLDDDGRATLSRLGITDVADLRAA

REVARRGPGLVPDGVEVHLLPFPDLGEQDAGIDDAAPHEHAFQRLLTGEGVEESEQSVNE

AATRYMIDEYRQFPTRNGAQRALHRVISLLADGHSVLTHCFAGKDRTGFVVATVLEAIGI

DRDTILADFLRSNDAAPALRAQISAMIAQRQDAELTPEVVTWTEARLSDGVLGVREVYLA

AARQTIDEEFGSLDAYLRAASVSESDVERLREALLA

>mid:MIP_07528 Putative tyrosine-protein phosphatase; K01104 protein-tyrosine phosphatase [EC:3.1.3.48] (A)

MTEALRELSGAWNFRDVSDGAPALKPGRLFRSGELSGLDDDGRATLSRLGITDVADLRAA

REVARRGPGLVPDGVEVHLLPFPDLGEQDAGTDDAAPHEHAFQRLLTGEGVEESEQSVNE

AATRYMIDEYRQFPTRNGAQRALHRVISLLADGHSVLTHCFAGKDRTGFVVATVLEAIGI

DRDTILADFLRSNDAAPALRAQISAMIAQRQDAELTPEVVTWTEARLSDGVLGVREVYLA

AARQTIDEEFGSLDAYLRAASVSESDVERLREALLA

>myo:OEM_49890 phosphotyrosine protein phosphatase ptpb; K01104 protein-tyrosine phosphatase [EC:3.1.3.48] (A)

MTEALRELSGAWNFRDVSDGAPALKPGRLFRSGELSGLDDDGRATLSRLGITDVADLRAA

REVARRGPGLVPDGVEVHLLPFPDLGEQDAGTDDAAPHEHAFQRLLTGEGAEEQSGQSVD

EAATRYMIDEYRQFPTRNGAQRALHRVISLLADGHSVLTHCFAGKDRTGFVVATVLEAIG

IDRDTILADFLRSNDAAPALRAQISAMIAQRQDAELTPEVVTWTEARLSDGVLGVREVYL

AAARQTIDEEFGSLDAYLRAASVSESDVERLREALLA

>msm:MSMEG_0100 phosphotyrosine protein phosphatase ptpb; K01104 protein-tyrosine phosphatase [EC:3.1.3.48] (A)

MTGPDGTSLELGGAWNFRDVAQETGIRPGVLYRSSELSKLSDDGRAVFKRLGITDVADLR

SHQEVQRRGPGQVPDGVAVHLLPFHPDDTSGQDAPHESTFQRVMSESPDGEDVTESARRY

MTEVYEEFPTLPGAHNAVRQVVSLLAAGRPVIAHCFAGKDRTGFTVATVLDAAGVDRDDI

FADFLRSNEAITPLRNRIMDSVRARSGDEPEIITFAEARLTDEVLGVREEYLAAAWKRLE

EAYGSLSGFMTAAGISPEELTALRDALR

>msg:MSMEI_0097 ptbB; Phosphotyrosine protein phosphatase PtpB (EC:3.1.3.48); K01104 protein-tyrosine phosphatase [EC:3.1.3.48] (A)

MTGPDGTSLELGGAWNFRDVAQETGIRPGVLYRSSELSKLSDDGRAVFKRLGITDVADLR

SHQEVQRRGPGQVPDGVAVHLLPFHPDDTSGQDAPHESTFQRVMSESPDGEDVTESARRY

MTEVYEEFPTLPGAHNAVRQVVSLLAAGRPVIAHCFAGKDRTGFTVATVLDAAGVDRDDI

FADFLRSNEAITPLRNRIMDSVRARSGDEPEIITFAEARLTDEVLGVREEYLAAAWKRLE

EAYGSLSGFMTAAGISPEELTALRDALR

>msb:LJ00_00510 phosphotyrosine protein phosphatase; K01104 protein-tyrosine phosphatase [EC:3.1.3.48] (A)

MTGPDGTSLELGGAWNFRDVAQETGIRPGVLYRSSELSKLSDDGRAVFKRLGITDVADLR

SHQEVQRRGPGQVPDGVAVHLLPFHPDDTSGQDAPHESTFQRVMSESPDGEDVTESARRY

MTEVYEEFPTLPGAHNAVRQVVSLLAAGRPVIAHCFAGKDRTGFTVATVLDAAGVDRDDI

FADFLRSNEAITPLRNRIMDSVRARSGDEPEIITFAEARLTDEVLGVREEYLAAAWKRLE

EAYGSLSGFMTAAGISPEELTALRDALR

>msn:LI99_00510 phosphotyrosine protein phosphatase; K01104 protein-tyrosine phosphatase [EC:3.1.3.48] (A)

MTGPDGTSLELGGAWNFRDVAQETGIRPGVLYRSSELSKLSDDGRAVFKRLGITDVADLR

SHQEVQRRGPGQVPDGVAVHLLPFHPDDTSGQDAPHESTFQRVMSESPDGEDVTESARRY

MTEVYEEFPTLPGAHNAVRQVVSLLAAGRPVIAHCFAGKDRTGFTVATVLDAAGVDRDDI

FADFLRSNEAITPLRNRIMDSVRARSGDEPEIITFAEARLTDEVLGVREEYLAAAWKRLE

EAYGSLSGFMTAAGISPEELTALRDALR

>msh:LI98_00510 phosphotyrosine protein phosphatase; K01104 protein-tyrosine phosphatase [EC:3.1.3.48] (A)

MTGPDGTSLELGGAWNFRDVAQETGIRPGVLYRSSELSKLSDDGRAVFKRLGITDVADLR

SHQEVQRRGPGQVPDGVAVHLLPFHPDDTSGQDAPHESTFQRVMSESPDGEDVTESARRY

MTEVYEEFPTLPGAHNAVRQVVSLLAAGRPVIAHCFAGKDRTGFTVATVLDAAGVDRDDI

FADFLRSNEAITPLRNRIMDSVRARSGDEPEIITFAEARLTDEVLGVREEYLAAAWKRLE

EAYGSLSGFMTAAGISPEELTALRDALR

>msa:Mycsm_00058 protein tyrosine/serine phosphatase; K01104 protein-tyrosine phosphatase [EC:3.1.3.48] (A)

MAVGTGELSGAWNFRDVAEETGVRPGRFFRSSELSHLDDSGREAIRRLGITDVADLRSAR

EVERHGPGLVPSGVEVHLLHFHEVSAVDGEAPHEAAFQRMMGEKPDDEDIAVAARRFMTE

EYLRFPTLSGAQRAVRQVISLLADERPVIAHCFAGKDRTGFTVATVLDAVGVDRDAVVAD

FLRSNDAVPRLREQILASIRDRTDTAEEVTFAEARLTDAVLGVQEDYLAASRRSIEQNYG

SLQGYLEAADVPPIDLARVRTALLG

>mul:MUL_4739 ptpB; phosphotyrosine protein phosphatase PtpB; K01104 protein-tyrosine phosphatase [EC:3.1.3.48] (A)

MAETGPELPGAWNFRDVADSTAALRPGRLFRSSELSGLDDQGRAALRRFKITDVADLRSS

REVARRGPGQVPAGVDVHLLPFPDLADDQAGTDDNAPHETAFKGLLTDGDAGESSEAIDQ

VAARYMADEYRQFPMRTGAQRAVHRVFTLLAAGRPVLTHCFAGKDRTGFVVATVLEAVGV

DREVILADYLRSNDAVPQLRDRVSEMIRERTDVELTPEVVTFTKARLSDGVLGVRPEYLA

AAWQTIDETFGSLDGYLADSGITEADRHQLRSALLG

>mva:Mvan_0115 protein tyrosine/serine phosphatase; K01104 protein-tyrosine phosphatase [EC:3.1.3.48] (A)

MAAAGDELSGAWNFRDVSEQTGVAPGRFFRASELSKLDDTGRAALKGLGVTDVADLRTSR

ELERHGPGLVPAGVEIHHLPFVETMAADGESPHEHAFQRMMTDKPDDEPVADAAARYMTE

EYGRIATAPFARRAVHRVVNLLGSERAVLAHCFAGKDRTGFTIAVVLEAAGVDRDAIMAD

YLRSNVAVPRLRESILATVRERAVDAPEIMELAEARLTDAVLGVREEYLDSARRTIDDQF

GSLTGYLESADVTDEDIRRLRKVLLG

>mgi:Mflv_0735 protein tyrosine/serine phosphatase; K01104 protein-tyrosine phosphatase [EC:3.1.3.48] (A)

MPFSGRELSGAWNFRDVSEPAGIAPGHLFRSSELSKLDDDGRGALIGFGVTDVADLRTAR

ELERHGPGRVPTEVEVHHLPFVETMAADGESPHEHAFQRMMTDKPDGEPIAEAAARYMSE

EYGRIATAPLAQRAVHRVVTLLGSGRSVLAHCFAGKDRTGFTVAVVLEAAGVDRDAIMAD

YLRSNEAVPQLRESILETIRVRAAEAPEIMEFAEARLTDSVLGVREDYLDVARRTLEAEF

GSLRDYLESAGVTDEDLARLRRVLHG

>msp:Mspyr1_00970 protein tyrosine/serine phosphatase; K01104 protein-tyrosine phosphatase [EC:3.1.3.48] (A)

MPFSGRELSGAWNFRDVSEPAGIAPGHLFRSSELSKLDDDGRGALIGFGVTDVADLRTAR

ELERHGPGRVPTEVEVHHLPFVETMAADGESPHEHAFQRMMTDKPDGEPIAEAAARYMSE

EYGRIATAPLAQRAVHRVVTLIGSGRSVLAHCFAGKDRTGFTIAVVLEAAGVDRDAIMAD

YLRSNEAVPQLRESILETVRLRAAEAPEIMELAEARLTDAVLGVREDYLDVARRTLEAEF

GSLRGYLESAGVTDEDLARLRRALHG

>mab:MAB_4591 Putative phosphotyrosine protein phosphatase; K01104 protein-tyrosine phosphatase [EC:3.1.3.48] (A)

MTISSAESKASPADNPVEGIWNFRDVGGADSPVTIRPGVLFRSSELSGLTPAGAQTLRAF

GVTDLYDLRSPTEIARTGSDRVDGGIRVHEVPFATPAAGEERAPHEIAYRQMMLESMTAG

RGPDPASQVEFMLAEYRRFPELPGTLVALKAVVDQLSSGATALVHCAAGKDRTGLLVGLL

LSSVGVDYADIAADYLRSNAAIGAMRAHISAQMNAAEPELAQTMAAMLSDEVLGVREEYL

DTGFRQVANVFGSPEAMLVAAGVDDDGLTALRAALT

>mabb:MASS_4618 phosphotyrosine protein phosphatase ptpb; K01104 protein-tyrosine phosphatase [EC:3.1.3.48] (A)

MTISSAESKASPADNPVEGIWNFRDVGGADSPVTIRPGVLFRSSELSGLTPAGAQTLRAF

GVTDLYDLRSPTEIARTGSDRVDGGIRVHEVPFATPAAGEERAPHEIAYRQMMLESMTAG

RGPDPASQVEFMLAEYRRFPELPGTLVALKAVVDQLSSGATALVHCAAGKDRTGLLVGLV

LSSVGIDYADIAADYLRSNAAIGAMRAHISAQMNAAEPELAQTMAAMLSDEVLGVREEYL

DTGFRQVTNVFGSPEAMLAAAGVDDEGLTALRAALT

>mmv:MYCMA_03570 phosphotyrosine protein phosphatase; K01104 protein-tyrosine phosphatase [EC:3.1.3.48] (A)

MTISSAESKASPADNPVEGIWNFRDVGGADSPVTIRPGVLFRSSELSGLTPAGAQTLRAF

GVTDLYDLRSPTEIARTGSDRVDGGIRVHEVPFATPAAGEERAPHEIAYRQMMLESMTAG

RGPDPASQVEFMLAEYRRFPELPGTLVALKAVVDQLSSGATALVHCAAGKDRTGLLVGLV

LSSVGIDYADIAADYLRSNAAIGAMRAHISAQMNAAEPELAQTMAAMLSDEVLGVREEYL

DTGFRQVTNVFGSPEAMLAAAGVDDEGLTALRAALT

>may:LA62_23300 phosphotyrosine protein phosphatase; K01104 protein-tyrosine phosphatase [EC:3.1.3.48] (A)

MTISSAESKASPADNPVEGIWNFRDVGGADSPVTIRPGVLFRSSELSGLTPAGAQTLRAF

GVTDLYDLRSPTEIARTGSDRVDGGIRVHEVPFATPAAGEERAPHEIAYRQMMLESMTAG

RGPDPASQVEFMLAEYRRFPELPGTLVALKAVVDQLSSGATALVHCAAGKDRTGLLVGLL

LSSVGVDYADIAADYLRSNAAIGAMRAHISAQMNAAEPELAQTMAAMLSDEVLGVREEYL

DTGFRQVANVFGSPEAMLVAAGVDDDGLTALRAALT

>mabo:NF82_22985 phosphotyrosine protein phosphatase; K01104 protein-tyrosine phosphatase [EC:3.1.3.48] (A)

MTISSAESKASPADNPVEGIWNFRDVGGADSPVTIRPGVLFRSSELSGLTPAGAQTLRAF

GVTDLYDLRSPTEIARTGSDRVDGGIRVHEVPFATPAAGEERAPHEIAYRQMMLESMTAG

RGPDPASQVEFMLAEYRRFPELPGTLVALKAVVDQLSSGATALVHCAAGKDRTGLLVGLL

LSSVGVDYADIAADYLRSNAAIGAMRAHISAQMNAAEPELAQTMAAMLSDEVLGVREEYL

DTGFRQVANVFGSPEAMLVAAGVDDDGLTALRAALT

>mabl:MMASJCM_4647 protein tyrosine phosphatase; K01104 protein-tyrosine phosphatase [EC:3.1.3.48] (A)

MTISSAESKASPADNPVEGIWNFRDVGGADSPVTIRPGVLFRSSELSGLTPAGAQTLRAF

GVTDLYDLRSPTEIARTGSDRVDGGIRVHEVPFATPAVGEERAPHEIAYRQMMLESMTAG

RGPDPASQVEFMLAEYRRFPELPGTLVALKAVVDQLSSGATALVHCAAGKDRTGLLVGLV

LSSVGIDYADIAADYLRSNAAIGAMRAHISAQMNAAEPELAQTMAAMLSDEVLGVREEYL

DTGFRQVTNVFGSPEAMLAAAGVDDEGLTALRAALT

>maz:LA61_23195 phosphotyrosine protein phosphatase; K01104 protein-tyrosine phosphatase [EC:3.1.3.48] (A)

MTISSAESKASPADNPVEGIWNFRDVGGADSPVTIRPGVLFRSSELSGLTPAGAQTLRAF

GVTDLYDLRSPTEIARTGSDRVDGGIRVHEVPFATPAAGEERAPHEIAYRQMMLESMTAG

RGPDPASQVEFMLAEYRRFPELPGTLVALKAVVDQLSSGATALVHCAAGKDRTGLLVGLL

LSSVGVDYADIAADYLRSNAAIGAMRAHISAQMNAAEPELAQTMAAMLSDEVLGVREEYL

DTGFRQVANVFGSPEAMLVAAGVDDDGLTALRAALT

>mak:LH56_01410 phosphotyrosine protein phosphatase; K01104 protein-tyrosine phosphatase [EC:3.1.3.48] (A)

MTISSAESKASPADNPVEGIWNFRDVGGADSPVTIRPGVLFRSSELSGLTPAGAQTLRAF

GVTDLYDLRSPTEIARTGSDRVDGGIRVHEVPFATPAAGEERAPHEIAYRQMMLESMTAG

RGPDPASQVEFMLAEYRRFPELPGTLVALKAVVDQLSSGATALVHCAAGKDRTGLLVGLV

LSSVGIDYADIAADYLRSNAAIGAMRAHISAQMNAAEPELAQTMAAMLSDEVLGVREEYL

DTGFRQVTNVFGSPEAMLAAAGVDDEGLTALRAALT

>mys:NF92_01470 phosphotyrosine protein phosphatase; K01104 protein-tyrosine phosphatase [EC:3.1.3.48] (A)

MTISSAESKASPADNPVEGIWNFRDVGGADSPVTIRPGVLFRSSELSGLTPAGAQTLRAF

GVTDLYDLRSPTEIARTGSDRVDGGIRVHEVPFATPAAGEERAPHEIAYRQMMLESMTAG

RGPDPASQVEFMLAEYRRFPELPGTLVALKAVVDQLSSGATALVHCAAGKDRTGLLVGLV

LSSVGIDYADIAADYLRSNAAIGAMRAHISAQMNAAEPELAQTMAAMLSDEVLGVREEYL

DTGFRQVTNVFGSPEAMLAAAGVDDEGLTALRAALT

>myc:NF90_01470 phosphotyrosine protein phosphatase; K01104 protein-tyrosine phosphatase [EC:3.1.3.48] (A)

MTISSAESKASPADNPVEGIWNFRDVGGADSPVTIRPGVLFRSSELSGLTPAGAQTLRAF

GVTDLYDLRSPTEIARTGSDRVDGGIRVHEVPFATPAAGEERAPHEIAYRQMMLESMTAG

RGPDPASQVEFMLAEYRRFPELPGTLVALKAVVDQLSSGATALVHCAAGKDRTGLLVGLV

LSSVGIDYADIAADYLRSNAAIGAMRAHISAQMNAAEPELAQTMAAMLSDEVLGVREEYL

DTGFRQVTNVFGSPEAMLAAAGVDDEGLTALRAALT

>mmc:Mmcs_0097 protein tyrosine/serine phosphatase; K01104 protein-tyrosine phosphatase [EC:3.1.3.48] (A)

MASAGDELSGAWNFRDVSSHTGVAPGVFFRASELSRLDDEGRAALSGYGVTDVADLRTLR

ELERHGPGRVPAGVDIHHLPFIETVASDAEAPHEHAFQRMMTDKPEGESIAAAAARYMTE

EYARIASAPLAQRAVRQVVTLLGSGRRVLAHCFAGKDRTGFTVAVVLEAAGVDREAVMAD

YLHSNTAVPQLRESILVTVRERAAETPEVLEMAEARLTESVLGVREEYLDAAFRTIAGEY

GSLEGYLSAAGVSADELARLRSTLRG

>mkm:Mkms_0106 protein tyrosine/serine phosphatase; K01104 protein-tyrosine phosphatase [EC:3.1.3.48] (A)

MASAGDELSGAWNFRDVSSHTGVAPGVFFRASELSRLDDEGRAALSGYGVTDVADLRTLR

ELERHGPGRVPAGVDIHHLPFIETVASDAEAPHEHAFQRMMTDKPEGESIAAAAARYMTE

EYARIASAPLAQRAVRQVVTLLGSGRRVLAHCFAGKDRTGFTVAVVLEAAGVDREAVMAD

YLHSNTAVPQLRESILVTVRERAAETPEVLEMAEARLTESVLGVREEYLDAAFRTIAGEY

GSLEGYLSAAGVSADELARLRSTLRG

>mjl:Mjls_0087 protein tyrosine/serine phosphatase; K01104 protein-tyrosine phosphatase [EC:3.1.3.48] (A)

MASAGDELSGAWNFRDVSSHTGVAPGVFFRASELSRLDDEGRAALSGYGVTDVADLRTLR

ELERHGPGRVPAGVDIHHLPFIETVASDAEAPHEHAFQRMMTDKPEGESIAAAAARYMTE

EYARIASAPLAQRAVRQVVTLLGSGRRVLAHCFAGKDRTGFTVAVVLEAAGVDREAVMAD

YLHSNTAVPQLRESILVTVRERAAETPEVLEMAEARLTESVLGVREEYLDAAFRTIAGEY

GSLEGYLSAAGVSADELARLRSTLRG

>mjd:JDM601_0097 ptpB; phosphotyrosine protein phosphatase PtpB; K01104 protein-tyrosine phosphatase [EC:3.1.3.48] (A)

MSYRGEVTSQPDLPGAWNFRDVSETTGALRPGRLFRSSELSRLDDRGREQLLRIGVADVA

DLRSPREVTRRGPGLVPDGVDIHLLPFPDLAADQAPESSSPHEDAFRKMMAEKPDADTAA

DVAARYMLEEYQRFPTLGGSRRAVQRVVSLLAAGRPVLAHCFAGKDRTGFVVAVVLQAAG

VDRDAVVADYLRSNTAVPVLRDRIMDMIRSRPEDEITPEVLTFTEARLSDEVLGVRADYL

EAAHRSIEQNFGSLDAFLHASGVTDADRDGLRAALVD

>mmi:MMAR_0373 ptpB; phosphotyrosine protein phosphatase PtpB; K01104 protein-tyrosine phosphatase [EC:3.1.3.48] (A)

MAETGPELPGAWNFRDVADSTAALRPGRLFRSSELSGLDDQGRAALRRFKITDVADLRSS

REVARRGPGQVPAGVDVHLLPFPDLADDQAGTDDNAPHETAFKRLLTDGDAGESSEAIDQ

VAARYMADEYRQFPMRTGAQRAVHRVFTLLAAGRPVLTHCFAGKDRTGFVVATVLEAVGV

DREVILADYLRSNDAVPQLRDRISEMIRERTDVELTPEVVTFTKARLSDGVLGVRPEYLA

AAWQTIDETFGSLDGYLADSGITEADRHQLRSALLG

>mrh:MycrhN_1611 protein tyrosine/serine phosphatase; K01104 protein-tyrosine phosphatase [EC:3.1.3.48] (A)

MAANPDALSGAWNFRDIAEEAGIRPGRFFRSSELSGLDDSGRAGLRGFGITDVADLRSER

EVERRGAGRVPGGVVIHRLPFHELSKPGAEAPHEQSFQRMMTEKPDDEDVAIAAGRFMTE

EYQNFPGLAGAQLAVRQVISLLAEGRPVITHCFAGKDRTGFTVATVLEAVGVDRDAIMSD

FLRSNAAVPQLRDSILTSIRSRAEDTTDEIVTFAEARLTEEVLGVREDYLVASRRSIDEQ

YGSLVKYLEAVGVTPDQLQRLRNSLLG

>mmm:W7S_24895 phosphotyrosine protein phosphatase ptpb; K01104 protein-tyrosine phosphatase [EC:3.1.3.48] (A)

MTEALRELSGAWNFRDVSDGAPALKPGRLFRSGELSGLDDDGRATLSRLGITDVADLRAA

REVARRGPGLVPDGVEVHLLPFPDLGEQDAGTDDAAPHEHAFQRLLTGEGAEEQSGQSVD

EAATRYMIDEYRQFPTRNGAQRALHRVISLLADGHSVLTHCFAGKDRTGFVVATVLEAIG

IDRDTILADFLRSNDAAPALRAQISAMIAQRQDAELTPEVVTWTEARLSDGVLGVREVYL

AAARQTIDEEFGSLDAYLRAASVSESDVERLREALLA

>mcb:Mycch_0100 protein tyrosine/serine phosphatase; K01104 protein-tyrosine phosphatase [EC:3.1.3.48] (A)

MASSGGELSGAWNFRDVAEQTGIAPGRFFRASELSKLDDHGRAALAGYGVTDVADLRTLR

ELERHGPGLVPTGVDIHHLPFIETTASDDEAPHEHAFQRMMTDKPDDESVADAAARYMTE

EYGRIATAPLAQRAVHQVVALLGSGRRVLAHCFAGKDRTGFTIAVVLEAAGVDRDAIMAD

YLRSNVAVPQLRESILANVRARAVEAPEVLELAEARLTESVLGVREDYLDMARRTLDAEF

GSLTGYLEAAGVTGEDLGRLRAALND

>mli:MULP_00359 ptpB; protein tyrosine phosphatase, PtpB (EC:3.1.3.48); K01104 protein-tyrosine phosphatase [EC:3.1.3.48] (A)

MAETGPELPGAWNFRDVADSTAALRPGRLFRSSELSGLDDQGRAAQRRFKITDVADLRSS

REVARRGPGQVPAGVDVHLLPFPDLADDQAGTDDNAPHETAFKRLLTDGDAGESSEAIDQ

VAARHMADEYRQFPMRTGAQRAVHRVFTLLAAGRPVLTHCFAGKDRTGFVVATVLEAVGV

DREVILADYLRSNDAVPQLRDRISEMIRERTDVELTPEVVTFTKARLSDGVLGVRPEYLA

AAWQTIDETFGSLDGYLADSGITEADRHQLRSALLG

>mkn:MKAN_16100 phosphotyrosine protein phosphatase; K01104 protein-tyrosine phosphatase [EC:3.1.3.48] (A)

MADLELPGAWNFRDVADSTALRPGRLFRSSELSRLDDAGRETLRRLAITDVADLRSSREV

VRRGPGRVPAGVDIHLLPFPDLADDDAGTDADAPHEHAFRRLMTSSPDGESIDQAAVRYM

TDEYRQFPTRTGAQRALHRVVTLLAGGRAVLTHCFAGKDRTGFVVATVLEAVGVDREAIL

TDFLRSNDAVPRLREQIAEMIQQRSEAELTPEVVTFTEARLSDGVLGVRPEYLAASWQTI

DETWGSVDAYLRNAGITPADVGRLRDGLLG

>mks:LG40_15930 phosphotyrosine protein phosphatase; K01104 protein-tyrosine phosphatase [EC:3.1.3.48] (A)

MADLELPGAWNFRDVADSTALRPGRLFRSSELSRLDDAGRETLRRLAITDVADLRSSREV

VRRGPGRVPAGVDIHLLPFPDLADDDAGTDADAPHEHAFRRLMTSSPDGESIDQAAVRYM

TDEYRQFPTRTGAQRALHRVVTLLAGGRAVLTHCFAGKDRTGFVVATVLEAVGVDREAIL

TDFLRSNDAVPRLREQIAEMIQQRSEAELTPEVVTFTEARLSDGVLGVRPEYLAASWQTI

DETWGSVDAYLRNAGITPADVGRLRDGLLG

>mki:LH54_16045 phosphotyrosine protein phosphatase; K01104 protein-tyrosine phosphatase [EC:3.1.3.48] (A)

MADLELPGAWNFRDVADSTALRPGRLFRSSELSRLDDAGRETLRRLAITDVADLRSSREV

VRRGPGRVPAGVDIHLLPFPDLADDDAGTDADAPHEHAFRRLMTSSPDGESIDQAAVRYM

TDEYRQFPTRTGAQRALHRVVTLLAGGRAVLTHCFAGKDRTGFVVATVLEAVGVDREAIL

TDFLRSNDAVPRLREQIAEMIQQRSEAELTPEVVTFTEARLSDGVLGVRPEYLAASWQTI

DETWGSVDAYLRNAGITPADVGRLRDGLLG

>mne:D174_01785 phosphotyrosine protein phosphatase; K01104 protein-tyrosine phosphatase [EC:3.1.3.48] (A)

MVELTGAWNFRDVAESAGIRPGLLFRSSELSKLTDEGRSTLLALGINDVADLRSRRELER

RGAGQVPADVALHHLPFHFEDDSAQDAPHEATFQRVMSESPEGEDVAEAAKRYMTEVYEE

FPALTGAQVAVRQVISMLADGRPVIAHCFAGKDRTGFTVATVLEAVGVPREAIMIDFLRS

NEAIDQLRDSIMESVKARAGEDPAASAFIEARLTDAVLGVREEYLEAAHRVRDERYGSVS

GFLQSAGITDDELTRLRSRLTN

>myv:G155_00430 phosphotyrosine protein phosphatase; K01104 protein-tyrosine phosphatase [EC:3.1.3.48] (A)

MTTELGELSGAWNFRDVADQTAIRPGLLYRSSQLSQLSDAGRAVFRRLGITDVADLRSHQ

EVQRQGTGQVPDGVAVHLLPFHPDNGATQDAPHESTFQRVMSESGEGEDITAAARRYMTE

VYEEFPTLPGAHSAVREVISLLAQERPVIAHCFAGKDRTGFTVATVLETVGVDRDRIIAD

FLASNDAIPALRNRIMESVRARSQEAPEMITYAEARLTDEVLGVREEYLDAAWRKLEADY

GSVAGFVEAAGVSPEDLAALRTNLVG

>mye:AB431_01380 phosphotyrosine protein phosphatase; K01104 protein-tyrosine phosphatase [EC:3.1.3.48] (A)

MSHTHLSGAWNFRDVADTTGIRPGKFFRSSELSRLDDDGRDAFRRLGITDVADLRSPQEL

ERRGQGAVPDGVAIHLLPFPDLSNTTADAPHETSWQKMMTEKVDDEDVEDAAERFMTGEY

EKFPVLAGAQRAVRQVFSLLSAGRPVITHCFAGKDRTGFTVAVVLESIGVPRDEVLTDFL

RSNDAVESLRERIMESIVSRAGETPEIATFAEARLTNGVLGVREGYLATAHRVIAENYGD

LDGFLRTAGVSEEDVARTRKELLG

>mgo:AFA91_07405 phosphotyrosine protein phosphatase; K01104 protein-tyrosine phosphatase [EC:3.1.3.48] (A)

MTAPDGTSLELGGAWNFRDVAQETGIRPGVLYRSSELSKLSDDGRAVFKRLGITDVADLR

SHQEVQRRGPGQVPDGVVVHLLPFHPDDVSGQDAPHETTFARVMSESPDDEDVTESARRY

MTEVYEEFPTLPGAHNAVRQVVSLLASGRPVIAHCFAGKDRTGFTVATVLDAAGVDRDDI

LADFLRSNEAIPSLRTRIMDSVRARSGDEPEIITFAEARLTDEVLGVREEYLAAAWKRLE

EAYGSLSGFITAAGVAPDQLDALRGALR

>mft:XA26_00890 Protein tyrosine phosphatase (EC:3.1.3.48); K01104 protein-tyrosine phosphatase [EC:3.1.3.48] (A)

MTTELGELSGAWNFRDVADQTAIRPGLLYRSSQLSQLSDAGRAVFRRLGITDVADLRSHQ

EVQRQGTGQVPDGVAVHLLPFHPDNGATQDAPHESTFQRVMSESGEGEDITAAARRYMTE

VYEEFPTLPGAHSAVREVISLLAQERPVIAHCFAGKDRTGFTVATVLETVGVDRDRIIAD

FLASNDAIPALRNRIMESVRARSQEAPEMITYAEARLTDEVLGVREEYLDAAWRKLEAAY

GSVAGFVEAAGVSPEDLAALRTNLAG

>mhad:B586_01615 phosphotyrosine protein phosphatase; K01104 protein-tyrosine phosphatase [EC:3.1.3.48] (A)

MAEAIRELPGAWNFRDVSDSTGALLRPGRLFRSSELSRLDDDGRATLRRLGITDVADLRA

AREVSRGGPGRVPDGIEIHRLPFPDIAAPKADDGAETDGAAPHERAFQQLLANAVAEDSD

QSINAAATRYMTDEYRQFPMRNGAQSALHRVITLLAAGRSVLTHCFAGKDRTGFVVATVL

ETIGIDRDSIVADFLRSNDAAPQLRARVSEMIQQRAEMTELTPEVVTFTAARLSDGVLGV

REEYLAAARQTIDDAYGSLDGYLRDAGITEADVDQLRSTLLS

Rv0202c Homologs

>mtu:Rv0202c mmpL11; transmembrane transport protein MmpL11; K20466 heme transporter (A)

MMRLSRNLRRCRWLVFTGWLLALVPAVYLAMTQSGNLTGGGFEVAGSQSLLVHDQLDAHY

PDRGAPALALVAAPRPDASYQDIDNAVALLRQIASELPGVTEAPNPTQRPPQPDRPYVVS

LRLDARNAGTSDVAKKLRDRIGVKGDQSGQTANGKVRLYVIGQGALSAAAAANTKHDIAN

AERWNLPIILMVLVAVFGSLAAAAIPLALAVCTVVITMGLVFVLSMHTTMSVFVTSTVSM

FGIALAVDYSLFILMRYREELRCGRRPPDAVDAAMATSGLAVVLSGMTVIASLTGIYLIN

TPALRSMATGAILAVAVAMLTSATLTPAVLATFARAAAKRSALVHWSRRPASTQSWFWSR

WVGWVMRRPWITALAASTVLLVMAAPATLMVLGNSLLRQFDSSHEIRTGAAAAAQALGPG

ALGPVQVLVRFDAGGASAPEHSQTIAAIRHRIAQAPNVVSVAPPRFADDNGSALLSAVLS

VDPEDLGARDTITWMRTQLPRVAGAAQVDVGGPTALIKDFDDRVSATQPLVLVFVAVIAF

LMLLISIRSVFLAFKGVLMTLLSVAAAYGSLVMVFQWGWARGLGFPALHSIDSTVPPLVL

AMTFGLSMDYEIFLLTRIRERFLQTGQTRDAVAYGVRTSARTITSAALIMIAVFCGFAFA

GMPLVAEIGVACAVAIAVDATVVRLVLVPALMAMFDRWNWWLPRWLAHILPSVDFDRPLP

KVDLGDVVVIPDDFAAAIPPSADVRMVLKSAAKLKRLAPDAICVTDPLAFTGCGCDGKAL

DQVQLAYRNGIARAISWGQRPVHPVTVWRKRLAVALDALQTTTWECGGVQTHRAGPGYRR

RSPVETTNVALPTGDRLQIPTGAETLRFKGYLIMSRNSSHDYADFADLVDTMAPETAAAV

LAGMDRYYSCQAPGRQWMATQLVGRLADPQPSDLGDQSPGADAQAKWEEVRRRCLSVAVA

MLEEAR

>mtv:RVBD_0202c transmembrane transporter MmpL11; K20466 heme transporter (A)

MMRLSRNLRRCRWLVFTGWLLALVPAVYLAMTQSGNLTGGGFEVAGSQSLLVHDQLDAHY

PDRGAPALALVAAPRPDASYQDIDNAVALLRQIASELPGVTEAPNPTQRPPQPDRPYVVS

LRLDARNAGTSDVAKKLRDRIGVKGDQSGQTANGKVRLYVIGQGALSAAAAANTKHDIAN

AERWNLPIILMVLVAVFGSLAAAAIPLALAVCTVVITMGLVFVLSMHTTMSVFVTSTVSM

FGIALAVDYSLFILMRYREELRCGRRPPDAVDAAMATSGLAVVLSGMTVIASLTGIYLIN

TPALRSMATGAILAVAVAMLTSATLTPAVLATFARAAAKRSALVHWSRRPASTQSWFWSR

WVGWVMRRPWITALAASTVLLVMAAPATLMVLGNSLLRQFDSSHEIRTGAAAAAQALGPG

ALGPVQVLVRFDAGGASAPEHSQTIAAIRHRIAQAPNVVSVAPPRFADDNGSALLSAVLS

VDPEDLGARDTITWMRTQLPRVAGAAQVDVGGPTALIKDFDDRVSATQPLVLVFVAVIAF

LMLLISIRSVFLAFKGVLMTLLSVAAAYGSLVMVFQWGWARGLGFPALHSIDSTVPPLVL

AMTFGLSMDYEIFLLTRIRERFLQTGQTRDAVAYGVRTSARTITSAALIMIAVFCGFAFA

GMPLVAEIGVACAVAIAVDATVVRLVLVPALMAMFDRWNWWLPRWLAHILPSVDFDRPLP

KVDLGDVVVIPDDFAAAIPPSADVRMVLKSAAKLKRLAPDAICVTDPLAFTGCGCDGKAL

DQVQLAYRNGIARAISWGQRPVHPVTVWRKRLAVALDALQTTTWECGGVQTHRAGPGYRR

RSPVETTNVALPTGDRLQIPTGAETLRFKGYLIMSRNSSHDYADFADLVDTMAPETAAAV

LAGMDRYYSCQAPGRQWMATQLVGRLADPQPSDLGDQSPGADAQAKWEEVRRRCLSVAVA

MLEEAR

>mtc:MT0212 membrane protein MmpL11; K20466 heme transporter (A)

MMRLSRNLRRCRWLVFTGWLLALVPAVYLAMTQSGNLTGGGFEVAGSQSLLVHDQLDAHY

PDRGAPALALVAAPRPDASYQDIDNAVALLRQIASELPGVTEAPNPTQRPPQPDRPYVVS

LRLDARNAGTSDVAKKLRDRIGVKGDQSGQTANGKVRLYVIGQGALSAAAAANTKHDIAN

AERWNLPIILMVLVAVFGSLAAAAIPLALAVCTVVITMGLVFVLSMHTTMSVFVTSTVSM

FGIALAVDYSLFILMRYREELRCGRRPPDAVDAAMATSGLAVVLSGMTVIASLTGIYLIN

TPALRSMATGAILAVAVAMLTSATLTPAVLATFARAAAKRSALVHWSRRPASTQSWFWSR

WVGWVMRRPWITALAASTVLLVMAAPATLMVLGNSLLRQFDSSHEIRTGAAAAAQALGPG

ALGPVQVLVRFDAGGASAPEHSQTIAAIRHRIAQAPNVVSVAPPRFADDNGSALLSAVLS

VDPEDLGARDTITWMRTQLPRVAGAAQVDVGGPTALIKDFDDRVSATQPLVLVFVAVIAF

LMLLISIRSVFLAFKGVLMTLLSVAAAYGSLVMVFQWGWARGLGFPALHSIDSTVPPLVL

AMTFGLSMDYEIFLLTRIRERFLQTGQTRDAVAYGVRTSARTITSAALIMIAVFCGFAFA

GMPLVAEIGVACAVAIAVDATVVRLVLVPALMAMFDRWNWWLPRWLAHILPSVDFDRPLP

KVDLGDVVVIPDDFAAAIPPSADVRMVLKSAAKLKRLAPDAICVTDPLAFTGCGCDGKAL

DQVQLAYRNGIARAISWGQRPVHPVTVWRKRLAVALDALQTTTWECGGVQTHRAGPGYRR

RSPVETTNVALPTGDRLQIPTGAETLRFKGYLIMSRNSSHDYADFADLVDTMAPETAAAV

LAGMDRYYSCQAPGRQWMATQLVGRLADPQPSDLGDQSPGADAQAKWEEVRRRCLSVAVA

MLEEAR

>mra:MRA_0210 mmpL11; membrane protein MmpL11; K20466 heme transporter (A)

MMRLSRNLRRCRWLVFTGWLLALVPAVYLAMTQSGNLTGGGFEVAGSQSLLVHDQLDAHY

PDRGAPALALVAAPRPDASYQDIDNAVALLRQIASELPGVTEAPNPTQRPPQPDRPYVVS

LRLDARNAGTSDVAKKLRDRIGVKGDQSGQTANGKVRLYVIGQGALSAAAAANTKHDIAN

AERWNLPIILMVLVAVFGSLAAAAIPLALAVCTVVITMGLVFVLSMHTTMSVFVTSTVSM

FGIALAVDYSLFILMRYREELRCGRRPPDAVDAAMATSGLAVVLSGMTVIASLTGIYLIN

TPALRSMATGAILAVAVAMLTSATLTPAVLATFARAAAKRSALVHWSRRPASTQSWFWSR

WVGWVMRRPWITALAASTVLLVMAAPATLMVLGNSLLRQFDSSHEIRTGAAAAAQALGPG

ALGPVQVLVRFDAGGASAPEHSQTIAAIRHRIAQAPNVVSVAPPRFADDNGSALLSAVLS

VDPEDLGARDTITWMRTQLPRVAGAAQVDVGGPTALIKDFDDRVSATQPLVLVFVAVIAF

LMLLISIRSVFLAFKGVLMTLLSVAAAYGSLVMVFQWGWARGLGFPALHSIDSTVPPLVL

AMTFGLSMDYEIFLLTRIRERFLQTGQTRDAVAYGVRTSARTITSAALIMIAVFCGFAFA

GMPLVAEIGVACAVAIAVDATVVRLVLVPALMAMFDRWNWWLPRWLAHILPSVDFDRPLP

KVDLGDVVVIPDDFAAAIPPSADVRMVLKSAAKLKRLAPDAICVTDPLAFTGCGCDGKAL

DQVQLAYRNGIARAISWGQRPVHPVTVWRKRLAVALDALQTTTWECGGVQTHRAGPGYRR

RSPVETTNVALPTGDRLQIPTGAETLRFKGYLIMSRNSSHDYADFADLVDTMAPETAAAV

LAGMDRYYSCQAPGRQWMATQLVGRLADPQPSDLGDQSPGADAQAKWEEVRRRCLSVAVA

MLEEAR

>mtf:TBFG_10204 transmembrane transport protein mmpL11; K20466 heme transporter (A)

MMRLSRNLRRCRWLVFTGWLLALVPAVYLAMTQSGNLTGGGFEVAGSQSLLVHDQLDAHY

PDRGAPALALVAAPRPDASYQDIDNAVALLRQIASELPGVTEAPNPTQRPPQPDRPYVVS

LRLDARNAGTSDVAKKLRDRIGVKGDQSGQTANGKVRLYVIGQGALSAAAAANTKHDIAN

AERWNLPIILMVLVAVFGSLAAAAIPLALAVCTVVITMGLVFVLSMHTTMSVFVTSTVSM

FGIALAVDYSLFILMRYREELRCGRRPPDAVDAAMATSGLAVVLSGMTVIASLTGIYLIN

TPALRSMATGAILAVAVAMLTSATLTPAVLATFARAAAKRSALVHWSRRPASTQSWFWSR

WVGWVMRRPWITALAASTVLLVMAAPATLMVLGNSLLRQFDSSHEIRTGAAAAAQALGPG

ALGPVQVLVRFDAGGASAPEHSQTIAAIRHRIAQAPNVVSVAPPRFADDNGSALLSAVLS

VDPEDLGARDTITWMRTQLPRVAGAAQVDVGGPTALIKDFDDRVSATQPLVLVFVAVIAF

LMLLISIRSVFLAFKGVLMTLLSVAAAYGSLVMVFQWGWARGLGFPALHSIDSTVPPLVL

AMTFGLSMDYEIFLLTRIRERFLQTGQTRDAVAYGVRTSARTITSAALIMIAVFCGFAFA

GMPLVAEIGVACAVAIAVDATVVRLVLVPALMAMFDRWNWWLPRWLAHILPSVDFDRPLP

KVDLGDVVVIPDDFAAAIPPSADVRMVLKSAAKLKRLAPDAICVTDPLAFTGCGCDGKAL

DQVQLAYRNGIARAISWGQRPVHPVTVWRKRLAVALDALQTTTWECGGVQTHRAGPGYRR

RSPVETTNVALPTGDRLQIPTGAETLRFKGYLIMSRNSSHDYADFADLVDTMAPETAAAV

LAGMDRYYSCQAPGRQWMATQLVGRLADPQPSDLGDQSPGADAQAKWEEVRRRCLSVAVA

MLEEAR

>mtb:TBMG_00203 transmembrane transporter mmpL11; K20466 heme transporter (A)

MMRLSRNLRRCRWLVFTGWLLALVPAVYLAMTQSGNLTGGGFEVAGSQSLLVHDQLDAHY

PDRGAPALALVAAPRPDASYQDIDNAVALLRQIASELPGVTEAPNPTQRPPQPDRPYVVS

LRLDARNAGTSDVAKKLRDRIGVKGDQSGQTANGKVRLYVIGQGALSAAAAANTKHDIAN

AERWNLPIILMVLVAVFGSLAAAAIPLALAVCTVVITMGLVFVLSMHTTMSVFVTSTVSM

FGIALAVDYSLFILMRYREELRCGRRPPDAVDAAMATSGLAVVLSGMTVIASLTGIYLIN

TPALRSMATGAILAVAVAMLTSATLTPAVLATFARAAAKRSALVHWSRRPASTQSWFWSR

WVGWVMRRPWITALAASTVLLVMAAPATLMVLGNSLLRQFDSSHEIRTGAAAAAQALGPG

ALGPVQVLVRFDAGGASAPEHSQTIAAIRHRIAQAPNVVSVAPPRFADDNGSALLSAVLS

VDPEDLGARDTITWMRTQLPRVAGAAQVDVGGPTALIKDFDDRVSATQPLVLVFVAVIAF

LMLLISIRSVFLAFKGVLMTLLSVAAAYGSLVMVFQWGWARGLGFPALHSIDSTVPPLVL

AMTFGLSMDYEIFLLTRIRERFLQTGQTRDAVAYGVRTSARTITSAALIMIAVFCGFAFA

GMPLVAEIGVACAVAIAVDATVVRLVLVPALMAMFDRWNWWLPRWLAHILPSVDFDRPLP

KVDLGDVVVIPDDFAAAIPPSADVRMVLKSAAKLKRLAPDAICVTDPLAFTGCGCDGKAL

DQVQLAYRNGIARAISWGQRPVHPVTVWRKRLAVALDALQTTTWECGGVQTHRAGPGYRR

RSPVETTNVALPTGDRLQIPTGAETLRFKGYLIMSRNSSHDYADFADLVDTMAPETAAAV

LAGMDRYYSCQAPGRQWMATQLVGRLADPQPSDLGDQSPGADAQAKWEEVRRRCLSVAVA

MLEEAR

>mtk:TBSG_00205 transmembrane transporter mmpL11; K20466 heme transporter (A)

MMRLSRNLRRCRWLVFTGWLLALVPAVYLAMTQSGNLTGGGFEVAGSQSLLVHDQLDAHY

PDRGAPALALVAAPRPDASYQDIDNAVALLRQIASELPGVTEAPNPTQRPPQPDRPYVVS

LRLDARNAGTSDVAKKLRDRIGVKGDQSGQTANGKVRLYVIGQGALSAAAAANTKHDIAN

AERWNLPIILMVLVAVFGSLAAAAIPLALAVCTVVITMGLVFVLSMHTTMSVFVTSTVSM

FGIALAVDYSLFILMRYREELRCGRRPPDAVDAAMATSGLAVVLSGMTVIASLTGIYLIN

TPALRSMATGAILAVAVAMLTSATLTPAVLATFARAAAKRSALVHWSRRPASTQSWFWSR

WVGWVMRRPWITALAASTVLLVMAAPATLMVLGNSLLRQFDSSHEIRTGAAAAAQALGPG

ALGPVQVLVRFDAGGASAPEHSQTIAAIRHRIAQAPNVVSVAPPRFADDNGSALLSAVLS

VDPEDLGARDTITWMRTQLPRVAGAAQVDVGGPTALIKDFDDRVSATQPLVLVFVAVIAF

LMLLISIRSVFLAFKGVLMTLLSVAAAYGSLVMVFQWGWARGLGFPALHSIDSTVPPLVL

AMTFGLSMDYEIFLLTRIRERFLQTGQTRDAVAYGVRTSARTITSAALIMIAVFCGFAFA

GMPLVAEIGVACAVAIAVDATVVRLVLVPALMAMFDRWNWWLPRWLAHILPSVDFDRPLP

KVDLGDVVVIPDDFAAAIPPSADVRMVLKSAAKLKRLAPDAICVTDPLAFTGCGCDGKAL

DQVQLAYRNGIARAISWGQRPVHPVTVWRKRLAVALDALQTTTWECGGVQTHRAGPGYRR

RSPVETTNVALPTGDRLQIPTGAETLRFKGYLIMSRNSSHDYADFADLVDTMAPETAAAV

LAGMDRYYSCQAPGRQWMATQLVGRLADPQPSDLGDQSPGADAQAKWEEVRRRCLSVAVA

MLEEAR

>mtz:TBXG_000204 transmembrane transporter mmpL11; K20466 heme transporter (A)

MMRLSRNLRRCRWLVFTGWLLALVPAVYLAMTQSGNLTGGGFEVAGSQSLLVHDQLDAHY

PDRGAPALALVAAPRPDASYQDIDNAVALLRQIASELPGVTEAPNPTQRPPQPDRPYVVS

LRLDARNAGTSDVAKKLRDRIGVKGDQSGQTANGKVRLYVIGQGALSAAAAANTKHDIAN

AERWNLPIILMVLVAVFGSLAAAAIPLALAVCTVVITMGLVFVLSMHTTMSVFVTSTVSM

FGIALAVDYSLFILMRYREELRCGRRPPDAVDAAMATSGLAVVLSGMTVIASLTGIYLIN

TPALRSMATGAILAVAVAMLTSATLTPAVLATFARAAAKRSALVHWSRRPASTQSWFWSR

WVGWVMRRPWITALAASTVLLVMAAPATLMVLGNSLLRQFDSSHEIRTGAAAAAQALGPG

ALGPVQVLVRFDAGGASAPEHSQTIAAIRHRIAQAPNVVSVAPPRFADDNGSALLSAVLS

VDPEDLGARDTITWMRTQLPRVAGAAQVDVGGPTALIKDFDDRVSATQPLVLVFVAVIAF

LMLLISIRSVFLAFKGVLMTLLSVAAAYGSLVMVFQWGWARGLGFPALHSIDSTVPPLVL

AMTFGLSMDYEIFLLTRIRERFLQTGQTRDAVAYGVRTSARTITSAALIMIAVFCGFAFA

GMPLVAEIGVACAVAIAVDATVVRLVLVPALMAMFDRWNWWLPRWLAHILPSVDFDRPLP

KVDLGDVVVIPDDFAAAIPPSADVRMVLKSAAKLKRLAPDAICVTDPLAFTGCGCDGKAL

DQVQLAYRNGIARAISWGQRPVHPVTVWRKRLAVALDALQTTTWECGGVQTHRAGPGYRR

RSPVETTNVALPTGDRLQIPTGAETLRFKGYLIMSRNSSHDYADFADLVDTMAPETAAAV

LAGMDRYYSCQAPGRQWMATQLVGRLADPQPSDLGDQSPGADAQAKWEEVRRRCLSVAVA

MLEEAR

>mtg:MRGA327_01275 hypothetical protein; K20466 heme transporter (A)

MMRLSRNLRRCRWLVFTGWLLALVPAVYLAMTQSGNLTGGGFEVAGSQSLLVHDQLDAHY

PDRGAPALALVAAPRPDASYQDIDNAVALLRQIASELPGVTEAPNPTQRPPQPDRPYVVS

LRLDARNAGTSDVAKKLRDRIGVKGDQSGQTANGKVRLYVIGQGALSAAAAANTKHDIAN

AERWNLPIILMVLVAVFGSLAAAAIPLALAVCTVVITMGLVFVLSMHTTMSVFVTSTVSM

FGIALAVDYSLFILMRYREELRCGRRPPDAVDAAMATSGLAVVLSGMTVIASLTGIYLIN

TPALRSMATGAILAVAVAMLTSATLTPAVLATFARAAAKRSALVHWSRRPASTQSWFWSR

WVGWVMRRPWITALAASTVLLVMAAPATLMVLGNSLLRQFDSSHEIRTGAAAAAQALGPG

ALGPVQVLVRFDAGGASAPEHSQTIAAIRHRIAQAPNVVSVAPPRFADDNGSALLSAVLS

VDPEDLGARDTITWMRTQLPRVAGAAQVDVGGPTALIKDFDDRVSATQPLVLVFVAVIAF

LMLLISIRSVFLAFKGVLMTLLSVAAAYGSLVMVFQWGWARGLGFPALHSIDSTVPPLVL

AMTFGLSMDYEIFLLTRIRERFLQTGQTRDAVAYGVRTSARTITSAALIMIAVFCGFAFA

GMPLVAEIGVACAVAIAVDATVVRLVLVPALMAMFDRWNWWLPRWLAHILPSVDFDRPLP

KVDLGDVVVIPDDFAAAIPPSADVRMVLKSAAKLKRLAPDAICVTDPLAFTGCGCDGKAL

DQVQLAYRNGIARAISWGQRPVHPVTVWRKRLAVALDALQTTTWECGGVQTHRAGPGYRR

RSPVETTNVALPTGDRLQIPTGAETLRFKGYLIMSRNSSHDYADFADLVDTMAPETAAAV

LAGMDRYYSCQAPGRQWMATPVGWSPGRSATV

>mti:MRGA423_07150 transmembrane transport protein; K06994 putative drug exporter of the RND superfamily (A)

MLQRIARLAIAAPRRIIGFAVFVFIAAAVFGVPVADSLSPGGFQDPRSESARAIEVLTDK

FGQSGQKMLIVVTAAAGADSPPAREVGTDIVEVLRRSPLVYNVTSPWTVPPTAAADLLST

DGKSGLIVVNVKGGENDAQNHAQTLSDEVAHDRDGVTVRAGGSAMEYAQINRQNKDDLLV

MELIAIPLSFLVLIWVFGGLLAAGLPMAQAVLAVVGSMAVLRLVTFATEVSTFALNLSTA

LGLALAIDYTLLIVSRYRDELAEGSDRDEALIRTMATSGRTVLFSAVTVALSMSATALFP

MYFLKSFAYAGVATVAFVATASIVITPAAIVLLGPRLDALDVRRLVRRLLGRPDPVHKPV

KQLFWYRSSKFVMRRWLPVGTAVVALLVLLGLPFLSVKWGFPDDRVLPRSASARQVGDIL

RDDFGHDPATQIPIVVPDARGLGPVELDSYAAELSRVPDVSAVAAPTGTFVDGSWVGTPR

GATGLAEGSAFLTVSSTAPLFSRASDIQLKRLHQVAGPAGRSVVMAGVAQVNRDSVDAVT

DRLPMVLGLIAAITYVLLFLLTGSVVLPAKALVCNVLSLTAAFGALVWIFQEGHFGALGT

TPSGTLVANMPVLLFCIAFGLSMDYEVFLVSRIREYWLESGAARPARRSVAEVHAANDES

VALGVARTGRVITAAALVMSMSFAALIAAHVSFMRMFGLGLTLAVAADATLVRMVVVPAF

MHVTGRWNWWAPRPLAWLHERFGVSEAAEPVSRRRSHAGGLGKIAGRSDGQTIPASLTRN

G

>mte:CCDC5079_0187 transmembrane transport protein MmpL11; K20466 heme transporter (A)

MMRLSRNLRRCRWLVFTGWLLALVPAVYLAMTQSGNLTGGGFEVAGSQSLLVHDQLDAHY

PDRGAPALALVAAPRPDASYQDIDNAVALLRQIASELPGVTEAPNPTQRPPQPDRPYVVS

LRLDARNAGTSDVAKKLRDRIGVKGDQSGQTANGKVRLYVIGQGALSAAAAANTKHDIAN

AERWNLPIILMVLVAVFGSLAAAAIPLALAVCTVVITMGLVFVLSMHTTMSVFVTSTVSM

FGIALAVDYSLFILMRYREELRCGRRPPDAVDAAMATSGLAVVLSGMTVIASLTGIYLIN

TPALRSMATGAILAVAVAMLTSATLTPAVLATFARAAAKRSALVHWSRRPASTQSWFWSR

WVGWVMRRPWITALAASTVLLVMAAPATLMVLGNSLLRQFDSSHEIRTGAAAAAQALGPG

ALGPVQVLVRFDAGGASAPEHSQTIAAIRHRIAQAPNVVSVAPPRFADDNGSALLSAVLS

VDPEDLGARDTITWMRTQLPRVAGAAQVDVGGPTALIKDFDDRVSATQPLVLVFVAVIAF

LMLLISIRSVFLAFKGVLMTLLSVAAAYGSLVMVFQWGWARGLGFPALHSIDSTVPPLVL

AMTFGLSMDYEIFLLTRIRERFLQTGQTRDAVAYGVRTSARTITSAALIMIAVFCGFAFA

GMPLVAEIGVACAVAIAVDATVVRLVLVPALMAMFDRWNWWLPRWLAHILPSVDFDRPLP

KVDLGDVVVIPDDFAAAIPPSADVRMVLKSAAKLKRLAPDAICVTDPLAFTGCGCDGKAL

DQVQLAYRNGIARAISWGQRPVHPVTVWRKRLAVALDALQTTTWECGGVQTHRAGPGYRR

RSPVETTNVALPTGDRLQIPTGAETLRFKGYLIMSRNSSHDYADFADLVDTMAPETAAAV

LAGMDRYYSCQAPGRQWMATQLVGRLADPQPSDLGDQSPGADAQAKWEEVRRRCLSVAVA

MLEEAR

>mtur:CFBS_0218 mmpL11; transmembrane transporter MmpL11; K20466 heme transporter (A)

MMRLSRNLRRCRWLVFTGWLLALVPAVYLAMTQSGNLTGGGFEVAGSQSLLVHDQLDAHY

PDRGAPALALVAAPRPDASYQDIDNAVALLRQIASELPGVTEAPNPTQRPPQPDRPYVVS

LRLDARNAGTSDVAKKLRDRIGVKGDQSGQTANGKVRLYVIGQGALSAAAAANTKHDIAN

AERWNLPIILMVLVAVFGSLAAAAIPLALAVCTVVITMGLVFVLSMHTTMSVFVTSTVSM

FGIALAVDYSLFILMRYREELRCGRRPPDAVDAAMATSGLAVVLSGMTVIASLTGIYLIN

TPALRSMATGAILAVAVAMLTSATLTPAVLATFARAAAKRSALVHWSRRPASTQSWFWSR

WVGWVMRRPWITALAASTVLLVMAAPATLMVLGNSLLRQFDSSHEIRTGAAAAAQALGPG

ALGPVQVLVRFDAGGASAPEHSQTIAAIRHRIAQAPNVVSVAPPRFADDNGSALLSAVLS

VDPEDLGARDTITWMRTQLPRVAGAAQVDVGGPTALIKDFDDRVSATQPLVLVFVAVIAF

LMLLISIRSVFLAFKGVLMTLLSVAAAYGSLVMVFQWGWARGLGFPALHSIDSTVPPLVL

AMTFGLSMDYEIFLLTRIRERFLQTGQTRDAVAYGVRTSARTITSAALIMIAVFCGFAFA

GMPLVAEIGVACAVAIAVDATVVRLVLVPALMAMFDRWNWWLPRWLAHILPSVDFDRPLP

KVDLGDVVVIPDDFAAAIPPSADVRMVLKSAAKLKRLAPDAICVTDPLAFTGCGCDGKAL

DQVQLAYRNGIARAISWGQRPVHPVTVWRKRLAVALDALQTTTWECGGVQTHRAGPGYRR

RSPVETTNVALPTGDRLQIPTGAETLRFKGYLIMSRNSSHDYADFADLVDTMAPETAAAV

LAGMDRYYSCQAPGRQWMATQLVGRLADPQPSDLGDQSPGADAQAKWEEVRRRCLSVAVA

MLEEAR

>mtl:CCDC5180_0185 transmembrane transport protein MmpL11; K20466 heme transporter (A)

MMRLSRNLRRCRWLVFTGWLLALVPAVYLAMTQSGNLTGGGFEVAGSQSLLVHDQLDAHY

PDRGAPALALVAAPRPDASYQDIDNAVALLRQIASELPGVTEAPNPTQRPPQPDRPYVVS

LRLDARNAGTSDVAKKLRDRIGVKGDQSGQTANGKVRLYVIGQGALSAAAAANTKHDIAN

AERWNLPIILMVLVAVFGSLAAAAIPLALAVCTVVITMGLVFVLSMHTTMSVFVTSTVSM

FGIALAVDYSLFILMRYREELRCGRRPPDAVDAAMATSGLAVVLSGMTVIASLTGIYLIN

TPALRSMATGAILAVAVAMLTSATLTPAVLATFARAAAKRSALVHWSRRPASTQSWFWSR

WVGWVMRRPWITALAASTVLLVMAAPATLMVLGNSLLRQFDSSHEIRTGAAAAAQALGPG

ALGPVQVLVRFDAGGASAPEHSQTIAAIRHRIAQAPNVVSVAPPRFADDNGSALLSAVLS

VDPEDLGARDTITWMRTQLPRVAGAAQVDVGGPTALIKDFDDRVSATQPLVLVFVAVIAF

LMLLISIRSVFLAFKGVLMTLLSVAAAYGSLVMVFQWGWARGLGFPALHSIDSTVPPLVL

AMTFGLSMDYEIFLLTRIRERFLQTGQTRDAVAYGVRTSARTITSAALIMIAVFCGFAFA

GMPLVAEIGVACAVAIAVDATVVRLVLVPALMAMFDRWNWWLPRWLAHILPSVDFDRPLP

KVDLGDVVVIPDDFAAAIPPSADVRMVLKSAAKLKRLAPDAICVTDPLAFTGCGCDGKAL

DQVQLAYRNGIARAISWGQRPVHPVTVWRKRLAVALDALQTTTWECGGVQTHRAGPGYRR

RSPVETTNVALPTGDRLQIPTGAETLRFKGYLIMSRNSSHDYADFADLVDTMAPETAAAV

LAGMDRYYSCQAPGRQWMATQLVGRLADPQPSDLGDQSPGADAQAKWEEVRRRCLSVAVA

MLEEAR

>mto:MTCTRI2_0206 mmpL11; transmembrane transport protein MmpL11; K20466 heme transporter (A)

MMRLSRNLRRCRWLVFTGWLLALVPAVYLAMTQSGNLTGGGFEVAGSQSLLVHDQLDAHY

PDRGAPALALVAAPRPDASYQDIDNAVALLRQIASELPGVTEAPNPTQRPPQPDRPYVVS

LRLDARNAGTSDVAKKLRDRIGVKGDQSGQTANGKVRLYVIGQGALSAAAAANTKHDIAN

AERWNLPIILMVLVAVFGSLAAAAIPLALAVCTVVITMGLVFVLSMHTTMSVFVTSTVSM

FGIALAVDYSLFILMRYREELRCGRRPPDAVDAAMATSGLAVVLSGMTVIASLTGIYLIN

TPALRSMATGAILAVAVAMLTSATLTPAVLATFARAAAKRSALVHWSRRPASTQSWFWSR

WVGWVMRRPWITALAASTVLLVMAAPATLMVLGNSLLRQFDSSHEIRTGAAAAAQALGPG

ALGPVQVLVRFDAGGASAPEHSQTIAAIRHRIAQAPNVVSVAPPRFADDNGSALLSAVLS

VDPEDLGARDTITWMRTQLPRVAGAAQVDVGGPTALIKDFDDRVSATQPLVLVFVAVIAF

LMLLISIRSVFLAFKGVLMTLLSVAAAYGSLVMVFQWGWARGLGFPALHSIDSTVPPLVL

AMTFGLSMDYEIFLLTRIRERFLQTGQTRDAVAYGVRTSARTITSAALIMIAVFCGFAFA

GMPLVAEIGVACAVAIAVDATVVRLVLVPALMAMFDRWNWWLPRWLAHILPSVDFDRPLP

KVDLGDVVVIPDDFAAAIPPSADVQMVLKSAAKLKRLAPDAICVTDPLAFTGCGCDGKAL

DQVQLAYRNGIARAISWGQRPVHPVTVWRKRLAVALDALQTTTWECGGVQTHRAGPGYRR

RSPVETTNVALPTGDRLQIPTGAETLRFKGYLIMSRNSSHDYADFADLVDTMAPETAAAV

LAGMDRYYSCQAPGRQWMATQLVGRLADPQPSDLGDQSPGADAQAKWEEVRRRCLSVAVA

MLEEAR

>mtd:UDA_0202c mmpL11; mmpL11; K20466 heme transporter (A)

MMRLSRNLRRCRWLVFTGWLLALVPAVYLAMTQSGNLTGGGFEVAGSQSLLVHDQLDAHY

PDRGAPALALVAAPRPDASYQDIDNAVALLRQIASELPGVTEAPNPTQRPPQPDRPYVVS

LRLDARNAGTSDVAKKLRDRIGVKGDQSGQTANGKVRLYVIGQGALSAAAAANTKHDIAN

AERWNLPIILMVLVAVFGSLAAAAIPLALAVCTVVITMGLVFVLSMHTTMSVFVTSTVSM

FGIALAVDYSLFILMRYREELRCGRRPPDAVDAAMATSGLAVVLSGMTVIASLTGIYLIN

TPALRSMATGAILAVAVAMLTSATLTPAVLATFARAAAKRSALVHWSRRPASTQSWFWSR

WVGWVMRRPWITALAASTVLLVMAAPATLMVLGNSLLRQFDSSHEIRTGAAAAAQALGPG

ALGPVQVLVRFDAGGASAPEHSQTIAAIRHRIAQAPNVVSVAPPRFADDNGSALLSAVLS

VDPEDLGARDTITWMRTQLPRVAGAAQVDVGGPTALIKDFDDRVSATQPLVLVFVAVIAF

LMLLISIRSVFLAFKGVLMTLLSVAAAYGSLVMVFQWGWARGLGFPALHSIDSTVPPLVL

AMTFGLSMDYEIFLLTRIRERFLQTGQTRDAVAYGVRTSARTITSAALIMIAVFCGFAFA

GMPLVAEIGVACAVAIAVDATVVRLVLVPALMAMFDRWNWWLPRWLAHILPSVDFDRPLP

KVDLGDVVVIPDDFAAAIPPSADVRMVLKSAAKLKRLAPDAICVTDPLAFTGCGCDGKAL

DQVQLAYRNGIARAISWGQRPVHPVTVWRKRLAVALDALQTTTWECGGVQTHRAGPGYRR

RSPVETTNVALPTGDRLQIPTGAETLRFKGYLIMSRNSSHDYADFADLVDTMAPETAAAV

LAGMDRYYSCQAPGRQWMATQLVGRLADPQPSDLGDQSPGADAQAKWEEVRRRCLSVAVA

MLEEAR

>mtn:ERDMAN_0229 mmpL11; transmembrane transport protein; K20466 heme transporter (A)

MMRLSRNLRRCRWLVFTGWLLALVPAVYLAMTQSGNLTGGGFEVAGSQSLLVHDQLDAHY

PDRGAPALALVAAPRPDASYQDIDNAVALLRQIASELPGVTEAPNPTQRPPQPDRPYVVS

LRLDARNAGTSDVAKKLRDRIGVKGDQSGQTANGKVRLYVIGQGALSAAAAANTKHDIAN

AERWNLPIILMVLVAVFGSLAAAAIPLALAVCTVVITMGLVFVLSMHTTMSVFVTSTVSM

FGIALAVDYSLFILMRYREELRCGRRPPDAVDAAMATSGLAVVLSGMTVIASLTGIYLIN

TPALRSMATGAILAVAVAMLTSATLTPAVLATFARAAAKRSALVHWSRRPASTQSWFWSR

WVGWVMRRPWITALAASTVLLVMAAPATLMVLGNSLLRQFDSSHEIRTGAAAAAQALGPG

ALGPVQVLVRFDAGGASAPEHSQTIAAIRHRIAQAPNVVSVAPPRFADDNGSALLSAVLS

VDPEDLGARDTITWMRTQLPRVAGAAQVDVGGPTALIKDFDDRVSATQPLVLVFVAVIAF

LMLLISIRSVFLAFKGVLMTLLSVAAAYGSLVMVFQWGWARGLGFPALHSIDSTVPPLVL

AMTFGLSMDYEIFLLTRIRERFLQTGQTRDAVAYGVRTSARTITSAALIMIAVFCGFAFA

GMPLVAEIGVACAVAIAVDATVVRLVLVPALMAMFDRWNWWLPRWLAHILPSVDFDRPLP

KVDLGDVVVIPDDFAAAIPPSADVRMVLKSAAKLKRLAPDAICVTDPLAFTGCGCDGKAL

DQVQLAYRNGIARAISWGQRPVHPVTVWRKRLAVALDALQTTTWECGGVQTHRAGPGYRR

RSPVETTNVALPTGDRLQIPTGAETLRFKGYLIMSRNSSHDYADFADLVDTMAPETAAAV

LAGMDRYYSCQAPGRQWMATQLVGRLADPQPSDLGDQSPGADAQAKWEEVRRRCLSVAVA

MLEEAR

>mtj:J112_01110 transmembrane transport protein; K20466 heme transporter (A)

MMRLSRNLRRCRWLVFTGWLLALVPAVYLAMTQSGNLTGGGFEVAGSQSLLVHDQLDAHY

PDRGAPALALVAAPRPDASYQDIDNAVALLRQIASELPGVTEAPNPTQRPPQPDRPYVVS

LRLDARNAGTSDVAKKLRDRIGVKGDQSGQTANGKVRLYVIGQGALSAAAAANTKHDIAN

AERWNLPIILMVLVAVFGSLAAAAIPLALAVCTVVITMGLVFVLSMHTTMSVFVTSTVSM

FGIALAVDYSLFILMRYREELRCGRRPPDAVDAAMATSGLAVVLSGMTVIASLTGIYLIN

TPALRSMATGAILAVAVAMLTSATLTPAVLATFARAAAKRSALVHWSRRPASTQSWFWSR

WVGWVMRRPWITALAASTVLLVMAAPATLMVLGNSLLRQFDSSHEIRTGAAAAAQALGPG

ALGPVQVLVRFDAGGASAPEHSQTIAAIRHRIAQAPNVVSVAPPRFADDNGSALLSAVLS

VDPEDLGARDTITWMRTQLPRVAGAAQVDVGGPTALIKDFDDRVSATQPLVLVFVAVIAF

LMLLISIRSVFLAFKGVLMTLLSVAAAYGSLVMVFQWGWARGLGFPALHSIDSTVPPLVL

AMTFGLSMDYEIFLLTRIRERFLQTGQTRDAVAYGVRTSARTITSAALIMIAVFCGFAFA

GMPLVAEIGVACAVAIAVDATVVRLVLVPALMAMFDRWNWWLPRWLAHILPSVDFDRPLP

KVDLGDVVVIPDDFAAAIPPSADVRMVLKSAAKLKRLAPDAICVTDPLAFTGCGCDGKAL

DQVQLAYRNGIARAISWGQRPVHPVTVWRKRLAVALDALQTTTWECGGVQTHRAGPGYRR

RSPVETTNVALPTGDRLQIPTGAETLRFKGYLIMSRNSSHDYADFADLVDTMAPETAAAV

LAGMDRYYSCQAPGRQWMATQLVGRLADPQPSDLGDQSPGADAQAKWEEVRRRCLSVAVA

MLEEAR

>mtub:MT7199_0206 putative conserved protein TRANSPORT protein MMPL11; K20466 heme transporter (A)

MMRLSRNLRRCRWLVFTGWLLALVPAVYLAMTQSGNLTGGGFEVAGSQSLLVHDQLDAHY

PDRGAPALALVAAPRPDASYQDIDNAVALLRQIASELPGVTEAPNPTQRPPQPDRPYVVS

LRLDARNAGTSDVAKKLRDRIGVKGDQSGQTANGKVRLYVIGQGALSAAAAANTKHDIAN

AERWNLPIILMVLVAVFGSLAAAAIPLALAVCTVVITMGLVFVLSMHTTMSVFVTSTVSM

FGIALAVDYSLFILMRYREELRCGRRPPDAVDAAMATSGLAVVLSGMTVIASLTGIYLIN

TPALRSMATGAILAVAVAMLTSATLTPAVLATFARAAAKRSALVHWSRRPASTQSWFWSR

WVGWVMRRPWITALAASTVLLVMAAPATLMVLGNSLLRQFDSSHEIRTGAAAAAQALGPG

ALGPVQVLVRFDAGGASAPEHSQTIAAIRHRIAQAPNVVSVAPPRFADDNGSALLSAVLS

VDPEDLGARDTITWMRTQLPRVAGAAQVDVGGPTALIKDFDDRVSATQPLVLVFVAVIAF

LMLLISIRSVFLAFKGVLMTLLSVAAAYGSLVMVFQWGWARGLGFPALHSIDSTVPPLVL

AMTFGLSMDYEIFLLTRIRERFLQTGQTRDAVAYGVRTSARTITSAALIMIAVFCGFAFA

GMPLVAEIGVACAVAIAVDATVVRLVLVPALMAMFDRWNWWLPRWLAHILPSVDFDRPLP

KVDLGDVVVIPDDFAAAIPPSADVRMVLKSAAKLKRLAPDAICVTDPLAFTGCGCDGKAL

DQVQLAYRNGIARAISWGQRPVHPVTVWRKRLAVALDALQTTTWECGGVQTHRAGPGYRR

RSPVETTNVALPTGDRLQIPTGAETLRFKGYLIMSRNSSHDYADFADLVDTMAPETAAAV

LAGMDRYYSCQAPGRQWMATQLVGRLADPQPSDLGDQSPGADAQAKWEEVRRRCLSVAVA

MLEEAR

>mtuc:J113_08025 transmembrane transport protein; K06994 putative drug exporter of the RND superfamily (A)

MLQRIARLAIAAPRRIIGFAVFVFIAAAVFGVPVADSLSPGGFQDPRSESARAIEVLTDK

FGQSGQKMLIVVTAAAGADSPPAREVGTDIVEVLRRSPLVYNVTSPWTVPPTAAADLLST

DGKSGLIVVNVKGGENDAQNHAQTLSDEVAHDRDGVTVRAGGSAMEYAQINRQNKDDLLV

MELIAIPLSFLVLIWVFGGLLAAGLPMAQAVLAVVGSMAVLRLVTFATEVSTFALNLSTA

LGLALAIDYTLLIVSRYRDELAEGSDRDEALIRTMATSGRTVLFSAVTVALSMSATALFP

MYFLKSFAYAGVATVAFVATASIVITPAAIVLLGPRLDALDVRRLVRRLLGRPDPVHKPV

KQLFWYRSSKFVMRRWLPVGTAVVALLVLLGLPFLSVKWGFPDDRVLPRSASARQVGDIL

RDDFGHDPATQIPIVVPDARGLGPVELDSYAAELSRVPDVSAVTAPTGTFVDGSWVGTPR

GATGLAEGSAFLTVSSTAPLFSRASDIQLKRLHQVAGPAGRSVVMAGVAQVNRDSVDAVT

DRLPMVLGLIAAITYVLLFLLTGSVVLPAKALVCNVLSLTAAFGALVWIFQEGHFGALGT

TPSGTLVANMPVLLFCIAFGLSTDYEVFLVSRIREYWLESGAARPARRSVAEVHAANDES

VALGVARTGRVITAAALVMSMSFAALIAAHVSFMRMFGLGLTLAVAADATLVRMVVVPAF

MHVTGRWNWWAPRPLAWLHERFGVSEAAEPVSRRRSHAGGLGKIAGRSDGQTIPASLTRN

G

>mtue:J114_06180 transmembrane transport protein; K06994 putative drug exporter of the RND superfamily (A)

MLQRIARLAIAAPRRIIGFAVFVFIAAAVFGVPVADSLSPGGFQDPRSESARAIEVLTDK

FGQSGQKMLIVVTAAAGADSPPAREVGTDIVEVLRRSPLVYNVTSPWTVPPTAAADLLST

DGKSGLIVVNVKGGENDAQNHAQTLSDEVAHDRDGVTVRAGGSAMEYAQINRQNKDDLLV

MELIAIPLSFLVLIWVFGGLLAAGLPMAQAVLAVVGSMAVLRLVTFATEVSTFALNLSTA

LGLALAIDYTLLIVSRYRDELAEGSDRDEALIRTMATSGRTVLFSAVTVALSMSATALFP

MYFLKSFAYAGVATVAFVATASIVITPAAIVLLGPRLDALDVRRLVRRLLGRPDPVHKPV

KQLFWYRSSKFVMRRWLPVGTAVVALLVLLGLPFLSVKWGFPDDRVLPRSASARQVGDIL

RDDFGHDPATQIPIVVPDARGLGPVELDSYAAELSRVPDVSAVAAPTGTFVDGSWVGTPR

GATGLAEGSAFLTVSSTAPLFSRASDIQLKRLHQVAGPAGRSVVMAGVAQVNRDSVDAVT

DRLPMVLGLIAAITYVLLFLLTGSVVLPAKALVCNVLSLTAAFGALVWIFQEGHFGALGT

TPSGTLVANMPVLLFCIAFGLSMDYEVFLVSRIREYWLESGAARPARRSVAEVHAANDES

VALGVARTGRVITAAALVMSMSFAALIAAHVSFMRMFGLGLTLAVAADATLVRMVVVPAF

MHVTGRWNWWAPRPLAWLHERFGVSEAAEPVSRRRSHAGGLGKIAGRSDGQTIPASLTRN

G

>mtx:M943_01085 membrane protein; K20466 heme transporter (A)

MMRLSRNLRRCRWLVFTGWLLALVPAVYLAMTQSGNLTGGGFEVAGSQSLLVHDQLDAHY

PDRGAPALALVAAPRPDASYQDIDNAVALLRQIASELPGVTEAPNPTQRPPQPDRPYVVS

LRLDARNAGTSDVAKKLRDRIGVKGDQSGQTANGKVRLYVIGQGALSAAAAANTKHDIAN

AERWNLPIILMVLVAVFGSLAAAAIPLALAVCTVVITMGLVFVLSMHTTMSVFVTSTVSM

FGIALAVDYSLFILMRYREELRCGRRPPDAVDAAMATSGLAVVLSGMTVIASLTGIYLIN

TPALRSMATGAILAVAVAMLTSATLTPAVLATFARAAAKRSALVHWSRRPASTQSWFWSR

WVGWVMRRPWITALAASTVLLVMAAPATLMVLGNSLLRQFDSSHEIRTGAAAAAQALGPG

ALGPVQVLVRFDAGGASAPEHSQTIAAIRHRIAQAPNVVSVAPPRFADDNGSALLSAVLS

VDPEDLGARDTITWMRTQLPRVAGAAQVDVGGPTALIKDFDDRVSATQPLVLVFVAVIAF

LMLLISIRSVFLAFKGVLMTLLSVAAAYGSLVMVFQWGWARGLGFPALHSIDSTVPPLVL

AMTFGLSMDYEIFLLTRIRERFLQTGQTRDAVAYGVRTSARTITSAALIMIAVFCGFAFA

GMPLVAEIGVACAVAIAVDATVVRLVLVPALMAMFDRWNWWLPRWLAHILPSVDFDRPLP

KVDLGDVVVIPDDFAAAIPPSADVRMVLKSAAKLKRLAPDAICVTDPLAFTGCGCDGKAL

DQVQLAYRNGIARAISWGQRPVHPVTVWRKRLAVALDALQTTTWECGGVQTHRAGPGYRR

RSPVETTNVALPTGDRLQIPTGAETLRFKGYLIMSRNSSHDYADFADLVDTMAPETAAAV

LAGMDRYYSCQAPGRQWMATQLVGRLADPQPSDLGDQSPGADAQAKWEEVRRRCLSVAVA

MLEEAR

>mtuh:I917_01485 transmembrane transporter mmpL3; K20470 trehalose monomycolate/heme transporter (A)

MFAWWGRTVYRYRFIVIGVMVALCLGGGVFGLSLGKHVTQSGFYDXGSQSVQASVLGDQV

YGRDRSGHIVAIFQAPAGKTVDDPAWSKKVVDELNRFQQDHPDQVLGWAGYLRASQATGM

ATADKKYTFVSIPLKGDDDDTILNNYKAIAPDLQRLDGGTVKLAGLQPVAEALTGTIATD

QRRMEVLALPLVAVXXFFVFGGVIAAGLPVMVGGLCIAGALGIMRFLAIFGPVHYFAQPV

VSLIGLGIAIDYGLFIVSRFREEIAEGYDTETAVRRTVITAGRTVTFSAXLIVASAIGLL

LFPQGFLKSLTYATIASVMLSAILSITVLPACLGILGKHVDALGVRTLFRVPFLANWKIS

AAYLNWLADRLQRTKTREEVEAGFWGKLVNRVXXRPVLFAAPIVIIMILLIIPVGKLSLG

GISEKYLPPTNSVRQAQEEFDKLFPGYRTNPLTLVIQTSNHQPVTDAQIADIRSKAMAIG

GFIEPDNDPANMWQERAYAVGASKDPSVRVLQNGLINPADASKKLTELRAITPPKGITVL

VGGTPALELDSIHGLFAKMPLMVVILLTTTIVLMFLAFGSVVLPIKATLMSALTLGSTMG

ILTWIFVDGHFSKWLNFTPTPLTAPVIGLIIALVFGLSTDYEVFLVSRMVEARERGMSTQ

XAIRIGTAATGRIITAAALIVAVVAGAFXFSDLVMMKYLAFGLMAALLLDATVVRMFLVP

SVMKLLGDDCWWAPRWARRLQTRIGLGEIHLPDERKRPVSNGRPARPPVTAGLVAARAAG

DPRPPHDPTHPLAESPRPARSSPASSPELTPALEATAAPAAPSGASTTRMQIGSSTEPPT

TRLAAAGRSVQSPASTPPPTPTPPSAPSAGQTRAMPLAANRSTDAAGDPAEPTAALPIIR

SDGDDSEAATEQLNARGTSDKTRQRRRGGGALSAQDLLRREGRL

>mtul:TBHG_00202 transmembrane transporter MmpL11; K20466 heme transporter (A)

MMRLSRNLRRCRWLVFTGWLLALVPAVYLAMTQSGNLTGGGFEVAGSQSLLVHDQLDAHY

PDRGAPALALVAAPRPDASYQDIDNAVALLRQIASELPGVTEAPNPTQRPPQPDRPYVVS

LRLDARNAGTSDVAKKLRDRIGVKGDQSGQTANGKVRLYVIGQGALSAAAAANTKHDIAN

AERWNLPIILMVLVAVFGSLAAAAIPLALAVCTVVITMGLVFVLSMHTTMSVFVTSTVSM

FGIALAVDYSLFILMRYREELRCGRRPPDAVDAAMATSGLAVVLSGMTVIASLTGIYLIN

TPALRSMATGAILAVAVAMLTSATLTPAVLATFARAAAKRSALVHWSRRPASTQSWFWSR

WVGWVMRRPWITALAASTVLLVMAAPATLMVLGNSLLRQFDSSHEIRTGAAAAAQALGPG

ALGPVQVLVRFDAGGASAPEHSQTIAAIRHRIAQAPNVVSVAPPRFADDNGSALLSAVLS

VDPEDLGARDTITWMRTQLPRVAGAAQVDVGGPTALIKDFDDRVSATQPLVLVFVAVIAF

LMLLISIRSVFLAFKGVLMTLLSVAAAYGSLVMVFQWGWARGLGFPALHSIDSTVPPLVL

AMTFGLSMDYEIFLLTRIRERFLQTGQTRDAVAYGVRTSARTITSAALIMIAVFCGFAFA

GMPLVAEIGVACAVAIAVDATVVRLVLVPALMAMFDRWNWWLPRWLAHILPSVDFDRPLP

KVDLGDVVVIPDDFAAAIPPSADVRMVLKSAAKLKRLAPDAICVTDPLAFTGCGCDGKAL

DQVQLAYRNGIARAISWGQRPVHPVTVWRKRLAVALDALQTTTWECGGVQTHRAGPGYRR

RSPVETTNVALPTGDRLQIPTGAETLRFKGYLIMSRNSSHDYADFADLVDTMAPETAAAV

LAGMDRYYSCQAPGRQWMATQLVGRLADPQPSDLGDQSPGADAQAKWEEVRRRCLSVAVA

MLEEAR

>mtut:HKBT1_0218 mmpL11; transmembrane transporter MmpL11; K20466 heme transporter (A)

MMRLSRNLRRCRWLVFTGWLLALVPAVYLAMTQSGNLTGGGFEVAGSQSLLVHDQLDAHY

PDRGAPALALVAAPRPDASYQDIDNAVALLRQIASELPGVTEAPNPTQRPPQPDRPYVVS

LRLDARNAGTSDVAKKLRDRIGVKGDQSGQTANGKVRLYVIGQGALSAAAAANTKHDIAN

AERWNLPIILMVLVAVFGSLAAAAIPLALAVCTVVITMGLVFVLSMHTTMSVFVTSTVSM

FGIALAVDYSLFILMRYREELRCGRRPPDAVDAAMATSGLAVVLSGMTVIASLTGIYLIN

TPALRSMATGAILAVAVAMLTSATLTPAVLATFARAAAKRSALVHWSRRPASTQSWFWSR

WVGWVMRRPWITALAASTVLLVMAAPATLMVLGNSLLRQFDSSHEIRTGAAAAAQALGPG

ALGPVQVLVRFDAGGASAPEHSQTIAAIRHRIAQAPNVVSVAPPRFADDNGSALLSAVLS

VDPEDLGARDTITWMRTQLPRVAGAAQVDVGGPTALIKDFDDRVSATQPLVLVFVAVIAF

LMLLISIRSVFLAFKGVLMTLLSVAAAYGSLVMVFQWGWARGLGFPALHSIDSTVPPLVL

AMTFGLSMDYEIFLLTRIRERFLQTGQTRDAVAYGVRTSARTITSAALIMIAVFCGFAFA

GMPLVAEIGVACAVAIAVDATVVRLVLVPALMAMFDRWNWWLPRWLAHILPSVDFDRPLP

KVDLGDVVVIPDDFAAAIPPSADVRMVLKSAAKLKRLAPDAICVTDPLAFTGCGCDGKAL

DQVQLAYRNGIARAISWGQRPVHPVTVWRKRLAVALDALQTTTWECGGVQTHRAGPGYRR

RSPVETTNVALPTGDRLQIPTGAETLRFKGYLIMSRNSSHDYADFADLVDTMAPETAAAV

LAGMDRYYSCQAPGRQWMATQLVGRLADPQPSDLGDQSPGADAQAKWEEVRRRCLSVAVA

MLEEAR

>mtuu:HKBT2_0218 mmpL11; transmembrane transporter MmpL11; K20466 heme transporter (A)

MMRLSRNLRRCRWLVFTGWLLALVPAVYLAMTQSGNLTGGGFEVAGSQSLLVHDQLDAHY

PDRGAPALALVAAPRPDASYQDIDNAVALLRQIASELPGVTEAPNPTQRPPQPDRPYVVS

LRLDARNAGTSDVAKKLRDRIGVKGDQSGQTANGKVRLYVIGQGALSAAAAANTKHDIAN

AERWNLPIILMVLVAVFGSLAAAAIPLALAVCTVVITMGLVFVLSMHTTMSVFVTSTVSM

FGIALAVDYSLFILMRYREELRCGRRPPDAVDAAMATSGLAVVLSGMTVIASLTGIYLIN

TPALRSMATGAILAVAVAMLTSATLTPAVLATFARAAAKRSALVHWSRRPASTQSWFWSR

WVGWVMRRPWITALAASTVLLVMAAPATLMVLGNSLLRQFDSSHEIRTGAAAAAQALGPG

ALGPVQVLVRFDAGGASAPEHSQTIAAIRHRIAQAPNVVSVAPPRFADDNGSALLSAVLS

VDPEDLGARDTITWMRTQLPRVAGAAQVDVGGPTALIKDFDDRVSATQPLVLVFVAVIAF

LMLLISIRSVFLAFKGVLMTLLSVAAAYGSLVMVFQWGWARGLGFPALHSIDSTVPPLVL

AMTFGLSMDYEIFLLTRIRERFLQTGQTRDAVAYGVRTSARTITSAALIMIAVFCGFAFA

GMPLVAEIGVACAVAIAVDATVVRLVLVPALMAMFDRWNWWLPRWLAHILPSVDFDRPLP

KVDLGDVVVIPDDFAAAIPPSADVRMVLKSAAKLKRLAPDAICVTDPLAFTGCGCDGKAL

DQVQLAYRNGIARAISWGQRPVHPVTVWRKRLAVALDALQTTTWECGGVQTHRAGPGYRR

RSPVETTNVALPTGDRLQIPTGAETLRFKGYLIMSRNSSHDYADFADLVDTMAPETAAAV

LAGMDRYYSCQAPGRQWMATQLVGRLADPQPSDLGDQSPGADAQAKWEEVRRRCLSVAVA

MLEEAR

>mtq:HKBS1_0218 mmpL11; transmembrane transporter MmpL11; K20466 heme transporter (A)

MMRLSRNLRRCRWLVFTGWLLALVPAVYLAMTQSGNLTGGGFEVAGSQSLLVHDQLDAHY

PDRGAPALALVAAPRPDASYQDIDNAVALLRQIASELPGVTEAPNPTQRPPQPDRPYVVS

LRLDARNAGTSDVAKKLRDRIGVKGDQSGQTANGKVRLYVIGQGALSAAAAANTKHDIAN

AERWNLPIILMVLVAVFGSLAAAAIPLALAVCTVVITMGLVFVLSMHTTMSVFVTSTVSM

FGIALAVDYSLFILMRYREELRCGRRPPDAVDAAMATSGLAVVLSGMTVIASLTGIYLIN

TPALRSMATGAILAVAVAMLTSATLTPAVLATFARAAAKRSALVHWSRRPASTQSWFWSR

WVGWVMRRPWITALAASTVLLVMAAPATLMVLGNSLLRQFDSSHEIRTGAAAAAQALGPG

ALGPVQVLVRFDAGGASAPEHSQTIAAIRHRIAQAPNVVSVAPPRFADDNGSALLSAVLS

VDPEDLGARDTITWMRTQLPRVAGAAQVDVGGPTALIKDFDDRVSATQPLVLVFVAVIAF

LMLLISIRSVFLAFKGVLMTLLSVAAAYGSLVMVFQWGWARGLGFPALHSIDSTVPPLVL

AMTFGLSMDYEIFLLTRIRERFLQTGQTRDAVAYGVRTSARTITSAALIMIAVFCGFAFA

GMPLVAEIGVACAVAIAVDATVVRLVLVPALMAMFDRWNWWLPRWLAHILPSVDFDRPLP

KVDLGDVVVIPDDFAAAIPPSADVRMVLKSAAKLKRLAPDAICVTDPLAFTGCGCDGKAL

DQVQLAYRNGIARAISWGQRPVHPVTVWRKRLAVALDALQTTTWECGGVQTHRAGPGYRR

RSPVETTNVALPTGDRLQIPTGAETLRFKGYLIMSRNSSHDYADFADLVDTMAPETAAAV

LAGMDRYYSCQAPGRQWMATQLVGRLADPQPSDLGDQSPGADAQAKWEEVRRRCLSVAVA

MLEEAR

>mbo:Mb0208c mmpL11; transmembrane transport protein MmpL11; K20466 heme transporter (A)

MMRLSRNLRRCRWLVFTGWLLALVPAVYLAMTQSGNLTGGGFEVAGSQSLLVHDQLDAHY

PDRGAPALALVAAPRPDASYQDIDNAVALLRQIASELPGVTEAPNPTQRPPQPDRPYVVS

LRLDARNAGTSDVAKKLRDRIGVKGDQSGQTANGKVRLYVIGQGALSAAAAANTKHDIAN

AERWNLPIILMVLVAVFGSLAAAAIPLALAVCTVVITMGLVFVLSMHTTMSVFVTSTVSM

FGIALAVDYSLFILMRYREELRCGRRPPDAVDAAMATSGLAVVLSGMTVIASLTGIYLIN

TPALRSMATGAILAVAVAMLTSATLTPAVLATFARAAAKRSALVHWSRRPASTQSWFWSR

WVGWVMRRPWITALAASTVLLVMAAPATLMVLGNSLLRQFDSSHEIRTGAAAAAQALGPG

ALGPVQVLVRFDAGGASAPEHSQTIAAIRHRIAQAPNVVSVAPPRFADDNGSALLSAVLS

VDPEDLGARDTITWMRTQLPRVAGAAQVDVGGPTALIKDFDDRVSATQPLVLVFVAVIAF

LMLLISIRSVFLAFKGVLMTLLSVAAAYGSLVMVFQWGWARGLGFPALHSIDSTVPPLVL

AMTFGLSMDYEIFLLTRIRERFLQTGQTRDAVAYGVRTSARTITSAALIMIAVFCGFAFA

GMPLVAEIGVACAVAIAVDATVVRLVLVPALMAMFDRWNWWLPRWLAHILPSVDFDRPLP

KVDLGDVVVIPDDFAAAIPPSADVRMVLKSAAKLKRLAPDAICVTDPLAFTGCGCDGKAL

DQVQLAYRNGIARAISWGQRPVHPVTVWRKRLAVALDALQTTTWECGGVQTHRAGPGYRR

RSPVETTNVALPTGDRLQIPTGAETLRFKGYLIMSRNSSHDYADFADLVDTMAPETAAAV

LAGMDRYYSCQAPGRQWMATQLVGRLADPQPSDLGDQSPGADAQAKWEEVRRRCLSVAVA

MLEEAR

>mbb:BCG_0239c mmpL11; Probable conserved transmembrane transport protein mmpL11; K20466 heme transporter (A)

MMRLSRNLRRCRWLVFTGWLLALVPAVYLAMTQSGNLTGGGFEVAGSQSLLVHDQLDAHY

PDRGAPALALVAAPRPDASYQDIDNAVALLRQIASELPGVTEAPNPTQRPPQPDRPYVVS

LRLDARNAGTSDVAKKLRDRIGVKGDQSGQTANGKVRLYVIGQGALSAAAAANTKHDIAN

AERWNLPIILMVLVAVFGSLAAAAIPLALAVCTVVITMGLVFVLSMHTTMSVFVTSTVSM

FGIALAVDYSLFILMRYREELRCGRRPPDAVDAAMATSGLAVVLSGMTVIASLTGIYLIN

TPALRSMATGAILAVAVAMLTSATLTPAVLATFARAAAKRSALVHWSRRPASTQSWFWSR

WVGWVMRRPWITALAASTVLLVMAAPATLMVLGNSLLRQFDSSHEIRTGAAAAAQALGPG

ALGPVQVLVRFDAGGASAPEHSQTIAAIRHRIAQAPNVVSVAPPRFADDNGSALLSAVLS

VDPEDLGARDTITWMRTQLPRVAGAAQVDVGGPTALIKDFDDRVSATQPLVLVFVAVIAF

LMLLISIRSVFLAFKGVLMTLLSVAAAYGSLVMVFQWGWARGLGFPALHSIDSTVPPLVL

AMTFGLSMDYEIFLLTRIRERFLQTGQTRDAVAYGVRTSARTITSAALIMIAVFCGFAFA

GMPLVAEIGVACAVAIAVDATVVRLVLVPALMAMFDRWNWWLPRWLAHILPSVDFDRPLP

KVDLGDVVVIPDDFAAAIPPSADVRMVLKSAAKLKRLAPDAICVTDPLAFTGCGCDGKAL

DQVQLAYRNGIARAISWGQRPVHPVTVWRKRLAVALDALQTTTWECGGVQTHRAGPGYRR

RSPVETTNVALPTGDRLQIPTGAETLRFKGYLIMSRNSSHDYADFADLVDTMAPETAAAV

LAGMDRYYSCQAPGRQWMATQLVGRLADPQPSDLGDQSPGADAQAKWEEVRRRCLSVAVA

MLEEAR

>mbt:JTY_0208 mmpL11; putative transmembrane transport protein; K20466 heme transporter (A)

MMRLSRNLRRCRWLVFTGWLLALVPAVYLAMTQSGNLTGGGFEVAGSQSLLVHDQLDAHY

PDRGAPALALVAAPRPDASYQDIDNAVALLRQIASELPGVTEAPNPTQRPPQPDRPYVVS

LRLDARNAGTSDVAKKLRDRIGVKGDQSGQTANGKVRLYVIGQGALSAAAAANTKHDIAN

AERWNLPIILMVLVAVFGSLAAAAIPLALAVCTVVITMGLVFVLSMHTTMSVFVTSTVSM

FGIALAVDYSLFILMRYREELRCGRRPPDAVDAAMATSGLAVVLSGMTVIASLTGIYLIN

TPALRSMATGAILAVAVAMLTSATLTPAVLATFARAAAKRSALVHWSRRPASTQSWFWSR

WVGWVMRRPWITALAASTVLLVMAAPATLMVLGNSLLRQFDSSHEIRTGAAAAAQALGPG

ALGPVQVLVRFDAGGASAPEHSQTIAAIRHRIAQAPNVVSVAPPRFADDNGSALLSAVLS

VDPEDLGARDTITWMRTQLPRVAGAAQVDVGGPTALIKDFDDRVSATQPLVLVFVAVIAF

LMLLISIRSVFLAFKGVLMTLLSVAAAYGSLVMVFQWGWARGLGFPALHSIDSTVPPLVL

AMTFGLSMDYEIFLLTRIRERFLQTGQTRDAVAYGVRTSARTITSAALIMIAVFCGFAFA

GMPLVAEIGVACAVAIAVDATVVRLVLVPALMAMFDRWNWWLPRWLAHILPSVDFDRPLP

KVDLGDVVVIPDDFAAAIPPSADVRMVLKSAAKLKRLAPDAICVTDPLAFTGCGCDGKAL

DQVQLAYRNGIARAISWGQRPVHPVTVWRKRLAVALDALQTTTWECGGVQTHRAGPGYRR

RSPVETTNVALPTGDRLQIPTGAETLRFKGYLIMSRNSSHDYADFADLVDTMAPETAAAV

LAGMDRYYSCQAPGRQWMATQLVGRLADPQPSDLGDQSPGADAQAKWEEVRRRCLSVAVA

MLEEAR

>mbm:BCGMEX_0208c mmpL11; Putative transmembrane transport protein; K20466 heme transporter (A)

MMRLSRNLRRCRWLVFTGWLLALVPAVYLAMTQSGNLTGGGFEVAGSQSLLVHDQLDAHY

PDRGAPALALVAAPRPDASYQDIDNAVALLRQIASELPGVTEAPNPTQRPPQPDRPYVVS

LRLDARNAGTSDVAKKLRDRIGVKGDQSGQTANGKVRLYVIGQGALSAAAAANTKHDIAN

AERWNLPIILMVLVAVFGSLAAAAIPLALAVCTVVITMGLVFVLSMHTTMSVFVTSTVSM

FGIALAVDYSLFILMRYREELRCGRRPPDAVDAAMATSGLAVVLSGMTVIASLTGIYLIN

TPALRSMATGAILAVAVAMLTSATLTPAVLATFARAAAKRSALVHWSRRPASTQSWFWSR

WVGWVMRRPWITALAASTVLLVMAAPATLMVLGNSLLRQFDSSHEIRTGAAAAAQALGPG

ALGPVQVLVRFDAGGASAPEHSQTIAAIRHRIAQAPNVVSVAPPRFADDNGSALLSAVLS

VDPEDLGARDTITWMRTQLPRVAGAAQVDVGGPTALIKDFDDRVSATQPLVLVFVAVIAF

LMLLISIRSVFLAFKGVLMTLLSVAAAYGSLVMVFQWGWARGLGFPALHSIDSTVPPLVL

AMTFGLSMDYEIFLLTRIRERFLQTGQTRDAVAYGVRTSARTITSAALIMIAVFCGFAFA

GMPLVAEIGVACAVAIAVDATVVRLVLVPALMAMFDRWNWWLPRWLAHILPSVDFDRPLP

KVDLGDVVVIPDDFAAAIPPSADVRMVLKSAAKLKRLAPDAICVTDPLAFTGCGCDGKAL

DQVQLAYRNGIARAISWGQRPVHPVTVWRKRLAVALDALQTTTWECGGVQTHRAGPGYRR

RSPVETTNVALPTGDRLQIPTGAETLRFKGYLIMSRNSSHDYADFADLVDTMAPETAAAV

LAGMDRYYSCQAPGRQWMATQLVGRLADPQPSDLGDQSPGADAQAKWEEVRRRCLSVAVA

MLEEAR

>mbk:K60_002230 transmembrane transport protein MmpL11; K20466 heme transporter (A)

MMRLSRNLRRCRWLVFTGWLLALVPAVYLAMTQSGNLTGGGFEVAGSQSLLVHDQLDAHY

PDRGAPALALVAAPRPDASYQDIDNAVALLRQIASELPGVTEAPNPTQRPPQPDRPYVVS

LRLDARNAGTSDVAKKLRDRIGVKGDQSGQTANGKVRLYVIGQGALSAAAAANTKHDIAN

AERWNLPIILMVLVAVFGSLAAAAIPLALAVCTVVITMGLVFVLSMHTTMSVFVTSTVSM

FGIALAVDYSLFILMRYREELRCGRRPPDAVDAAMATSGLAVVLSGMTVIASLTGIYLIN

TPALRSMATGAILAVAVAMLTSATLTPAVLATFARAAAKRSALVHWSRRPASTQSWFWSR

WVGWVMRRPWITALAASTVLLVMAAPATLMVLGNSLLRQFDSSHEIRTGAAAAAQALGPG

ALGPVQVLVRFDAGGASAPEHSQTIAAIRHRIAQAPNVVSVAPPRFADDNGSALLSAVLS

VDPEDLGARDTITWMRTQLPRVAGAAQVDVGGPTALIKDFDDRVSATQPLVLVFVAVIAF

LMLLISIRSVFLAFKGVLMTLLSVAAAYGSLVMVFQWGWARGLGFPALHSIDSTVPPLVL

AMTFGLSMDYEIFLLTRIRERFLQTGQTRDAVAYGVRTSARTITSAALIMIAVFCGFAFA

GMPLVAEIGVACAVAIAVDATVVRLVLVPALMAMFDRWNWWLPRWLAHILPSVDFDRPLP

KVDLGDVVVIPDDFAAAIPPSADVRMVLKSAAKLKRLAPDAICVTDPLAFTGCGCDGKAL

DQVQLAYRNGIARAISWGQRPVHPVTVWRKRLAVALDALQTTTWECGGVQTHRAGPGYRR

RSPVETTNVALPTGDRLQIPTGAETLRFKGYLIMSRNSSHDYADFADLVDTMAPETAAAV

LAGMDRYYSCQAPGRQWMATQLVGRLADPQPSDLGDQSPGADAQAKWEEVRRRCLSVAVA

MLEEAR

>mbx:BCGT_4004 Integral membrane protein; K20466 heme transporter (A)

MMRLSRNLRRCRWLVFTGWLLALVPAVYLAMTQSGNLTGGGFEVAGSQSLLVHDQLDAHY

PDRGAPALALVAAPRPDASYQDIDNAVALLRQIASELPGVTEAPNPTQRPPQPDRPYVVS

LRLDARNAGTSDVAKKLRDRIGVKGDQSGQTANGKVRLYVIGQGALSAAAAANTKHDIAN

AERWNLPIILMVLVAVFGSLAAAAIPLALAVCTVVITMGLVFVLSMHTTMSVFVTSTVSM

FGIALAVDYSLFILMRYREELRCGRRPPDAVDAAMATSGLAVVLSGMTVIASLTGIYLIN

TPALRSMATGAILAVAVAMLTSATLTPAVLATFARAAAKRSALVHWSRRPASTQSWFWSR

WVGWVMRRPWITALAASTVLLVMAAPATLMVLGNSLLRQFDSSHEIRTGAAAAAQALGPG

ALGPVQVLVRFDAGGASAPEHSQTIAAIRHRIAQAPNVVSVAPPRFADDNGSALLSAVLS

VDPEDLGARDTITWMRTQLPRVAGAAQVDVGGPTALIKDFDDRVSATQPLVLVFVAVIAF

LMLLISIRSVFLAFKGVLMTLLSVAAAYGSLVMVFQWGWARGLGFPALHSIDSTVPPLVL

AMTFGLSMDYEIFLLTRIRERFLQTGQTRDAVAYGVRTSARTITSAALIMIAVFCGFAFA

GMPLVAEIGVACAVAIAVDATVVRLVLVPALMAMFDRWNWWLPRWLAHILPSVDFDRPLP

KVDLGDVVVIPDDFAAAIPPSADVRMVLKSAAKLKRLAPDAICVTDPLAFTGCGCDGKAL

DQVQLAYRNGIARAISWGQRPVHPVTVWRKRLAVALDALQTTTWECGGVQTHRAGPGYRR

RSPVETTNVALPTGDRLQIPTGAETLRFKGYLIMSRNSSHDYADFADLVDTMAPETAAAV

LAGMDRYYSCQAPGRQWMATQLVGRLADPQPSDLGDQSPGADAQAKWEEVRRRCLSVAVA

MLEEAR

>mbz:LH58_01120 membrane protein; K20466 heme transporter (A)

MMRLSRNLRRCRWLVFTGWLLALVPAVYLAMTQSGNLTGGGFEVAGSQSLLVHDQLDAHY

PDRGAPALALVAAPRPDASYQDIDNAVALLRQIASELPGVTEAPNPTQRPPQPDRPYVVS

LRLDARNAGTSDVAKKLRDRIGVKGDQSGQTANGKVRLYVIGQGALSAAAAANTKHDIAN

AERWNLPIILMVLVAVFGSLAAAAIPLALAVCTVVITMGLVFVLSMHTTMSVFVTSTVSM

FGIALAVDYSLFILMRYREELRCGRRPPDAVDAAMATSGLAVVLSGMTVIASLTGIYLIN

TPALRSMATGAILAVAVAMLTSATLTPAVLATFARAAAKRSALVHWSRRPASTQSWFWSR

WVGWVMRRPWITALAASTVLLVMAAPATLMVLGNSLLRQFDSSHEIRTGAAAAAQALGPG

ALGPVQVLVRFDAGGASAPEHSQTIAAIRHRIAQAPNVVSVAPPRFADDNGSALLSAVLS

VDPEDLGARDTITWMRTQLPRVAGAAQVDVGGPTALIKDFDDRVSATQPLVLVFVAVIAF

LMLLISIRSVFLAFKGVLMTLLSVAAAYGSLVMVFQWGWARGLGFPALHSIDSTVPPLVL

AMTFGLSMDYEIFLLTRIRERFLQTGQTRDAVAYGVRTSARTITSAALIMIAVFCGFAFA

GMPLVAEIGVACAVAIAVDATVVRLVLVPALMAMFDRWNWWLPRWLAHILPSVDFDRPLP

KVDLGDVVVIPDDFAAAIPPSADVRMVLKSAAKLKRLAPDAICVTDPLAFTGCGCDGKAL

DQVQLAYRNGIARAISWGQRPVHPVTVWRKRLAVALDALQTTTWECGGVQTHRAGPGYRR

RSPVETTNVALPTGDRLQIPTGAETLRFKGYLIMSRNSSHDYADFADLVDTMAPETAAAV

LAGMDRYYSCQAPGRQWMATQLVGRLADPQPSDLGDQSPGADAQAKWEEVRRRCLSVAVA

MLEEAR

>maf:MAF_02030 mmpL11; putative conserved transmembrane transport protein MMPL11; K20466 heme transporter (A)

MMRLSRNLRRCRWLVFTGWLLALVPAVYLAMTQSGNLTGGGFEVAGSQSLLVHDQLDAHY

PDRGAPALALVAAPRPDASYQDIDNAVALLRQIASELPGVTEAPNPTQRPPQPDRPYVVS

LRLDARNAGTSDVAKKLRDRIGVKGDQSGQTANGKVRLYVIGQGALSAAAAANTKHDIAN

AERWNLPIILMVLVAVFGSLAAAAIPLALAVCTVVITMGLVFVLSMHTTMSVFVTSTVSM

FGIALAVDYSLFILMRYREELRCGRRPPDAVDAAMATSGLAVVLSGMTVIASLTGIYLIN

TPALRSMATGAILAVAVAMLTSATLTPAVLATFARAAAKRSALVHWSRRPASTQSWFWSR

WVGWVMRRPWITALAASTVLLVMAAPATLMVLGNSLLRQFDSSHEIRTGAAAAAQALGPG

ALGPVQVLVRFDAGGASAPEHSQTIAAIRHRIAQAPNVVSVAPPRFADDNGSALLSAVLS

VDPEDLGARDTITWMRTQLPRVAGAAQVDVGGPTALIKDFDDRVSATQPLVLVFVAVIAF

LMLLISIRSVFLAFKGVLMTLLSVAAAYGSLVMVFQWGWARGLGFPALHSIDSTVPPLVL

AMTFGLSMDYEIFLLTRIRERFLQTGQTRDAVAYGVRTSARTITSAALIMIAVFCGFAFA

GMPLVAEIGVACAVAIAVDATVVRLVLVPALMAMFDRWNWWLPRWLAHILPSVDFDRPLP

KVDLGDVVVIPDDFAAAIPPSADVRMVLKSAAKLKRLAPDAICVTDPLAFTGCGCDGKAL

DQVQLAYRNGIARAISWGQRPVHPVTVWRKRLAVALDALQTTTWECGGVQTHRAGPGYRR

RSPVETTNVALPTGDRLQIPTGAETLRFKGYLIMSRNSSHDYADFADLVDTMAPETAAAV

LAGMDRYYSCQAPGRQWMATQLVGRLADPQPSDLGDQSPGADAQAKWEEVRRRCLSVAVA

MLEEAR

>mce:MCAN_02081 mmpL11; putative conserved transmembrane transport protein MMPL11; K20466 heme transporter (A)

MMRLSRNLRRCRWLVFTGWLLALVPAVYLAMTQSGNLTGGGFEVAGSQSLLVHDQLDAHY

PDRGAPALALVAAPRPDASYQDIDNAVALLRQIASELPGVTEAPNPTQRPPQPDRPYVVS

LRLDARNAGTSDVAKKLRDRIGVKGDQSGQTANGKVRLYVIGQGALSAAAAANTKHDIAN

AERWNLPIILMVLVAVFGSLAAAAIPLALAVCTVVITMGLVFVLSMHTTMSVFVTSTVSM

FGIALAVDYSLFILMRYREELRCGRRPPDAVDAAMATSGLAVVLSGMTVIASLTGIYLIN

TPALRSMATGAILAVAVAMLTSATLTPAVLATFARAAAKRSALVHWSRRPASTQSWFWSR

WVGWVMRRPWITALAASTVLLVMAAPATLMVLGNSLLRQFDSSHEIRTGAAAAAQALGPG

ALGPVQVLVRFDAGGASAPEHSQTIAAIRHRMAQAPNVVSVAPPRFADDNGSALLSAVLS

VDPEDLGARDTITWMRTQLPRVAGAAQVDVGGPTALIKDFDDRVSATQPLVLVFVAVIAF

LMLLISIRSVFLAFKGVLMTLLSVAAAYGSLVMVFQWGWARGLGFPALHSIDSTVPPLVL

AMTFGLSMDYEIFLLTRIRERFLQTGQTRDAVAYGVRTSARTITSAALIMIAVFCGFAFA

GMPLVAEIGVACAVAIAVDATVVRLVLVPALMAMFDRWNWWLPRWLAHILPSVDFDRPLP

KVDLGDVVVIPDGFAAAIPPSADVRMVLKSAAKLKRLAPDAICVADPLAFTGCGCNGKAL

DQVQLAYRNGIARAMSWGQRPVHPVTVWRKRLAVALDALQTTTWECGGVQTHRAGPGYRR

SSPVETTNVALPTGDRLQIPTGAETLRFKGYLIMSRNSSHDYADFADLVDTMAPETAAAV

LAGMDRYYSCQAPGRQWMATQLVGRLADPQPSDLDDQSPGADAQAKWEEVRRRCLSVAVA

MLEEAR

>mcq:BN44_10236 mmpL; Conserved membrane protein of unknown function, Mmpl11, possible transport protein; K20466 heme transporter (A)

MMRLSRNLRRCRWLVFTGWLLALVPAVYLAMTQSGNLTGGGFEVAGSQSLLVHDQLDAHY

PDRGAPALALVAAPRPDASYQDIDNAVALLRQIASELPGVTEAPNPTQRPPQPDRPYVVS

LRLDARNAGTSDVAKKLRDRIGVKGDQSGQTANGKVRLYVIGQGALSAAAAANTKHDIAN

AERWNLPIILMVLVAVFGSLAAAAIPLALAVCTVVITMGLVFVLSMHTTMSVFVTSTVSM

FGIALAVDYSLFILMRYREELRCGRRPPDAVDAAMATSGLAVVLSGMTVIASLTGIYLIN

TPALRSMATGAILAVAVAMLTSATLTPAVLATFARAAAKRSALVHWSRRPASTQSWFWSR

WVGWVMRRPWITALAASTVLLVMAAPATLMVLGNSLLRQFDSSHEIRTGAAAAAQALGPG

ALGPVQVLVRFDAGGASAPEHSQTIAAIRHRMAQAPNVVSVAPPRFADDNGSALLSAVLS

VDPEDLGARDTITWMRTQLPRVAGAAQVDVGGPTALIKDFDDRVSATQPLVLVFVAVIAF

LMLLISIRSVFLAFKGVLMTLLSVAAAYGSLVMVFQWGWARGLGFPALHSIDSTVPPLVL

AMTFGLSMDYEIFLLTRIRERFLQTGQTRDAVAYGVRTSARTITSAALIMIAVFCGFAFA

GMPLVAEIGVACAVAIAVDATVVRLVLVPALMAMFDRWNWWLPRWLAHILPSVDFDRPLP

KVDLGDVVVIPDGFAAAIPPSADVRMVLKSAAKLKRLAPDAICVADPLAFTGCGCNGKAL

DQVQLAYRNGIARAMSWGQRPVHPVTVWRKRLAVALDALQTTTWECGGVQTHRAGPGYRR

SSPVETTNVALPTGDRLQIPTGAETLRFKGYLIMSRNSSHDYADFADLVDTMAPETAAAV

LAGMDRYYSCQAPGRQWMATQLVGRLADPQPSDLDDQSPGADAQAKWEEVRRRCLSVAVA

MLEEAR

>mcv:BN43_10229 mmpL; Conserved membrane protein of unknown function, Mmpl11, possible transport protein; K20466 heme transporter (A)

MMRLSRNLRRCRWLVFAGWLLALVPAVYLAMTQSGNLTGGGFEVAGSQSLLVHDQLDAHY

PDRGAPALALVAAPRPDASYQDIDNAVALLRQIASELPGVTEAPNPTQRPPQPDRPYVVS

LRLDARNAGTSDVAKKLRDRIGVKGDQSGQTANGKVRLYVIGQGALSAAAAANTKHDIAN

AERWNLPVILMVLVAVFGSLAAAAIPLALAVCTVVITMGLVFVLSMHTTMSVFVTSTVSM

FGIALAVDYSLFILMRYREELRCGRRPPDAVDAAMATSGLAVVLSGMTVIASLTGIYLIN

TPALRSMATGAILAVAVAMLTSATLTPAVLATFARAAAKRSALVHWSRRPASTQSWFWSR

WVGWVMRRPWITALAASTVLLVMAAPATLMVLGNSLLRQFDSSHEIRTGAAAAAQALGPG

ALGPVQVLVRFDAGGASAPEHSQTIAAIRHRMAQAPNVVSVAPPRFADDNGSALLSAVLS

VDPEDLGARDTITWMRTQLPRVAGAAQVDVGGPTALIKDFDDQVSATQPLVLVFVAVIAF

LMLLISIRSVFLAFKGVLMTLLSVAAAYGSLVMVFQWGWARGLGFPALHSIDSTVPPLVL

AMTFGLSMDYEIFLLTRIRERFLQTGQTRDAVAYGVRTSARTITSAALIMIAVFCGFAFA

GMPLVAEIGVACAVAIAVDATVVRLVLVPALMAMFDRWNWWLPRWLAHILPSVDFDRPLP

KVDLGDVVVIPDDFAAAIPPSADVRMVLKSAAKLKRLAPDAICVADPLAFTGCECNGKAL

DQVQLAYRNGIARAMSWGQRPVHPVTVWRKRLAVALDALQTTTWECGGVQTHRAGPGYRR

RSPVETTNVALPTGDRLQIPTGAETLRFKGYLIMSRNSSHDYADFADLVDTMAPETAAAV

LAGMDRYYSCQAPGRQWMATQLVGRLADPQPSDLDDQSPGADAQAKWEEVRRRCLSVAVA

MLEEAR

>mcx:BN42_10246 mmpL; Conserved membrane protein of unknown function, Mmpl11, possible transport protein; K20466 heme transporter (A)

MMRLSRNLRRCRWLVFTGWLLALVPAVYLAMTQSGNLTGGGFEVAGSQSLLVHDQLDAHY

PDRGAPALALVAAPRPDASYQDIDNAVALLRQIASELPGVTETPNPTQRPPQPDRPYVVS

LRLDARNAGTSDVAKKLRDRIGVKGDQSGQTANGKVRLYVIGQGALSAAAAANTKHDIAN

AERWNLPIILMVLVAVFGSLAAAAIPLALAVCTVVITMGLVFVLSMHTTMSVFVTSTVSM

FGIALAVDYSLFILMRYREELRCGRRPPDAVDAAMATSGLAVVLSGMTVIASLTGIYLIN

TPALRSMATGAILAVAVAMLTSATLTPAVLATFARAAAKRSALVHWSWRPASTQSWFWSR

WVGWVMRRPWITALAASTVLLVMAAPATSMVLGNSLLRQFDSSHEIRTGAAAAAQALGPG

ALGPVQVLVRFDAGGASAPEHSQTIAAIRHRMAQAPNVVSVAPPRFADDNGSALLSAVLS

VDPEDLGARDTITWMRTQLPRVAGAAQVDVGGPTALIKDFDDRVSATQPLVLVFVAVIAF

LMLLISIRSVFLAFKGVLMTLLSVAAAYGSLVMVFQWGWARGLGFPALHSIDSTVPPLVL

AMTFGLSMDYEIFLLTRIRERFLQTGQTRDAVAYGVRTSARTITSAALIMIAVFCGFAFA

GMPLVAEIGVACAVAIAVDATVVRLVLVPALMAMFDRWNWWLPRWLAHILPSVDFDRPLP

KVDLGDVVVIPDDFAAAIPPSADVRMVLKSAAKLKRLAPDAICVADPLAFTGCGCNGKAL

DQVQLAYRNGIARAMSWGQRPVHPVTVWRKRLAVALDALQTTTWECGGVQTHRAGPSYRR

SSPVETTNVALPTGDRLQIPTGAETLRFKGYLIMSRNSSHDYADFADLVDTMAPETAAAV

LAGMDRYYSCQAPGRQWMATQLVGRLADPQPSDLDDQSPGADAQAKWEEVRRRCLSVAVA

MLEEAR

>mcz:BN45_10224 mmpL; Conserved membrane protein of unknown function, Mmpl11, possible transport protein; K20466 heme transporter (A)

MMRLSRNLRRCRWLVFTGWLLALVPAVYLAMTQSGNLTGGGFEVAGSQSLLVHDQLDAHY

PDRGAPALALVAAPRPDASYQDIDYAVALLRQIASELPGVTEAPNPTQRPPQPDRPYVVS

LRLDARNAGTSDVAKKLRDRIGVKGDQSGQTANGKVRLYVIGQGALSAAAAANTKHDIAN

AERWNLPIILMVLVAVFGSLAAAAIPLALAVCTVVITMGLVFVLSMHTTMSVFVTSTVSM

FGIALAVDYSLFILMRYREELRCGRRPPDAVDAAMATSGLAVVLSGMTVIASLTGIYLIN

TPALRSMATGAILAVAVAMLTSATLTPAVLATFARAAAKRSALVHWSRRPASTQSWFWSR

WVGWVMRRPWITALAASTVLLVMAAPATSMVLGNSLLRQFDSSHEIRTGAAAAAQALGPG

ALGPVQVLVRFDAGGASAPEHSQTIAAIRHRMAQAPNVVSVAPPRFADDNGSALLSAVLS

VDPEDLGARDTITWMRTQLPRVAGAAQVDVGGPTALIKDFDDRVSATQPLVLVFVAVIAF

LMLLISIRSVFLAFKGVLMTLLSVAAAYGSLVMVFQWGWARGLGFPALHSIDSTVPPLVL

AMTFGLSMDYEIFLLTRIRERFLQTGQTRDAVAYGVRTSARTITSAALIMIAVFCGFAFA

GMPLVAEIGVACAVAIAVDATVVRLVLVPALMAMFDRWNWWLPQWLAHILPSVDFDRPLP

KVDLGDVVVIPDDFAAAIPPSADVRMVLKSAAKLKRLAPDAICVTDPLAFTGCGCNGKAL

DQVQLAYRNGIARAMSWGQRPVHPVTVWRKRLAVALDALQTTTWECGGVQTHRAGPGYRR

RSPVETTNVALPTGDRLQIPTGAETLRFKGYLIMSRNSSHDYADFADLVDTMAPETAAAV

LAGMDRYYSCQAPGRQWMATQLVGRLADPQPSDLGDQSPGADAQAKWEEVRRRCLSVAVA

MLEEAR

>mle:ML2617 hypothetical protein; K20466 heme transporter (A)

MRLSSYLRRFRWLVFTGWLLALVPAIYLAMTQSGKLTGGGFEVAGSQSLLVHDQLQEQYP

DQGAVSLALVAAPRSDASYQDMNDAVALLRRIVSEFPGVSEVPNPTQLPPRPDRPYGVSL

RLDDRNSVTSDVAKQLRTKVGIKGDQAGRTANGKVRLYVIGQGALSAAVAANSKHDIAEA

ERWNLPIILIVLLAVFGSLAAAAVPLALGVCTVVVTMGLVDLVSMHTIMSVFVTSTVSMF

GIALAVDYSLFILMRFREELRSGRQPQEAVDAAMATSGLAVVLSGMTVIASLTGIYLINT

AALKSMATGAILAVAIAMLASITLTPAALATFGRAAVKRSVLMHWSQRSECTQSLFWTRW

VGWVMHRPWISASAASTILIIMATPVTSMMLGNSLLRQFNSSHEIRAGVAAAAQALGPGA

LGPVQVLITFPDDPNTQASSPKHRQTIGAIRNRMLQAKNVMSVAPPQFADNNCSALLGAV

LSVDPEDLGARETVDWMRTELPKVPGAAHVNVGGPTALINDFDDRVAKTEPLMLVFVALI

AFVMLLISIRSVFLAFKGVLMTLLSVAAAYGSLVMVFQWGWLENLGFTHINSIDSTVPPL

VLAMTFGLSMDYEIFLLTRIRERFLQTGHTRDAVAYGVSTSARTITSAALIMIAVFVGFA

FAGMPLVAEIGVACAVAIAVDVTAVRLVLVPTLMAMFAQWNWWLPRWLSRALPAVDFDKP

FPPVDLNEIVVLPADISATKVPCGDLRMVLKLAAKLKNLAPDAICVADPLAFTGCGRNNK

RSDRVLPGAATQESEEDPAMGKASDSTAALTAAQVGPVTRTNGHWTARNLVIGLTHRNSI

TRVMPWSDRPVHPFTLWRSRFSVAIDALEAHIAVQADAPDQPNYQRCSPVETAHVQLPTG

DRLLIPTGAETLRLVSYLIMCRNSIRDYAELADMVDAIEPETAAVVLTELDRYYSCQLPM

RQWMATQLVRRLSDPHPVDLTEDQWSDPDNKAEWQDVRQRCLSVAVAMLEEAR

>mlb:MLBr02617 conserved integral membrane protein; K20466 heme transporter (A)

MRLSSYLRRFRWLVFTGWLLALVPAIYLAMTQSGKLTGGGFEVAGSQSLLVHDQLQEQYP

DQGAVSLALVAAPRSDASYQDMNDAVALLRRIVSEFPGVSEVPNPTQLPPRPDRPYGVSL

RLDDRNSVTSDVAKQLRTKVGIKGDQAGRTANGKVRLYVIGQGALSAAVAANSKHDIAEA

ERWNLPIILIVLLAVFGSLAAAAVPLALGVCTVVVTMGLVDLVSMHTIMSVFVTSTVSMF

GIALAVDYSLFILMRFREELRSGRQPQEAVDAAMATSGLAVVLSGMTVIASLTGIYLINT

AALKSMATGAILAVAIAMLASITLTPAALATFGRAAVKRSVLMHWSQRSECTQSLFWTRW

VGWVMHRPWISASAASTILIIMATPVTSMMLGNSLLRQFNSSHEIRAGVAAAAQALGPGA

LGPVQVLITFPDDPNTQASSPKHRQTIGAIRNRMLQAKNVMSVAPPQFADNNCSALLGAV

LSVDPEDLGARETVDWMRTELPKVPGAAHVNVGGPTALINDFDDRVAKTEPLMLVFVALI

AFVMLLISIRSVFLAFKGVLMTLLSVAAAYGSLVMVFQWGWLENLGFTHINSIDSTVPPL

VLAMTFGLSMDYEIFLLTRIRERFLQTGHTRDAVAYGVSTSARTITSAALIMIAVFVGFA

FAGMPLVAEIGVACAVAIAVDVTAVRLVLVPTLMAMFAQWNWWLPRWLSRALPAVDFDKP

FPPVDLNEIVVLPADISATKVPCGDLRMVLKLAAKLKNLAPDAICVADPLAFTGCGRNNK

RSDRVLPGAATQESEEDPAMGKASDSTAALTAAQVGPVTRTNGHWTARNLVIGLTHRNSI

TRVMPWSDRPVHPFTLWRSRFSVAIDALEAHIAVQADAPDQPNYQRCSPVETAHVQLPTG

DRLLIPTGAETLRLVSYLIMCRNSIRDYAELADMVDAIEPETAAVVLTELDRYYSCQLPM

RQWMATQLVRRLSDPHPVDLTEDQWSDPDNKAEWQDVRQRCLSVAVAMLEEAR

>mpa:MAP_3637c mmpL11; MmpL11; K20466 heme transporter (A)

MMRLSRSLRKYRWLVFAGWLLALVPAVYLALTQSGNLTGGGFDVAGSQSLAVHDQLEDLY

HDQGGSSLALVAAPRADASYQDMNDAVAQLRRIAAEVPGTTEIPNPTQRPPQPDRPYVLS

VRLDSRNTSDVAKQLRTKVGIKGDQPGQTANGRVRLYVIGQGALSAAAAANTKHDIAAAE

KWNLPVILIVLLAVFGSLAAAAIPLALGICTVVVTMGLVYLLSAYTTMSVFVTSTVSMFG

IALAVDYSLFILMRFREELRSGRQPREAVDAAMATSGLAVVLSGMTVIASLTGIYVINTP

ALKSMATGAILAVAVAMLTSTTLTPAALATFGRAAAKRSALLHWSRRPESTQSKFWNRWI

GWVMRRPWMSALAASLVLLVMAAPAASMVLGNSLLRQFDSSHEIRAGVGAAAQALGPGAL

GPIRVLINFPDGGAASPEHSHTVGAVRQRMAQAPNIVSVSPPQFAEDNGSALLSAVLSVD

PEDMKARETVGWMRAELPKVPEAGTARVDVGGPTALIKDFDDRVSATEPLVLGFVALIAF

VMLLVSIHSVFLALKGVLMTLLSVAAAYGSLVMVFQWGWLRDLGFAQISSIDSTVPPLVL

AMTFGLSMDYEIFLLTRIRERFLHSGNTRDAVAYGVSTSARTITSAALIMSAVFVGFAFA

GMPLVAEIGVACAVAIAVDATVVRLVMVPALMAMFAQWNWWLPPWLSRVLPSVDFDRPLP

EVDLGDVVVIPDDISALTAPSADLRMVLKSAAKLKHLAPDAICVTDPLAFTGCGRTTAAG

ADPARGLGPGQIPHQVALREEKVGVAAGPGEKTGSNGHTNGSAGAKKPAARNGRNGIAKA

IAGADRPVHPVTLWRGRLSVALDALQTDPDSGTDRPRFRRRSPVETTNVQLPTGDRLLVP

TGAETLRLKGYLLMCRNSRRDYADFADMVDALEPETAAVVLAGMDRYYCCESSRRQWIAT

QLVRRLADPDPCDYPDDQGPDADAPADWEQIRQRCLAVAVAMLEEAR

>mao:MAP4_0137 transmembrane transport protein; K20466 heme transporter (A)

MMRLSRSLRKYRWLVFAGWLLALVPAVYLALTQSGNLTGGGFDVAGSQSLAVHDQLEDLY

HDQGGSSLALVAAPRADASYQDMNDAVAQLRRIAAEVPGTTEIPNPTQRPPQPDRPYVLS

VRLDSRNTSDVAKQLRTKVGIKGDQPGQTANGRVRLYVIGQGALSAAAAANTKHDIAAAE

KWNLPVILIVLLAVFGSLAAAAIPLALGICTVVVTMGLVYLLSAYTTMSVFVTSTVSMFG

IALAVDYSLFILMRFREELRSGRQPREAVDAAMATSGLAVVLSGMTVIASLTGIYVINTP

ALKSMATGAILAVAVAMLTSTTLTPAALATFGRAAAKRSALLHWSRRPESTQSKFWNRWI

GWVMRRPWMSALAASLVLLVMAAPAASMVLGNSLLRQFDSSHEIRAGVGAAAQALGPGAL

GPIRVLINFPDGGAASPEHSHTVGAVRQRMAQAPNIVSVSPPQFAEDNGSALLSAVLSVD

PEDMKARETVGWMRAELPKVPEAGTARVDVGGPTALIKDFDDRVSATEPLVLGFVALIAF

VMLLVSIHSVFLALKGVLMTLLSVAAAYGSLVMVFQWGWLRDLGFAQISSIDSTVPPLVL

AMTFGLSMDYEIFLLTRIRERFLHSGNTRDAVAYGVSTSARTITSAALIMSAVFVGFAFA

GMPLVAEIGVACAVAIAVDATVVRLVMVPALMAMFAQWNWWLPPWLSRVLPSVDFDRPLP

EVDLGDVVVIPDDISALTAPSADLRMVLKSAAKLKHLAPDAICVTDPLAFTGCGRTTAAG

ADPARGLGPGQIPHQVALREEKVGVAAGPGEKTGSNGHTNGSAGAKKPAARNGRNGIAKA

IAGADRPVHPVTLWRGRLSVALDALQTDPDSGTDRPRFRRRSPVETTNVQLPTGDRLLVP

TGAETLRLKGYLLMCRNSRRDYADFADMVDALEPETAAVVLAGMDRYYCCESSRRQWIAT

QLVRRLADPDPCDYPDDQGPDADAPADWEQIRQRCLAVAVAMLEEAR

>mavi:RC58_00640 membrane protein; K20466 heme transporter (A)

MMRLSRSLRKYRWLVFAGWLLALVPAVYLALTQSGNLTGGGFDVAGSQSLAVHDQLEDLY

HDQGGSSLALVAAPRADASYQDMNDAVAQLRRIAAEVPGTTEIPNPTQRPPQPDRPYVLS

VRLDSRNTSDVAKQLRTKVGIKGDQPGQTANGRVRLYVIGQGALSAAAAANTKHDIAAAE

KWNLPVILIVLLAVFGSLAAAAIPLALGICTVVVTMGLVYLLSAYTTMSVFVTSTVSMFG

IALAVDYSLFILMRFREELRSGRQPREAVDAAMATSGLAVVLSGMTVIASLTGIYVINTP

ALKSMATGAILAVAVAMLTSTTLTPAALATFGRAAAKRSALLHWSRRPESTQSKFWNRWI

GWVMRRPWMSALAASLVLLVMAAPAASMVLGNSLLRQFDSSHEIRAGVGAAAQALGPGAL

GPIRVLINFPDGGAASPEHSHTVGAVRQRMAQAPNIVSVSPPQFAEDNGSALLSAVLSVD

PEDMKARETVGWMRAELPKVPEAGTARVDVGGPTALIKDFDDRVSATEPLVLGFVALIAF

VMLLVSIHSVFLALKGVLMTLLSVAAAYGSLVMVFQWGWLRDLGFAQISSIDSTVPPLVL

AMTFGLSMDYEIFLLTRIRERFLHSGNTRDAVAYGVSTSARTITSAALIMSAVFVGFAFA

GMPLVAEIGVACAVAIAVDATVVRLVMVPALMAMFAQWNWWLPPWLSRVLPSVDFDRPLP

EVDLGDVVVIPDDISALTAPSADLRMVLKSAAKLKHLAPDAICVTDPLAFTGCGRTTAAG

ADPARGLGPGQIPHQVALREEKVGVAAGPGEKTGSNGHTNGSAGAKKPAARNGRNGIAKA

IAGADRPVHPVTLWRGRLSVALDALQTDPDSGTDRPRFRRRSPVETTNVQLPTGDRLLVP

TGAETLRLKGYLLMCRNSRRDYADFADMVDALEPETAAVVLAGMDRYYCCESSRRQWIAT

QLVRRLADPDPCDYPDDQGPDADAPADWEQIRQRCLAVAVAMLEEAR

>mavu:RE97_00645 membrane protein; K20466 heme transporter (A)

MMRLSRSLRKYRWLVFAGWLLALVPAVYLALTQSGNLTGGGFDVAGSQSLAVHDQLEDLY

HDQGGSSLALVAAPRADASYQDMNDAVAQLRRIAAEVPGTTEIPNPTQRPPQPDRPYVLS

VRLDSRNTSDVAKQLRTKVGIKGDQPGQTANGRVRLYVIGQGALSAAAAANTKHDIAAAE

KWNLPVILIVLLAVFGSLAAAAIPLALGICTVVVTMGLVYLLSAYTTMSVFVTSTVSMFG

IALAVDYSLFILMRFREELRSGRQPREAVDAAMATSGLAVVLSGMTVIASLTGIYVINTP

ALKSMATGAILAVAVAMLTSTTLTPAALATFGRAAAKRSALLHWSRRPESTQSKFWNRWI

GWVMRRPWMSALAASLVLLVMAAPAASMVLGNSLLRQFDSSHEIRAGVGAAAQALGPGAL

GPIRVLINFPDGGAASPEHSHTVGAVRQRMAQAPNIVSVSPPQFAEDNGSALLSAVLSVD

PEDMKARETVGWMRAELPKVPEAGTARVDVGGPTALIKDFDDRVSATEPLVLGFVALIAF

VMLLVSIHSVFLALKGVLMTLLSVAAAYGSLVMVFQWGWLRDLGFAQISSIDSTVPPLVL

AMTFGLSMDYEIFLLTRIRERFLHSGNTRDAVAYGVSTSARTITSAALIMSAVFVGFAFA

GMPLVAEIGVACAVAIAVDATVVRLVMVPALMAMFAQWNWWLPPWLSRVLPSVDFDRPLP

EVDLGDVVVIPDDISALTAPSADLRMVLKSAAKLKHLAPDAICVTDPLAFTGCGRTTAAG

ADPARGLGPGQIPHQVALREEKVGVAAGPGEKTGSNGHTNGSAGAKKPAARNGRNGIAKA

IAGADRPVHPVTLWRGRLSVALDALQTDPDSGTDRPRFRRRSPVETTNVQLPTGDRLLVP

TGAETLRLKGYLLMCRNSRRDYADFADMVDALEPETAAVVLAGMDRYYCCESSRRQWIAT

QLVRRLADPDPCDYPDDQGPDADAPADWEQIRQRCLAVAVAMLEEAR

>mav:MAV_4973 MmpL11 protein; K20466 heme transporter (A)

MMRLSRSLRKYRWLVFAGWLLALVPAVYLALTQSGNLTGGGFDVAGSQSLAVHDQLEDLY

HDQGGSSLALVAAPRADASYQDMNDAVAQLRRIAAEVPGTTEIPNPTQRPPQPDRPYVLS

VRLDSRNTSDVAKQLRTKVGIKGDQPGQTANGRVRLYVIGQGALSAAAAANTKHDIAAAE

KWNLPVILIVLLAVFGSLAAAAIPLALGICTVVVTMGLVYLLSAYTTMSVFVTSTVSMFG

IALAVDYSLFILMRFREELRSGRQPREAVDAAMATSGLAVVLSGMTVIASLTGIYVINTP

ALKSMATGAILAVAVAMLTSTTLTPAALATFGRAAAKRSALLHWSRRPESTQSKFWNRWI

GWVMRRPWMSALAASLVLLVMAAPAASMVLGNSLLRQFDSSHEIRAGVGAAAQALGPGAL

GPIRVLINFPDGGAASPEHSHTVGAVRQRMAQAPNIVSVSPPQFAEDNGSALLSAVLSVD

PEDMKARETVGWMRAELPKVSEAGTARVDVGGPTALIKDFDDRVSATEPLVLGFVALIAF

VMLLVSIHSVFLALKGVLMTLLSVAAAYGSLVMVFQWGWLRDLGFAQISSIDSTVPPLVL

AMTFGLSMDYEIFLLTRIRERFLHSGNTRDAVAYGVSTSARTITSAALIMIAVFVGFAFA

GMPLVAEIGVACAVAIAVDATVVRLVMVPALMAMFAQWNWWLPPWLSRVLPSVDFDRPLP

EVDLGDVVVIPDDISALTAPSADLRMVLKSAAKLKHLAPDAICVTDPLAFTGCGRTTAAG

ADPARGLGPGQIPHQVALREEKVGVAAGPGEKTGSNGHTNGSAGAKKPAARNGRNGIAKA

IAGADRPVHPVTLWRGRLSVALDALQTDPDSGADRPRFRRRSPVETTNVQLPTGDRLLVP

TGAEALRLKGYLLMCRNSRRDYADFADMVDALEPETAAVVLAGMDRYYCCESSRRQWIAT

QLVRRLADPDPCDYPDDQGPDADAPADWEQIRQRCLAVAVAMLEEAR

>mavr:LA63_23290 membrane protein; K20466 heme transporter (A)

MMRLSRSLRKYRWLVFAGWLLALVPAVYLALTQSGNLTGGGFDVAGSQSLAVHDQLEDLY

HDQGGSSLALVAAPRADASYQDMNDAVAQLRRIAAEVPGTTEIPNPTQRPPQPDRPYVLS

VRLDSRNTSDVAKQLRTKVGIKGDQPGQTANGRVRLYVIGQGALSAAAAANTKHDIAAAE

KWNLPVILIVLLAVFGSLAAAAIPLALGICTVVVTMGLVYLLSAYTTMSVFVTSTVSMFG

IALAVDYSLFILMRFREELRSGRQPREAVDAAMATSGLAVVLSGMTVIASLTGIYVINTP

ALKSMATGAILAVAVAMLTSTTLTPAALATFGRAAAKRSALLHWSRRPESTQSKFWNRWI

GWVMRRPWMSALAASLVLLVMAAPAASMVLGNSLLRQFDSSHEIRAGVGAAAQALGPGAL

GPIRVLINFPDGGAASPEHSHTVGAVRQRMAQAPNIVSVSPPQFAEDNGSALLSAVLSVD

PEDMKARETVGWMRAELPKVSEAGTARVDVGGPTALIKDFDDRVSATEPLVLGFVALIAF

VMLLVSIHSVFLALKGVLMTLLSVAAAYGSLVMVFQWGWLRDLGFAQISSIDSTVPPLVL

AMTFGLSMDYEIFLLTRIRERFLHSGNTRDAVAYGVSTSARTITSAALIMIAVFVGFAFA

GMPLVAEIGVACAVAIAVDATVVRLVMVPALMAMFAQWNWWLPPWLSRVLPSVDFDRPLP

EVDLGDVVVIPDDISALTAPSADLRMVLKSAAKLKHLAPDAICVTDPLAFTGCGRTTAAG

ADPARGLGPGQIPHQVALREEKVGVAAGPGEKTGSNGHTNGSAGAKKPAARNGRNGIAKA

IAGADRPVHPVTLWRGRLSVALDALQTDPDSGADRPRFRRRSPVETTNVQLPTGDRLLVP

TGAEALRLKGYLLMCRNSRRDYADFADMVDALEPETAAVVLAGMDRYYCCESSRRQWIAT

QLVRRLADPDPCDYPDDQGPDADAPADWEQIRQRCLAVAVAMLEEAR

>mavd:NF84_23010 membrane protein; K20466 heme transporter (A)

MMRLSRSLRKYRWLVFAGWLLALVPAVYLALTQSGNLTGGGFDVAGSQSLAVHDQLEDLY

HDQGGSSLALVAAPRADASYQDMNDAVAQLRRIAAEVPGTTEIPNPTQRPPQPDRPYVLS

VRLDSRNTSDVAKQLRTKVGIKGDQPGQTANGRVRLYVIGQGALSAAAAANTKHDIAAAE

KWNLPVILIVLLAVFGSLAAAAIPLALGICTVVVTMGLVYLLSAYTTMSVFVTSTVSMFG

IALAVDYSLFILMRFREELRSGRQPREAVDAAMATSGLAVVLSGMTVIASLTGIYVINTP

ALKSMATGAILAVAVAMLTSTTLTPAALATFGRAAAKRSALLHWSRRPESTQSKFWNRWI

GWVMRRPWMSALAASLVLLVMAAPAASMVLGNSLLRQFDSSHEIRAGVGAAAQALGPGAL

GPIRVLINFPDGGAASPEHSHTVGAVRQRMAQAPNIVSVSPPQFAEDNGSALLSAVLSVD

PEDMKARETVGWMRAELPKVSEAGTARVDVGGPTALIKDFDDRVSATEPLVLGFVALIAF

VMLLVSIHSVFLALKGVLMTLLSVAAAYGSLVMVFQWGWLRDLGFAQISSIDSTVPPLVL

AMTFGLSMDYEIFLLTRIRERFLHSGNTRDAVAYGVSTSARTITSAALIMIAVFVGFAFA

GMPLVAEIGVACAVAIAVDATVVRLVMVPALMAMFAQWNWWLPPWLSRVLPSVDFDRPLP

EVDLGDVVVIPDDISALTAPSADLRMVLKSAAKLKHLAPDAICVTDPLAFTGCGRTTAAG

ADPARGLGPGQIPHQVALREEKVGVAAGPGEKTGSNGHTNGSAGAKKPAARNGRNGIAKA

IAGADRPVHPVTLWRGRLSVALDALQTDPDSGADRPRFRRRSPVETTNVQLPTGDRLLVP

TGAEALRLKGYLLMCRNSRRDYADFADMVDALEPETAAVVLAGMDRYYCCESSRRQWIAT

QLVRRLADPDPCDYPDDQGPDADAPADWEQIRQRCLAVAVAMLEEAR

>mava:LA64_23225 membrane protein; K20466 heme transporter (A)

MMRLSRSLRKYRWLVFAGWLLALVPAVYLALTQSGNLTGGGFDVAGSQSLAVHDQLEDLY

HDQGGSSLALVAAPRADASYQDMNDAVAQLRRIAAEVPGTTEIPNPTQRPPQPDRPYVLS

VRLDSRNTSDVAKQLRTKVGIKGDQPGQTANGRVRLYVIGQGALSAAAAANTKHDIAAAE

KWNLPVILIVLLAVFGSLAAAAIPLALGICTVVVTMGLVYLLSAYTTMSVFVTSTVSMFG

IALAVDYSLFILMRFREELRSGRQPREAVDAAMATSGLAVVLSGMTVIASLTGIYVINTP

ALKSMATGAILAVAVAMLTSTTLTPAALATFGRAAAKRSALLHWSRRPESTQSKFWNRWI

GWVMRRPWMSALAASLVLLVMAAPAASMVLGNSLLRQFDSSHEIRAGVGAAAQALGPGAL

GPIRVLINFPDGGAASPEHSHTVGAVRQRMAQAPNIVSVSPPQFAEDNGSALLSAVLSVD

PEDMKARETVGWMRAELPKVSEAGTARVDVGGPTALIKDFDDRVSATEPLVLGFVALIAF

VMLLVSIHSVFLALKGVLMTLLSVAAAYGSLVMVFQWGWLRDLGFAQISSIDSTVPPLVL

AMTFGLSMDYEIFLLTRIRERFLHSGNTRDAVAYGVSTSARTITSAALIMIAVFVGFAFA

GMPLVAEIGVACAVAIAVDATVVRLVMVPALMAMFAQWNWWLPPWLSRVLPSVDFDRPLP

EVDLGDVVVIPDDISALTAPSADLRMVLKSAAKLKHLAPDAICVTDPLAFTGCGRTTAAG

ADPARGLGPGQIPHQVALREEKVGVAAGPGEKTGSNGHTNGSAGAKKPAARNGRNGIAKA

IAGADRPVHPVTLWRGRLSVALDALQTDPDSGADRPRFRRRSPVETTNVQLPTGDRLLVP

TGAEALRLKGYLLMCRNSRRDYADFADMVDALEPETAAVVLAGMDRYYCCESSRRQWIAT

QLVRRLADPDPCDYPDDQGPDADAPADWEQIRQRCLAVAVAMLEEAR

>mit:OCO_48980 mmpL11 protein; K20466 heme transporter (A)

MMRLSRSLRKYRWLVFTGWLLALVPAVYLALTQSGNLTGGGFDVAGSQSLAVHDQLEELY

HDQGGSSLALVAAPRADATYQDMNEAVAQLRRLTAEVPGTSEIPNPTQRPPQPDRPYVLS

VRLDSRNTSDVAKKLRTKVGIKGDQPGQTANGRVRLYVIGQGALSAAAAANTKHDIAAAE

RWNLPVILIVLLAVFGSLAAAAIPLALGVCTVVVTMGLVYLLSAYTTMSVFVTSTVSMFG

IALAVDYSLFILMRFREELRSGRQPREAVDAAMATSGLAVVLSGATVVASLTGIYVINTP

ALKSMATGAILAVAVAMLTSTTLTPAALATFGRAAAKRSGFLHWSRRPESTQSRFWNRWI

GWVMRRPWMSALAASLVLLVMAAPAASMVLGNSLLRQFDSSHEIRAGVGAAAQALGPGAL

GPIRVLINFPEGGAASPEHSHTVGAVRQRMTQAPNIVSVSPPQYAEDNGSALLSAVLSVD

PEDMRARETVGWMRAELPKVQQLGTARVDVGGPTALIKDFDDQVSATEPLVLGFVALIAF

VMLLVSVHSVFLALKGVLMTLLSVAAAYGSLVMVFQWGWLKDLGFAQISSIDSTVPPLVL

AMTFGLSMDYEIFLLTRIRERFLHSGNTRDAVAYGVSTSARTITSAALIMIAVFVGFAFA

GMPLVAEIGVACAVAIAVDATVVRLVMVPALMAMFAQWNWWLPPWLSRVLPSVDFDRPLP

EVDLGDVVVIPDDISALVAPSADLRMVLKSAAKLKHLAPDAICVTDPLAFSGCGRGRGQA

SSGPDEERTRGLGPGQIPHQVALGEEKVAVGVTAADRKTATNGHANGSSGPKKLVGGLAA

RNGIAKAISGSDRPVHPVTLWRGRLSVAIDALETDPDAAPAGATDRPRYARRSPVETTHV

QLPTGDRLLVPTGAETLRLKGYLIMCRNSRRDYADFADMVDAMEPETAAVVLAGMDRYYC

CESSRRQCIATQLVRRLADPDPCDYPEDQGSEADANSDWEGIRERCLSVAVAMLEEAR

>mir:OCQ_49980 mmpL11 protein; K20466 heme transporter (A)

MMRLSRSLRKYRWLVFTGWLLALVPAVYLALTQSGNLTGGGFDVAGSQSLAVHDQLEELY

HDQGGSSLALVAAPRADATYQDMNEAVAQLRRLTAEVPGTSEIPNPTQRPPQPDRPYVLS

VRLDSRNTSDVAKKLRTKVGIKGDQPGQTANGRVRLYVIGQGALSAAAAANTKHDIAAAE

RWNLPVILIVLLAVFGSLAAAAIPLALGVCTVVVTMGLVYLLSAYTTMSVFVTSTVSMFG

IALAVDYSLFILMRFREELRSGRQPREAVDAAMATSGLAVVLSGATVVASLTGIYVINTP

ALKSMATGAILAVAVAMLTSTTLTPAALATFGRAAAKRSGFLHWSRRPESTQSRFWNRWI

GWVMRRPWMSALAASLVLLVMAAPAASMVLGNSLLRQFDSSHEIRAGVGAAAQALGPGAL

GPIRVLINFPEGGAASPEHSHTVGAVRQRMTQAPNIVSVSPPQYAEDNGSALLSAVLSVD

PEDMRARETVGWMRAELPKVQQLGTARVDVGGPTALIKDFDDQVSATEPLVLGFVALIAF

VMLLVSVHSVFLALKGVLMTLLSVAAAYGSLVMVFQWGWLKDLGFAQISSIDSTVPPLVL

AMTFGLSMDYEIFLLTRIRERFLHSGNTRDAVAYGVSTSARTITSAALIMIAVFVGFAFA

GMPLVAEIGVACAVAIAVDATVVRLVMVPALMAMFAQWNWWLPPWLSRVLPSVDFDRPLP

EVDLGDVVVIPDDISALVAPSADLRMVLKSAAKLKHLAPDAICVTDPLAFSGCGRGRGQA

SSGPDEERTRGLGPGQIPHQVALGEEKVAVGVTAADRKTATNGHANGSSGPKKLVGGLAA

RNGIAKAISGSDRPVHPVTLWRGRLSVAIDALETDPDAAPAGATDRPRYARRSPVETTHV

QLPTGDRLLVPTGAETLRLKGYLIMCRNSRRDYADFADMVDAMEPETAAVVLAGMDRYYC

CESSRRQCIATQLVRRLADPDPCDYPEDQGSEADANSDWEGIRERCLSVAVAMLEEAR

>mia:OCU_48920 mmpL11 protein; K20466 heme transporter (A)

MMRLSRSLRKYRWLVFTGWLLALVPAVYLALTQSGNLTGGGFDVAGSQSLAVHDQLEELY

HDQGGSSLALVAAPRADATYQDMNEAVAQLRRLTAEVPGTSEIPNPTQRPPQPDRPYVLS

VRLDSRNTSDVAKKLRTKVGIKGDQPGQTANGRVRLYVIGQGALSAAAAANTKHDIAAAE

RWNLPVILIVLLAVFGSLAAAAIPLALGVCTVVVTMGLVYLLSAYTTMSVFVTSTVSMFG

IALAVDYSLFILMRFREELRSGRQPREAVDAAMATSGLAVVLSGATVVASLTGIYVINTP

ALKSMATGAILAVAVAMLTSTTLTPAALATFGRAAAKRSGFLHWSRRPESTQSRFWNRWI

GWVMRRPWMSALAASLVLLVMAAPAASMVLGNSLLRQFDSSHEIRAGVGAAAQALGPGAL

GPIRVLINFPEGGAASPEHSHTVGAVRQRMTQAPNIVSVSPPQYAEDNGSALLSAVLSVD

PEDMRARETVGWMRGELPKVPQLGTARVDVGGPTALIKDFDDQVSATEPLVLGFVALIAF

VMLLVSVHSVFLALKGVLMTLLSVAAAYGSLVMVFQWGWLKDLGFAQISSIDSTVPPLVL

AMTFGLSMDYEIFLLTRIRERFLHSGNTRDAVAYGVSTSARTITSAALIMIAVFVGFAFA

GMPLVAEIGVACAVAIAVDATVVRLVMVPALMAMFAQWNWWLPPWLSRVLPSVDFDRPLP

EVDLGDVVVIPDDISALVAPSADLRMVLKSAAKLKHLAPDAICVTDPLAFSGCGRSGGQA

SSGPDEERTRGLGPGQIPHQVALGEEKVAVGVTAGDRKTATNGHANGSSGPKKLVGGLAA

RNGIAKAISGSDRPVHPVTLWRGRLSVAIDALETDPDAAPAGATDRPRYARRSPVETTHV

QLPTGDRLLVPTGAETLRLKGYLIMCRNSRRDYADFADMVDAMEPETAAVVLAGMDRYYC

CESSRRQCIATQLVRRLADPDPCDYPEDQGSEADANSDWEGIRERCLSVAVAMLEEAR

>mie:LG41_23200 membrane protein; K20466 heme transporter (A)

MMRLSRSLRKYRWLVFTGWLLALVPAVYLALTQSGNLTGGGFDVAGSQSLAVHDQLEELY

HDQGGSSLALVAAPRADATYQDMNEAVAQLRRLTAEVPGTSEIPNPTQRPPQPDRPYVLS

VRLDSRNTSDVAKKLRTKVGIKGDQPGQTANGRVRLYVIGQGALSAAAAANTKHDIAAAE

RWNLPVILIVLLAVFGSLAAAAIPLALGVCTVVVTMGLVYLLSAYTTMSVFVTSTVSMFG

IALAVDYSLFILMRFREELRSGRQPREAVDAAMATSGLAVVLSGATVVASLTGIYVINTP

ALKSMATGAILAVAVAMLTSTTLTPAALATFGRAAAKRSGFLHWSRRPESTQSRFWNRWI

GWVMRRPWMSALAASLVLLVMAAPAASMVLGNSLLRQFDSSHEIRAGVGAAAQALGPGAL

GPIRVLINFPEGGAASPEHSHTVGAVRQRMTQAPNIVSVSPPQYAEDNGSALLSAVLSVD

PEDMRARETVGWMRAELPKVQQLGTARVDVGGPTALIKDFDDQVSATEPLVLGFVALIAF

VMLLVSVHSVFLALKGVLMTLLSVAAAYGSLVMVFQWGWLKDLGFAQISSIDSTVPPLVL

AMTFGLSMDYEIFLLTRIRERFLHSGNTRDAVAYGVSTSARTITSAALIMIAVFVGFAFA

GMPLVAEIGVACAVAIAVDATVVRLVMVPALMAMFAQWNWWLPPWLSRVLPSVDFDRPLP

EVDLGDVVVIPDDISALVAPSADLRMVLKSAAKLKHLAPDAICVTDPLAFSGCGRGRGQA

SSGPDEERTRGLGPGQIPHQVALGEEKVAVGVTAADRKTATNGHANGSSGPKKLVGGLAA

RNGIAKAISGSDRPVHPVTLWRGRLSVAIDALETDPDAAPAGATDRPRYARRSPVETTHV

QLPTGDRLLVPTGAETLRLKGYLIMCRNSRRDYADFADMVDAMEPETAAVVLAGMDRYYC

CESSRRQCIATQLVRRLADPDPCDYPEDQGSEADANSDWEGIRERCLSVAVAMLEEAR

>mid:MIP_07415 Putative membrane protein mmpL11; K20466 heme transporter (A)

MMRLSRSLRKYRWLVFTGWLLALVPAVYLALTQSGNLTGGGFDVAGSQSLAVHDQLEELY

HDQGGSSLALVAAPRADATYQDMNEAVAQLRRLTAEVPGTSEIPNPTQRPPQPDRPYVLS

VRLDSRNTSDVAKKLRTKVGIKGDQPGQTANGRVRLYVIGQGALSAAAAANTKHDIAAAE

RWNLPVILIVLLAVFGSLAAAAIPLALGVCTVVVTMGLVYLLSAYTTMSVFVTSTVSMFG

IALAVDYSLFILMRFREELRSGRQPREAVDAAMATSGLAVVLSGATVVASLTGIYVINTP

ALKSMATGAILAVAVAMLTSTTLTPAALATFGRAAAKRSGFLHWSRRPESTQSRFWNRWI

GWVMRRPWMSALAASLVLLVMAAPAASMVLGNSLLRQFDSSHEIRAGVGAAAQALGPGAL

GPIRVLINFPEGGAASPEHSHTVGAVRQRMTQAPNIVSVSPPQYAEDNGSALLSAVLSVD

PEDMRARETVGWMRAELPKVQQLGTARVDVGGPTALIKDFDDQVSATEPLVLGFVALIAF

VMLLVSVHSVFLALKGVLMTLLSVAAAYGSLVMVFQWGWLKDLGFAQISSIDSTVPPLVL

AMTFGLSMDYEIFLLTRIRERFLHSGNTRDAVAYGVSTSARTITSAALIMIAVFVGFAFA

GMPLVAEIGVACAVAIAVDATVVRLVMVPALMAMFAQWNWWLPPWLSRVLPSVDFDRPLP

EVDLGDVVVIPDDISALVAPSADLRMVLKSAAKLKHLAPDAICVTDPLAFSGCGRGRGQA

SSGPDEERTRGLGPGQIPHQVALGEEKVAVGVTAADRKTATNGHANGSSGPKKLVGGLAA

RNGIAKAISGSDRPVHPVTLWRGRLSVAIDALETDPDAAPAGATDRPRYARRSPVETTHV

QLPTGDRLLVPTGAETLRLKGYLIMCRNSRRDYADFADMVDAMEPETAAVVLAGMDRYYC

CESSRRQCIATQLVRRLADPDPCDYPEDQGSEADANSDWEGIRERCLSVAVAMLEEAR

>myo:OEM_49130 mmpL11 protein; K20466 heme transporter (A)

MMRLSRSLRKYRWLVFTGWLLALVPAVYLALTQSGNLTGGGFDVAGSQSLAVHDQLEELY

HDQGGSSLALVAAPRADATYQDMNEAVAQLRRLTAEVPGTSEIPNPTQRPPQPDRPYVLS

VRLDSRNTSDVAKKLRTKVGIKGDQPGQTANGRVRLYVIGQGALSAAAAANTKHDIAAAE

RWNLPVILIVLLAVFGSLAAAAIPLALGVCTVVVTMGLVYLLSAYTTMSVFVTSTVSMFG

IALAVDYSLFILMRFREELRSGRQPREAVDAAMATSGLAVVLSGATVVASLTGIYVINTP

ALKSMATGAILAVAVAMLTSTTLTPAALATFGRAAAKRSGFLHWSRRPESTQSRFWNRWI

GWVMRRPWMSALAASLVLLVMAAPAASMVLGNSLLRQFDSSHEIRAGVGAAAQALGPGAL

GPIRVLINFPEGGAASPEHSHTVGAVRQRMTQAPNIVSVSPPQYAEDNGSALLSAVLSVD

PEDMRARETVGWMRAELPKVPQLGTARVDVGGPTALIKDFDDQVSATEPLVLGFVALIAF

VMLLVSVHSVFLALKGVLMTLLSVAAAYGSLVMVFQWGWLKDLGFAQISSIDSTVPPLVL

AMTFGLSMDYEIFLLTRIRERFLHSGNTRDAVAYGVSTSARTITSAALIMIAVFVGFAFA

GMPLVAEIGVACAVAIAVDATVVRLVMVPALMAMFAQWNWWLPSWLSRVLPSVDFDRPLP

EVDLGDVVVIPDDISALVAPSADLRMVLKSAAKLKHLAPDAICVTDPLAFSGCGRGRGQA

SSGPDEERTRGLGPGQIPHQVALGEEKVAVGVTAGERKTATNGHANGSSGPKKLVGGLAA

RNGIAKAISGSDRPVHPVTLWRGRLSVAIDALETDPDAAPAGATDRPRYARRSPVETTHV

QLPTGDRLLVPTGAETLRLKGYLIMCRNSRRDYADFADMVDAMEPETAAVVLAGMDRYYC

CESSRRQCIATQLVRRLADPDPCDYPEDQGSEADANSDWEGIRERCLSVAVAMLEEAR

>msm:MSMEG_0241 MmpL11 protein; K20466 heme transporter (A)

MMRLSSTLRRFRWAVFATWLLLLVPSIYLALNQSSNLTGGGFEVEGSQSLHVQRQLEEHF

PDQGASPLALVAAPRADASYEDMNAAVVHLEKLAAEVPSVKIVPNPQQPAPQPDRPYVIT

LQLDFNNTGAVDVAKQLRQKVGIHGEEPGESQNGKVKFYVIGQGALGAAATQATKHDIAA

AEKWNMPIVLIVLLAVFGSLAAAALPLVLGVCTVVVTMGLVYLLSMFTTMSVFVTSTVSM

FGIAVAIDYSLFILMRFREELRAGRDQQDAIDAAMATSGLAVALSGLTVIASVTGIYLIN

TPVLVSMATGAILAVAVAVLTSTTLTPAVLATFGKAAAKRSSYLHWSRRAEAAQSRFWTR

WTGAVMRRPWASAIAAAILLLVLAAPAFNMVLGNSMQRQFDPTHEIRGGVNAAADALGPG

ALGPIRVLVTFPGEGDASSQAATTTIEAVRQQMTKAPSVVSVQPPVVSDDNDSALLSAVL

SVDPEDMAAREAIDWMRAELPGVAGQNATIDVGGPTALIKDFDDRVSATQPLVFVFVALI

AFVMLLVSIRSVVLAFKGVLMTVLSVAAAYGSLVVVFQWGWLEQLGFPRISSLDSTIPPL

VLAMTFGLSMDYEIFLLTRIRERFLQTNSTRDAVAYGVSTSARTITSAALIMIAVFIGFA

FAGMPLVAQLGVACAVAIAVDATVVRLVLVPALMAMFDQWNWWLPRWLDKILPEVDFEKP

LPKIEVTDLVIIPDNIAALGPSGSDLRTMVRTAARMKTLAPQTISVADPLAFSGCTRPTT

RLSTQRAGRPKAHTPGLHPVTMWRGRLSVAVDALQTEADTEQAPVERRGPVETTNVQLPT

GDRLQIPTGAETLRLAGYLIMCRNTTKDFEDFARLVDLMDSHTAALVLASMDRYYCGRDP

SNRWVATQLVRRLADPQPSDEHDVRMSGPDAAEDWEKVRQRCLSVAVAMLEEAK

>msg:MSMEI_0234 mmpL11; Transmembrane transport protein mmpL11; K20466 heme transporter (A)

MMRLSSTLRRFRWAVFATWLLLLVPSIYLALNQSSNLTGGGFEVEGSQSLHVQRQLEEHF

PDQGASPLALVAAPRADASYEDMNAAVVHLEKLAAEVPSVKIVPNPQQPAPQPDRPYVIT

LQLDFNNTGAVDVAKQLRQKVGIHGEEPGESQNGKVKFYVIGQGALGAAATQATKHDIAA

AEKWNMPIVLIVLLAVFGSLAAAALPLVLGVCTVVVTMGLVYLLSMFTTMSVFVTSTVSM

FGIAVAIDYSLFILMRFREELRAGRDQQDAIDAAMATSGLAVALSGLTVIASVTGIYLIN

TPVLVSMATGAILAVAVAVLTSTTLTPAVLATFGKAAAKRSSYLHWSRRAEAAQSRFWTR

WTGAVMRRPWASAIAAAILLLVLAAPAFNMVLGNSMQRQFDPTHEIRGGVNAAADALGPG

ALGPIRVLVTFPGEGDASSQAATTTIEAVRQQMTKAPSVVSVQPPVVSDDNDSALLSAVL

SVDPEDMAAREAIDWMRAELPGVAGQNATIDVGGPTALIKDFDDRVSATQPLVFVFVALI

AFVMLLVSIRSVVLAFKGVLMTVLSVAAAYGSLVVVFQWGWLEQLGFPRISSLDSTIPPL

VLAMTFGLSMDYEIFLLTRIRERFLQTNSTRDAVAYGVSTSARTITSAALIMIAVFIGFA

FAGMPLVAQLGVACAVAIAVDATVVRLVLVPALMAMFDQWNWWLPRWLDKILPEVDFEKP

LPKIEVTDLVIIPDNIAALGPSGSDLRTMVRTAARMKTLAPQTISVADPLAFSGCTRPTT

RLSTQRAGRPKAHTPGLHPVTMWRGRLSVAVDALQTEADTEQAPVERRGPVETTNVQLPT

GDRLQIPTGAETLRLAGYLIMCRNTTKDFEDFARLVDLMDSHTAALVLASMDRYYCGRDP

SNRWVATQLVRRLADPQPSDEHDVRMSGPDAAEDWEKVRQRCLSVAVAMLEEAK

>msb:LJ00_01210 membrane protein; K20466 heme transporter (A)

MMRLSSTLRRFRWAVFATWLLLLVPSIYLALNQSSNLTGGGFEVEGSQSLHVQRQLEEHF

PDQGASPLALVAAPRADASYEDMNAAVVHLEKLAAEVPSVKIVPNPQQPAPQPDRPYVIT

LQLDFNNTGAVDVAKQLRQKVGIHGEEPGESQNGKVKFYVIGQGALGAAATQATKHDIAA

AEKWNMPIVLIVLLAVFGSLAAAALPLVLGVCTVVVTMGLVYLLSMFTTMSVFVTSTVSM

FGIAVAIDYSLFILMRFREELRAGRDQQDAIDAAMATSGLAVALSGLTVIASVTGIYLIN

TPVLVSMATGAILAVAVAVLTSTTLTPAVLATFGKAAAKRSSYLHWSRRAEAAQSRFWTR

WTGAVMRRPWASAIAAAILLLVLAAPAFNMVLGNSMQRQFDPTHEIRGGVNAAADALGPG

ALGPIRVLVTFPGEGDASSQAATTTIEAVRQQMTKAPSVVSVQPPVVSDDNDSALLSAVL

SVDPEDMAAREAIDWMRAELPGVAGQNATIDVGGPTALIKDFDDRVSATQPLVFVFVALI

AFVMLLVSIRSVVLAFKGVLMTVLSVAAAYGSLVVVFQWGWLEQLGFPRISSLDSTIPPL

VLAMTFGLSMDYEIFLLTRIRERFLQTNSTRDAVAYGVSTSARTITSAALIMIAVFIGFA

FAGMPLVAQLGVACAVAIAVDATVVRLVLVPALMAMFDQWNWWLPRWLDKILPEVDFEKP

LPKIEVTDLVIIPDNIAALGPSGSDLRTMVRTAARMKTLAPQTISVADPLAFSGCTRPTT

RLSTQRAGRPKAHTPGLHPVTMWRGRLSVAVDALQTEADTEQAPVERRGPVETTNVQLPT

GDRLQIPTGAETLRLAGYLIMCRNTTKDFEDFARLVDLMDSHTAALVLASMDRYYCGRDP

SNRWVATQLVRRLADPQPSDEHDVRMSGPDAAEDWEKVRQRCLSVAVAMLEEAK

>msn:LI99_01210 membrane protein; K20466 heme transporter (A)

MMRLSSTLRRFRWAVFATWLLLLVPSIYLALNQSSNLTGGGFEVEGSQSLHVQRQLEEHF

PDQGASPLALVAAPRADASYEDMNAAVVHLEKLAAEVPSVKIVPNPQQPAPQPDRPYVIT

LQLDFNNTGAVDVAKQLRQKVGIHGEEPGESQNGKVKFYVIGQGALGAAATQATKHDIAA

AEKWNMPIVLIVLLAVFGSLAAAALPLVLGVCTVVVTMGLVYLLSMFTTMSVFVTSTVSM

FGIAVAIDYSLFILMRFREELRAGRDQQDAIDAAMATSGLAVALSGLTVIASVTGIYLIN

TPVLVSMATGAILAVAVAVLTSTTLTPAVLATFGKAAAKRSSYLHWSRRAEAAQSRFWTR

WTGAVMRRPWASAIAAAILLLVLAAPAFNMVLGNSMQRQFDPTHEIRGGVNAAADALGPG

ALGPIRVLVTFPGEGDASSQAATTTIEAVRQQMTKAPSVVSVQPPVVSDDNDSALLSAVL

SVDPEDMAAREAIDWMRAELPGVAGQNATIDVGGPTALIKDFDDRVSATQPLVFVFVALI

AFVMLLVSIRSVVLAFKGVLMTVLSVAAAYGSLVVVFQWGWLEQLGFPRISSLDSTIPPL

VLAMTFGLSMDYEIFLLTRIRERFLQTNSTRDAVAYGVSTSARTITSAALIMIAVFIGFA

FAGMPLVAQLGVACAVAIAVDATVVRLVLVPALMAMFDQWNWWLPRWLDKILPEVDFEKP

LPKIEVTDLVIIPDNIAALGPSGSDLRTMVRTAARMKTLAPQTISVADPLAFSGCTRPTT

RLSTQRAGRPKAHTPGLHPVTMWRGRLSVAVDALQTEADTEQAPVERRGPVETTNVQLPT

GDRLQIPTGAETLRLAGYLIMCRNTTKDFEDFARLVDLMDSHTAALVLASMDRYYCGRDP

SNRWVATQLVRRLADPQPSDEHDVRMSGPDAAEDWEKVRQRCLSVAVAMLEEAK

>msh:LI98_01210 membrane protein; K20466 heme transporter (A)

MMRLSSTLRRFRWAVFATWLLLLVPSIYLALNQSSNLTGGGFEVEGSQSLHVQRQLEEHF

PDQGASPLALVAAPRADASYEDMNAAVVHLEKLAAEVPSVKIVPNPQQPAPQPDRPYVIT

LQLDFNNTGAVDVAKQLRQKVGIHGEEPGESQNGKVKFYVIGQGALGAAATQATKHDIAA

AEKWNMPIVLIVLLAVFGSLAAAALPLVLGVCTVVVTMGLVYLLSMFTTMSVFVTSTVSM

FGIAVAIDYSLFILMRFREELRAGRDQQDAIDAAMATSGLAVALSGLTVIASVTGIYLIN

TPVLVSMATGAILAVAVAVLTSTTLTPAVLATFGKAAAKRSSYLHWSRRAEAAQSRFWTR

WTGAVMRRPWASAIAAAILLLVLAAPAFNMVLGNSMQRQFDPTHEIRGGVNAAADALGPG

ALGPIRVLVTFPGEGDASSQAATTTIEAVRQQMTKAPSVVSVQPPVVSDDNDSALLSAVL

SVDPEDMAAREAIDWMRAELPGVAGQNATIDVGGPTALIKDFDDRVSATQPLVFVFVALI

AFVMLLVSIRSVVLAFKGVLMTVLSVAAAYGSLVVVFQWGWLEQLGFPRISSLDSTIPPL

VLAMTFGLSMDYEIFLLTRIRERFLQTNSTRDAVAYGVSTSARTITSAALIMIAVFIGFA

FAGMPLVAQLGVACAVAIAVDATVVRLVLVPALMAMFDQWNWWLPRWLDKILPEVDFEKP

LPKIEVTDLVIIPDNIAALGPSGSDLRTMVRTAARMKTLAPQTISVADPLAFSGCTRPTT

RLSTQRAGRPKAHTPGLHPVTMWRGRLSVAVDALQTEADTEQAPVERRGPVETTNVQLPT

GDRLQIPTGAETLRLAGYLIMCRNTTKDFEDFARLVDLMDSHTAALVLASMDRYYCGRDP

SNRWVATQLVRRLADPQPSDEHDVRMSGPDAAEDWEKVRQRCLSVAVAMLEEAK

>msa:Mycsm_00129 putative RND superfamily drug exporter; K20466 heme transporter (A)

MRLSSNLRRYRWAVFAVWLLLLVPSIYLAMNQSGNLTGGGFEVEGSQSLHVQRELEQHFP

GQGASPLALVAAPRADASFEDMTAAVAQLERIAAEVPSVKVAPNPQQPPPQPDRPYVVTL

QLDFNNTGAVDVAKQLRQKVGITGDQPGEIENGKVKLYVIGQGALGAAATLATKHDIAQA

EQWNLPIVLIVLLAVFGSLAAAAMPLVLGICTVVVTMGLVYLLSMYTTMSVFVTSTVSMF

GIALAIDYSLFILMRFREELRAGRDPQQAADAAMATSGLAVVLSGLTVIASVTGIYLINT

PVLQSMATGAILAVAVAVLTSTTLTPAVLATFGRAAAKRSSYLHWSRRVESTQSRFWSRW

TGWVMRRPWVSALLAAAFLLTLAAPAFSMVLGNSMQRQFEPTHEIRGGVNAAAEALGPGA

LGPVRVLVTFPDGNAASAAAKQPALEAVRQKMAQGPHVVTVSPPVFGDDYRRALLSAVLS

VDPEDMGARDTVDWMRAQLPQTDTGARIDVGGPTALIKDFDDRVSKTQPMVFGFVALIAF

VMLLISIRSVFLAFKGVLMTVLSVAAAYGSLVAVFQWGWLEDLGFKPISSLDSTIPPLVL

AMTFGLSMDYEIFLLTRIRERFLQTKNTRDAVAYGVSTSARTITSAALIMIAVFIGFAFA

GMPLVAQLGVACAVAIAVDATVVRLVLVPALMAMFDEWNWWLPRWLARILPSVDFEKPIP

KVDLPDLVIIPDDISSLGPTGSDLRMVVKSAARLKSLAPDTITVADPLALGVAPVTQPMP

AIVNGGAPRNGATNGNGVKKNGKPGPHSPTEMHPVTIWRGRLSVALDALETEADSERAPV

ARSSPVETTNVQLPTGDRLQVPTGAETLRLKSYLVMCRNTTRDFAEFAELVDSMETHTAA

VVLASMDRYYCGDRSRKQWVATQLVRRLADPQPSDEHDTRMSGPEAEADWAKVRERCLSV

AVAMLEEAR

>mul:MUL_1092 mmpL11; conserved transmembrane transport protein MmpL11; K20466 heme transporter (A)

MMRLSRNLRRFRWLVFTGWMLALVPAIYLALTQSGNLTGGGFEVAGSQSLRVHDVLEAQY

PDQGASSLALVATPRPDASYQDITDAVAQLRRIAGEFPGVSEVPDPTQRPPQPDRPYVVS

LRLDARNAGTSDLAKKLRARVGVEGDKPGQTADGRVRLYVIGQGALSAAAAANTKHDIAA

AERWNLPIILIVLLAVFGSLTAAAIALALGICTVVVTMGLVYLLSMHTTMSVFVTSTVSM

FGIALAVDYSLFILMRFREELRSGRQPQEAADAAMATSGLAVVLSGMTVVASLSGIYLIN

TPALRSMATGAILAVAVAILTSATLTPAVLATFARAAAKRSALLHWSRRPESTQSRFWTR

WVGWVMHRPWITALSASVVLLLMAAPAASMVLGNSLLRQFDSSHEIRAGVSAAAQALGPG

ALGPVQVLIKFPDGGASTPAHSQTVGAVRQRMAQGPNVTTVAPPKFAEDNDSALLSAVLS

VDPEDMGARHTVDWMRSELPQVAGGAQFDVGGSTALIKDFDDRVSATEPLVLAFVALIAF

VMLLISIHSVLLALKGVLMTLLSVAAAYGSLVMVFQWGWGERLGFPQLTSIDSTVPPLVL

AMTFGLSMDYEIFLLTRIRERFLHTGRTRDAVAYGVSTSARTITSAALIMIAVFCGFAFA

GMPLVAEIGVACAVAIAVDATIVRLIMVPALMAMFSQWNWWLPRWLGRILPSVDFDRPLP

EVDLADVVVIPEDIAATIPPGADLRMVLKSAAKLKQLAPDTICVADPLAFTGCEGVGKGP

ERIPELVKDGLNLAGGTDTAGPGGGAEAKNGSKGQSAARKAVGLAYRSGIARAMSWAERP

VHPVTVWRGRLSIALDALETNASERRAGPGSGREPNYRRRSPVETTNVQLPTGDRLLIPT

GAETLRLKGYLIMSRNSSRDYAELADMVEAMEPETAAVVLAGMDRYYCCQPPRRQWIATQ

LVRRLADPHPSDVDVEWPQPDDKADWEEVRQRCLAVAVAMLEEAR

>mva:Mvan_0188 MmpL11; K20466 heme transporter (A)

MLRVSSNLRRFRWAVFAVWLLLLVPSIYLALNQSSHLTGGGFEVEGSQSLYVQRQLEAQF

PDQGASPLALVAAPRSDASYDDMTSAVSHLERLAAEVPSVTVVPTPQQPPPRPDRPYVIT

LQLDFENTGAVDVAKQLREKVGVDGEEPGEMENGKVRLYVIGQGALGAAATLATKHDIAQ

AEKWNFPIVLVILLAVFGSLAAAAMPLLLGVCTVVVTMGLVFLLSMYTTMSVFVTSTVSM

FGIAVAIDYSLFILMRFREELRAGREPEDAADAAMATSGLAVLLSGLTVIASVTGIYLIN

TPVLQSMATGAILAVAVAVLTSTTLTPAVLATFGRRAAKRSSYLHWGRGVEATQSKFWSR

WTGWVMRRPWVSATAAAAVLLAFAAPAFSMMLGNSMQRQFDPTHEIRGGVNAAAEALGPG

ALGPIRVLVTFPVDDGAEAAAPLLESLRQKMGQAPNTVSVSPPVFGSDGRSALLSAVLSV

DPEDMGARDTVDWMREQLPPVAGDAARVDVGGPTALIKDFDDRVSQTQPLVFVFVALIAF

VMLLVSIRSVFLAFKGVLMTVLSVAAAYGSLVVVFQWGWLADLGFKQISSLDSTIPPLVL

ALTFGLSMDYEIFLLTRIRERFLQTGNTRDAVAYGVSTSARTITSAALIMIAVFIGFAFA

GMPLVAQLGVACAVAIAVDATIVRLVLVPALMAMFDEWNWWLPRWLDRILPSVDFEKPLP

KADIGDLVIIPDDISALAPSGSDLRTVVKSAAKLKTLAPQAITVADPLAFSGCLPCGKIS

GNRVGRDERITAAVTNRAHGKTVAVKLPKHPVTMWRGRLDVALDALAVEQSAGNGEPRIE

RISPMETTNVLLPTGDRLQIPTGAETLRLKGFLIMSRNTARDYRDFTELVDSMDVDTAAD

VLAGMDRYYAGQPARAHWVATQLVRRLAEPLPSDGPDIAAAGADEETEWAKVRQRCLSVA

VAMLEETR

>mgi:Mflv_0464 MmpL11; K20466 heme transporter (A)

MMRVSSNLRRFRWAVFAVWLLLLLPSVYLAMNQAGNLTGGGFEVEGSQSLYVQRQLEAQF

PDQGASPLALVAAPRPDASFEDMNNAVAQLESLAAEIPSITVVPSPQQPPPQPDRPYVVT

LAMDFENTGAVDIAKQLREKVGVDGEDAGETENGKVRLYVIGQGALGAAATEATKDDIAQ

AEKWNFPIVLVILLAVFGSLAAAAMPLLLGVCTVVVTMGVVFVLSMYTTMSVFVTSTVSM

FGIAVAIDYSLFILMRFREELRAGREPEDAADAAMATSGLAVVLSGLTVIASVTGIYLIN

TPVLQSMATGAILAVAIAVLTSTTLTPAVLATFGRRAAKRSSYLHWGRGVEATQSKFWTR

WTGWVMRRPWLSAMAAAAVLLTFAAPAFSMMLGNSMQRQFDTTHEIRGGVSAAAEALGPG

ALGPIRVLITFPDGDAASASAKEPLLAELRQTMAGAPNVVSVTPPVFGTDYRIALLSAVL

SVDPEDMGARETIDWMREKLPPVAGDAASIDVGGPTALIKDFDDKVSATQPLVFAFVALI

AFVMLLVSIRSVFLAFKGVLMTVLSVAAAYGSLVVVFQWGWLSSLGFDQLSSLDSTIPPL

VLALTFGLSMDYEIFLLTRIRERFLQTGNTRDAVAYGVSTSARTITSAALIMIAVFIGFA

FAGMPLVAQLGVACAVAIAVDATIVRLVLVPALMAMFDEWNWWLPRWLDRLLPSVDFEKP

LPKADIGDLVIIPDDISALAPSGSDLRTVVKSAAKLKTLVPQAVTVADPLAFSGCLPCNK

MTGTRVGRDERITAAVSNRAHGKTVAVKLPKHPVTMWRGRLDVALEALATERAQNEGHPT

IERISPLETTNVLLPTGDRLQIPTGAETLRLKSYLILERNSTRDFSEFSELVDSMDTSTA

AEVLASMDRYYSGQSARDQQAGARTRHWVATQLVRRLAEPAPADGPDIAAAGADGEAEWA

TVRQRCLSVAVAMLEETR

>msp:Mspyr1_02910 predicted RND superfamily drug exporter; K20466 heme transporter (A)

MRVSSNLRRFRWAVFAVWLLLLLPSVYLAMNQAGNLTGGGFEVEGSQSLYVQRQLEAQFP

DQGASPLALVAAPRPDASFEDMNNAVAQLESLAAEIPSITVVPSPQQPPPQPDRPYVVTL

AMDFENTGAVDIAKQLREKVGVDGEDAGETENGKVRLYVIGQGALGAAATEATKDDIAQA

EKWNFPIVLVILLAVFGSLAAAAMPLLLGVCTVVVTMGVVFVLSMYTTMSVFVTSTVSMF

GIAVAIDYSLFILMRFREELRAGREPEDAADAAMATSGLAVVLSGLTVIASVTGIYLINT

PVLQSMATGAILAVAIAVLTSTTLTPAVLATFGRRAAKRSSYLHWGRGVEATQSKFWTRW

TGWVMRRPWLSAMAAAAVLLTFAAPAFSMMLGNSMQRQFDTTHEIRGGVSAAAEALGPGA

LGPIRVLITFPDGDAASASAKEPLLAELRQTMAGAPNVVSVTPPVFGTDYRIALLSAVLS

VDPEDMGARETIDWMREKLPPVAGDAASIDVGGPTALIKDFDDKVSATQPLVFAFVALIA

FVMLLVSIRSVFLAFKGVLMTVLSVAAAYGSLVVVFQWGWLSSLGFDQLSSLDSTIPPLV

LALTFGLSMDYEIFLLTRIRERFLQTGNTRDAVAYGVSTSARTITSAALIMIAVFIGFAF

AGMPLVAQLGVACAVAIAVDATIVRLVLVPALMAMFDEWNWWLPRWLDRLLPSVDFEKPL

PKADIGDLVIIPDDISALAPSGSDLRTVVKSAAKLKTLVPQAVTVADPLAFSGCLPCNKM

TGTRVGRDERITAAVSNRAHGKTVAVKLPKHPVTMWRGRLDVALEALATERAQNEGHPTI

ERISPLETTNVLLPTGDRLQIPTGAETLRLKSYLILERNSTRDFSEFSELVDSMDTSTAA

EVLASMDRYYSGQSARDQQAGARTRHWVATQLVRRLAEPAPADGPDIAAAGADGEAEWAT

VRQRCLSVAVAMLEETR

>mab:MAB_4529 Putative membrane protein, MmpL; K20466 heme transporter (A)

MMRTSDYLRRYRWLTVAVWLILIVAAVGLVAGRGEKLTGGGFEVAGSQSLKVHDAMETGF

REQGASPLALVAAPRPDATVSDMAAAVDYLKQVAAQIPQVSVEPIPPQLTPQPDRPFVVT

LRIGFDNTGATDIAKSLRKKIGVHGGQPGQIAGGRVSLYVIGQGALGAAMTEQIAGDIKK

AEKWNIPVILIVLIAAFGSLAAASLPLLLGVCTVALSVAIVLALSNITTISVFALPTVTM

IGLAVAVDYSLFILMRYREELNSGKNRQEAIAGAMATSGLTVTFSGLTVIAALAGIYLIG

TPALASMASGAIIVVAIAVLTSTTLMPALLAIFGKAAANQSRFLRVSPLRKHSIPFWRKW

TQQVMRRPWLSALGASIFLLALAAPSLQMHVSNSMLRQFDSSHEIRRGIDAAAVAMGPGA

LGPVQVLLTFKDSPAHAHAQALETAKETIAQAPNIARVSDVSFSTDGHDALISAVLSVDP

EANAARDTVSWLRANVPDALENSGADINVGGPTALLFDYDTRVEHSLLGVFAFVCVLAFV

MLLATLRSPVLALKGVVMTVLSVAAAYGSLVIVFQWGWLEFLGFHKANIDSSIPPLALAL

TFGLTMDYEIFLLARVRERYLRTGDCGDAVSYGVRTSARTITSAALIMIAVFIGFAFVGM

PLVAQLGVACAVAIAVDATVVRLVLVPALMAMFDKRNWWLPGWLDHVLPTVDFETPCVDE

LPGPHSESALRLQGSGFEEPRIDYHAIAVSAARLKRLADGGTITAPDTQAVLADRWRDNL

TVALEALQTSKNGTDTDIVEMPGPLSRVTPVEITQVKLPTGQKCVVPTDAEALRMQGLLL

MRRNAERDYTEFARLASAMEPRMAAKVLAGIDQHYCVQSNQRWVSSQLVRRLADPKPGDG

AMECPTVRRQCLSVAVAMLEDAR

>mabb:MASS_4555 putative membrane protein, MmpL; K20466 heme transporter (A)

MQSVMMRTSDYLRRYRWLTVAVWLILIVAAVGLVAGRGEKLTGGGFEVAGSQSLKVHDAM

ETGFREQGASPLALVAAPRPDATVSDMAAAVDYLKQVAAQIPQVSVEPIPPQLTPQPDRP

FVVTLRIGFDNTGATDIAKSLRKKIGVQGGQPGQIAGGRVSLYVIGQGALGAAMTEQIAG

DIKKAEKWNIPVILIVLIAAFGSLAAASLPLLLGVCTVALSVAIVLALSNITTISVFALP

TVTMIGLAVAVDYSLFILMRYREELNSGKNRQEAIAGAMATSGLTVTFSGLTVIAALAGI

YLIGTPALASMASGAIIVVAIAVLTSTTLMPALLAIFGKAAANQSRFLRVSPLRKHSIPF

WRKWTQQVMRRPWLSALGASIFLLALAAPSLQMHVSNSMLRQFDSSHEIRRGIDAAAVAM

GPGALGPVQVLLTFKDSPAHAHAQALETAKETIAQAPNIARVSDVSFSTDGHDALISAVL

SVDPEANAARDTVSWLRANVPDALENSGADINVGGPTALLFDYDTRVEHSLLGVFAFVCV

LAFVMLLATLRSPVLALKGVVMTVLSVAAAYGSLVIVFQWGWLEFLGFHKANIDSSIPPL

ALALTFGLTMDYEIFLLARVRERYLRTGDCGDAVSYGVRTSARTITSAALIMIAVFIGFA

FVGMPLVAQLGVACAVAIAVDATVVRLVLVPALMAMFDKRNWWLPGWLDRVLPTVDFETP

CVDELPGPHSESALRLQGSGFEEPRIDYHAIAVSAARLKRLADGGTVTAPDTQAVLADRW

RDNLTVALEALQTGKNGTDTDTVEMPGPLSRVTPVEITQVKLPTGQKCVVPTDAEALRMQ

GLLLMRRNAERDYTEFARLASAMEPRMAAKVLAGIDQHYCVQSNQRWVSSQLVRRLADPK

PGDGAMECPTVRRQCLSVAVAMLEDAR

>mmv:MYCMA_2505 membrane protein; K20466 heme transporter (A)

MMRTSDYLRRYRWLTVAVWLILIVAAVGLVAGRGEKLTGGGFEVAGSQSLKVHDAMETGF

REQGASPLALVAAPRPDATVSDMAAAVDYLKQVAAQIPQVSVEPIPPQLTPQPDRPFVVT

LRIGFDNTGATDIAKSLRKKIGVQGGQPGQIAGGRVSLYVIGQGALGAAMTEQIAGDIKK

AEKWNIPVILIVLIAAFGSLAAASLPLLLGVCTVALSVAIVLALSNITTISVFALPTVTM

IGLAVAVDYSLFILMRYREELNSGKNRQEAIAGAMATSGLTVTFSGLTVIAALAGIYLIG

TPALASMASGAIIVVAIAVLTSTTLMPALLAIFGKTAANQSRFLRVSPLRKHSIPFWRKW

TQQVMRRPWLSALGASIFLLALAAPSLQMHVSNSMLRQFDSSHEIRRGIDAAAVAMGPGA

LGPVQVLLTFKDSPAHAHAQALETTKETIAQAPNIARVSEVSFSTDGHDALISAVLSVDP

EANAARDTVSWLRANVPDALENSGADINVGGPTALLFDYDTRVEHSLLGVFAFVCVLAFV

MLLATLRSPVLALKGVVMTVLSVAAAYGSLVIVFQWGWLEFLGFHKANIDSSIPPLALAL

TFGLTMDYEIFLLARVRERYLRTGDCGDAVSYGVRTSARTITSAALIMIAVFIGFAFVGM

PLVAQLGVACAVAIAVDATVVRLVLVPALMAMFDKRNWWLPGWLDRVLPTVDFETPCVDE

LPGPHSESALRLQGSGFEEPRIDYHAIAVSAARLKRLADGGTVTAPDTQAVLADRWRDNL

TVALEALQTGKNGTDTDTVEMPGPLSRVTPVEITQVKLPTGQKCVVPTDAEALRMQGLLL

MRRNAERDYTEFARLASAMEPRMAAKVLAGIDQHYCVQSNQRWVSSQLVRRLADPKPGDG

AMECPTVRRQCLSVAVAMLEDAR

>may:LA62_22990 membrane protein; K20466 heme transporter (A)

MMRTSDYLRRYRWLTVAVWLILIVAAVGLVAGRGEKLTGGGFEVAGSQSLKVHDAMETGF

REQGASPLALVAAPRPDATVSDMAAAVDYLKQVAAQIPQVSVEPIPPQLTPQPDRPFVVT

LRIGFDNTGATDIAKSLRKKIGVHGGQPGQIAGGRVSLYVIGQGALGAAMTEQIAGDIKK

AEKWNIPVILIVLIAAFGSLAAASLPLLLGVCTVALSVAIVLALSNITTISVFALPTVTM

IGLAVAVDYSLFILMRYREELNSGKNRQEAIAGAMATSGLTVTFSGLTVIAALAGIYLIG

TPALASMASGAIIVVAIAVLTSTTLMPALLAIFGKAAANQSRFLRVSPLRKHSIPFWRKW

TQQVMRRPWLSALGASIFLLALAAPSLQMHVSNSMLRQFDSSHEIRRGIDAAAVAMGPGA

LGPVQVLLTFKDSPAHAHAQALETAKETIAQAPNIARVSDVSFSTDGHDALISAVLSVDP

EANAARDTVSWLRANVPDALENSGADINVGGPTALLFDYDTRVEHSLLGVFAFVCVLAFV

MLLATLRSPVLALKGVVMTVLSVAAAYGSLVIVFQWGWLEFLGFHKANIDSSIPPLALAL

TFGLTMDYEIFLLARVRERYLRTGDCGDAVSYGVRTSARTITSAALIMIAVFIGFAFVGM

PLVAQLGVACAVAIAVDATVVRLVLVPALMAMFDKRNWWLPGWLDHVLPTVDFETPCVDE

LPGPHSESALRLQGSGFEEPRIDYHAIAVSAARLKRLADGGTITAPDTQAVLADRWRDNL

TVALEALQTSKNGTDTDIVEMPGPLSRVTPVEITQVKLPTGQKCVVPTDAEALRMQGLLL

MRRNAERDYTEFARLASAMEPRMAAKVLAGIDQHYCVQSNQRWVSSQLVRRLADPKPGDG

AMECPTVRRQCLSVAVAMLEDAR

>mabo:NF82_22675 membrane protein; K20466 heme transporter (A)

MMRTSDYLRRYRWLTVAVWLILIVAAVGLVAGRGEKLTGGGFEVAGSQSLKVHDAMETGF

REQGASPLALVAAPRPDATVSDMAAAVDYLKQVAAQIPQVSVEPIPPQLTPQPDRPFVVT

LRIGFDNTGATDIAKSLRKKIGVHGGQPGQIAGGRVSLYVIGQGALGAAMTEQIAGDIKK

AEKWNIPVILIVLIAAFGSLAAASLPLLLGVCTVALSVAIVLALSNITTISVFALPTVTM

IGLAVAVDYSLFILMRYREELNSGKNRQEAIAGAMATSGLTVTFSGLTVIAALAGIYLIG

TPALASMASGAIIVVAIAVLTSTTLMPALLAIFGKAAANQSRFLRVSPLRKHSIPFWRKW

TQQVMRRPWLSALGASIFLLALAAPSLQMHVSNSMLRQFDSSHEIRRGIDAAAVAMGPGA

LGPVQVLLTFKDSPAHAHAQALETAKETIAQAPNIARVSDVSFSTDGHDALISAVLSVDP

EANAARDTVSWLRANVPDALENSGADINVGGPTALLFDYDTRVEHSLLGVFAFVCVLAFV

MLLATLRSPVLALKGVVMTVLSVAAAYGSLVIVFQWGWLEFLGFHKANIDSSIPPLALAL

TFGLTMDYEIFLLARVRERYLRTGDCGDAVSYGVRTSARTITSAALIMIAVFIGFAFVGM

PLVAQLGVACAVAIAVDATVVRLVLVPALMAMFDKRNWWLPGWLDHVLPTVDFETPCVDE

LPGPHSESALRLQGSGFEEPRIDYHAIAVSAARLKRLADGGTITAPDTQAVLADRWRDNL

TVALEALQTSKNGTDTDIVEMPGPLSRVTPVEITQVKLPTGQKCVVPTDAEALRMQGLLL

MRRNAERDYTEFARLASAMEPRMAAKVLAGIDQHYCVQSNQRWVSSQLVRRLADPKPGDG

AMECPTVRRQCLSVAVAMLEDAR

>mabl:MMASJCM_4584 transmembrane transport protein; K20466 heme transporter (A)

MQSVMMRTSDYLRRYRWLTVAVWLILIVAAVGLVAGRGEKLTGGGFEVAGSQSLKVHDAM

ETGFREQGASPLALVAAPRPDATVSDMAAAVDYLKQVAAQIPQVSVEPIPPQLTPQPDRP

FVVTLRIGFDNTGATDIAKSLRKKIGVQGGQPGQIAGGRVSLYVIGQGALGAAMTEQIAG

DIKKAEKWNIPVILIVLIAAFGSLAAASLPLLLGVCTVALSVAIVLALSNITTISVFALP

TVTMIGLAVAVDYSLFILMRYREELNSGKNRQEAIAGAMATSGLTVTFSGLTVIAALAGI

YLIGTPALASMASGAIIVVAIAVLTSTTLMPALLAIFGKAAANQSRFLRVSPLRKHSIPF

WRKWTQQVMRRPWLSALGASIFLLALAAPSLQMHVSNSMLRQFDSSHEIRRGIDAAAVAM

GPGALGPVQVLLTFKDSPAHAHAQALETAKETIAQAPNIARVSDVSFSTDGHDALISAVL

SVDPEANAARDTVSWLRANVPDALENSGADINVGGPTALLFDYDTRVEHSLLGVFAFVCV

LAFVMLLATLRSPVLALKGVVMTVLSVAAAYGSLVIVFQWGWLEFLGFHKANIDSSIPPL

ALALTFGLTMDYEIFLLARVRERYLRTGDCGDAVSYGVRTSARTITSAALIMIAVFIGFA

FVGMPLVAQLGVACAVAIAVDATVVRLVLVPALMAMFDKRNWWLPGWLDRVLPTVDFETP

CVDELPGPHSESALRLQGSGFEEPRIDYHAIAVSAARLKRLADGGTVTAPDTQAVLADRW

RDNLTVALEALQTGKNGTDTDTVEMPGPLSRVTPVEITQVKLPTGQKCVVPTDAEALRMQ

GLLLMRRNAERDYTEFARLASAMEPRMAAKVLAGIDQHYCVQSNQRWVSSQLVRRLADPK

PGDGAMECPTVRRQCLSVAVAMLEDAR

>maz:LA61_22885 membrane protein; K20466 heme transporter (A)

MMRTSDYLRRYRWLTVAVWLILIVAAVGLVAGRGEKLTGGGFEVAGSQSLKVHDAMETGF

REQGASPLALVAAPRPDATVSDMAAAVDYLKQVAAQIPQVSVEPIPPQLTPQPDRPFVVT

LRIGFDNTGATDIAKSLRKKIGVHGGQPGQIAGGRVSLYVIGQGALGAAMTEQIAGDIKK

AEKWNIPVILIVLIAAFGSLAAASLPLLLGVCTVALSVAIVLALSNITTISVFALPTVTM

IGLAVAVDYSLFILMRYREELNSGKNRQEAIAGAMATSGLTVTFSGLTVIAALAGIYLIG

TPALASMASGAIIVVAIAVLTSTTLMPALLAIFGKAAANQSRFLRVSPLRKHSIPFWRKW

TQQVMRRPWLSALGASIFLLALAAPSLQMHVSNSMLRQFDSSHEIRRGIDAAAVAMGPGA

LGPVQVLLTFKDSPAHAHAQALETAKETIAQAPNIARVSDVSFSTDGHDALISAVLSVDP

EANAARDTVSWLRANVPDALENSGADINVGGPTALLFDYDTRVEHSLLGVFAFVCVLAFV

MLLATLRSPVLALKGVVMTVLSVAAAYGSLVIVFQWGWLEFLGFHKANIDSSIPPLALAL

TFGLTMDYEIFLLARVRERYLRTGDCGDAVSYGVRTSARTITSAALIMIAVFIGFAFVGM

PLVAQLGVACAVAIAVDATVVRLVLVPALMAMFDKRNWWLPGWLDHVLPTVDFETPCVDE

LPGPHSESALRLQGSGFEEPRIDYHAIAVSAARLKRLADGGTITAPDTQAVLADRWRDNL

TVALEALQTSKNGTDTDIVEMPGPLSRVTPVEITQVKLPTGQKCVVPTDAEALRMQGLLL

MRRNAERDYTEFARLASAMEPRMAAKVLAGIDQHYCVQSNQRWVSSQLVRRLADPKPGDG

AMECPTVRRQCLSVAVAMLEDAR

>mak:LH56_01720 membrane protein; K20466 heme transporter (A)

MMRTSDYLRRYRWLTVAVWLILIVAAVGLVAGRGEKLTGGGFEVAGSQSLKVHDAMETGF

REQGASPLALVAAPRPDATVSDMAAAVDYLKQVAAQIPQVSVEPIPPQLTPQPDRPFVVT

LRIGFDNTGATDIAKSLRKKIGVQGGQPGQIAGGRVSLYVIGQGALGAAMTEQIAGDIKK

AEKWNIPVILIVLIAAFGSLAAASLPLLLGVCTVALSVAIVLALSNITTISVFALPTVTM

IGLAVAVDYSLFILMRYREELNSGKNRQEAIAGAMATSGLTVTFSGLTVIAALAGIYLIG

TPALASMASGAIIVVAIAVLTSTTLMPALLAIFGKAAANQSRFLRVSPLRKHSIPFWRKW

TQQVMRRPWLSALGASIFLLALAAPSLQMHVSNSMLRQFDSSHEIRRGIDAAAVAMGPGA

LGPVQVLLTFKDSPAHAHAQALETAKETIAQAPNIARVSDVSFSTDGHDALISAVLSVDP

EANAARDTVSWLRANVPDALENSGADINVGGPTALLFDYDTRVEHSLLGVFAFVCVLAFV

MLLATLRSPVLALKGVVMTVLSVAAAYGSLVIVFQWGWLEFLGFHKANIDSSIPPLALAL

TFGLTMDYEIFLLARVRERYLRTGDCGDAVSYGVRTSARTITSAALIMIAVFIGFAFVGM

PLVAQLGVACAVAIAVDATVVRLVLVPALMAMFDKRNWWLPGWLDRVLPTVDFETPCVDE

LPGPHSESALRLQGSGFEEPRIDYHAIAVSAARLKRLADGGTVTAPDTQAVLADRWRDNL

TVALEALQTGKNGTDTDTVEMPGPLSRVTPVEITQVKLPTGQKCVVPTDAEALRMQGLLL

MRRNAERDYTEFARLASAMEPRMAAKVLAGIDQHYCVQSNQRWVSSQLVRRLADPKPGDG

AMECPTVRRQCLSVAVAMLEDAR

>mys:NF92_01780 membrane protein; K20466 heme transporter (A)

MMRTSDYLRRYRWLTVAVWLILIVAAVGLVAGRGEKLTGGGFEVAGSQSLKVHDAMETGF

REQGASPLALVAAPRPDATVSDMAAAVDYLKQVAAQIPQVSVEPIPPQLTPQPDRPFVVT

LRIGFDNTGATDIAKSLRKKIGVQGGQPGQIAGGRVSLYVIGQGALGAAMTEQIAGDIKK

AEKWNIPVILIVLIAAFGSLAAASLPLLLGVCTVALSVAIVLALSNITTISVFALPTVTM

IGLAVAVDYSLFILMRYREELNSGKNRQEAIAGAMATSGLTVTFSGLTVIAALAGIYLIG

TPALASMASGAIIVVAIAVLTSTTLMPALLAIFGKTAANQSRFLRVSPLRKHSIPFWRKW

TQQVMRRPWLSALGASIFLLALAAPSLQMHVSNSMLRQFDSSHEIRRGIDAAAVAMGPGA

LGPVQVLLTFKDSPAHAHAQALETTKETIAQAPNIARVSEVSFSTDGHDALISAVLSVDP

EANAARDTVSWLRANVPDALENSGADINVGGPTALLFDYDTRVEHSLLGVFAFVCVLAFV

MLLATLRSPVLALKGVVMTVLSVAAAYGSLVIVFQWGWLEFLGFHKANIDSSIPPLALAL

TFGLTMDYEIFLLARVRERYLRTGDCGDAVSYGVRTSARTITSAALIMIAVFIGFAFVGM

PLVAQLGVACAVAIAVDATVVRLVLVPALMAMFDKRNWWLPGWLDRVLPTVDFETPCVDE

LPGPHSESALRLQGSGFEEPRIDYHAIAVSAARLKRLADGGTVTAPDTQAVLADRWRDNL

TVALEALQTGKNGTDTDTVEMPGPLSRVTPVEITQVKLPTGQKCVVPTDAEALRMQGLLL

MRRNAERDYTEFARLASAMEPRMAAKVLAGIDQHYCVQSNQRWVSSQLVRRLADPKPGDG

AMECPTVRRQCLSVAVAMLEDAR

>myc:NF90_01780 membrane protein; K20466 heme transporter (A)

MMRTSDYLRRYRWLTVAVWLILIVAAVGLVAGRGEKLTGGGFEVAGSQSLKVHDAMETGF

REQGASPLALVAAPRPDATVSDMAAAVDYLKQVAAQIPQVSVEPIPPQLTPQPDRPFVVT

LRIGFDNTGATDIAKSLRKKIGVQGGQPGQIAGGRVSLYVIGQGALGAAMTEQIAGDIKK

AEKWNIPVILIVLIAAFGSLAAASLPLLLGVCTVALSVAIVLALSNITTISVFALPTVTM

IGLAVAVDYSLFILMRYREELNSGKNRQEAIAGAMATSGLTVTFSGLTVIAALAGIYLIG

TPALASMASGAIIVVAIAVLTSTTLMPALLAIFGKTAANQSRFLRVSPLRKHSIPFWRKW

TQQVMRRPWLSALGASIFLLALAAPSLQMHVSNSMLRQFDSSHEIRRGIDAAAVAMGPGA

LGPVQVLLTFKDSPAHAHAQALETTKETIAQAPNIARVSEVSFSTDGHDALISAVLSVDP

EANAARDTVSWLRANVPDALENSGADINVGGPTALLFDYDTRVEHSLLGVFAFVCVLAFV

MLLATLRSPVLALKGVVMTVLSVAAAYGSLVIVFQWGWLEFLGFHKANIDSSIPPLALAL

TFGLTMDYEIFLLARVRERYLRTGDCGDAVSYGVRTSARTITSAALIMIAVFIGFAFVGM

PLVAQLGVACAVAIAVDATVVRLVLVPALMAMFDKRNWWLPGWLDRVLPTVDFETPCVDE

LPGPHSESALRLQGSGFEEPRIDYHAIAVSAARLKRLADGGTVTAPDTQAVLADRWRDNL

TVALEALQTGKNGTDTDTVEMPGPLSRVTPVEITQVKLPTGQKCVVPTDAEALRMQGLLL

MRRNAERDYTEFARLASAMEPRMAAKVLAGIDQHYCVQSNQRWVSSQLVRRLADPKPGDG

AMECPTVRRQCLSVAVAMLEDAR

>mmc:Mmcs_0162 MmpL11; K20466 heme transporter (A)

MMRLSGTLRRFRWAVFGAWVLLLVPAIYLALNHSSNLTGGGFEVEASQSLHVQYELEDHF

PDQGASPLALVAAPRADASYDDMNAAVAHLEQIATQVPSVKISPNPQQPPPQPDRPYVIT

LSLDFNNTGATDVAKDLRTRVGIDGEEPGEFRDGKVKMYVIGQGALGAAASQAIKTDVAQ

AEQWNLPIVLIVLLAVFGSFAAAAMPLLLGVCTVAVTMGLVYLLSTITTMSVFVASTVSM

FGIALAVDYSLFILMRFREELRAGRDAQQAADAAMATSGLAVLLSGMTVIASVTGIYLIN

TPVLTSMATGAILAVAVAVLTSTTLTPAVLATFGRAAAKRSSYLHFSRRPDTTQSRFWTR

WTGWVMRRPWVAATSAALVLLTMAAPAFMMELGNSMQRQFEPTHEVRGGVNAAAEALGPG

ALGPVRVLVTFPDGNPASAAAKEPTLEAVRAEMSKAPNVATVTPAEFGDDYRSALLSAVL

TVDPEDKGARETVDWMRANLPSTAGPNVSVDVGGPTALIKDFDDRVSATQPLVFGFVALI

AFLMLLISIRSVVLALKGVLMTVLSVAAAYGSLVAVFQWGWLEPLGFEPIPSLDSTIPPL

VLAMTFGLSMDYEIFLLTRIRERFLQTGNTRDAVAYGVSTSARTITSAALIMIAVFIGFA

FAGMPLVAQLGVACAVAIAVDATIVRLVLVPALMAMFDRWNWWLPKWLDRVLPSVDFEKP

LPKVQIDDLVIIPDDISALGPSGADLRGMVKSAARMKTLAPQTVTVADPLAFSGCQPIKQ

RMVARNGGDQTVKLRTGAGLWRGGRNGHGDGNGSTVRTTLPVHPVTLWRGRLSVALDALE

TAQQTEAETDRAPVERRTPVETTNVQLPTGDRLQIPTGAETLRLKSYLIMCRNSSRDYAE

FAELVESMETQTAAIVLSGMDRYYCGQQPKKQWVATQLVRRLADPQPSDEHDAAISDPDA

ESDWALVRQRCLSVAVAMLEEAR

>mkm:Mkms_0171 MmpL11; K20466 heme transporter (A)

MMRLSGTLRRFRWAVFGAWVLLLVPAIYLALNHSSNLTGGGFEVEASQSLHVQYELEDHF

PDQGASPLALVAAPRADASYDDMNAAVAHLEQIATQVPSVKISPNPQQPPPQPDRPYVIT

LSLDFNNTGATDVAKDLRTRVGIDGEEPGEFRDGKVKMYVIGQGALGAAASQAIKTDVAQ

AEQWNLPIVLIVLLAVFGSFAAAAMPLLLGVCTVAVTMGLVYLLSTITTMSVFVASTVSM

FGIALAVDYSLFILMRFREELRAGRDAQQAADAAMATSGLAVLLSGMTVIASVTGIYLIN

TPVLTSMATGAILAVAVAVLTSTTLTPAVLATFGRAAAKRSSYLHFSRRPDTTQSRFWTR

WTGWVMRRPWVAATSAALVLLTMAAPAFMMELGNSMQRQFEPTHEVRGGVNAAAEALGPG

ALGPVRVLVTFPDGNPASAAAKEPTLEAVRAEMSKAPNVATVTPAEFGDDYRSALLSAVL

TVDPEDKGARETVDWMRANLPSTAGPNVSVDVGGPTALIKDFDDRVSATQPLVFGFVALI

AFLMLLISIRSVVLALKGVLMTVLSVAAAYGSLVAVFQWGWLEPLGFEPIPSLDSTIPPL

VLAMTFGLSMDYEIFLLTRIRERFLQTGNTRDAVAYGVSTSARTITSAALIMIAVFIGFA

FAGMPLVAQLGVACAVAIAVDATIVRLVLVPALMAMFDRWNWWLPKWLDRVLPSVDFEKP

LPKVQIDDLVIIPDDISALGPSGADLRGMVKSAARMKTLAPQTVTVADPLAFSGCQPIKQ

RMVARNGGDQTVKLRTGAGLWRGGRNGHGDGNGSTVRTTLPVHPVTLWRGRLSVALDALE

TAQQTEAETDRAPVERRTPVETTNVQLPTGDRLQIPTGAETLRLKSYLIMCRNSSRDYAE

FAELVESMETQTAAIVLSGMDRYYCGQQPKKQWVATQLVRRLADPQPSDEHDAAISDPDA

ESDWALVRQRCLSVAVAMLEEAR

>mjl:Mjls_0152 MmpL11; K20466 heme transporter (A)

MMRLSGTLRRFRWAVFGAWVLLLVPAIYLALNHSSNLTGGGFEVEGSQSLHVQYELEDHF

PDQGASPLALVAAPRADASYDDMNAAVAHLEQIATQVPSVKISPNPQQPPPQPDRPYVIT

LSLDFNNTGATDVAKDLRTRVGIDGEEPGEFRDGKVKMYVIGQGALGAAASQAIKTDVAQ

AEQWNLPIVLIVLLAVFGSFAAAAMPLLLGVCTVAVTMGLVYLLSTITTMSVFVASTVSM

FGIALAVDYSLFILMRFREELRAGRDAQQAADAAMATSGLAVLLSGMTVIASVTGIYLIN

TPVLTSMATGAILAVAVAVLTSTTLTPAVLATFGRAAAKRSSYLHFSRRPDTTQSRFWTR

WTGWVMRRPWVAATSAALVLLTMAAPAFMMELGNSMQRQFEPTHEVRGGVNAAAEALGPG

ALGPVRVLVTFPDGNAASAAAKEPTLEAVRAEMSKAPNVATVTPAEFGDDYRSALLSAVL

TVDPEDKGARETVDWMRANLPSTAGPNVSVDVGGPTALIKDFDDRVSATQPLVFGFVALI

AFLMLLISIRSVVLALKGVLMTVLSVAAAYGSLVAVFQWGWLEPLGFEPIPSLDSTIPPL

VLAMTFGLSMDYEIFLLTRIRERFLQTGNTRDAVAYGVSTSARTITSAALIMIAVFIGFA

FAGMPLVAQLGVACAVAIAVDATIVRLVLVPALMAMFDRWNWWLPKWLDRVLPSVDFEKP

LPKVQIDDLVIIPDDISALGPSGADLRGMVKSAARMKTLAPQTVTVADPLAFSGCQPIKQ

RMVARNGGDQTVKLRTGAGLWRGGRDGNGSTVRTTLPVHPVTLWRGRLSVALDALETAQQ

TEAETDRAPVERRTPVETTNVQLPTGDRLQIPTGAETLRLKSYLIMCRNSSRDYAEFAEL

VESMETQTAAIVLSGMDRYYCGQQPKKQWVATQLVRRLADPQPSDEHDAAISDPDAESDW

ALVRQRCLSVAVAMLEEAR

>mjd:JDM601_0165 mmpL11; transmembrane transport protein MmpL11; K20466 heme transporter (A)

MLPSLYLAANHSGHLTGGGFEVEGSQSLYVQYQLEDHFPDQGASPLALVAAPRADATYHD

MQDAVALLQRVAGEVPSVTVVPDQMQPPPRPDRPYVVSLRVDFDNTGAVDIAKKLRARLG

VEGEHPGRIENGSVNLYVIGQGALGAAASAKTKQDIAAAEQWNMPIVLIVLLAVFGSLAA

AAIPLALGIGTVIVTMGLVYLLSMFTTMSVFVTSTVSMFGIALAIDYSLFILMRYREELR

AGRQPQEAVDAAMATSGLAVVLSGLTVVASLTGIYLINTPVLVSMATGAILAVSVAVLAA

VSLTPVVLAVFGRPVAKRSALLNWSRSPAAQSRFWTRWTAWVMRRPWVSALLAAGLLLTL

AAPALSLEQGNSMLRQFDSSHEIRGGVAAAAQALGPGALGPVRVLVTIPGADASAPSHAE

TFAAVRQEMSRAPNIASVSPPVFGDDDSSGLLSAVLSVDPEDMAARETIDWMRDQLPAAA

GSSSVRVDVGGPTALIKDFDDRVAATEPLVLVFVALIAFVMLLISIQSVFLAFKGVVMTV

LSVGAAYGSLVIVFQWGWLERFGLTPTGSVDSFIPPIVLAMTFGLSMDYEIFLLTRIRER

FLQSGNTHDAVAHGVSTSARTITSAALIMIAVFIGFAFAGMPLVAELGVACAVAIAVDAT

VVRLVLVPALMAIFAEWNWWLPRWLAWLLPSVDFEKPLPTVDLGDVVMIPDDISALITPS

ADLRAVVKSAARLKELVPDAVCVGDPLALRGCGMAEVATSKIEALPVAAATAPAGGKAVG

NALAKLAGGWPSRGGGGRALPRAIRPVHPVTVWRRRLAVALDALETESWVANEIGFEGPA

LTRCRPMETAAVQLPTGDRLQVPTCAETLRLKGYLLMSRNSPRDYRELAELVDVLDPQTV

AAALGGVDRYYSGQPADRQWMATQLVCRLADPEPVEREVDNSGDGAPPAGTEWEHVRARC

LAVAVSMLEEAR

>mmi:MMAR_0442 mmpL11; conserved transmembrane transport protein MmpL11; K20466 heme transporter (A)

MMRLSRNLRRFRWLVFTGWMLALVPAIYLALTQSGNLTGGGFEVAGSQSLQVHDVLEAQY

PDQGASSLALVATPRPDASYQDITDAVAQLRRIAGEFPGVSEVPDPTQRPPQPDRPYVVS

LRLDARNAGTSDLAKKLRARVGVEGDKPGQTADGRVRLYVIGQGALSAAAAANTKHDIAA

AERWNLPIILIVLLAVFGSLTAAAIPLALGICTVVVTMGLVYLLSMHTTMSVFVTSTVSM

FGIALAVDYSLFILMRFREELRSGRQPQEAVDAAMATSGLAVVLSGMTVVASLSGIYLIN

TPALRSMATGAILAVAVAMLTSATLTPAVLATFARAAAKRSALLHWSRRPESTQSRFWTR

WVGWVMHRPWITALSASVVLLLMAAPAASMVLGNSLLRQFDSSHEIRAGVSAAAQALGPG

ALGPVQVLIKFPDGGASTPAHSQTVGAVRQRMAQGPNVTTVAPPKFAEDNDSALLSAVLS

VDPEDMGARHTVDWMRSELPQVAGGAQVDVGGSTALIKDFDDRVSATEPLVLAFVALIAF

VMLLISIHSVLLALKGVLMTLLSVAAAYGSLVMVFQWGWGERLGFPQLTSIDSTVPPLVL

AMTFGLSMDYEIFLLTRIRERFLHTGRTRDAVAYGVSTSARTITSAALIMIAVFCGFAFA

GMPLVAEIGVACAVAIAVDATIVRLIMVPALMAMFSQWNWWLPRWLGRILPSVDFDRPLP

EVDLADVVVIPEDIAATIPPGADLRMVLKSAAKLKQLAPDTICVADPLAFTGCERVGKGP

ERIPELVKDGLNLAGGTDTAGPGGAAEAKNGSNGQSAARKAVGLAYRSGIARAMSWAERP

VHPVTVWRGRLSIALDALETNASERRAGPGGGREPSYRRRSPVETTNVQLPTGDRLLIPT

GAETLRLKGYLIMSRNSSRDYAELADMVEAMEPETAAVVLAGMDRYYCCQPPRRQWIATQ

LVRRLADPHPSDVDVEWPQPDDKADWEEVRQRCLAVAVAMLEEAR

>mrh:MycrhN_1544 putative RND superfamily drug exporter; K20466 heme transporter (A)

MRLSSTLRRYRWAVFAVWLLLLVPSIYLALNQSSNLTGGGFEVEGSQSLRVQRELEDNFP

DQGASPLALVAAPRADASFDDMNDAVAFLERAAAEVPSVRVVPNPLGVPPTRSVGGQPAP

QPDRPYVVTLQLDFNNTGAVDVAKKLRQKVGVNGEDPGEIENGKVKLYVIGQGALGAAAT

QATKHDIAQAEQWNLPIVLIVLLAVFGSLAAAAMPLVLGVCTVVVTMGLVYLLSMYTQMS

VFVTSTVSMFGIALAIDYSLFILMRFREELRSGRDPQQAADAAMATSGLAVVLSGLTVIA

SVTGIYLIQTPVLASMATGAILAVAVAVLTSTTLTPAVLATFGEAAAKRSSYLHWSRRPE

TTQSRFWTRWTGWVMRRPWLAALGASALLLTLAAPAFSMVLGNSMQRQFEPTHEIRGGVN

AAAQALGPGALGPVRVLVSFPDGNAASAPSKGPLLDTVRQRMAQGPNVVSVAPPVFGEDY

RHAMLSAVLSVDPEDMGARETVDWMRAQLPSTPGLGDARIDVGGPTALIKDFDDQVSAKQ

PLVFAFVALIAFVMLLIAIRSVFLALKGVLMTVLSVAAAYGSLVAVFQWGWLEPLGFEPI

SSLDSTIPPLVLAMTFGLSMDYEIFLLTRIRERFLQTGNTRDAVAYGVSTSARTITSAAL

IMIAVFIGFAFAGMPLVAQLGVACAVAIAVDATVVRLVLVPALMAMFDEWNWWLPRWLDR

ILPSVDFEKPLPKVDMPDLIIIPDDLSSLGPTGSELRMVVKSAAKLKTLVPQAVTVADPL

AFSGCMPVKQLRSATRLNSTHRNGAGGKALLTADLPPHPVTMWRGRLSVALEALQAASDL

ERAPMERSSPVETTNVQLPTGDRLQIPTGAETLRLQGYLIMCRNSTRDYADLADLVEAME

TRTAALVLAGMDRYYCGPDPRKQWVATQLVRRLADPQPSDEHDTRMSGPQAEADWAKVRE

RCLSVAVAMLEEAR

>mmm:W7S_24515 mmpL11 protein; K20466 heme transporter (A)

MMRLSRSLRKYRWLVFTGWLLALVPAVYLALTQSGNLTGGGFDVAGSQSLAVHDQLEELY

HDQGGSSLALVAAPRADATYQDMNEAVAQLRRLTAEVPGTSEIPNPTQRPPQPDRPYVLS

VRLDSRNTSDVAKKLRTKVGIKGDQPGQTANGRVRLYVIGQGALSAAAAANTKHDIAAAE

RWNLPVILIVLLAVFGSLAAAAIPLALGVCTVVVTMGLVYLLSAYTTMSVFVTSTVSMFG

IALAVDYSLFILMRFREELRSGRQPREAVDAAMATSGLAVVLSGATVVASLTGIYVINTP

ALKSMATGAILAVAVAMLTSTTLTPAALATFGRAAAKRSGFLHWSRRPESTQSRFWNRWI

GWVMRRPWMSALAASLVLLVMAAPAASMVLGNSLLRQFDSSHEIRAGVGAAAQALGPGAL

GPIRVLINFPEGGAASPEHSHTVGAVRQRMTQAPNIVSVSPPQYAEDNGSALLSAVLSVD

PEDMRARETVGWMRAELPKVQQLGTARVDVGGPTALIKDFDDQVSATEPLVLGFVALIAF

VMLLVSVHSVFLALKGVLMTLLSVAAAYGSLVMVFQWGWLKDLGFAQISSIDSTVPPLVL

AMTFGLSMDYEIFLLTRIRERFLHSGNTRDAVAYGVSTSARTITSAALIMIAVFVGFAFA

GMPLVAEIGVACAVAIAVDATVVRLVMVPALMAMFAQWNWWLPPWLSRVLPSVDFDRPLP

EVDLGDVVVIPDDISALVAPSADLRMVLKSAAKLKHLAPDAICVTDPLAFSGCGRGRGQA

SSGPDEERTRGLGPGQIPHQVALGEEKVAVGVTAGDRKTATNGHANGSSGPKKLVGGLAA

RNGIAKAISGSDRPVHPVTLWRGRLSVAIDALETDPDAAPAGATDRPRYARRSPVETTHV

QLPTGDRLLVPTGAETLRLKGYLIMCRNSRRDYADFADMVDAMEPETAAVVLAGMDRYYC

CESSRRQCIATQLVRRLADPDPCDYPEDQGSEADANSDWEGIRERCLSVAVAMLEEAR

>mcb:Mycch_0176 putative RND superfamily drug exporter; K20466 heme transporter (A)

MRVSSNLRRFRWAVFAVWLLLLVPAVYLAMNQSDNLTGGGFEVDGSQSLYVQHQIEAQFP

DQGASPLALVAAPRPDASFDDMNAAVARLQSIAAEVPSVTVKPNPAQPPPQPDRPYVITL

QLDFENTGAVDVAKQLREKVGITGDQPGEMENGKVRLYVIGQGALGAAATLATKHDIAQA

ERWNLPIVLIVLLAVFGSLAAAAMPLLLGICTVVVTMGLVYLLSMYTTMSVFVTSTVSMF

GIAVAIDYSLFILMRFREELRAGREPQDAADAAMATSGLAVLLSGLTVIASVTGIYLINT

PVLQSMATGAILAVAVAVLTSTTLTPAVLATFGRRAARRSSYLHWGRGVEATQSKFWTRW

TGWVMRRPWASALAAAVLLLTLASPAFSMVLGNSMQRQFDPTHEIRGGVNAAAEALGPGA

LGPIRVLVTFPDGSAASAPAKEPLLGAIRAKMAQAPSTVSVSPPVFGNDYRSALLSAVLS

VDPEDMSARETVDWMRAQLPPVAGDTARIDVGGPTALIKDFDDRVSKTQPLVFGFVALIA

FVMLLISIRSVFLAFKGVLMTVLSVAAAYGSLVMVFQWGWLSELGFKQISSLDSTIPPLV

LALTFGLSMDYEIFLLTRIRERFLQTGNTRDAVAYGVSTSARTITSAALIMIAVFIGFAF

AGMPLVAQLGVACAVAIAVDATVVRLVLVPALMAMFDEWNWWLPHWLDRLLPSVDFEKPL

PKADIGDLIIIPDDISALAPSGSDLRTVVKSAAKLKTLAPQIITVADPLAFSGCQPCGKL

VPGRLKSEGRVPAAIGNRAHGKTVAVKLPKHPVTMWRGRLDVALDALAVERSVATAAEQA

GHTSLERVSPMETTNVLLPTGDRLQIPTGAETLRLKSYLVMARNDSRDYKDFAELADAMD

AQTAAEVLAGMDRYYCGQPARAHWVATQLVRRLADPQPVDGPDIAASGAAAETEWAKVRQ

RCLSVAVAMLEEAR

>mli:MULP_00434 mmpL11; conserved transmembrane transport protein MmpL11; K20466 heme transporter (A)

MRLSRNLRRFRWLVFTGWMLALVPAIYLALTQSGNLTGGGFEVAGSQSLRVHDVLEAQYP

DQGASSLALVATPRPDASYQDITDAVAQLRRIAGEFPGVSEVPDPTQRPPQPDRPYVVSL

RLDARNAGTSDLAKKLRARVGVEGDKPGQTADGRVRLYVIGQGALSAAAAANTKHDIAAA

ERWNLPIILIVLLAVFGSLTAAAIPLALGICTVVVTMGLVYLLSMHTTMSVFVTSTVSMF

GIALAVDYSLFILMRFREELRSGRQPQEAVDAAMATSGLAVVLSGMTVVASLSGIYLINT

PALRSMATGAILAVAVAMLTSATLTPAVLATFARAAAKRSALLHWSRRPESTQSRFWTRW

VGWVMHRPWITALSASVVLLLMAAPAASMVLGNSLLRQFDSSHEIRAGVSAAAQALGPGA

LGPVQVLIKFPDGGASTPAHSQTVGAVRQRMAQGPNVTTVAPPKFAEDNDSALLSAVLSV

DPEDMGARHTVDWMRSELPQVAGGAQVDVGGSTALIKDFDDRVSATEPLVLAFVALIAFV

MLLISIHSVLLALKGVLMTLLSVAAAYGSLVMVFQWGWGERLGFPQLTSIDSTVPPLVLA

MTFGLSMDYEIFLLTRIRERFLHTGRTRDAVAYGVSTSARTITSAALIMIAVFCGFAFAG

MPLVAEIGVACAVAIAVDATIVRLIMVPALMAMFSQWNWWLPRWLGRILPSVDFDRPLPE

VDLADVVVIPEDIAATIPPGADLRMVLKSAAKLKQLAPDTICVADPLAFTGCERVGKGPE

RIPELVKDGLNLAGGTDTAGPGGAAEAKNGSNGQSAARKAVGLAYRSGIARAMSWAERPV

HPVTVWRGRLSIALDALETNASERRAGPGSGREPSYRRRSPVETTNVQLPTGDRLLIPTG

AETLRLKGYLIMSRNSSRDYAELADMVEAMEPETAAVVLAGMDRYYCCQPPRRQWIATQL

VRRLADPHPSDVDVEWPQPDDKADWEEVRQRCLAVAVAMLEEAR

>mkn:MKAN_16475 membrane protein; K20466 heme transporter (A)

MREKYRENNIQQFMMRLSRNLRRFRWLVFTGWFLALVPAIYLAMTQSGNLTGGGFEVAGS

QSLLVHDVLDAQYPDQEAPSLALVATPRPDASFQDINDAVARLRSIAGEFPGVSEVPNPT

QRPPQPDRPYVVSLRLDARNAGTSDIAKKLRQKIGVKGDKPGQTADGRVRLYVIGQGALS

AAAAANTKHDIADAERWNLPIILIVLLAVFGSLAAAAIPLALGICTVVVTMGLVYLLSMH

TTMSVFVTSTVSMFGIALAVDYSLFILMRFREELRTGRRPQEAVDAAMATSGLAVVLSGM

TVIASLTGIYLINTPALRSMATGAILAVAVAMLTSATLTPALLATFARAAAKRSALLHWS

RRPESTQSRFWARWVGWVMRRPWITALSASLVLLLMAAPATQMVLGNSLLRQFDSSHEIR

AGVSAAAQALGPGALGPAQVLVTFPDGGASTPAHSQTIAAIRQQMTQAPDVASVAPPKFA

ADNRSALLSAVLSVDPEDMGARHTVDWMRTHLPGAADGARIDVGGPTALIKDFDDRVAAT

QPLVLAFVALIAFVMLLVSVHSMFLALKGVLMTLLSVAAAYGSLVMVFQWGWAEKLGFPH

LSSIDSTVPPLVLAMTFGLSMDYEIFLLTRIRERFLHTGQTRDAVAYGVSTSARTITSAA

LIMIAVFCGFAFAGMPLVAEIGVACAVAIAVDATIVRLILVPALMAMFSQWNWWLPRWLA

RMLPSVDFDRPLPEVDLADVVVIPDDIAAAIAPSGDLRMVLKSAARFKHLAPESICVADP

LAFTGCGRSARGPEHVPELVRDGADVAVPATAPAASAPASAASAKTGGQSAASKVGQAYR

NSLARAMSWAERPVHPVTVWRGRLAIALDALETDAAERAAAKSGTDRPRFERRSPVETTN

VQLPTGDRLLIPTGAETLRLKGYLIMSRNSSRDYAEFADMVEAMEPETAAVVLAGMDRYY

CCQPLGSYSRRQWMATQLVRRLADPHPSDVDDEWPDPDARANWEEVRQRCLAVAVAMLEE

AR

>mks:LG40_16295 membrane protein; K20466 heme transporter (A)

MMRLSRNLRRFRWLVFTGWFLALVPAIYLAMTQSGNLTGGGFEVAGSQSLLVHDVLDAQY

PDQEAPSLALVATPRPDASFQDINDAVARLRSIAGEFPGVSEVPNPTQRPPQPDRPYVVS

LRLDARNAGTSDIAKKLRQKIGVKGDKPGQTADGRVRLYVIGQGALSAAAAANTKHDIAD

AERWNLPIILIVLLAVFGSLAAAAIPLALGICTVVVTMGLVYLLSMHTTMSVFVTSTVSM

FGIALAVDYSLFILMRFREELRTGRRPQEAVDAAMATSGLAVVLSGMTVIASLTGIYLIN

TPALRSMATGAILAVAVAMLTSATLTPALLATFARAAAKRSALLHWSRRPESTQSRFWAR

WVGWVMRRPWITALSASLVLLLMAAPATQMVLGNSLLRQFDSSHEIRAGVSAAAQALGPG

ALGPAQVLVTFPDGGASTPAHSQTIAAIRQQMTQAPDVASVAPPKFAADNRSALLSAVLS

VDPEDMGARHTVDWMRTHLPGAADGARIDVGGPTALIKDFDDRVAATQPLVLAFVALIAF

VMLLVSVHSMFLALKGVLMTLLSVAAAYGSLVMVFQWGWAEKLGFPHLSSIDSTVPPLVL

AMTFGLSMDYEIFLLTRIRERFLHTGQTRDAVAYGVSTSARTITSAALIMIAVFCGFAFA

GMPLVAEIGVACAVAIAVDATIVRLILVPALMAMFSQWNWWLPRWLARMLPSVDFDRPLP

EVDLADVVVIPDDIAAAIAPSGDLRMVLKSAARFKHLAPESICVADPLAFTGCGRSARGP

EHVPELVRDGADVAVPATAPAASAPASAASAKTGGQSAASKVGQAYRNSLARAMSWAERP

VHPVTVWRGRLAIALDALETDAAERAAAKSGTDRPRFERRSPVETTNVQLPTGDRLLIPT

GAETLRLKGYLIMSRNSSRDYAEFADMVEAMEPETAAVVLAGMDRYYCCQPLGSYSRRQW

MATQLVRRLADPHPSDVDDEWPDPDARANWEEVRQRCLAVAVAMLEEAR

>mki:LH54_21920 membrane protein; K20466 heme transporter (A)

MLHRIARLAIAAPGRVVAVAVLVMIATAIFGVPAIKKLSAGGGLDPGAESSHVAALLAQK

FGQGDMGMLITVTADGGAQGPQASAVGTDLVERLKNSSDVRGVRSAWTESPAAARSLISK

DGKTGLIVVAISGGETNAQKTAKQLSEELVHDRDGVRVRAGGEATVYWQVNTQTQNDLLL

MESVALPLSFLVLVWVFGGVFAAALPVAVGGFAILGSLASLRAISLFTNVSIFALNLTVA

MGMALAIDYTLLILSRFRDELAERQTRDAALIRAMTTAGRTVVFSAMTVALSMATLVLFP

QYFLKSFGYAGVAVVAFAAAAAIVVTPAAIVLLDGRLDSLDVRPLLRRILWGVPRQPASF

QQWSWYRWTKSVMRHAVPIGIAITALLLLLGSPFHRARWGFADDRILPASASARQVGDQL

RNDFAGSGIPDISVVLPEATGVSPADLDAYAAALSRVPDVSSVTSPGGTFVHGILAGSPA

APSGIKDRSAFVTVGSTAPLYSAASAVQLDRLHAVPTPAGRLVQLGGVAQSNRDSVHAIA

TRLPMVLTFIVLITVVLVFLLTGSAVLPVKAVLMNMLSLTAAFGALVWVFQEGHLGGLGT

TATGTIGVQLPVLLFCIAFGLSMDYEVFLISRIREYWLASDHGPGANDESVALGVAHTGR

VITAAALIMVIAFAALMAAQVSFMRLFGFGLTAAVLVDATLVRMLLVPAFMQVLGRLNWW

APEPLARVHARFGLSERAGHQPGAEKPTGTVH

>mne:D174_01410 membrane protein; K20466 heme transporter (A)

MMRLSSSLRRYRWAVFVVWVLLLVPSVYLAINHSDRLTGGGFEVAGSQSLDLQHQLEEQF

PDQGASPLALVAVPRADASFEDMNEAVAQLRRIAGEVPSVTEAPNPQQPPPAPDRPYVVQ

LKVDFNNSGAVDIANQLRDKVGIEGEQAGLSDNGKVRLYVIGQGALGAAAQQATKHDIAQ

AEKWNAPVVLIILLAVFGSLAAAAMPLLLGICTVVVTMGVVFLLSHVMAMSVFATSTVSM

FGIAVAVDYSLFILMRYREELRAGRDSAQAIDGAMATSGLAVVLSGLTVIASVSGIYLIN

TPILESMATGAILAVAVAVLTSTTLIPAVLATFGRSAAKRSRLLHWSRRPDTTQSRFWTR

WIGAVMRRPWVSASFAAVMLLVMAAPAFGMTLGNSMLRQFDPSHEIRGGVNAAAEALGPG

ALGPVRVMVTFPDGQADGPAAQAALATVGRTMAEGPDVAAVTPPVIGKDNDSALLSAVLT

VDPEDFRARDAVDWMRTELPKLDIGNAQIDVGGPTALIKDFDDQVSMAQPWVFVFVAVIA

FVMLLIAIRSVFLALKGVLMTVLSVAAAYGSLVVVFQWGWLEDLGFAPLESLDSTVPPLV

LAMTFGLSMDYEIFLLTRIRERFLITNNTRDAVAYGISTSARTITSAALIMIAVFIGFAF

AGMPLVAQIGVACATAIAVDATIVRLVLVPALMAMFDEWNWWLPRWLDRILPSVDFEKPL

PKVDISDLVLVPETALAAPGADLRMVVKSAAKLKRLAPQTVVVPDPLAFTGCGPAPGERR

LSGRLPTITSTGSITRPVHPVTMWRGRINVALDALETASDSEKLPVQRRSPIEATTVQLP

TGDRLLIPTGAETLRMKSYLLMCRNTSRDFAEFARLVDSMETDTAARVLTGMDRYYCGQR

PRAHWVATQLVRRLADPRPSDDPDAPAPAETDWDQVRQRCLSVAVAMLDRTELEEAR

>myv:G155_00955 membrane protein; K20466 heme transporter (A)

MMRLSSYLRRFRWAVFATWLLLLVPSIYLAIDQSSNLTGGGFDVEGSQSLHVQRQLEEHF

PDQGASPLALIASPRADASFEDMNAAVSELEKIAGEVPSVKIVPNPQQPAPQPDRPYVIT

LQLDFNNTGAVDIAKQLRQKVGVHGEDPGESENGKVKYYVIGQGALGAAATQATKHDIAA

AEKWNLPIVLIVLLAVFGSLAAAALPLVLGICTVVVTMGLVYLLSMYTQMSVFVTSTVSM

FGIALAVDYSLFILMRYREELRAGRDPADAVDAAMATSGLAVALSGLTVIASVTGIYIIN

TPVLVSMATGAILAVSVAVLTSTTLTPAVLATFGQAAAKRSSYLHWSRRAEATQSRFWTR

WTGAVMRRPWASAVAATILLLILAAPAFGMVLGNSMQRQFEPTHEIRGGVNAAADALGPG

ALGPVRVLVTFPEGTADDAKGTAALDSVRQEMTRAPNIQAVQPPVFADNNTSALLSAVLS

VDPEDLAARESVDWMRDKLPAAAGSNARVDVGGPTALIKDFDDRVSSTQPLVFLFVALIA

FVMLLVSIRSVLLALKGVLMTVLSVAAAYGSLVVVFQWGWFEELGFEKISSLDSTVPPLV

LAMTFGLSMDYEIFLLTRIRERFLQTNSTRDAVAYGVSTSARTITSAALIMIAVFIGFAF

AGMPLVAQLGVACAVAIAVDATVVRLILVPALMAMFDEWNWWLPHWLDRLLPEVDFEKPL

PKVEVTDLVIIPDDISALGPSGSDLRMMVRTAARMKHLAPQTIIVTDPLAFSGCSKPSAR

LAARRPGGPKRCPGVHPVTMWRGRLSVAVDALEAEAEAERAPMERLGPVETTNVQLPTGD

RLQIPTGAETLRLKSYLVMCRNSTKDFEEFADLVESMETETAALVLSGMDRYYCGQNPKS

RWVATQLVRRLADPQPSDEHDFVMSDPDAAAEWEKVRQRCLSVAVAMLEEAK

>mye:AB431_01675 membrane protein; K20466 heme transporter (A)

MMRLSSNLRRFRWLVFTCWLLALVPAVYLALAESNHLTGGGFDVAGSQSLHVQYQLEDHY

PQQGASPLALVAAPRADASYADMNTAVAQLEEAAKQVPSVVVVPNPSQPAPAPDRPYVVS

LRLDFNNTGAVDVARQLRQKIGVTGDQPGQISNGRVKLYVIGQGALGAAAQTSTKHDIAE

AEQWNLPIVLIVLLAVFGSLAAAAVPLLLAVCTVVVTMGVVYLLSTVMSMSVFVTSTVSM

FGIALAVDYSLFILMRFREELRAGRDPQQAADAAMATSGLAVLLSGLTVIASLTGIYLIN

TPVLRSMATGAILAVAIAVLTSTTLTPAVLATFGRPVARRSPLLQWSRRGEATQSRFWTR

WVGAVMRRPWLSALGATVVLLLMAAPAFSMVLGNSMQRQFPATHEVRGGVAAAAQALGPG

ALGPVRVLVTFPGGAASDPKNAAALEAINTEISKAPDVVSVSTPVFADNNGSALISAILS

VDPEDLAARNSIDWMRSHLRALPATNGAQIDVGGPTALIKDFDDRVGQTQPLVFVFVAAI

AFVMLLISVRSVFLAFKGVLMTVLSVAAAYGSLVMVFQWGWLEGLGFHRITSIDSTIPPL

VLAMTFGLSMDYEIFLLTRIRERYLQAGDTRDAVAYGVSTSARTITSAALIMIAVFIGFA

FAGMPLVAELGVACAVAIAVDATIVRLVLVPALMAMFDQWNWWLPSWLARILPSVDFEKP

LPKVDLGDLVVIPDDISALVPPSADLKMVVKGAAKLKTLAPDAVSVADPLAFTGCNALAG

KVKGSDDARARPVRIGPGGTSTVKLIRGYRSARTATARPVHPVTMWRGRLAIALDALETG

ADTRHPAVERLSPVETTHVQLPTGDRLQIPTCAETLRMQAYLVLCRNSRSDFAEFADLVG

GMDTETAAVVLAGMDRYYCSGQAERQWVATQLVRRLADPDPSDVDDADRWAGPEGAAEWE

RVRQRCLAVAVAILEEAR

>mgo:AFA91_07990 membrane protein; K20466 heme transporter (A)

MMRLSSTLRRFRWAVFATWLLLLVPSVYLALNQSSNLTGGGFEVEGSQSLHVQRQLEEHF

PDQGASPLALVAAPRADASYQDMNSAVAHLERIAAEVPSVTIVPNPQQPAPQPDRPYVIT

LQLGFDNTGAVDVAKQLRQKVGIQGEEPGQSENGKVKFYVIGQGALGAAATQATKHDIAA

AEKWNMPIVLIVLLAVFGSLAAAALPLVLGVCTVVVTMGLVYLLSMFTTMSVFVTSTVSM

FGIALAIDYSLFILMRFREELRAGRDHQDAIDSAMATSGLAVALSGLTVIASVTGIYLIN

TPVLVSMATGAILAVAIAVLTSTTLTPAVLATFGKAAAKRSSYLHWSRRAEATQSRFWTR

WTGAVMRRPWASATAAAILLLVLAAPAFDMVLGNSMQRQFEPTHEIRGGVNAAADALGPG

ALGPVRVLVTFPGQADASGPPETAAVEAVRQQMTKAPNVISVQPPVLSDNKDSALLSAVL

SVDPEDMAARQAIDWMRAELPAAAGDNAAIDVGGPTALIKDFDDRVSATQPLVFVFVALI

AFVMLLVSIRSVVLAFKGVLMTVLSVAAAYGSLVAVFQWGWLEQLGFAKIDSLDSTIPPL

VLAMTFGLSMDYEIFLLTRIRERFLQTNNTRDAVAYGVSTSARTITSAALIMIAVFIGFA

FAGMPLVAQLGVACAVAIAVDATVVRLVLVPALMAMFDQWNWWLPRWLDRVLPEVDFEKP

LPKIEVTDLVIIPDNIAALGPTGSDLRMMVRTAARMKTLAPQTISVADPLAFSGCTRPTT

RLSAQRKPMHAAPGLHPVTMWRGRLSVAVDALQTEAETEQAPVERRGPVETTNVQLPTGD

RLQIPTGAETLRLKGYLIMCRNTTRDFEEFAQLVDLMDSHTAALVLAGMDRYYCGQDPRN

RWVATQLVRRLADPQPSDEHDVRMSGPDAAEDWEKVRQRCLSVAVAMLEEAK

>mft:XA26_01910 Transmembrane transport protein MmpL13; K20466 heme transporter (A)

MMRLSSYLRRFRWAVFATWLLLLVPSIYLAIDQSSNLTGGGFDVEGSQSLHVQRQLEEHF

PDQGASPLALIASPRADASFEDMNAAVSELEKIAGEVPSVKIVPNPQQPAPQPDRPYVIT

LQLDFNNTGAVDIAKQLRQKVGVHGEDPGESENGKVKYYVIGQGALGAAATQATKHDIAA

AEKWNLPIVLIVLLAVFGSLAAAALPLVLGICTVVVTMGLVYLLSMYTQMSVFVTSTVSM

FGIALAVDYSLFILMRYREELRAGRDPADAVDAAMATSGLAVALSGLTVIASVTGIYIIN

TPVLVSMATGAILAVSVAVLTSTTLTPAVLATFGQAAAKRSSYLHWSRRAEATQSRFWTR

WTGAVMRRPWASAVAATILLLILAAPAFGMVLGNSMQRQFEPTHEIRGGVNAAADALGPG

ALGPVRVLVTFPEGTADDAKGTAALDSVRQEMTRAPNVQAVQPPVFADNNTSALLSAVLS

VDPEDLAARESVDWMRDKLPAAAGSNARVDVGGPTALIKDFDDRVSSTQPLVFLFVALIA

FVMLLVSIRSVLLALKGVLMTVLSVAAAYGSLVVVFQWGWFEELGFEKISSLDSTVPPLV

LAMTFGLSMDYEIFLLTRIRERFLQTNSTRDAVAYGVSTSARTITSAALIMIAVFIGFAF

AGMPLVAQLGVACAVAIAVDATVVRLILVPALMAMFDEWNWWLPHWLDRLLPEVDFEKPL

PKVEVTDLVIIPDDISALGPSGSDLRMMVRTAARMKHLAPQTIIVTDPLAFSGCSKPSAR

LAARRPGGPKRCPGVHPVTMWRGRLSVAVDALEAEAEAERAPMERLGPVETTNVQLPTGD

RLQIPTGAETLRLKSYLVMCRNSTKDFEEFADLVESMETETAALVLSGMDRYYCGQNPKS

RWVATQLVRRLADPQPSDEHDFVMSDPDAAAEWEKVRQRCLSVAVAMLEEAK

>mhad:B586_01340 hypothetical protein; K20466 heme transporter (A)

MMRLSRCLRRFRWLVFTGWLLTLVPAIYLAMTQSGNLTGGGFEVAGSQSLLVHDQLQEEY

PDQGAASLALVAAPRPDASYQDMNDAVALLKRIAGEFPGVSEVPSPTQLPPRPDRPYVVS

LRLDARNSGTSDVAKQLRTKVGIRGDQSGRTANAHVRLYVIGQGALSAAAAANSKHDIAE

AERWNLPIILIVLLAVFGSLAAAAVPLALGACTVVVTMGLVYLISMHTTMSVFVTSTVSM

FGIALAVDYSLFILMRFREELRSGRQPQEAVDAAMATSGLAVVLSGMTVIASLTGIYLIN

TPALRSMATGAILAVAIAMLTSATLTPAVLATFGRAAAKRSALLHWSRRPECTQSRFWNR

WVGWVMHRPWISALAASTLLIIMAAPATSMVLGNSLLRQFDSSHEIRAGVSAAAQALGPG

ALGPVQVLITFPGDPNVQASSAEHSQTIAAIRDRMAQAPNVLSVAPPQFADNNGSALLSA

VLSVDPEDLGARETVDWMRTELPKVPGAAHVAVGGPTALIKDFDDRVSKTEPLVLVFVAL

IAFVMLLISIRSVFLAFKGVLMTLLSVATAYGSLVMVFQWGWLENLGFTHINSIDSTVPP

LVLAMTFGLSMDYEIFLLTRIRERFLQTGRTRDAVAYGVSTSARTITSAALIMIAVFVGF

AFAGMPLVAEIGVACAVAIAVDATVVRLVLVPALMAMFAQWNWWLPCWLSRALPSVDFDR

PLPAVDLGDIVVIPDDISAAMVPSGDLRIVLKSAAKLKHLAPDAICVADPLAFTGCGHNN

KRLDRVLPGAAHEPEEGRASDDTAALPAAPACTTTSTNGHSAARKFIIGLAYRNSIARAM

PWSDRPVHPVTLWRGRLSVAIDALQTQVVSQAPDRPTYQRRRPVETTHVQLPTGDRLLIP

TGAETLRLVGYLIMSRNSSRDYAELADMVDALEPETAAVVLAELDRYYSCQPPIRQWMAT

QLVRRLSDPHPVDLTDDQWSDPDATAEWQEVRQRCLSVAVAMLEEAR

Rv0244c Homologs

>mtu:Rv0244c fadE5; acyl-CoA dehydrogenase FadE5 (A)

MSHYRSNVRDQVFNLFEVLGVDKALGHGEFSDVDVDTARDMLAEVSRLAEGPVAESFVEG

DRNPPVFDPKTHSVMLPESFKKSVNAMLEAGWDKVGIDEALGGMPMPKAVVWALHEHILG

ANPAVWMYAGGAGFAQILYHLGTEEQKKWAVLAAERGWGSTMVLTEPDAGSDVGAARTKA

VQQADGSWHIDGVKRFITSGDSGDLFENIFHLVLARPEGAGPGTKGLSLYFVPKFLFDVE

TGEPGERNGVFVTNVEHKMGLKVSATCELAFGQHGVPAKGWLVGEVHNGIAQMFEVIEQA

RMMVGTKAIATLSTGYLNALQYAKSRVQGADLTQMTDKTAPRVTITHHPDVRRSLMTQKA

YAEGLRALYLYTATFQDAAVAEVVHGVDAKLAVKVNDLMLPVVKGVGSEQAYAKLTESLQ

TLGGSGFLQDYPIEQYIRDAKIDSLYEGTTAIQAQDFFFRKIVRDKGVALAHVSGQIQEF

VDSGAGNGRLKTERALLAKALTDVQGMAAALTGYLMAAQQDVTSLYKVGLGSVRFLMSVG

DLIIGWLLQRQAAVAVAALDAGATGDERSFYEGKVAVASFFAKNFLPLLTSTREVIETLD

NDIMELDEAAF

>mtv:RVBD_0244c acyl-CoA dehydrogenase FadE5 (A)

MSHYRSNVRDQVFNLFEVLGVDKALGHGEFSDVDVDTARDMLAEVSRLAEGPVAESFVEG

DRNPPVFDPKTHSVMLPESFKKSVNAMLEAGWDKVGIDEALGGMPMPKAVVWALHEHILG

ANPAVWMYAGGAGFAQILYHLGTEEQKKWAVLAAERGWGSTMVLTEPDAGSDVGAARTKA

VQQADGSWHIDGVKRFITSGDSGDLFENIFHLVLARPEGAGPGTKGLSLYFVPKFLFDVE

TGEPGERNGVFVTNVEHKMGLKVSATCELAFGQHGVPAKGWLVGEVHNGIAQMFEVIEQA

RMMVGTKAIATLSTGYLNALQYAKSRVQGADLTQMTDKTAPRVTITHHPDVRRSLMTQKA

YAEGLRALYLYTATFQDAAVAEVVHGVDAKLAVKVNDLMLPVVKGVGSEQAYAKLTESLQ

TLGGSGFLQDYPIEQYIRDAKIDSLYEGTTAIQAQDFFFRKIVRDKGVALAHVSGQIQEF

VDSGAGNGRLKTERALLAKALTDVQGMAAALTGYLMAAQQDVTSLYKVGLGSVRFLMSVG

DLIIGWLLQRQAAVAVAALDAGATGDERSFYEGKVAVASFFAKNFLPLLTSTREVIETLD

NDIMELDEAAF

>mtc:MT0258 acyl-CoA dehydrogenase (A)

MSHYRSNVRDQVFNLFEVLGVDKALGHGEFSDVDVDTARDMLAEVSRLAEGPVAESFVEG

DRNPPVFDPKTHSVMLPESFKKSVNAMLEAGWDKVGIDEALGGMPMPKAVVWALHEHILG

ANPAVWMYAGGAGFAQILYHLGTEEQKKWAVLAAERGWGSTMVLTEPDAGSDVGAARTKA

VQQADGSWHIDGVKRFITSGDSGDLFENIFHLVLARPEGAGPGTKGLSLYFVPKFLFDVE

TGEPGERNGVFVTNVEHKMGLKVSATCELAFGQHGVPAKGWLVGEVHNGIAQMFEVIEQA

RMMVGTKAIATLSTGYLNALQYAKSRVQGADLTQMTDKTAPRVTITHHPDVRRSLMTQKA

YAEGLRALYLYTATFQDAAVAEVVHGVDAKLAVKVNDLMLPVVKGVGSEQAYAKLTESLQ

TLGGSGFLQDYPIEQYIRDAKIDSLYEGTTAIQAQDFFFRKIVRDKGVALAHVSGQIQEF

VDSGAGNGRLKTERALLAKALTDVQGMAAALTGYLMAAQQDVTSLYKVGLGSVRFLMSVG

DLIIGWLLQRQAAVAVAALDAGATGDERSFYEGKVAVASFFAKNFLPLLTSTREVIETLD

NDIMELDEAAF

>mra:MRA_0253 fadE5; acyl-CoA dehydrogenase FadE5 (A)

MSHYRSNVRDQVFNLFEVLGVDKALGHGEFSDVDVDTARDMLAEVSRLAEGPVAESFVEG

DRNPPVFDPKTHSVMLPESFKKSVNAMLEAGWDKVGIDEALGGMPMPKAVVWALHEHILG

ANPAVWMYAGGAGFAQILYHLGTEEQKKWAVLAAERGWGSTMVLTEPDAGSDVGAARTKA

VQQADGSWHIDGVKRFITSGDSGDLFENIFHLVLARPEGAGPGTKGLSLYFVPKFLFDVE

TGEPGERNGVFVTNVEHKMGLKVSATCELAFGQHGVPAKGWLVGEVHNGIAQMFEVIEQA

RMMVGTKAIATLSTGYLNALQYAKSRVQGADLTQMTDKTAPRVTITHHPDVRRSLMTQKA

YAEGLRALYLYTATFQDAAVAEVVHGVDAKLAVKVNDLMLPVVKGVGSEQAYAKLTESLQ

TLGGSGFLQDYPIEQYIRDAKIDSLYEGTTAIQAQDFFFRKIVRDKGVALAHVSGQIQEF

VDSGAGNGRLKTERALLAKALTDVQGMAAALTGYLMAAQQDVTSLYKVGLGSVRFLMSVG

DLIIGWLLQRQAAVAVAALDAGATGDERSFYEGKVAVASFFAKNFLPLLTSTREVIETLD

NDIMELDEAAF

>mtf:TBFG_10247 acyl-CoA dehydrogenase fadE5 (A)

MSHYRSNVRDQVFNLFEVLGVDKALGHGEFSDVDVDTARDMLAEVSRLAEGPVAESFVEG

DRNPPVFDPKTHSVMLPESFKKSVNAMLEAGWDKVGIDEALGGMPMPKAVVWALHEHILG

ANPAVWMYAGGAGFAQILYHLGTEEQKKWAVLAAERGWGSTMVLTEPDAGSDVGAARTKA

VQQADGSWHIDGVKRFITSGDSGDLFENIFHLVLARPEGAGPGTKGLSLYFVPKFLFDVE

TGEPGERNGVFVTNVEHKMGLKVSATCELAFGQHGVPAKGWLVGEVHNGIAQMFEVIEQA

RMMVGTKAIATLSTGYLNALQYAKSRVQGADLTQMTDKTAPRVTITHHPDVRRSLMTQKA

YAEGLRALYLYTATFQDAAVAEVVHGVDAKLAVKVNDLMLPVVKGVGSEQAYAKLTESLQ

TLGGSGFLQDYPIEQYIRDAKIDSLYEGTTAIQAQDFFFRKIVRDKGVALAHVSGQIQEF

VDSGAGNGRLKTERALLAKALTDVQGMAAALTGYLMAAQQDVTSLYKVGLGSVRFLMSVG

DLIIGWLLQRQAAVAVAALDAGATGDERSFYEGKVAVASFFAKNFLPLLTSTREVIETLD

NDIMELDEAAF

>mtb:TBMG_00246 acyl-CoA dehydrogenase fadE5 (A)

MSHYRSNVRDQVFNLFEVLGVDKALGHGEFSDVDVDTARDMLAEVSRLAEGPVAESFVEG

DRNPPVFDPKTHSVMLPESFKKSVNAMLEAGWDKVGIDEALGGMPMPKAVVWALHEHILG

ANPAVWMYAGGAGFAQILYHLGTEEQKKWAVLAAERGWGSTMVLTEPDAGSDVGAARTKA

VQQADGSWHIDGVKRFITSGDSGDLFENIFHLVLARPEGAGPGTKGLSLYFVPKFLFDVE

TGEPGERNGVFVTNVEHKMGLKVSATCELAFGQHGVPAKGWLVGEVHNGIAQMFEVIEQA

RMMVGTKAIATLSTGYLNALQYAKSRVQGADLTQMTDKTAPRVTITHHPDVRRSLMTQKA

YAEGLRALYLYTATFQDAAVAEVVHGVDAKLAVKVNDLMLPVVKGVGSEQAYAKLTESLQ

TLGGSGFLQDYPIEQYIRDAKIDSLYEGTTAIQAQDFFFRKIVRDKGVALAHVSGQIQEF

VDSGAGNGRLKTERALLAKALTDVQGMAAALTGYLMAAQQDVTSLYKVGLGSVRFLMSVG

ALIIGWLLQRQAAVAVAALDAGATGDERSFYEGKVAVASFFAKNFLPLLTSTREVIETLD

NDIMELDEAAF

>mtk:TBSG_00249 acyl-CoA dehydrogenase fadE5 (A)

MSHYRSNVRDQVFNLFEVLGVDKALGHGEFSDVDVDTARDMLAEVSRLAEGPVAESFVEG

DRNPPVFDPKTHSVMLPESFKKSVNAMLEAGWDKVGIDEALGGMPMPKAVVWALHEHILG

ANPAVWMYAGGAGFAQILYHLGTEEQKKWAVLAAERGWGSTMVLTEPDAGSDVGAARTKA

VQQADGSWHIDGVKRFITSGDSGDLFENIFHLVLARPEGAGPGTKGLSLYFVPKFLFDVE

TGEPGERNGVFVTNVEHKMGLKVSATCELAFGQHGVPAKGWLVGEVHNGIAQMFEVIEQA

RMMVGTKAIATLSTGYLNALQYAKSRVQGADLTQMTDKTAPRVTITHHPDVRRSLMTQKA

YAEGLRALYLYTATFQDAAVAEVVHGVDAKLAVKVNDLMLPVVKGVGSEQAYAKLTESLQ

TLGGSGFLQDYPIEQYIRDAKIDSLYEGTTAIQAQDFFFRKIVRDKGVALAHVSGQIQEF

VDSGAGNGRLKTERALLAKALTDVQGMAAALTGYLMAAQQDVTSLYKVGLGSVRFLMSVG

ALIIGWLLQRQAAVAVAALDAGATGDERSFYEGKVAVASFFAKNFLPLLTSTREVIETLD

NDIMELDEAAF

>mtz:TBXG_000247 acyl-CoA dehydrogenase fadE5 (A)

MSHYRSNVRDQVFNLFEVLGVDKALGHGEFSDVDVDTARDMLAEVSRLAEGPVAESFVEG

DRNPPVFDPKTHSVMLPESFKKSVNAMLEAGWDKVGIDEALGGMPMPKAVVWALHEHILG

ANPAVWMYAGGAGFAQILYHLGTEEQKKWAVLAAERGWGSTMVLTEPDAGSDVGAARTKA

VQQADGSWHIDGVKRFITSGDSGDLFENIFHLVLARPEGAGPGTKGLSLYFVPKFLFDVE

TGEPGERNGVFVTNVEHKMGLKVSATCELAFGQHGVPAKGWLVGEVHNGIAQMFEVIEQA

RMMVGTKAIATLSTGYLNALQYAKSRVQGADLTQMTDKTAPRVTITHHPDVRRSLMTQKA

YAEGLRALYLYTATFQDAAVAEVVHGVDAKLAVKVNDLMLPVVKGVGSEQAYAKLTESLQ

TLGGSGFLQDYPIEQYIRDAKIDSLYEGTTAIQAQDFFFRKIVRDKGVALAHVSGQIQEF

VDSGAGNGRLKTERALLAKALTDVQGMAAALTGYLMAAQQDVTSLYKVGLGSVRFLMSVG

ALIIGWLLQRQAAVAVAALDAGATGDERSFYEGKVAVASFFAKNFLPLLTSTREVIETLD

NDIMELDEAAF

>mtg:MRGA327_01565 acyl-CoA dehydrogenase fadE5 (A)

MSHYRSNVRDQVFNLFEVLGVDKALGHGEFSDVDVDTARDMLAEVSRLAEGPVAESFVEG

DRNPPVFDPKTHSVMLPESFKKSVNAMLEAGWDKVGIDEALGGMPMPKAVVWALHEHILG

ANPAVWMYAGGAGFAQILYHLGTEEQKKWAVLAAERGWGSTMVLTEPDAGSDVGAARTKA

VQQADGSWHIDGVKRFITSGDSGDLFENIFHLVLARPEGAGPGTKGLSLYFVPKFLFDVE

TGEPGERNGVFVTNVEHKMGLKVSATCELAFGQHGVPAKGWLVGEVHNGIAQMFEVIEQA

RMMVGTKAIATLSTGYLNALQYAKSRVQGADLTQMTDKTAPRVTITHHPDVRRSLMTQKA

YAEGLRALYLYTATFQDAAVAEVVHGVDAKLAVKVNDLMLPVVKGVGSEQAYAKLTESLQ

TLGGSGFLQDYPIEQYIRDAKIDSLYEGTTAIQAQDFFFRKIVRDKGVALAHVSGQIQEF

VDSGAGNGRLKTERALLAKALTDVQGMAAALTGYLMAAQQDVTSLYKVGLGSVRFLMSVG

DLIIGWLLQRQAAVAVAALDAGATGDERSFYEGKVAVASFFAKNFLPLLTSTREVIETLD

NDIMELDEAAF

>mti:MRGA423_01570 acyl-CoA dehydrogenase fadE5 (A)

MSHYRSNVRDQVFNLFEVLGVDKALGHGEFSDVDVDTARDMLAEVSRLAEGPVAESFVEG

DRNPPVFDPKTHSVMLPESFKKSVNAMLEAGWDKVGIDEALGGMPMPKAVVWALHEHILG

ANPAVWMYAGGAGFAQILYHLGTEEQKKWAVLAAERGWGSTMVLTEPDAGSDVGAARTKA

VQQADGSWHIDGVKRFITSGDSGDLFENIFHLVLARPEGAGPGTKGLSLYFVPKFLFDVE

TGEPGERNGVFVTNVEHKMGLKVSATCELAFGQHGVPAKGWLVGEVHNGIAQMFEVIEQA

RMMVGTKAIATLSTGYLNALQYAKSRVQGADLTQMTDKTAPRVTITHHPDVRRSLMTQKA

YAEGLRALYLYTATFQDAAVAEVVHGVDAKLAVKVNDLMLPVVKGVGSEQAYAKLTESLQ

TLGGSGFLQDYPIEQYIRDAKIDSLYEGTTAIQAQDFFFRKIVRDKGVALAHVSGQIQEF

VDSGAGNGRLKTERALLAKALTDVQGMAAALTGYLMAAQQDVTSLYKVGLGSVRFLMSVG

DLIIGWLLQRQAAVAVAALDAGATGDERSFYEGKVAVASFFAKNFLPLLTSTREVIETLD

NDIMELDEAAF

>mte:CCDC5079_0228 acyl-CoA dehydrogenase FADE5 (A)

MSHYRSNVRDQVFNLFEVLGVDKALGHGEFSDVDVDTARDMLAEVSRLAEGPVAESFVEG

DRNPPVFDPKTHSVMLPESFKKSVNAMLEAGWDKVGIDEALGGMPMPKAVVWALHEHILG

ANPAVWMYAGGAGFAQILYHLGTEEQKKWAVLAAERGWGSTMVLTEPDAGSDVGAARTKA

VQQADGSWHIDGVKRFITSGDSGDLFENIFHLVLARPEGAGPGTKGLSLYFVPKFLFDVE

TGEPGERNGVFVTNVEHKMGLKVSATCELAFGQHGVPAKGWLVGEVHNGIAQMFEVIEQA

RMMVGTKAIATLSTGYLNALQYAKSRVQGADLTQMTDKTAPRVTITHHPDVRRSLMTQKA

YAEGLRALYLYTATFQDAAVAEVVHGVDAKLAVKVNDLMLPVVKGVGSEQAYAKLTESLQ

TLGGSGFLQDYPIEQYIRDAKIDSLYEGTTAIQAQDFFFRKIVRDKGVALAHVSGQIQEF

VDSGAGNGRLKTERALLAKALTDVQGMAAALTGYLMAAQQDVTSLYKVGLGSVRFLMSVG

DLIIGWLLQRQAAVAVAALDAGATGDERSFYEGKVAVASFFAKNFLPLLTSTREVIETLD

NDIMELDEAAF

>mtur:CFBS_0261 fadE5; acyl-CoA dehydrogenase FadE5 (A)

MSHYRSNVRDQVFNLFEVLGVDKALGHGEFSDVDVDTARDMLAEVSRLAEGPVAESFVEG

DRNPPVFDPKTHSVMLPESFKKSVNAMLEAGWDKVGIDEALGGMPMPKAVVWALHEHILG

ANPAVWMYAGGAGFAQILYHLGTEEQKKWAVLAAERGWGSTMVLTEPDAGSDVGAARTKA

VQQADGSWHIDGVKRFITSGDSGDLFENIFHLVLARPEGAGPGTKGLSLYFVPKFLFDVE

TGEPGERNGVFVTNVEHKMGLKVSATCELAFGQHGVPAKGWLVGEVHNGIAQMFEVIEQA

RMMVGTKAIATLSTGYLNALQYAKSRVQGADLTQMTDKTAPRVTITHHPDVRRSLMTQKA

YAEGLRALYLYTATFQDAAVAEVVHGVDAKLAVKVNDLMLPVVKGVGSEQAYAKLTESLQ

TLGGSGFLQDYPIEQYIRDAKIDSLYEGTTAIQAQDFFFRKIVRDKGVALAHVSGQIQEF

VDSGAGNGRLKTERALLAKALTDVQGMAAALTGYLMAAQQDVTSLYKVGLGSVRFLMSVG

DLIIGWLLQRQAAVAVAALDAGATGDERSFYEGKVAVASFFAKNFLPLLTSTREVIETLD

NDIMELDEAAF

>mtl:CCDC5180_0226 acyl-CoA dehydrogenase FADE5 (A)

MSHYRSNVRDQVFNLFEVLGVDKALGHGEFSDVDVDTARDMLAEVSRLAEGPVAESFVEG

DRNPPVFDPKTHSVMLPESFKKSVNAMLEAGWDKVGIDEALGGMPMPKAVVWALHEHILG

ANPAVWMYAGGAGFAQILYHLGTEEQKKWAVLAAERGWGSTMVLTEPDAGSDVGAARTKA

VQQADGSWHIDGVKRFITSGDSGDLFENIFHLVLARPEGAGPGTKGLSLYFVPKFLFDVE

TGEPGERNGVFVTNVEHKMGLKVSATCELAFGQHGVPAKGWLVGEVHNGIAQMFEVIEQA

RMMVGTKAIATLSTGYLNALQYAKSRVQGADLTQMTDKTAPRVTITHHPDVRRSLMTQKA

YAEGLRALYLYTATFQDAAVAEVVHGVDAKLAVKVNDLMLPVVKGVGSEQAYAKLTESLQ

TLGGSGFLQDYPIEQYIRDAKIDSLYEGTTAIQAQDFFFRKIVRDKGVALAHVSGQIQEF

VDSGAGNGRLKTERALLAKALTDVQGMAAALTGYLMAAQQDVTSLYKVGLGSVRFLMSVG

DLIIGWLLQRQAAVAVAALDAGATGDERSFYEGKVAVASFFAKNFLPLLTSTREVIETLD

NDIMELDEAAF

>mto:MTCTRI2_0249 fadE5; acyl-CoA dehydrogenase FADE5 (A)

MSHYRSNVRDQVFNLFEVLGVDKALGHGEFSDVDVDTARDMLAEVSRLAEGPVAESFVEG

DRNPPVFDPKTHSVMLPESFKKSVNAMLEAGWDKVGIDEALGGMPMPKAVVWALHEHILG

ANPAVWMYAGGAGFAQILYHLGTEEQKKWAVLAAERGWGSTMVLTEPDAGSDVGAARTKA

VQQADGSWHIDGVKRFITSGDSGDLFENIFHLVLARPEGAGPGTKGLSLYFVPKFLFDVE

TGEPGERNGVFVTNVEHKMGLKVSPTCELAFGQHGVPAKGWLVGEVHNGIAQMFEVIEQA

RMMVGTKAIATLSTGYLNALQYAKSRVQGADLTQMTDKTAPRVTITHHPDVRRSLMTQKA

YAEGLRALYLYTATFQDAAVAEVVHGVDAKLAVKVNDLMLPVVKGVGSEQAYAKLTESLQ

TLGGSGFLQDYPIEQYIRDAKIDSLYEGTTAIQAQDFFFRKIVRDKGVALAHVSGQIQEF

VDSGAGNGRLKTERALLAKALTDVQGMAAALTGYLMAAQQDVTSLYKVGLGSVRFLMSVG

DLIIGWLLQRQAAVAVAALDAGATGDERSFYEGKVAVASFFAKNFLPLLTSTREVIETLD

NDIMELDEAAF

>mtd:UDA_0244c fadE5; fadE5 (A)

MSHYRSNVRDQVFNLFEVLGVDKALGHGEFSDVDVDTARDMLAEVSRLAEGPVAESFVEG

DRNPPVFDPKTHSVMLPESFKKSVNAMLEAGWDKVGIDEALGGMPMPKAVVWALHEHILG

ANPAVWMYAGGAGFAQILYHLGTEEQKKWAVLAAERGWGSTMVLTEPDAGSDVGAARTKA

VQQADGSWHIDGVKRFITSGDSGDLFENIFHLVLARPEGAGPGTKGLSLYFVPKFLFDVE

TGEPGERNGVFVTNVEHKMGLKVSATCELAFGQHGVPAKGWLVGEVHNGIAQMFEVIEQA

RMMVGTKAIATLSTGYLNALQYAKSRVQGADLTQMTDKTAPRVTITHHPDVRRSLMTQKA

YAEGLRALYLYTATFQDAAVAEVVHGVDAKLAVKVNDLMLPVVKGVGSEQAYAKLTESLQ

TLGGSGFLQDYPIEQYIRDAKIDSLYEGTTAIQAQDFFFRKIVRDKGVALAHVSGQIQEF

VDSGAGNGRLKTERALLAKALTDVQGMAAALTGYLMAAQQDVTSLYKVGLGSVRFLMSVG

DLIIGWLLQRQAAVAVAALDAGATGDERSFYEGKVAVASFFAKNFLPLLTSTREVIETLD

NDIMELDEAAF

>mtn:ERDMAN_0273 fadE5; acyl-CoA dehydrogenase (EC:1.3.99.-) (A)

MSHYRSNVRDQVFNLFEVLGVDKALGHGEFSDVDVDTARDMLAEVSRLAEGPVAESFVEG

DRNPPVFDPKTHSVMLPESFKKSVNAMLEAGWDKVGIDEALGGMPMPKAVVWALHEHILG

ANPAVWMYAGGAGFAQILYHLGTEEQKKWAVLAAERGWGSTMVLTEPDAGSDVGAARTKA

VQQADGSWHIDGVKRFITSGDSGDLFENIFHLVLARPEGAGPGTKGLSLYFVPKFLFDVE

TGEPGERNGVFVTNVEHKMGLKVSATCELAFGQHGVPAKGWLVGEVHNGIAQMFEVIEQA

RMMVGTKAIATLSTGYLNALQYAKSRVQGADLTQMTDKTAPRVTITHHPDVRRSLMTQKA

YAEGLRALYLYTATFQDAAVAEVVHGVDAKLAVKVNDLMLPVVKGVGSEQAYAKLTESLQ

TLGGSGFLQDYPIEQYIRDAKIDSLYEGTTAIQAQDFFFRKIVRDKGVALAHVSGQIQEF

VDSGAGNGRLKTERALLAKALTDVQGMAAALTGYLMAAQQDVTSLYKVGLGSVRFLMSVG

DLIIGWLLQRQAAVAVAALDAGATGDERSFYEGKVAVASFFAKNFLPLLTSTREVIETLD

NDIMELDEAAF

>mtj:J112_01325 acyl-CoA dehydrogenase (A)

MSHYRSNVRDQVFNLFEVLGVDKALGHGEFSDVDVDTARDMLAEVSRLAEGPVAESFVEG

DRNPPVFDPKTHSVMLPESFKKSVNAMLEAGWDKVGIDEALGGMPMPKAVVWALHEHILG

ANPAVWMYAGGAGFAQILYHLGTEEQKKWAVLAAERGWGSTMVLTEPDAGSDVGAARTKA

VQQADGSWHIDGVKRFITSGDSGDLFENIFHLVLARPEGAGPGTKGLSLYFVPKFLFDVE

TGEPGERNGVFVTNVEHKMGLKVSATCELAFGQHGVPAKGWLVGEVHNGIAQMFEVIEQA

RMMVGTKAIATLSTGYLNALQYAKSRVQGADLTQMTDKTAPRVTITHHPDVRRSLMTQKA

YAEGLRALYLYTATFQDAAVAEVVHGVDAKLAVKVNDLMLPVVKGVGSEQAYAKLTESLQ

TLGGSGFLQDYPIEQYIRDAKIDSLYEGTTAIQAQDFFFRKIVRDKGVALAHVSGQIQEF

VDSGAGNGRLKTERALLAKALTDVQGMAAALTGYLMAAQQDVTSLYKVGLGSVRFLMSVG

DLIIGWLLQRQAAVAVAALDAGATGDERSFYEGKVAVASFFAKNFLPLLTSTREVIETLD

NDIMELDEAAF

>mtub:MT7199_0249 putative ACYL-CoA DEHYDROGENASE FADE5 (EC:1.3.99.-) (A)

MSHYRSNVRDQVFNLFEVLGVDKALGHGEFSDVDVDTARDMLAEVSRLAEGPVAESFVEG

DRNPPVFDPKTHSVMLPESFKKSVNAMLEAGWDKVGIDEALGGMPMPKAVVWALHEHILG

ANPAVWMYAGGAGFAQILYHLGTEEQKKWAVLAAERGWGSTMVLTEPDAGSDVGAARTKA

VQQADGSWHIDGVKRFITSGDSGDLFENIFHLVLARPEGAGPGTKGLSLYFVPKFLFDVE

TGEPGERNGVFVTNVEHKMGLKVSATCELAFGQHGVPAKGWLVGEVHNGIAQMFEVIEQA

RMMVGTKAIATLSTGYLNALQYAKSRVQGADLTQMTDKTAPRVTITHHPDVRRSLMTQKA

YAEGLRALYLYTATFQDAAVAEVVHGVDAKLAVKVNDLMLPVVKGVGSEQAYAKLTESLQ

TLGGSGFLQDYPIEQYIRDAKVDSLYEGTTAIQAQDFFFRKIVRDKGVALAHVSGQIQEF

VDSGAGNGRLKTERALLAKALTDVQGMAAALTGYLMAAQQDVTSLYKVGLGSVRFLMSVG

DLIIGWLLQRQAAVAVAALDAGATGDERSFYEGKVAVVSFFAKNFLPLLTSTREVIETLD

NDIMELDEAAF

>mtuc:J113_22855 acyl-CoA dehydrogenase (A)

MVGWAGNPSFDLFKLPEEHDEMRSAIRALAEKEIAPHAAEVDEKARFPEEALVALNSSGF

NAVHIPEEYGGQGADSVATCIVIEEVARVDASASLIPAVNKLGTMGLILRGSEELKKQVL

PALAAEGAMASYALSEREAGSDAASMRTRAKADGDHWILNGAKCWITNGGKSTWYTVMAV

TDPDRGANGISAFMVHKDDEGFTVGPKERKLGIKGSPTTELYFENCRIPGDRIIGEPGTG

FKTALATLDHTRPTIGAQAVGIAQGALDAAIAYTKDRKQFGESISTFQAVQFMLADMAMK

VEAARLMVYSAAARAERGEPDLGFISAASKCFASDVAMEVTTDAVQLFGGAGYTTDFPVE

RFMRDAKITQIYEGTNQIQRVVMSRALLR

>mtue:J114_01335 acyl-CoA dehydrogenase (A)

MSHYRSNVRDQVFNLFEVLGVDKALGHGEFSDVDVDTARDMLAEVSRLAEGPVAESFVEG

DRNPPVFDPKTHSVMLPESFKKSVNAMLEAGWDKVGIDEALGGMPMPKAVVWALHEHILG

ANPAVWMYAGGAGFAQILYHLGTEEQKKWAVLAAERGWGSTMVLTEPDAGSDVGAARTKA

VQQADGSWHIDGVKRFITSGDSGDLFENIFHLVLARPEGAGPGTKGLSLYFVPKFLFDVE

TGEPGERNGVFVTNVEHKMGLKVSATCELAFGQHGVPAKGWLVGEVHNGIAQMFEVIEQA

RMMVGTKAIATLSTGYLNALQYAKSRVQGADLTQMTDKTAPRVTITHHPDVRRSLMTQKA

YAEGLRALYLYTATFQDAAVAEVVHGVDAKLAVKVNDLMLPVVKGVGSEQAYAKLTESLQ

TLGGSGFLQDYPIEQYIRDAKIDSLYEGTTAIQAQDFFFRKIVRDKGVALAHVSGQIQEF

VDSGAGNGRLKTERALLAKALTDVQGMAAALTGYLMAAQQDVTSLYKVGLGSVRFLMSVG

DLIIGWLLQRQAAVAVAALDAGATGDERSFYEGKVAVASFFAKNFLPLLTSTREVIETLD

NDIMELDEAAF

>mtx:M943_01300 butyryl-CoA dehydrogenase (A)

MSHYRSNVRDQVFNLFEVLGVDKALGHGEFSDVDVDTARDMLAEVSRLAEGPVAESFVEG

DRNPPVFDPKTHSVMLPESFKKSVNAMLEAGWDKVGIDEALGGMPMPKAVVWALHEHILG

ANPAVWMYAGGAGFAQILYHLGTEEQKKWAVLAAERGWGSTMVLTEPDAGSDVGAARTKA

VQQADGSWHIDGVKRFITSGDSGDLFENIFHLVLARPEGAGPGTKGLSLYFVPKFLFDVE

TGEPGERNGVFVTNVEHKMGLKVSATCELAFGQHGVPAKGWLVGEVHNGIAQMFEVIEQA

RMMVGTKAIATLSTGYLNALQYAKSRVQGADLTQMTDKTAPRVTITHHPDVRRSLMTQKA

YAEGLRALYLYTATFQDAAVAEVVHGVDAKLAVKVNDLMLPVVKGVGSEQAYAKLTESLQ

TLGGSGFLQDYPIEQYIRDAKIDSLYEGTTAIQAQDFFFRKIVRDKGVALAHVSGQIQEF

VDSGAGNGRLKTERALLAKALTDVQGMAAALTGYLMAAQQDVTSLYKVGLGSVRFLMSVG

DLIIGWLLQRQAAVAVAALDAGATGDERSFYEGKVAVASFFAKNFLPLLTSTREVIETLD

NDIMELDEAAF

>mtuh:I917_01765 Putative acyl-CoA dehydrogenase FadE5 (A)

MSHYRSNVRDQVFNLFEVLGVDKALXHGEFSDVDVDTARDMLAEVSRLAEGPVAESFVEG

DRNPPVFDPKTHSVMLPESFKKSVNAMLEAGWDKVGIDEALGGMPMPKAVVWALHEHILG

ANPAVWMYAGGAGFAQILYHLGTEEQKKWAVLAAERGWGSTMVLTEPDAGSDVGAARTKA

VQQADGSWHIDGVKRFITSGDSGDLFENIFHLVLARPEGAGPGTKGLSLYFVPKFLFDVE

TGEPGERNGVFVTNVEHKMGLKVSATCELAFGQHGVPAKGWLVGEVHNGIAQMFEVIEQA

RMMVGTKAIATLSTGYLNALQYAKSRVQGADLTQMTDKTAPRVTITHHPDVRRSLMTQKA

YAEGLRALYLYTATFQDAAVAEVVHGVDAKLAVKVNDLMLPVVKGVGSEQAYAKLTESLQ

TLGGSGFLQDYPIEQYIRDAKIDSLYEGTTAIQAQDFFFRKIVRDEXXXVGARVRPDPGV

RRQRCWQRPAEDRTRAAGAXALTDVQGMAAALTGYLMAAQQDVTSLYKVGLGSVRFLMSV

GDLIIGWXLQRQAAVAVAALDAGATGDERSFYEGKVAVASFFAKNFLPLLTSTREVIETL

DNDIMELDEAAF

>mtul:TBHG_00244 acyl-CoA dehydrogenase FadE5 (A)

MSHYRSNVRDQVFNLFEVLGVDKALGHGEFSDVDVDTARDMLAEVSRLAEGPVAESFVEG

DRNPPVFDPKTHSVMLPESFKKSVNAMLEAGWDKVGIDEALGGMPMPKAVVWALHEHILG

ANPAVWMYAGGAGFAQILYHLGTEEQKKWAVLAAERGWGSTMVLTEPDAGSDVGAARTKA

VQQADGSWHIDGVKRFITSGDSGDLFENIFHLVLARPEGAGPGTKGLSLYFVPKFLFDVE

TGEPGERNGVFVTNVEHKMGLKVSATCELAFGQHGVPAKGWLVGEVHNGIAQMFEVIEQA

RMMVGTKAIATLSTGYLNALQYAKSRVQGADLTQMTDKTAPRVTITHHPDVRRSLMTQKA

YAEGLRALYLYTATFQDAAVAEVVHGVDAKLAVKVNDLMLPVVKGVGSEQAYAKLTESLQ

TLGGSGFLQDYPIEQYIRDAKIDSLYEGTTAIQAQDFFFRKIVRDKGVALAHVSGQIQEF

VDSGAGNGRLKTERALLAKALTDVQGMAAALTGYLMAAQQDVTSLYKVGLGSVRFLMSVG

DLIIGWLLQRQAAVAVAALDAGATGDERSFYEGKVAVASFFAKNFLPLLTSTREVIETLD

NDIMELDEAAF

>mtut:HKBT1_0261 fadE5; acyl-CoA dehydrogenase FadE5 (A)

MSHYRSNVRDQVFNLFEVLGVDKALGHGEFSDVDVDTARDMLAEVSRLAEGPVAESFVEG

DRNPPVFDPKTHSVMLPESFKKSVNAMLEAGWDKVGIDEALGGMPMPKAVVWALHEHILG

ANPAVWMYAGGAGFAQILYHLGTEEQKKWAVLAAERGWGSTMVLTEPDAGSDVGAARTKA

VQQADGSWHIDGVKRFITSGDSGDLFENIFHLVLARPEGAGPGTKGLSLYFVPKFLFDVE

TGEPGERNGVFVTNVEHKMGLKVSATCELAFGQHGVPAKGWLVGEVHNGIAQMFEVIEQA

RMMVGTKAIATLSTGYLNALQYAKSRVQGADLTQMTDKTAPRVTITHHPDVRRSLMTQKA

YAEGLRALYLYTATFQDAAVAEVVHGVDAKLAVKVNDLMLPVVKGVGSEQAYAKLTESLQ

TLGGSGFLQDYPIEQYIRDAKIDSLYEGTTAIQAQDFFFRKIVRDKGVALAHVSGQIQEF

VDSGAGNGRLKTERALLAKALTDVQGMAAALTGYLMAAQQDVTSLYKVGLGSVRFLMSVG

DLIIGWLLQRQAAVAVAALDAGATGDERSFYEGKVAVASFFAKNFLPLLTSTREVIETLD

NDIMELDEAAF

>mtuu:HKBT2_0261 fadE5; acyl-CoA dehydrogenase FadE5 (A)

MSHYRSNVRDQVFNLFEVLGVDKALGHGEFSDVDVDTARDMLAEVSRLAEGPVAESFVEG

DRNPPVFDPKTHSVMLPESFKKSVNAMLEAGWDKVGIDEALGGMPMPKAVVWALHEHILG

ANPAVWMYAGGAGFAQILYHLGTEEQKKWAVLAAERGWGSTMVLTEPDAGSDVGAARTKA

VQQADGSWHIDGVKRFITSGDSGDLFENIFHLVLARPEGAGPGTKGLSLYFVPKFLFDVE

TGEPGERNGVFVTNVEHKMGLKVSATCELAFGQHGVPAKGWLVGEVHNGIAQMFEVIEQA

RMMVGTKAIATLSTGYLNALQYAKSRVQGADLTQMTDKTAPRVTITHHPDVRRSLMTQKA

YAEGLRALYLYTATFQDAAVAEVVHGVDAKLAVKVNDLMLPVVKGVGSEQAYAKLTESLQ

TLGGSGFLQDYPIEQYIRDAKIDSLYEGTTAIQAQDFFFRKIVRDKGVALAHVSGQIQEF

VDSGAGNGRLKTERALLAKALTDVQGMAAALTGYLMAAQQDVTSLYKVGLGSVRFLMSVG

DLIIGWLLQRQAAVAVAALDAGATGDERSFYEGKVAVASFFAKNFLPLLTSTREVIETLD

NDIMELDEAAF

>mtq:HKBS1_0261 fadE5; acyl-CoA dehydrogenase FadE5 (A)

MSHYRSNVRDQVFNLFEVLGVDKALGHGEFSDVDVDTARDMLAEVSRLAEGPVAESFVEG

DRNPPVFDPKTHSVMLPESFKKSVNAMLEAGWDKVGIDEALGGMPMPKAVVWALHEHILG

ANPAVWMYAGGAGFAQILYHLGTEEQKKWAVLAAERGWGSTMVLTEPDAGSDVGAARTKA

VQQADGSWHIDGVKRFITSGDSGDLFENIFHLVLARPEGAGPGTKGLSLYFVPKFLFDVE

TGEPGERNGVFVTNVEHKMGLKVSATCELAFGQHGVPAKGWLVGEVHNGIAQMFEVIEQA

RMMVGTKAIATLSTGYLNALQYAKSRVQGADLTQMTDKTAPRVTITHHPDVRRSLMTQKA

YAEGLRALYLYTATFQDAAVAEVVHGVDAKLAVKVNDLMLPVVKGVGSEQAYAKLTESLQ

TLGGSGFLQDYPIEQYIRDAKIDSLYEGTTAIQAQDFFFRKIVRDKGVALAHVSGQIQEF

VDSGAGNGRLKTERALLAKALTDVQGMAAALTGYLMAAQQDVTSLYKVGLGSVRFLMSVG

DLIIGWLLQRQAAVAVAALDAGATGDERSFYEGKVAVASFFAKNFLPLLTSTREVIETLD

NDIMELDEAAF

>mbo:Mb0250c fadE5; acyl-CoA dehydrogenase (EC:1.3.99.-) (A)

MSHYRSNVRDQVFNLFEVLGVDKALGHGEFSDVDVDTARDMLAEVSRLAEGPVAESFVEG

DRNPPVFDPKTHSVMLPESFKKSVNAMLEAGWDKVGIDEALGGMPMPKAVVWALHEHILG

ANPAVWMYAGGAGFAQILYHLGTEEQKKWAVLAAERGWGSTMVLTEPDAGSDVGAARTKA

VQQADGSWHIDGVKRFITSGDSGDLFENIFHLVLARPEGAGPGTKGLSLYFVPKFLFDVE

TGEPGERNGVFVTNVEHKMGLKVSATCELAFGQHGVPAKGWLVGEVHNGIAQMFEVIEQA

RMMVGTKAIATLSTGYLNALQYAKSRVQGADLTQMTDKTAPRVTITHHPDVRRSLMTQKA

YAEGLRALYLYTATFQDAAVAEVVHGVDAKLAVRVNDLMLPVVKGVGSEQAYAKLTESLQ

TLGGSGFLQDYPIEQYIRDAKIDSLYEGTTAIQAQDFFFRKIVRDKGVALAHVSGQIQAF

VDSGAGNGRLKTERALLAKALTDVQGMAAALTGYLMAAQQDVTSLYKVGLGSVRFLMSVG

DLIIGWLLQRQAAVAVAALDAGATGDERSFYEGKVAVASFFAKNFLPLLTSTREVIETLD

NDIMELDEAAF

>mbb:BCG_0282c fadE5; Probable acyl-CoA dehydrogenase fadE5 (EC:1.3.99.-) (A)

MSHYRSNVRDQVFNLFEVLGVDKALGHGEFSDVDVDTARDMLAEVSRLAEGPVAESFVEG

DRNPPVFDPKTHSVMLPESFKKSVNAMLEAGWDKVGIDEALGGMPMPKAVVWALHEHILG

ANPAVWMYAGGAGFAQILYHLGTEEQKKWAVLAAERGWGSTMVLTEPDAGSDVGAARTKA

VQQADGSWHIDGVKRFITSGDSGDLFENIFHLVLARPEGAGPGTKGLSLYFVPKFLFDVE

TGEPGERNGVFVTNVEHKMGLKVSATCELAFGQHGVPAKGWLVGEVHNGIAQMFEVIEQA

RMMVGTKAIATLSTGYLNALQYAKSRVQGADLTQMTDKTAPRVTITHHPDVRRSLMTQKA

YAEGLRALYLYTATFQDAAVAEVVHGVDAKLAVRVNDLMLPVVKGVGSEQAYAKLTESLQ

TLGGSGFLQDYPIEQYIRDAKIDSLYEGTTAIQAQDFFFRKIVRDKGVALAHVSGQIQAF

VDSGAGNGRLKTERALLAKALTNVQGMAAALTGYLMAAQQDVTSLYKVGLGSVRFLMSVG

DLIIGWLLQRQAAVAVAALDAGATGDERSFYEGKVAVASFFAKNFLPLLTSTREVIETLD

NDIMELDEAAF

>mbt:JTY_0251 fadE5; putative acyl-CoA dehydrogenase (A)

MSHYRSNVRDQVFNLFEVLGVDKALGHGEFSDVDVDTARDMLAEVSRLAEGPVAESFVEG

DRNPPVFDPKTHSVMLPESFKKSVNAMLEAGWDKVGIDEALGGMPMPKAVVWALHEHILG

ANPAVWMYAGGAGFAQILYHLGTEEQKKWAVLAAERGWGSTMVLTEPDAGSDVGAARTKA

VQQADGSWHIDGVKRFITSGDSGDLFENIFHLVLARPEGAGPGTKGLSLYFVPKFLFDVE

TGEPGERNGVFVTNVEHKMGLKVSATCELAFGQHGVPAKGWLVGEVHNGIAQMFEVIEQA

RMMVGTKAIATLSTGYLNALQYAKSRVQGADLTQMTDKTAPRVTITHHPDVRRSLMTQKA

YAEGLRALYLYTATFQDAAVAEVVHGVDAKLAVRVNDLMLPVVKGVGSEQAYAKLTESLQ

TLGGSGFLQDYPIEQYIRDAKIDSLYEGTTAIQAQDFFFRKIVRDKGVALAHVSGQIQAF

VDSGAGNGRLKTERALLAKALTNVQGMAAALTGYLMAAQQDVTSLYKVGLGSVRFLMSVG

DLIIGWLLQRQAAVAVAALDAGATGDERSFYEGKVAVASFFAKNFLPLLTSTREVIETLD

NDIMELDEAAF

>mbm:BCGMEX_0251c fadE5; putative acyl-CoA dehydrogenase (A)

MSHYRSNVRDQVFNLFEVLGVDKALGHGEFSDVDVDTARDMLAEVSRLAEGPVAESFVEG

DRNPPVFDPKTHSVMLPESFKKSVNAMLEAGWDKVGIDEALGGMPMPKAVVWALHEHILG

ANPAVWMYAGGAGFAQILYHLGTEEQKKWAVLAAERGWGSTMVLTEPDAGSDVGAARTKA

VQQADGSWHIDGVKRFITSGDSGDLFENIFHLVLARPEGAGPGTKGLSLYFVPKFLFDVE

TGEPGERNGVFVTNVEHKMGLKVSATCELAFGQHGVPAKGWLVGEVHNGIAQMFEVIEQA

RMMVGTKAIATLSTGYLNALQYAKSRVQGADLTQMTDKTAPRVTITHHPDVRRSLMTQKA

YAEGLRALYLYTATFQDAAVAEVVHGVDAKLAVRVNDLMLPVVKGVGSEQAYAKLTESLQ

TLGGSGFLQDYPIEQYIRDAKIDSLYEGTTAIQAQDFFFRKIVRDKGVALAHVSGQIQAF

VDSGAGNGRLKTERALLAKALTNVQGMAAALTGYLMAAQQDVTSLYKVGLGSVRFLMSVG

DLIIGWLLQRQAAVAVAALDAGATGDERSFYEGKVAVASFFAKNFLPLLTSTREVIETLD

NDIMELDEAAF

>mbk:K60_002660 acyl-CoA dehydrogenase fadE5 (A)

MSHYRSNVRDQVFNLFEVLGVDKALGHGEFSDVDVDTARDMLAEVSRLAEGPVAESFVEG

DRNPPVFDPKTHSVMLPESFKKSVNAMLEAGWDKVGIDEALGGMPMPKAVVWALHEHILG

ANPAVWMYAGGAGFAQILYHLGTEEQKKWAVLAAERGWGSTMVLTEPDAGSDVGAARTKA

VQQADGSWHIDGVKRFITSGDSGDLFENIFHLVLARPEGAGPGTKGLSLYFVPKFLFDVE

TGEPGERNGVFVTNVEHKMGLKVSATCELAFGQHGVPAKGWLVGEVHNGIAQMFEVIEQA

RMMVGTKAIATLSTGYLNALQYAKSRVQGADLTQMTDKTAPRVTITHHPDVRRSLMTQKA

YAEGLRALYLYTATFQDAAVAEVVHGVDAKLAVRVNDLMLPVVKGVGSEQAYAKLTESLQ

TLGGSGFLQDYPIEQYIRDAKIDSLYEGTTAIQAQDFFFRKIVRDKGVALAHVSGQIQAF

VDSGAGNGRLKTERALLAKALTNVQGMAAALTGYLMAAQQDVTSLYKVGLGSVRFLMSVG

DLIIGWLLQRQAAVAVAALDAGATGDERSFYEGKVAVASFFAKNFLPLLTSTREVIETLD

NDIMELDEAAF

>mbx:BCGT_0009 3-methylmercaptopropionyl-CoA dehydrogenase (DmdC) (A)

MSHYRSNVRDQVFNLFEVLGVDKALGHGEFSDVDVDTARDMLAEVSRLAEGPVAESFVEG

DRNPPVFDPKTHSVMLPESFKKSVNAMLEAGWDKVGIDEALGGMPMPKAVVWALHEHILG

ANPAVWMYAGGAGFAQILYHLGTEEQKKWAVLAAERGWGSTMVLTEPDAGSDVGAARTKA

VQQADGSWHIDGVKRFITSGDSGDLFENIFHLVLARPEGAGPGTKGLSLYFVPKFLFDVE

TGEPGERNGVFVTNVEHKMGLKVSATCELAFGQHGVPAKGWLVGEVHNGIAQMFEVIEQA

RMMVGTKAIATLSTGYLNALQYAKSRVQGADLTQMTDKTAPRVTITHHPDVRRSLMTQKA

YAEGLRALYLYTATFQDAAVAEVVHGVDAKLAVRVNDLMLPVVKGVGSEQAYAKLTESLQ

TLGGSGFLQDYPIEQYIRDAKIDSLYEGTTAIQAQDFFFRKIVRDKGVALAHVSGQIQAF

VDSGAGNGRLKTERALLAKALTNVQGMAAALTGYLMAAQQDVTSLYKVGLGSVRFLMSVG

DLIIGWLLQRQAAVAVAALDAGATGDERSFYEGKVAVASFFAKNFLPLLTSTREVIETLD

NDIMELDEAAF

>mbz:LH58_01345 butyryl-CoA dehydrogenase (A)

MSHYRSNVRDQVFNLFEVLGVDKALGHGEFSDVDVDTARDMLAEVSRLAEGPVAESFVEG

DRNPPVFDPKTHSVMLPESFKKSVNAMLEAGWDKVGIDEALGGMPMPKAVVWALHEHILG

ANPAVWMYAGGAGFAQILYHLGTEEQKKWAVLAAERGWGSTMVLTEPDAGSDVGAARTKA

VQQADGSWHIDGVKRFITSGDSGDLFENIFHLVLARPEGAGPGTKGLSLYFVPKFLFDVE

TGEPGERNGVFVTNVEHKMGLKVSATCELAFGQHGVPAKGWLVGEVHNGIAQMFEVIEQA

RMMVGTKAIATLSTGYLNALQYAKSRVQGADLTQMTDKTAPRVTITHHPDVRRSLMTQKA

YAEGLRALYLYTATFQDAAVAEVVHGVDAKLAVRVNDLMLPVVKGVGSEQAYAKLTESLQ

TLGGSGFLQDYPIEQYIRDAKIDSLYEGTTAIQAQDFFFRKIVRDKGVALAHVSGQIQAF

VDSGAGNGRLKTERALLAKALTDVQGMAAALTGYLMAAQQDVTSLYKVGLGSVRFLMSVG

DLIIGWLLQRQAAVAVAALDAGATGDERSFYEGKVAVASFFAKNFLPLLTSTREVIETLD

NDIMELDEAAF

>maf:MAF_02450 fadE5; putative acyl-CoA dehydrogenase FADE5 (EC:1.3.99.-) (A)

MSHYRSNVRDQVFNLFEVLGVDKALGHGEFSDVDVDTARDMLAEVSRLAEGPVAESFVEG

DRNPPVFDPKTHSVMLPESFKKSVNAMLEAGWDKVGIDEALGGMPMPKAVVWALHEHILG

ANPAVWMYAGGAGFAQILYHLGTEEQKKWAVLAAERGWGSTMVLTEPDAGSDVGAARTKA

VQQADGSWHIDGVKRFITSGDSGDLFENIFHLVLARPEGAGPGTKGLSLYFVPKFLFDVE

TGEPGERNGVFVTNVEHKMGLKVSATCELAFGQHGVPAKGWLVGEVYNGIAQMFEVIEQA

RMMVGTKAIATLSTGYLNALQYAKSRVQGADLTQMTDKTAPRVTITHHPDVRRSLMTQKA

YAEGLRALYLYTATFQDAAVAEVVHGVDAKLAVKVNDLMLPVVKGVGSEQAYAKLTESLQ

TLGGSGFLQDYPIEQYIRDAKIDSLYEGTTAIQAQDFFFRKIVRDKGVALAHVSGQIQEF

VDSGAGNGRLKTERALLAKALTDVQGMAAALTGYLMAAQQDVTSLYKVGLGSVRFLMSVG

DLIIGWLLQRQAAVAVAALDAGATGDERSFYEGKVAVASFFAKNFLPLLTSTREVIETLD

NDIMELDEAAF

>mce:MCAN_02511 fadE5; putative acyl-CoA dehydrogenase FADE5 (A)

MSHYRSNVRDQVFNLFEVLGVDKALGHGEFSDVDVDTARDMLAEVSRLAEGPVAESFVEG

DRNPPVFDPKTHSVMLPESFKKSVNAMLEAGWDKVGIDEALGGMPMPKAVVWALHEHILG

ANPAVWMYAGGAGFAQILYHLGTEEQKKWAVLAAERGWGSTMVLTEPDAGSDVGAARTKA

VQQADGSWHIDGVKRFITSGDSGDLFENIFHLVLARPEGAGPGTKGLSLYFVPKFLFDAE

TGEPGERNGVFVTNVEHKMGLKVSTTCELAFGQHGVPAKGWLVGEVHNGIAQMFEVIEQA

RMMVGTKAIATLSTGYLNALQYAKSRVQGADLTQMTDKTAPRVTITHHPDVRRSLMTQKA

YAEGLRALYLYTATFQDAAVAEVVHGVDAKLAVKVNDLMLPVVKGVGSEQAYAKLTESLQ

TLGGSGFLQDYPIEQYIRDAKIDSLYEGTTAIQAQDFFFRKIVRDKGVALAHVSGQIQEF

VDSGAGNGRLKTERALLAKALTDVQGMAAALTGYLMAAQQDVTSLYKVGLGSVRFLMSVG

DLIIGWLLQRQAAVGVAALDAGATGDERSFYEGKVAVASFFAKNFLPLLTSTREVIETLD

NDIMELDEAAF

>mcq:BN44_10281 fadE; Putative acyl-CoA dehydrogenase FadE5 (EC:1.3.99.-) (A)

MSHYRSNVRDQVFNLFEVLGVDKALGHGEFSDVDVDTARDMLAEVSRLAEGPVAESFVEG

DRNPPVFDPKTHSVMLPESFKKSVNAMLEAGWDKVGIDEALGGMPMPKAVVWALHEHILG

ANPAVWMYAGGAGFAQILYHLGTEEQKKWAVLAAERGWGSTMVLTEPDAGSDVGAARTKA

VQQADGSWHIDGVKRFITSGDSGDLFENIFHLVLARPEGAGPGTKGLSLYFVPKFLFDVE

TGEPGERNGVFVTNVEHKMGLKVSATCELAFGQHGVPAKGWLVGEVHNGIAQMFEVIEQA

RMMVGTKAIATLSTGYLNALQYAKSRVQGADLTQMTDKTAPRVTITHHPDVRRSLMTQKA

YAEGLRALYLYTATFQDAAVAEVVHGVDAKLAVKVNDLMLPVVKGVGSEQAYAKLTESLQ

TLGGSGFLQDYPIEQYIRDAKIDSLYEGTTAIQAQDFFFRKIVRDKGVALAHVSGQIQEF

VDSGAGNGRLKTERALLAKALTDVQGMAAALTGYLMAAQQDVTSLYKVGLGSVRFLMSVG

DLIIGWLLQRQAAVGVAALDAGATGDERSFYEGKVAVASFFAKNFLPLLTSTREVIETLD

NDIMELDEAAF

>mcv:BN43_10276 fadE; Putative acyl-CoA dehydrogenase FadE5 (EC:1.3.99.-) (A)

MSHYRSNVRDQVFNLFEVLGVDKALGHGEFSDVDVDTARDMLAEVSRLAEGPVAESFVEG

DRNPPVFDPKSHSVMLPESFKKSVNAMLEAGWDKVGIDEALGGMPMPKAVVWALHEHILG

ANPAVWMYAGGAGFAQILYHLGTEEQKKWAVLAAERGWGSTMVLTEPDAGSDVGAARTKA

VQQADGSWHIDGVKRFITSGDSGDLFENIFHLVLARPEGAGPGTKGLSLYFVPKFLFDVE

TGEPGERNGVFVTNVEHKMGLKVSATCELAFGQHGVPAKGWLVGEVHNGIAQMFEVIEQA

RMMVGTKAIATLSTGYLNALQYAKSRVQGADLTQMTDKTAPRVTITHHPDVRRSLMTQKA

YAEGLRALYLYTATFQDAAVAEVVHGVDAKLAVKVNDLMLPVVKGVGSEQAYAKLTESLQ

TLGGSGFLQDYPIEQYIRDAKIDSLYEGTTAIQAQDFFFRKIVRDKGVALAHVSGQIQEF

VDSGAGNGRLKTERALLAKALTDVQGMAAALTGYLMAAQQDVTSLYKVGLGSVRFLMSVG

DLIIGWLLQRQAAVAVAALDAGATGDERSFYEGKVAVASFFAKNFLPLLTSTREVIETLD

NDIMELDEAAF

>mcx:BN42_10288 fadE; Putative acyl-CoA dehydrogenase FadE5 (EC:1.3.99.-) (A)

MSHYRSNVRDQVFNLFEVLGVDKALGHGEFSDVDVDTARDMLAEVSRLAEGPVAESFVEG

DRNPPVFDPKTHSVMLPESFKKSVNAMLEAGWDKVGIDEALGGMPMPKAVVWALHEHILG

ANPAVWMYAGGAGFAQILYHLGTEEQKKWAVLAAERGWGSTMVLTEPDAGSDVGAARTKA

VQQADGSWHIDGVKRFITSGDSGDLFENIFHLVLARPEGAGPGTKGLSLYFVPKFLFDVE

TGEPGERNGVFVTNVEHKMGLKVSATCELAFGQHGVPAKGWLVGEVHNGIAQMFEVIEQA

RMMVGTKAIATLSTGYLNALQYAKSRVQGADLTQMTDKTAPRVTITHHPDVRRSLMTQKA

YAEGLRALYLYTATFQDAAVAEVVHGVDAKLAVKVNDLMLPVVKGVGSEQAYAKLTESLQ

TLGGSGFLQDYPIEQYIRDAKIDSLYEGTTAIQAQDFFFRKIVRDKGVALAHVSGQIQEF

VDSGSGNGRLKTERALLAKALTDVQAMAAALTGHLMAAQQDVTSLYKVGLGSVRFLMSVG

DLIIGWLLQRQAAVAVAALDSGATGDERSFYEGKVAVASFFAKNFLPLLTSTREVIETLD

NDIMELDEAAF

>mcz:BN45_10268 fadE; Putative acyl-CoA dehydrogenase FadE5 (EC:1.3.99.-) (A)

MSHYRSNVRDQVFNLFEVLGVDKALGHGEFSDVDVDTARDMLAEVSRLAEGPVAESFVEG

DRNPPVFDPKTHSVMLPESFKKSVNAMLEAGWDKVGIDEALGGMPMPKAVVWALHEHILG

ANPAVWMYAGGAGFAQILYHLGTEEQKKWAVLAAERGWGSTMVLTEPDAGSDVGAARTKA

VQQADGSWHIDGVKRFITSGDSGDLFENIFHLVLARPEGAGPGTKGLSLYFVPKFLFDVE

TGEPGERNGVFVTNVEHKMGLKVSATCELAFGQHGVPAKGWLVGEVHNGIAQMFEVIEQA

RMMVGTKAIATLSTGYLNALQYAKSRVQGADLTQMTDKTAPRVTITHHPDVRRSLMTQKA

YAEGLRALYLYTATFQDAAVAEAVHGVDAKLAVKVNDLMLPVVKGVGSEQAYAKLTESLQ

TLGGSGFLQDYPIEQYIRDAKIDSLYEGTTAIQAQDFFFRKIVRDKGVALAYVSGQIQEF

VDSGSGNGRLKTERALLAKALTDVQGMAAALTGYLMAAQQDVTSLYKVGLGSVRFLMSVG

DLIIGWLLQRQAAVAVAALDAGATGDERSFYEGKVAVASFFAKNFLPLLTSTREVIETLD

NDIMELDEAAF

>mle:ML2563 fadE5; acyl-CoA dehydrogenase (A)

MSHYKSNVRDQVFNLFEVLGVDKAFGTDEFSNLDADTAYEMLTEVSRLAEGPVAASFAEG

DRNPPVFHPETHSITLPESFKKSCRAVAEAGWSKAGIDEALGGTPMPKALLWALHEHLMG

ANPAVWIYAGGAGFANILYHLGTEKQKKWAVWAAERNWGSTMVLTEPDAGSDVGAGRTKA

VHQEDDSWHIDGVKRFITSGDSDDLFENIFHLVLARPEGAGPGTKGLSLFFVPKFLFDFE

TGELGERNGVFVTNVEHKMGLKVSATCELSFGQHGVPAKGWLVGDVHDGIAQMFEVIEQA

RMMVGTKAIATLSTGYLNALEYAKSRVQGADLTQMTDKTAPRVTITHHPDVRRSLMTQKA

YAEGLRALYLYTATFQDAAVAEAVHGVDAKLAVKINDLMLPLVKGVGSEQAYAKLTESLQ

TLGGSGFLQDYPIEQYIRDAKIDSLYEGTTAIQAQDFFFRKIVRDKGVALSYVSGQIQQF

VDSETGNGRLKSERELLAKALTDIQGMEASLTGYLMAAQQDVTSLYKVGLGSVRFLMSVG

DLIIGWLLQRQAAVAVQALDAGASSAERSFYEGKVAVSSFFAKNFLPLLTSTREVLEVLD

NDIMELDEAAF

>mlb:MLBr02563 fadE5; acyl-CoA dehydrogenase; K00257 [EC:1.3.99.-] (A)

MSHYKSNVRDQVFNLFEVLGVDKAFGTDEFSNLDADTAYEMLTEVSRLAEGPVAASFAEG

DRNPPVFHPETHSITLPESFKKSCRAVAEAGWSKAGIDEALGGTPMPKALLWALHEHLMG

ANPAVWIYAGGAGFANILYHLGTEKQKKWAVWAAERNWGSTMVLTEPDAGSDVGAGRTKA

VHQEDDSWHIDGVKRFITSGDSDDLFENIFHLVLARPEGAGPGTKGLSLFFVPKFLFDFE

TGELGERNGVFVTNVEHKMGLKVSATCELSFGQHGVPAKGWLVGDVHDGIAQMFEVIEQA

RMMVGTKAIATLSTGYLNALEYAKSRVQGADLTQMTDKTAPRVTITHHPDVRRSLMTQKA

YAEGLRALYLYTATFQDAAVAEAVHGVDAKLAVKINDLMLPLVKGVGSEQAYAKLTESLQ

TLGGSGFLQDYPIEQYIRDAKIDSLYEGTTAIQAQDFFFRKIVRDKGVALSYVSGQIQQF

VDSETGNGRLKSERELLAKALTDIQGMEASLTGYLMAAQQDVTSLYKVGLGSVRFLMSVG

DLIIGWLLQRQAAVAVQALDAGASSAERSFYEGKVAVSSFFAKNFLPLLTSTREVLEVLD

NDIMELDEAAF

>mpa:MAP_3694c fadE5; FadE5 (A)

MSHYKSNVRDQEFNLFEVLGVDKALGQGEFSDLDADTAREMLNEISRLAEGPIADSFVEG

DRNPPVFDPKTHSVTLPESFKKSVHAVIEAGWDKVGIDEALGGVAMPKALLWALHEHILG

ANPAVWMYAGGAGFANILYHLGTDEQKKWAVMAAERGWGSTMVLTEPDAGSDVGAGRTKA

VKQDDGSWHIDGVKRFITSADSDDLFENIFHLVLARPEGAGPGTKGLSLFFVPKFLFDFE

TGELGERNGVFVTNVEHKMGLKVSATCELSFGQHDVPAKGWLVGEVHNGIAQMFEVIEQA

RMMVGTKAIATLSTGYLNALEYAKSRVQGADMTQMTDKTAPRVTITHHPDVRRSLMTQKA

YAEGLRALYLFTSTYQDAAVAEALYGVDAELAVKVNDLMLPVVKGVGSEQAYAKLTESLQ

TFGGSGFLQDYPIEQYIRDSKIDSLYEGTTAIQAQDFFFRKIVRDKGVALAHVSEQIQKF

VDSESGNGRLKSERELLAKALADVQGMATALTGYLMAAQEDVTSLYKVGLGSVRFLMSVG

DLIIGWLLQRQAAVAVAALDAGASGEDRSFYEGKIAVASFFAKNFLPMLTSTREVIETLD

NDIMELDEAAF

>mao:MAP4_0077 acyl-CoA dehydrogenase (A)

MSHYKSNVRDQEFNLFEVLGVDKALGQGEFSDLDADTAREMLNEISRLAEGPIADSFVEG

DRNPPVFDPKTHSVTLPESFKKSVHAVIEAGWDKVGIDEALGGVAMPKALLWALHEHILG

ANPAVWMYAGGAGFANILYHLGTDEQKKWAVMAAERGWGSTMVLTEPDAGSDVGAGRTKA

VKQDDGSWHIDGVKRFITSADSDDLFENIFHLVLARPEGAGPGTKGLSLFFVPKFLFDFE

TGELGERNGVFVTNVEHKMGLKVSATCELSFGQHDVPAKGWLVGEVHNGIAQMFEVIEQA

RMMVGTKAIATLSTGYLNALEYAKSRVQGADMTQMTDKTAPRVTITHHPDVRRSLMTQKA

YAEGLRALYLFTSTYQDAAVAEALYGVDAELAVKVNDLMLPVVKGVGSEQAYAKLTESLQ

TFGGSGFLQDYPIEQYIRDSKIDSLYEGTTAIQAQDFFFRKIVRDKGVALAHVSEQIQKF

VDSESGNGRLKSERELLAKALADVQGMATALTGYLMAAQEDVTSLYKVGLGSVRFLMSVG

DLIIGWLLQRQAAVAVAALDAGASGEDRSFYEGKIAVASFFAKNFLPMLTSTREVIETLD

NDIMELDEAAF

>mavi:RC58_00360 butyryl-CoA dehydrogenase (A)

MSHYKSNVRDQEFNLFEVLGVDKALGQGEFSDLDADTAREMLNEISRLAEGPIADSFVEG

DRNPPVFDPKTHSVTLPESFKKSVHAVIEAGWDKVGIDEALGGVAMPKALLWALHEHILG

ANPAVWMYAGGAGFANILYHLGTDEQKKWAVMAAERGWGSTMVLTEPDAGSDVGAGRTKA

VKQDDGSWHIDGVKRFITSADSDDLFENIFHLVLARPEGAGPGTKGLSLFFVPKFLFDFE

TGELGERNGVFVTNVEHKMGLKVSATCELSFGQHDVPAKGWLVGEVHNGIAQMFEVIEQA

RMMVGTKAIATLSTGYLNALEYAKSRVQGADMTQMTDKTAPRVTITHHPDVRRSLMTQKA

YAEGLRALYLFTSTYQDAAVAEALYGVDAELAVKVNDLMLPVVKGVGSEQAYAKLTESLQ

TFGGSGFLQDYPIEQYIRDSKIDSLYEGTTAIQAQDFFFRKIVRDKGVALAHVSEQIQKF

VDSESGNGRLKSERELLAKALADVQGMATALTGYLMAAQEDVTSLYKVGLGSVRFLMSVG

DLIIGWLLQRQAAVAVAALDAGASGEDRSFYEGKIAVASFFAKNFLPMLTSTREVIETLD

NDIMELDEAAF

>mavu:RE97_00365 butyryl-CoA dehydrogenase (A)

MSHYKSNVRDQEFNLFEVLGVDKALGQGEFSDLDADTAREMLNEISRLAEGPIADSFVEG

DRNPPVFDPKTHSVTLPESFKKSVHAVIEAGWDKVGIDEALGGVAMPKALLWALHEHILG

ANPAVWMYAGGAGFANILYHLGTDEQKKWAVMAAERGWGSTMVLTEPDAGSDVGAGRTKA

VKQDDGSWHIDGVKRFITSADSDDLFENIFHLVLARPEGAGPGTKGLSLFFVPKFLFDFE

TGELGERNGVFVTNVEHKMGLKVSATCELSFGQHDVPAKGWLVGEVHNGIAQMFEVIEQA

RMMVGTKAIATLSTGYLNALEYAKSRVQGADMTQMTDKTAPRVTITHHPDVRRSLMTQKA

YAEGLRALYLFTSTYQDAAVAEALYGVDAELAVKVNDLMLPVVKGVGSEQAYAKLTESLQ

TFGGSGFLQDYPIEQYIRDSKIDSLYEGTTAIQAQDFFFRKIVRDKGVALAHVSEQIQKF

VDSESGNGRLKSERELLAKALADVQGMATALTGYLMAAQEDVTSLYKVGLGSVRFLMSVG

DLIIGWLLQRQAAVAVAALDAGASGEDRSFYEGKIAVASFFAKNFLPMLTSTREVIETLD

NDIMELDEAAF

>mav:MAV_4914 putative acyl-CoA dehydrogenase (A)

MSHYKSNVRDQEFNLFEVLGVDKALGQGEFSDLDADTAREMLNEISRLAEGPIADSFVEG

DRNPPVFDPKTHSVTLPESFKKSVHAVIEAGWDKVGIDEALGGVAMPKALLWALHEHILG

ANPAVWMYAGGAGFANILYHLGTDEQKKWAVMAAERGWGSTMVLTEPDAGSDVGAGRTKA

VKQDDGSWHIDGVKRFITSADSDDLFENIFHLVLARPEGAGPGTKGLSLFFVPKFLFDFE

TGELGERNGVFVTNVEHKMGLKVSATCELSFGQHDVPAKGWLVGEVHNGIAQMFEVIEQA

RMMVGTKAIATLSTGYLNALEYAKSRVQGADMTQMTDKTAPRVTITHHPDVRRSLMTQKA

YAEGLRALYLFTSTYQDAAVAEALYGVDAELAVKVNDLMLPVVKGVGSEQAYAKLTESLQ

TFGGSGFLQDYPIEQYIRDSKIDSLYEGTTAIQAQDFFFRKIVRDKGVALAHVSEQIQKF

VDSESGNGRLKSERELLAKALADVQGMATALTGYLMAAQEDVTSLYKVGLGSVRFLMSVG

DLIIGWLLQRQAAVAVAALDAGASGEDRSFYEGKIAVASFFAKNFLPMLTSTREVIETLD

NDIMELDEAAF

>mavr:LA63_23000 butyryl-CoA dehydrogenase (A)

MSHYKSNVRDQEFNLFEVLGVDKALGQGEFSDLDADTAREMLNEISRLAEGPIADSFVEG

DRNPPVFDPKTHSVTLPESFKKSVHAVIEAGWDKVGIDEALGGVAMPKALLWALHEHILG

ANPAVWMYAGGAGFANILYHLGTDEQKKWAVMAAERGWGSTMVLTEPDAGSDVGAGRTKA

VKQDDGSWHIDGVKRFITSADSDDLFENIFHLVLARPEGAGPGTKGLSLFFVPKFLFDFE

TGELGERNGVFVTNVEHKMGLKVSATCELSFGQHDVPAKGWLVGEVHNGIAQMFEVIEQA

RMMVGTKAIATLSTGYLNALEYAKSRVQGADMTQMTDKTAPRVTITHHPDVRRSLMTQKA

YAEGLRALYLFTSTYQDAAVAEALYGVDAELAVKVNDLMLPVVKGVGSEQAYAKLTESLQ

TFGGSGFLQDYPIEQYIRDSKIDSLYEGTTAIQAQDFFFRKIVRDKGVALAHVSEQIQKF

VDSESGNGRLKSERELLAKALADVQGMATALTGYLMAAQEDVTSLYKVGLGSVRFLMSVG

DLIIGWLLQRQAAVAVAALDAGASGEDRSFYEGKIAVASFFAKNFLPMLTSTREVIETLD

NDIMELDEAAF

>mavd:NF84_22735 butyryl-CoA dehydrogenase (A)

MSHYKSNVRDQEFNLFEVLGVDKALGQGEFSDLDADTAREMLNEISRLAEGPIADSFVEG

DRNPPVFDPKTHSVTLPESFKKSVHAVIEAGWDKVGIDEALGGVAMPKALLWALHEHILG

ANPAVWMYAGGAGFANILYHLGTDEQKKWAVMAAERGWGSTMVLTEPDAGSDVGAGRTKA

VKQDDGSWHIDGVKRFITSADSDDLFENIFHLVLARPEGAGPGTKGLSLFFVPKFLFDFE

TGELGERNGVFVTNVEHKMGLKVSATCELSFGQHDVPAKGWLVGEVHNGIAQMFEVIEQA

RMMVGTKAIATLSTGYLNALEYAKSRVQGADMTQMTDKTAPRVTITHHPDVRRSLMTQKA

YAEGLRALYLFTSTYQDAAVAEALYGVDAELAVKVNDLMLPVVKGVGSEQAYAKLTESLQ

TFGGSGFLQDYPIEQYIRDSKIDSLYEGTTAIQAQDFFFRKIVRDKGVALAHVSEQIQKF

VDSESGNGRLKSERELLAKALADVQGMATALTGYLMAAQEDVTSLYKVGLGSVRFLMSVG

DLIIGWLLQRQAAVAVAALDAGASGEDRSFYEGKIAVASFFAKNFLPMLTSTREVIETLD

NDIMELDEAAF

>mava:LA64_22935 butyryl-CoA dehydrogenase (A)

MSHYKSNVRDQEFNLFEVLGVDKALGQGEFSDLDADTAREMLNEISRLAEGPIADSFVEG

DRNPPVFDPKTHSVTLPESFKKSVHAVIEAGWDKVGIDEALGGVAMPKALLWALHEHILG

ANPAVWMYAGGAGFANILYHLGTDEQKKWAVMAAERGWGSTMVLTEPDAGSDVGAGRTKA

VKQDDGSWHIDGVKRFITSADSDDLFENIFHLVLARPEGAGPGTKGLSLFFVPKFLFDFE

TGELGERNGVFVTNVEHKMGLKVSATCELSFGQHDVPAKGWLVGEVHNGIAQMFEVIEQA

RMMVGTKAIATLSTGYLNALEYAKSRVQGADMTQMTDKTAPRVTITHHPDVRRSLMTQKA

YAEGLRALYLFTSTYQDAAVAEALYGVDAELAVKVNDLMLPVVKGVGSEQAYAKLTESLQ

TFGGSGFLQDYPIEQYIRDSKIDSLYEGTTAIQAQDFFFRKIVRDKGVALAHVSEQIQKF

VDSESGNGRLKSERELLAKALADVQGMATALTGYLMAAQEDVTSLYKVGLGSVRFLMSVG

DLIIGWLLQRQAAVAVAALDAGASGEDRSFYEGKIAVASFFAKNFLPMLTSTREVIETLD

NDIMELDEAAF

>mit:OCO_48220 putative acyl-CoA dehydrogenase (A)

MSHYKSNVRDQAFNLFEVLGVDKALGQGEYSDLDVDTANEMLNEMSRLAEGPIADSFVEG

DRNPPVFDPKTHSVTLPESFKKSVHAVIEAGWDKVGIDEALGGVAMPKSLLWALHEHILG

ANPAVWMYAGGAGFANILYHLGTEEQKKWAVLAAERGWGSTMVLTEPDAGSDVGAGRTKA

VKQDDGSWHIDGVKRFITSADSGDLFENIFHLVLARPEGAGPGTKGLSLFFVPKFLFDFE

TGELGERNGVFVTNVEHKMGLKVSATCELSFGQHDVPAKGWLVGEVHNGIAQMFDVIEQA

RMMVGTKAIATLSTGYLNALEYAKSRVQGADMTQMTDKTAPRVTITHHPDVRRSLMTQKA

YAEGLRALYLFTSTYQDAAVAEALHGVDAELAVKVNDLMLPVVKGVGSEQAYAKLTESLQ

TFGGSGFLQDYPIEQYIRDAKIDSLYEGTTAIQAQDFFFRKIVRDKGVALAHVSEQIQKF

VDSESGNGRLKTEREYLAKALTDVQAMATSLTGYLMAAQEDVTSLYKVGLGSVRFLMSVG

DLVIGWLLQRQAAVAVAALDAGASGDDKAFYEGKVAVASFFAKNFLPMLTSTREVIETLD

NDIMELDEAAF

>mir:OCQ_49260 putative acyl-CoA dehydrogenase (A)

MSHYKSNVRDQAFNLFEVLGVDKALGQGEYSDLDVDTANEMLNEMSRLAEGPIADSFVEG

DRNPPVFDPKTHSVTLPESFKKSVHAVIEAGWDKVGIDEALGGVAMPKSLLWALHEHILG

ANPAVWMYAGGAGFANILYHLGTEEQKKWAVLAAERGWGSTMVLTEPDAGSDVGAGRTKA

VKQDDGSWHIDGVKRFITSADSGDLFENIFHLVLARPEGAGPGTKGLSLFFVPKFLFDFE

TGELGERNGVFVTNVEHKMGLKVSATCELSFGQHDVPAKGWLVGEVHNGIAQMFDVIEQA

RMMVGTKAIATLSTGYLNALEYAKSRVQGADMTQMTDKTAPRVTITHHPDVRRSLMTQKA

YAEGLRALYLFTSTYQDASVAEALHGVDAELAVKVNDLMLPVVKGVGSEQAYAKLTESLQ

TFGGSGFLQDYPIEQYIRDAKIDSLYEGTTAIQAQDFFFRKIVRDKGVALAHVSEQIQKF

VDSESGNGRLKTEREYLAKALTDVQAMATSLTGYLMAAQEDVTSLYKVGLGSVRFLMSVG

DLVIGWLLQRQAAVAVAALDAGASGDDKAFYEGKVAVASFFAKNFLPMLTSTREVIETLD

NDIMELDEAAF

>mia:OCU_48180 putative acyl-CoA dehydrogenase (A)

MSHYKSNVRDQAFNLFEVLGVDKALGQGEYSDLDVDTANEMLNEMSRLAEGPIADSFVEG

DRNPPVFDPKTHSVTLPESFKKSVHAVIEAGWDKVGIDEALGGVAMPKSLLWALHEHILG

ANPAVWMYAGGAGFANILYHLGTEEQKKWAVLAAERGWGSTMVLTEPDAGSDVGAGRTKA

VKQDDGSWHIDGVKRFITSADSGDLFENIFHLVLARPEGAGPGTKGLSLFFVPKFLFDFE

TGELGERNGVFVTNVEHKMGLKVSATCELSFGQHDVPAKGWLVGEVHNGIAQMFDVIEQA

RMMVGTKAIATLSTGYLNALEYAKSRVQGADMTQMTDKTAPRVTITHHPDVRRSLMTQKA

YAEGLRALYLFTSTYQDAAVAEALHGVDAELAVKVNDLMLPVVKGVGSEQAYAKLTESLQ

TFGGSGFLQDYPIEQYIRDAKIDSLYEGTTAIQAQDFFFRKIVRDKGVALAHVSEQIQKF

VDSESGNGRLKTEREYLAKALTDVQAMATSLTGYLMAAQEDVTSLYKVGLGSVRFLMSVG

DLVIGWLLQRQAAVAVAALDAGASGDDKAFYEGKVAVASFFAKNFLPMLTSTREVIETLD

NDIMELDEAAF

>mie:LG41_22850 butyryl-CoA dehydrogenase (A)

MSHYKSNVRDQAFNLFEVLGVDKALGQGEYSDLDVDTANEMLNEMSRLAEGPIADSFVEG

DRNPPVFDPKTHSVTLPESFKKSVHAVIEAGWDKVGIDEALGGVAMPKSLLWALHEHILG

ANPAVWMYAGGAGFANILYHLGTEEQKKWAVLAAERGWGSTMVLTEPDAGSDVGAGRTKA

VKQDDGSWHIDGVKRFITSADSGDLFENIFHLVLARPEGAGPGTKGLSLFFVPKFLFDFE

TGELGERNGVFVTNVEHKMGLKVSATCELSFGQHDVPAKGWLVGEVHNGIAQMFDVIEQA

RMMVGTKAIATLSTGYLNALEYAKSRVQGADMTQMTDKTAPRVTITHHPDVRRSLMTQKA

YAEGLRALYLFTSTYQDAAVAEALHGVDAELAVKVNDLMLPVVKGVGSEQAYAKLTESLQ

TFGGSGFLQDYPIEQYIRDAKIDSLYEGTTAIQAQDFFFRKIVRDKGVALAHVSEQIQKF

VDSESGNGRLKTEREYLAKALTDVQAMATSLTGYLMAAQEDVTSLYKVGLGSVRFLMSVG

DLVIGWLLQRQAAVAVAALDAGASGDDKAFYEGKVAVASFFAKNFLPMLTSTREVIETLD

NDIMELDEAAF

>mid:MIP_07295 Acyl-CoA dehydrogenase, short-chain specific (A)

MSHYKSNVRDQAFNLFEVLGVDKALGQGEYSDLDVDTANEMLNEMSRLAEGPIADSFVEG

DRNPPVFDPKTHSVTLPESFKKSVHAVIEAGWDKVGIDEALGGVAMPKSLLWALHEHILG

ANPAVWMYAGGAGFANILYHLGTEEQKKWAVLAAERGWGSTMVLTEPDAGSDVGAGRTKA

VKQDDGSWHIDGVKRFITSADSGDLFENIFHLVLARPEGAGPGTKGLSLFFVPKFLFDFE

TGELGERNGVFVTNVEHKMGLKVSATCELSFGQHDVPAKGWLVGEVHNGIAQMFDVIEQA

RMMVGTKAIATLSTGYLNALEYAKSRVQGADMTQMTDKTAPRVTITHHPDVRRSLMTQKA

YAEGLRALYLFTSTYQDASVAEALHGVDAELAVKVNDLMLPVVKGVGSEQAYAKLTESLQ

TFGGSGFLQDYPIEQYIRDAKIDSLYEGTTAIQAQDFFFRKIVRDKGVALAHVSEQIQKF

VDSESGNGRLKTEREYLAKALTDVQAMATSLTGYLMAAQEDVTSLYKVGLGSVRFLMSVG

DLVIGWLLQRQAAVAVAALDAGASGDDKAFYEGKVAVASFFAKNFLPMLTSTREVIETLD

NDIMELDEAAF

>myo:OEM_48390 putative acyl-CoA dehydrogenase (A)

MSHYKSNVRDQAFNLFEVLGVDKALGQGEYSDLDVDTANEMLNEMSRLAEGPIADSFVEG

DRNPPVFDPKTHSVTLPESFKKSVHAVIEAGWDKVGIDEALGGVAMPKSLLWALHEHILG

ANPAVWMYAGGAGFANILYHLGTEEQKKWAVLAAERGWGSTMVLTEPDAGSDVGAGRTKA

VKQDDGSWHIDGVKRFITSADSGDLFENIFHLVLARPEGAGPGTKGLSLFFVPKFLFDFE

TGELGERNGVFVTNVEHKMGLKVSATCELSFGQHDVPAKGWLVGEVHNGIAQMFDVIEQA

RMMVGTKAIATLSTGYLNALEYAKSRVQGADMTQMTDKTAPRVTITHHPDVRRSLMTQKA

YAEGLRALYLFTSTYQDAAVAEALHGVDAELAVKVNDLMLPVVKGVGSEQAYAKLTESLQ

TFGGSGFLQDYPIEQYIRDAKIDSLYEGTTAIQAQDFFFRKIVRDKGVALAHVSEQIQKF

VDSESGNGRLKTEREYLAKALTDVQAMATSLTGYLMAAQEDVTSLYKVGLGSVRFLMSVG

DLVIGWLLQRQAAVAVAALDAGASGDDKAFYEGKVAVASFFAKNFLPMLTSTREVIETLD

NDIMELDEAAF

>msm:MSMEG_0406 acyl-CoA dehydrogenase (A)

MSHYKSNVRDQVFNLFEVFGVDKVLGADKFSDLDADTAREMLTEIARLAEGPIAESFVEG

DRNPPVFDPETHTVTLPEGFKKSMRALFDGGWDKVGLAEHLGGIPMPRALQWALIEHILG

ANPAAYMYAMGPGMSEIFYNNGTDEQKKWATIAAERGWGATMVLTEPDAGSDVGAGRTKA

VQQPDGTWHIEGVKRFITSADSDDLFENIMHLVLARPEGAGPGTKGLSLFFVPKFHFDHE

TGEIGERNGVFVTNVEHKMGLKVSATCELSLGQHGIPAVGWLVGEVHNGIAQMFDVIEQA

RMMVGTKAIATLSTGYLNALEYAKERVQGADMTQMTDKTAPRVTITHHPDVRRSLMTQKA

YAEGLRAIYLYTATFQDAEVAQAVHGVDGDLAARVNDLLLPIVKGFGSETAYAKLTESLQ

TLGGSGFLQDYPIEQYIRDSKIDSLYEGTTAIQAQDFFFRKIIRDKGQALAYVAGEIEQF

IKNENGNGRLKTERELLATALADVQGMAASLTGYLMAAQEDAASIYKVGLGSVRFLMAVG

DLLSGWLLARQAAVAIEKLDAGATGADKSFYEGKIAAASFFAKNMLPLLTSTRQIIENLD

NDVMELDEAAF

>msg:MSMEI_0396 fadE5; Acyl-CoA dehydrogenase (EC:1.3.8.1) (A)

MSHYKSNVRDQVFNLFEVFGVDKVLGADKFSDLDADTAREMLTEIARLAEGPIAESFVEG

DRNPPVFDPETHTVTLPEGFKKSMRALFDGGWDKVGLAEHLGGIPMPRALQWALIEHILG

ANPAAYMYAMGPGMSEIFYNNGTDEQKKWATIAAERGWGATMVLTEPDAGSDVGAGRTKA

VQQPDGTWHIEGVKRFITSADSDDLFENIMHLVLARPEGAGPGTKGLSLFFVPKFHFDHE

TGEIGERNGVFVTNVEHKMGLKVSATCELSLGQHGIPAVGWLVGEVHNGIAQMFDVIEQA

RMMVGTKAIATLSTGYLNALEYAKERVQGADMTQMTDKTAPRVTITHHPDVRRSLMTQKA

YAEGLRAIYLYTATFQDAEVAQAVHGVDGDLAARVNDLLLPIVKGFGSETAYAKLTESLQ

TLGGSGFLQDYPIEQYIRDSKIDSLYEGTTAIQAQDFFFRKIIRDKGQALAYVAGEIEQF

IKNENGNGRLKTERELLATALADVQGMAASLTGYLMAAQEDAASIYKVGLGSVRFLMAVG

DLLSGWLLARQAAVAIEKLDAGATGADKSFYEGKIAAASFFAKNMLPLLTSTRQIIENLD

NDVMELDEAAF

>msb:LJ00_02020 butyryl-CoA dehydrogenase (A)

MSHYKSNVRDQVFNLFEVFGVDKVLGADKFSDLDADTAREMLTEIARLAEGPIAESFVEG

DRNPPVFDPETHTVTLPEGFKKSMRALFDGGWDKVGLAEHLGGIPMPRALQWALIEHILG

ANPAAYMYAMGPGMSEIFYNNGTDEQKKWATIAAERGWGATMVLTEPDAGSDVGAGRTKA

VQQPDGTWHIEGVKRFITSADSDDLFENIMHLVLARPEGAGPGTKGLSLFFVPKFHFDHE

TGEIGERNGVFVTNVEHKMGLKVSATCELSLGQHGIPAVGWLVGEVHNGIAQMFDVIEQA

RMMVGTKAIATLSTGYLNALEYAKERVQGADMTQMTDKTAPRVTITHHPDVRRSLMTQKA

YAEGLRAIYLYTATFQDAEVAQAVHGVDGDLAARVNDLLLPIVKGFGSETAYAKLTESLQ

TLGGSGFLQDYPIEQYIRDSKIDSLYEGTTAIQAQDFFFRKIIRDKGQALAYVAGEIEQF

IKNENGNGRLKTERELLATALADVQGMAASLTGYLMAAQEDAASIYKVGLGSVRFLMAVG

DLLSGWLLARQAAVAIEKLDAGATGADKSFYEGKIAAASFFAKNMLPLLTSTRQIIENLD

NDVMELDEAAF

>msn:LI99_02020 butyryl-CoA dehydrogenase (A)

MSHYKSNVRDQVFNLFEVFGVDKVLGADKFSDLDADTAREMLTEIARLAEGPIAESFVEG

DRNPPVFDPETHTVTLPEGFKKSMRALFDGGWDKVGLAEHLGGIPMPRALQWALIEHILG

ANPAAYMYAMGPGMSEIFYNNGTDEQKKWATIAAERGWGATMVLTEPDAGSDVGAGRTKA

VQQPDGTWHIEGVKRFITSADSDDLFENIMHLVLARPEGAGPGTKGLSLFFVPKFHFDHE

TGEIGERNGVFVTNVEHKMGLKVSATCELSLGQHGIPAVGWLVGEVHNGIAQMFDVIEQA

RMMVGTKAIATLSTGYLNALEYAKERVQGADMTQMTDKTAPRVTITHHPDVRRSLMTQKA

YAEGLRAIYLYTATFQDAEVAQAVHGVDGDLAARVNDLLLPIVKGFGSETAYAKLTESLQ

TLGGSGFLQDYPIEQYIRDSKIDSLYEGTTAIQAQDFFFRKIIRDKGQALAYVAGEIEQF

IKNENGNGRLKTERELLATALADVQGMAASLTGYLMAAQEDAASIYKVGLGSVRFLMAVG

DLLSGWLLARQAAVAIEKLDAGATGADKSFYEGKIAAASFFAKNMLPLLTSTRQIIENLD

NDVMELDEAAF

>msh:LI98_02020 butyryl-CoA dehydrogenase (A)

MSHYKSNVRDQVFNLFEVFGVDKVLGADKFSDLDADTAREMLTEIARLAEGPIAESFVEG

DRNPPVFDPETHTVTLPEGFKKSMRALFDGGWDKVGLAEHLGGIPMPRALQWALIEHILG

ANPAAYMYAMGPGMSEIFYNNGTDEQKKWATIAAERGWGATMVLTEPDAGSDVGAGRTKA

VQQPDGTWHIEGVKRFITSADSDDLFENIMHLVLARPEGAGPGTKGLSLFFVPKFHFDHE

TGEIGERNGVFVTNVEHKMGLKVSATCELSLGQHGIPAVGWLVGEVHNGIAQMFDVIEQA

RMMVGTKAIATLSTGYLNALEYAKERVQGADMTQMTDKTAPRVTITHHPDVRRSLMTQKA

YAEGLRAIYLYTATFQDAEVAQAVHGVDGDLAARVNDLLLPIVKGFGSETAYAKLTESLQ

TLGGSGFLQDYPIEQYIRDSKIDSLYEGTTAIQAQDFFFRKIIRDKGQALAYVAGEIEQF

IKNENGNGRLKTERELLATALADVQGMAASLTGYLMAAQEDAASIYKVGLGSVRFLMAVG

DLLSGWLLARQAAVAIEKLDAGATGADKSFYEGKIAAASFFAKNMLPLLTSTRQIIENLD

NDVMELDEAAF

>msa:Mycsm_00204 acyl-CoA dehydrogenase (A)

MSHYKSNVRDQVFNLFEVFGVDKALGEGAYTDLDTDTATEMLGEMARLAEGPIAESFVDS

DRNPPVFDPNTHEVKLPEPFKKSMRALLDGGWDKIGLVEELGGMPMPRALQWALIEHVLG

ANPAAYMYAMGAGMAQILWNLGTDEQKKWAVLAAERGWTATMVLTEPDAGSDVGAGRTKA

VQQPDGTWHIDGVKRFITSGDADDIGENIMHLVLARPEGAGPGTKGLSLFFVPKFHFDPE

TGEPGERNGVFVTNVEHKMGLKVSATCELSLGQHGVPAVGWLVGEVHDGIAQMFDVIEQA

RMMVGTKAIATLSTGYLNALEYAKERVQGADLTQMTDKTAPRVTITHHPDVRRSLMTQKA

YAEGLRALYLYTATFQDSAVAKALHDVDAELAVRVNDLMLPLVKGVGSEQAYAKLTESLQ

TFGGSGFLQDYPIEQYIRDAKIDSLYEGTTAIQAQDFFFRKLVRDKGQALAYVAGQVEQF

VKNEAGNGRLKAERALLATALEDVQGMAATLTGYLMGAQENPAELYKVGLGSVRFLMSVG

DLVIGWLLQQQAAVAIEALDAGASGDDRAFYEGKIAVASFFAKNMLPLLTSTRAVIDTLD

NEIMELDEAAF

>mul:MUL_1168 fadE5; acyl-CoA dehydrogenase FadE5 (A)

MWHSSTRRTVVSHYKSNVRDQVFNLFEVLGVDKALGEGQFSDLDTDTAREMLTEISRLAE

GPVAASFIEGDRNPPVFDPKTHTVTLPESFKDSVRAVIEAGWDKAGLDEELGGMPMPRAL

MGALHEHLLGANPAVWMYSGGAGFAQIMYHLGTEEQKKWAVMASERGWGSTMVLTEPDAG

SDVGAGRTKAVEQADGSWHIDGVKRFITSGDSDDLFENIFHLVLARPEGAGPGTKGLSLF

FVPKFLFDFETGELGERNGVFVTNVEHKMGLKVSATCELSLGQHGVPAKGWLIGEVHEGI

AQMFEVIEQARMMVGTKAIATLSTGYLNALEYAKSRVQGADLTQMTDKTAPRVTITHHPD

VRRSLMTQKAYAEGLRALYLYTATFQDAAVAEAAHGVDAKLAVKINDLLLPVVKGVGSEQ

AYANLTESLQTLGGSGFLQDYPIEQYIRDAKIDSLYEGTTAIQAQDFFFRKIVRDKGVAL

AHVSGQIQEFVDSETGNGRLKTERALLAKALTDVQAMAATLTGYLMAAQQDINSLYKVGL

GSVRFLMSVGDLVIAWLLQRQAAVAVAALDAGATGDERSFYEGKVAVASFFAKNFLPLLT

STREVIDTLDNDIMELDEAAF

>mva:Mvan_0265 acyl-CoA dehydrogenase domain protein (A)

MSHYKSNVRDQVFNLFEVLGVDKALGQGSYSDLDADTAVEMLGEMARLAEGPVAASFEDG

DRNPPVFDPKAHTVTLPESFKKSVRAVIDGGWDKIGVDEELGGTPMPRALFWAIQEHILG

ANPAVYMYAMGAGFADIFYHLGTDEQKKWAKLAADRGWGSTMVLTEPDAGSDVGAGRTKA

VQQPDGTWHIDGVKRFITSADSDDLFENIMHLVLARPEGAGPGTKGLSLFFVPKFHFDPE

TGEPGERNGVFVTNVEHKMGLKVSATCELSLGQHGVPAVGWLVGEVHEGIAQMFDVIEQA

RMMVGTKAIATLSTGYLNALEYAKERVQGADLTQMTDKTAPRVTITHHPDVRRSLMTQKA

YAEGLRALYLYTATHQDAEVAKALYGIEPELALKVNDLMLPIVKGVGSEQAYAKLTESLQ

TLGGSGFLQDYPIEQYIRDAKIDSLYEGTTAIQAQDFFFRKIVRDKGVALAHVAGQIEQF

VKAETGNGRLKAERALLATALEDVQGMAASLTGYLMASQEDAKSIYKVGLGSVRFLMSVG

DLVLGWLLQQQAAVAIEKLDAGAQGDDRAFYEGKVAVASFFAKNFLPLLTSTRSIVENLD

NEVMELDEAAF

>mgi:Mflv_0409 acyl-CoA dehydrogenase domain protein (A)

MGHYKSNVRDQVFNLFEVLGVDKALGQGAYAELDVDTVTEMLGEMAKLAEGPVADSFAEG

DRNPPVFDPKTHSVTLPEAFKKSVRAVTDGGWDKLSASEEIGGAALPSVISWALQEHILG

ANPAVYMYAMGAGFADIFYHLGTDEQKKWAKLAADRGWGSTMVLTEPDAGSDVGAGRTKA

VQQPDGTWHIDGVKRFITSADSDDLFENIMHLVLARPEGAGPGTKGLSLFFVPKFHFDHE

TGEPGERNGVFVTNVEHKMGLKISTTCELSLGQHGTPAVGWLVGEVHDGIAQMFDVIEQA

RMMVGTKAIATLSTGYLNALAYAKDRVQGADLTQMTDKTAPRVTITHHPDVRRSLMTQKA

YAEGLRALYLFTATHQNAEMAKVVHGIEPDLAVRVNDLMLPIVKGVGSEQAYAKLTESLQ

TFGGSGFLQDYPIEQYIRDAKIDSLYEGTTAIQAQDFFFRKIVRDKGVALAHVAGQIEQF

VKAETGNGRLKAERALLATALEDVQGMAASLTGYLMASQEDQAAIYKVGLGSVRFLMSVG

DLVIGWLLQQQAAVAIEKLDAGVEGADRAFYEGKIAVASFFAKNFLPLLTSTRSIIENLD

NEVMELDEASF

>msp:Mspyr1_03460 acyl-CoA dehydrogenase (A)

MGHYKSNVRDQVFNLFEVLGVDKALGQGAYAELDVDTVTEMLGEMAKLAEGPVADSFAEG

DRNPPVFDPKTHSVTLPEAFKKSVRAVTDGGWDKLSASEEIGGAALPSVISWALQEHILG

ANPAVYMYAMGAGFADIFYHLGTDEQKKWAKLAADRGWGSTMVLTEPDAGSDVGAGRTKA

VQQPDGTWHIDGVKRFITSADSDDLFENIMHLVLARPEGAGPGTKGLSLFFVPKFHFDHE

TGEPGERNGVFVTNVEHKMGLKISTTCELSLGQHGTPAVGWLVGEVHDGIAQMFDVIEQA

RMMVGTKAIATLSTGYLNALAYAKDRVQGADLTQMTDKTAPRVTITHHPDVRRSLMTQKA

YAEGLRALYLFTATHQNAEMAKVVHGIEPDLAVRVNDLMLPIVKGVGSEQAYAKLTESLQ

TFGGSGFLQDYPIEQYIRDAKIDSLYEGTTAIQAQDFFFRKIVRDKGVALAHVAGQIEQF

VKAETGNGRLKAERALLATALEDVQGMAASLTGYLMASQEDQAAIYKVGLGSVRFLMSVG

DLVIGWLLQQQAAVAIEKLDAGVEGADRAFYEGKIAVASFFAKNFLPLLTSTRSIIENLD

NEVMELDEASF

>mab:MAB_4437 Probable acyl-CoA dehydrogenase FadE (A)

MTHMGHYKSNVRDLEFNLFELFKIQQVFGGEEFPELDEDTARTFLAEMRTLAEGPLADSF

AEGDRNPPVFDPETHSVAIPEAFKKSVKAITEAGWDRVGLQEELGGTPVPRSLSWAIQEM

ILGANPAVWMYSGGAGFAQIFYNIATDEQKKWAEFVAERGWGATMVLTEPDAGSDVGAGR

TKAVKQDDGSWHIDGVKRFITSADSDDMFENIMHLVLARPEGAGPGTKGLSLFFVPKFIP

NFETGEPGERNGVFVTNVEHKMGLKVSATCELSFGQHGVPAKGWLVGEVHNGIAQMFDVI

EQARMMVGTKAIATLSTGYLNALEYAKTRVQGADMTQLTDKTAPRVTITHHPDVRRSLMT

QKVYAEGLRALYLYATTFQDAVAAKTINGVEAEDAVKLNDLLLPVIKGVGSERAYEKLTE

SLQTFGGSGFLQDYPIEQYIRDAKIDSLYEGTTAIQAQDFFFRKIVKDQGKSLAFISGQI

EEFVKSETGNGRLKAERALLATALEDVQGMAASMTGNLMAAQQEITQVYRVGTASVRFLM

SVGDLLIGWLLQRQAAVAIEALDAGATGADKSFYEGKIAAASFFAKNMLPLLTSTRAVLE

NVDNDIMELDEAAF

>mabb:MASS_4474 acyl-CoA dehydrogenase FadE (A)

MTHMGHYKSNVRDLEFNLFELFKIQQVFGGEEFPELDEDTARTFLAEMRTLAEGPLADSF

AEGDRNPPVFDPETHSVAIPEAFKKSVKAITEAGWDRVGLQEELGGTPVPRSLSWAIQEM

ILGANPAVWMYSGGAGFAQIFYNIATDEQKKWAEFVAERGWGATMVLTEPDAGSDVGAGR

TKAVKQDDGSWHIDGVKRFITSADSDDMFENIMHLVLARPEGAGPGTKGLSLFFVPKFIP

NFETGEPGERNGVFVTNVEHKMGLKVSATCELSFGQHGVPAKGWLVGEVHNGIAQMFDVI

EQARMMVGTKAIATLSTGYLNALEYAKTRVQGADMTQLTDKTAPRVTITHHPDVRRSLMT

QKVYAEGLRALYLYATTFQDAVAAKTINGVEAEDAVKLNDLLLPVIKGVGSERAYEKLTE

SLQTFGGSGFLQDYPIEQYIRDAKIDSLYEGTTAIQAQDFFFRKIVKDQGKSLAFVSGQI

EEFVKSETGNGRLKAERALLATALEDVQGMAASMTANLMAAQQEITQVYKVGTASVRFLM

SVGDLLIGWLLQRQAAVAIEALDAGATGADKSFYEGKIAAASFFAKNMLPLLTSTRAVLE

NVDNDIMELDEAAF

>mmv:MYCMA_2466 butyryl-CoA dehydrogenase (A)

MGHYKSNVRDLEFNLFELFKIQQVFGGEEFPELDEDTARTFLAEMRTLAEGPLADSFAEG

DRNPPVFDPETHSVAIPEAFKKSVKAITEAGWDRVGLQEELGGTPVPRSLSWAIQEMILG

ANPAVWMYSGGAGFAQIFYNIATDEQKKWAEFVAERGWGATMVLTEPDAGSDVGAGRTKA

VKQDDGSWHIDGVKRFITSADSDDMFENIMHLVLARPEGAGPGTKGLSLFFVPKFIPNFE

TGEPGERNGVFVTNVEHKMGLKVSATCELSFGQHGVPAKGWLVGEVHNGIAQMFDVIEQA

RMMVGTKAIATLSTGYLNALEYAKTRVQGADMTQLTDKTAPRVTITHHPDVRRSLMTQKV

YAEGLRALYLYATTFQDAVAAKTINGVEAEDAVKLNDLLLPVIKGVGSERAYEKLTESLQ

TFGGSGFLQDYPIEQYIRDAKIDSLYEGTTAIQAQDFFFRKIVKDQGKSLAFVSGQIEEF

VKSETGNGRLKAERALLATALEDVQGMAASMTANLMAAQQEITQVYKVGTASVRFLMSVG

DLLIGWLLQRQAAVAIEALDAGATGADKSFYEGKIAAASFFAKNMLPLLTSTRAVLENVD

NDIMELDEAAF

>may:LA62_22525 butyryl-CoA dehydrogenase (A)

MGHYKSNVRDLEFNLFELFKIQQVFGGEEFPELDEDTARTFLAEMRTLAEGPLADSFAEG

DRNPPVFDPETHSVAIPEAFKKSVKAITEAGWDRVGLQEELGGTPVPRSLSWAIQEMILG

ANPAVWMYSGGAGFAQIFYNIATDEQKKWAEFVAERGWGATMVLTEPDAGSDVGAGRTKA

VKQDDGSWHIDGVKRFITSADSDDMFENIMHLVLARPEGAGPGTKGLSLFFVPKFIPNFE

TGEPGERNGVFVTNVEHKMGLKVSATCELSFGQHGVPAKGWLVGEVHNGIAQMFDVIEQA

RMMVGTKAIATLSTGYLNALEYAKTRVQGADMTQLTDKTAPRVTITHHPDVRRSLMTQKV

YAEGLRALYLYATTFQDAVAAKTINGVEAEDAVKLNDLLLPVIKGVGSERAYEKLTESLQ

TFGGSGFLQDYPIEQYIRDAKIDSLYEGTTAIQAQDFFFRKIVKDQGKSLAFISGQIEEF

VKSETGNGRLKAERALLATALEDVQGMAASMTGNLMAAQQEITQVYRVGTASVRFLMSVG

DLLIGWLLQRQAAVAIEALDAGATGADKSFYEGKIAAASFFAKNMLPLLTSTRAVLENVD

NDIMELDEAAF

>mabo:NF82_22215 butyryl-CoA dehydrogenase (A)

MGHYKSNVRDLEFNLFELFKIQQVFGGEEFPELDEDTARTFLAEMRTLAEGPLADSFAEG

DRNPPVFDPETHSVAIPEAFKKSVKAITEAGWDRVGLQEELGGTPVPRSLSWAIQEMILG

ANPAVWMYSGGAGFAQIFYNIATDEQKKWAEFVAERGWGATMVLTEPDAGSDVGAGRTKA

VKQDDGSWHIDGVKRFITSADSDDMFENIMHLVLARPEGAGPGTKGLSLFFVPKFIPNFE

TGEPGERNGVFVTNVEHKMGLKVSATCELSFGQHGVPAKGWLVGEVHNGIAQMFDVIEQA

RMMVGTKAIATLSTGYLNALEYAKTRVQGADMTQLTDKTAPRVTITHHPDVRRSLMTQKV

YAEGLRALYLYATTFQDAVAAKTINGVEAEDAVKLNDLLLPVIKGVGSERAYEKLTESLQ

TFGGSGFLQDYPIEQYIRDAKIDSLYEGTTAIQAQDFFFRKIVKDQGKSLAFISGQIEEF

VKSETGNGRLKAERALLATALEDVQGMAASMTGNLMAAQQEITQVYRVGTASVRFLMSVG

DLLIGWLLQRQAAVAIEALDAGATGADKSFYEGKIAAASFFAKNMLPLLTSTRAVLENVD

NDIMELDEAAF

>mabl:MMASJCM_4499 3-methylmercaptopropionyl-CoA dehydrogenase (A)

MGHYKSNVRDLEFNLFELFKIQQVFGGEEFPELDEDTARTFLAEMRTLAEGPLADSFAEG

DRNPPVFDPETHSVAIPEAFKKSVKAITEAGWDRVGLQEELGGTPVPRSLSWAIQEMILG

ANPAVWMYSGGAGFAQIFYNIATDEQKKWAEFVAERGWGATMVLTEPDAGSDVGAGRTKA

VKQDDGSWHIDGVKRFITSADSDDMFENIMHLVLARPEGAGPGTKGLSLFFVPKFIPNFE

TGEPGERNGVFVTNVEHKMGLKVSATCELSFGQHGVPAKGWLVGEVHNGIAQMFDVIEQA

RMMVGTKAIATLSTGYLNALEYAKTRVQGADMTQLTDKTAPRVTITHHPDVRRSLMTQKV

YAEGLRALYLYATTFQDAVAAKTINGVEAEDAVKLNDLLLPVIKGVGSERAYEKLTESLQ

TFGGSGFLQDYPIEQYIRDAKIDSLYEGTTAIQAQDFFFRKIVKDQGKSLAFVSGQIEEF

VKSETGNGRLKAERALLATALEDVQGMAASMTANLMAAQQEITQVYKVGTASVRFLMSVG

DLLIGWLLQRQAAVAIEALDAGATGADKSFYEGKIAAASFFAKNMLPLLTSTRAVLENVD

NDIMELDEAAF

>maz:LA61_22420 butyryl-CoA dehydrogenase (A)

MGHYKSNVRDLEFNLFELFKIQQVFGGEEFPELDEDTARTFLAEMRTLAEGPLADSFAEG

DRNPPVFDPETHSVAIPEAFKKSVKAITEAGWDRVGLQEELGGTPVPRSLSWAIQEMILG

ANPAVWMYSGGAGFAQIFYNIATDEQKKWAEFVAERGWGATMVLTEPDAGSDVGAGRTKA

VKQDDGSWHIDGVKRFITSADSDDMFENIMHLVLARPEGAGPGTKGLSLFFVPKFIPNFE

TGEPGERNGVFVTNVEHKMGLKVSATCELSFGQHGVPAKGWLVGEVHNGIAQMFDVIEQA

RMMVGTKAIATLSTGYLNALEYAKTRVQGADMTQLTDKTAPRVTITHHPDVRRSLMTQKV

YAEGLRALYLYATTFQDAVAAKTINGVEAEDAVKLNDLLLPVIKGVGSERAYEKLTESLQ

TFGGSGFLQDYPIEQYIRDAKIDSLYEGTTAIQAQDFFFRKIVKDQGKSLAFISGQIEEF

VKSETGNGRLKAERALLATALEDVQGMAASMTGNLMAAQQEITQVYRVGTASVRFLMSVG

DLLIGWLLQRQAAVAIEALDAGATGADKSFYEGKIAAASFFAKNMLPLLTSTRAVLENVD

NDIMELDEAAF

>mak:LH56_02150 butyryl-CoA dehydrogenase (A)

MGHYKSNVRDLEFNLFELFKIQQVFGGEEFPELDEDTARTFLAEMRTLAEGPLADSFAEG

DRNPPVFDPETHSVAIPEAFKKSVKAITEAGWDRVGLQEELGGTPVPRSLSWAIQEMILG

ANPAVWMYSGGAGFAQIFYNIATDEQKKWAEFVAERGWGATMVLTEPDAGSDVGAGRTKA

VKQDDGSWHIDGVKRFITSADSDDMFENIMHLVLARPEGAGPGTKGLSLFFVPKFIPNFE

TGEPGERNGVFVTNVEHKMGLKVSATCELSFGQHGVPAKGWLVGEVHNGIAQMFDVIEQA

RMMVGTKAIATLSTGYLNALEYAKTRVQGADMTQLTDKTAPRVTITHHPDVRRSLMTQKV

YAEGLRALYLYATTFQDAVAAKTINGVEAEDAVKLNDLLLPVIKGVGSERAYEKLTESLQ

TFGGSGFLQDYPIEQYIRDAKIDSLYEGTTAIQAQDFFFRKIVKDQGKSLAFVSGQIEEF

VKSETGNGRLKAERALLATALEDVQGMAASMTANLMAAQQEITQVYKVGTASVRFLMSVG

DLLIGWLLQRQAAVAIEALDAGATGADKSFYEGKIAAASFFAKNMLPLLTSTRAVLENVD

NDIMELDEAAF

>mys:NF92_02200 butyryl-CoA dehydrogenase (A)

MGHYKSNVRDLEFNLFELFKIQQVFGGEEFPELDEDTARTFLAEMRTLAEGPLADSFAEG

DRNPPVFDPETHSVAIPEAFKKSVKAITEAGWDRVGLQEELGGTPVPRSLSWAIQEMILG

ANPAVWMYSGGAGFAQIFYNIATDEQKKWAEFVAERGWGATMVLTEPDAGSDVGAGRTKA

VKQDDGSWHIDGVKRFITSADSDDMFENIMHLVLARPEGAGPGTKGLSLFFVPKFIPNFE

TGEPGERNGVFVTNVEHKMGLKVSATCELSFGQHGVPAKGWLVGEVHNGIAQMFDVIEQA

RMMVGTKAIATLSTGYLNALEYAKTRVQGADMTQLTDKTAPRVTITHHPDVRRSLMTQKV

YAEGLRALYLYATTFQDAVAAKTINGVEAEDAVKLNDLLLPVIKGVGSERAYEKLTESLQ

TFGGSGFLQDYPIEQYIRDAKIDSLYEGTTAIQAQDFFFRKIVKDQGKSLAFVSGQIEEF

VKSETGNGRLKAERALLATALEDVQGMAASMTANLMAAQQEITQVYKVGTASVRFLMSVG

DLLIGWLLQRQAAVAIEALDAGATGADKSFYEGKIAAASFFAKNMLPLLTSTRAVLENVD

NDIMELDEAAF

>myc:NF90_02200 butyryl-CoA dehydrogenase (A)

MGHYKSNVRDLEFNLFELFKIQQVFGGEEFPELDEDTARTFLAEMRTLAEGPLADSFAEG

DRNPPVFDPETHSVAIPEAFKKSVKAITEAGWDRVGLQEELGGTPVPRSLSWAIQEMILG

ANPAVWMYSGGAGFAQIFYNIATDEQKKWAEFVAERGWGATMVLTEPDAGSDVGAGRTKA

VKQDDGSWHIDGVKRFITSADSDDMFENIMHLVLARPEGAGPGTKGLSLFFVPKFIPNFE

TGEPGERNGVFVTNVEHKMGLKVSATCELSFGQHGVPAKGWLVGEVHNGIAQMFDVIEQA

RMMVGTKAIATLSTGYLNALEYAKTRVQGADMTQLTDKTAPRVTITHHPDVRRSLMTQKV

YAEGLRALYLYATTFQDAVAAKTINGVEAEDAVKLNDLLLPVIKGVGSERAYEKLTESLQ

TFGGSGFLQDYPIEQYIRDAKIDSLYEGTTAIQAQDFFFRKIVKDQGKSLAFVSGQIEEF

VKSETGNGRLKAERALLATALEDVQGMAASMTANLMAAQQEITQVYKVGTASVRFLMSVG

DLLIGWLLQRQAAVAIEALDAGATGADKSFYEGKIAAASFFAKNMLPLLTSTRAVLENVD

NDIMELDEAAF

>mmc:Mmcs_0242 acyl-CoA dehydrogenase-like protein (A)

MSHYKSNVRDQVFNLFEVLGVDKAMGQGDFSELDADTAREMLDEMARLAEGPIAESFFEG

DRNPPVFDPKTHSVKLPEPFKKSMRALFDAGWDKAGMPEELGGMPMPRALQWALIEHVLG

ANPAAYMYAMGGGMAHILHNLGTEEQKKWAVLAAERGWGATMVLTEPDAGSDVGAGRTKA

VKQDDGSWHIEGVKRFITSADSDDLFENIFHLVLARPEGAGPGTKGLSLFFVPKFLVDFE

TGELGERNGAFVTNVEHKMGLKVSATCELSLGQHGVPAKGWLVGEVHNGIAQMFDVIEQA

RMMVGTKAIATLSTGYLNALEYAKSRVQGADMTQMTDKTAPRVTITHHPDVRRSLMTQKA

YAEGLRALYLYTATFQDTAVAKAVHDVDSELAVRVNDLMLPIVKGVGSEQAYAKLTESLQ

TFGGSGFLQDYPIEQYIRDAKIDSLYEGTTAIQAQDFFFRKIVRDKGQALAYVAGQIEQF

VKSESGNGRLKAERALLATALEDVQAMAASLTGYLMAAQENPAELYKVGLGSVRFLMSVG

DLVIGWLLQQQAAVAIGALDGGATGEDKAFYEGKVAVASFFAKNFLPLLTSTRQVVEHLD

NEIMELDEAAF

>mkm:Mkms_0252 acyl-CoA dehydrogenase domain protein (A)

MSHYKSNVRDQVFNLFEVLGVDKAMGQGDFSELDADTAREMLDEMARLAEGPIAESFFEG

DRNPPVFDPKTHSVKLPEPFKKSMRALFDAGWDKAGMPEELGGMPMPRALQWALIEHVLG

ANPAAYMYAMGGGMAHILHNLGTEEQKKWAVLAAERGWGATMVLTEPDAGSDVGAGRTKA

VKQDDGSWHIEGVKRFITSADSDDLFENIFHLVLARPEGAGPGTKGLSLFFVPKFLVDFE

TGELGERNGAFVTNVEHKMGLKVSATCELSLGQHGVPAKGWLVGEVHNGIAQMFDVIEQA

RMMVGTKAIATLSTGYLNALEYAKSRVQGADMTQMTDKTAPRVTITHHPDVRRSLMTQKA

YAEGLRALYLYTATFQDTAVAKAVHDVDSELAVRVNDLMLPIVKGVGSEQAYAKLTESLQ

TFGGSGFLQDYPIEQYIRDAKIDSLYEGTTAIQAQDFFFRKIVRDKGQALAYVAGQIEQF

VKSESGNGRLKAERALLATALEDVQAMAASLTGYLMAAQENPAELYKVGLGSVRFLMSVG

DLVIGWLLQQQAAVAIGALDGGATGEDKAFYEGKVAVASFFAKNFLPLLTSTRQVVEHLD

NEIMELDEAAF

>mjl:Mjls_0232 acyl-CoA dehydrogenase domain protein (A)

MSHYKSNVRDQVFNLFEVLGVDKAMGQGDFSELDADTAREMLDEMARLAEGPIAESFFEG

DRNPPVFDPKTHSVKLPEPFKKSMRALFDAGWDKAGMPEELGGMPMPRALQWALIEHVLG

ANPAAYMYAMGGGMAHILHNLGTEEQKKWAVLAAERGWGATMVLTEPDAGSDVGAGRTKA

VKQDDGSWHIDGVKRFITSADSDDLFENIFHLVLARPEGAGPGTKGLSLFFVPKFLVDFE

TGELGERNGAFVTNVEHKMGLKVSATCELSLGQHGVPAKGWLVGEVHNGIAQMFDVIEQA

RMMVGTKAIATLSTGYLNALEYAKSRVQGADMTQMTDKTAPRVTITHHPDVRRSLMTQKS

YAEGLRALYLYTATFQDTAVAKAVHDVDSELAVRVNDLMLPIVKGVGSEQAYAKLTESLQ

TFGGSGFLQDYPIEQYIRDAKIDSLYEGTTAIQAQDFFFRKIVRDKGQALAYVAGQIEQF

VKSESGNGRLKAERALLATALEDVQAMAASLTGYLMAAQENPAELYKVGLGSVRFLMSVG

DLVIGWLLQQQAAVAIGALDGGATGEDKAFYEGKVAVASFFAKNFLPLLTSTRQVVEHLD

NEIMELDEAAF

>mjd:JDM601_0229 fadE5; acyl-CoA dehydrogenase FadE5 (A)

MSHYKSNVRDQVFNLFEVFGLDQVFGQGEYTDLDVDTATEMLGEMVRLAEGPIADSFVDG

DRNPPVFDPATHTVTLPESFKKSVRATLEAGWDRAGIDEQLGGMPMPKALMWALHEHILG

ANPAVWMYAGGAGFANIFFHVATEEQKKWAVIAAERGWGSTMVLTEPDAGSDVGAGRTKA

VQQADGSWHIDGVKRFITSADSDDLFENIFHLVLARPEGAKPGTKGLSLFFVPKFLFDFE

TGELGERNGVFVTNVEHKMGLKVSATCELTFGQHGVPAKGWLVGEVHNGIAQMFDVIEQA

RMMVGTKAIATLSTGYLNALEYAKERVQGADLTQMTDKSAPRVTITHHPDVRRSLMTQKA

YAEGLRALYLYTSTYQDAAVAEKLHGVEPDLAVRINDLMLPIVKGVGSEQAYAKLTESLQ

TLGGSGFLQDYPIEQYIRDAKIDSLYEGTTAIQAQDFFFRKIIRDKGQALAHVAGEIEAF

IANESGNGRLKAERELLATALADVQGMAATLTGYLMAAQEDIASIYKVGLGSVRFLMSVG

DLMIGWLLARQAAVAVEALDAAAGDLSAADRAFYEGKIAVASFFTKNFLPLLTSTRSVID

AIDNDIMELDEAAF

>mmi:MMAR_0505 fadE5; acyl-CoA dehydrogenase FadE5 (A)

MSHYKSNVRDQVFNLFEVLGVDKALGEGQFSDLDTDTAREMLTEISRLAEGPVAASFIEG

DRNPPVFDPKTHTVTLPESFKDSVRAVIEAGWDKAGLDEELGGMPMPRALMWALHEHLLG

ANPAVWMYSGGAGFAQIMYHLGTEEQKKWAVMASERGWGSTMVLTEPDAGSDVGAGRTKA

VEQADGSWHIDGVKRFITSGDSDDLFENIFHLVLARPEGAGPGTKGLSLFFVPKFLFDFE

TGELGERNGVFVTNVEHKMGLKVSATCELSLGQHGVPAKGWLIGEVHEGIAQMFEVIEQA

RMMVGTKAIATLSTGYLNALEYAKSRVQGADLTQMTDKTAPRVTITHHPDVRRSLMTQKA

YAEGLRALYLYTATFQDAAVAEAAHGVDAKLAVKINDLLLPVVKGVGSEQAYAKLTESLQ

TLGGSGFLQDYPIEQYIRDAKIDSLYEGTTAIQAQDFFFRKIVRDKGVALAHVSGQIQEF

VDSETGNGRLKTERALLAKALTDVQAMAATLTGYLMAAQQDINSLYKVGLGSVRFLMSVG

DLVIAWLLQRQAAVAVAALDAGATGDERSFYEGKVAVASFFAKNFLPLLTSTREVIDTLD

NDIMELDEAAF

>mrh:MycrhN_1474 acyl-CoA dehydrogenase (A)

MSHYKSNVRDQEFNLFEVFGLDKVLGDGAYADIDAETARDMLGEMAKLAAGPVAESFADA

DRNPPVFDPKTHTVALPESFKKSMRVLLDGGWDKVGVLEELGGMPMPRALQWALIEHILG

ANPAAYMYAMGSGMAEIFYKNGTDEQKKWAVLAAERGWGATMVLTEPDAGSDVGAGRTKA

VQQADGTWHIDGVKRFITSGDSDDLFENIMHLVLARPEGAGPGTKGLSLFFVPKYHFDTE

TGEPGERNGVYVTNVEHKMGLKVSATCELSLGQHDKPAVGWLVGEVHDGIAQMFDVIEQA

RMMVGTKAIATLSTGYLNALEYAKERVQGADMTQMTDKTAPRVTITHHPDVRRSLMTQKA

YAEGLRALYLFTATYQDAAVAKAVHDIEPELAVKVNDLLLPIVKGVGSEQAYAKLTESLQ

TFGGSGFLQDYPVEQYIRDAKIDSLYEGTTAIQAQDFFFRKIVRDKGVALAHVAGQIEQF

VKNESGNGRLKAERALLATALEDVQAMAATLTGYLMGAQENPAELYKVGLGSVRFLMSVG

DLMIGWLLAKQAAVAIEALDKGAADADRAFYEGKVGVASFFAKNFLPLLTSTRTVIENLD

NDVMELDEASF

>mmm:W7S_24170 putative acyl-CoA dehydrogenase (A)

MSHYKSNVRDQAFNLFEVLGVDKALGQGEYSDLDVDTANEMLNEMSRLAEGPIADSFVEG

DRNPPVFDPKTHSVTLPESFKKSVHAVIEAGWDKVGIDEALGGVAMPKSLLWALHEHILG

ANPAVWMYAGGAGFANILYHLGTEEQKKWAVLAAERGWGSTMVLTEPDAGSDVGAGRTKA

VKQDDGSWHIDGVKRFITSADSGDLFENIFHLVLARPEGAGPGTKGLSLFFVPKFLFDFE

TGELGERNGVFVTNVEHKMGLKVSATCELSFGQHDVPAKGWLVGEVHNGIAQMFDVIEQA

RMMVGTKAIATLSTGYLNALEYAKSRVQGADMTQMTDKTAPRVTITHHPDVRRSLMTQKA

YAEGLRALYLFTSTYQDASVAEALHGVDAELAVKVNDLMLPVVKGVGSEQAYAKLTESLQ

TFGGSGFLQDYPIEQYIRDAKIDSLYEGTTAIQAQDFFFRKIVRDKGVALAHVSEQIQKF

VDSESGNGRLKTEREYLAKALTDVQAMATSLTGYLMAAQEDVTSLYKVGLGSVRFLMSVG

DLVIGWLLQRQAAVAVAALDAGASGDDKAFYEGKVAVASFFAKNFLPMLTSTREVIETLD

NDIMELDEAAF

>mcb:Mycch_0233 acyl-CoA dehydrogenase (A)

MSHYKSNVRDQVFNLFEVLGVDKALGEGAYADFDVETATEMLAEMARLAEGPVAESFEEG

DRNPPVFDPKTHTVTLPEGFKKSVRAVIEGGWDKLGIDEELGGTPMPRALSWALQEHVLG

ANPAVYMYAMGAGFADIFYHLGTEEQKKWAKLAADRGWGSTMVLTEPDAGSDVGAGRTKA

VQQPDGSWHIDGVKRFITSADSDDLFENIMHLVLARPEGAGPGTKGLSLFFVPKFLFDQE

TGEPGERNGVFVTNVEHKMGLKVSATCELSLGQHGVPAKGWLVGEVHNGIAQMFDVIEQA

RMMVGTKAIATLSTGYLNALAYAKDRVQGADLTQMTDKTAPRVSITHHPDVRRSLMTQKA

YAEGLRALYLYTATHQDAEVAKTLYGIEPELAVKVNDLLLPIVKGVGSEQAYAKLTESLQ

TFGGSGFLQDYPIEQYIRDAKIDSLYEGTTAIQAQDFFFRKIVRDKGVALAHVAGQIEQF

VKAETGNGRLKAERALLATALEDVQGMAATLTGYLMASQEDPASIYKVGLGSVRFLMSVG

DLVLGWLLQQQAAVAISKLDAGAEGDDRSFYEGKVAVASFFAKNFLPLLTSTRSIIDNLD

NEVMELDEAAF

>mli:MULP_00506 fadE5; acyl-CoA dehydrogenase FadE5 (EC:1.3.99.-) (A)

MSHYKSNVRDQVFNLFEVLGVDKALGEGQFSDLDTDTAREMLTEISRLAEGPVAASFIEG

DRNPPVFDPKTHTVTLPESFKDSVRAVIEAGWDKAGLDEELGGMPMPRALMGALHEHLLG

ANPAVWMYSGGAGFAQIMYHLGTEEQKKWAVMASERGWGSTMVLTEPDAGSDVGAGRTKA

VEQTDGSWHIDGVKRFITSGDSDDLFENIFHLVLARPEGAGPGTKGLSLFFVPKFLFDFE

TGELGERNGVFVTNVEHKMGLKVSATCELSLGQHGVPAKGWLIGEVHEGIAQMFEVIEQA

RMMVGTKAIATLSTGYLNALEYAKSRVQGADLTQMTDKTAPRVTITHHPDVRRSLMTQKA

YAEGLRALYLYTATFQDAAVAEAAHGVDAKLAVKINDLLLPVVKGVGSEQAYAKLTESLQ

TLGGSGFLQDYPIEQYIRDAKIDSLYEGTTAIQAQDFFFRKIVRDKGVALAHVSGQIQEF

VDSETGNGRLKTERALLAKALTDVQAMAATLTGYLMAAQQDINSLYKVGLGSVRFLMSVG

DLVIAWLLQRQAAVAVAALDAGATGDERSFYEGKVAVASFFAKNFLPLLTSTREVIDTLD

NDIMELDEAAF

>mkn:MKAN_16790 butyryl-CoA dehydrogenase (A)

MSHYKSNVRDQVFTLFEMLGVDQALGKGEFSDLDVDTAREMLVEVSRLAEGPVAASFVEG

DRNPPEFDPKTHSVTLPEAFKDSVRAVMDGGWDKAGIEEALGGMPMPKALVWALHEHFLG

ANPAVWMYASGGGFSNILYHLGTEEQKKWAVLAAERGWGSTMVLTEPDAGSDVGAGRTKA

VQQEDGSWHIDGVKRFITSGDSGDLFENIFHLVLARPEGAGPGTKGLSLFFVPKFLFDFE

TGELGERNGVFVTNVEHKMGLKVSATCELSLGQHGVPAKGWLVGEVHNGIAQMFEVIEQA

RMMVGTKAIATLSTGYLNALEYAKSRVQGADLTQMTDKTAPRVTITHHPDVRRSLMTQKA

YAEGLRALYLYTATFQDAAVAEVVHGVDAKLAAKVNDLMLPIVKGVGSEQAYAKLTESLQ

TLGGSGFLQDYPIEQYIRDAKIDSLYEGTTAIQAQDFFFRKIVRDKGVALAHVSGQIQEF

VDSESGNGRLKTERALLAKALADVQAMAAAMTGYLMAAQQDVTSLYKVGLGSVRFLMSVG

DLIIGWLLQRQAAVAVAALDAGATGEERAYYEGKVAVASFFAKNFLPLLTSTRDVIETLD

NDIMELDEAAF

>mks:LG40_16615 butyryl-CoA dehydrogenase (A)

MSHYKSNVRDQVFTLFEMLGVDQALGKGEFSDLDVDTAREMLVEVSRLAEGPVAASFVEG

DRNPPEFDPKTHSVTLPEAFKDSVRAVMDGGWDKAGIEEALGGMPMPKALVWALHEHFLG

ANPAVWMYASGGGFSNILYHLGTEEQKKWAVLAAERGWGSTMVLTEPDAGSDVGAGRTKA

VQQEDGSWHIDGVKRFITSGDSGDLFENIFHLVLARPEGAGPGTKGLSLFFVPKFLFDFE

TGELGERNGVFVTNVEHKMGLKVSATCELSLGQHGVPAKGWLVGEVHNGIAQMFEVIEQA

RMMVGTKAIATLSTGYLNALEYAKSRVQGADLTQMTDKTAPRVTITHHPDVRRSLMTQKA

YAEGLRALYLYTATFQDAAVAEVVHGVDAKLAAKVNDLMLPIVKGVGSEQAYAKLTESLQ

TLGGSGFLQDYPIEQYIRDAKIDSLYEGTTAIQAQDFFFRKIVRDKGVALAHVSGQIQEF

VDSESGNGRLKTERALLAKALADVQAMAAAMTGYLMAAQQDVTSLYKVGLGSVRFLMSVG

DLIIGWLLQRQAAVAVAALDAGATGEERAYYEGKVAVASFFAKNFLPLLTSTRDVIETLD

NDIMELDEAAF

>mki:LH54_16725 butyryl-CoA dehydrogenase (A)

MSHYKSNVRDQVFTLFEMLGVDQALGKGEFSDLDVDTAREMLVEVSRLAEGPVAASFVEG

DRNPPEFDPKTHSVTLPEAFKDSVRAVMDGGWDKAGIEEALGGMPMPKALVWALHEHFLG

ANPAVWMYASGGGFSNILYHLGTEEQKKWAVLAAERGWGSTMVLTEPDAGSDVGAGRTKA

VQQEDGSWHIDGVKRFITSGDSGDLFENIFHLVLARPEGAGPGTKGLSLFFVPKFLFDFE

TGELGERNGVFVTNVEHKMGLKVSATCELSLGQHGVPAKGWLVGEVHNGIAQMFEVIEQA

RMMVGTKAIATLSTGYLNALEYAKSRVQGADLTQMTDKTAPRVTITHHPDVRRSLMTQKA

YAEGLRALYLYTATFQDAAVAEVVHGVDAKLAAKVNDLMLPIVKGVGSEQAYAKLTESLQ

TLGGSGFLQDYPIEQYIRDAKIDSLYEGTTAIQAQDFFFRKIVRDKGVALAHVSGQIQEF

VDSESGNGRLKTERALLAKALADVQAMAAAMTGYLMAAQQDVTSLYKVGLGSVRFLMSVG

DLIIGWLLQRQAAVAVAALDAGATGEERAYYEGKVAVASFFAKNFLPLLTSTRDVIETLD

NDIMELDEAAF

>mne:D174_03055 butyryl-CoA dehydrogenase (A)

MSHYKSNVRDQVFTLFDVLGLDKALGEGSFAELDADTAREMINEMARLAEGPIAESFADG

DRNPPIFDPATHTVKLPESFKKSVNATIEGGWNKVALDEELGGMPAPKALLWALNEHILG

ANPAVWMYGGGAGFAQIFAHNGTEEQKKWAAIAAENNWGATMVLTEPDAGSDVGAGRTKA

VQQPDGTWHIDGVKRFITSADADDLFPNIAHLVLARPEGAGPGTKGLSLFFVPKFHFDKE

TGELGERNGVFVTNVEHKMGLKVSATCELTFGQHGVPAVGWLVGEVHNGIAQMFDVIEQA

RMMVGTKAIATLSTGYLNALEYAKERVQGADLTQMTDKTAPRVTITHHPDVRRSLMTQKA

YAEGMRALYMYTATFQDAEVAKALHDVDPDLAVRVNDLLLPVVKGFGSETAYRYLTESLQ

TLGGSGFLQDYPIEQYIRDAKIDSLYEGTTAIQAQDFFFRKIVRDKGQALAYVSGQIDTF

VKNETGNGRLKAERALLATALEDVQGMAATLTGYLMASQEDQSAIYKVGLGSVRFLMSVG

DLIIGWLLQREAAVAIEKLDEGATGADKAFYEGKIAAASFFAKNFLPLLTSTRTVLDALD

NEVMELDEASF

>myv:G155_01560 butyryl-CoA dehydrogenase (A)

MGHYKSNVRDQVFNLFDVFGLDKALGAGDYADLDADTAREMLNEMARLAEGPIAESFADG

DRNPPVFDPKTHSVALPESFKKSVRAAIDGGWDKVGLYEELGGVPAPKALLWALNEHILG

ANPAVWMYAGGAAFAQIFYDNATEEQKKWAVLASERGWGATMVLTEPDAGSDVGAGRTKA

VKQDDGSWHIDGVKRFITSADSDDLFENIFHLVLARPEGAGPGTKGLSLFFVPKFLFDFE

TGELGERNGVYVTNVEHKMGLKVSATCELTFGQHDKPAKGWLVGEVHNGIAQMFDVIEQA

RMMVGTKAIATLSTGYLNALEYAKERVQGADLTQMMDKTAPRVTITHHPDVRRSLMTQKS

YAEGMRALYLYTATFQDKDVAKALHGVDGELAHKVNDLLLPVVKGFGSEQAYAKLTESLQ

TLGGSGFLQDYPIEQYIRDAKIDSLYEGTTAIQAQDFFFRKIVRDKGQALAFVAGEIEQF

IKNETGNGRLKTERELLGTALADVQGMAASLTGYLMAAQEDAASIYKVGLGSVRFLLSVG

DLLLGWLLARQAAVAIEKLDAGATGEDRTYLEGKIAAASFFAKNMLPLLTSTRQVIETID

NEVMELDEAAF

>mye:AB431_02000 butyryl-CoA dehydrogenase (A)

MSHYKSNVRDQVFNLFEVFGFDKALGEGEYSDLDADTVQEMLGEMARLAEGPLAASFTDG

DRNPPVFDPESHTVTLPEPFKKSVKAFIDAGWDKVGLDEALGGMPMPKTLVWALHEHVLG

ANPAVWMYAGGAGFAQIVYNLGTEEQKKWAIKAAERGWGSTMVLTEPDAGSDVGAGRTKA

VKQDDGSWHIDGVKRFITSADSDDLFENILHLVLARPEGAGPGTKGLSLFIVPKYHFDFE

TGEPGERNGVFVTNVEHKMGLKVSATCELTFGQHGIPAKGWLVGEVHEGIAQMFDVIEQA

RMMVGTKAIATLSTGYLNALEYAKERVQGADLTQMTDKTAPRVTITHHPDVRRSLMTQKA

YAEGLRALYLYTSTYQDAAVAKDVQGVEADLAVRVNDLLLPIVKGVGSEQAYAKLTESLQ

TFGGSGFLQDYPVEQYIRDAKIDSLYEGTTAIQAQDFFFRKIVRDKGVALAHVAGQIESF

ISNESGNGRLKSERELLAKALEDVQAMAASMTANLMAAQEDAASIYKVGLASVRFLMSVG

DLVIGWLLQRQAAVAIAALDAGATGADRSFYEGKIAVASFFAKNMLPLLTSTRQVIETLD

NDIMELDEAAF

>mgo:AFA91_08965 butyryl-CoA dehydrogenase (A)

MSHYKSNVRDQVFNLFEVFGVDKVLGADKFSDLDADTAREMLTEIARLAEGPIAESFVEG

DRNPPVFDPETHTVTLPEGFKKSMRALFDGGWDKVGLAEHLGGIPMPRALQWALIEHILG

ANPAAYMYAMGPGMSEIFYNNGTDEQKKWATIAAERGWGATMVLTEPDAGSDVGAGRTKA

VQQPDGTWHIEGVKRFITSADSDDLFENIMHLVLARPEGAGPGTKGLSLFFVPKFHFDPE

TGEPGERNGVFVTNVEHKMGLKVSATCELSLGQHGIPAVGWLVGEVHNGIAQMFDVIEQA

RMMVGTKAIATLSTGYLNALEYAKERVQGADMTQMTDKTAPRVTITHHPDVRRSLMTQKA

YAEGLRAIYLYTATFQDAEVAKAVHGVEGDLAARVNDLLLPIVKGFGSETAYAKLTESLQ

TLGGSGFLQDYPIEQYIRDSKIDSLYEGTTAIQAQDFFFRKIIRDKGQALAYVAGEIEQF

IKNENGNGRLKAERELLATALADVQGMAAAMTGYLMAAQEEPASIYKVGLASVRFLMAVG

DLLAGWLLTRQAAVAIEKLDAGATGADKSFYEGKIAAASFFAKNMLPLLTSTRQIVENLD

NDVMELDEAAF

>mft:XA26_03350 DmdC-like protein (A)

MGHYKSNVRDQVFNLFDVFGLDKALGAGDYADLDADTAREMLNEMARLAEGPIAESFADG

DRNPPVFDPKTHSVALPESFKKSVRAAIDGGWDKVGLYEELGGVPAPKALLWALNEHILG

ANPAVWMYAGGAAFAQIFYDNATEEQKKWAVLASERGWGATMVLTEPDAGSDVGAGRTKA

VKQDDGSWHIDGVKRFITSADSDDLFENIFHLVLARPEGAGPGTKGLSLFFVPKFLFDFE

TGELGERNGVYVTNVEHKMGLKVSATCELTFGQHDKPAKGWLVGEVHNGIAQMFDVIEQA

RMMVGTKAIATLSTGYLNALEYAKERVQGADLTQMMDKTAPRVTITHHPDVRRSLMTQKS

YAEGMRALYLYTATFQDKDVAKALHGVDGELAHKVNDLLLPVVKGFGSEQAYAKLTESLQ

TLGGSGFLQDYPIEQYIRDAKIDSLYEGTTAIQAQDFFFRKIVRDKGQALAFVAGEIEQF

IKNETGNGRLKTERELLGTALADVQGMAASLTGYLMAAQEDAASIYKVGLGSVRFLLSVG

DLLLGWLLARQAAVAIEKLDAGATGEDRTYLEGKIAAASFFAKNMLPLLTSTRQVIETID

NEVMELDEAAF

>mhad:B586_01040 butyryl-CoA dehydrogenase (A)

MSHYKSNVRDQVFNLFEVLGVDKAFGAGQFSDLDTDTAHEMLTEVSRLAEGPVAASFADG

DRNPPVFHPETHSVTLPESFKKSCRAVAEAGWNKAGIDEALGGMPMPKALVWALHEHLLG

ANPAVWMYAGGAGFANILYHLGTEEQKKWAVLAAERNWGSTMVLTEPDAGSDVGAGRTKA

VQQEDGSWHIDGVKRFITSGDSDDLFENIFHLVLARPEGAGPGTKGLSLFFVPKFLFDFE

TGELGERNGVFVTNVEHKMGLKVSATCELSLGQHGVPAKGWLVGEVHDGIAQMFEVIEQA

RMMVGTKAIATLSTGYLNALEYAKSRVQGADLTQMTDKTAPRVTITHHPDVRRSLMTQKA

YAEGLRALYLYTATFQDAAVAEAVHGVDAKLAVKINDLMLPVVKGVGSEQAYAKLTESLQ

TLGGSGFLQDYPIEQYIRDAKIDSLYEGTTAIQAQDFFFRKIVRDKGVALAYVSGQIQQF

VDSETGNGRLKSERELLAKALADVQGMAATLTGYLMAAQQDVTSLYKVGLGSVRFLMSVG

DLIIGWLLQRQAAVAVAALDAGATGAERSFYEGKVVVSSFFAKNFLPLLTSTREVLEALD

NDIMELDEAAF

Rv0362 Homologs

>mtu:Rv0362 mgtE; Mg2+ transport transmembrane protein MgtE; K06213 magnesium transporter (A)

MSIRPAENSTLDIRHVIGIGTPKAVDLWLDVVTELPDRARELGSLSKAELGKLGPLLDGT

NAVELFESIDDKLAAEALHAMDPSLAATFLEALDSDHAANILREFKEPKREALLTLLPLE

RAMVLRGLLSWPEDCAAAHMVPETLTVRPNMTVSQAVASVRERASGLRSDARTTAYVYVT

DADSHLLGVIAFRALVLANPEQRVRELMGDDLIVVSPLTDKELAAQTIMGHNLMAVPVVD

ADNRLLGIIAEDEAIDIAEEEATEDAERQGGSAPLEVPYLRASPWLLWRKRVVWLLVLFA

AEAYTGSVLRAFSDEMEAVIALAFFIPLLIGTGGNTGTQIATTLVRAMATGQVRFRDVPA

VLAKELSTGVLVGLTMAAAAVVRAWTLGVGPQVTLTVALTVAAIVVWSSLVAAVLPPLLK

KLRIDPAIVSGPMIATIVDGTGLLIYFLVAHLTLTELHGL

>mtv:RVBD_0362 Mg2+ transport transmembrane protein MgtE; K06213 magnesium transporter (A)

MSIRPAENSTLDIRHVIGIGTPKAVDLWLDVVTELPDRARELGSLSKAELGKLGPLLDGT

NAVELFESIDDKLAAEALHAMDPSLAATFLEALDSDHAANILREFKEPKREALLTLLPLE

RAMVLRGLLSWPEDCAAAHMVPETLTVRPNMTVSQAVASVRERASGLRSDARTTAYVYVT

DADSHLLGVIAFRALVLANPEQRVRELMGDDLIVVSPLTDKELAAQTIMGHNLMAVPVVD

ADNRLLGIIAEDEAIDIAEEEATEDAERQGGSAPLEVPYLRASPWLLWRKRVVWLLVLFA

AEAYTGSVLRAFSDEMEAVIALAFFIPLLIGTGGNTGTQIATTLVRAMATGQVRFRDVPA

VLAKELSTGVLVGLTMAAAAVVRAWTLGVGPQVTLTVALTVAAIVVWSSLVAAVLPPLLK

KLRIDPAIVSGPMIATIVDGTGLLIYFLVAHLTLTELHGL

>mtc:MT0378 divalent cation transporter, MgtE family; K06213 magnesium transporter (A)

MSIRPAENSTLDIRHVIGIGTPKAVDLWLDVVTELPDRARELGSLSKAELGKLGPLLDGT

NAVELFESIDDKLAAEALHAMDPSLAATFLEALDSDHAANILREFKEPKREALLTLLPLE

RAMVLRGLLSWPEDCAAAHMVPETLTVRPNMTVSQAVASVRERASGLRSDARTTAYVYVT

DADSHLLGVIAFRALVLANPEQRVRELMGDDLIVVSPLTDKELAAQTIMGHNLMAVPVVD

ADNRLLGIIAEDEAIDIAEEEATEDAERQGGSAPLEVPYLRASPWLLWRKRVVWLLVLFA

AEAYTGSVLRAFSDEMEAVIALAFFIPLLIGTGGNTGTQIATTLVRAMATGQVRFRDVPA

VLAKELSTGVLVGLTMAAAAVVRAWTLGVGPQVTLTVALTVAAIVVWSSLVAAVLPPLLK

KLRIDPAIVSGPMIATIVDGTGLLIYFLVAHLTLTELHGL

>mra:MRA_0371 mgtE; Mg2+ transport transmembrane protein MgtE; K06213 magnesium transporter (A)

MSIRPAENSTLDIRHVIGIGTPKAVDLWLDVVTELPDRARELGSLSKAELGKLGPLLDGT

NAVELFESIDDKLAAEALHAMDPSLAATFLEALDSDHAANILREFKEPKREALLTLLPLE

RAMVLRGLLSWPEDCAAAHMVPETLTVRPNMTVSQAVASVRERASGLRSDARTTAYVYVT

DADSHLLGVIAFRALVLANPEQRVRELMGDDLIVVSPLTDKELAAQTIMGHNLMAVPVVD

ADNRLLGIIAEDEAIDIAEEEATEDAERQGGSAPLEVPYLRASPWLLWRKRVVWLLVLFA

AEAYTGSVLRAFSDEMEAVIALAFFIPLLIGTGGNTGTQIATTLVRAMATGQVRFRDVPA

VLAKELSTGVLVGLTMAAAAVVRAWTLGVGPQVTLTVALTVAAIVVWSSLVAAVLPPLLK

KLRIDPAIVSGPMIATIVDGTGLLIYFLVAHLTLTELHGL

>mtf:TBFG_10367 Mg2+ transport transmembrane protein mgtE; K06213 magnesium transporter (A)

MSIRPAENSTLDIRHVIGIGTPKAVDLWLDVVTELPDRARELGSLSKAELGKLGPLLDGT

NAVELFESIDDKLAAEALHAMDPSLAATFLEALDSDHAANILREFKEPKREALLTLLPLE

RAMVLRGLLSWPEDCAAAHMVPETLTVRPNMTVSQAVASVRERASGLRSDARTTAYVYVT

DADSHLLGVIAFRALVLANPEQRVRELMGDDLIVVSPLTDKELAAQTIMGHNLMAVPVVD

ADNRLLGIIAEDEAIDIAEEEATEDAERQGGSAPLEVPYLRASPWLLWRKRVVWLLVLFA

AEAYTGSVLRAFSDEMEAVIALAFFIPLLIGTGGNTGTQIATTLVRAMATGQVRFRDVPA

VLAKELSTGVLVGLTMAAAAVVRAWTLGVGPQVTLTVALTVAAIVVWSSLVAAVLPPLLK

KLRIDPAIVSGPMIATIVDGTGLLIYFLVAHLTLTELHGL

>mtb:TBMG_00366 Mg2+ transport transmembrane protein mgtE; K06213 magnesium transporter (A)

MSIRPAENSTLDIRHVIGIGTPKAVDLWLDVVTELPDRARELGSLSKAELGKLGPLLDGT

NAVELFESIDDKLAAEALHAMDPSLAATFLEALDSDHAANILREFKEPKREALLTLLPLE

RAMVLRGLLSWPEDCAAAHMVPETLTVRPNMTVSQAVASVRERASGLRSDARTTAYVYVT

DADSHLLGVIAFRALVLANPEQRVRELMGDDLIVVSPLTDKELAAQTIMGHNLMAVPVVD

ADNRLLGIIAEDEAIDIAEEEATEDAERQGGSAPLEVPYLRASPWLLWRKRVVWLLVLFA

AEAYTGSVLRAFSDEMEAVIALAFFIPLLIGTGGNTGTQIATTLVRAMATGQVRFRDVPA

VLAKELSTGVLVGLTMAAAAVVRAWTLGVGPQVTLTVALTVAAIVVWSSLVAAVLPPLLK

KLRIDPAIVSGPMIATIVDGTGLLIYFLVAHLTLTELHGL

>mtk:TBSG_00369 Mg2+ transport transmembrane protein mgtE; K06213 magnesium transporter (A)

MSIRPAENSTLDIRHVIGIGTPKAVDLWLDVVTELPDRARELGSLSKAELGKLGPLLDGT

NAVELFESIDDKLAAEALHAMDPSLAATFLEALDSDHAANILREFKEPKREALLTLLPLE

RAMVLRGLLSWPEDCAAAHMVPETLTVRPNMTVSQAVASVRERASGLRSDARTTAYVYVT

DADSHLLGVIAFRALVLANPEQRVRELMGDDLIVVSPLTDKELAAQTIMGHNLMAVPVVD

ADNRLLGIIAEDEAIDIAEEEATEDAERQGGSAPLEVPYLRASPWLLWRKRVVWLLVLFA

AEAYTGSVLRAFSDEMEAVIALAFFIPLLIGTGGNTGTQIATTLVRAMATGQVRFRDVPA

VLAKELSTGVLVGLTMAAAAVVRAWTLGVGPQVTLTVALTVAAIVVWSSLVAAVLPPLLK

KLRIDPAIVSGPMIATIVDGTGLLIYFLVAHLTLTELHGL

>mtz:TBXG_000364 Mg2+ transport transmembrane protein mgtE; K06213 magnesium transporter (A)

MSIRPAENSTLDIRHVIGIGTPKAVDLWLDVVTELPDRARELGSLSKAELGKLGPLLDGT

NAVELFESIDDKLAAEALHAMDPSLAATFLEALDSDHAANILREFKEPKREALLTLLPLE

RAMVLRGLLSWPEDCAAAHMVPETLTVRPNMTVSQAVASVRERASGLRSDARTTAYVYVT

DADSHLLGVIAFRALVLANPEQRVRELMGDDLIVVSPLTDKELAAQTIMGHNLMAVPVVD

ADNRLLGIIAEDEAIDIAEEEATEDAERQGGSAPLEVPYLRASPWLLWRKRVVWLLVLFA

AEAYTGSVLRAFSDEMEAVIALAFFIPLLIGTGGNTGTQIATTLVRAMATGQVRFRDVPA

VLAKELSTGVLVGLTMAAAAVVRAWTLGVGPQVTLTVALTVAAIVVWSSLVAAVLPPLLK

KLRIDPAIVSGPMIATIVDGTGLLIYFLVAHLTLTELHGL

>mtg:MRGA327_02295 putative Mg2+ transport transmembrane protein MGTE; K06213 magnesium transporter (A)

MSIRPAENSTLDIRHVIGIGTPKAVDLWLDVVTELPDRARELGSLSKAELGKLGPLLDGT

NAVELFESIDDKLAAEALHAMDPSLAATFLEALDSDHAANILREFKEPKREALLTLLPLE

RAMVLRGLLSWPEDCAAAHMVPETLTVRPNMTVSQAVASVRERASGLRSDARTTAYVYVT

DADSHLLGVIAFRALVLANPEQRVRELMGDDLIVVSPLTDKELAAQTIMGHNLMAVPVVD

ADNRLLGIIAEDEAIDIAEEEATEDAERQGGSAPLEVPYLRASPWLLWRKRVVWLLVLFA

AEAYTGSVLRAFSDEMEAVIALAFFIPLLIGTGGNTGTQIATTLVRAMATGQVRFRDVPA

VLAKELSTGVLVGLTMAAAAVVRAWTLGVGPQVTLTVALTVAAIVVWSSLVAAVLPPLLK

KLRIDPAIVSGPMIATIVDGTGLLIYFLVAHLTLTELHGL

>mti:MRGA423_02270 putative Mg2+ transport transmembrane protein MGTE; K06213 magnesium transporter (A)

MSIRPAENSTLDIRHVIGIGTPKAVDLWLDVVTELPDRARELGSLSKAELGKLGPLLDGT

NAVELFESIDDKLAAEALHAMDPSLAATFLEALDSDHAANILREFKEPKREALLTLLPLE

RAMVLRGLLSWPEDCAAAHMVPETLTVRPNMTVSQAVASVRERASGLRSDARTTAYVYVT

DADSHLLGVIAFRALVLANPEQRVRELMGDDLIVVSPLTDKELAAQTIMGHNLMAVPVVD

ADNRLLGIIAEDEAIDIAEEEATEDAERQGGSAPLEVPYLRASPWLLWRKRVVWLLVLFA

AEAYTGSVLRAFSDEMEAVIALAFFIPLLIGTGGNTGTQIATTLVRAMATGQVRFRDVPA

VLAKELSTGVLVGLTMAAAAVVRAWTLGVGPQVTLTVALTVAAIVVWSSLVAAVLPPLLK

KLRIDPAIVSGPMIATIVDGTGLLIYFLVAHLTLTELHGL

>mte:CCDC5079_0338 Mg2+ transport transmembrane protein MgtE; K06213 magnesium transporter (A)

MSIRPAENSTLDIRHVIGIGTPKAVDLWLDVVTELPDRARELGSLSKAELGKLGPLLDGT

NAVELFESIDDKLAAEALHAMDPSLAATFLEALDSDHAANILREFKEPKREALLTLLPLE

RAMVLRGLLSWPEDCAAAHMVPETLTVRPNMTVSQAVASVRERASGLRSDARTTAYVYVT

DADSHLLGVIAFRALVLANPEQRVRELMGDDLIVVSPLTDKELAAQTIMGHNLMAVPVVD

ADNRLLGIIAEDEAIDIAEEEATEDAERQGGSAPLEVPYLRASPWLLWRKRVVWLLVLFA

AEAYTGSVLRAFSDEMEAVIALAFFIPLLIGTGGNTGTQIATTLVRAMATGQVRFRDVPA

VLAKELSTGVLVGLTMAAAAVVRAWTLGVGPQVTLTVALTVAAIVVWSSLVAAVLPPLLK

KLRIDPAIVSGPMIATIVDGTGLLIYFLVAHLTLTELHGL

>mtur:CFBS_0378 hypothetical protein; K06213 magnesium transporter (A)

MSIRPAENSTLDIRHVIGIGTPKAVDLWLDVVTELPDRARELGSLSKAELGKLGPLLDGT

NAVELFESIDDKLAAEALHAMDPSLAATFLEALDSDHAANILREFKEPKREALLTLLPLE

RAMVLRGLLSWPEDCAAAHMVPETLTVRPNMTVSQAVASVRERASGLRSDARTTAYVYVT

DADSHLLGVIAFRALVLANPEQRVRELMGDDLIVVSPLTDKELAAQTIMGHNLMAVPVVD

ADNRLLGIIAEDEAIDIAEEEATEDAERQGGSAPLEVPYLRASPWLLWRKRVVWLLVLFA

AEAYTGSVLRAFSDEMEAVIALAFFIPLLIGTGGNTGTQIATTLVRAMATGQVRFRDVPA

VLAKELSTGVLVGLTMAAAAVVRAWTLGVGPQVTLTVALTVAAIVVWSSLVAAVLPPLLK

KLRIDPAIVSGPMIATIVDGTGLLIYFLVAHLTLTELHGL

>mtl:CCDC5180_0335 Mg2+ transport transmembrane protein MgtE; K06213 magnesium transporter (A)

MSIRPAENSTLDIRHVIGIGTPKAVDLWLDVVTELPDRARELGSLSKAELGKLGPLLDGT

NAVELFESIDDKLAAEALHAMDPSLAATFLEALDSDHAANILREFKEPKREALLTLLPLE

RAMVLRGLLSWPEDCAAAHMVPETLTVRPNMTVSQAVASVRERASGLRSDARTTAYVYVT

DADSHLLGVIAFRALVLANPEQRVRELMGDDLIVVSPLTDKELAAQTIMGHNLMAVPVVD

ADNRLLGIIAEDEAIDIAEEEATEDAERQGGSAPLEVPYLRASPWLLWRKRVVWLLVLFA

AEAYTGSVLRAFSDEMEAVIALAFFIPLLIGTGGNTGTQIATTLVRAMATGQVRFRDVPA

VLAKELSTGVLVGLTMAAAAVVRAWTLGVGPQVTLTVALTVAAIVVWSSLVAAVLPPLLK

KLRIDPAIVSGPMIATIVDGTGLLIYFLVAHLTLTELHGL

>mto:MTCTRI2_0369 mgtE; Mg2+ transport transmembrane protein MgtE; K06213 magnesium transporter (A)

MSIRPAENSTLDIRHVIGIGTPKAVDLWLDVVTELPDRARELGSLSKAELGKLGPLLDGT

NAVELFESIDDKLAAEALHAMDPSLAATFLEALDSDHAANILREFKEPKREALLTLLPLE

RAMVLRGLLSWPEDCAAAHMVPETLTVRPNMTVSQAVASVRERASGLRSDARTTAYVYVT

DADSHLLGVIAFRALVLANPEQRVRELMGDDLIVVSPLTDKELAAQTIMGHNLMAVPVVD

ADNRLLGIIAEDEAIDIAEEEATEDAERQGGSAPLEVPYLRASPWLLWRKRVVWLLVLFA

AEAYTGSVLRAFSDEMEAVIALAFFIPLLIGTGGNTGTQIATTLVRAMATGQVRFRDVPA

VLAKELSTGVLVGLTMAAAAVVRAWTLGVGPQVTLTVALTVAAIVVWSSLVAAVLPPLLK

KLRIDPAIVSGPMIATIVDGTGLLIYFLVAHLTLTELHGL

>mtd:UDA_0362 mgtE; mgtE; K06213 magnesium transporter (A)

MSIRPAENSTLDIRHVIGIGTPKAVDLWLDVVTELPDRARELGSLSKAELGKLGPLLDGT

NAVELFESIDDKLAAEALHAMDPSLAATFLEALDSDHAANILREFKEPKREALLTLLPLE

RAMVLRGLLSWPEDCAAAHMVPETLTVRPNMTVSQAVASVRERASGLRSDARTTAYVYVT

DADSHLLGVIAFRALVLANPEQRVRELMGDDLIVVSPLTDKELAAQTIMGHNLMAVPVVD

ADNRLLGIIAEDEAIDIAEEEATEDAERQGGSAPLEVPYLRASPWLLWRKRVVWLLVLFA

AEAYTGSVLRAFSDEMEAVIALAFFIPLLIGTGGNTGTQIATTLVRAMATGQVRFRDVPA

VLAKELSTGVLVGLTMAAAAVVRAWTLGVGPQVTLTVALTVAAIVVWSSLVAAVLPPLLK

KLRIDPAIVSGPMIATIVDGTGLLIYFLVAHLTLTELHGL

>mtn:ERDMAN_0402 mgtE; Mg2+ transport transmembrane protein; K06213 magnesium transporter (A)

MSIRPAENSTLDIRHVIGIGTPKAVDLWLDVVTELPDRARELGSLSKAELGKLGPLLDGT

NAVELFESIDDKLAAEALHAMDPSLAATFLEALDSDHAANILREFKEPKREALLTLLPLE

RAMVLRGLLSWPEDCAAAHMVPETLTVRPNMTVSQAVASVRERASGLRSDARTTAYVYVT

DADSHLLGVIAFRALVLANPEQRVRELMGDDLIVVSPLTDKELAAQTIMGHNLMAVPVVD

ADNRLLGIIAEDEAIDIAEEEATEDAERQGGSAPLEVPYLRASPWLLWRKRVVWLLVLFA

AEAYTGSVLRAFSDEMEAVIALAFFIPLLIGTGGNTGTQIATTLVRAMATGQVRFRDVPA

VLAKELSTGVLVGLTMAAAAVVRAWTLGVGPQVTLTVALTVAAIVVWSSLVAAVLPPLLK

KLRIDPAIVSGPMIATIVDGTGLLIYFLVAHLTLTELHGL

>mtj:J112_01945 Mg2+ transport transmembrane protein; K06213 magnesium transporter (A)

MSIRPAENSTLDIRHVIGIGTPKAVDLWLDVVTELPDRARELGSLSKAELGKLGPLLDGT

NAVELFESIDDKLAAEALHAMDPSLAATFLEALDSDHAANILREFKEPKREALLTLLPLE

RAMVLRGLLSWPEDCAAAHMVPETLTVRPNMTVSQAVASVRERASGLRSDARTTAYVYVT

DADSHLLGVIAFRALVLANPEQRVRELMGDDLIVVSPLTDKELAAQTIMGHNLMAVPVVD

ADNRLLGIIAEDEAIDIAEEEATEDAERQGGSAPLEVPYLRASPWLLWRKRVVWLLVLFA

AEAYTGSVLRAFSDEMEAVIALAFFIPLLIGTGGNTGTQIATTLVRAMATGQVRFRDVPA

VLAKELSTGVLVGLTMAAAAVVRAWTLGVGPQVTLTVALTVAAIVVWSSLVAAVLPPLLK

KLRIDPAIVSGPMIATIVDGTGLLIYFLVAHLTLTELHGL

>mtub:MT7199_0368 putative Mg2+ TRANSPORT protein protein MGTE; K06213 magnesium transporter (A)

MSIRPAENSTLDIRHVIGIGTPKAVDLWLDVVTELPDRARELGSLSKAELGKLGPLLDGT

NAVELFESIDDKLAAEALHAMDPSLAATFLEALDSDHAANILREFKEPKREALLTLLPLE

RAMVLRGLLSWPEDCAAAHMVPETLTVRPNMTVSQAVASVRERASGLRSDARTTAYVYVT

DADSHLLGVIAFRALVLANPEQRVRELMGDDLIVVSPLTDKELAAQTIMGHNLMAVPVVD

ADNRLLGIIAEDEAIDIAEEEATEDAERQGGSAPLEVPYLRASPWLLWRKRVVWLLVLFA

AEAYTGSVLRAFSDEMEAVIALAFFIPLLIGTGGNTGTQIATTLVRAMATGQVRFRDVPA

VLAKELSTGVLVGLTMAAAAVVRAWTLGVGPQVTLTVALTVAAIVVWSSLVAAVLPPLLK

KLRIDPAIVSGPMIATIVDGTGLLIYFLVAHLTLTELHGL

>mtuc:J113_02595 Mg2+ transport transmembrane protein; K06213 magnesium transporter (A)

MSIRPAENSTLDIRHVIGIGTPKAVDLWLDVVTELPDRARELGSLSKAELGKLGPLLDGT

NAVELFESIDDKLAAEALHAMDPSLAATFLEALDSDHAANILREFKEPKREALLTLLPLE

RAMVLRGLLSWPEDCAAAHMVPETLTVRPNMTVSQAVASVRERASGLRSDARTTAYVYVT

DADSHLLGVIAFRALVLANPEQRVRELMGDDLIVVSPLTDKELAAQTIMGHNLMAVPVVD

ADNRLLGIIAEDEAIDIAEEEATEDAERQGGSAPLEVPYLRASPWLLWRKRVVWLLVLFA

AEAYTGSVLRAFSDEMEAVIALAFFIPLLIGTGGNTGTQIATTLVRAMATGQVRFRDVPA

VLAKELSTGVLVGLTMAAAAVVRAWTLGVGPQVTLTVALTVAAIVVWSSLVAAVLPPLLK

KLRIDPAIVSGPMIATIVDGTGLLIYFLVAHLTLTELHGL

>mtue:J114_01935 Mg2+ transport transmembrane protein; K06213 magnesium transporter (A)

MSIRPAENSTLDIRHVIGIGTPKAVDLWLDVVTELPDRARELGSLSKAELGKLGPLLDGT

NAVELFESIDDKLAAEALHAMDPSLAATFLEALDSDHAANILREFKEPKREALLTLLPLE

RAMVLRGLLSWPEDCAAAHMVPETLTVRPNMTVSQAVASVRERASGLRSDARTTAYVYVT

DADSHLLGVIAFRALVLANPEQRVRELMGDDLIVVSPLTDKELAAQTIMGHNLMAVPVVD

ADNRLLGIIAEDEAIDIAEEEATEDAERQGGSAPLEVPYLRASPWLLWRKRVVWLLVLFA

AEAYTGSVLRAFSDEMEAVIALAFFIPLLIGTGGNTGTQIATTLVRAMATGQVRFRDVPA

VLAKELSTGVLVGLTMAAAAVVRAWTLGVGPQVTLTVALTVAAIVVWSSLVAAVLPPLLK

KLRIDPAIVSGPMIATIVDGTGLLIYFLVAHLTLTELHGL

>mtx:M943_01890 magnesium transporter; K06213 magnesium transporter (A)

MSIRPAENSTLDIRHVIGIGTPKAVDLWLDVVTELPDRARELGSLSKAELGKLGPLLDGT

NAVELFESIDDKLAAEALHAMDPSLAATFLEALDSDHAANILREFKEPKREALLTLLPLE

RAMVLRGLLSWPEDCAAAHMVPETLTVRPNMTVSQAVASVRERASGLRSDARTTAYVYVT

DADSHLLGVIAFRALVLANPEQRVRELMGDDLIVVSPLTDKELAAQTIMGHNLMAVPVVD

ADNRLLGIIAEDEAIDIAEEEATEDAERQGGSAPLEVPYLRASPWLLWRKRVVWLLVLFA

AEAYTGSVLRAFSDEMEAVIALAFFIPLLIGTGGNTGTQIATTLVRAMATGQVRFRDVPA

VLAKELSTGVLVGLTMAAAAVVRAWTLGVGPQVTLTVALTVAAIVVWSSLVAAVLPPLLK

KLRIDPAIVSGPMIATIVDGTGLLIYFLVAHLTLTELHGL

>mtuh:I917_02585 Mg2+ transporter; K06213 magnesium transporter (A)

MSXRPAENSTLDIRHVIGIGTPKAVDLWLDVVTELPDRARELGSLSKAELGKLGPLLDGT

NAVELFESIDDKLAAEALHAMDPSLAATFLXALDSDHAANILREFKEPKREALLTLLPLE

RAMVLRGLLSWPEDCAAAHMVPETLTVRPNMTVSQAVASVRERASGLRSDARTTAYVYVT

DADSHLLGVIAFRALVLANPEQRVRELMGDDLIVVSPLTDKELAAQTIMGHNLMAVPVVD

ADNRLLGIIAEDEAIDIAEEEATEDAERQGGSAPLEVPYLRASPWLLWRKRVXWLLVLFA

AEAYTGSVXRXFSDEMEAXXALAFFIPLLIGTGGNTGTQIATTLVRAMATGQVRFRDVPA

VLAKELSTGVLVGLTMAAAAVVRAWTLGVGPQVTLTXXXTVAAIVVWSSLVAXVLPPLLK

KLRIDPAIVSGPMIATIVDGTGLLIYFLVAHLTLTELHGL

>mtul:TBHG_00357 Mg2+ transport transmembrane protein MgtE; K06213 magnesium transporter (A)

MSIRPAENSTLDIRHVIGIGTPKAVDLWLDVVTELPDRARELGSLSKAELGKLGPLLDGT

NAVELFESIDDKLAAEALHAMDPSLAATFLEALDSDHAANILREFKEPKREALLTLLPLE

RAMVLRGLLSWPEDCAAAHMVPETLTVRPNMTVSQAVASVRERASGLRSDARTTAYVYVT

DADSHLLGVIAFRALVLANPEQRVRELMGDDLIVVSPLTDKELAAQTIMGHNLMAVPVVD

ADNRLLGIIAEDEAIDIAEEEATEDAERQGGSAPLEVPYLRASPWLLWRKRVVWLLVLFA

AEAYTGSVLRAFSDEMEAVIALAFFIPLLIGTGGNTGTQIATTLVRAMATGQVRFRDVPA

VLAKELSTGVLVGLTMAAAAVVRAWTLGVGPQVTLTVALTVAAIVVWSSLVAAVLPPLLK

KLRIDPAIVSGPMIATIVDGTGLLIYFLVAHLTLTELHGL

>mtut:HKBT1_0378 hypothetical protein; K06213 magnesium transporter (A)

MSIRPAENSTLDIRHVIGIGTPKAVDLWLDVVTELPDRARELGSLSKAELGKLGPLLDGT

NAVELFESIDDKLAAEALHAMDPSLAATFLEALDSDHAANILREFKEPKREALLTLLPLE

RAMVLRGLLSWPEDCAAAHMVPETLTVRPNMTVSQAVASVRERASGLRSDARTTAYVYVT

DADSHLLGVIAFRALVLANPEQRVRELMGDDLIVVSPLTDKELAAQTIMGHNLMAVPVVD

ADNRLLGIIAEDEAIDIAEEEATEDAERQGGSAPLEVPYLRASPWLLWRKRVVWLLVLFA

AEAYTGSVLRAFSDEMEAVIALAFFIPLLIGTGGNTGTQIATTLVRAMATGQVRFRDVPA

VLAKELSTGVLVGLTMAAAAVVRAWTLGVGPQVTLTVALTVAAIVVWSSLVAAVLPPLLK

KLRIDPAIVSGPMIATIVDGTGLLIYFLVAHLTLTELHGL

>mtuu:HKBT2_0378 hypothetical protein; K06213 magnesium transporter (A)

MSIRPAENSTLDIRHVIGIGTPKAVDLWLDVVTELPDRARELGSLSKAELGKLGPLLDGT

NAVELFESIDDKLAAEALHAMDPSLAATFLEALDSDHAANILREFKEPKREALLTLLPLE

RAMVLRGLLSWPEDCAAAHMVPETLTVRPNMTVSQAVASVRERASGLRSDARTTAYVYVT

DADSHLLGVIAFRALVLANPEQRVRELMGDDLIVVSPLTDKELAAQTIMGHNLMAVPVVD

ADNRLLGIIAEDEAIDIAEEEATEDAERQGGSAPLEVPYLRASPWLLWRKRVVWLLVLFA

AEAYTGSVLRAFSDEMEAVIALAFFIPLLIGTGGNTGTQIATTLVRAMATGQVRFRDVPA

VLAKELSTGVLVGLTMAAAAVVRAWTLGVGPQVTLTVALTVAAIVVWSSLVAAVLPPLLK

KLRIDPAIVSGPMIATIVDGTGLLIYFLVAHLTLTELHGL

>mtq:HKBS1_0378 hypothetical protein; K06213 magnesium transporter (A)

MSIRPAENSTLDIRHVIGIGTPKAVDLWLDVVTELPDRARELGSLSKAELGKLGPLLDGT

NAVELFESIDDKLAAEALHAMDPSLAATFLEALDSDHAANILREFKEPKREALLTLLPLE

RAMVLRGLLSWPEDCAAAHMVPETLTVRPNMTVSQAVASVRERASGLRSDARTTAYVYVT

DADSHLLGVIAFRALVLANPEQRVRELMGDDLIVVSPLTDKELAAQTIMGHNLMAVPVVD

ADNRLLGIIAEDEAIDIAEEEATEDAERQGGSAPLEVPYLRASPWLLWRKRVVWLLVLFA

AEAYTGSVLRAFSDEMEAVIALAFFIPLLIGTGGNTGTQIATTLVRAMATGQVRFRDVPA

VLAKELSTGVLVGLTMAAAAVVRAWTLGVGPQVTLTVALTVAAIVVWSSLVAAVLPPLLK

KLRIDPAIVSGPMIATIVDGTGLLIYFLVAHLTLTELHGL

>mbo:Mb0369 mgtE; Mg2+ transporter; K06213 magnesium transporter (A)

MSIRPAENSTLDIRHVIGIGTPKAVDLWLDVVTELPDRARELGSLSKAELGKLGPLLDGT

NAVELFESIDDKLAAEALHAMDPSLAATFLEALDSDHAANILREFKEPKREALLTLLPLE

RAMVLRGLLSWPEDCAAAHMVPETLTVRPNMTVSQAVASVRERASGLRSDARTTAYVYVT

DADSHLLGVIAFRALVLANPEQRVRELMGDDLIVVSPLTDKELAAQTIMGHNLMAVPVVD

ADNRLLGIIAEDEAIDIAEEEATEDAERQGGSAPLEVPYLRASPWLLWRKRAVWLLVLFA

AEAYTGSVLRAFSDEMEAVIALAFFIPLLIGTGGNTGTQIATTLVRAMATGQVRFRDVPA

VLAKELSTGVLVGLTMAAAAVVRAWTLGVGPQVTLTVALTVAAIVVWSSLVAAVLPPLLK

KLRIDPAIVSGPMIATIVDGTGLLIYFLVAHLTLTELHGL

>mbb:BCG_0400 mgtE; Possible Mg2+ transport transmembrane protein mgtE; K06213 magnesium transporter (A)

MSIRPAENSTLDIRHVIGIGTPKAVDLWLDVVTELPDRARELGSLSKAELGKLGPLLDGT

NAVELFESIDDKLAAEALHAMDPSLAATFLEALDSDHAANILREFKEPKREALLTLLPLE

RAMVLRGLLSWPEDCAAAHMVPETLTVRPNMTVSQAVASVRERASGLRSDARTTAYVYVT

DADSHLLGVIAFRALVLANPEQRVRELMGDDLIVVSPLTDKELAAQTIMGHNLMAVPVVD

ADNRLLGIIAEDEAIDIAEEEATEDAERQGGSAPLEVPYLRASPWLLWRKRVVWLLVLFA

AEAYTGSVLRAFSDEMEAVIALAFFIPLLIGTGGNTGTQIATTLVRAMATGQVRFRDVPA

VLAKELSTGVLVGLTMAAAAVVRAWTLGVGPQVTLTVALTVAAIVVWSSLVAAVLPPLLK

KLRIDPAIVSGPMIATIVDGTGLLIYFLVAHLTLTELHGL

>mbt:JTY_0370 mgtE; putative Mg2+ transport transmembrane protein; K06213 magnesium transporter (A)

MSIRPAENSTLDIRHVIGIGTPKAVDLWLDVVTELPDRARELGSLSKAELGKLGPLLDGT

NAVELFESIDDKLAAEALHAMDPSLAATFLEALDSDHAANILREFKEPKREALLTLLPLE

RAMVLRGLLSWPEDCAAAHMVPETLTVRPNMTVSQAVASVRERASGLRSDARTTAYVYVT

DADSHLLGVIAFRALVLANPEQRVRELMGDDLIVVSPLTDKELAAQTIMGHNLMAVPVVD

ADNRLLGIIAEDEAIDIAEEEATEDAERQGGSAPLEVPYLRASPWLLWRKRVVWLLVLFA

AEAYTGSVLRAFSDEMEAVIALAFFIPLLIGTGGNTGTQIATTLVRAMATGQVRFRDVPA

VLAKELSTGVLVGLTMAAAAVVRAWTLGVGPQVTLTVALTVAAIVVWSSLVAAVLPPLLK

KLRIDPAIVSGPMIATIVDGTGLLIYFLVAHLTLTELHGL

>mbm:BCGMEX_0370 mgtE; Putative Mg2+ transport transmembrane protein; K06213 magnesium transporter (A)

MSIRPAENSTLDIRHVIGIGTPKAVDLWLDVVTELPDRARELGSLSKAELGKLGPLLDGT

NAVELFESIDDKLAAEALHAMDPSLAATFLEALDSDHAANILREFKEPKREALLTLLPLE

RAMVLRGLLSWPEDCAAAHMVPETLTVRPNMTVSQAVASVRERASGLRSDARTTAYVYVT

DADSHLLGVIAFRALVLANPEQRVRELMGDDLIVVSPLTDKELAAQTIMGHNLMAVPVVD

ADNRLLGIIAEDEAIDIAEEEATEDAERQGGSAPLEVPYLRASPWLLWRKRVVWLLVLFA

AEAYTGSVLRAFSDEMEAVIALAFFIPLLIGTGGNTGTQIATTLVRAMATGQVRFRDVPA

VLAKELSTGVLVGLTMAAAAVVRAWTLGVGPQVTLTVALTVAAIVVWSSLVAAVLPPLLK

KLRIDPAIVSGPMIATIVDGTGLLIYFLVAHLTLTELHGL

>mbk:K60_003820 Mg2+ transporter; K06213 magnesium transporter (A)

MSIRPAENSTLDIRHVIGIGTPKAVDLWLDVVTELPDRARELGSLSKAELGKLGPLLDGT

NAVELFESIDDKLAAEALHAMDPSLAATFLEALDSDHAANILREFKEPKREALLTLLPLE

RAMVLRGLLSWPEDCAAAHMVPETLTVRPNMTVSQAVASVRERASGLRSDARTTAYVYVT

DADSHLLGVIAFRALVLANPEQRVRELMGDDLIVVSPLTDKELAAQTIMGHNLMAVPVVD

ADNRLLGIIAEDEAIDIAEEEATEDAERQGGSAPLEVPYLRASPWLLWRKRVVWLLVLFA

AEAYTGSVLRAFSDEMEAVIALAFFIPLLIGTGGNTGTQIATTLVRAMATGQVRFRDVPA

VLAKELSTGVLVGLTMAAAAVVRAWTLGVGPQVTLTVALTVAAIVVWSSLVAAVLPPLLK

KLRIDPAIVSGPMIATIVDGTGLLIYFLVAHLTLTELHGL

>mbx:BCGT_0131 putative mg2+ transport transmembrane protein mgte; K06213 magnesium transporter (A)

MSIRPAENSTLDIRHVIGIGTPKAVDLWLDVVTELPDRARELGSLSKAELGKLGPLLDGT

NAVELFESIDDKLAAEALHAMDPSLAATFLEALDSDHAANILREFKEPKREALLTLLPLE

RAMVLRGLLSWPEDCAAAHMVPETLTVRPNMTVSQAVASVRERASGLRSDARTTAYVYVT

DADSHLLGVIAFRALVLANPEQRVRELMGDDLIVVSPLTDKELAAQTIMGHNLMAVPVVD

ADNRLLGIIAEDEAIDIAEEEATEDAERQGGSAPLEVPYLRASPWLLWRKRVVWLLVLFA

AEAYTGSVLRAFSDEMEAVIALAFFIPLLIGTGGNTGTQIATTLVRAMATGQVRFRDVPA

VLAKELSTGVLVGLTMAAAAVVRAWTLGVGPQVTLTVALTVAAIVVWSSLVAAVLPPLLK

KLRIDPAIVSGPMIATIVDGTGLLIYFLVAHLTLTELHGL

>mbz:LH58_01955 magnesium transporter; K06213 magnesium transporter (A)

MSIRPAENSTLDIRHVIGIGTPKAVDLWLDVVTELPDRARELGSLSKAELGKLGPLLDGT

NAVELFESIDDKLAAEALHAMDPSLAATFLEALDSDHAANILREFKEPKREALLTLLPLE

RAMVLRGLLSWPEDCAAAHMVPETLTVRPNMTVSQAVASVRERASGLRSDARTTAYVYVT

DADSHLLGVIAFRALVLANPEQRVRELMGDDLIVVSPLTDKELAAQTIMGHNLMAVPVVD

ADNRLLGIIAEDEAIDIAEEEATEDAERQGGSAPLEVPYLRASPWLLWRKRAVWLLVLFA

AEAYTGSVLRAFSDEMEAVIALAFFIPLLIGTGGNTGTQIATTLVRAMATGQVRFRDVPA

VLAKELSTGVLVGLTMAAAAVVRAWTLGVGPQVTLTVALTVAAIVVWSSLVAAVLPPLLK

KLRIDPAIVSGPMIATIVDGTGLLIYFLVAHLTLTELHGL

>maf:MAF_03640 mgtE; putative Mg2+ transport transmembrane protein MGTE; K06213 magnesium transporter (A)

MSIRPAENSTLDIRHVIGIGTPKAVDLWLDVVTELPDRARELGSLSKAELGKLGPLLDGT

NAVELFESIDDKLAAEALHAMDPSLAATFLEALDSDHAANILREFKEPKREALLTLLPLE

RAMVLRGLLSWPEDCAAAHMVPETLTVRPNMTVSQAVASVRERASGLRSDARTTAYVYVT

DADSHLLGVIAFRALVLANPEQRVRELMGDDLIVVSPLTDKELAAQTIMGHNLMAVPVVD

ADNRLLGIIAEDEAIDIAEEEATEDAERQGGSAPLEVPYLRASPWLLWRKRVVWLLVLFA

AEAYTGSVLRAFSDEMEAVIALAFFIPLLIGTGGNTGTQIATTLVRAMATGQVRFRDVPA

VLAKELSTGVLVGLTMAAAAVVRAWTLGVGPQVTLTVALTVAAIVVWSSLVAAVLPPLLK

KLRIDPAIVSGPMIATIVDGTGLLIYFLVAHLTLTELHGL

>mce:MCAN_03631 mgtE; putative Mg2+ transport transmembrane protein MGTE; K06213 magnesium transporter (A)

MSIRPAENSTLDIRHVIGIGTPKAVDLWLDVVTELPDRARELGSLSKAELGKLGPLLDGT

NAVELFESIDDKLAAEALHAMDPSLAATFLEALDSDHAANILREFKEPKREALLTLLPLE

RAMVLRGLLSWPEDCAAAHMVPETLTVRPNMTVSQAVASVRERASGLRSDARTTAYVYVT

DADSHLLGVIAFRALVLANPEQRVRELMGDDLIVVSPLTDKELAAQTIMGHNLMAVPVVD

ADNRLLGIIAEDEAIDIAEEEATEDAERQGGSAPLEVPYLRASPWLLWRKRVVWLLVLFA

AEAYTGSVLRAFSDEMEAVIALAFFIPLLIGTGGNTGTQIATTLVRAMATGQVRFRDVPA

VLAKELSTGVLVGLTMAAAAVVRAWTLGVGPQVTLTVALTVAAIVVWSSLVAAVLPPLLK

KLRIDPAIVSGPMIATIVDGTGLLIYFLVAHLTLTELHGL

>mcq:BN44_10403 Putative Mg2+ transport transmembrane protein MgtE; K06213 magnesium transporter (A)

MSIRPAENSTLDIRHVIGIGTPKAVDLWLDVVTELPDRARELGSLSKAELGKLGPLLDGT

NAVELFESIDDKLAAEALHAMDPSLAATFLEALDSDHAANILREFKEPKREALLTLLPLE

RAMVLRGLLSWPEDCAAAHMVPETLTVRPNMTVSQAVASVRERASGLRSDARTTAYVYVT

DADSHLLGVIAFRALVLANPEQRVRELMGDDLIVVSPLTDKELAAQTIMGHNLMAVPVVD

ADNRLLGIIAEDEAIDIAEEEATEDAERQGGSAPLEVPYLRASPWLLWRKRVVWLLVLFA

AEAYTGSVLRAFSDEMEAVIALAFFIPLLIGTGGNTGTQIATTLVRAMATGQVRFRDVPA

VLAKELSTGVLVGLTMAAAAVVRAWTLGVGPQVTLTVALTVAAIVVWSSLVAAVLPPLLK

KLRIDPAIVSGPMIATIVDGTGLLIYFLVAHLTLTELHGL

>mcv:BN43_10395 Putative Mg2+ transport transmembrane protein MgtE; K06213 magnesium transporter (A)

MSIRPAENSTLDIRHVIGIGTPKAVDLWLDVVTELPDRARELGSLSKAELGKLGPLLDGT

NAVELFESIDDKLAAEALHAMDPSLAATFLEALDSDHAANILREFKEPKREALLTLLPLE

RAMVLRGLLSWPEDCAAAHMVPETLTVRPNMTVSQAVASVRERASGLRSDARTTAYVYVT

DADSHLLGVIAFRALVLANPEQRVRELMGDDLIVVSPLTDKELAAQTIMGHNLMAVPVVD

ADNRLLGIIAEDEAIDIAEEEATEDAERQGGSAPLEVPYLRASPWLLWRKRVVWLLVLFA

AEAYTGSVLRAFSDEMEAVIALAFFIPLLIGTGGNTGTQIATTLVRAMATGQVRFRDVPA

VLAKELSTGVLVGLTMAGAAVVRAWTLGVGPQVTLTVALTVAAIVVWSSLVAAVLPPLLK

KLRIDPAIVSGPMIATIVDGTGLLIYFMVAHLTLTELHGL

>mcx:BN42_20089 Putative Mg2+ transport transmembrane protein MgtE; K06213 magnesium transporter (A)

MSIRPAENSTLDIRHVIGIGTPKAVDLWLDVVTELPDRARELGSLSKAELGKLGPLLDGT

NAVELFESIDDKLAAEALHAMDPSLAATFLEALDSDHAANILREFKEPKREALLTLLPLE

RAMVLRGLLSWPEDCAAAHMVPETLTVRPNMTVSQAVASVRERASGLRSDARTTAYVYVT

DADSHLLGVIAFRALVLANPEQRVRELMGDDLIVVSPLTDKELAAQTIMGHNLMAVPVVD

ADNRLLGIIAEDEAIDIAEEEATEDAERQGGSAPLEVPYLRASPWLLWRKRVVWLLVLFA

AEAYTGSVLRAFSDEMEAVIALAFFIPLLIGTGGNTGTQIATTLVRAMATGQVRFRDVPA

VLAKELSTGVLVGLTMAGAAVVRAWTLGVGPQVTLTVALTVAAIVVWSSLVAAVLPPLLK

KLRIDPAIVSGPMIATIVDGTGLLIYFMVAHLTLTELHGL

>mcz:BN45_10404 Putative Mg2+ transport transmembrane protein MgtE; K06213 magnesium transporter (A)

MSIRPAENSTLDIRHVIGIGTPKAVDLWLDVVTELPDRARELGSLSKAELGKLGPLLDGT

NAVELFESIDDKLAAEALHAMDPSLAATFLEALDSDHAANILREFKEPKREALLTLLPLE

RAMVLRGLLSWPEDCAAAHMVPETLTVRPNMTVSQAVASVRERASGLRSDARTTAYVYVT

DADAHLLGVIAFRALVLANPEQRVRELMGDDLIVVSPLTDKELAAQTIMGHNLMAVPVVD

ADNRLLGIIAEDEAIDIAEEEATEDAERQGGSAPLEVPYLRASPWLLWRKRVVWLLVLFA

AEAYTGSVLRAFSDEMEAVIALAFFIPLLIGTGGNTGTQIATTLVRAMATGQVRFRDVPA

VLAKELSTGVLVGLTMAGAAVVRAWTLGVGPQVTLTVALTVAAIVVWSSLVAAVLPPLLK

KLRIDPAIVSGPMIATIVDGTGLLIYFMVAHLTLTELHGL

>mle:ML0387 guaB2; inosine 5'-monophosphate dehydrogenase (EC:1.1.1.205); K00088 IMP dehydrogenase [EC:1.1.1.205] (A)

MIRGMSNLKESSDFVASSYVRLGGLMDDPAATGGDNPHKVAMLGLTFDDVLLLPAASDVV

PATADISSQLTKKIRLKVPLVSSAMDTVTEARMAIAMARAGGMGVLHRNLPVGEQAGQVE

TVKRSEAGMVTDPVTCRPDNTLAQVGALCARFRISGLPVVDDSGALAGIITNRDMRFEVD

QSKQVAEVMTKTPLITAAEGVSADAALGLLRRNKIEKLPVVDGHGRLTGLITVKDFVKTE

QHPLATKDNDGRLLVGAAVGVGGDAWVRAMMLVDAGVDVLIVDTAHAHNRLVLDMVGKLK

VEIGDRVQVIGGNVATRSAAAALVEAGADAVKVGVGPGSTCTTRVVAGVGAPQITAILEA

VAACGPAGVPVIADGGLQYSGDIAKALAAGASTTMLGSLLAGTAEAPGELIFVNGKQFKS

YRGMGSLGAMQGRGGDKSYSKDRYFADDALSEDKLVPEGIEGRVPFRGPLSSVIHQLVGG

LRAAMGYTGSPTIEVLQQAQFVRITPAGLKESHPHDVAMTVEAPNYYPR

>mlb:MLBr00387 guaB2; putative inosine-5'-monophosphate dehydrogenase (EC:1.1.1.205); K00088 IMP dehydrogenase [EC:1.1.1.205] (A)

MIRGMSNLKESSDFVASSYVRLGGLMDDPAATGGDNPHKVAMLGLTFDDVLLLPAASDVV

PATADISSQLTKKIRLKVPLVSSAMDTVTEARMAIAMARAGGMGVLHRNLPVGEQAGQVE

TVKRSEAGMVTDPVTCRPDNTLAQVGALCARFRISGLPVVDDSGALAGIITNRDMRFEVD

QSKQVAEVMTKTPLITAAEGVSADAALGLLRRNKIEKLPVVDGHGRLTGLITVKDFVKTE

QHPLATKDNDGRLLVGAAVGVGGDAWVRAMMLVDAGVDVLIVDTAHAHNRLVLDMVGKLK

VEIGDRVQVIGGNVATRSAAAALVEAGADAVKVGVGPGSTCTTRVVAGVGAPQITAILEA

VAACGPAGVPVIADGGLQYSGDIAKALAAGASTTMLGSLLAGTAEAPGELIFVNGKQFKS

YRGMGSLGAMQGRGGDKSYSKDRYFADDALSEDKLVPEGIEGRVPFRGPLSSVIHQLVGG

LRAAMGYTGSPTIEVLQQAQFVRITPAGLKESHPHDVAMTVEAPNYYPR

>mpa:MAP_2550 hypothetical protein (A)

MGSVNRVYIARLARILVLGPLGESVGRVRDVVISISIVRQQPRVLGLVVDLATRRSIFIP

ILRVAAIDPNAVTLSTGSVSLRHFEQRPGEVLAIGQVLDTVVKVNDPELPELAGVDVVVT

DLGIEQTRTRDWMVTRVAVRPQRRLRRRGPVHVVDWRNVQGLTPSALALPGQAVAQLLEQ

FEGRKPVDVADAIRGLPPKRRYEVLKALNDDRLADILQELPELDQAEVLSQLGTERSADV

LEEMDPDDAADLLGVLNPTDAEMLLKRMDPGDSASVRRLLTHSPDTAGGLMTSNPVVLTP

DTAVAEALARARDPDLTAALSSMVFVVRPPTATPTGRYLGCVPLQRLLREAPAELVGGIV

DSDLLTLRPETPLVAVTRYLAAYNLVCGPVVDDENHLLGAVTVDDLLDHLLPPDWRVDMQ

ELDTAGRLEGLGGSG

>mao:MAP4_1270 hypothetical protein (A)

MGSVNRVYIARLARILVLGPLGESVGRVRDVVISISIVRQQPRVLGLVVDLATRRSIFIP

ILRVAAIDPNAVTLSTGSVSLRHFEQRPGEVLAIGQVLDTVVKVNDPELPELAGVDVVVT

DLGIEQTRTRDWMVTRVAVRPQRRLRRRGPVHVVDWRNVQGLTPSALALPGQAVAQLLEQ

FEGRKPVDVADAIRGLPPKRRYEVLKALNDDRLADILQELPELDQAEVLSQLGTERSADV

LEEMDPDDAADLLGVLNPTDAEMLLKRMDPGDSASVRRLLTHSPDTAGGLMTSNPVVLTP

DTAVAEALARARDPDLTAALSSMVFVVRPPTATPTGRYLGCVPLQRLLREAPAELVGGIV

DSDLLTLRPETPLVAVTRYLAAYNLVCGPVVDDENHLLGAVTVDDLLDHLLPPDWRVDMQ

ELDTAGRLEGLGGSG

>mavi:RC58_06285 magnesium transporter (A)

MGSVNRVYIARLARILVLGPLGESVGRVRDVVISISIVRQQPRVLGLVVDLATRRSIFIP

ILRVAAIDPNAVTLSTGSVSLRHFEQRPGEVLAIGQVLDTVVKVNDPELPELAGVDVVVT

DLGIEQTRTRDWMVTRVAVRPQRRLRRRGPVHVVDWRNVQGLTPSALALPGQAVAQLLEQ

FEGRKPVDVADAIRGLPPKRRYEVLKALNDDRLADILQELPELDQAEVLSQLGTERSADV

LEEMDPDDAADLLGVLNPTDAEMLLKRMDPGDSASVRRLLTHSPDTAGGLMTSNPVVLTP

DTAVAEALARARVPDLTAALSSMVFVVRPPTATPTGRYLGCVPLQRLLREAPAELVGGIV

DSDLLTLRPETPLVAVTRYLAAYNLVCGPVVDDENHLLGAVTVDDLLDHLLPPDWRVDMQ

ELDTAGRLEGLGGSG

>mavu:RE97_06280 magnesium transporter (A)

MGSVNRVYIARLARILVLGPLGESVGRVRDVVISISIVRQQPRVLGLVVDLATRRSIFIP

ILRVAAIDPNAVTLSTGSVSLRHFEQRPGEVLAIGQVLDTVVKVNDPELPELAGVDVVVT

DLGIEQTRTRDWMVTRVAVRPQRRLRRRGPVHVVDWRNVQGLTPSALALPGQAVAQLLEQ

FEGRKPVDVADAIRGLPPKRRYEVLKALNDDRLADILQELPELDQAEVLSQLGTERSADV

LEEMDPDDAADLLGVLNPTDAEMLLKRMDPGDSASVRRLLTHSPDTAGGLMTSNPVVLTP

DTAVAEALARARDPDLTAALSSMVFVVRPPTATPTGRYLGCVPLQRLLREAPAELVGGIV

DSDLLTLRPETPLVAVTRYLAAYNLVCGPVVDDENHLLGAVTVDDLLDHLLPPDWRVDMQ

ELDTAGRLEGLGGSG

>mav:MAV_1371 Mg/Co/Ni transporter MgtE (A)

MYIARLARMLVLGPLGESVGRVRDVVISISIVRQQPRVLGLVVDLATRRSIFIPILRVAA

IDPNAVTLSTGSVSLRHFEQRPGEVLAIGQVLDTVVKVNDPELPELAGVDVVVTDLGIEQ

TRTRDWMVTRVAVRPQRRLRRRGPVHVVDWRNVQGLTPSALALPGQAVAQLLEQFEGRKP

VDVADAIRGLPPKRRYEVLKALNDDRLADILQELPELDQAEVLSQLGTERSADVLEEMDP

DDAADLLGVLNPTDAEMLLKRMDPGDSASVRRLLTHSPDTAGGLMTSNPVVLTPDTAVAE

ALARARDPDLTAALSSMVFVVRPPTATPTGRYLGCVPLQRLLREAPAELVGGIVDSDLLT

LRPETPLVAVTRYLAAYNLVCGPVVDDENHLLGAVTVDDLLDHLLPPDWRVDMQELDTAG

RLEGLGGSG

>mavr:LA63_06250 magnesium transporter (A)

MGSVNRVYIARLARMLVLGPLGESVGRVRDVVISISIVRQQPRVLGLVVDLATRRSIFIP

ILRVAAIDPNAVTLSTGSVSLRHFEQRPGEVLAIGQVLDTVVKVNDPELPELAGVDVVVT

DLGIEQTRTRDWMVTRVAVRPQRRLRRRGPVHVVDWRNVQGLTPSALALPGQAVAQLLEQ

FEGRKPVDVADAIRGLPPKRRYEVLKALNDDRLADILQELPELDQAEVLSQLGTERSADV

LEEMDPDDAADLLGVLNPTDAEMLLKRMDPGDSASVRRLLTHSPDTAGGLMTSNPVVLTP

DTAVAEALARARDPDLTAALSSMVFVVRPPTATPTGRYLGCVPLQRLLREAPAELVGGIV

DSDLLTLRPETPLVAVTRYLAAYNLVCGPVVDDENHLLGAVTVDDLLDHLLPPDWRGGMQ

ELDTARRLEGLGGSG

>mavd:NF84_09545 magnesium transporter MgtE (A)

MGTSEDKAPGQPVIHLSQLLRAPVLARSGETVGRVEDVIVRLRGADEYPLVTGIVAGVGG

RRVFVGDKSIHEYSADRVLLTKNKIDLRGFERREGEVLLRTDVLGHRLIDVATVELVRAY

DIELEQTAAGWMVARLDTRRPPRLFGLIKHSGGHASRDWKAFEPLIGHARSDAVRRLSDR

FGELKAAEIADLLEEADKAEGGEILDRVHSDPELEADVFEELDPEKASRLLDEMPDDEVA

ALLGRMRADDAADAIVDLRQSRRRRVLDLMPAPQRTKVITLMGFNPESAGGLMNVDSVSC

AASATAAEALALIASSHSIQPEALIKVHVLDEDRRLDGVVSVITLLQVDPSETLERLMDS

DPVRVNADADLTDIALLMADFNLYSIPVVDEQDHLLGVVTVDDVLEATIPEDWRRREPAP

RPIREITTAEDRPLPGGNAP

>mava:LA64_09760 magnesium transporter MgtE (A)

MGTSEDKAPGQPVIHLSQLLRAPVLARSGETVGRVEDVIVRLRGADEYPLVTGIVAGVGG

RRVFVGDKSIHEYSADRVLLTKNKIDLRGFERREGEVLLRTDVLGHRLIDVATVELVRAY

DIELEQTAAGWMVARLDTRRPPRLFGLIKHSGGHASRDWKAFEPLIGHARSDAVRRLSDR

FGELKAAEIADLLEEADKAEGGEILDRVHSDPELEADVFEELDPEKASRLLDEMPDDEVA

ALLGRMRADDAADAIVDLRQSRRRRVLDLMPAPQRTKVITLMGFNPESAGGLMNVDSVSC

AASATAAEALALIASSHSIQPEALIKVHVLDEDRRLDGVVSVITLLQVDPSETLERLMDS

DPVRVNADADLTDIALLMADFNLYSIPVVDEQDHLLGVVTVDDVLEATIPEDWRRREPAP

RPIREITTAEDRPLPGGNAP

>mit:OCO_12800 hypothetical protein (A)

MGSVNRVYIARLARMLVLGPLGESVGRIRDVVISISIVRQQPRVLGLVVDLATRRSIFIP

ILRVASIEPNAVTLSTGNVSLRHFVQRPGEVLAIGQVLDTQVKVNDPELPELAGLDVVVT

DLGIEQNRTRDWMVSRVAVRTHRRLGRRGPVHVVDWQSVQGLTPSALALPGQGVAQLLGQ

FEGRKPVDVADAIRGLPAKRRYEVLRALNDDRLADILQELPEQDQAEVLSQLGTERSADV

LEEMDPDDAADLLGMLNPTDAEMLLTRMDPDESAPVRRLLTHSPDTAGGLMTSNPVVLTP

DTSVAEALARVRDPDLSTALSSMVFVARPPTATPTGRYLGCVHLQRLLREAPAELVGGIV

DSELLTLTPETPLVAVTRYLAAYNLVCGPVVDDQNHLLGAVTVDDLLDHLLPHDWRVDMQ

ELDTAGRFEGLGGSS

>mir:OCQ_12780 hypothetical protein (A)

MGSVNRVYIARLARMLVLGPLGESVGRIRDVVISISIVRQQPRVLGLVVDLATRRSIFIP

ILRVASIEPNAVTLSTGNVSLRHFVQRPGEVLAIGQVLDTQVKVNDPELPELAGLDVVVT

DLGIEQNRTRDWMVSRVAVRTHRRLGRRGPVHVVDWQSVQGLTPSALALPGQGVAQLLGQ

FEGRKPVDVADAIRGLPAKRRYEVLRALNDDRLADILQELPEQDQAEVLSQLGTERSADV

LEEMDPDDAADLLGMLNPTDAEMLLTRMDPDESAPVRRLLTHSPDTAGGLMTSNPVVLTP

DTSVAEALARVRDPDLSTALSSMVFVARPPTATPTGRYLGCVHLQRLLREAPAELVGGIV

DSELLTLTPETPLVAVTRYLAAYNLVCGPVVDDQNHLLGAVTVDDLLDHLLPHDWRVDMQ

ELDTAGRFEGLGGSS

>mia:OCU_12760 hypothetical protein (A)

MGSVNRVYIARLARMLVLGPLGESVGRIRDVVISISIVRQQPRVLGLVVDLATRRSIFIP

ILRVASIEPNAVTLSTGNVSLRHFVQRPGEVLAIGQVLDTQVKVNDPELPELAGLDVVVT

DLGIEQNRTRDWMVSRVAVRTHRRLGRRGPVHVVDWQSVQGLTPSALALPGQGVAQLLGQ

FEGRKPVDVADAIRGLPAKRRYEVLRALNDDRLADILQELPEQDQAEVLSQLGTERSADV

LEEMDPDDAADLLGMLNPTDAEMLLTRMDPDESAPVRRLLTHSPDTAGGLMTSNPVVLTP

DTSVAEALARVRDPDLSTALSSMVFVARPPTATPTGRYLGCVHLQRLLREAPAELVGGIV

DSELLTLTPETPLVAVTRYLAAYNLVCGPVVDDQNHLLGAVTVDDLLDHLLPHDWRVDMQ

ELDTAGRFEGLGGSS

>mie:LG41_06175 magnesium transporter (A)

MGSVNRVYIARLARMLVLGPLGESVGRIRDVVISISIVRQQPRVLGLVVDLATRRSIFIP

ILRVASIEPNAVTLSTGNVSLRHFVQRPGEVLAIGQVLDTQVKVNDPELPELAGLDVVVT

DLGIEQNRTRDWMVSRVAVRTHRRLGRRGPVHVVDWQSVQGLTPSALALPGQGVAQLLGQ

FEGRKPVDVADAIRGLPAKRRYEVLRALNDDRLADILQELPEQDQAEVLSQLGTERSADV

LEEMDPDDAADLLGMLNPTDAEMLLTRMDPDESAPVRRLLTHSPDTAGGLMTSNPVVLTP

DTSVAEALARVRDPDLSTALSSMVFVARPPTATPTGRYLGCVHLQRLLREAPAELVGGIV

DSELLTLTPETPLVAVTRYLAAYNLVCGPVVDDQNHLLGAVTVDDLLDHLLPHDWRVDMQ

ELDTAGRFEGLGGSS

>mid:MIP_02029 Magnesium transporter mgtE (A)

MGSVNRVYIARLARMLVLGPLGESVGRIRDVVISISIVRQQPRVLGLVVDLATRRSIFIP

ILRVASIEPNAVTLSTGNVSLRHFVQRPGEVLAIGQVLDTQVKVNDPELPELAGLDVVVT

DLGIEQNRTRDWMVSRVAVRTHRRLGRRGPVHVVDWQSVQGLTPSALALPGQGVAQLLGQ

FEGRKPVDVADAIRGLPAKRRYEVLRALNDDRLADILQELPEQDQAEVLSQLGTERSADV

LEEMDPDDAADLLGMLNPTDAEMLLTRMDPDESAPVRRLLTHSPDTAGGLMTSNPVVLTP

DTSVAEALARVRDPDLSTALSSMVFVARPPTATPTGRYLGCVHLQRLLREAPAELVGGIV

DSELLTLTPETPLVAVTRYLAAYNLVCGPVVDDQNHLLGAVTVDDLLDHLLPHDWRVDMQ

ELDTAGRFEGLGGSS

>myo:OEM_12940 hypothetical protein (A)

MGSVNRVYIARLARMLVLGPLGESVGRIRDVVVSISIVRQQPRVLGLVVDLATRRSIFIP

ILRVASIEPNAVTLSTGNVSLRHFVQRPGEVLAIGQVLDTQVKVNDPELPELAGLDVVVT

DLGIEQNRTRDWMVSRVAVRTHRRLGRRGPVHVVAWQSVQGLTPSALALPGQGVAQLLGQ

FEGRKPVDVADAIRGLPAKRRYEVLRALNDDRLADILQELPEQDQAEVLSQLGTERSADV

LEEMDPDDAADLLGMLNPTDAEMLLTRMDPDESAPVRRLLTHSPDTAGGLMTSNPVVLTP

DTSVAEALARVRDPDLSTALSSMVFVARPPTATPTGRYLGCVHLQRLLREAPAELVGGIV

DSELLTLTPETPLVAVTRYLAAYNLVCGPVVDDQNHLLGAVTVDDLLDHLLPHDWRVDMQ

ELDTAGRFEGLGGSS

>msm:MSMEG_6269 mgtE; magnesium transporter; K06213 magnesium transporter (A)

MTTRDATIDLRQTVASNTPKAVELWLEVTTDSDERERQLAALSPAERRGLGDLLDANTGA

ELLCSVEIDLAARLLKSMSDSAAARVLQLLNSPDAADILRELDEHRREAVLGAMPIERAR

ALLDVLAWPEDSVAARMHTDTPSVVPSATIAEAVDQIRDYAAAHPDGAVGASVCVVDADN

TLRGAVRLRELVLAQPQVAIGTLMRDVPVTVTPLTDIEEAAKTLIEHKLDELPVVDAEGR

LLGILVEDDAIEAVEREATEDAERQGGSEPLDVPYLRASPWLLWRKRIVWLLVLFAAEAY

TGTVLRAFEDEMEAVVALAFFIPLLIGTGGNTGTQITTTLVRAMGTGQIRFRDLPAIVSK

EMSTGFLIAVAMAAAALIRAWTLGVGPEVTLTVCLTVAAIVLWASLVSSVLPLVLRKLRV

DPAVVSAPMIATVVDGTGLMIYFWIAHLTLPQLAGL

>msg:MSMEI_6107 mgtE; Mg2+ transporter mgtE; K06213 magnesium transporter (A)

MTTRDATIDLRQTVASNTPKAVELWLEVTTDSDERERQLAALSPAERRGLGDLLDANTGA

ELLCSVEIDLAARLLKSMSDSAAARVLQLLNSPDAADILRELDEHRREAVLGAMPIERAR

ALLDVLAWPEDSVAARMHTDTPSVVPSATIAEAVDQIRDYAAAHPDGAVGASVCVVDADN

TLRGAVRLRELVLAQPQVAIGTLMRDVPVTVTPLTDIEEAAKTLIEHKLDELPVVDAEGR

LLGILVEDDAIEAVEREATEDAERQGGSEPLDVPYLRASPWLLWRKRIVWLLVLFAAEAY

TGTVLRAFEDEMEAVVALAFFIPLLIGTGGNTGTQITTTLVRAMGTGQIRFRDLPAIVSK

EMSTGFLIAVAMAAAALIRAWTLGVGPEVTLTVCLTVAAIVLWASLVSSVLPLVLRKLRV

DPAVVSAPMIATVVDGTGLMIYFWIAHLTLPQLAGL

>msb:LJ00_31000 magnesium transporter; K06213 magnesium transporter (A)

MTTRDATIDLRQTVASNTPKAVELWLEVTTDSDERERQLAALSPAERRGLGDLLDANTGA

ELLCSVEIDLAARLLKSMSDSAAARVLQLLNSPDAADILRELDEHRREAVLGAMPIERAR

ALLDVLAWPEDSVAARMHTDTPSVVPSATIAEAVDQIRDYAAAHPDGAVGASVCVVDADN

TLRGAVRLRELVLAQPQVAIGTLMRDVPVTVTPLTDIEEAAKTLIEHKLDELPVVDAEGR

LLGILVEDDAIEAVEREATEDAERQGGSEPLDVPYLRASPWLLWRKRIVWLLVLFAAEAY

TGTVLRAFEDEMEAVVALAFFIPLLIGTGGNTGTQITTTLVRAMGTGQIRFRDLPAIVSK

EMSTGFLIAVAMAAAALIRAWTLGVGPEVTLTVCLTVAAIVLWASLVSSVLPLVLRKLRV

DPAVVSAPMIATVVDGTGLMIYFWIAHLTLPQLAGL

>msn:LI99_31005 magnesium transporter; K06213 magnesium transporter (A)

MTTRDATIDLRQTVASNTPKAVELWLEVTTDSDERERQLAALSPAERRGLGDLLDANTGA

ELLCSVEIDLAARLLKSMSDSAAARVLQLLNSPDAADILRELDEHRREAVLGAMPIERAR

ALLDVLAWPEDSVAARMHTDTPSVVPSATIAEAVDQIRDYAAAHPDGAVGASVCVVDADN

TLRGAVRLRELVLAQPQVAIGTLMRDVPVTVTPLTDIEEAAKTLIEHKLDELPVVDAEGR

LLGILVEDDAIEAVEREATEDAERQGGSEPLDVPYLRASPWLLWRKRIVWLLVLFAAEAY

TGTVLRAFEDEMEAVVALAFFIPLLIGTGGNTGTQITTTLVRAMGTGQIRFRDLPAIVSK

EMSTGFLIAVAMAAAALIRAWTLGVGPEVTLTVCLTVAAIVLWASLVSSVLPLVLRKLRV

DPAVVSAPMIATVVDGTGLMIYFWIAHLTLPQLAGL

>msh:LI98_31010 magnesium transporter; K06213 magnesium transporter (A)

MTTRDATIDLRQTVASNTPKAVELWLEVTTDSDERERQLAALSPAERRGLGDLLDANTGA

ELLCSVEIDLAARLLKSMSDSAAARVLQLLNSPDAADILRELDEHRREAVLGAMPIERAR

ALLDVLAWPEDSVAARMHTDTPSVVPSATIAEAVDQIRDYAAAHPDGAVGASVCVVDADN

TLRGAVRLRELVLAQPQVAIGTLMRDVPVTVTPLTDIEEAAKTLIEHKLDELPVVDAEGR

LLGILVEDDAIEAVEREATEDAERQGGSEPLDVPYLRASPWLLWRKRIVWLLVLFAAEAY

TGTVLRAFEDEMEAVVALAFFIPLLIGTGGNTGTQITTTLVRAMGTGQIRFRDLPAIVSK

EMSTGFLIAVAMAAAALIRAWTLGVGPEVTLTVCLTVAAIVLWASLVSSVLPLVLRKLRV

DPAVVSAPMIATVVDGTGLMIYFWIAHLTLPQLAGL

>msa:Mycsm_04853 Mg/Co/Ni transporter MgtE with CBS domain (A)

MAAVNRVYAARLAGMVVLGPDGESIGRVRDVVISIGVARHQPRVLGLVVELLTRRRIFVP

ILRVTAIEPSAVTLATGNVSLRRFSQRPNEVLVLGEVIETRVRVDDPDLPELAGIDVDVV

DLGIEQSRTRDWVVTKVAVRPQRRLGRRSNVSIVDWNRVQGLTPSGLAMPDQGVGQLLEQ

FEGQRPIEVAEAIRELPEKRRFEVVRALDDERLADVLQELPEDEQAEVLQHLETDRAADV

LEAMDPDDAADLLGTMTPADAEQFLRRMDPEDSEDVRRLLSHSPDTAGGLMTSEPVVLAP

DTTVAEALARVRDPDLTPAVASLVFVTRAPTATPTGHYLGCVHLQRLLREPPAELVSGIL

DKDLPSLSPEDPLAAVTRYFAAYNLVCGPVVDEENHLLGAVSVDDVLDHLLPDDWREREE

PELSGALPDVAGQAAPRKL

>mul:MUL_0900 guaB2; inosine-5'-monophosphate (imp) dehydrogenase, GuaB2; K00088 IMP dehydrogenase [EC:1.1.1.205] (A)

MSRGMSHLEDSSDLVDTPYMRDARVGDLSGDSVPTGGDDPHKVAMLGLTFDDVLLLPAAS

DVVPSTADTSSQLTKKIRLKVPLVSSAMDTVTESRMAIAMARAGGMGVLHRNLPVAEQAS

QVETVKRSEAGMVTDPVTCRPDNTLAQVDALCARFRISGLPVVDDAGALVGIITNRDMRF

EVDQSKQVAEVMTKAPLITAQEGVSASAALGLLRRHKIEKLPVVDGSGRLTGLITVKDFV

KTEQHPLATKDSDGRLLVGAAVGVGGDAWVRAMMLVDAGVDVLIVDTAHAHNRLVLDMVS

KLKLEVGERVEVVGGNVATRSAAAALVDAGADAVKVGVGPGSICTTRVVAGVGAPQITAI

LEAVAVCRRAGIPVIADGGLQYSGDIAKALAAGASTAMLGSLLAGTAEAPGELIFVNGKQ

YKSYRGMGSLGAMQGRGGAKSYSKDRYFADDALSEDKLVPEGIEGRVPFRGPLASVIHQL

TGGLRAAMGYTGSPTIEVLQQAQFVRITSAGLKESHPHDVAMTVEAPNYYAR

>mva:Mvan_0051 magnesium transporter; K06213 magnesium transporter (A)

MSSPTSAAVRLTDILPTAGPGEIQAWLRSVAGPAERRHQVSRLSRAELRRLGEVLDGRTA

EILLESLDDELAARAVTAMDAAVAATLLAGLDTDHATDILREMRAPARDSVLSAMPADRS

EALRRVLEWPRESAAAHMIPEALAITSELTVADAVEQLRRDAVELRVDAHTSAYIYVTDR

DRRLLGVAAFRDLVLADPGRHVSELMNDDLLWVSPLTDAEEAAQALEDHNLVAVPVVDAD

MRLLGILTQSTAAEIAEEEATEDAERQGGSEPLDMPYLRASPWHLWRKRIGWLLLLFVAE

AYTGTVLRHFEEEMEAVVALAFFIPLLIGTGGNTGTQITTTLVRALATGDVRFRDVPSIV

AKELSTGMLIALTMALAAVIRAWTLGVGPEVTLCVSLTIGAIVLWSSFIASILPPLLKKC

RLDPALVSAPAIATIVDGTGLIIYFWIAHLTLAQLQGL

>mgi:Mflv_4176 magnesium transporter; K06213 magnesium transporter (A)

MADITAPPSETQLKSLLRALDDLDLPALTALLRPLSAIQVVDVLERLDVHERAVLYRILP

KDRALEVFEILPPSLQGDLVGALQDDAVAALFADMDPDDRVELLDELPATVAGRLMHGLP

PDERELTAVVLGYPQRSIGRRMSPEFVSVRPTMTTAEALTRVSAGLDDAETVYMLPVVDD

ERVLIGVVSLRRLLTAVPGTTIVEVMRPPHWARATEDAETAARRCADLRVLALPIVDNET

RLVGILTVDDALQILETAESEDQARIAGTEPLRRPYLTAPVVSLVRSRVVWLLVLAIGAT

LTVQVLEVFEATLSEVVTLALFVPLLIGTGGNTGNQAATTVTRALALGDVGPRDLGRVLL

RELRVGLSLGLLLGGLAFAVTSLVYDRSIGTVIGLTLVSLCTMAATVGGAMPLIARAIRV

DPAVFSNPFISTFVDATGLLIYFTIARAVLGI

>msp:Mspyr1_35190 Mg2+ transporter MgtE; K06213 magnesium transporter (A)

MADITAPPSETQLKSLLRALDDLDLPALTALLRPLSAIQVVDVLERLDVHERAVLYRILP

KDRALEVFEILPPSLQGDLVGALQDDAVAALFADMDPDDRVELLDELPATVAGRLMHGLP

PDERELTAVVLGYPQRSIGRRMSPEFVSVRPTMTTAEALTRVSAGLDDAETVYMLPVVDD

ERVLIGVVSLRRLLTAVPGTTIVEVMRPPHWARATEDAETAARRCADLRVLALPIVDNET

RLVGILTVDDALQILETAESEDQARIAGTEPLRRPYLTAPVVSLVRSRVVWLLVLAIGAT

LTVQVLEVFEATLSEVVTLALFVPLLIGTGGNTGNQAATTVTRALALGDVGPRDLGRVLL

RELRVGLSLGLLLGGLAFAVTSLVYDRSIGTVIGLTLVSLCTMAATVGGAMPLIARAIRV

DPAVFSNPFISTFVDATGLLIYFTIARAVLGI

>mab:MAB_1524c hypothetical protein (A)

MAAVSKVFAARLSGLVVLGPDGESIGRVRDVVIGMGVARKQPRVIGLVVEMLTRRRIFVP

MLRVTAIEPGSVTLNTGNVSLRRFEQRPSEALVLGQVLDTTVRTDDPELEQFHGVDLTVV

DLGLEQTRTRDWVVTRVAVRSPRRLGRRTGVQVTDWSHIQGLTPSTLNLPGQGVAQLMLQ

FEGMRPVEVADAIRELPPKRRDEVLGAFDDERLADILQELPEDDQAEVLTKLEDERAADV

LEAMDPDDAADLLGELPPAEAESLLALMDPEDSEPVRRLLTHSPNTAGGMMTPEPVILTP

NTTVAEALARVRDPDLTPALSSLVFVVRPPTATPTGRYLGCVHLQRLLREPPYAMVGGIL

DTDLPYLDAEAPLAEVTRYFAAYNLVCGPVIDREDHLLGAVTVDDVLDHLMPDGWRAEEP

EAVAGGDRP

>mabb:MASS_1517 hypothetical protein (A)

MAAVSKVFAARLSGLVVLGPDGESIGRVRDVVIGMGVARKQPRVIGLVVEMLTRRRIFVP

MLRVTAIEPGSVTLNTGNVSLRRFEQRPSEALVLGQVLDTTVRTDDPELEQFYGVDLTVV

DLGLEQTRTRDWVVTRVAVRSPRRLGRRTGVQVTDWSHIQGLTPSTLNLPGQGVAQLMLQ

FEGMRPVEVADAIRELPPKRRDEVLGAFDDERLADILQELPEDDQAEVLTKLKDERAADV

LEAMDPDDAADLLGELPPAEAESLLALMDPEDSEPVRRLLTHSPNTAGGMMTPEPVILTP

NTTVAEALARVRDPDLTPALSSLVFVVRPPTATPTGRYLGCVHLQRLLREPPYAMVGGIL

DTDLPYLDAEAPLAEVTRYFAAYNLVCGPVIDREDHLLGAVTVDDVLDHLMPDGWRAEEP

EAVAGGDRP

>mmv:MYCMA_0833 magnesium transporter (A)

MAAVSKVFAARLSGLVVLGPDGESIGRVRDVVIGMGVARKQPRVIGLVVEMLTRRRIFVP

MLRVTAIEPGSVTLNTGNVSLRRFEQRPSEALVLGQVLDTTVRTDDPELEQFYGVDLTVV

DLGLEQTRTRDWVVTRVAVRSPRRLGRRTGVQVTDWSHIQGLTPSTLNLPGQGVAQLMLQ

FEGMRPVEVADAIRELPPKRRDEVLGAFDDERLADILQELPEDDQAEVLTKLEDERAADV

LEAMDPDDAADLLGELPPAEAESLLALMDPEDSEPVRRLLTHSPNTAGGMMTPEPVILTP

NTTVAEALARVRDPDLTPALSSLVFVVRPPTATPTGRYLGCVHLQRLLREPPYAMVGGIL

DTDLPYLDAEAPLAEVTRYFAAYNLVCGPVIDREDHLLGAVTVDDVLDHLMPDGWRAEEP

EAVAGGDRP

>may:LA62_07745 magnesium transporter (A)

MAAVSKVFAARLSGLVVLGPDGESIGRVRDVVIGMGVARKQPRVIGLVVEMLTRRRIFVP

MLRVTAIEPGSVTLNTGNVSLRRFEQRPSEALVLGQVLDTTVRTDDPELEQFHGVDLTVV

DLGLEQTRTRDWVVTRVAVRSPRRLGRRTGVQVTDWSHIQGLTPSTLNLPGQGVAQLMLQ

FEGMRPVEVADAIRELPPKRRDEVLGAFDDERLADILQELPEDDQAEVLTKLEDERAADV

LEAMDPDDAADLLGELPPAEAESLLALMDPEDSEPVRRLLTHSPNTAGGMMTPEPVILTP

NTTVAEALARVRDPDLTPALSSLVFVVRPPTATPTGRYLGCVHLQRLLREPPYAMVGGIL

DTDLPYLDAEAPLAEVTRYFAAYNLVCGPVIDREDHLLGAVTVDDVLDHLMPDGWRAEEP

EAVAGGDRP

>mabo:NF82_07705 magnesium transporter (A)

MAAVSKVFAARLSGLVVLGPDGESIGRVRDVVIGMGVARKQPRVIGLVVEMLTRRRIFVP

MLRVTAIEPGSVTLNTGNVSLRRFEQRPSEALVLGQVLDTTVRTDDPELEQFHGVDLTVV

DLGLEQTRTRDWVVTRVAVRSPRRLGRRTGVQVTDWSHIQGLTPSTLNLPGQGVAQLMLQ

FEGMRPVEVADAIRELPPKRRDEVLGAFDDERLADILQELPEDDQAEVLTKLEDERAADV

LEAMDPDDAADLLGELPPAEAESLLALMDPEDSEPVRRLLTHSPNTAGGMMTPEPVILTP

NTTVAEALARVRDPDLTPALSSLVFVVRPPTATPTGRYLGCVHLQRLLREPPYAMVGGIL

DTDLPYLDAEAPLAEVTRYFAAYNLVCGPVIDREDHLLGAVTVDDVLDHLMPDGWRAEEP

EAVAGGDRP

>mabl:MMASJCM_1543 Mg Co Ni transporter (A)

MSKVFAARLSGLVVLGPDGESIGRVRDVVIGMGVARKQPRVIGLVVEMLTRRRIFVPMLR

VTAIEPGSVTLNTGNVSLRRFEQRPSEALVLGQVLDTTVRTDDPELEQFYGVDLTVVDLG

LEQTRTRDWVVTRVAVRSPRRLGRRTGVQVTDWSHIQGLTPSTLNLPGQGVAQLMLQFEG

MRPVEVADAIRELPPKRRDEVLGAFDDERLADILQELPEDDQAEVLTKLKDERAADVLEA

MDPDDAADLLGELPPAEAESLLALMDPEDSEPVRRLLTHSPNTAGGMMTPEPVILTPNTT

VAEALARVRDPDLTPALSSLVFVVRPPTATPTGRYLGCVHLQRLLREPPYAMVGGILDTD

LPYLDAEAPLAEVTRYFAAYNLVCGPVIDREDHLLGAVTVDDVLDHLMPDGWRAEEPEAV

AGGDRP

>maz:LA61_07640 magnesium transporter (A)

MAAVSKVFAARLSGLVVLGPDGESIGRVRDVVIGMGVARKQPRVIGLVVEMLTRRRIFVP

MLRVTAIEPGSVTLNTGNVSLRRFEQRPSEALVLGQVLDTTVRTDDPELEQFHGVDLTVV

DLGLEQTRTRDWVVTRVAVRSPRRLGRRTGVQVTDWSHIQGLTPSTLNLPGQGVAQLMLQ

FEGMRPVEVADAIRELPPKRRDEVLGAFDDERLADILQELPEDDQAEVLTKLEDERAADV

LEAMDPDDAADLLGELPPAEAESLLALMDPEDSEPVRRLLTHSPNTAGGMMTPEPVILTP

NTTVAEALARVRDPDLTPALSSLVFVVRPPTATPTGRYLGCVHLQRLLREPPYAMVGGIL

DTDLPYLDAEAPLAEVTRYFAAYNLVCGPVIDREDHLLGAVTVDDVLDHLMPDGWRAEEP

EAVAGGDRP

>mak:LH56_15475 magnesium transporter (A)

MAAVSKVFAARLSGLVVLGPDGESIGRVRDVVIGMGVARKQPRVIGLVVEMLTRRRIFVP

MLRVTAIEPGSVTLNTGNVSLRRFEQRPSEALVLGQVLDTTVRTDDPELEQFYGVDLTVV

DLGLEQTRTRDWVVTRVAVRSPRRLGRRTGVQVTDWSHIQGLTPSTLNLPGQGVAQLMLQ

FEGMRPVEVADAIRELPPKRRDEVLGAFDDERLADILQELPEDDQAEVLTKLKDERAADV

LEAMDPDDAADLLGELPPAEAESLLALMDPEDSEPVRRLLTHSPNTAGGMMTPEPVILTP

NTTVAEALARVRDPDLTPALSSLVFVVRPPTATPTGRYLGCVHLQRLLREPPYAMVGGIL

DTDLPYLDAEAPLAEVTRYFAAYNLVCGPVIDREDHLLGAVTVDDVLDHLMPDGWRAEEP

EAVAGGDRP

>mys:NF92_15965 magnesium transporter (A)

MAAVSKVFAARLSGLVVLGPDGESIGRVRDVVIGMGVARKQPRVIGLVVEMLTRRRIFVP

MLRVTAIEPGSVTLNTGNVSLRRFEQRPSEALVLGQVLDTTVRTDDPELEQFYGVDLTVV

DLGLEQTRTRDWVVTRVAVRSPRRLGRRTGVQVTDWSHIQGLTPSTLNLPGQGVAQLMLQ

FEGMRPVEVADAIRELPPKRRDEVLGAFDDERLADILQELPEDDQAEVLTKLEDERAADV

LEAMDPDDAADLLGELPPAEAESLLALMDPEDSEPVRRLLTHSPNTAGGMMTPEPVILTP

NTTVAEALARVRDPDLTPALSSLVFVVRPPTATPTGRYLGCVHLQRLLREPPYAMVGGIL

DTDLPYLDAEAPLAEVTRYFAAYNLVCGPVIDREDHLLGAVTVDDVLDHLMPDGWRAEEP

EAVAGGDRP

>myc:NF90_15970 magnesium transporter (A)

MAAVSKVFAARLSGLVVLGPDGESIGRVRDVVIGMGVARKQPRVIGLVVEMLTRRRIFVP

MLRVTAIEPGSVTLNTGNVSLRRFEQRPSEALVLGQVLDTTVRTDDPELEQFYGVDLTVV

DLGLEQTRTRDWVVTRVAVRSPRRLGRRTGVQVTDWSHIQGLTPSTLNLPGQGVAQLMLQ

FEGMRPVEVADAIRELPPKRRDEVLGAFDDERLADILQELPEDDQAEVLTKLEDERAADV

LEAMDPDDAADLLGELPPAEAESLLALMDPEDSEPVRRLLTHSPNTAGGMMTPEPVILTP

NTTVAEALARVRDPDLTPALSSLVFVVRPPTATPTGRYLGCVHLQRLLREPPYAMVGGIL

DTDLPYLDAEAPLAEVTRYFAAYNLVCGPVIDREDHLLGAVTVDDVLDHLMPDGWRAEEP

EAVAGGDRP

>mmc:Mmcs_1103 CBS domain containing protein (A)

MERLRAPATARPADAELLGPPDRALHHASLRIPVGPPDATVADTLNAMRGKHFDSAASVA

VIDGDRLAGIVTIERMFGAHEGALLRDVMDPDPPVVAPDTDQERAAWEAVHRAEPGLAVV

DENGRFRGLISPHRLLTVLLAEHDEDLARLGGFLRGAATARSSSVEAVRRRLWHRLPWLV

LGLLGAMVSAVLMSAFEVQLDANLAVAYFIPGIVYLADAVGTQTETLAIRGLSVGVGIRR

ITGRETLTGLSVGVLLGALMWPVVAVMTSSMALASAVSVAVLGASAIATVVALFLPWLLQ

RLGMDPAFGAGPLSTVVQDLLTIVIYFGAVTVLVS

>mkm:Mkms_1120 CBS domain containing protein (A)

MERLRAPATARPADAELLGPPDRALHHASLRIPVGPPDATVADTLNAMRGKHFDSAASVA

VIDGDRLAGIVTIERMFGAHEGALLRDVMDPDPPVVAPDTDQERAAWEAVHRAEPGLAVV

DENGRFRGLISPHRLLTVLLAEHDEDLARLGGFLRGAATARSSSVEAVRRRLWHRLPWLV

LGLLGAMVSAVLMSAFEVQLDANLAVAYFIPGIVYLADAVGTQTETLAIRGLSVGVGIRR

ITGRETLTGLSVGVLLGALMWPVVAVMTSSMALASAVSVAVLGASAIATVVALFLPWLLQ

RLGMDPAFGAGPLSTVVQDLLTIVIYFGAVTVLVS

>mjl:Mjls_1132 CBS domain containing protein (A)

MERLRAPAAARPADPELLGPPDRALHHASLRIPVAPPDASVADTLNAMRGKHFDSAASVA

VIEGDRLAGIVTIERMFGAHEGALLRDVMDPDPPVVAPDTDQERAAWEAVHRAEPGLAVV

DENGRFRGLISPHRLLTVLLAEHDEDLARLGGFLRGAATARSSSVEAVRRRLWHRLPWLV

LGLLGAMVSAVLMSAFEVQLDANLAVAYFIPGIVYLADAVGTQTETLAIRGLSVGVGIRR

ITGRETLTGLSVGVLLGALMWPVVAVMTSSMALASAVSVAVLGASAIATVVALFLPWLLQ

RLGMDPAFGAGPLSTVVQDLLTIVIYFGAVTVLVS

>mjd:JDM601_1202 transport transmembrane protein (A)

MLVLGPFGESVGRVRDVVVSISIVRQQPRVLGLVVELPTRRRIFVPILRVAAIEPNAVTL

NTGTVSLRKFTQRPGEVLVLGQVLDTKVRVNDPELPELAGVDVAITDLAIEQSRTRDWLV

TRIAVRHSRRLGRRAAVHIVDWHNVQGLTPSALAMPGQGVAQLLQQFEGWRPIEVADAIR

ELPPKRRFEVINALDNGRLADILQELPEERQAAVLGQLGTERAADVLEEMDPDDAADLLG

AMNPNDAELLLARMDPDDSDPVRRLLEHSPDTAGGLMTSNPVVLTADTSVAEALARVRDP

DLTPALSSLVFVTRPPTATPTGHYLGCVPLQRLLRVPPADLVGGIVDSDLPALNAEVSLA

ELTRYFAAYNLVCGPVLDEQNHLLGAVTVDDVLDHLLPHDWRETPEPDLGPKFAGRPS

>mmi:MMAR_0670 mgtE; Mg2+ transport transmembrane protein MgtE; K06213 magnesium transporter (A)

MSIHYPTVEQSTIDIRQVVGIGTPKAVDLWLDVVTDAPDRVRELASLSKAELAKLGDLLD

TTSGVELFESVDDMLAAEALQAMDSAVAASLLDALDSDHAANILREFKTAKRDALLASLP

LKRAVVLRGLLSWPEDSAAAHMVPETLTVGANMTVLDAIATVREHAAGLRSDSRTTAYVY

VIDADSHLLGVVAFRALVLADPERLVSELMTEDLIVVSPLTDKELAAQTLMTHNLMAVPV

VDGENRLLGIIAEDEALDITQEEATEDAERQGGSAPLEVPYLRASPWLLWRKRVVWLLVL

FVAEAYTGSVLRAFSDEMEAVIALAFFIPLLIGTGGNTGTQIATTLVRAMATGQVRFRDV

PAVLVKELSTGALVGFTMAVAAVIRAWTLNVGPQVTVTVAVTVAAIVVWSSLVAAILPPL

LKKLRIDPAIVSGPLIATIVDGTGLIIYFMIAHLTLTQLQGL

>mrh:MycrhN_3701 Mg/Co/Ni transporter MgtE with CBS domain (A)

MAAVNRVYAARLVGMVVLGPDGESLGRVRDVVISISIVRQQPRVLGLVVELLTRRRIFVP

ILRVTAIDPGAVTLATGSVSLRKFTQRPGEVLVLGQVVETVVRVDDPDLPQLAGIDVYVV

DLGIEQTRTRDWMVTKVAVRPPRRLARRSNVHIVDWQHVQGLTPSGLAMPDQGVAQLLEQ

FAGQRAVEVAEAIRELPDKRRYEVVNALDDERLADVLQELSEDQQAAMLKQLPAERAADV

LEAMDPDDAADLLGAMTPLDAEQFLRRMDPEDSEDVRRLLAHSPDTAGGLMTSEPVVLAP

DTTVAEALARVRDPDLTPALASLVFVTRAPTATPTGHYLGCVHLQRLLREPPADLVSGIV

DKDLPNLGPEDSLAAVTRYFAAYNLVCGAVVDEQNHLLGAVSVDDVLDHLLPHDWRDREE

PELFTTSEGAS

>mmm:W7S_06235 hypothetical protein (A)

MGSVNRVYIARLARMLVLGPLGESVGRIRDVVISISIVRQQPRVLGLVVDLATRRSIFIP

ILRVASIEPNAVTLSTGNVSLRHFVQRPGEVLAIGQVLDTQVKVNDPELPELAGLDVVVT

DLGIEQNRTRDWMVSRVAVRTHRRLGRRGPVHVVAWQSVQGLTPSALALPGQGVAQLLGQ

FEGRKPVDVADAIRGLPAKRRYEVLRALNDDRLADILQELPEQDQAEVLSQLGTERSADV

LEEMDPDDAADLLGMLNPTDAEMLLTRMDPDESAPVRRLLTHSPDTAGGLMTSNPVVLTP

DTSVAEALARVRDPDLSTALSSMVFVARPPTATPTGRYLGCVHLQRLLREAPAELVGGIV

DSELLTLTPETPLVAVTRYLAAYNLVCGPVVDDQNHLLGAVTVDDLLDHLLPHDWRVDMQ

ELDTAGRFEGLGGSS

>mcb:Mycch_3891 Mg/Co/Ni transporter MgtE with CBS domain (A)

MASVNRVYAARLAGMVVLGPDGESIGRVRDIVIGITIVRQQPRVLGLVIELLSRRRIFVP

ILRVTAIEPGAVTLSTGNVSLRRFSQRPGEVLVLGQVVETRVRVDDPDLSQLAGIDVVVV

DLGIEQTRSRDWMVTKVAVRPQRRLGRRSNVYTVDWQHVQGLTPSGLAMPDQGVAQLLEQ

FEGQRPIEVADAIRELPAKRRYEVFNALDDERLADVLQELPEDEQVTVLRQLKTDRAADV

LEAMDPDDAADLLGTMTPADAEQFLRRMDPEDSEDVRRLLSHSPNTAGGLMTSEPVVLAP

DTTVAEALARVRDPDLTPALASLVFVVRPPTATPTGRYLGCVHLQRLLREPPAALVSGIV

DTDLPSLRPEDSLGALTRYFAAYNLVCGPVVDEESHLLGAVSVDDVLDHLLPDDWREREA

EPEIVTAERTT

>mli:MULP_00663 mgtE; Mg2 transport transmembrane protein MgtE; K06213 magnesium transporter (A)

MVTDAPDRVRELASLSKAELAKLGDLLDTTSGVELFESVDDMLAAEALQAMDSAVAASLL

DALDSDHAANILREFKTAKRDALLASLPLKRAVVLRGLLSWPEDSAAAHMVPEALTVGAN

MTVLDAIATVREHAAGLRSDSRTTAYVYVIDADSHLLGVVAFRALVLADPERLVSELMTE

DLIVVSPLTDKELAAQTLMTHNLMAVPVVDGENRLLGIIAEDEALDITQEEATEDAERQG

GSAPLEVPYLRASPWLLWRKRVVWLLVLFVAEAYTGSVLRAFSDEMEAVIALAFFIPLLI

GTGGNTGTQIATTLVRAMATGQVRFRDVPAPDVVDDGGRDGRPQRRNQWIARRRHGRGSA

RAGGQQQHGRTRQHQDAERGGDAARNVAGRSCRLLGGQRHPLHGEKEPNRKGQRRPHAQI

AVRQKSGCADRVGRGDIHQICQSKVANRGDCENDEAYQCHTGDDEHYLQCFTHTRQMNAD

KECINGQVNPPSIANAEQAQRLYVCADEGGDRRRCD

>mkn:MKAN_06905 magnesium transporter (A)

MGSVNRVYVARLSRMMVLGPLGESFGRVRDVVISISIVRQQPRVLGLVVDLATRRSIFIP

ILRVAAIEPDAVTLATGSVSLRHFEQRPGEVLAIGQVLDTPVKVNDPALPELASADLVVT

DLGIEQTRTRDWLVTRVAVRTHRRLGRRGPVHIVDWQFVHGLTPSALAMPGQAVAQLLDQ

FEGRRPVDVADAIRGLPSKRRYEVFRALDNERLADILQELPESDQAEVLSQLGTDRAADV

LEEMDPDDAADLLGVLNPNDAEALLTRMDPDDSNSVRRLLKHSPDTAGGLMTSNPVVLTP

DTSVAEALARVRDPDLTPALSSMVFVARPPTATPTGHYLGCVQLQRLLREPPAELVGGIV

DTDLLTLTPETPLAAVTRYFAAYNLVCGPVVDDQSHLLGAVTVDDLLDHLLPHDWRVDVQ

QLDPAGRPAKSGGTR

>mks:LG40_06835 magnesium transporter (A)

MGSVNRVYVARLSRMMVLGPLGESFGRVRDVVISISIVRQQPRVLGLVVDLATRRSIFIP

ILRVAAIEPDAVTLATGSVSLRHFEQRPGEVLAIGQVLDTPVKVNDPALPELASADLVVT

DLGIEQTRTRDWLVTRVAVRTHRRLGRRGPVHIVDWQFVHGLTPSALAMPGQAVAQLLDQ

FEGRRPVDVADAIRGLPSKRRYEVFRALDNERLADILQELPESDQAEVLSQLGTDRAADV

LEEMDPDDAADLLGVLNPNDAEALLTRMDPDDSNSVRRLLKHSPDTAGGLMTSNPVVLTP

DTSVAEALARVRDPDLTPALSSMVFVARPPTATPTGHYLGCVQLQRLLREPPAELVGGIV

DTDLLTLTPETPLAAVTRYFAAYNLVCGPVVDDQSHLLGAVTVDDLLDHLLPHDWRVDVQ

QLDPAGRPAKSGGTR

>mki:LH54_06845 magnesium transporter (A)

MGSVNRVYVARLSRMMVLGPLGESFGRVRDVVISISIVRQQPRVLGLVVDLATRRSIFIP

ILRVAAIEPDAVTLATGSVSLRHFEQRPGEVLAIGQVLDTPVKVNDPALPELASADLVVT

DLGIEQTRTRDWLVTRVAVRTHRRLGRRGPVHIVDWQFVHGLTPSALAMPGQAVAQLLDQ

FEGRRPVDVADAIRGLPSKRRYEVFRALDNERLADILQELPESDQAEVLSQLGTDRAADV

LEEMDPDDAADLLGVLNPNDAEALLTRMDPDDSNSVRRLLKHSPDTAGGLMTSNPVVLTP

DTSVAEALARVRDPDLTPALSSMVFVARPPTATPTGHYLGCVQLQRLLREPPAELVGGIV

DTDLLTLTPETPLAAVTRYFAAYNLVCGPVVDDQSHLLGAVTVDDLLDHLLPHDWRVDVQ

QLDPAGRPAKSGGTR

>mne:D174_19545 magnesium transporter; K06213 magnesium transporter (A)

MGSTTATALRSAIAKRDVAAWLRSFKDPAERSHQLAKCSADDLRALGPLLDSDGATELFE

SIDDELTAYVLAAMAAPDAASLVDALDLDHAADVLRDMKDTPREQVLSVLPQIRADVLRG

LLSWPPDSVAAHMVPEALTVRPDMTVGEAVASIRGDAATLRSDSRTGAYVYVTDDDNRLL

GVVAFRDLVLCDAQDTVAALTNSDVLSVSPLTDAEEAAQALVDYNLVAVPVVDADNRLLG

ILTENTANDITAEEATEDAERQGGSEPLEVPYLRASPWLLWRKRIGWLLLLFIAEAYTGT

VLRHFEEEMEAVVALAFFIPLLIGTGGNTGTQITTTLVRAMATGQVRLRDVPAILAKEMS

TGMMIALTMALAALIRAWMLGVGPEITLTVCLTVAAIVLWSSFVASILPPVLKKCRVDPA

VVSAPMIATIVDGTGLIIYFMIAHATLSQLQGL

>myv:G155_27920 magnesium transporter; K06213 magnesium transporter (A)

MSNTRSGTATINLREAVAMDTAKAIELWLEMESEPTERERQIAALAAGDRGRLGELLDTK

TGPELMTTIDAHLAAKVLKATPAAAAAGVLSALDAPHAAEILRRFDDPRRDALLDAMVID

RARMLRTVLSWPEDSVAAHMQPDALTVAPAATVAQAVDQIREHAAHRPHGSAGAYVYVVD

PQGVLLGAVRLRALVLTPADRPVEELMEEAVTAAPLTDVEEAAMTLIDHQLDELPVVDAE

NRLLGVLTEDDAVQVAEQEATEDAERQGGSAPLEVPYLQASPWLLWRKRIVWLLVLFAAE

AYTGTVLRAFEEEMEAVVALAFFIPLLIGTGGNTGTQITTTLVRAMGTGQIRFRDVPAIV

TKEMSTGALIAVAMAAAALVRSWTLGVGPQVTLTVCLTVAAIVMWASLVSSVLPLLLKKV

KVDPAVVSAPMIATIVDGTGLMIYFWIAHLTLPQLAGL

>mye:AB431_23035 magnesium transporter (A)

MASVNRVYAARLAGLVVLGPDGESLGRVRDVVISMSIVRQQPRVLGLVVEMLTRRRIFVP

ILRVTAIEPNAVTLNTGNVSLRRFAQRPGEVLVLGQVLDSRVRVNDPELPQMSGVDVIVV

DLGIEQSRSRDWVVTRVAVRSHRRLGRRSTIQVVDWHNVHGLTPSALSMPGQGVAQLLHQ

FEGQRPIEVADAIRDLPAKRRTELINALDDERLADVLQELPEDDQTVLLLQLDTERAADV

LEAMDPDDAADLLGVLNPTEAEVLLRRMDPEDSEPVRRLLSHSPDTAGGLMTSNPVVLAP

DTTVAEALARVRDPDLTPALASLVFVVRPPTATPTGRYLGCVHLQALLREPPANLVSGIV

DTDLPSCTPNTSLAGVTRYFAAYNLVCGPVVDEENHLLGAVTVDDVLDHLLPDDWRESME

DPQLSDGVIGAEGTT

>mgo:AFA91_03555 magnesium transporter; K06213 magnesium transporter (A)

MSTTRDATIDLRQTVALNTPKAVELWLEVTTDSDERERQLATLSHAERRRLGDLLDANTG

AELLCSVELDLAARLLKSVSEASAARVLAVLNAPDAADILRELDEHRREAVLAAMPIERA

RALLDVLAWPQDSVAARMHTETPSVAPSATIAEAVDQIRDYAAAHPDETVGASVWVVDED

NTLRGAVRLRDLVLAQPQLPVGTLMRDVPVTVTPLTDIEEAAKTLIEHRLDELPVVDAER

RLLGVLVEDDAIEAVERKTTEDAERQGGSEPLDVPYLRASPWLLWRKRIVWLLALFVAEA

YTGTVLRAFEDEMEAVVALAFFIPLLIGTGGNTGTQITTTLVRAMGTGQIRFRDLPAIVS

KEVSTGFLIAVAMAVAALIRAWSLGVGPEVTLTVCLTVAAIVMWSSLVSSVLPLVLKKLR

VDPAVVSAPMIATVVDGTGLMIYFWIAHLTLPQLAGL

>mft:XA26_56420 Mg/Co/Ni transporter MgtE; K06213 magnesium transporter (A)

MSNTRSGTATINLREAVAMDTAKAIELWLEMESEPTERERQVAALAAGDRGRLGELLDTK

TGPELMTTIDAHLAAKVLKATPAAAAAGVLSALDAPHAAEILRRFDDPRRDALLDAMVID

RARMLRTVLSWPEDSVAAHMQPDALTVAPAATVAQAVDQIREHAAHRPHGSAGAYVYVVD

PQGVLLGAVRLRALVLTPADRPVEELMEEAVTAAPLTDVEDAAMTLIDHQLDELPVVDAE

NRLLGVFTEDDAVQVAEQEATEDAERQGGSAPLEVPYLQASPWLLWRKRIVWLLVLFAAE

AYTGTVLRAFEEEMEAVVALAFFIPLLIGTGGNTGTQITTTLVRAMGTGQIRFRDVPAIV

TKEMSTGALIAVAMAAAALVRSWTLGVGPQVTLTVCLTVAAIVMWASLVSSVLPLLLKKV

KVDPAVVSAPMIATIVDGTGLMIYFWIAHLTLPQLAGL

>mhad:B586_07750 magnesium transporter (A)

MGSVNKVYVARLARMLVLGPLGESFGRVRDVVISISIVRQQPRILGLVVDLATRRSIFIP

MLRVAAIEPDAVTLATSNVSLHHFGQRPGEVLALGQVLDTPVRVNDPALPELAGVDVVIT

DLGIEQTRTRDWMVTKIAVRAQRRLGRRGPVHVVDWQYVQGLTPSALAMPGKGVAQLLDQ

FDGRKAVDVADAIRGLPRARRYEVLKALHDERLADILQELPELYQADVLSQLGTERAADV

LEAMDPDDAADLLGVLSPTDAELLLTRMDPGDSGAVRRLLTYPPDTAGGLMTSDPVVLTP

DTAVAEALARVRDSDLSPALSSMVFVARPPTATPTGPYLGCVHLQRLLREAPAELVGGII

DTDLPTLTPETPLAAVTRYLAAYNLVCGPVLDDQNHLLGAVTVDDLLDHLLPHDWRVDVP

EFSRCGTAGIPGGS

Rv0391 Homologs

>mtu:Rv0391 metZ; O-succinylhomoserine sulfhydrylase; K10764 O-succinylhomoserine sulfhydrylase [EC:2.5.1.-] (A)

MTDESSVRTPKALPDGVSQATVGVRGGMLRSGFEETAEAMYLTSGYVYGSAAVAEKSFAG

ELDHYVYSRYGNPTVSVFEERLRLIEGAPAAFATASGMAAVFTSLGALLGAGDRLVAARS

LFGSCFVVCSEILPRWGVQTVFVDGDDLSQWERALSVPTQAVFFETPSNPMQSLVDIAAV

TELAHAAGAKVVLDNVFATPLLQQGFPLGVDVVVYSGTKHIDGQGRVLGGAILGDREYID

GPVQKLMRHTGPAMSAFNAWVLLKGLETLAIRVQHSNASAQRIAEFLNGHPSVRWVRYPY

LPSHPQYDLAKRQMSGGGTVVTFALDCPEDVAKQRAFEVLDKMRLIDISNNLGDAKSLVT

HPATTTHRAMGPEGRAAIGLGDGVVRISVGLEDTDDLIADIDRALS

>mtv:RVBD_0391 O-succinylhomoserine sulfhydrylase MetZ; K10764 O-succinylhomoserine sulfhydrylase [EC:2.5.1.-] (A)

MTDESSVRTPKALPDGVSQATVGVRGGMLRSGFEETAEAMYLTSGYVYGSAAVAEKSFAG

ELDHYVYSRYGNPTVSVFEERLRLIEGAPAAFATASGMAAVFTSLGALLGAGDRLVAARS

LFGSCFVVCSEILPRWGVQTVFVDGDDLSQWERALSVPTQAVFFETPSNPMQSLVDIAAV

TELAHAAGAKVVLDNVFATPLLQQGFPLGVDVVVYSGTKHIDGQGRVLGGAILGDREYID

GPVQKLMRHTGPAMSAFNAWVLLKGLETLAIRVQHSNASAQRIAEFLNGHPSVRWVRYPY

LPSHPQYDLAKRQMSGGGTVVTFALDCPEDVAKQRAFEVLDKMRLIDISNNLGDAKSLVT

HPATTTHRAMGPEGRAAIGLGDGVVRISVGLEDTDDLIADIDRALS

>mtc:MT0402 K10764 O-succinylhomoserine sulfhydrylase [EC:2.5.1.-] | (GenBank) metZ; O-succinylhomoserine sulfhydrylase (EC:4.2.99.-) (A)

MTDESSVRTPKALPDGVSQATVGVRGGMLRSGFEETAEAMYLTSGYVYGSAAVAEKSFAG

ELDHYVYSRYGNPTVSVFEERLRLIEGAPAAFATASGMAAVFTSLGALLGAGDRLVAARS

LFGSCFVVCSEILPRWGVQTVFVDGDDLSQWERALSVPTQAVFFETPSNPMQSLVDIAAV

TELAHAAGAKVVLDNVFATPLLQQGFPLGVDVVVYSGTKHIDGQGRVLGGAILGDREYID

GPVQKLMRHTGPAMSAFNAWVLLKGLETLAIRVQHSNASAQRIAEFLNGHPSVRWVRYPY

LPSHPQYDLAKRQMSGGGTVVTFALDCPEDVAKQRAFEVLDKMRLIDISNNLGDAKSLVT

HPATTTHRAMGPEGRAAIGLGDGVVRISVGLEDTDDLIADIDRALS

>mra:MRA_0398 K10764 O-succinylhomoserine sulfhydrylase [EC:2.5.1.-] | (GenBank) metZ; O-succinylhomoserine sulfhydrylase (A)

MTDESSVRTPKALPDGVSQATVGVRGGMLRSGFEETAEAMYLTSGYVYGSAAVAEKSFAG

ELDHYVYSRYGNPTVSVFEERLRLIEGAPAAFATASGMAAVFTSLGALLGAGDRLVAARS

LFGSCFVVCSEILPRWGVQTVFVDGDDLSQWERALSVPTQAVFFETPSNPMQSLVDIAAV

TELAHAAGAKVVLDNVFATPLLQQGFPLGVDVVVYSGTKHIDGQGRVLGGAILGDREYID

GPVQKLMRHTGPAMSAFNAWVLLKGLETLAIRVQHSNASAQRIAEFLNGHPSVRWVRYPY

LPSHPQYDLAKRQMSGGGTVVTFALDCPEDVAKQRAFEVLDKMRLIDISNNLGDAKSLVT

HPATTTHRAMGPEGRAAIGLGDGVVRISVGLEDTDDLIADIDRALS

>mtf:TBFG_10395 K10764 O-succinylhomoserine sulfhydrylase [EC:2.5.1.-] | (GenBank) O-succinylhomoserine sulfhydrylase metZ (A)

MTDESSVRTPKALPDGVSQATVGVRGGMLRSGFEETAEAMYLTSGYVYGSAAVAEKSFAG

ELDHYVYSRYGNPTVSVFEERLRLIEGAPAAFATASGMAAVFTSLGALLGAGDRLVAARS

LFGSCFVVCSEILPRWGVQTVFVDGDDLSQWERALSVPTQAVFFETPSNPMQSLVDIAAV

TELAHAAGAKVVLDNVFATPLLQQGFPLGVDVVVYSGTKHIDGQGRVLGGAILGDREYID

GPVQKLMRHTGPAMSAFNAWVLLKGLETLAIRVQHSNASAQRIAEFLNGHPSVRWVRYPY

LPSHPQYDLAKRQMSGGGTVVTFALDCPEDVAKQRAFEVLDKMRLIDISNNLGDAKSLVT

HPATTTHRAMGPEGRAAIGLGDGVVRISVGLEDTDDLIADIDRALS

>mtb:TBMG_00391 K10764 O-succinylhomoserine sulfhydrylase [EC:2.5.1.-] | (GenBank) O-succinylhomoserine sulfhydrylase metZ (A)

MTDESSVRTPKALPDGVSQATVGVRGGMLRSGFEETAEAMYLTSGYVYGSAAVAEKSFAG

ELDHYVYSRYGNPTVSVFEERLRLIEGAPAAFATASGMAAVFTSLGALLGAGDRLVAARS

LFGSCFVVCSEILPRWGVQTVFVDGDDLSQWERALSVPTQAVFFETPSNPMQSLVDIAAV

TELAHAAGAKVVLDNVFATPLLQQGFPLGVDVVVYSGTKHIDGQGRVLGGAILGDREYID

GPVQKLMRHTGPAMSAFNAWVLLKGLETLAIRVQHSNASAQRIAEFLNGHPSVRWVRYPY

LPSHPQYDLAKRQMSGGGTVVTFALDCPEDVAKQRAFEVLDKMRLIDISNNLGDAKSLVT

HPATTTHRAMGPEGRAAIGLGDGVVRISVGLEDTDDLIADIDRALS

>mtk:TBSG_00394 K10764 O-succinylhomoserine sulfhydrylase [EC:2.5.1.-] | (GenBank) O-succinylhomoserine sulfhydrylase metZ (A)

MTDESSVRTPKALPDGVSQATVGVRGGMLRSGFEETAEAMYLTSGYVYGSAAVAEKSFAG

ELDHYVYSRYGNPTVSVFEERLRLIEGAPAAFATASGMAAVFTSLGALLGAGDRLVAARS

LFGSCFVVCSEILPRWGVQTVFVDGDDLSQWERALSVPTQAVFFETPSNPMQSLVDIAAV

TELAHAAGAKVVLDNVFATPLLQQGFPLGVDVVVYSGTKHIDGQGRVLGGAILGDREYID

GPVQKLMRHTGPAMSAFNAWVLLKGLETLAIRVQHSNASAQRIAEFLNGHPSVRWVRYPY

LPSHPQYDLAKRQMSGGGTVVTFALDCPEDVAKQRAFEVLDKMRLIDISNNLGDAKSLVT

HPATTTHRAMGPEGRAAIGLGDGVVRISVGLEDTDDLIADIDRALS

>mtz:TBXG_000389 K10764 O-succinylhomoserine sulfhydrylase [EC:2.5.1.-] | (GenBank) O-succinylhomoserine sulfhydrylase metZ (A)

MTDESSVRTPKALPDGVSQATVGVRGGMLRSGFEETAEAMYLTSGYVYGSAAVAEKSFAG

ELDHYVYSRYGNPTVSVFEERLRLIEGAPAAFATASGMAAVFTSLGALLGAGDRLVAARS

LFGSCFVVCSEILPRWGVQTVFVDGDDLSQWERALSVPTQAVFFETPSNPMQSLVDIAAV

TELAHAAGAKVVLDNVFATPLLQQGFPLGVDVVVYSGTKHIDGQGRVLGGAILGDREYID

GPVQKLMRHTGPAMSAFNAWVLLKGLETLAIRVQHSNASAQRIAEFLNGHPSVRWVRYPY

LPSHPQYDLAKRQMSGGGTVVTFALDCPEDVAKQRAFEVLDKMRLIDISNNLGDAKSLVT

HPATTTHRAMGPEGRAAIGLGDGVVRISVGLEDTDDLIADIDRALS

>mtg:MRGA327_02470 K10764 O-succinylhomoserine sulfhydrylase [EC:2.5.1.-] | (GenBank) O-succinylhomoserine sulfhydrylase (A)

MTDESSVRTPKALPDGVSQATVGVRGGMLRSGFEETAEAMYLTSGYVYGSAAVAEKSFAG

ELDHYVYSRYGNPTVSVFEERLRLIEGAPAAFATASGMAAVFTSLGALLGAGDRLVAARS

LFGSCFVVCSEILPRWGVQTVFVDGDDLSQWERALSVPTQAVFFETPSNPMQSLVDIAAV

TELAHAAGAKVVLDNVFATPLLQQGFPLGVDVVVYSGTKHIDGQGRVLGGAILGDREYID

GPVQKLMRHTGPAMSAFNAWVLLKGLETLAIRVQHSNASAQRIAEFLNGHPSVRWVRYPY

LPSHPQYDLAKRQMSGGGTVVTFALDCPEDVAKQRAFEVLDKMRLIDISNNLGDAKSLVT

HPATTTHRAMGPEGRAAIGLGDGVVRISVGLEDTDDLIADIDRALS

>mti:MRGA423_00205 K00652 8-amino-7-oxononanoate synthase [EC:2.3.1.47] | (GenBank) 8-amino-7-oxononanoate synthase (A)

MPTGLGYDFLRPVEDSGINDLKHYYFMADLADGQPLGRANLYSVCFDLATTDRKLTPAWR

TTIKRWFPGFMTFRFLECGLLTMVSNPLALRSDTDLERVLPVLAGQMDQLAHDDGSDFLM

IRDVDPEHYQRYLDILRPLGFRPALGFSRVDTTISWSSVEEALGCLSHKRRLPLKTSLEF

RERFGIEVEELDEYAEHAPVLARLWRNVKTEAKDYQREDLNPEFFAACSRHLHGRSRLWL

FRYQGTPIAFFLNVWGADENYILLEWGIDRDFEHYRKANLYRAALMLSLKDAISRDKRRM

EMGITNYFTKLRIPGARVIPTIYFLRHSTDPVHTATLARMMMHNIQRPTLPDDMSEEFCR

WEERIRLDQDGLPEHDIFRKIDRQHKYTGLKLGGVYGFYPRFTGPQRSTVKAAELGEIVL

LGTNSYLGLATHPEVVEASAEATRRYGTGCSGSPLLNGTLDLHVSLEQELACFLGKPAAV

LCSTGYQSNLAAISALCESGDMIIQDALNHRSLFDAARLSGADFTLYRHNDMDHLARVLR

RTEGRRRIIVVDAVFSMEGTVADLATIAELADRHGCRVYVDESHALGVLGPDGRGASAAL

GVLARMDVVMGTFSKSFASVGGFIAGDRPVVDCIRHNGSGHVFSASLPPAAAAATHAALR

VSRREPDRRARVLAAAEYMATGLARQGYQAEYHGTAIVPVILGNPTVAHAGYLRLMRSGV

YVNPVAPPAVPEERSGFRTSYLADHRQSDLDRALHVFAGLAEDLTPQGAAL

>mte:CCDC5079_0365 K10764 O-succinylhomoserine sulfhydrylase [EC:2.5.1.-] | (GenBank) O-succinylhomoserine sulfhydrylase (A)

METGMTDESSVRTPKALPDGVSQATVGVRGGMLRSGFEETAEAMYLTSGYVYGSAAVAEK

SFAGELDHYVYSRYGNPTVSVFEERLRLIEGAPAAFATASGMAAVFTSLGALLGAGDRLV

AARSLFGSCFVVCSEILPRWGVQTVFVDGDDLSQWERALSVPTQAVFFETPSNPMQSLVD

IAAVTELAHAAGAKVVLDNVFATPLLQQGFPLGVDVVVYSGTKHIDGQGRVLGGAILGDR

EYIDGPVQKLMRHTGPAMSAFNAWVLLKGLETLAIRVQHSNASAQRIAEFLNGHPSVRWV

RYPYLPSHPQYDLAKRQMSGGGTVVTFALDCPEDVAKQRAFEVLDKMRLIDISNNLGDAK

SLVTHPATTTHRAMGPEGRAAIGLGDGVVRISVGLEDTDDLIADIDRALS

>mtur:CFBS_0406 K10764 O-succinylhomoserine sulfhydrylase [EC:2.5.1.-] | (GenBank) metZ; O-succinylhomoserine sulfhydrylase (A)

MTDESSVRTPKALPDGVSQATVGVRGGMLRSGFEETAEAMYLTSGYVYGSAAVAEKSFAG

ELDHYVYSRYGNPTVSVFEERLRLIEGAPAAFATASGMAAVFTSLGALLGAGDRLVAARS

LFGSCFVVCSEILPRWGVQTVFVDGDDLSQWERALSVPTQAVFFETPSNPMQSLVDIAAV

TELAHAAGAKVVLDNVFATPLLQQGFPLGVDVVVYSGTKHIDGQGRVLGGAILGDREYID

GPVQKLMRHTGPAMSAFNAWVLLKGLETLAIRVQHSNASAQRIAEFLNGHPSVRWVRYPY

LPSHPQYDLAKRQMSGGGTVVTFALDCPEDVAKQRAFEVLDKMRLIDISNNLGDAKSLVT

HPATTTHRAMGPEGRAAIGLGDGVVRISVGLEDTDDLIADIDRALS

>mtl:CCDC5180_0361 K10764 O-succinylhomoserine sulfhydrylase [EC:2.5.1.-] | (GenBank) O-succinylhomoserine sulfhydrylase (A)

METGMTDESSVRTPKALPDGVSQATVGVRGGMLRSGFEETAEAMYLTSGYVYGSAAVAEK

SFAGELDHYVYSRYGNPTVSVFEERLRLIEGAPAAFATASGMAAVFTSLGALLGAGDRLV

AARSLFGSCFVVCSEILPRWGVQTVFVDGDDLSQWERALSVPTQAVFFETPSNPMQSLVD

IAAVTELAHAAGAKVVLDNVFATPLLQQGFPLGVDVVVYSGTKHIDGQGRVLGGAILGDR

EYIDGPVQKLMRHTGPAMSAFNAWVLLKGLETLAIRVQHSNASAQRIAEFLNGHPSVRWV

RYPYLPSHPQYDLAKRQMSGGGTVVTFALDCPEDVAKQRAFEVLDKMRLIDISNNLGDAK

SLVTHPATTTHRAMGPEGRAAIGLGDGVVRISVGLEDTDDLIADIDRALS

>mto:MTCTRI2_0394 K10764 O-succinylhomoserine sulfhydrylase [EC:2.5.1.-] | (GenBank) metZ; O-succinylhomoserine sulfhydrylase (A)

MTDESSVRTPKALPDGVSQATVGVRGGMLRSGFEETAEAMYLTSGYVYGSAAVAEKSFAG

ELDHYVYSRYGNPTVSVFEERLRLIEGAPAAFATASGMAAVFTSLGALLGAGDRLVAARS

LFGSCFVVCSEILPRWGVQTVFVDGDDLSQWERALSVPTQAVFFETPSNPMQSLVDIAAV

TELAHAAGAKVVLDNVFATPLLQQGFPLGVDVVVYSGTKHIDGQGRVLGGAILGDREYID

GPVQKLMRHTGPAMSAFNAWVLLKGLETLAIRVQHSNASAQRIAEFLNGHPSVRWVRYPY

LPSHPQYDLAKRQMSGGGTVVTFALDCPEDVAKQRAFEVLDKMRLIDISNNLGDAKSLVT

HPATTTHRAMGPEGRAAIGLGDGVVRISVGLEDTDDLIADIDRALS

>mtd:UDA_0391 K10764 O-succinylhomoserine sulfhydrylase [EC:2.5.1.-] | (GenBank) metZ; metZ (A)

MTDESSVRTPKALPDGVSQATVGVRGGMLRSGFEETAEAMYLTSGYVYGSAAVAEKSFAG

ELDHYVYSRYGNPTVSVFEERLRLIEGAPAAFATASGMAAVFTSLGALLGAGDRLVAARS

LFGSCFVVCSEILPRWGVQTVFVDGDDLSQWERALSVPTQAVFFETPSNPMQSLVDIAAV

TELAHAAGAKVVLDNVFATPLLQQGFPLGVDVVVYSGTKHIDGQGRVLGGAILGDREYID

GPVQKLMRHTGPAMSAFNAWVLLKGLETLAIRVQHSNASAQRIAEFLNGHPSVRWVRYPY

LPSHPQYDLAKRQMSGGGTVVTFALDCPEDVAKQRAFEVLDKMRLIDISNNLGDAKSLVT

HPATTTHRAMGPEGRAAIGLGDGVVRISVGLEDTDDLIADIDRALS

>mtn:ERDMAN_0431 K10764 O-succinylhomoserine sulfhydrylase [EC:2.5.1.-] | (GenBank) metZ; O-succinylhomoserine sulfhydrylase (EC:4.2.99.-) (A)

METGMTDESSVRTPKALPDGVSQATVGVRGGMLRSGFEETAEAMYLTSGYVYGSAAVAEK

SFAGELDHYVYSRYGNPTVSVFEERLRLIEGAPAAFATASGMAAVFTSLGALLGAGDRLV

AARSLFGSCFVVCSEILPRWGVQTVFVDGDDLSQWERALSVPTQAVFFETPSNPMQSLVD

IAAVTELAHAAGAKVVLDNVFATPLLQQGFPLGVDVVVYSGTKHIDGQGRVLGGAILGDR

EYIDGPVQKLMRHTGPAMSAFNAWVLLKGLETLAIRVQHSNASAQRIAEFLNGHPSVRWV

RYPYLPSHPQYDLAKRQMSGGGTVVTFALDCPEDVAKQRAFEVLDKMRLIDISNNLGDAK

SLVTHPATTTHRAMGPEGRAAIGLGDGVVRISVGLEDTDDLIADIDRALS

>mtj:J112_02090 K10764 O-succinylhomoserine sulfhydrylase [EC:2.5.1.-] | (GenBank) O-succinylhomoserine sulfhydrylase (A)

MTDESSVRTPKALPDGVSQATVGVRGGMLRSGFEETAEAMYLTSGYVYGSAAVAEKSFAG

ELDHYVYSRYGNPTVSVFEERLRLIEGAPAAFATASGMAAVFTSLGALLGAGDRLVAARS

LFGSCFVVCSEILPRWGVQTVFVDGDDLSQWERALSVPTQAVFFETPSNPMQSLVDIAAV

TELAHAAGAKVVLDNVFATPLLQQGFPLGVDVVVYSGTKHIDGQGRVLGGAILGDREYID

GPVQKLMRHTGPAMSAFNAWVLLKGLETLAIRVQHSNASAQRIAEFLNGHPSVRWVRYPY

LPSHPQYDLAKRQMSGGGTVVTFALDCPEDVAKQRAFEVLDKMRLIDISNNLGDAKSLVT

HPATTTHRAMGPEGRAAIGLGDGVVRISVGLEDTDDLIADIDRALS

>mtub:MT7199_0396 K10764 O-succinylhomoserine sulfhydrylase [EC:2.5.1.-] | (GenBank) putative O-SUCCINYLHOMOSERINE SULFHYDRYLASE METZ (OSH SULFHYDRYLASE) (EC:4.2.99.-) (A)

MTDESSVRTPKALPDGVSQATVGVRGGMLRSGFEETAEAMYLTSGYVYGSAAVAEKSFAG

ELDHYVYSRYGNPTVSVFEERLRLIEGAPAAFATASGMAAVFTSLGALLGAGDRLVAARS

LFGSCFVVCSEILPRWGVQTVFVDGDDLSQWERALSVPTQAVFFETPSNPMQSLVDIAAV

TELAHAAGAKVVLDNVFATPLLQQGFPLGVDVVVYSGTKHIDGQGRVLGGAILGDREYID

GPVQKLMRHTGPAMSAFNAWVLLKGLETLAIRVQHSNASAQRIAEFLNGHPSVRWVRYPY

LPSHPQYDLAKRQMSGGGTVVTFALDCPEDVAKQRAFEVLDKMRLIDISNNLGDAKSLVT

HPATTTHRAMGPEGRAAIGLGDGVVRISVGLEDTDDLIADIDRALS

>mtuc:J113_07570 K01739 cystathionine gamma-synthase [EC:2.5.1.48] | (GenBank) cystathionine gamma-synthase (EC:2.5.1.48) (A)

MSEDRTGHQGISGPATRAIHAGYRPDPATGAVNVPIYASSTFAQDGVGGLRGGFEYARTG

NPTRAALEASLAAVEEGAFARAFSSGMAATDCALRAMLRPGDHVVIPDDAYGGTFRLIDK

VFTRWDVQYTPVRLADLDAVGAAITPRTRLIWVETPTNPLLSIADITAIAELGTDRSAKV

LVDNTFASPALQQPLRLGADVVLHSTTKYIGGHSDVVGGALVTNDEELDEEFAFLQNGAG

AVPGPFDAYLTMRGLKTLVLRMQRHSENACAVAEFLADHPSVSSVLYPGLPSHPGHEIAA

RQMRGFGGMVSVRMRAGRRAAQDLCAKTRVFILAESLGGVESLIEHPSAMTHASTAGSQL

EVPDDLVRLSVGIEDIADLLGDLEQALG

>mtue:J114_02085 K10764 O-succinylhomoserine sulfhydrylase [EC:2.5.1.-] | (GenBank) O-succinylhomoserine sulfhydrylase (A)

MTDESSVRTPKALPDGVSQATVGVRGGMLRSGFEETAEAMYLTSGYVYGSAAVAEKSFAG

ELDHYVYSRYGNPTVSVFEERLRLIEGAPAAFATASGMAAVFTSLGALLGAGDRLVAARS

LFGSCFVVCSEILPRWGVQTVFVDGDDLSQWERALSVPTQAVFFETPSNPMQSLVDIAAV

TELAHAAGAKVVLDNVFATPLLQQGFPLGVDVVVYSGTKHIDGQGRVLGGAILGDREYID

GPVQKLMRHTGPAMSAFNAWVLLKGLETLAIRVQHSNASAQRIAEFLNGHPSVRWVRYPY

LPSHPQYDLAKRQMSGGGTVVTFALDCPEDVAKQRAFEVLDKMRLIDISNNLGDAKSLVT

HPATTTHRAMGPEGRAAIGLGDGVVRISVGLEDTDDLIADIDRALS

>mtx:M943_02025 K10764 O-succinylhomoserine sulfhydrylase [EC:2.5.1.-] | (GenBank) O-succinylhomoserine sulfhydrylase (A)

MTDESSVRTPKALPDGVSQATVGVRGGMLRSGFEETAEAMYLTSGYVYGSAAVAEKSFAG

ELDHYVYSRYGNPTVSVFEERLRLIEGAPAAFATASGMAAVFTSLGALLGAGDRLVAARS

LFGSCFVVCSEILPRWGVQTVFVDGDDLSQWERALSVPTQAVFFETPSNPMQSLVDIAAV

TELAHAAGAKVVLDNVFATPLLQQGFPLGVDVVVYSGTKHIDGQGRVLGGAILGDREYID

GPVQKLMRHTGPAMSAFNAWVLLKGLETLAIRVQHSNASAQRIAEFLNGHPSVRWVRYPY

LPSHPQYDLAKRQMSGGGTVVTFALDCPEDVAKQRAFEVLDKMRLIDISNNLGDAKSLVT

HPATTTHRAMGPEGRAAIGLGDGVVRISVGLEDTDDLIADIDRALS

>mtuh:I917_02790 K10764 O-succinylhomoserine sulfhydrylase [EC:2.5.1.-] | (GenBank) O-succinylhomoserine sulfhydrylase (A)

MTDESSVRTPKALPDGVSQXTVGVRGGMLRXGXEETAEAMYLTSGYVXXSAAVAEKSFAG

ELXHYVYSRYGNPTVSVFEERLRLIEGAPAAFAXASGMAAVFTSLGALLGAGDRLVAARS

LFGSXFVVCSEILPRWGVQTVFVDGDDLSQWERALSVPTQAVFFETPSNPMQSLVDIAAV

TELAHAAGAKVVLDNVFATPLLQQGFPLGVDVVVYSGTKHIDGQGRVLGGAILGDREYID

GPVQKLMRHTGPAMSAFNAWVLLKGLETLAIRVQHSNASAQRIAEFLNGHPSVRWVXXPX

LPSHPQYDLAKRQMSGGGTVVTFALDCPEDVAKQRAFEVLDKMRLIDISNNLGDAKSLVX

XPATTTHRAMXXEGRAAIGLGDGVVRISVGLEDTDDLIADIDRALS

>mtul:TBHG_00384 K10764 O-succinylhomoserine sulfhydrylase [EC:2.5.1.-] | (GenBank) O-succinylhomoserine sulfhydrylase MetZ (A)

MTDESSVRTPKALPDGVSQATVGVRGGMLRSGFEETAEAMYLTSGYVYGSAAVAEKSFAG

ELDHYVYSRYGNPTVSVFEERLRLIEGAPAAFATASGMAAVFTSLGALLGAGDRLVAARS

LFGSCFVVCSEILPRWGVQTVFVDGDDLSQWERALSVPTQAVFFETPSNPMQSLVDIAAV

TELAHAAGAKVVLDNVFATPLLQQGFPLGVDVVVYSGTKHIDGQGRVLGGAILGDREYID

GPVQKLMRHTGPAMSAFNAWVLLKGLETLAIRVQHSNASAQRIAEFLNGHPSVRWVRYPY

LPSHPQYDLAKRQMSGGGTVVTFALDCPEDVAKQRAFEVLDKMRLIDISNNLGDAKSLVT

HPATTTHRAMGPEGRAAIGLGDGVVRISVGLEDTDDLIADIDRALS

>mtut:HKBT1_0406 K10764 O-succinylhomoserine sulfhydrylase [EC:2.5.1.-] | (GenBank) metZ; O-succinylhomoserine sulfhydrylase (A)

MTDESSVRTPKALPDGVSQATVGVRGGMLRSGFEETAEAMYLTSGYVYGSAAVAEKSFAG

ELDHYVYSRYGNPTVSVFEERLRLIEGAPAAFATASGMAAVFTSLGALLGAGDRLVAARS

LFGSCFVVCSEILPRWGVQTVFVDGDDLSQWERALSVPTQAVFFETPSNPMQSLVDIAAV

TELAHAAGAKVVLDNVFATPLLQQGFPLGVDVVVYSGTKHIDGQGRVLGGAILGDREYID

GPVQKLMRHTGPAMSAFNAWVLLKGLETLAIRVQHSNASAQRIAEFLNGHPSVRWVRYPY

LPSHPQYDLAKRQMSGGGTVVTFALDCPEDVAKQRAFEVLDKMRLIDISNNLGDAKSLVT

HPATTTHRAMGPEGRAAIGLGDGVVRISVGLEDTDDLIADIDRALS

>mtuu:HKBT2_0406 K10764 O-succinylhomoserine sulfhydrylase [EC:2.5.1.-] | (GenBank) metZ; O-succinylhomoserine sulfhydrylase (A)

MTDESSVRTPKALPDGVSQATVGVRGGMLRSGFEETAEAMYLTSGYVYGSAAVAEKSFAG

ELDHYVYSRYGNPTVSVFEERLRLIEGAPAAFATASGMAAVFTSLGALLGAGDRLVAARS

LFGSCFVVCSEILPRWGVQTVFVDGDDLSQWERALSVPTQAVFFETPSNPMQSLVDIAAV

TELAHAAGAKVVLDNVFATPLLQQGFPLGVDVVVYSGTKHIDGQGRVLGGAILGDREYID

GPVQKLMRHTGPAMSAFNAWVLLKGLETLAIRVQHSNASAQRIAEFLNGHPSVRWVRYPY

LPSHPQYDLAKRQMSGGGTVVTFALDCPEDVAKQRAFEVLDKMRLIDISNNLGDAKSLVT

HPATTTHRAMGPEGRAAIGLGDGVVRISVGLEDTDDLIADIDRALS

>mtq:HKBS1_0406 K10764 O-succinylhomoserine sulfhydrylase [EC:2.5.1.-] | (GenBank) metZ; O-succinylhomoserine sulfhydrylase (A)

MTDESSVRTPKALPDGVSQATVGVRGGMLRSGFEETAEAMYLTSGYVYGSAAVAEKSFAG

ELDHYVYSRYGNPTVSVFEERLRLIEGAPAAFATASGMAAVFTSLGALLGAGDRLVAARS

LFGSCFVVCSEILPRWGVQTVFVDGDDLSQWERALSVPTQAVFFETPSNPMQSLVDIAAV

TELAHAAGAKVVLDNVFATPLLQQGFPLGVDVVVYSGTKHIDGQGRVLGGAILGDREYID

GPVQKLMRHTGPAMSAFNAWVLLKGLETLAIRVQHSNASAQRIAEFLNGHPSVRWVRYPY

LPSHPQYDLAKRQMSGGGTVVTFALDCPEDVAKQRAFEVLDKMRLIDISNNLGDAKSLVT

HPATTTHRAMGPEGRAAIGLGDGVVRISVGLEDTDDLIADIDRALS

>mbo:Mb0397 K10764 O-succinylhomoserine sulfhydrylase [EC:2.5.1.-] | (RefSeq) metZ; O-succinylhomoserine sulfhydrylase (EC:4.2.99.-) (A)

MTDESSVRTPKALPDGVSQATVGVRGGMLRSGFEETAEAMYLTSGYVYGSAAVAEKSFAG

ELDHYVYSRYGNPTVSVFEERLRLIEGAPAAFATASGMAAVFTSLGALLGAGDRLVAARS

LFGSCFVVCSEILPRWGVQTVFVDGDDLSQWERALSVPTQAVFFETPSNPMQSLVDIAAV

TELAHAAGAKVVLDNVFATPLLQQGFPLGVDVVVYSGTKHIDGQGRVLGGAILGDREYID

GPVQKLMRHTGPAMSAFNAWVLLKGLETLAIRVQHSNASAQRIAEFLNGHPSVRWVRYPY

LPSHPQYDLAKRQMSGGGTVVTFALDCPEDVAKQRAFEVLDKMRLIDISNNLGDAKSLVT

HPATTTHRAMGPEGRAAIGLGDGVVRISVGLEDTDDLIADIDRALS

>mbb:BCG_0428 K10764 O-succinylhomoserine sulfhydrylase [EC:2.5.1.-] | (GenBank) metZ; Probable o-succinylhomoserine sulfhydrylase metZ (EC:4.2.99.-) (A)

MTDESSVRTPKALPDGVSQATVGVRGGMLRSGFEETAEAMYLTSGYVYGSAAVAEKSFAG

ELDHYVYSRYGNPTVSVFEERLRLIEGAPAAFATASGMAAVFTSLGALLGAGDRLVAARS

LFGSCFVVCSEILPRWGVQTVFVDGDDLSQWERALSVPTQAVFFETPSNPMQSLVDIAAV

TELAHAAGAKVVLDNVFATPLLQQGFPLGVDVVVYSGTKHIDGQGRVLGGAILGDREYID

GPVQKLMRHTGPAMSAFNAWVLLKGLETLAIRVQHSNASAQRIAEFLNGHPSVRWVRYPY

LPSHPQYDLAKRQMSGGGTVVTFALDCPEDVAKQRAFEVLDKMRLIDISNNLGDAKSLVT

HPATTTHRAMGPEGRAAIGLGDGVVRISVGLEDTDDLIADIDRALS

>mbt:JTY_0398 K10764 O-succinylhomoserine sulfhydrylase [EC:2.5.1.-] | (GenBank) metZ; O-succinylhomoserine sulfhydrylase (A)

MTDESSVRTPKALPDGVSQATVGVRGGMLRSGFEETAEAMYLTSGYVYGSAAVAEKSFAG

ELDHYVYSRYGNPTVSVFEERLRLIEGAPAAFATASGMAAVFTSLGALLGAGDRLVAARS

LFGSCFVVCSEILPRWGVQTVFVDGDDLSQWERALSVPTQAVFFETPSNPMQSLVDIAAV

TELAHAAGAKVVLDNVFATPLLQQGFPLGVDVVVYSGTKHIDGQGRVLGGAILGDREYID

GPVQKLMRHTGPAMSAFNAWVLLKGLETLAIRVQHSNASAQRIAEFLNGHPSVRWVRYPY

LPSHPQYDLAKRQMSGGGTVVTFALDCPEDVAKQRAFEVLDKMRLIDISNNLGDAKSLVT

HPATTTHRAMGPEGRAAIGLGDGVVRISVGLEDTDDLIADIDRALS

>mbm:BCGMEX_0398 K10764 O-succinylhomoserine sulfhydrylase [EC:2.5.1.-] | (GenBank) metZ; O-succinylhomoserine sulfhydrylase (A)

MTDESSVRTPKALPDGVSQATVGVRGGMLRSGFEETAEAMYLTSGYVYGSAAVAEKSFAG

ELDHYVYSRYGNPTVSVFEERLRLIEGAPAAFATASGMAAVFTSLGALLGAGDRLVAARS

LFGSCFVVCSEILPRWGVQTVFVDGDDLSQWERALSVPTQAVFFETPSNPMQSLVDIAAV

TELAHAAGAKVVLDNVFATPLLQQGFPLGVDVVVYSGTKHIDGQGRVLGGAILGDREYID

GPVQKLMRHTGPAMSAFNAWVLLKGLETLAIRVQHSNASAQRIAEFLNGHPSVRWVRYPY

LPSHPQYDLAKRQMSGGGTVVTFALDCPEDVAKQRAFEVLDKMRLIDISNNLGDAKSLVT

HPATTTHRAMGPEGRAAIGLGDGVVRISVGLEDTDDLIADIDRALS

>mbk:K60_004100 K10764 O-succinylhomoserine sulfhydrylase [EC:2.5.1.-] | (GenBank) O-succinylhomoserine sulfhydrylase (A)

METGMTDESSVRTPKALPDGVSQATVGVRGGMLRSGFEETAEAMYLTSGYVYGSAAVAEK

SFAGELDHYVYSRYGNPTVSVFEERLRLIEGAPAAFATASGMAAVFTSLGALLGAGDRLV

AARSLFGSCFVVCSEILPRWGVQTVFVDGDDLSQWERALSVPTQAVFFETPSNPMQSLVD

IAAVTELAHAAGAKVVLDNVFATPLLQQGFPLGVDVVVYSGTKHIDGQGRVLGGAILGDR

EYIDGPVQKLMRHTGPAMSAFNAWVLLKGLETLAIRVQHSNASAQRIAEFLNGHPSVRWV

RYPYLPSHPQYDLAKRQMSGGGTVVTFALDCPEDVAKQRAFEVLDKMRLIDISNNLGDAK

SLVTHPATTTHRAMGPEGRAAIGLGDGVVRISVGLEDTDDLIADIDRALS

>mbx:BCGT_0161 K10764 O-succinylhomoserine sulfhydrylase [EC:2.5.1.-] | (GenBank) O-acetylhomoserine sulfhydrylase (EC:2.5.1.49) (A)

METGMTDESSVRTPKALPDGVSQATVGVRGGMLRSGFEETAEAMYLTSGYVYGSAAVAEK

SFAGELDHYVYSRYGNPTVSVFEERLRLIEGAPAAFATASGMAAVFTSLGALLGAGDRLV

AARSLFGSCFVVCSEILPRWGVQTVFVDGDDLSQWERALSVPTQAVFFETPSNPMQSLVD

IAAVTELAHAAGAKVVLDNVFATPLLQQGFPLGVDVVVYSGTKHIDGQGRVLGGAILGDR

EYIDGPVQKLMRHTGPAMSAFNAWVLLKGLETLAIRVQHSNASAQRIAEFLNGHPSVRWV

RYPYLPSHPQYDLAKRQMSGGGTVVTFALDCPEDVAKQRAFEVLDKMRLIDISNNLGDAK

SLVTHPATTTHRAMGPEGRAAIGLGDGVVRISVGLEDTDDLIADIDRALS

>mbz:LH58_02095 K10764 O-succinylhomoserine sulfhydrylase [EC:2.5.1.-] | (GenBank) O-succinylhomoserine sulfhydrylase (A)

MTDESSVRTPKALPDGVSQATVGVRGGMLRSGFEETAEAMYLTSGYVYGSAAVAEKSFAG

ELDHYVYSRYGNPTVSVFEERLRLIEGAPAAFATASGMAAVFTSLGALLGAGDRLVAARS

LFGSCFVVCSEILPRWGVQTVFVDGDDLSQWERALSVPTQAVFFETPSNPMQSLVDIAAV

TELAHAAGAKVVLDNVFATPLLQQGFPLGVDVVVYSGTKHIDGQGRVLGGAILGDREYID

GPVQKLMRHTGPAMSAFNAWVLLKGLETLAIRVQHSNASAQRIAEFLNGHPSVRWVRYPY

LPSHPQYDLAKRQMSGGGTVVTFALDCPEDVAKQRAFEVLDKMRLIDISNNLGDAKSLVT

HPATTTHRAMGPEGRAAIGLGDGVVRISVGLEDTDDLIADIDRALS

>maf:MAF_03930 K10764 O-succinylhomoserine sulfhydrylase [EC:2.5.1.-] | (GenBank) metZ; putative O-succinylhomoserine sulfhydrylase MetZ (OSH sulfhydrylase) (EC:4.2.99.-) (A)

MTDESSVRTPKALPDGVSQATVGVRGGMLRSGFEETAEAMYLTSGYVYGSAAVAEKSFAG

ELDHYVYSRYGNPTVSVFEERLRLIEGAPAAFATASGMAAVFTSLGALLGAGDRLVAARS

LFGSCFVVCSEILPRWGVQTVFVDGDDLSQWERALSVPTQAVFFETPSNPMQSLVDIAAV

TELAHAAGAKVVLDNVFATPLLQQGFPLGVDVVVYSGTKHIDGQGRVLGGAILGDREYID

GPVQKLMRHTGPAMSAFNAWVLLKGLETLAIRVQHSNASAQRIAEFLNGHPSVRWVRYPY

LPSHPQYDLAKRQMSGGGTVVTFALDCPEDVAKQRAFEVLDKMRLIDISNNLGDAKSLVT

HPATTTHRAMGPEGRAAIGLGDGVVRISVGLEDTDDLIADIDRALS

>mce:MCAN_03931 K10764 O-succinylhomoserine sulfhydrylase [EC:2.5.1.-] | (GenBank) metZ; putative O-succinylhomoserine sulfhydrylase METZ (OSH sulfhydrylase) (A)

MTDESSVRTPKALPDGVSQATVGVRGGMLRSGFEETAEAMYLTSGYVYGSAAVAEKSFAG

ELDHYVYSRYGNPTVSVFEERLRLIEGAPAAFATASGMAAVFTSLGALLGAGDRLVAARS

LFGSCFVVCSEILPRWGVQTVFVDGDDLSQWERALAVPTQAVFFETPSNPMQSLVDIAAV

TELAHAAGAKVVLDNVFATPLLQQGFPLGVDVVVYSGTKHIDGQGRVLGGAILGDREYID

GPVQKLMRHTGPAMSAFNAWVLLKGLETLAIRVQHSNASAQRIAEFLNGHPSVRWVRYPY

LPSHPQYDLAKRQMSGGGTVVTFALDCPEDVAKQRAFEVLDKMRLIDISNNLGDAKSLVT

HPATTTHRAMGPEGRAAIGLGDGVVRISVGLEDTDDLIADIDRALS

>mcq:BN44_10433 K10764 O-succinylhomoserine sulfhydrylase [EC:2.5.1.-] | (GenBank) metZ; O-succinylhomoserine sulfhydrylase MetZ (EC:2.5.1.-) (A)

MTDESSVRTPQALPDGVSQATVGVRGGMLRSGFEETAEAMYLTSGYVYGSAAVAEKSFAG

ELDHYVYSRYGNPTVSVFEERLRLIEGAPAAFATASGMAAVFTSLGALLGAGDRLVAARS

LFGSCFVVCSEILPRWGVQTVFVDGDDLSQWERALSVPTQAVFFETPSNPMQSLVDIAAV

TELAHAAGAKVVLDNVFATPLLQQGFPLGVDVVVYSGTKHIDGQGRVLGGAILGDREYID

GPVQKLMRHTGPAMSAFNAWVLLKGLETLAIRVQHSNASAQRIAEFLNGHPSVRWVRYPY

LPSHPQYDLAKRQMSGGGTVVTFALDCPEDVAKQRAFEVLDKMRLIDISNNLGDAKSLVT

HPATTTHRAMGPEGRAAIGLGDGVVRISVGLEDTDDLIADIDRALS

>mcv:BN43_10424 K10764 O-succinylhomoserine sulfhydrylase [EC:2.5.1.-] | (GenBank) metZ; O-succinylhomoserine sulfhydrylase MetZ (EC:2.5.1.-) (A)

MTDESSVRTPKALPDGVSQATVGVRGGMLRSGFEETAEAMYLTSGYVYGSAAVAEKSFAG

ELDHYVYSRYGNPTVSVFEERLRLIEGAPAAFATASGMAAVFTSLGALLGAGDRLVAARS

LFGSCFVVCSEILPRWGVQTVFVDGDDLSQWERALSVPTQAVFFETPSNPMQSLVDIAAV

TELAHAAGAKVVLDNVFATPLLQQGFPLGVDVVVYSGTKHIDGQGRVLGGAILGDREYID

GPVQKLMRHTGPAMSAFNAWVLLKGLETLAIRVQHSNASAQRIAEFLNGHPSVRWVRYPY

LPSHPQYDLAKRQMSGGGTVVTFALDCPEDVAKQRAFEVLDKMRLIDISNNLGDAKSLVT

HPATTTHRAMGPEGRATIGLGDGVVRISVGLEDTDDLIADIDRALS

>mcx:BN42_20117 K10764 O-succinylhomoserine sulfhydrylase [EC:2.5.1.-] | (GenBank) metZ; O-succinylhomoserine sulfhydrylase MetZ (EC:2.5.1.-) (A)

MTDESSVRTPKALPDGVSQATVGVRGGMLRSGFEETAEAMYLTSGYVYGSAAVAEKSFAG

ELDHYVYSRYGNPTVSVFEERLRLIEGAPAAFATASGMAAVFTSLGALLGAGDRLVAARS

LFGSCFVVCSEILPRWGVQTVFVDGDDLSQWERALAVPTQAVFFETPSNPMQLLVDIAAV

TELAHAAGAKVVLDNVFATPLLQQGFPLGVDVVVYSGTKHIDGQGRVLGGAILGDREYID

GPVQKLMRHTGPAMSAFNAWVLLKGLETLAIRVQHSNASAQRIAEFLNGHPSVRWARYPY

LPSHPQYDLAKRQMSGGGTVVTFALDCPEDVAKQRAFEVLDKMRLIDISNNLGDAKSLVT

HPATTTHRAMGPEGRAAIGLGDGVVRISVGLEDTDDLIADIDRALS

>mcz:BN45_10435 K10764 O-succinylhomoserine sulfhydrylase [EC:2.5.1.-] | (GenBank) metZ; O-succinylhomoserine sulfhydrylase MetZ (EC:2.5.1.-) (A)

MTRPGEPVRTPKALPDGVGQATVGVRGGMLRSGFEETAEAMYLTSGYVYGSAAVAEKSFA

GELDHYVYSRYGNPTVSVFEERLRLIEGAPAAFATASGMAAVFTSLGALLAAGDRLVAAR

SLFGSCFVVCNEILPRWGVQTVFVDGDDLSQWERALSVPTQAVFFETPSNPMQSLVDIAA

VTELAHAAGAKVVLDNVFATPLLQRGFPLGVDVVVYSGTKHIDGQGRVLGGAILGDREYI

DGPVQKLMRHTGPAMSAFNAWVLLKGLETLAIRVQHSNASAQRIAEFLNGHPSVRWVRYP

YLPSHPQHDLAKRQMSGGGTVVTFALDCAEDVAKQRAFEVLDKMRLIDISNNLGDAKSLV

THPATTTHRAMGPEGRAAIGLGDGVVRISVGLEDTDDLIADIDRALS

>mle:ML0275 K10764 O-succinylhomoserine sulfhydrylase [EC:2.5.1.-] | (RefSeq) metZ; O-succinylhomoserine sulfhydrylase (A)

MTDDRSVRTPKALPDGVTAATVGVRGGLLRSRFEETAETMYLTSGYVYESAVVAEKSFTG

ELDHFVYSRYGNPTVTMFEERLRLIEGAPAAFVTASGMAAVFTSLGALLASGDRLVAARS

LFGSCFVVCNEILPRWGVETVFVDGEDLVQWEQALSVPTQAVFFETPSNPMQSLVDIAAV

TELAHAAGAKVVLDNAFATPLLQHGLPLGVDVVIYSGTKHIDGQGRVLGGAILGDRDYID

GPVQKLMRHTGPAMSAFNAWILLKGLETLAIRVDHCNSSAHRIAEFLEKHPAVSWVRYPF

LVSHPQYDLAKRQMSGGGTVVTFALNSPEDAAKQRAFEVLDRLRLIDISNNFGDVKSLIT

HPATTTHRAMGPEGRAAIGLGDGVVRISIGLEDAADLIADIDQALS

>mlb:MLBr00275 K10764 O-succinylhomoserine sulfhydrylase [EC:2.5.1.-] | (GenBank) metZ; putative o-succinylhomoserine sulfhydrylase (EC:4.2.99.-) (A)

MTDDRSVRTPKALPDGVTAATVGVRGGLLRSRFEETAETMYLTSGYVYESAVVAEKSFTG

ELDHFVYSRYGNPTVTMFEERLRLIEGAPAAFVTASGMAAVFTSLGALLASGDRLVAARS

LFGSCFVVCNEILPRWGVETVFVDGEDLVQWEQALSVPTQAVFFETPSNPMQSLVDIAAV

TELAHAAGAKVVLDNAFATPLLQHGLPLGVDVVIYSGTKHIDGQGRVLGGAILGDRDYID

GPVQKLMRHTGPAMSAFNAWILLKGLETLAIRVDHCNSSAHRIAEFLEKHPAVSWVRYPF

LVSHPQYDLAKRQMSGGGTVVTFALNSPEDAAKQRAFEVLDRLRLIDISNNFGDVKSLIT

HPATTTHRAMGPEGRAAIGLGDGVVRISIGLEDAADLIADIDQALS

>mpa:MAP_3873 K10764 O-succinylhomoserine sulfhydrylase [EC:2.5.1.-] | (GenBank) metZ; MetZ (A)

MSQAGDDSVRTPPALPDGVSQATIGVRGGLLRSEFDETAEALYLTSGYVYESAAVAEQSF

TGELDHFVYSRYGNPTVTMFEERLRLLEGAPAAFATASGMAAVFTSLGALLAAGDRLVAA

RSLFGSCFVVCNEILPRWGVQTVFVDGDDLAQWEEALSVPTAAVFFETPSNPMQSLVDIA

AVTELAHAAGAKVVLDNVFATPLLQQGIPLGVDVVVYSGTKHIDGQGRVLGGAILGDRDY

IDGPVQKLMRHTGPAMSAFNAWVLLKGLETMAIRVEHSNSSAHRIAEFLETHPAVRWVRY

PYLPSHPQYDLAKRQMSGGGTVITFALDCPDDKAKQRAFEVLDKLTLIDISNNLGDAKSL

VTHPATTTHRAMGPEGRAAIGLGDGVVRISVGLEGTDDLIADIDRALG

>mao:MAP4_3988 K10764 O-succinylhomoserine sulfhydrylase [EC:2.5.1.-] | (GenBank) O-acetylhomoserine sulfhydrylase MetZ (A)

MSQAGDDSVRTPPALPDGVSQATIGVRGGLLRSEFDETAEALYLTSGYVYESAAVAEQSF

TGELDHFVYSRYGNPTVTMFEERLRLLEGAPAAFATASGMAAVFTSLGALLAAGDRLVAA

RSLFGSCFVVCNEILPRWGVQTVFVDGDDLAQWEEALSVPTAAVFFETPSNPMQSLVDIA

AVTELAHAAGAKVVLDNVFATPLLQQGIPLGVDVVVYSGTKHIDGQGRVLGGAILGDRDY

IDGPVQKLMRHTGPAMSAFNAWVLLKGLETMAIRVEHSNSSAHRIAEFLETHPAVRWVRY

PYLPSHPQYDLAKRQMSGGGTVITFALDCPDDKAKQRAFEVLDKLTLIDISNNLGDAKSL

VTHPATTTHRAMGPEGRAAIGLGDGVVRISVGLEGTDDLIADIDRALG

>mavi:RC58_19805 K10764 O-succinylhomoserine sulfhydrylase [EC:2.5.1.-] | (GenBank) O-succinylhomoserine sulfhydrylase (A)

MSQAGDDSVRTPPALPDGVSQATIGVRGGLLRSEFDETAEALYLTSGYVYESAAVAEQSF

TGELDHFVYSRYGNPTVTMFEERLRLLEGAPAAFATASGMAAVFTSLGALLAAGDRLVAA

RSLFGSCFVVCNEILPRWGVQTVFVDGDDLAQWEEALSVPTAAVFFETPSNPMQSLVDIA

AVTELAHAAGAKVVLDNVFATPLLQQGIPLGVDVVVYSGTKHIDGQGRVLGGAILGDRDY

IDGPVQKLMRHTGPAMSAFNAWVLLKGLETMAIRVEHSNSSAHRIAEFLETHPAVRWVRY

PYLPSHPQYDLAKRQMSGGGTVITFALDCPDDKAKQRAFEVLDKLTLIDISNNLGDAKSL

VTHPATTTHRAMGPEGRAAIGLGDGVVRISVGLEGTDDLIADIDRALG

>mavu:RE97_19860 K10764 O-succinylhomoserine sulfhydrylase [EC:2.5.1.-] | (GenBank) O-succinylhomoserine sulfhydrylase (A)

MSQAGDDSVRTPPALPDGVSQATIGVRGGLLRSEFDETAEALYLTSGYVYESAAVAEQSF

TGELDHFVYSRYGNPTVTMFEERLRLLEGAPAAFATASGMAAVFTSLGALLAAGDRLVAA

RSLFGSCFVVCNEILPRWGVQTVFVDGDDLAQWEEALSVPTAAVFFETPSNPMQSLVDIA

AVTELAHAAGAKVVLDNVFATPLLQQGIPLGVDVVVYSGTKHIDGQGRVLGGAILGDRDY

IDGPVQKLMRHTGPAMSAFNAWVLLKGLETMAIRVEHSNSSAHRIAEFLETHPAVRWVRY

PYLPSHPQYDLAKRQMSGGGTVITFALDCPDDKAKQRAFEVLDKLTLIDISNNLGDAKSL

VTHPATTTHRAMGPEGRAAIGLGDGVVRISVGLEGTDDLIADIDRALG

>mav:MAV_4773 K10764 O-succinylhomoserine sulfhydrylase [EC:2.5.1.-] | (GenBank) metZ; O-succinylhomoserine sulfhydrylase (EC:4.2.99.-) (A)

MSQAGDDSVRTPPALPDGVSQATIGVRGGLLRSGFDETAEALYLTSGYVYESAAVAEQSF

TGELDHFVYSRYGNPTVTMFEERLRLLEGAPAAFATASGMAAVFTSLGALLAAGDRLVAA

RSLFGSCFVVCNEILPRWGVQTVFVDGDDLAQWEEALSVPTAAVFFETPSNPMQSLVDIA

AVTELAHAAGAKVVLDNVFATPLLQQGIPLGVDVVVYSGTKHIDGQGRVLGGAILGDRDY

IDGPVQKLMRHTGPAMSAFNAWVLLKGLETMAIRVEHSNSSAHRIAEFLETHPAVRWVRY

PYLPSHPQYDLAKRQMSGGGTVITFALDCPDDKAKQRAFEVLDKLTLIDISNNLGDAKSL

VTHPATTTHRAMGPEGRAAIGLGDGVVRISVGLEGTDDLIADIDRALG

>mavr:LA63_22265 K10764 O-succinylhomoserine sulfhydrylase [EC:2.5.1.-] | (GenBank) O-succinylhomoserine sulfhydrylase (A)

MSQAGDDSVRTPPALPDGVSQATIGVRGGLLRSGFDETAEALYLTSGYVYESAAVAEQSF

TGELDHFVYSRYGNPTVTMFEERLRLLEGAPAAFATASGMAAVFTSLGALLAAGDRLVAA

RSLFGSCFVVCNEILPRWGVQTVFVDGDDLAQWEEALSVPTAAVFFETPSNPMQSLVDIA

AVTELAHAAGAKVVLDNVFATPLLQQGIPLGVDVVVYSGTKHIDGQGRVLGGAILGDRDY

IDGPVQKLMRHTGPAMSAFNAWVLLKGLETMAIRVEHSNSSAHRIAEFLETHPAVRWVRY

PYLPSHPQYDLAKRQMSGGGTVITFALDCPDDKAKQRAFEVLDKLTLIDISNNLGDAKSL

VTHPATTTHRAMGPEGRAAIGLGDGVVRISVGLEGTDDLIADIDRALG

>mavd:NF84_22045 K10764 O-succinylhomoserine sulfhydrylase [EC:2.5.1.-] | (GenBank) O-succinylhomoserine sulfhydrylase (A)

MSQAGDDSVRTPPALPDGVSQATIGVRGGLLRSGFDETAEALYLTSGYVYESAAVAEQSF

TGELDHFVYSRYGNPTVTMFEERLRLLEGAPAAFATASGMAAVFTSLGALLAAGDRLVAA

RSLFGSCFVVCNEILPRWGVQTVFVDGDDLAQWEEALSVPTAAVFFETPSNPMQSLVDIA

AVTELAHAAGAKVVLDNVFATPLLQQGIPLGVDVVVYSGTKHIDGQGRVLGGAILGDRDY

IDGPVQKLMRHTGPAMSAFNAWVLLKGLETMAIRVEHSNSSAHRIAEFLETHPAVRWVRY

PYLPSHPQYDLAKRQMSGGGTVITFALDCPDDKAKQRAFEVLDKLTLIDISNNLGDAKSL

VTHPATTTHRAMGPEGRAAIGLGDGVVRISVGLEGTDDLIADIDRALG

>mava:LA64_22235 K10764 O-succinylhomoserine sulfhydrylase [EC:2.5.1.-] | (GenBank) O-succinylhomoserine sulfhydrylase (A)

MSQAGDDSVRTPPALPDGVSQATIGVRGGLLRSGFDETAEALYLTSGYVYESAAVAEQSF

TGELDHFVYSRYGNPTVTMFEERLRLLEGAPAAFATASGMAAVFTSLGALLAAGDRLVAA

RSLFGSCFVVCNEILPRWGVQTVFVDGDDLAQWEEALSVPTAAVFFETPSNPMQSLVDIA

AVTELAHAAGAKVVLDNVFATPLLQQGIPLGVDVVVYSGTKHIDGQGRVLGGAILGDRDY

IDGPVQKLMRHTGPAMSAFNAWVLLKGLETMAIRVEHSNSSAHRIAEFLETHPAVRWVRY

PYLPSHPQYDLAKRQMSGGGTVITFALDCPDDKAKQRAFEVLDKLTLIDISNNLGDAKSL

VTHPATTTHRAMGPEGRAAIGLGDGVVRISVGLEGTDDLIADIDRALG

>mit:OCO_46840 K10764 O-succinylhomoserine sulfhydrylase [EC:2.5.1.-] | (GenBank) O-succinylhomoserine sulfhydrylase (A)

MTPADHDSVRIPAPLPEGVSQATIGVRGGLLRSGFDETAEALYLTSGYVYETAAVAEQSF

TGEVDRFVYSRYGNPTVTMFEERLRLLEGAPAAFATASGMAAVFTSLGALLGAGDRLVAS

RSLFGSCFVVCNEILPRWGVETVFVDGDDLSQWEQALSVPTTAVFFETPSNPMQSLVDIA

AVTELAHAAGAKVVLDNVFATPLLQQGVPLGVDVVVYSGTKHIDGQGRVLGGAILGDKEY

IDGPVQKLMRHTGPAMSAFNAWVLLKGLETMAIRVEHSNSSAYRIAEFLETHPAVSWVRY

PYLSSHPQYDLAKRQMSGGGTVITFALDCPDSRAKQRAFEVLDKLTLIDISNNLGDAKSL

VTHPATTTHRAMGPEGRAAIGLGDGVVRISVGLEGTDDLIADIDRALS

>mir:OCQ_47860 K10764 O-succinylhomoserine sulfhydrylase [EC:2.5.1.-] | (GenBank) O-succinylhomoserine sulfhydrylase (A)

MTPADHDSVRTPAPLPEGVSQATIGVRGGLLRSGFDETAEALYLTSGYVYETAAVAEQSF

TGEVDRFVYSRYGNPTVTMFEERLRLLEGAPAAFATASGMAAVFTSLGALLGAGDRLVAS

RSLFGSCFVVCNEILPRWGVETVFVDGDDLSQWEQALSVPTTAVFFETPSNPMQSLVDIA

AVTELAHAAGAKVVLDNVFATPLLQQGVPLGVDVVVYSGTKHIDGQGRVLGGAILGDKEY

IDGPVQKLMRHTGPAMSAFNAWVLLKGLETMAIRVEHSNSSAYRIAEFLETHPAVSWVRY

PYLSSHPQYDLAKRQMSGGGTVITFALDCPDSRAKQRAFEVLDKLTLIDISNNLGDAKSL

VTHPATTTHRAMGPEGRAAIGLGDGVVRISVGLEGTDDLIADIDRALS

>mia:OCU_46570 K10764 O-succinylhomoserine sulfhydrylase [EC:2.5.1.-] | (GenBank) O-succinylhomoserine sulfhydrylase (A)

MTPADHDSVRTPAPLPEGVSQATIGVRGGLLRSGFDETAEALYLTSGYVYETAAVAEQSF

TGEVDRFVYSRYGNPTVTMFEERLRLLEGAPAAFATASGMAAVFTSLGALLGAGDRLVAS

RSLFGSCFVVCNEILPRWGVETVFVDGDDLSQWEQALSVPTTAVFFETPSNPMQSLVDIA

AVTELAHAAGAKVVLDNVFATPLLQQGVPLGVDVVVYSGTKHIDGQGRVLGGAILGDKEY

IDGPVQKLMRHTGPAMSAFNAWVLLKGLETMAIRVEHSNSSAYRIAEFLETHPAVSWVRY

PYLSSHPQYDLAKRQMSGGGTVITFALDCPDSRAKQRAFEVLDKLTLIDISNNLGDAKSL

VTHPATTTHRAMGPEGRAAIGLGDGVVRISVGLEGTDDLIADIDRALS

>mie:LG41_22165 K10764 O-succinylhomoserine sulfhydrylase [EC:2.5.1.-] | (GenBank) O-succinylhomoserine sulfhydrylase (A)

MTPADHDSVRTPAPLPEGVSQATIGVRGGLLRSGFDETAEALYLTSGYVYETAAVAEQSF

TGEVDRFVYSRYGNPTVTMFEERLRLLEGAPAAFATASGMAAVFTSLGALLGAGDRLVAS

RSLFGSCFVVCNEILPRWGVETVFVDGDDLSQWEQALSVPTTAVFFETPSNPMQSLVDIA

AVTELAHAAGAKVVLDNVFATPLLQQGVPLGVDVVVYSGTKHIDGQGRVLGGAILGDKEY

IDGPVQKLMRHTGPAMSAFNAWVLLKGLETMAIRVEHSNSSAYRIAEFLETHPAVSWVRY

PYLSSHPQYDLAKRQMSGGGTVITFALDCPDSRAKQRAFEVLDKLTLIDISNNLGDAKSL

VTHPATTTHRAMGPEGRAAIGLGDGVVRISVGLEGTDDLIADIDRALS

>mid:MIP_07087 K10764 O-succinylhomoserine sulfhydrylase [EC:2.5.1.-] | (GenBank) O-succinylhomoserine sulfhydrylase (A)

MTPADHDSVRTPAPLPEGVSQATIGVRGGLLRSGFDETAEALYLTSGYVYETAAVAEQSF

TGEVDRFVYSRYGNPTVTMFEERLRLLEGAPAAFATASGMAAVFTSLGALLGAGDRLVAS

RSLFGSCFVVCNEILPRWGVETVFVDGDDLSQWEQALSVPTTAVFFETPSNPMQSLVDIA

AVTELAHAAGAKVVLDNVFATPLLQQGVPLGVDVVVYSGTKHIDGQGRVLGGAILGDKEY

IDGPVQKLMRHTGPAMSAFNAWVLLKGLETMAIRVEHSNSSAYRIAEFLETHPAVSWVRY

PYLSSHPQYDLAKRQMSGGGTVITFALDCPDSRAKQRAFEVLDKLTLIDISNNLGDAKSL

VTHPATTTHRAMGPEGRAAIGLGDGVVRISVGLEGTDDLIADIDRALS

>myo:OEM_46950 K10764 O-succinylhomoserine sulfhydrylase [EC:2.5.1.-] | (GenBank) O-succinylhomoserine sulfhydrylase (A)

MTPADHDSVRTPAPLPEGVSQATIGVRGGLLRSGFDETAEALYLTSGYVYETAAVAEQSF

TGEVDRFVYSRYGNPTVTMFEERLRLLEGAPAAFATASGMAAVFTSLGALLGAGDRLVAS

RSLFGSCFVVCNEVLPRWGVETVFVDGDDLSQWEQALSVPTTAVFFETPSNPMQSLVDIA

AVTELAHAAGAKVVLDNVFATPLLQQGVPLGVDVVVYSGTKHIDGQGRVLGGAILGDKEY

IDGPVQKLMRHTGPAMSAFNAWVLLKGLETMAIRVEHSNSSAYRIAEFLETHPAVSWVRY

PYLSSHPQYDLAKRQMSGGGTVITFALDCPDSRAKQRAFEVLDKLTLIDISNNLGDAKSL

VTHPATTTHRAMGPEGRAAIGLGDGVVRISVGLEGTDDLIADIDRALS

>msm:MSMEG_0769 K10764 O-succinylhomoserine sulfhydrylase [EC:2.5.1.-] | (RefSeq) metZ; O-succinylhomoserine sulfhydrylase (EC:4.2.99.-) (A)

MTDDIPSVRIPAPLPDGVSQATIGVRGGLLRSGFEETAEAMYLTSGYVYETAAAAEKAFT

GDIDRYVYSRYGNPTISMFEERLRLIEGAPACFATATGMAAVFTALGALLGAGDRLVAAR

SLFGSCFVVCNEILPRWGVETVFVDGDDLSQWEEALSVPTTAVFFETPSNPMQSLVDIAA

VSEMAHAAGAKVVLDNVFATPLLQQGIPMGADVVVYSGTKHIDGQGRVLGGAILGDQEYI

DGPVQKLMRHTGPAISAFNAWVLLKGLETLAVRVDYSNRSAQRVAEFLEGHPAVRWVKYP

FLQSHPQYELAKRQMRGGGTVVTFELDGDDGKARAFEVLDKLRVIDISNNLGDAKSLITH

PATTTHRAMGPEGRAAIGLGDGVVRISIGLEGTEDLIADLDQALS

>msg:MSMEI_0753 K10764 O-succinylhomoserine sulfhydrylase [EC:2.5.1.-] | (GenBank) metZ; O-succinylhomoserine sulfhydrylase (EC:2.5.1.48) (A)

MTDDIPSVRIPAPLPDGVSQATIGVRGGLLRSGFEETAEAMYLTSGYVYETAAAAEKAFT

GDIDRYVYSRYGNPTISMFEERLRLIEGAPACFATATGMAAVFTALGALLGAGDRLVAAR

SLFGSCFVVCNEILPRWGVETVFVDGDDLSQWEEALSVPTTAVFFETPSNPMQSLVDIAA

VSEMAHAAGAKVVLDNVFATPLLQQGIPMGADVVVYSGTKHIDGQGRVLGGAILGDQEYI

DGPVQKLMRHTGPAISAFNAWVLLKGLETLAVRVDYSNRSAQRVAEFLEGHPAVRWVKYP

FLQSHPQYELAKRQMRGGGTVVTFELDGDDGKARAFEVLDKLRVIDISNNLGDAKSLITH

PATTTHRAMGPEGRAAIGLGDGVVRISIGLEGTEDLIADLDQALS

>msb:LJ00_03820 K10764 O-succinylhomoserine sulfhydrylase [EC:2.5.1.-] | (GenBank) O-succinylhomoserine sulfhydrylase (A)

MTDDIPSVRIPAPLPDGVSQATIGVRGGLLRSGFEETAEAMYLTSGYVYETAAAAEKAFT

GDIDRYVYSRYGNPTISMFEERLRLIEGAPACFATATGMAAVFTALGALLGAGDRLVAAR

SLFGSCFVVCNEILPRWGVETVFVDGDDLSQWEEALSVPTTAVFFETPSNPMQSLVDIAA

VSEMAHAAGAKVVLDNVFATPLLQQGIPMGADVVVYSGTKHIDGQGRVLGGAILGDQEYI

DGPVQKLMRHTGPAISAFNAWVLLKGLETLAVRVDYSNRSAQRVAEFLEGHPAVRWVKYP

FLQSHPQYELAKRQMRGGGTVVTFELDGDDGKARAFEVLDKLRVIDISNNLGDAKSLITH

PATTTHRAMGPEGRAAIGLGDGVVRISIGLEGTEDLIADLDQALS

>msn:LI99_03820 K10764 O-succinylhomoserine sulfhydrylase [EC:2.5.1.-] | (GenBank) O-succinylhomoserine sulfhydrylase (A)

MTDDIPSVRIPAPLPDGVSQATIGVRGGLLRSGFEETAEAMYLTSGYVYETAAAAEKAFT

GDIDRYVYSRYGNPTISMFEERLRLIEGAPACFATATGMAAVFTALGALLGAGDRLVAAR

SLFGSCFVVCNEILPRWGVETVFVDGDDLSQWEEALSVPTTAVFFETPSNPMQSLVDIAA

VSEMAHAAGAKVVLDNVFATPLLQQGIPMGADVVVYSGTKHIDGQGRVLGGAILGDQEYI

DGPVQKLMRHTGPAISAFNAWVLLKGLETLAVRVDYSNRSAQRVAEFLEGHPAVRWVKYP

FLQSHPQYELAKRQMRGGGTVVTFELDGDDGKARAFEVLDKLRVIDISNNLGDAKSLITH

PATTTHRAMGPEGRAAIGLGDGVVRISIGLEGTEDLIADLDQALS

>msh:LI98_03820 K10764 O-succinylhomoserine sulfhydrylase [EC:2.5.1.-] | (GenBank) O-succinylhomoserine sulfhydrylase (A)

MTDDIPSVRIPAPLPDGVSQATIGVRGGLLRSGFEETAEAMYLTSGYVYETAAAAEKAFT

GDIDRYVYSRYGNPTISMFEERLRLIEGAPACFATATGMAAVFTALGALLGAGDRLVAAR

SLFGSCFVVCNEILPRWGVETVFVDGDDLSQWEEALSVPTTAVFFETPSNPMQSLVDIAA

VSEMAHAAGAKVVLDNVFATPLLQQGIPMGADVVVYSGTKHIDGQGRVLGGAILGDQEYI

DGPVQKLMRHTGPAISAFNAWVLLKGLETLAVRVDYSNRSAQRVAEFLEGHPAVRWVKYP

FLQSHPQYELAKRQMRGGGTVVTFELDGDDGKARAFEVLDKLRVIDISNNLGDAKSLITH

PATTTHRAMGPEGRAAIGLGDGVVRISIGLEGTEDLIADLDQALS

>msa:Mycsm_00411 K10764 O-succinylhomoserine sulfhydrylase [EC:2.5.1.-] | (GenBank) O-succinylhomoserine sulfhydrylase (A)

MESRRPAMETVLSNHDQVPSVRRPAQLPDGVSQATIGVRGGLLRSGFEETAEAMYLTSGY

VYSSAAEAEKAFTGDIDRYVYSRYGNPTISMFEERLRLIEGAPACFATATGMAAVFTSLG

ALLGAGDRLVAARSLFGSCFVVCSEILPRWGVETVFVDGDDLSQWEEALSVPTQAVFFET

PSNPMQSLVDIAAVCDLAHAAGAKVVLDNVFATPILQQGFPLGVDVVVYSGTKHIDGQGR

VLGGAILGDQQYIDEPVQKLMRHTGPALSPFNAWTLLKGLETLTVRVEHQNASAQRVAEF

LEKHPSVSWVKYPFLESHPQYDLAKRQMTGGGTVVTFELKGGTKERAFEVLDKLQIIDIS

NNLGDSKSLITHPATTTHRAMGPEGRAAIGLGDGVVRISVGLEGTEDLIGDLDQALS

>mul:MUL_1438 K01740 O-acetylhomoserine (thiol)-lyase [EC:2.5.1.49] | (GenBank) metC; O-acetylhomoserine sulfhydrylase MetC (A)

MSAENTSTDADPTAHWSFETKQIHAGQQPDSATNARALPIYQTTSYTFENTAHAAALFGL

EVPGNIYTRLGNPTTDVVEQRIAALEGGVAALFLSSGQAAETFGILNLAGAGDHIVSSPR

LYGGTYNLFHYSLAKLGIEVSFVDDPDNLDSWQAAVRPNTKAFFGETISNPQIDLLDTPG

VAEVAHRNGIPLIVDNTIATPYLIRPFTQGADIVVHSATKYLGGHGAAIAGVIVDGGTFD

WTQGRFPEFTTPDPSYHGVVFAELGAPAYALKARVQLLRDLGSAASPFNAFLVAQGLETL

SLRIERHVSNAQRVAEFLADREDVVTVNYAGLPGSPWHERAKKLSPKGTGAVLSFELAGG

VEAGKAFVNALKLHSHVANIGDVRSLVIHPASTTHAQLSPAEQLSTGVSPGLVRLAVGIE

GIEDILADLELGFAAAREFSGDSQAVAAI

>mva:Mvan_0688 K10764 O-succinylhomoserine sulfhydrylase [EC:2.5.1.-] | (GenBank) O-succinylhomoserine sulfhydrylase (EC:2.5.1.48) (A)

MTEVPSVRIPAVLPEGVSQETIGVRGGLLRSGFEETAEALYLTSGYVYESAAAAEKAFTG

EIDRYVYSRYGNPTVSMFEERLRLIEGAPACFATSSGMSAVFTALGALLGAGDRLVAARS

LFGSCFVVCNEILPRWGVETVFVDGDDLSQWEQALSVPTQAVFFETPSNPMQSLVDIAAV

CELAHAAGAKVVLDNVFATPILQQGFPLGVDVVVYSGTKHIDGQGRVLGGAILGSKEYID

EPVQKLMRHTGPALSPFNAWTLLKGLETLSLRVQHQNSSAHRIAEFLEQHPSVSWVKYPF

LESHPQYDLAKRQMTGGGTVVTFELAGAGGAGNPDAAKERAFEVLDKLQIVDISNNLGDA

KSLITHPATTTHRAMGPEGRAAIGLGDGVVRISIGLEGTEDLLADLDRALG

>mgi:Mflv_0219 K10764 O-succinylhomoserine sulfhydrylase [EC:2.5.1.-] | (GenBank) O-succinylhomoserine sulfhydrylase (EC:2.5.1.48) (A)

MSPEPDSVPSVRIPTPLPDGVGQATIGVRGGLLRSGFEETAEAIYLTSGYVYESAAEAEK

AFTGEIDRYVYSRYGNPTISMFEERLRLLEGAPACFGTASGMSAVFTSLGALLGAGDRLV

AARSLFGSCFVVCNEILPRWGVETVFVDGEDLAQWEEALSVPTQAVFFETPSNPMQSLVD

IAAVCDLAHAAGAKVVLDNVFATPILQQGMPLGADVVVYSGTKHIDGQGRVLGGAILGDK

EYIDGPVQKLMRHTGPALSPFNAWTLLKGLETMALRVQHQNSSAHRIAEFLEGHSAVSWV

KYPFLESHPQYDLAKRQMTGGGTVVTFELAGATKERAFEVLDKLQIVDISNNLGDAKSLI

THPATTTHRAMGPEGRAAIGLGDGVVRISVGLEGTEDLIADLDRALG

>msp:Mspyr1_05580 K10764 O-succinylhomoserine sulfhydrylase [EC:2.5.1.-] | (GenBank) O-succinylhomoserine sulfhydrylase (A)

MSPEPDSVPSVRIPTPLPDGVGQATIGVRGGLLRSGFEETAEAIYLTSGYVYESAAEAEK

AFTGEIDRYVYSRYGNPTISMFEERLRLLEGAPACFGTASGMSAVFTSLGALLGAGDRLV

AARSLFGSCFVVCNEILPRWGVETVFVDGEDLAQWEEALSVPTQAVFFETPSNPMQSLVD

IAAVCDLAHAAGAKVVLDNVFATPILQQGMPLGADVVVYSGTKHIDGQGRVLGGAILGDK

EYIDGPVQKLMRHTGPALSPFNAWTLLKGLETMALRVQHQNSSAHRIAEFLEGHSAVSWV

KYPFLESHPQYDLAKRQMTGGGTVVTFELAGATKERAFEVLDKLQIVDISNNLGDAKSLI

THPATTTHRAMGPEGRAAIGLGDGVVRISVGLEGTEDLIADLDRALG

>mab:MAB_4242c K10764 O-succinylhomoserine sulfhydrylase [EC:2.5.1.-] | (RefSeq) Probable O-succinylhomoserine sulfhydrylase MetZ (A)

MTQTPSGGSSVRIPKALPDGVSQATIGVRGGLLRSEFEETAEAMYLTSGYVYESAAAAER

AFTGEVDRYVYSRYGNPTISMFEERLRLIEGAEAAFATATGMSAVFTALGALLGAGDRLV

AARSLFGSCFVVCNEILPRWGVETVFVDGEDISQWEEALSVPTQAVFFETPSNPMQSLVD

IEAVCTLAHASGAKVVLDNVFATPLLQQGIPLGADVVVYSGTKHIDGQGRVLGGAILGET

EYIEGPVKTLMRHTGPALSPFNAWTLVKGLETLDLRVRHANDSAYKIAQFLEQHPAVRWV

RYPFLGTHPQYELAKRQMRGGGTVVTFELDADGDAGKQRAFQVLDGTSLIDISNNLGDSK

SLITHPATTTHRAMGPEGRAAIGLSDGVVRLSVGLESTDDLIADLERALG

>mabb:MASS_4278 K10764 O-succinylhomoserine sulfhydrylase [EC:2.5.1.-] | (GenBank) O-succinylhomoserine sulfhydrylase (A)

MTEDQKDIPAVSDCLRPSGSSVRIPKALPDGVSQATIGVRGGLLRSEFEETAEAMYLTSG

YVYESAAAAERAFTGEVDRYVYSRYGNPTISMFEERLRLIEGAEAAFATATGMSAVFTAL

GALLGAGDRLVAARSLFGSCFVVCNEILPRWGVETVFVDGEDISQWEEALSVPTQAVFFE

TPSNPMQSLVDIEAVCTLAHASGAKVVLDNVFATPLLQQGIPLGADVVVYSGTKHIDGQG

RVLGGAILGETEYIEGPVKTLMRHTGPALSPFNAWTLVKGLETLDLRVRHANDSAYRIAQ

FLEQHPAVRWVRYPFLTTHPQYELAKRQMRGGGTVVTFELDADGDAGKQRAFQVLDGTSL

IDISNNLGDSKSLITHPATTTHRAMGPEGRAAIGLSDGVVRLSVGLESTDDLIADLERAL

G

>mmv:MYCMA_2362 K10764 O-succinylhomoserine sulfhydrylase [EC:2.5.1.-] | (GenBank) O-succinylhomoserine sulfhydrylase (A)

MSDCLRPSGSSVRIPKALPDGVSQATIGVRGGLLRSEFEETAEAMYLTSGYVYESAAAAE

RAFTGEVDRYVYSRYGNPTISMFEERLRLIEGAEAAFATATGMSAVFTALGALLGAGDRL

VAARSLFGSCFVVCNEILPRWGVETVFVDGEDISQWEEALSVPTQAVFFETPSNPMQSLV

DIEAVCTLAHASGAKVVLDNVFATPLLQQGIPLGADVVVYSGTKHIDGQGRVLGGAILGE

TEYIEGPVKTLMRHTGPALSPFNAWTLVKGLETLDLRVRHANDSAYRIAQFLEQHPAVRW

VRYPFLTTHPQYELAKRQMRGGGTVVTFELDADGDAGKQRAFQVLDGTSLIDISNNLGDS

KSLITHPATTTHRAMGPEGRAAIGLSDGVVRLSVGLESTDDLIADLERALG

>may:LA62_21560 K10764 O-succinylhomoserine sulfhydrylase [EC:2.5.1.-] | (GenBank) O-succinylhomoserine sulfhydrylase (A)

MTQTPSGGSSVRIPKALPDGVSQATIGVRGGLLRSEFEETAEAMYLTSGYVYESAAAAER

AFTGEVDRYVYSRYGNPTISMFEERLRLIEGAEAAFATATGMSAVFTALGALLGAGDRLV

AARSLFGSCFVVCNEILPRWGVETVFVDGEDISQWEEALSVPTQAVFFETPSNPMQSLVD

IEAVCTLAHASGAKVVLDNVFATPLLQQGIPLGADVVVYSGTKHIDGQGRVLGGAILGET

EYIEGPVKTLMRHTGPALSPFNAWTLVKGLETLDLRVRHANDSAYKIAQFLEQHPAVRWV

RYPFLGTHPQYELAKRQMRGGGTVVTFELDADGDAGKQRAFQVLDGTSLIDISNNLGDSK

SLITHPATTTHRAMGPEGRAAIGLSDGVVRLSVGLESTDDLIADLERALG

>mabo:NF82_21250 K10764 O-succinylhomoserine sulfhydrylase [EC:2.5.1.-] | (GenBank) O-succinylhomoserine sulfhydrylase (A)

MTQTPSGGSSVRIPKALPDGVSQATIGVRGGLLRSEFEETAEAMYLTSGYVYESAAAAER

AFTGEVDRYVYSRYGNPTISMFEERLRLIEGAEAAFATATGMSAVFTALGALLGAGDRLV

AARSLFGSCFVVCNEILPRWGVETVFVDGEDISQWEEALSVPTQAVFFETPSNPMQSLVD

IEAVCTLAHASGAKVVLDNVFATPLLQQGIPLGADVVVYSGTKHIDGQGRVLGGAILGET

EYIEGPVKTLMRHTGPALSPFNAWTLVKGLETLDLRVRHANDSAYKIAQFLEQHPAVRWV

RYPFLGTHPQYELAKRQMRGGGTVVTFELDADGDAGKQRAFQVLDGTSLIDISNNLGDSK

SLITHPATTTHRAMGPEGRAAIGLSDGVVRLSVGLESTDDLIADLERALG

>mabl:MMASJCM_4329 K10764 O-succinylhomoserine sulfhydrylase [EC:2.5.1.-] | (GenBank) O-acetylhomoserine sulfhydrylase O-succinylhomoserine sulfhydrylase (A)

MTEDQKDIPAVSDCLRPSGSSVRIPKALPDGVSQATIGVRGGLLRSEFEETAEAMYLTSG

YVYESAAAAERAFTGEVDRYVYSRYGNPTISMFEERLRLIEGAEAAFATATGMSAVFTAL

GALLGAGDRLVAARSLFGSCFVVCNEILPRWGVETVFVDGEDISQWEEALSVPTQAVFFE

TPSNPMQSLVDIEAVCTLAHASGAKVVLDNVFATPLLQQGIPLGADVVVYSGTKHIDGQG

RVLGGAILGETEYIEGPVKTLMRHTGPALSPFNAWTLVKGLETLDLRVRHANDSAYKIAQ

FLEQHPAVRWVRYPFLATHPQYELAKRQMRGGGTVVTFELDADGDAGKQRAFQVLDGTSL

IDISNNLGDSKSLITHPATTTHRAMGPEGRAAIGLSDGVVRLSVGLESTDDLIADLERAL

G

>maz:LA61_21455 K10764 O-succinylhomoserine sulfhydrylase [EC:2.5.1.-] | (GenBank) O-succinylhomoserine sulfhydrylase (A)

MTQTPSGGSSVRIPKALPDGVSQATIGVRGGLLRSEFEETAEAMYLTSGYVYESAAAAER

AFTGEVDRYVYSRYGNPTISMFEERLRLIEGAEAAFATATGMSAVFTALGALLGAGDRLV

AARSLFGSCFVVCNEILPRWGVETVFVDGEDISQWEEALSVPTQAVFFETPSNPMQSLVD

IEAVCTLAHASGAKVVLDNVFATPLLQQGIPLGADVVVYSGTKHIDGQGRVLGGAILGET

EYIEGPVKTLMRHTGPALSPFNAWTLVKGLETLDLRVRHANDSAYKIAQFLEQHPAVRWV

RYPFLGTHPQYELAKRQMRGGGTVVTFELDADGDAGKQRAFQVLDGTSLIDISNNLGDSK

SLITHPATTTHRAMGPEGRAAIGLSDGVVRLSVGLESTDDLIADLERALG

>mak:LH56_02975 K10764 O-succinylhomoserine sulfhydrylase [EC:2.5.1.-] | (GenBank) O-succinylhomoserine sulfhydrylase (A)

MSDCLRPSGSSVRIPKALPDGVSQATIGVRGGLLRSEFEETAEAMYLTSGYVYESAAAAE

RAFTGEVDRYVYSRYGNPTISMFEERLRLIEGAEAAFATATGMSAVFTALGALLGAGDRL

VAARSLFGSCFVVCNEILPRWGVETVFVDGEDISQWEEALSVPTQAVFFETPSNPMQSLV

DIEAVCTLAHASGAKVVLDNVFATPLLQQGIPLGADVVVYSGTKHIDGQGRVLGGAILGE

TEYIEGPVKTLMRHTGPALSPFNAWTLVKGLETLDLRVRHANDSAYKIAQFLEQHPAVRW

VRYPFLATHPQYELAKRQMRGGGTVVTFELDADGDAGKQRAFQVLDGTSLIDISNNLGDS

KSLITHPATTTHRAMGPEGRAAIGLSDGVVRLSVGLESTDDLIADLERALG

>mys:NF92_03030 K10764 O-succinylhomoserine sulfhydrylase [EC:2.5.1.-] | (GenBank) O-succinylhomoserine sulfhydrylase (A)

MSDCLRPSGSSVRIPKALPDGVSQATIGVRGGLLRSEFEETAEAMYLTSGYVYESAAAAE

RAFTGEVDRYVYSRYGNPTISMFEERLRLIEGAEAAFATATGMSAVFTALGALLGAGDRL

VAARSLFGSCFVVCNEILPRWGVETVFVDGEDISQWEEALSVPTQAVFFETPSNPMQSLV

DIEAVCTLAHASGAKVVLDNVFATPLLQQGIPLGADVVVYSGTKHIDGQGRVLGGAILGE

TEYIEGPVKTLMRHTGPALSPFNAWTLVKGLETLDLRVRHANDSAYRIAQFLEQHPAVRW

VRYPFLTTHPQYELAKRQMRGGGTVVTFELDADGDAGKQRAFQVLDGTSLIDISNNLGDS

KSLITHPATTTHRAMGPEGRAAIGLSDGVVRLSVGLESTDDLIADLERALG

>myc:NF90_03030 K10764 O-succinylhomoserine sulfhydrylase [EC:2.5.1.-] | (GenBank) O-succinylhomoserine sulfhydrylase (A)

MSDCLRPSGSSVRIPKALPDGVSQATIGVRGGLLRSEFEETAEAMYLTSGYVYESAAAAE

RAFTGEVDRYVYSRYGNPTISMFEERLRLIEGAEAAFATATGMSAVFTALGALLGAGDRL

VAARSLFGSCFVVCNEILPRWGVETVFVDGEDISQWEEALSVPTQAVFFETPSNPMQSLV

DIEAVCTLAHASGAKVVLDNVFATPLLQQGIPLGADVVVYSGTKHIDGQGRVLGGAILGE

TEYIEGPVKTLMRHTGPALSPFNAWTLVKGLETLDLRVRHANDSAYRIAQFLEQHPAVRW

VRYPFLTTHPQYELAKRQMRGGGTVVTFELDADGDAGKQRAFQVLDGTSLIDISNNLGDS

KSLITHPATTTHRAMGPEGRAAIGLSDGVVRLSVGLESTDDLIADLERALG

>mmc:Mmcs_0526 K10764 O-succinylhomoserine sulfhydrylase [EC:2.5.1.-] | (GenBank) O-succinylhomoserine sulfhydrylase (EC:2.5.1.48) (A)

MSSEVPSRTQGADKPSVRRPAELPEGVGQATIGVRGGLLRSGFEETAEALYLTSGYVYAS

AAEAEKAFTGEIDRYVYSRYGNPTISMFEERLRLIEGAPACFATATGMAAVFTSLGALLG

AGDRLVAARSLFGSCFVVCSEILPRWGVETVFVDGDDLSQWEEALSVPTQAVFFETPSNP

MQSLVDIAAVTELAHAAGAKVVLDNVFATPLLQQGFPLGVDVVVYSGTKHIDGQGRVLGG

AILGSQEYIDGPVQKLMRHTGPAISAFNAWTLLKGLETLAVRVDYSNRSAERIAEFLEGH

SSVSWVKYPFLESHPQYDLAKRQMRGGGTVVTFELAGGKERAFEVLDKLQIIDISNNLGD

AKSLITHPATTTHRAMGPEGRAAIGLGDGVVRVSVGLEDTDDLIRDLDRALG

>mkm:Mkms_0538 K10764 O-succinylhomoserine sulfhydrylase [EC:2.5.1.-] | (GenBank) O-succinylhomoserine sulfhydrylase (EC:2.5.1.48) (A)

MSSEVPSRTQGADKPSVRRPAELPEGVGQATIGVRGGLLRSGFEETAEALYLTSGYVYAS

AAEAEKAFTGEIDRYVYSRYGNPTISMFEERLRLIEGAPACFATATGMAAVFTSLGALLG

AGDRLVAARSLFGSCFVVCSEILPRWGVETVFVDGDDLSQWEEALSVPTQAVFFETPSNP

MQSLVDIAAVTELAHAAGAKVVLDNVFATPLLQQGFPLGVDVVVYSGTKHIDGQGRVLGG

AILGSQEYIDGPVQKLMRHTGPAISAFNAWTLLKGLETLAVRVDYSNRSAERIAEFLEGH

SSVSWVKYPFLESHPQYDLAKRQMRGGGTVVTFELAGGKERAFEVLDKLQIIDISNNLGD

AKSLITHPATTTHRAMGPEGRAAIGLGDGVVRVSVGLEDTDDLIRDLDRALG

>mjl:Mjls_0516 K10764 O-succinylhomoserine sulfhydrylase [EC:2.5.1.-] | (GenBank) O-succinylhomoserine sulfhydrylase (EC:2.5.1.48) (A)

MSSEVPSRTQGADKPSVRRPAELPEGVGQATIGVRGGLLRSGFEETAEALYLTSGYVYAS

AAEAEKAFTGEIDRYVYSRYGNPTISMFEERLRLIEGAPACFATATGMAAVFTSLGALLG

AGDRLVAARSLFGSCFVVCSEILPRWGVETVFVDGDDLSQWEEALSVPTQAVFFETPSNP

MQSLVDIAAVTELAHAAGAKVVLDNVFATPLLQQGLPLGVDVVVYSGTKHIDGQGRVLGG

AILGSQEYIDGPVQKLMRHTGPAISAFNAWTLLKGLETLAVRVDYSNRSAERIAEFLEGH

SSVSWVKYPFLESHPQYDLAKRQMRGGGTVVTFELAGGKERAFEVLDKLQIIDISNNLGD

AKSLITHPATTTHRAMGPEGRAAIGLGDGVVRVSVGLEDTDDLIRDLDRALG

>mjd:JDM601_0369 K10764 O-succinylhomoserine sulfhydrylase [EC:2.5.1.-] | (GenBank) metZ; O-succinylhomoserine sulfhydrylase MetZ (A)

MRVPKPLPDGVSAATVGVRGGILRSQFDETTEALFLSSGYIYDSAAAAEQAFTGEVDRFV

YSRYGNPTVSMFEERLRLIEGAPAAFATASGMAAVFVSLGALLGAGDRLVAARSLFGSCF

VVCNEILPRWGVETVFVDGDDMAQWEQALSVPTTAVFFETPSNPMQSLVDIAAVTELAHA

AGAKVVLDNVFATPLLQQGFPLGVDVVVYSGTKHLDGQGRVLGGAILGEQEYIDGPVQNL

MRHTGPAISAFNAWLLLKGLETLAVRVDYANAAALRIAEYLEQHRAVRWVRYPFLPSHPQ

YDLARRQMSGGGTVITFELRTLRDAPDGTAKQRAFELLDKLRLIDISNNLGDVKSLITHP

ATTTHRAMGPEGRAAIGLGDGVVRISVGLEGVDDLIADLDQALG

>mmi:MMAR_0688 K10764 O-succinylhomoserine sulfhydrylase [EC:2.5.1.-] | (GenBank) metZ; O-succinylhomoserine sulfhydrylase, MetZ (A)

MTDDRSVPEAERPKNPRKLPDGVSQATIGVRGGMLRSGFDETAEALYLTSGYVYESAAMA

EQSFAGELDHFLYSRYGNPTVAMFEERLRLIEAAPAVFATASGMAAVFTALGALLGAGDR

LVASRSLFGSCFVVCNEILPRWGVETVFVDGDDLEQWEQALSVPTEAVFFETPSNPMQSL

VDIAAVTELAHAAGAKVVLDNVFATPLLQQGIPLGVDVVVYSGTKHIDGQGRVLGGAILG

DQDYIDGPVQKLMRHTGPALSAFNAWILLKGLETLAIRVQHSNSSALRIAEFLQGHPAVR

WVRYPYLPSHPQYDLAKRQMSGGGTVVTFALDAPESAAKRRAFEVLDKLRLIDISNNLGD

TKSLITHPATTTHRAMGPEGRAAIGLGDAVVRISVGLEGAEDLIADIDQALG

>mrh:MycrhN_1298 K10764 O-succinylhomoserine sulfhydrylase [EC:2.5.1.-] | (GenBank) O-succinylhomoserine sulfhydrylase (A)

MSPDGVPSVRQPAELPDGVSQATIGVRGGLLRSGFEETAEAMYLTSGYVYSSAAEAEKAF

TGDIDRYVYSRYGNPTISMFEERLRLIEGAPACFATATGMAAVFTSLGALLGAGDRLVAA

RSLFGSCFVVCNEILPRWGVETVFVDGDDLSQWEEALSVPTKAVFFETPSNPMQQLVDIS

AVCELAHAAGAKVVLDNVFATPILQQGMPLGADVVVYSGTKHIDGQGRVLGGAILGDKQY

IDEPVQKLMRHTGPALSPFNAWTLLKGLETLAIRVDYQNASAHRVAEFLESHPAVSWVRY

PFLESHPQYDLAKRQMTGGGTVVTFELKGGTKDRAFEVLDKLRIVDISNNLGDSKSLITH

PATTTHRAMGPEGRAAIGLGDGVVRISVGLEGTEDLIGDLDQALG

>mmm:W7S_23505 K10764 O-succinylhomoserine sulfhydrylase [EC:2.5.1.-] | (GenBank) O-succinylhomoserine sulfhydrylase (A)

MTPADHDSVRTPAPLPEGVSQATIGVRGGLLRSGFDETAEALYLTSGYVYETAAVAEQSF

TGEVDRFVYSRYGNPTVTMFEERLRLLEGAPAAFATASGMAAVFTSLGALLGAGDRLVAS

RSLFGSCFVVCNEILPRWGVETVFVDGDDLSQWEQALSVPTTAVFFETPSNPMQSLVDIA

AVTELAHAAGAKVVLDNVFATPLLQQGVPLGVDVVVYSGTKHIDGQGRVLGGAILGDKEY

IDGPVQKLMRHTGPAMSAFNAWVLLKGLETMAIRVEHSNSSAYRIAEFLETHPAVSWVRY

PYLSSHPQYDLAKRQMSGGGTVITFALDCPDSRAKQRAFEVLDKLTLIDISNNLGDAKSL

VTHPATTTHRAMGPEGRAAIGLGDGVVRISVGLEGTDDLIADIDRALS

>mcb:Mycch_0456 K10764 O-succinylhomoserine sulfhydrylase [EC:2.5.1.-] | (GenBank) O-succinylhomoserine sulfhydrylase (A)

MSEQEVPSVRRPIELPAGVSQSTIGVRGGLLRSGFEETAEALYLTSGYVYESAAAAEKAF

TGEIDRYVYSRYGNPTIQMFEERLRLLEGAPACFATATGMSAVFTALGALLGAGDRLVAA

RSLFGSCFVVCNEILPRWGVETVFVDGEDLSQWEEALSVPTQAVFFETPSNPMQSLVDIA

AVCDVAHAAGAKVVLDNVFATPILQQGMPLGADVVVYSGTKHIDGQGRVLGGAILGSREY

IDEPVQKLMRHTGPALSPFNAWTLLKGLETLALRVQYQNSSAHRIAEFLEQHPGVSWVRY

PFLESHPQYDLAKRQMTGGGTVVTFELKGGTKERAFEVLDKLAIIDISNNLGDAKSLITH

PATTTHRAMGPDGRAAIGLGDGVVRVSIGLESTDDLIADLDRALS

>mli:MULP_00687 K10764 O-succinylhomoserine sulfhydrylase [EC:2.5.1.-] | (GenBank) metZ; O-succinylhomoserine sulfhydrylase, MetZ (EC:4.2.99.-) (A)

MTDDRSVPEAERPKNPRKLPDGVSQATIGVRGGMLRSGFDETAETLYLTSGYVYESAAMA

EQSFAGELDHFLYSRYGNPTVAMFEERLRLIEAAPAVFATASGMAAVFTALGALLGAGDR

LVASRSLFGSCFVVCNEILPRWGVETVFVDGDDLEQWEQALSVPTEAVFFETPSNPMQSL

VDIAAVTELAHAAGAKVVLDNVFATPLLQQGIPLGVDVVVYSGTKHIDGQGRVLGGAILG

DQDYIDGPVQKLMRHTGPALSAFNAWILLKGLETLAIRVQHSNSSALRIAEFLQGHPAVR

WVRYPYLPSHPQYDLAKRQMSGGGTVVTFALDAPESAAKRRAFEVLDKLRLIDISNNLGD

TKSLITHPATTTHRAMGPEGRAAIGLGDAVVRISVGLEGAEDLIADIDQALG

>mkn:MKAN_17720 K10764 O-succinylhomoserine sulfhydrylase [EC:2.5.1.-] | (GenBank) O-succinylhomoserine sulfhydrylase (A)

MTDHPAIRTPKVLPDGVGQATIGVRGGLLRSGFDETAEAMYLTSGYVYESAAAAEQSFSG

ELDHFVYSRYGNPTVAMFEERLRLIEGAPAAFATASGMAAVFTSLGALLAAGDRLVAARS

LFGSCFVVCNEILPRWGVETVFVDGDDLSQWEQALADPSKPTRAVFFETPANPMQSLVDI

AAVTELAHAAGAKVVLDNVFATPLLQQGFPLGVDVVVYSGTKHIDGQGRVLGGAILGDKE

YIDGPVQKLMRHTGPAMSAFNAWVLLKGLETLAVRVECSNSSALRIAAFLEAHPAVSWVR

YPFLPSHPQYNLAKRQMSGGGTVVTFALDAPESAAKQRAFEVLDKLALIDISNNLGDAKS

LVTHPATTTHRAMGPEGRAAIGLGDGVVRISVGLEGADDLIADLDRALG

>mks:LG40_17550 K10764 O-succinylhomoserine sulfhydrylase [EC:2.5.1.-] | (GenBank) O-succinylhomoserine sulfhydrylase (A)

MTDHPAIRTPKVLPDGVGQATIGVRGGLLRSGFDETAEAMYLTSGYVYESAAAAEQSFSG

ELDHFVYSRYGNPTVAMFEERLRLIEGAPAAFATASGMAAVFTSLGALLAAGDRLVAARS

LFGSCFVVCNEILPRWGVETVFVDGDDLSQWEQALADPSKPTRAVFFETPANPMQSLVDI

AAVTELAHAAGAKVVLDNVFATPLLQQGFPLGVDVVVYSGTKHIDGQGRVLGGAILGDKE

YIDGPVQKLMRHTGPAMSAFNAWVLLKGLETLAVRVECSNSSALRIAAFLEAHPAVSWVR

YPFLPSHPQYNLAKRQMSGGGTVVTFALDAPESAAKQRAFEVLDKLALIDISNNLGDAKS

LVTHPATTTHRAMGPEGRAAIGLGDGVVRISVGLEGADDLIADLDRALG

>mki:LH54_17650 K10764 O-succinylhomoserine sulfhydrylase [EC:2.5.1.-] | (GenBank) O-succinylhomoserine sulfhydrylase (A)

MTDHPAIRTPKVLPDGVGQATIGVRGGLLRSGFDETAEAMYLTSGYVYESAAAAEQSFSG

ELDHFVYSRYGNPTVAMFEERLRLIEGAPAAFATASGMAAVFTSLGALLAAGDRLVAARS

LFGSCFVVCNEILPRWGVETVFVDGDDLSQWEQALADPSKPTRAVFFETPANPMQSLVDI

AAVTELAHAAGAKVVLDNVFATPLLQQGFPLGVDVVVYSGTKHIDGQGRVLGGAILGDKE

YIDGPVQKLMRHTGPAMSAFNAWVLLKGLETLAVRVECSNSSALRIAAFLEAHPAVSWVR

YPFLPSHPQYNLAKRQMSGGGTVVTFALDAPESAAKQRAFEVLDKLALIDISNNLGDAKS

LVTHPATTTHRAMGPEGRAAIGLGDGVVRISVGLEGADDLIADLDRALG

>mne:D174_04675 K10764 O-succinylhomoserine sulfhydrylase [EC:2.5.1.-] | (GenBank) O-succinylhomoserine sulfhydrylase (A)

MSDVPSVRIPAPLPDGVSQATIGVRGGLLRSGFEETAEALYLTSGYVYESAADAERAFTG

EIDRYVYSRYGNPTISMFEERLRLIEGAPAAFATATGMAAVFTALGALLGAGDRLVAARS

LFGSCFVVCNEILPRWGVETVFVDGDDLSQWEEALSVPTQAVFFETPSNPMQSLVDIAAV

AELAHAAGAKVVLDNVFATPLLQQGIPLGADVVVYSGTKHIDGQGRVLGGAILGDKEYID

GPVQKLMRHTGPAISAFNAWTLLKGLETLAVRVDYANRSAQRVAEFLEQQSGVNWVKYPF

LQSHPQYELAQRQMRGGGTVVTFEVDGGKDAAFKLLDKLRVIDISNNLGDAKSLITHPAT

TTHRAMGPEGRAAIGLGDGVVRVSVGLEGTEDLIADLGQALS

>myv:G155_03885 K10764 O-succinylhomoserine sulfhydrylase [EC:2.5.1.-] | (GenBank) O-succinylhomoserine sulfhydrylase (A)

MTDIPSVRIPAALPEGVSQATIGVRGGLLRSEFEETAEAMYLTSGYVYESASAAEKAFTG

EIDRFVYSRYGNPTISMFEERLRLIEDAPACFATATGMAAVFTALGALLGAGDRLVAARS

LFGSCFVVCNEILPRWGVETVFVDGDDLAQWEEALSVPTTAVFFETPSNPMQSLVDIAAV

SELAHAAGAKVVLDNVFATPLLQQGLPLGADVVVYSGTKHIDGQGRVLGGAILGDKEYID

GPVQKLMRHTGPAISAFNAWTLLKGLETLHVRVDYANRSAQRIAEFLEGHPSVSWVKYPF

LKSHPQYDLAQRQMRGGGTVITFELNATDGKARAFEVLDKLKVVDISNNLGDAKTLITHP

ATTTHRAMGPEGRAAIGLGDGVVRISIGLEGTEDLIADLDQALS

>mye:AB431_03060 K10764 O-succinylhomoserine sulfhydrylase [EC:2.5.1.-] | (GenBank) O-succinylhomoserine sulfhydrylase (A)

MTDEVPSVRIPAPLPDGVSQATLGVRGGLLRSEFEETAEGMYLTSGYVYSSAAEAEKAFT

GEIDRYVYSRYGNPTISMFEERLRLIEGAPAAFATASGMAAVFTSLGALLGAGDRLVAAR

SLFGSCFVVCNEILPRWGVETVFVDGDDLSQWEEALSKPTQAVFFETPSNPMQSLVDIAA

VSEMAHSAGAKVVLDNVFATPLLQQGFPLGADVVVYSGTKHIDGQGRVLGGAILGDREYI

DGPVQKLMRHTGPALSAFNAWVLLKGLETMAVRVDYSNRSAQRIAEFLQAQPGVNWVKYP

FLESHPQFDLAKRQMRGGGTVVTFELDGGKSRAFEVLDKLQIIDISNNLGDAKTLITHPA

TTTHRAMGPEGRASIGLGDGVVRISVGLEGTEDLITDLERALG

>mgo:AFA91_01570 K10764 O-succinylhomoserine sulfhydrylase [EC:2.5.1.-] | (GenBank) O-succinylhomoserine sulfhydrylase (A)

MTDDIPSVRIPAPLPDGVSQATIGVRGGLLRSGFEETAEALYLTSGYVYETAEAAEKAFT

GDIDRYVYSRYGNPTISMFEERLRLIEGAPACFATATGMAAVFTALGALLGAGDRLVAAR

SLFGSCFVVCNEILPRWGVETVFVDGDDLSQWEQALSEPTQAVFFETPSNPMQTLVDIAA

VSEMAHAAGAKVVLDNVFATPLLQQGMPLGADVVVYSGTKHIDGQGRVLGGAILGEREYI

DGPVQKLMRHTGPAISAFNAWTLLKGLETMAVRVDYSNRSAQRVAEFLEGHSAVRWVKYP

LLESHPQYELAKRQMRGGGTVVTFELDGADGKARAFEVLDKLRVIDISNNLGDAKSLITH

PATTTHRAMGPEGRAAIGLGDGVVRISVGLEGTEDLIADLDQALS

>mft:XA26_07300 K10764 O-succinylhomoserine sulfhydrylase [EC:2.5.1.-] | (GenBank) O-acetylhomoserine sulfhydrylase (EC:2.5.1.49 2.5.1.48) (A)

MTDIPSVRIPAALPEGVSQATIGVRGGLLRSEFEETAEAMYLTSGYVYESASAAEKAFTG

EIDRFVYSRYGNPTISMFEERLRLIEDAPACFATATGMAAVFTALGALLGAGDRLVAARS

LFGSCFVVCNEILPRWGVETVFVDGDDLAQWEEALSVPTTAVFFETPSNPMQSLVDIAAV

SELAHAAGAKVVLDNVFATPLLQQGLPLGADVVVYSGTKHIDGQGRVLGGAILGDKEYID

GPVQKLMRHTGPAISAFNAWTLLKGLETLHVRVDYANRSAQRIAEFLEGHPSVSWVKYPF

LKSHPQYDLAQRQMRGGGTVITFELNATDGKARAFEVLDKLKVVDISNNLGDAKTLITHP

ATTTHRAMGPEGRAAIGLGDGVVRISIGLEGTEDLIADLDQALS

>mhad:B586_00380 K10764 O-succinylhomoserine sulfhydrylase [EC:2.5.1.-] | (GenBank) O-succinylhomoserine sulfhydrylase (A)

MTDDGSVRTPKALPDGISQATMGVRGGLLRSQFEETAEAMYLTSGYVYESAAVAEKSFTG

ELDHFVYSRYGNPTVTMFEERLRLIEGAPAAFATASGMAAVFTSLGALLAAGDRLVAARS

LFGSCFVVCNEILPRWGVETVFVDGDDLAQWEQALSVPTQAVFFETPSNPMQSLVDIAAV

TELAHATGAKVVLDNVFATPLLQQGFPLGADVVVYSGTKHIDGQGRVLGGAILGDREYID

GPVQKLMRHTGPAMSAFNAWILLKGLETLAIRVEHCNSSAHRIAEFLQRHPAVSWVRYPF

LVSHPQYDLAKRQMSGGGTVVTFALNSPENAAKQRAFEVLDKLRLIDISNNLGDAKSLIT

HPATTTHRAMGPEGRAAIGLGDGVVRISVGLEDTDDLIADIDQALS

Rv0450c Homologs

>mtu:Rv0450c K06994 putative drug exporter of the RND superfamily | (RefSeq) mmpL4; transmembrane transport protein MmpL4 (A)

MSTKFANDSNTNARPEKPFIARMIHAFAVPIILGWLAVCVVVTVFVPSLEAVGQERSVSL

SPKDAPSFEAMGRIGMVFKEGDSDSFAMVIIEGNQPLGDAAHKYYDGLVAQLRADKKHVQ

SVQDLWGDPLTAAGVQSNDGKAAYVQLSLAGNQGTPLANESVEAVRSIVESTPAPPGIKA

YVTGPSALAADMHHSGDRSMARITMVTVAVIFIMLLLVYRSIITVVLLLITVGVELTAAR

GVVAVLGHSGAIGLTTFAVSLLTSLAIAAGTDYGIFIIGRYQEARQAGEDKEAAYYTMYR

GTAHVILGSGLTIAGATFCLSFARMPYFQTLGIPCAVGMLVAVAVALTLGPAVLHVGSRF

GLFDPKRLLKVRGWRRVGTVVVRWPLPVLVATCAIALVGLLALPGYKTSYNDRDYLPDFI

PANQGYAAADRHFSQARMKPEILMIESDHDMRNPADFLVLDKLAKGIFRVPGISRVQAIT

RPEGTTMDHTSIPFQISMQNAGQLQTIKYQRDRANDMLKQADEMATTIAVLTRMHSLMAE

MASTTHRMVGDTEEMKEITEELRDHVADFDDFWRPIRSYFYWEKHCYGIPICWSFRSIFD

ALDGIDKLSEQIGVLLGDLREMDRLMPQMVAQIPPQIEAMENMRTMILTMHSTMTGIFDQ

MLEMSDNATAMGKAFDAAKNDDSFYLPPEVFKNKDFQRAMKSFLSSDGHAARFIILHRGD

PQSPEGIKSIDAIRTAAEESLKGTPLEDAKIYLAGTAAVFHDISEGAQWDLLIAAISSLC

LIFIIMLIITRAFIAAAVIVGTVALSLGASFGLSVLLWQHILAIHLHWLVLAMSVIVLLA

VGSDYNLLLVSRFKQEIGAGLKTGIIRSMGGTGKVVTNAGLVFAVTMASMAVSDLRVIGQ

VGTTIGLGLLFDTLIVRSFMTPSIAALLGRWFWWPLRVRSRPARTPTVPSETQPAGRPLA

MSSDRLG

>mtv:RVBD_0450c K06994 putative drug exporter of the RND superfamily | (GenBank) transmembrane transporter MmpL4 (A)

MSTKFANDSNTNARPEKPFIARMIHAFAVPIILGWLAVCVVVTVFVPSLEAVGQERSVSL

SPKDAPSFEAMGRIGMVFKEGDSDSFAMVIIEGNQPLGDAAHKYYDGLVAQLRADKKHVQ

SVQDLWGDPLTAAGVQSNDGKAAYVQLSLAGNQGTPLANESVEAVRSIVESTPAPPGIKA

YVTGPSALAADMHHSGDRSMARITMVTVAVIFIMLLLVYRSIITVVLLLITVGVELTAAR

GVVAVLGHSGAIGLTTFAVSLLTSLAIAAGTDYGIFIIGRYQEARQAGEDKEAAYYTMYR

GTAHVILGSGLTIAGATFCLSFARMPYFQTLGIPCAVGMLVAVAVALTLGPAVLHVGSRF

GLFDPKRLLKVRGWRRVGTVVVRWPLPVLVATCAIALVGLLALPGYKTSYNDRDYLPDFI

PANQGYAAADRHFSQARMKPEILMIESDHDMRNPADFLVLDKLAKGIFRVPGISRVQAIT

RPEGTTMDHTSIPFQISMQNAGQLQTIKYQRDRANDMLKQADEMATTIAVLTRMHSLMAE

MASTTHRMVGDTEEMKEITEELRDHVADFDDFWRPIRSYFYWEKHCYGIPICWSFRSIFD

ALDGIDKLSEQIGVLLGDLREMDRLMPQMVAQIPPQIEAMENMRTMILTMHSTMTGIFDQ

MLEMSDNATAMGKAFDAAKNDDSFYLPPEVFKNKDFQRAMKSFLSSDGHAARFIILHRGD

PQSPEGIKSIDAIRTAAEESLKGTPLEDAKIYLAGTAAVFHDISEGAQWDLLIAAISSLC

LIFIIMLIITRAFIAAAVIVGTVALSLGASFGLSVLLWQHILAIHLHWLVLAMSVIVLLA

VGSDYNLLLVSRFKQEIGAGLKTGIIRSMGGTGKVVTNAGLVFAVTMASMAVSDLRVIGQ

VGTTIGLGLLFDTLIVRSFMTPSIAALLGRWFWWPLRVRSRPARTPTVPSETQPAGRPLA

MSSDRLG

>mtc:MT0466 K06994 putative drug exporter of the RND superfamily | (GenBank) membrane protein, MmpL family (A)

MSTKFANDSNTNARPEKPFIARMIHAFAVPIILGWLAVCVVVTVFVPSLEAVGQERSVSL

SPKDAPSFEAMGRIGMVFKEGDSDSFAMVIIEGNQPLGDAAHKYYDGLVAQLRADKKHVQ

SVQDLWGDPLTAAGVQSNDGKAAYVQLSLAGNQGTPLANESVEAVRSIVESTPAPPGIKA

YVTGPSALAADMHHSGDRSMARITMVTVAVIFIMLLLVYRSIITVVLLLITVGVELTAAR

GVVAVLGHSGAIGLTTFAVSLLTSLAIAAGTDYGIFIIGRYQEARQAGEDKEAAYYTMYR

GTAHVILGSGLTIAGATFCLSFARMPYFQTLGIPCAVGMLVAVAVALTLGPAVJHVGSRF

GLFDPKRLLKVRGWRRVGTVVVRWPLPVLVATCAIALVGLLALPGYKTSYNDRDYLPDFI

PANQGYAAADRHFSQARMKPEILMIESDHDMRNPADFLVLDKLAKGIFRVPGISRVQAIT

RPEGTTMDHTSIPFQISMQNAGQLQTIKYQRDRANDMLKQADEMATTIAVLTRMHSLMAE

MASTTHRMVGDTEEMKEITEELRDHVADFDDFWRPIRSYFYWEKHCYGIPICWSFRSIFD

ALDGIDKLSEQIGVLLGDLREMDRLMPQMVAQIPPQIEAMENMRTMILTMHSTMTGIFDQ

MLEMSDNATAMGKAFDAAKNDDSFYLPPEVFKNKDFQRAMKSFLSSDGHAARFIILHRGD

PQSPEGIKSIDAIRTAAEESLKGTPLEDAKIYLAGTAAVFHDISEGAQWDLLIAAISSLC

LIFIIMLIITRAFIAAAVIVGTVALSLGASFGLSVLLWQHILAIHLHWLVLAMSVIVLLA

VGSDYNLLLVSRFKQEIGAGLKTGIIRSMGGTGKVVTNAGLVFAVTMASMAVSDLRVIGQ

VGTTIGLGLLFDTLIVRSFMTPSIAALLGRWFWWPLRVRSRPARTPTVPSETQPAGRPLA

MSSDRLG

>mra:MRA_0455 K06994 putative drug exporter of the RND superfamily | (GenBank) mmpL4; transmembrane transport protein MmpL4 (A)

MSTKFANDSNTNARPEKPFIARMIHAFAVPIILGWLAVCVVVTVFVPSLEAVGQERSVSL

SPKDAPSFEAMGRIGMVFKEGDSDSFAMVIIEGNQPLGDAAHKYYDGLVAQLRADKKHVQ

SVQDLWGDPLTAAGVQSNDGKAAYVQLSLAGNQGTPLANESVEAVRSIVESTPAPPGIKA

YVTGPSALAADMHHSGDRSMARITMVTVAVIFIMLLLVYRSIITVVLLLITVGVELTAAR

GVVAVLGHSGAIGLTTFAVSLLTSLAIAAGTDYGIFIIGRYQEARQAGEDKEAAYYTMYR

GTAHVILGSGLTIAGATFCLSFARMPYFQTLGIPCAVGMLVAVAVALTLGPAVLHVGSRF

GLFDPKRLLKVRGWRRVGTVVVRWPLPVLVATCAIALVGLLALPGYKTSYNDRDYLPDFI

PANQGYAAADRHFSQARMKPEILMIESDHDMRNPADFLVLDKLAKGIFRVPGISRVQAIT

RPEGTTMDHTSIPFQISMQNAGQLQTIKYQRDRANDMLKQADEMATTIAVLTRMHSLMAE

MASTTHRMVGDTEEMKEITEELRDHVADFDDFWRPIRSYFYWEKHCYGIPICWSFRSIFD

ALDGIDKLSEQIGVLLGDLREMDRLMPQMVAQIPPQIEAMENMRTMILTMHSTMTGIFDQ

MLEMSDNATAMGKAFDAAKNDDSFYLPPEVFKNKDFQRAMKSFLSSDGHAARFIILHRGD

PQSPEGIKSIDAIRTAAEESLKGTPLEDAKIYLAGTAAVFHDISEGAQWDLLIAAISSLC

LIFIIMLIITRAFIAAAVIVGTVALSLGASFGLSVLLWQHILAIHLHWLVLAMSVIVLLA

VGSDYNLLLVSRFKQEIGAGLKTGIIRSMGGTGKVVTNAGLVFAVTMASMAVSDLRVIGQ

VGTTIGLGLLFDTLIVRSFMTPSIAALLGRWFWWPLRVRSRPARTPTVPSETQPAGRPLA

MSSDRLG

>mtf:TBFG_10456 K06994 putative drug exporter of the RND superfamily | (GenBank) transmembrane transport protein mmpL4 (A)

MSTKFANDSNTNARPEKPFIARMIHAFAVPIILGWLAVCVVVTVFVPSLEAVGQERSVSL

SPKDAPSFEAMGRIGMVFKEGDSDSFAMVIIEGNQPLGDAAHKYYDGLVAQLRADKKHVQ

SVQDLWGDPLTAAGVQSNDGKAAYVQLSLAGNQGTPLANESVEAVRSIVESTPAPPGIKA

YVTGPSALAADMHHSGDRSMARITMVTVAVIFIMLLLVYRSIITVVLLLITVGVELTAAR

GVVAVLGHSGAIGLTTFAVSLLTSLAIAAGTDYGIFIIGRYQEARQAGEDKEAAYYTMYR

GTAHVILGSGLTIAGATFCLSFARMPYFQTLGIPCAVGMLVAVAVALTLGPAVLHVGSRF

GLFDPKRLLKVRGWRRVGTVVVRWPLPVLVATCAIALVGLLALPGYKTSYNDRDYLPDFI

PANQGYAAADRHFSQARMKPEILMIESDHDMRNPADFLVLDKLAKGIFRVPGISRVQAIT

RPEGTTMDHTSIPFQISMQNAGQLQTIKYQRDRANDMLKQADEMATTIAVLTRMHSLMAE

MASTTHRMVGDTEEMKEITEELRDHVADFDDFWRPIRSYFYWEKHCYGIPICWSFRSIFD

ALDGIDKLSEQIGVLLGDLREMDRLMPQMVAQIPPQIEAMENMRTMILTMHSTMTGIFDQ

MLEMSDNATAMGKAFDAAKNDDSFYLPPEVFKNKDFQRAMKSFLSSDGHAARFIILHRGD

PQSPEGIKSIDAIRTAAEESLKGTPLEDAKIYLAGTAAVFHDISEGAQWDLLIAAISSLC

LIFIIMLIITRAFIAAAVIVGTVALSLGASFGLSVLLWQHILAIHLHWLVLAMSVIVLLA

VGSDYNLLLVSRFKQEIGAGLKTGIIRSMGGTGKVVTNAGLVFAVTMASMAVSDLRVIGQ

VGTTIGLGLLFDTLIVRSFMTPSIAALLGRWFWWPLRVRSRPARTPTVPSETQPAGRPLA

MSSDRLG

>mtb:TBMG_00451 K06994 putative drug exporter of the RND superfamily | (GenBank) transmembrane transporter mmpL4 (A)

MSTKFANDSNTNARPEKPFIARMIHAFAVPIILGWLAVCVVVTVFVPSLEAVGQERSVSL
[truncated: 7,077,176 more chars]
