# Supplementary material for: Comprehensive Comparative Analysis of Cholesterol Catabolic Genes/Proteins in Mycobacterial Species
Source: Int J Mol Sci. 2019 Feb 27;20(5):1032. doi: 10.3390/ijms20051032 (PMC6429209; doi:10.3390/ijms20051032)
Supplement: Supplementary file 1 [file ijms-20-01032-s001.zip › Supplementary information/Supplementary Information.docx]

Article

Comprehensive comparative analysis of cholesterol catabolic genes/proteins in mycobacterial species

Rochelle van Wyk ^1^, Mari van Wyk ^1^, Samson Sitheni Mashele ^1^, David R Nelson ^2^, Khajamohiddin Syed ^3,^*

^1^ Unit for Drug Discovery Research, Department of Health Sciences, Faculty of Health and Environmental Sciences, Central University of Technology, Bloemfontein 9300, Free State, South Africa; rochellevanwyk0@gmail.com (R. vW.); marivanwyk764@gmail.com (M. vW.); smashele@cut.ac.za (S.S.M.)

^2^ Department of Microbiology, Immunology and Biochemistry, University of Tennessee Health Science Center, Memphis, TN 38163, USA; drnelson1@gmail.com (D.R.N.)

^3^ Department of Biochemistry and Microbiology, Faculty of Science and Agriculture, University of Zululand, KwaDlangezwa 3886, South Africa; khajamohiddinsyed@gmail.com (K.S.)

***** Correspondence: khajamohiddinsyed@gmail.com (K.S.)

*2.3.2. Excel program for extracting KEGG BLAST data*

To extract the required data from the BLAST output data obtained from the KEGG database, an Excel program written in an Excel worksheet was used. The generated program is presented below:

Sheet/tab 1: Original Data

Cell A2-A80 000:

Copy the output data from KEGG database into cell A1 to A80 000 (depending on the size of the data).

Cell B2-B80 000:

*=IF(ISNUMBER(SEARCH(">",A2)),"1",IF(ISNUMBER(SEARCH("Identities",A2)),"2",IF(ISNUMBER(SEARCH("Length",A2)),"3","N")))*

Determines if the character “>”, the words “Identities” or “Length” are present in the data in column A.

For “>”, return the number “1” (a “1” will populate the next column).

For “Identities”, return the number “2” (a “2” will populate the next column).

For “Length”, return the number “3” (a “3” will populate the next column).

Else, if none of the above is present, return the letter “N” (an “N” will populate the next column).

Cell C2-C80 000:

*=IF(B2="1",IF(B3="3",1,IF(B4="3",2,IF(B5="3",3,IF(B6="3",4,"many")))),"")*

Determine the number of rows the specific record consists of, up to a maximum of four lines. If the record is longer than four lines, the word “many” will be displayed, indicating that manual investigation and entry are required.

Cell D2-D80 000:

*=IF(B2="1", A2, "")*

If valid data to be filtered contains “>” in Column A, then display the data in this row.

Cell E2:E80 000:

*=IF(B2="2", A2, "")*

If valid data to be filtered contain “Identifies” in Column A, then display the data in this row.

Cell F2-F80 000:

*=IF(ISNUMBER(SEARCH(">",D2)),LEFT(D2, LEN(D2)-5),D2)*

If this row in Column D includes the character “>”, then it displays the first characters, less 5 than the length of Column D.

Cell G2-G80 000:

*=IF(C2=1,F2,IF(C2=2,(F2&""&A3),IF(C2=3,(F2&""&A3&" "&A4),IF(C2=4,(F2&""&A3&" "&A4&" "&A5),""))))*

Combines the data into one cell to include all separate rows with valid data.

Cell H2 - O80 000:

Find the position of the first “:”, first “ “, total length, first “(“, first “%”, second “(“, second “%” to determine the “Org Code”, “Code”, “Name”, “Identity” and “Positive” for *Final Data Blanks* sheet/tab (Columns O to S).

Sheet/tab 2: Final Data Blanks

“Unformatted” column is populated if valid data is present in the corresponding cell in the Original Data sheet/tab.

“Org Code” obtained from Original Data sheet: Column O.

“Code” obtained from Original Data: Column P.

“Name” obtained from Original Data: Column Q and multiple spaces filtered.

“Identity” obtained from Original Data: Column R.

“Positives” obtained from Original Data: Column S.

Sheet/tab 3: Final Data

The data in columns B to F in the “Final Data Blanks” sheet/tab were copied and pasted into the “Final Data” sheet/tab and sorted alphabetically according to the first column (Org Code) – this was just to make the copying into the BLAST sheet easier. The data was also copied and transpose pasted to display it horizontally in order to make copying easier.

Validation

Before using this program, validation was performed with three different datasets and continuous checks were carried out throughout the use of the program.

**Table S1.** Genes predicted to be specifically required for growth on cholesterol [26].

| **Gene** | **Location** | **Enzyme** |
| --- | --- | --- |
| *ppiA* | *Rv0009* | iron-regulated peptidyl-prolyl cis-trans isomerase A |
| *ptbB* | *Rv0153c* | phosphotyrosine protein phosphatase PTPB (protein-tyrosine-phosphatase) (PTPase) |
| *mmpL11* | *Rv0202c* | transmembrane transport protein MmpL11 |
| *fadE5* | *Rv0244c* | acyl-CoA dehydrogenase |
| *mgtE* | *Rv0362* | Mg2+ transport transmembrane protein MgtE |
| *metZ* | *Rv0391* | O-succinylhomoserine sulfhydrylase |
| *mmpL4* | *Rv0450c* | transmembrane transport protein MmpL4 |
|  | *Rv0485* | transcriptional regulatory protein |
|  | *Rv0495c* | HP |
| *mkl* | *Rv0655* | ribonucleotide ABC transporter ATP-binding protein |
| *pqqE* | *Rv0693* | coenzyme PQQ synthesis protein E |
| *lldD1* | *Rv0694* | L-lactate dehydrogenase (cytochrome) LldD1 |
|  | *Rv0695* | HP |
|  | *Rv0696* | membrane sugar transferase |
| *adhB* | *Rv0761c* | zinc-containing alcohol dehydrogenase NAD dependent ADHB |
|  | *Rv0805* | HP |
|  | *Rv0876c* | transmembrane protein |
| *echA9* | *Rv1071c* | 3-hydroxyisobutyryl-CoA hydrolase |
|  | *Rv1084* | HP |
|  | *Rv1096* | glycosyl hydrolase |
|  | *Rv1129c* | transcriptional regulator protein |
|  | *Rv1130* | HP |
| *gltA1* | *Rv1131* | citrate synthase |
| *mmpL10* | *Rv1183* | transmembrane transport protein MmpL10 |
| *fadD36* | *Rv1193* | acyl-CoA synthetase |
|  | *Rv1428c* | HP |
|  | *Rv1432* | dehydrogenase |
| *bcpB* | *Rv1608c* | peroxidoxin BcpB |
|  | *Rv1626* | two-component system transcriptional regulator |
|  | *Rv1627c* | lipid-transfer protein |
|  | *Rv1798* | HP |
|  | *Rv1906c* | HP |
|  | *Rv1919c* | HP |
| *mce3R* | *Rv1963c* | transcriptional repressor (probably TETR-family) MCE3R |
| *pks12* | *Rv2048c* | polyketide synthase pks12 |
|  | *Rv2118c* | RNA methyltransferase |
|  | *Rv2206* | transmembrane protein |
|  | *Rv2239c* | HP |
| *eis* | *Rv2416c* | HP |
| *tig* | *Rv2462c* | trigger factor |
|  | *Rv2506* | TetR family transcriptional regulator |
|  | *Rv2668* | HP |
|  | *Rv2681* | HP |
| *arsA* | *Rv2684* | arsenic-transport integral membrane protein ArsA |
| *sigB* | *Rv2710* | RNA polymerase sigma factor SigB |
|  | *Rv2799* | HP |
| *pknI* | *Rv2914c* | transmembrane serine/threonine-protein kinase I |
| *mutT1* | *Rv2985* | hydrolase MutT1 |
|  | *Rv3050c* | AsnC family transcriptional regulator |
| *fadE25* | *Rv3274c* | acyl-CoA dehydrogenase FADE25 |
| *gcp* | *Rv3419c* | putative DNA-binding/iron metalloprotein/AP endonuclease |
|  | *Rv3421c* | HP |
|  | *Rv3492c* | CHP MCE associated protein |
|  | *Rv3493c* | CHP MCE associated protein |
| *mce4F* | *Rv3494c* | Mce4 transport system |
| *mce4E / lprN* | *Rv3495c* | Mce4 transport system |
| *mce4D* | *Rv3496c* | Mce4 transport system |
| *mce4C* | *Rv3497c* | Mce4 transport system |
| *mce4B* | *Rv3498c* | Mce4 transport system |
| *mce4A* | *Rv3499c* | Mce4 transport system |
| *yrb4B / YrbE4B / supB* | *Rv3500c* | possible ABC transporter (Sterol uptake permease subunit) |
| *yrb4A / YrbE4A / supA* | *Rv3501c* | possible ABC transporter (Sterol uptake permease subunit) |
| *hsd4A* | *Rv3502c* | 17β-hydroxysteroid dehydrogenase (17β-HSD) |
| *fadD19* | *Rv3515c* | probable fatty-acid-CoA ligase |
| *kshA* | *Rv3526* | kerosteroid-9α-hydroxylase, oxygenase |
|  | *Rv3531c* | hypothetical protein |
| *hsaF* | *Rv3534c* | probable 4-hydroxy-2-oxovalerate aldolase / 4-hydroxy-2-ketovalerate aldolase |
| *hsaE* | *Rv3536c* | probable hydratase / 2-hydroxypentadienoate hydratase |
| *kstD* | *Rv3537* | 3-ketosteroid-Δ1-dehydrogenase (Δ1-KSTD) |
| *ltp2* | *Rv3540c* | probable ketoacyl-CoA thiolase |
|  | *Rv3542c* | CHP / putative enoyl-CoA hydratase |
| *fadE29* | *Rv3543c* | probable acyl-CoA dehydrogenase |
| *fadE28* | *Rv3544c* | probable acyl-CoA dehydrogenase |
| *cyp125* | *Rv3545c* | cytochrome P450 |
| *fadA5* | *Rv3546* | acetoacetyl-CoA thiolase |
|  | *Rv3548c* | probable short chain dehydrogenase/reductase |
|  | *Rv3549c* | probable short chain dehydrogenase/reductase |
| *ipdA* | *Rv3551* | ATP-dependent CoA transferase α subunit |
|  | *Rv3553* | possible oxidoreductase / 2-nitropropane dioxygenase |
|  | *Rv3559c* | probable oxidoreductase |
| *fadE30* | *Rv3560c* | probable acyl-CoA dehydrogenase |
| *fadD3* | *Rv3561* | acyl-CoA synthetase (AMP forming) |
| *fadE32* | *Rv3563* | probable acyl-CoA dehydrogenase |
| *fadE33* | *Rv3564* | probable acyl-CoA dehydrogenase |
| *hsaC* | *Rv3568c* | 3,4-DHSA dioxygenase |
| *hsaD* | *Rv3569c* | 4,9-DHSA hydrolase |
| *hsaA* | *Rv3570c* | 3-hydroxy-9,10-seconandrost-1,3,5(10)-triene-9,17-dione hydroxylase (3-HSA hydroxylase, reductase) |
| *kshB* | *Rv3571* | ketosteroid-9α-hydroxylase, reductase |
|  | *Rv3572* | HP |
| *fadE34* | *Rv3573c* | probable acyl-CoA dehydrogenase |
|  | *Rv3575c* | transcriptional regulatory protein LacI-family |
|  | *Rv3779* | transmembrane protein alanine and leucine rich |
| *papA2* | *Rv3820c* | polyketide synthase associated protein PapA2 |
| *papA1* | *Rv3824c* | polyketide synthase associated protein |
| *pks2* | *Rv3825c* | polyketide synthase PKS2 |
| *sigM* | *Rv3911* | RNA polymerase sigma factor SigM |

Abbreviations: 3-HAS = 3-hydroxy-9,10-secoandrosta-1,3,5(10)-triene-9,17-dione; 3,4-DHSA = 3,4-dihydroxy-9,10-secoandrosta-1,3,5(10)-triene-9,17-dione; 4,9-DHSA hydrolase = 4,5-9,10-diseco-3-hydroxy-5,9,17-trioxoandrosta-1(10),2-dien-4-oic acid; 17β-HSD = 17β-hydroxysteroid dehydrogenase; Δ1-KSTD = 3-ketosteroid-Δ1-dehydrogenase; ABC = ATP-binding cassette; ADH = alcohol dehydrogenase; AMP = adenosine monophosphate; AP = apurinic/apyrimidinic; ATP = adenosine triphosphate; Bcp = bacterioferritin comigratory protein; CHP = conserved hypothetical protein; CoA = co-enzyme A; DNA = deoxyribonucleic acid; HP = hypothetical protein; LldD = L-lactate dehydrogenase; MCE = mammalian cell entry; MgtE = Mg2+ transport transmembrane protein; MmpL = *Mycobacterium* membrane protein laboratory; NAD = nicotinamide adenine dinucleotide; pks = polyketide synthase; PQQ = pyrrolo-quinoline quinone; PTP/PTPase = phosphotyrosine protein phosphatase /protein-tyrosine-phosphatase; RNA = ribonucleic acid; TetR = tetracycline repressor

**Table S2.** Genes proven to be essential for survival of *M. tuberculosis* in macrophage cells and in murine infection [19].

| **Gene** | **Location** | **Enzyme** |
| --- | --- | --- |
| *mce4E / lprN* | *Rv3495c* | Mce4 transport system |
| *mce4C* | *Rv3497c* | Mce4 transport system |
| *mce4A* | *Rv3499c* | Mce4 transport system |
| *yrb4A / YrbE4A / supA* | *Rv3501c* | possible ABC transporter (Sterol uptake permease subunit) |
| *hsd4A* | *Rv3502c* | 17β-hydroxysteroid dehydrogenase (17β-HSD) |
|  | *Rv3519* | CHP |
| *ltp3* | *Rv3523* | probable ketoacyl-CoA thiolase |
| *kshA* | *Rv3526* | kerosteroid-9α-hydroxylase, oxygenase |
|  | *Rv3527* | hypothetical protein (HP) |
| *hsaF* | *Rv3534c* | probable 4-hydroxy-2-oxovalerate aldolase / 4-hydroxy-2-ketovalerate aldolase |
| *ltp2* | *Rv3540c* | probable ketoacyl-CoA thiolase |
|  | *Rv3541c* | CHP / putative enoyl-CoA hydratase |
|  | *Rv3542c* | CHP / putative enoyl-CoA hydratase |
| *fadE28* | *Rv3544c* | probable acyl-CoA dehydrogenase |
| *cyp125* | *Rv3545c* | cytochrome P450 |
| *fadA5* | *Rv3546* | acetoacetyl-CoA thiolase |
| *ipdA* | *Rv3551* | ATP-dependent CoA transferase α subunit |
| *ipdB* | *Rv3552* | ATP-dependent CoA transferase β subunit |
| *fadA6* | *Rv3556c* | acetoacetyl-CoA thiolase |
| *fadE30* | *Rv3560c* | probable acyl-CoA dehydrogenase |
| *fadE32* | *Rv3563* | probable acyl-CoA dehydrogenase |
| *hsaC* | *Rv3568c* | 3,4-DHSA dioxygenase |
| *kshB* | *Rv3571* | ketosteroid-9α-hydroxylase, reductase |
| *kstR* | *Rv3574* | Tet-R transcriptional regulator (repressor) |

Abbreviations: 3,4-DHSA = 3,4-dihydroxy-9,10-secoandrosta-1,3,5(10)-triene-9,17-dione; 17β-HSD = 17β-hydroxysteroid dehydrogenase; ABC = ATP-binding cassette; ATP = adenosine triphosphate; CHP = conserved hypothetical protein; CoA = co-enzyme A; HP = hypothetical protein; MCE = mammalian cell entry; Tet-R = tetracycline repressor

**Table S3.** Genes predicted to be essential for survival of *M. tuberculosis* in macrophage cells and in murine infection [19].

| **Gene** | **Location** | **Enzyme** |
| --- | --- | --- |
| *kstD* | *Rv3537* | 3-ketosteroid-Δ1-dehydrogenase (Δ1-KSTD) |
| *fadE28* | *Rv3544c* | probable acyl-CoA dehydrogenase |
| *ipdA* | *Rv3551* | ATP-dependent CoA transferase α subunit |
| *fadA6* | *Rv3556c* | acetoacetyl-CoA thiolase |
| *fadE30* | *Rv3560c* | probable acyl-CoA dehydrogenase |
| *fadE32* | *Rv3563* | probable acyl-CoA dehydrogenase |
| *hsaD* | *Rv3569c* | 4,9-DHSA hydrolase |
| *hsaA* | *Rv3570c* | 3-hydroxy-9,10-seconandrost-1,3,5(10)-triene-9,17-dione hydroxylase (3-HSA hydroxylase, reductase) |

Abbreviations: 3-HSA = 3-hydroxy-9,10-secoandrosta-1,3,5(10)-triene-9,17-dione; 4,9-DHSA = 4,5-9,10-diseco-3-hydroxy-5,9,17-trioxoandrosta-1(10),2-dien-4-oic acid; Δ1-KSTD = 3-ketosteroid-Δ1-dehydrogenase; ATP = adenosine triphosphate; CoA = co-enzyme A

**Table S4.** Genes predicted to be involved in cholesterol catabolism [25].

| **Gene** | **Location** | **Enzyme** |
| --- | --- | --- |
| *choD* | *Rv3409c* | cholesterol oxidase |
| *mce4F* | *Rv3494c* | Mce4 transport system |
| *mce4E / lprN* | *Rv3495c* | Mce4 transport system |
| *mce4D* | *Rv3496c* | Mce4 transport system |
| *mce4C* | *Rv3497c* | Mce4 transport system |
| *mce4B* | *Rv3498c* | Mce4 transport system |
| *mce4A* | *Rv3499c* | Mce4 transport system |
| *yrb4B / YrbE4B / supB* | *Rv3500c* | possible ABC transporter (Sterol uptake permease subunit) |
| *yrb4A / YrbE4A / supA* | *Rv3501c* | possible ABC transporter (Sterol uptake permease subunit) |
| *hsd4A* | *Rv3502c* | 17β-hydroxysteroid dehydrogenase (17β-HSD) |
| *fadE26* | *Rv3504* | probable acyl-CoA dehydrogenase |
| *fadE27* | *Rv3505* | probable acyl-CoA dehydrogenase |
| *fadD17* | *Rv3506* | possible fatty-acid-CoA ligase |
| *fadD19* | *Rv3515c* | probable fatty-acid-CoA ligase |
| *echA19* | *Rv3516* | possible enoyl-CoA hydratase |
| *ltp4* | *Rv3522* | probable ketoacyl-CoA thiolase |
| *ltp3* | *Rv3523* | probable ketoacyl-CoA thiolase |
| *kshA* | *Rv3526* | kerosteroid-9α-hydroxylase, oxygenase |
| *hsaF* | *Rv3534c* | probable 4-hydroxy-2-oxovalerate aldolase / 4-hydroxy-2-ketovalerate aldolase |
| *hsaG* | *Rv3535c* | probable aldehyde dehydrogenase |
| *hsaE* | *Rv3536c* | probable hydratase / 2-hydroxypentadienoate hydratase |
| *kstD* | *Rv3537* | 3-ketosteroid-Δ1-dehydrogenase (Δ1-KSTD) |
| *hsd4B* | *Rv3538* | probable enoyl-CoA hydratase |
| *hsaB* | *Rv3567c* | 3-hydroxy-9,10-seconandrost-1,3,5(10)-triene-9,17-dione hydroxylase (3-HSA hydroxylase, reductase) |
| *hsaC* | *Rv3568c* | 3,4-DHSA dioxygenase |
| *hsaD* | *Rv3569c* | 4,9-DHSA hydrolase |
| *hsaA* | *Rv3570c* | 3-hydroxy-9,10-seconandrost-1,3,5(10)-triene-9,17-dione hydroxylase (3-HSA hydroxylase, reductase) |
| *kshB* | *Rv3571* | ketosteroid-9α-hydroxylase, reductase |

Abbreviations: 3-HSA = 3-hydroxy-9,10-secoandrosta-1,3,5(10)-triene-9,17-dione; 3,4-DHSA = 3,4-dihydroxy-9,10-secoandrosta-1,3,5(10)-triene-9,17-dione; 4,9-DHSA hydrolase = 4,5-9,10-diseco-3-hydroxy-5,9,17-trioxoandrosta-1(10),2-dien-4-oic acid; 17β-HSD = 17β-hydroxysteroid dehydrogenase; Δ1-KSTD = 3-ketosteroid-Δ1-dehydrogenase; ABC = ATP-binding cassette; CoA = co-enzyme A; MCE = mammalian cell entry

**Table S5.** Genes involved in cholesterol catabolism in *M. tuberculosis* H37Rv but not confirmed or predicted as essential [19, 22, 25, 29, 33, 40, 42].

| **Gene** | **Location** | **Enzyme** |
| --- | --- | --- |
| *fadD10* | *Rv0099* | fatty acid-CoA synthase |
| *fadB2* | *Rv0468* | hydroxybutyryl-CoA dehydrogenase |
|  | *Rv1106c* | 3β-hydroxysteroid dehydrogenase (3β-HSD) |
| *mbtN (fadE14)* | *Rv1346* | acyl-CoA dehydrogenase |
| *fadB3* | *Rv1715* | hydroxybutyryl-CoA dehydrogenase |
| *fadD9* | *Rv2590* | fatty acid-CoA synthase |
| *fadE22* | *Rv3061c* | acyl-CoA dehydrogenase |
| *fadE24* | *Rv3139* | acyl-CoA dehydrogenase |
| *fadE23* | *Rv3140* | acyl-CoA dehydrogenase |
| *fdxD* | *Rv3503c* | probable ferredoxin |
| *PE PGRS53* | *Rv3507* | PE PGRS family |
| *PE PGRS54* | *Rv3508* | PE PGRS family |
| *ilvX* | *Rv3509c* | probable acetohydroxy-acid synthase |
|  | *Rv3510c* | CHP |
| *PE PGRS55* | *Rv3511* | PE PGRS family |
| *PE PGRS56* | *Rv3512* | PE PGRS family |
| *fadD18* | *Rv3513c* | possible fatty-acid-CoA ligase |
| *PE PGRS57* | *Rv3514* | PE PGRS family |
| *whiB3* | *Rv3517* | conserved hypothetical protein (CHP) / transcription factor |
| *cyp142* | *Rv3518c* | cytochrome P450 |
|  | *Rv3520c* | coenzyme F420-dependent oxidoreductase |
|  | *Rv3521* | CHP |
|  | *Rv3524* | probable conserved membrane protein |
|  | *Rv3525c* | possible siderophore binding protein |
|  | *Rv3528c* | HP |
|  | *Rv3529c* | CHP |
|  | *Rv3530c* | possible oxidoreductase |
| *PPE61* | *Rv3532* | PPE family |
| *PPE62* | *Rv3533c* | PPE family |
| *PPE63* | *Rv3539* | PE |
|  | *Rv3547* | CHP |
| *echA20* | *Rv3550* | possible enoyl-CoA hydratase |
| *fdxB* | *Rv3554* | possible electron transfer protein / ferredoxin |
|  | *Rv3555c* | CHP |
| *kstR2* | *Rv3557c* | Tet-R transcriptional regulator (repressor) |
| *PPE64* | *Rv3558* | PPE |
| *fadE31* | *Rv3562* | probable acyl-CoA dehydrogenase |
| *aspB* | *Rv3565* | possible aspartate aminotransferase |
|  | *Rv3566A* | CHP |
| *nhoA / nat* | *Rv3566c* | arylamine N-acetyltransferase |

Abbreviations: 3β-HSD = 3β-hydroxysteroid dehydrogenase; CHP = conserved hypothetical protein; CoA = co-enzyme A; HP = hypothetical protein; PE = protein family with highly conserved Proline-Glutamate residues near the start of their encoded proteins; PGRS = polymorphic GC-rich-repetitive sequence; PPE = protein family with highly conserved Proline-Proline-Glutamate; Tet-R = tetracycline repressor
